# Sustainable Peptide Synthesis Enabled by a Transient Protecting Group

**DOI:** 10.1002/anie.202003676

**Published:** 2020-05-29

**Authors:** Sascha Knauer, Niklas Koch, Christina Uth, Reinhard Meusinger, Olga Avrutina, Harald Kolmar

**Affiliations:** ^1^ Sulfotools GmbH In der Niederwiesen 24a 64291 Darmstadt Germany; ^2^ Institute for Organic Chemistry and Biochemistry TU Darmstadt Alarich-Weiss-Strasse 4 64287 Darmstadt Germany

**Keywords:** green chemistry, peptide synthesis, protecting groups, sustainability, water-based peptide synthesis

## Abstract

The growing interest in synthetic peptides has prompted the development of viable methods for their sustainable production. Currently, large amounts of toxic solvents are required for peptide assembly from protected building blocks, and switching to water as a reaction medium remains a major hurdle in peptide chemistry. We report an aqueous solid‐phase peptide synthesis strategy that is based on a water‐compatible 2,7‐disulfo‐9‐fluorenylmethoxycarbonyl (Smoc) protecting group. This approach enables peptide assembly under aqueous conditions, real‐time monitoring of building block coupling, and efficient postsynthetic purification. The procedure for the synthesis of all natural and several non‐natural Smoc‐protected amino acids is described, as well as the assembly of 22 peptide sequences and the fundamental issues of SPPS, including the protecting group strategy, coupling and cleavage efficiency, stability under aqueous conditions, and crucial side reactions.

## Graphical abstract

**Peptide synthesis goes green**: A water‐compatible 2,7‐disulfo‐9‐fluorenylmethoxycarbonyl (Smoc) protecting group has been developed that enables solid‐phase peptide synthesis under aqueous conditions as well as efficient postsynthetic purification of the peptides. This protecting group is fluorescent both when attached to an N_α_ atom and when cleaved and so also allows real‐time monitoring of building block coupling.

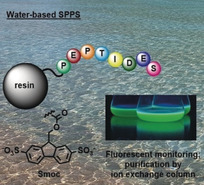

## Introduction

Peptides, natural biopolymers that comprise a chain of up to 100 covalently linked amino acids, are elementary components in all living systems and regulate many biological processes. Currently, synthetic peptides are produced industrially for the treatment of cancer, diabetes, as well as cardiovascular and neurodegenerative diseases,^1^, ^2^, ^3^ are used in cosmetic,^4^, ^5^ diagnostic, and medical technology products, as well as in veterinary medicine, agrochemistry, or as dietary supplements.^1^, ^6^

The history of peptide synthesis dates back to the turn of the twentieth century, when Curtius^7^ and then Fisher^8^, ^9^ presented early procedures for the chemical assembly of oligopeptides, thus having started “a systematic attack on a field of natural substances that have previously been avoided by chemists.”^10^ After the introduction of readily cleavable protecting groups that enabled the condensation of multifunctional amino acids,^11^, ^12^ solution‐phase synthetic methods allowed the production of impressively long peptidic molecules.^13^ However, the real breakthrough in the field of synthetic peptides was achieved when Merrifield presented his concept of solid‐phase peptide synthesis (SPPS) in 1963.^14^ Since then, peptide chemistry has been significantly developed in terms of more and more efficient synthetic procedures, having taken advantage of automation,^15^, ^16^ novel protection strategies,^17^ microwave assistance,^18^ improved isolation and purification methods, as well as advanced analytics. As a result, the field of synthetic peptides keeps rapidly growing, and the market of peptide pharmaceutics amounts to about 15 billion USD.^2^ Over the last decade, more than 100 peptides have been approved as drugs or for diagnostic applications.^2^, ^3^

To date, peptide synthesis has gained almost optimal efficiency in terms of coupling yield and duration. However, solvent consumption, which has been recognized as an economic and environmental issue for over a decade, makes SPPS one of the most inefficient approaches known.^19^, ^20^, ^21^, ^22^ In terms of sustainability, an ideal procedure could be outlined as a fully automated process with no waste of organic solvent, reduced consumption of all chemicals, and shortened reaction times, with online monitoring of each reaction step if required, and with successive automated purification.^23^ Recent advantages in microwave‐assisted SPPS successfully helped with the task of reduced chemical expenditure.^24^, ^25^ However, even when scaled down, the consumption of dimethylformamide (DMF) and *N*‐methyl‐2‐pyrrolidone (NMP), which are currently the solvents of choice in SPPS, is undesirable as they are classified by the European Reach Regulation as substances of very high concern because of their carcinogenic, mutagenic, or toxicity properties towards reproduction (CMR). Although a sustainable, green alternative to the existing SPPS scheme is highly desirable,^26^ switching to water—the natural medium for peptide biosynthesis—remains one of the most fascinating unsolved problems in organic chemistry.^27^

A number of alternative approaches have been proposed to overcome this hurdle, among them peptide synthesis in less harmful organic solvents or those referred to as “green” ones (cyrene, diethyl carbonate, anisole, etc.),^28^ solvent‐free methods based on ball‐milling, and the application of some water‐compatible systems with appropriate protecting groups and solvents.^21^, ^22^, ^27^, ^29^, ^30^, ^31^, ^32^, ^33^, ^34^, ^35^, ^36^, ^37^ However, the scope of these procedures is often limited in terms of peptide length, choice of amino acid derivatives, orthogonality, and compatibility with the desired synthetic step. Indeed, the longest sequence accessed by these methods consists of ten amino acids.^21^, ^22^, ^27^, ^29^, ^30^, ^31^, ^32^, ^33^, ^34^, ^35^, ^36^, ^37^

Effective online monitoring at each synthetic step is another challenge in SPPS, as the final yield strongly depends on the coupling efficiency. Of the two main approaches for the solid‐phase assembly of peptides, which are named according to the respective transient protection groups as Boc‐ and Fmoc‐SPPS (Boc=*tert*‐butyloxycarbonyl, Fmoc=9‐fluorenylmethoxycarbonyl), the latter allows one to follow an N‐terminal deprotection, but only when the coupling step has already been accomplished and, therefore, cannot be repeated when there is insufficient coupling.^38^ To date, no reliable method for inline SPPS monitoring has been reported. In addition, efficient purification is one of the bottlenecks of peptide production, which mainly relies on reversed‐phase high‐performance liquid chromatography (HPLC) and requires large amounts of organic solvents and a tremendous investment of time.

**Figure 1:**
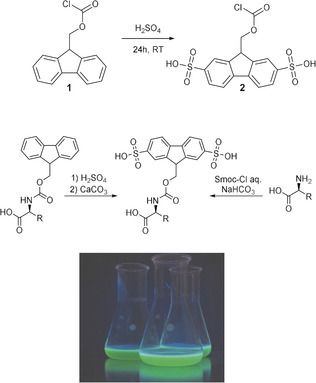
Top: Synthesis of Smoc‐chloride **2** from Fmoc‐chloride **1**. Middle: synthetic access to *N*‐Smoc‐amino acids. Bottom: Solution of **2** under irradiation with UV light.

**Scheme 1:**
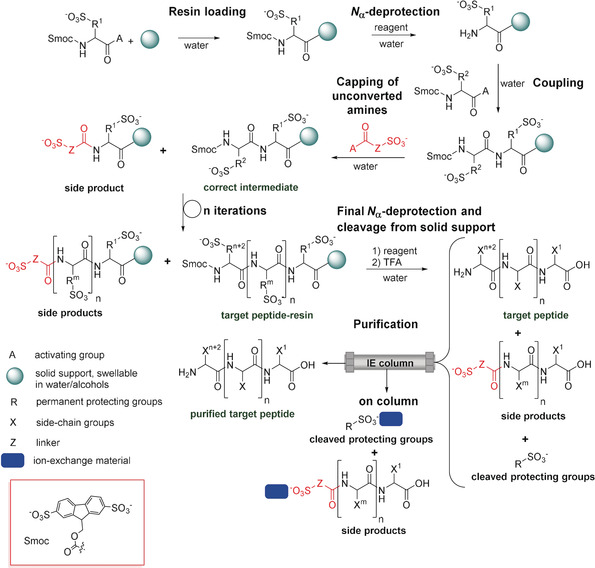
General Scheme of ASPPS.

Herein, we present a novel approach to peptide assembly in water as a sustainable alternative to the state‐of‐the‐art process (Scheme 1). The cornerstone of our aqueous solid‐phase peptide synthesis (ASPPS) is a water‐compatible 2,7‐disulfo‐9‐fluorenylmethoxycarbonyl (Smoc) protecting group that combines a fluorenyl system with charged sulfonic pendants (Figure 1). Amino acids bearing this group at the N‐terminus possess both high water solubility and pronounced fluorescence, which allows for real‐time monitoring (Figure 1).

## Results and Discussion

Our study started with the conversion of Fmoc‐chloride **1** into Smoc‐chloride **2** by treatment with oleum (Figure 1, see also Section 1.1 in the Supporting Information). Smoc‐protected building blocks (**3**–**32**, Table 2) were generated in two ways.

The direct conversion of *N*_α_‐Fmoc‐amino acids lacking functional side chains to generate the respective Smoc derivatives (**3**, **4**, **7**, **12**, **16**, **21**, **30**, **31**; Figure 1) was achieved by treatment with sulfuric acid followed by neutralization of any excess with calcium carbonate (see Section 1.2 in the Supporting Information). The second route relying on the use of Smoc‐Cl **2** (Figure 1) to address the free N‐terminus was successfully applied to all canonical amino acids as well as to a number of non‐natural building blocks, and gave the desired *N*_α_‐Smoc counterparts in high yields (Table 2, see also Section 1.2 in the Supporting Information). The moderate conversion obtained for 2‐aminoisobutyric acid (Aib, **32**) was most likely due to steric hindrance. NMR studies performed with *N*_α_‐Smoc‐amino acids **3**–**32** confirmed their structures (see Section 1.3 in the Supporting Information).

For amino acids with functional side chains, either a combination of the *N*_α_‐Smoc group with the standard side‐chain protecting groups or, with the exception of Lys, Glu, Asp, Ser, Thr, and Cys, were used unprotected in aqueous SPPS (Figure 1, Tables 1 and 2; see also Section 1.2 in the Supporting Information). Hence, compared to the state‐of‐the‐art peptide‐synthetic methods (Table 1), ASPPS offers significant advantages in terms of atom economy and reduced time required to remove side‐chain protecting groups.^39^, ^40^ Moreover, its application could be advantageous in view of preventing aggregation through side‐chain protecting groups. Indeed, avoiding bulky and hydrophobic moieties (e.g. trityl or 2,2,4,6,7‐pentamethyldihydrobenzofuran‐5‐sulfonyl (Pbf)) could be desirable for the assembly of “difficult” peptides bearing arginine, asparagine, or glutamine residues.

**Table 1:** Comparison of protecting schemes for Boc‐SPPS, Fmoc‐SPPS, and ASPPS.

Boc‐SPPS^[a]^	Fmoc‐SPPS	ASPPS
Boc‐Arg(Tos)‐OH	Fmoc‐Arg(Pbf)‐OH	Smoc‐Arg‐OH
Boc‐Asn(Trt)‐OH	Fmoc‐Asn(Trt)‐OH	Smoc‐Asn‐OH
Boc‐Asp(OBzl)‐OH	Fmoc‐Asp(OtBu)‐OH	Smoc‐Asp(OtBu)‐OH
Boc‐Cys(Acm)‐OH	Fmoc‐Cys(Trt)‐OH	Smoc‐Cys(Trt)‐OH
Boc‐Gln(Trt)‐OH	Fmoc‐Gln(Trt)‐OH	Smoc‐Gln‐OH
Boc‐Glu(OBzl)‐OH	Fmoc‐Glu(OtBu)‐OH	Smoc‐Glu(OtBu)‐OH
Boc‐His(Dnp)‐OH	Fmoc‐His(Trt)‐OH	Smoc‐His(Trt)‐OH^[a]^
Boc‐Lys(Cbz)‐OH	Fmoc‐Lys(Boc)‐OH	Smoc‐Lys(Boc)‐OH
Boc‐Ser(Bzl)‐OH	Fmoc‐Ser(tBu)‐OH	Smoc‐Ser(OtBu)‐OH
Boc‐Thr(Bzl)‐OH	Fmoc‐Thr(tBu)‐OH	Smoc‐Thr(OtBu)‐OH
Boc‐Trp(For)‐OH	Fmoc‐Trp(Boc)‐OH	Smoc‐Trp(Boc)‐OH^[b]^
Boc‐Tyr(Bzl)‐OH	Fmoc‐Tyr(tBu)‐OH	Smoc‐Tyr(OtBu)‐OH

[a] Smoc‐His was also synthesized without side‐chain protection. [b] Smoc‐Trp was also synthesized without side‐chain protection. Acm=acetamidomethyl, Bzl=benzyl, Cbz=benzyloxycarbonyl, Dnp=2,4‐dinitrophenyl, For=formyl, OBzl=benzyl ester, Tos=tosyl, Trt=trityl.

**Table 2:** Synthesized *N*‐Smoc‐amino acids.

No.	Abbreviation	Yield [%]
**3**	Smoc‐l‐Ala‐OH	87.2
**4**	Smoc‐d‐Ala‐OH	86.9
**5**	Smoc‐l‐Arg‐OH	85.7
**6**	Smoc‐l‐Arg(Pbf)‐OH	85.1
**7**	Smoc‐l‐Asn‐OH	90.4
**8**	Smoc‐l‐Asp(OtBu)‐OH	86.7
**9**	Smoc‐l‐Cys(Trt)‐OH	85.1
**10**	Smoc‐l‐Gln‐OH	90.8
**11**	Smoc‐l‐Glu(OtBu)‐OH	88.2
**12**	Smoc‐Gly‐OH	93.7
**13**	Smoc‐l‐His‐OH	92.4
**14**	Smoc‐l‐His(Trt)‐OH	86.6
**15**	Smoc‐l‐Ile‐OH	88.9
**16**	Smoc‐l‐Leu‐OH	90.6
**17**	Smoc‐d‐Leu‐OH	88.7
**18**	Smoc‐l‐Lys(Boc)‐OH	87.4
**19**	Smoc‐l‐Met‐OH	95.1
**20**	Smoc‐l‐Phe‐OH	93.7
**21**	Smoc‐l‐Pro‐OH	85.8
**22**	Smoc‐l‐Ser‐OH	90.0
**23**	Smoc‐l‐Ser(OtBu)‐OH	87.9
**24**	Smoc‐l‐Thr‐OH	92.2
**25**	Smoc‐l‐Thr(OtBu)‐OH	89.2
**26**	Smoc‐l‐Trp‐OH	90.7
**27**	Smoc‐l‐Trp(Boc)‐OH	86.9
**28**	Smoc‐l‐Tyr‐OH	89.7
**29**	Smoc‐l‐Tyr(tBu)‐OH	91.4
**30**	Smoc‐l‐Val‐OH	87.2
**31**	Smoc‐β‐Ala‐OH	92.5
**32**	Smoc‐Aib‐OH	57.8

During the course of the solid‐phase peptide assembly, the *N*_α_‐protecting group is cleaved under mild conditions at the beginning of each cycle (Scheme 1) to retain orthogonality with the side chains.^40^ Therefore, we examined various aqueous bases or their solutions in polar solvents for their ability to cleave an Smoc group (see Section 1.4 in the Supporting Information). We found that aqueous piperidine, piperazine, sodium hydroxide, ethanolamine, and ammonia readily liberated the N‐terminus within 5 minutes at ambient temperature together with the formation of the respective disulfonated dibenzofulvene and the products of water or base addition (see Figure S33).

After the conditions for the Smoc group cleavage had been thoroughly examined, the stability of the *N*_α_‐Smoc‐amino acids during aqueous peptide synthesis was studied by incubating Smoc‐Arg‐OH (**5**), Smoc‐Ile‐OH (**15**), Smoc‐Phe‐OH (**20**), Smoc‐Pro‐OH (**21**), and Smoc‐Ser‐OH (**22**) with 3 equiv aqueous NaHCO_3_. HPLC analysis after 7, 14, and 21 days showed that the examined constructs were sufficiently stable (see Section 1.5 in the Supporting Information). The fact that all the Smoc‐protected amino acids were soluble and stable at concentrations typically used in peptide synthesizers, makes them suitable building blocks for automated SPPS.

Since the early years of peptide synthesis, it has been generally accepted that the formation of a peptide bond necessitates anhydrous coupling conditions as otherwise the required active species are hydrolyzed and the equilibrium is shifted towards the initial reagents. However, in water the acidity of carboxylic acids is increased compared to that in polar aprotic solvents commonly applied in peptide synthesis.^41^ Therefore, the acidic proton of the carboxylic group is transferred to a water molecule, thus increasing the reactivity of the carboxylate ion towards carbodiimides.^42^ In the present study, we evaluated different coupling reagents and active ester‐forming compounds in the synthesis of the dipeptide Smoc‐l‐Pro‐l‐Tyr‐OMe (see Section 1.6 in the Supporting Information) at ambient temperature (see Table S11). In our hands, the combination of EDC‐HCl/Oxyma^43^ (EDC=1‐ethyl‐3‐(3‐dimethylaminopropyl)carbodiimide in the presence of sodium bicarbonate as a general base gave 90.7 % conversion after 25 min in water at ambient temperature. Interestingly, in 30 % aqueous acetonitrile, ethanol, or 2‐propanol, the reaction yield increased to almost quantitative (see Section 1.6 in the Supporting Information).

With all the *N*_α_‐Smoc‐protected natural and some noncanonical amino acids possessing significant stability in basic aqueous solutions, but readily losing their terminal amino protection upon mild treatment with particular water‐soluble bases, we expanded the classic Merrifield procedure^14^, ^40^ towards an aqueous solid‐phase peptide synthesis (ASPPS, Scheme 1). As a solid support, we used a water‐swellable resin from the repertoire of commercially available SPPS polymers (e.g. Tentagel, ChemMatrix, PEGA, etc.).^44^ After loading of the first amino acid (Scheme 1), when a commercial preloaded resin was not available, cleavage of the Smoc group was conducted with aqueous NaOH, NH_3_, piperazine, or ethanolamine. Coupling of the next activated amino acid was then performed, followed, if required, by capping any free amines with special sulfo‐tags based on sulfoacetic acid or similar compounds (Scheme 1). This step ensured labeling of all the remaining free amines and thus allowing for easy purification after global cleavage. This deprotection‐coupling‐capping cycle was repeated until the desired length of peptide had been reached.

In the present study, we assembled 22 peptides of different lengths and complexity, most of them bioactive molecules used in cosmetic or pharmaceutical applications (Table 3).

**Table 3:** Overview of the synthesized peptides.

No.	Peptide sequence	Solvent	Yield [mg] (%)^[a]^
**48**	H‐AGELS‐NH_2_ (pentapeptide‐31)	water	13.7 (57.7)
**49**	H‐GPQGPQ‐OH (hexapeptide‐9)	water	9.2 (38.7)
**50**	H‐EEMQRR‐HN_2_ (hexapeptide‐3)	water	21.7 (51.3)
**51**	Ac‐EEMQRR‐HN_2_ (acetylhexapeptide‐3)	water	18.7 (42.1)
**52**	Leu‐Enkephalin amide H‐YGGFL‐NH_2_	water	19.6 (70.8)
**53**	Met‐Enkephalin H‐YGGFM‐OH	30 % MeCN_aq_	9 (63)
**54**	Leu‐Enkephalin H‐YGGFL‐OH	30 % MeCN_aq_	10.3 (71.8)
**55**	(ACP) 65‐74 H‐VQAAIDYING‐OH	50 % MeCN_aq_	18 (36)
**56**	(ACP) 65‐74 H‐VQAAIDYING‐NH_2_	50 % MeCN_aq_	23 (46)
**57**	H‐GPRP‐OH	water	8 (38)
**58**	Smoc‐VVIA‐NH_2_	water	26 (67)
**59**	Smoc‐DIIW‐OH	water	22 (50)
**60**	Smoc‐E(OtBu)K(Boc)R(Pbf)S(tBu)C(Trt)‐OH	50 % MeCN_aq_	18 (44)
**61**	H‐CYEIS‐NH_2_	30 % MeCN_aq_	15 (48.9)
**62**	H‐ANKPG‐NH_2_	30 % MeCN_aq_	13 (35.6)
**63**	Pal‐GHK‐OH^[b]^	20 % EtOH_aq_	14.1 (54.2)
**64**	Pal‐GQPR‐OH^[b]^	20 % EtOH_aq_	18.7 (59.7)
**65**	H‐GPRPA‐NH_2_ Vialox	30 % MeCN_aq_	12 (48.4)
**66**	oxytocin	30 % MeCN_aq_	18 (35.7)
**67**	vasopressin	30 % MeCN_aq_	20 (39.7)
**68**	heptaarginine	30 % MeCN_aq_	23 (41.4)
**69**	leuphasyl	20 % MeCN_aq_	17.3 (57.8)

[a] Calculated from the average loading. [b] Pal: Palmitic acid.

**Figure 2:**
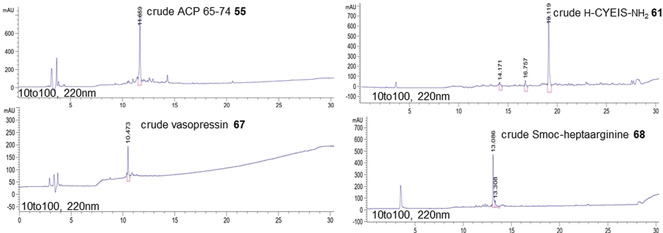
Selected RP‐HPLC traces of crude **55** (a standard validation sequence), **61** (a model peptide for racemization studies), **67** (natural peptide vasopressin), and **68** (an arginine‐rich peptide). The HPLC traces show peptides prior to any purification step.

All peptides were assembled manually on either a commercially available ChemMatrix H‐Rink amide or on a preloaded HMPB‐ChemMatrix resin. The ChemMatrix H‐Rink amide resin was loaded in a double coupling using a solution of *N*_α_‐Smoc‐amino acid (3 equiv), EDC‐HCl (**37**; 5.5 equiv), and Oxyma (**39**; 3 equiv) in aqueous NaHCO_3_. Smoc was cleaved with 1 m NaOH, 25 % aqueous ethanolamine, or 5–10 % aqueous piperazine. Coupling of the amino acids was performed in a similar manner as the loading of the first amino acid (for details see Section 1.7 in the Supporting Information). As expected, the lack of side‐chain protection significantly reduced the duration of the global cleavage. Indeed, even in the case of peptides with multiple arginine residues^39^ (**50**, **51**, **68**), the cleavage time did not exceed 1 h, and the purity of the crude peptide was reasonable (Figure 2).

**Figure 3:**
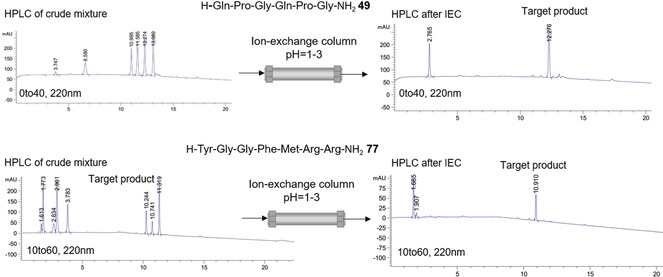
Sulfo‐tag purification concept. Two model peptides, **49** and **77**, were assembled considering the deficiencies of all the amino acids to facilitate the formation of by‐products. Peptide **77** was synthesized in a classic DMF‐based synthesis to show applicability in Fmoc‐SPPS.

To verify our “sulfo‐tag” purification approach we synthesized two model peptides, **49** and **77**, having considered the deficiencies of all the amino acids in coupling (0.95 equiv to the loading of the previous amino acid) to facilitate the formation of wrong, shortened sequences (Figure 3 and see Section 1.8 in the Supporting Information) After global acidolytic cleavage, all the labeled side products, the “correct” peptide of desired length, and the free side‐chain protecting groups resided in solution. The mixture was loaded onto an ion‐exchange column, and only the desired peptide lacking a sulfo‐tag at the N‐terminus eluted quickly; all the negatively charged sulfonated impurities were retained by the column (Figure 3). Since this is a fast, easy‐to‐handle, and sustainable purification option, ion‐exchange chromatography could serve as a pre‐purification step to remove most of the side products, thus facilitating subsequent HPLC separation.

In addition, two important side reactions commonly occurring in SPPS were studied. Racemization upon ASPPS was assessed by the assembly of the two model peptides H‐CYEIS‐NH_2_ (**61**) and H‐ANKPG‐NH_2_ (**62**; see Section 1.9 in the Supporting Information). Briefly, the racemization of the amino acids during the ASPPS corresponded to that observed in Fmoc‐based SPPS in DMF. The formation of aspartimide, a well‐documented side reaction occurring frequently at Asn‐R or Asp‐R sites, where R is an amino acid such as Gly, Ala, or Ser,^45^, ^46^ was studied on four model peptides derived from peptide scorpion toxin II (H‐VKDGYI‐NH_2_ (**70**), H‐VK(d‐D)GYI‐NH_2_ (**71**), H‐VKNGYI‐NH_2_ (**72**) and H‐VK(β‐D)GYI‐NH_2_ (**73**). Our data (see Section 1.10 in the Supporting Information) clearly indicate that aspartimide formation in water is temperature‐dependent, with the ratio of the β‐ to an α‐product being 1:3 at 4 °C (Figure S78) and 2.5:1 at 40 °C. Therefore, the deprotection of sequences prone to aspartimide formation in water should be performed at reduced temperature. Interestingly, β‐branching is the only aspartimide side reaction detected in ASPPS.

**Figure 4:**
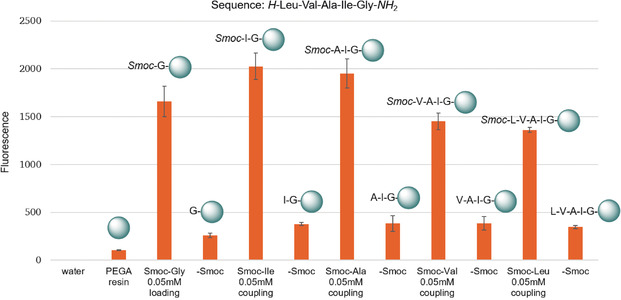
Fluorescence monitoring of the coupling and deprotection steps during ASPPS of the peptide H‐Leu‐Val‐Ala‐Ile‐Gly‐NH_2_.

An astonishing feature of the Smoc group is its fluorescence. Indeed, as a result of its electronic structure it possesses a distinct greenish glow (Figure 1), both when attached to an N_α_‐atom and when cleaved (see Sections 1.11 and 1.12 in the Supporting Information for details). These properties allow detection both in solution and when bound to the solid support, thus enabling for the first time a real‐time monitoring of both the coupling and deprotection steps. An assessment of the fluorescence at each coupling and deprotection step during the synthesis of the pentapeptide H‐LVAIG‐NH_2_ was performed by excitation at *λ*=280 nm and observing the emission at *λ*=340 nm (Figure 4, see also section 1.12 in the Supporting Information). The fluorescence allows the reaction steps to be distinguished. After cleavage of the Smoc group, the fluorescence adjusts to a baseline value with a specific auto‐fluorescence. After coupling of an *N*_α_‐Smoc‐amino acid, the fluorescence increases (Figure 4). The amino acids show intrinsically different fluorescence; this could be compensated by a normalization that takes into account the quantum yield of each amino acid.

## Conclusion

To summarize, we have developed a working concept for efficient aqueous solid‐phase peptide synthesis and demonstrated its applicability to the synthesis of 22 biologically active peptides. The coupling efficiency was assessed in water‐based systems by applying the respective *N*_α_‐Smoc‐amino acids and using different activation approaches. In our hands, several water‐compatible activating additives were found to be appropriate, with EDC‐HCl (**37**), Oxyma (**39**), and 2‐hydroxypyridine‐N‐oxide (HOPO; **40**) being the most efficient ones. Our experiments showed that although the coupling of amino acids in pure water gave peptides in reasonable yields and purity, the addition of organic co‐solvents enhanced the coupling performance significantly. Additional studies on the enantiomeric composition showed no increased racemization levels during the ASPPS process. The ionic properties of the Smoc protecting group gave rise to an elegant approach for the reliable purification of synthetic peptides. Merrifield had already shown that a monosulfonated Fmoc derivative could be applied for peptide isolation with ion‐exchange chromatography.^47^ This method was further optimized and integrated as a capping strategy into the ASPPS‐based peptide assembly. To that end, all the by‐products originating from incomplete coupling reactions are labeled with charged sulfo‐tags and can be easily removed by successive ion‐exchange chromatography after cleavage from the solid support. Our studies showed that sulfo‐tag capping could also be applied to Fmoc‐SPPS. This method allows tailoring of purification strategies depending on the required purity grade of the peptide. Moreover, the same method could be used to refine waste water (Figure S71).

It is important to mention that the development of specific, water‐optimized resins and respective linker systems still remains a challenge that must be addressed. However, this issue definitely does not apply to the current work. The next goal in the frame of this study is the development of automated procedures for the small‐ and large‐scale sustainable synthesis of peptides.

## Conflict of interest

The authors declare competing financial interests. S.K., H.K, and C.U. are the founders of Sulfotools GmbH, a small chemical company interested in aqueous peptide synthesis. N.K. is an employee of Sulfotools GmbH. O.A., S.K., H.K, and C.U. are named inventors on a patent application (WO 2016 050764) filed by the Technische Universität Darmstadt and Sulfotools GmbH on the aqueous peptide synthesis methodology described in this work. R.M. declares no competing financial interest.

## Conflict of interest

S.K. initiated, designed, and coordinated the project; S.K., C.U., N.K. designed the experiments, performed all the experiments and analysis; O.A., S.K. wrote the manuscript; R.M. performed NMR studies and acquisition. O.A. and H.K. advised on all aspects. All authors discussed the results and commented on the manuscript.

## Supporting information

As a service to our authors and readers, this journal provides supporting information supplied by the authors. Such materials are peer reviewed and may be re‐organized for online delivery, but are not copy‐edited or typeset. Technical support issues arising from supporting information (other than missing files) should be addressed to the authors.

Supplementary
