# Supplementary material for: Sustainable Peptide Synthesis Enabled by a Transient Protecting Group
Source: Angew Chem Int Ed Engl. 2020 May 29;59(31):12984–90. doi: 10.1002/anie.202003676 (PMC7496111; doi:10.1002/anie.202003676)
Supplement: Supplementary file 1 — Supplementary [file ANIE-59-12984-s001.pdf]

Angewandte Chemie
Eine Zeitschrift der Gesellschaft Deutscher Chemiker

Supporting Information

Sustainable Peptide Synthesis Enabled by a Transient Protecting Group

Sascha Knauer[+], Niklas Koch[+], Christina Uth[+], Reinhard Meusinger, Olga Avrutina, and Harald Kolmar**

anie_202003676_sm_miscellaneous_information.pdf

Supporting Information

Sustainable Peptide Synthesis Enabled by a Transient Protecting Group

Sascha Knauer,‡ Niklas Koch,‡ Christina Uth,‡ Reinhard Meusinger, Olga Avrutina, and Harald Kolmar**

Table of Contents

1. Synthesis 5
  1.1. Synthesis of 2,7-disulfo-9-fluorenylmethoxycarbonyl chloride (Smoc-Cl) 2 5
  1.2. Synthesis of N_{α}-Smoc amino acids 5
    1.2.1. General procedure 5
    1.2.2. Synthesis of Smoc-L-Ala-OH 3 5
    1.2.3. Synthesis of Smoc-D-Ala-OH 4 6
    1.2.4. Synthesis of Smoc-L-Arg-OH 5 7
    1.2.5. Synthesis of Smoc-L-Arg(Pbf)-OH 6 7
    1.2.6. Synthesis of Smoc-L-Asn-OH 7 8
    1.2.7. Synthesis of Smoc-L-Asp(OtBu)-OH 8 9
    1.2.8. Synthesis of Smoc-L-Cys(Trt)-OH 9 9
    1.2.9. Synthesis of Smoc-L-Gln-OH 10 10
    1.2.10. Synthesis of Smoc-L-Glu(OtBu)-OH 11 10
    1.2.11. Synthesis of Smoc-Gly-OH 12 11
    1.2.12. Synthesis of Smoc-L-His-OH 13 12
    1.2.13. Synthesis of Smoc-L-His(Trt)-OH 14 12
    1.2.14. Synthesis of Smoc-L-Ile-OH 15 13
    1.2.15. Synthesis of Smoc-L-Leu-OH 16 13
    1.2.16. Synthesis of Smoc-D-Leu-OH 17 14
    1.2.17. Synthesis of Smoc-L-Lys(Boc)-OH 18 15
    1.2.18. Synthesis of Smoc-L-Met-OH 19 15
    1.2.19. Synthesis of Smoc-L-Phe-OH 20 16
    1.2.20. Synthesis of Smoc-L-Pro-OH 21 16
    1.2.21. Synthesis of Smoc-L-Ser-OH 22 17
    1.2.22. Synthesis of Smoc-L-Ser(tBu)-OH 23 18
    1.2.23. Synthesis of Smoc-L-Thr-OH 24 18
    1.2.24. Synthesis of Smoc-L-Thr(tBu)-OH 25 19
    1.2.25. Synthesis of Smoc-L-Trp-OH 26 19
    1.2.26. Synthesis of Smoc-L-Trp(Boc)-OH 27 20
    1.2.27. Synthesis of Smoc-L-Tyr-OH 28 21
    1.2.28. Synthesis of Smoc-L-Tyr(tBu)-OH 29 21
    1.2.29. Synthesis of Smoc-L-Val-OH 30 22
    1.2.30. Synthesis of Smoc-β-Ala-OH 31 22
    1.2.31. Synthesis of Smoc-Aib-OH 32 23
  1.3. NMR studies 23
  1.4. N_{α}-Smoc deprotection 39
  1.5. Stability of Smoc-protected amino acids 40

1.6. Coupling efficiency in water-based systems ... 40
1.7. Aqueous SPPS (ASPPS) of model peptides ... 51
1.7.1. General procedure for peptide synthesis ... 51
1.7.2. Synthesis of H-AGELS-NH_2 (Pentapeptide-31) 48 ... 51
1.7.3. Synthesis of H-GPQGPQ-OH (Hexapeptide-9) 49 ... 52
1.7.4. Synthesis of H-EEMQRR-NH_2 (Hexapeptide 3) 50 ... 52
1.7.5. Synthesis of Ac-EEMQRR-NH_2 (Acetyl-Hexapeptide 3) 51 ... 52
1.7.6. Synthesis of Leu-Enkephalin amide 52 ... 53
1.7.7. Synthesis of Met-Enkephalin: H-YGGFM-OH 53 ... 53
1.7.8. Synthesis of Leu-Enkephalin: H-YGGFL-OH 54 ... 54
1.7.9. Synthesis of Acyl-Carrier-Protein (ACP) 65-74 peptide: H-VQAAIDYING-OH 55 ... 54
1.7.10. Synthesis of Acyl-Carrier-Protein (ACP) 65-74: H-VQAAIDYING-NH_2 56 ... 54
1.7.11. Synthesis of H-GPRP-OH 57 ... 55
1.7.12. Synthesis of Smoc-VVIA-NH_2 58 ... 55
1.7.13. Synthesis of Smoc-DIIW-OH 59 ... 56
1.7.14. Synthesis of Smoc-E(OtBu)K(Boc)R(Pbf)S(tBu)C(Trt)-OH 60 as model for a fully protected peptide. 56
1.7.15. Synthesis of model peptides 61,62 for racemization tests ... 57
1.7.16. Synthesis of Pal-GHK-OH 63 ... 58
1.7.17. Synthesis of Pal-GQPR-OH 64 ... 58
1.7.18. Synthesis of H-GPRPA-NH_2 Vialox (Pentapeptide-3) 65 ... 59
1.7.19. Synthesis of Oxytocin 66 ... 59
1.7.20. Synthesis of Vasopressin (peptide hormone) 67 ... 59
1.7.21. Synthesis of heptaarginine cell-penetrating peptide 68 ... 60
1.7.22. Synthesis of H-YDAGFL-OH Leuphasyl 69 ... 60
1.8. Purification by affinity chromatography ... 62
1.9. Racemization studies ... 65
1.10. Aspartimide formation studies ... 66
1.11. Fluorescent properties of the Smoc group ... 70
1.12. Fluorescence monitoring of resin loading and coupling status during ASPPS ... 72
2. Analytical Methods ... 73
2.1. Mass spectrometry ... 73
2.2. HR-MS ... 73
2.3. Liquid chromatography ... 73
2.4. NMR ... 73
3. Analytical data ... 74
3.1. Smoc-Chloride 2 ... 74
3.2. Amino Acids ... 77
3.2.1. Analytical data of Smoc-L-Ala-OH 3 ... 77
3.2.2. Analytical data of Smoc-D-Ala-OH 4 ... 80
3.2.3. Analytical data of Smoc-L-Arg-OH 5 ... 84
3.2.4. Analytical data of Smoc-L-Arg(Pbf)-OH 6 ... 87
3.2.5. Analytical data of Smoc-L-Asn-OH 7 ... 91
3.2.6. Analytical data of Smoc-L-Asp(OtBu)-OH 8 ... 94
3.2.7. Analytical data of Smoc-L-Cys(Trt)-OH 9 ... 98
3.2.8. Analytical data of Smoc-L-Gln-OH 10 ... 101
3.2.9. Analytical data of Smoc-L-Glu(OtBu)-OH 11 ... 105

3.2.10. Analytical data of Smoc-Gly-OH 12 ... 108
3.2.11. Analytical data of Smoc-L-His-OH 13 ... 112
3.2.12. Analytical data of Smoc-L-His(Trt)-OH 14 ... 115
3.2.13. Analytical data of Smoc-L-Ile-OH 15 ... 119
3.2.14. Analytical data of Smoc-L-Leu-OH 16 ... 122
3.2.15. Analytical data of Smoc-D-Leu-OH 17 ... 126
3.2.16. Analytical data of Smoc-L-Lys(Boc)-OH 18 ... 129
3.2.17. Analytical data of Smoc-L-Met-OH 19 ... 133
3.2.18. Analytical data of Smoc-L-Phe-OH 20 ... 136
3.2.19. Analytical data of Smoc-L-Pro-OH 21 ... 140
3.2.20. Analytical data of Smoc-L-Ser-OH 22 ... 143
3.2.21. Analytical data of Smoc-L-Ser(tBu)-OH 23 ... 147
3.2.22. Analytical data of Smoc-L-Thr-OH 24 ... 150
3.2.23. Analytical data of Smoc-L-Thr(tBu)-OH 25 ... 154
3.2.24. Analytical data of Smoc-L-Trp-OH 26 ... 157
3.2.25. Analytical data of Smoc-L-Trp(Boc)-OH 27 ... 161
3.2.26. Analytical data of Smoc-L-Tyr-OH 28 ... 164
3.2.27. Analytical data of Smoc-L-Tyr(tBu)-OH 29 ... 168
3.2.28. Analytical data of Smoc-L-Val-OH 30 ... 171
3.2.29. Analytical data of Smoc-β-Ala-OH 31 ... 175
3.2.30. Analytical data of Smoc-Aib-OH 32 ... 178
3.3. Analytical data of deprotection studies ... 182
3.3.1. Analytical data of Smoc-Arg-OH 5 deprotection ... 182
3.3.2. Analytical data of Smoc-Leu-OH 16 deprotection ... 184
3.3.3. Analytical data of Smoc-Tyr-OH 28 deprotection ... 186
3.4. Analytical data of stability studies of Smoc-protected amino acids ... 189
3.4.1. Analytical data of Smoc-Arg-OH 5 stability studies ... 189
3.4.2. Analytical data of Smoc-Ile-OH 15 stability studies ... 190
3.4.3. Analytical data of Smoc-Phe-OH 20 stability studies ... 191
3.4.4. Analytical data of Smoc-Pro-OH 21 stability studies ... 192
3.4.5. Analytical data of Smoc-Ser-OH 22 stability studies ... 193
3.5. Analytical data of coupling efficiency and solvent influence ... 194
3.5.1. Analytical Reference data ... 194
3.5.2. ESI-MS data of isolated side products ... 196
3.5.3. Analytical data of the synthesis of Smoc-L-Pro-L-Tyr-OMe 36 in water ... 196
3.5.4. Analytical data of the synthesis of Smoc-Pro-Tyr-OMe 36 in 30% aq. MeCN ... 198
3.5.5. Analytical data of the synthesis Smoc-Pro-Tyr-OMe 36 in 30% EtOAc water mixture (biphasic) 200
3.5.6. Analytical data of the synthesis Smoc-Pro-Tyr-OMe 36 in 30% aq. ethanol ... 202
3.5.7. Analytical data of the synthesis Smoc-Pro-Tyr-OMe 36 in 30% aq. isopropanol ... 204
3.5.8. Analytical data of the synthesis Smoc-Pro-Tyr-OMe 36 in 30% MeTHF water mixture (biphasic) 206
3.5.9. Analytical data of the synthesis Smoc-Pro-Tyr-OMe 36 in 10% aq. Me-THF ... 208
3.6. Analytical data of SPPS coupling efficiency test with Oxyma 39 and HOPO 40 ... 210
3.7. Peptides ... 212
3.7.1. Analytical data of H-AGELS-NH2 (Pentapeptide-31) 48 ... 212
3.7.2. Analytical data of H-GPQGPQ-OH (Hexapeptide-9) 49 ... 212

3.7.3. Analytical data of H-EEMQRR-NH2 (Hexapeptide 3) 50 ... 213
3.7.4. Analytical data of Ac-EEMQRR-NH2 (Acetyl-Hexapeptide 3) 51 ... 213
3.7.5. Analytical data of Synthesis of Leu-Enkephalin amide 52 ... 214
3.7.6. Analytical data of Synthesis of Met-Enkephalin 53 ... 214
3.7.7. Analytical data of Synthesis of Leu-Enkephalin 54 ... 215
3.7.8. Analytical data of Synthesis of H-VQAAIDYING-OH 55 ... 215
3.7.9. Analytical data of Synthesis of H-VQAAIDYING-NH_2 56 ... 216
3.7.10. Analytical data of Synthesis of H-GPRP-OH 57 ... 216
3.7.11. Analytical data of Synthesis of Smoc-VIAA-NH_2 58 ... 217
3.7.12. Analytical data of Synthesis of Smoc-DIIW-OH 59 ... 217
3.7.13. Analytical data of Smoc-E(OtBu)K(Boc)R(Pbf)S(tBu)C(Trt)-OH 60 ... 218
3.7.14. Analytical data of H-CYEIS-NH_2 61 ... 218
3.7.15. Analytical data of amino acid racemization of H-CYEIS-NH_2 61 by *C.A.T. GmbH & Co Chromatographie und Analysentechnik KG* (Tübingen, Germany) ... 219
3.7.16. Analytical data of H-ANKPG-NH_2 62 ... 223
3.7.17. Analytical data of amino acid racemization of H-ANKPG-NH_2 62 by *C.A.T. GmbH & Co Chromatographie und Analysentechnik KG* (Tübingen, Germany) ... 224
3.7.18. Analytical data of amino acid racemization of Smoc-Asn-OH 7 by *C.A.T. GmbH & Co Chromatographie und Analysentechnik KG* (Tübingen, Germany) ... 228
3.7.19. Analytical data of Pal-GHK-OH 63 ... 230
3.7.20. Analytical data of Pal-GQPR-OH 64 ... 230
3.7.21. Analytical data of H-GPRPA-NH_2 Vialox (Pentapeptide-3) 65 ... 231
3.7.22. Analytical data of Oxytocin 66 ... 231
3.7.23. Analytical data of Vasopressin 67 (peptide hormone) ... 232
3.7.24. Analytical data of heptaarginine 68 ... 232
3.7.25. Analytical data of H-YDAGFL-OH Leuphasyl 69 ... 233
3.8. Analytical data of Aspartimide formation ... 233
3.8.1. Reference HPLC data of peptides 70-73 ... 233
3.8.2. HPLC data of H-VKDGYI-OH 70 after 3h incubation with different bases ... 235
3.8.3. HPLC data of H-VKDGYI-OH 70 after 16h incubation with different bases ... 236
3.8.4. HPLC data of H-VK(D-D)GYI-NH_2 71 after 3h incubation with different bases ... 238
3.8.5. HPLC data of H-VK(D-D)GYI-NH_2 71 after 16h incubation with different bases ... 239
3.8.6. HPLC data of H-VKNGYI-NH_2 72 after 3h incubation with different bases ... 241
3.8.7. HPLC data of H-VKNGYI-NH_2 72 after 16h incubation with different bases ... 242
3.8.8. Temperature dependent formation of H-VK(β-D)GYI-NH_2 73 in water ... 244
3.9. HPLC data of the capping experiments ... 245
References ... 247

1. Synthesis

1.1. Synthesis of 2,7-disulfo-9-fluorenylmethoxycarbonyl chloride (Smoc-Cl) 2

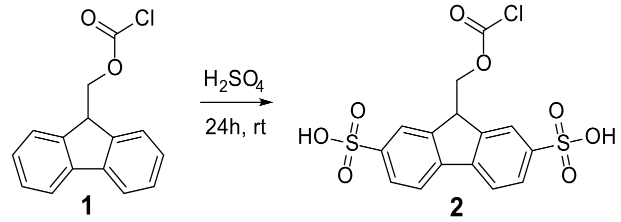

Figure S1: Synthesis of Smoc-Cl **2**.

60g Fmoc chloride **1** were dissolved in 250 ml dichloromethane (DCM). 62 ml of 20% oleum were slowly dropped into the solution under stirring. After precipitation of a white-grey solid, the solution was drained and 20 ml of 1,4-dioxane were added under ice-cooling and stirring until the dioxan-SO3 complex was formed (approx. 10-15min). To dissolve this rubber-like compound, 150 ml of 1,2-dichloroethane were added and stirred. SO_3-dioxane complex remained in solution, Smoc-Cl **2** precipitated as solid. Crude product was filtered, washed twice with DCM, and dried in vacuo and the used 1,2-dichlorethane was regenerated. Smoc-Cl **2** was obtained as a slightly yellow powder (yield ~75%).

HR-MS calc. for $C_{15}H_{11}ClO_8S_2$ m/z: 416.95111, meas. 416.95146 $[M\text{-}H]^-$.

^{1}H NMR (500 MHz, CD_3CN) δ: 4.55 (t, J = 5.7 Hz, 1H), 4.90 (d, J = 5.7 Hz, 2H), 8.00 (d, J = 8.1 Hz, 2H), 8.08 (d, J = 8.1 Hz, 2H), 8.18 (s, 2H).

^{13}C NMR (126 MHz, CD_3CN): δ 47.80, 72.67, 122.98, 124.48, 128.13, 140.82, 144.97, 145.68, 151.05.

1.2. Synthesis of N_α-Smoc amino acids

1.2.1. General procedure

Typically, 1 eq. of the corresponding amino acid was dissolved in water and 1 eq. Smoc-Cl **2** was added to the solution. The pH of the solution was adjusted to 8.5 to allow the formation of the N_α-Smoc amino acid. After 30 minutes, reaction mixture was frozen and lyophilized, with subsequent isolation by preparative HPLC, giving after freeze-drying the respective Smoc-protected amino acid as a white or slightly white powder (yields > 85%).

1.2.2. Synthesis of Smoc-L-Ala-OH 3

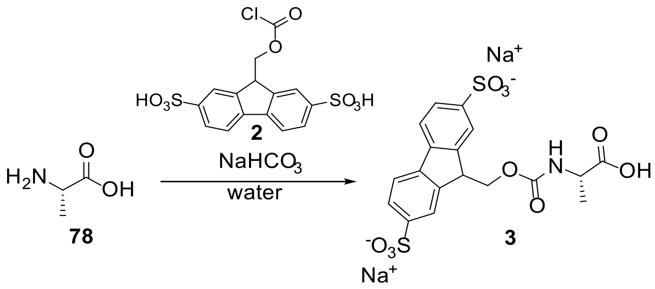

Figure S2: Synthesis of Smoc-L-Ala-OH **3**.

According to the general procedure, 2.00g (22.45 mmol, 1 eq.) L-alanine **78** and 9.36g (22.45 mmol, 1 eq.) Smoc-Cl **2** were dissolved in 30ml water and sodium hydrogen carbonate was added to adjust the

pH to 8.5. The reaction mixture was stirred at ambient temperature and the solvent was removed by lyophilization. The product was isolated by preparative RP-HPLC (gradient 0 to 40% B). After work up, 10.72g (87.2%) of Smoc-L-alanine **3** were obtained as a white powder.

RP-HPLC (gradient 0 to 40% B): t_R = 12.54 min.

ESI-MS calc. for $C_{18}H_{17}NO_{10}S_2$ m/z: 471.45, meas. 470.17 $[M+H]^-$; calc. 235.73, meas. 234.84 $[M+H]^{2-}$

HR-MS calc. for $C_{18}H_{17}NO_{10}S_2$ m/z: 472.03666, meas. 472.03698 $[M+H]^+$.

^{1}H NMR (500 MHz, DMSO-d_6) δ: 1.22 (d, J = 7.4 Hz, 3H), 3.95 (q, J = 7.3 Hz, 1H), 4.23 (t, J = 5.5 Hz, 1H), 4.38 (dd, J = 10.9, 5.7 Hz, 1H), 4.49 (dd, J = 10.9, 5.4 Hz, 1H), 7.52 (br, NH), 7.68 (d, J = 7.9 Hz, 2H), 7.83 (d, J = 7.9 Hz, 2H), 7.89 (s, 1H), 7.91 (s, 1H).

^{13}C NMR (126 MHz, DMSO) δ: 16.83, 47.02, 49.27, 65.14, 119.47, 121.97, 122.01, 125.31, 140.52, 143.99, 144.17, 147.16, 155.93, 174.24.

1.2.3. Synthesis of Smoc-D-Ala-OH 4

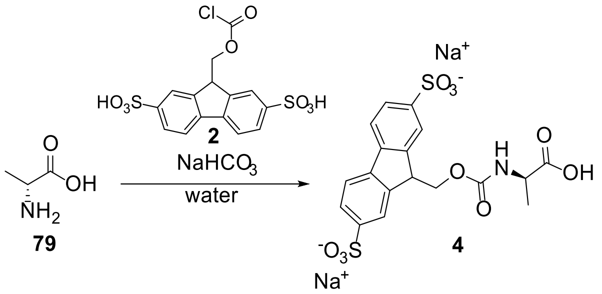

Figure S3: Synthesis of Smoc-D-Ala-OH **4**.

According to the general procedure, 2.00g (22.45 mmol, 1 eq.) D-alanine **79** and 9.36g (22.45 mmol, 1 eq.) Smoc-Cl **2** were dissolved in 30ml water and sodium hydrogen carbonate was added to adjust the pH to 8.5. The reaction mixture was stirred at ambient temperature and the solvent was removed by lyophilization. The product was isolated by preparative RP-HPLC (gradient 0 to 40% B). After work up, 10.58g (86.9%) of Smoc-D-alanine **4** were obtained as a white powder.

RP-HPLC (0 to 40% B): t_R = 12.29 min.

ESI-MS calc. for $C_{18}H_{17}NO_{10}S_2$ m/z: 471.45, meas. 470.05 $[M+H]^-$; calc. 235.73, meas. 234.92 $[M+H]^{2-}$

HR-MS calc. for $C_{15}H_{11}ClO_8S_2$ m/z: 470.0221, meas. 470.02370 $[M-H]^-$.

^{1}H NMR (500 MHz, DMSO-d_6) δ: 1.22 (d, J = 7.3 Hz, 3H), 3.95 (q, J = 7.3 Hz, 1H), 4.23 (t, J = 5.5 Hz, 1H), 4.37 (dd, J = 11.0, 5.7 Hz, 1H), 4.38 (dd, J = 10.9, 5.5 Hz, 1H), 7.5 (br, NH), 7.68 (d, J = 8.2 Hz, 2H), 7.83 (d, J = 7.9 Hz, 2H), 7.89 (s, 1H), 7.91 (s, 1H).

^{13}C NMR (126 MHz, DMSO) δ: 16.82, 47.01, 49.26, 65.11, 119.43, 121.94, 125.28, 140.49, 143.97, 144.15, 147.19, 155.91, 174.22.

1.2.4. Synthesis of Smoc-L-Arg-OH 5

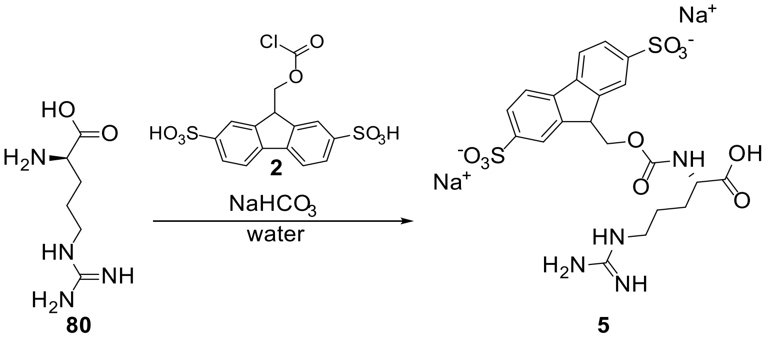

Figure S4: Synthesis of Smoc-L-Arg-OH **5**.

According to the general procedure, 3.91g (22.45 mmol, 1 eq.) L-arginine **80** and 9.36g (22.45 mmol, 1 eq.) Smoc-Cl **2** were dissolved in 30ml water and sodium hydrogen carbonate was added to adjust the pH to 8.5. The reaction mixture was stirred at ambient temperature and the solvent was removed by lyophilization. The product was isolated by preparative RP-HPLC (gradient 0 to 40% B). After work up, 11.55g (85.7%) of Smoc-L-arginine-OH **5** were obtained as a white powder.

RP-HPLC (0 to 40% B): t_R = 12.88 min.

ESI-MS calc. for $C_{21}H_{24}N_4O_{10}S_2$ m/z: 556.56, meas. 555.4 $[M-H]^-$; calc. 278.28, meas. 277.3 $[M-H]^{2-}$.

HR-MS calc. for $C_{21}H_{24}N_4O_{10}S_2$ m/z: 557.10066, meas. 557.10069 $[M+H]^+$.

^{1}H NMR (500 MHz, DMSO-d_6) δ: 1.47 (m, 2H), 1.56, 1.72 (m, 2H), 3.02 (dq, *J* = 13.0, 6.5 Hz, 1H), 3.10 (dq, *J* = 13.0, 6.5 Hz, 1H), 3.96 (dt, *J* = 9.2, 4.3 Hz, 1H), 4.32 (t, *J* = 5.5, 21), 4.33 (dd, *J* = 10.0, 6.3 Hz, 1H), 4.54 (dd, *J* = 10.0, 6.3 Hz, 1H), 7.52 (d, *J* = 8.5 Hz, NH), 7.70 (d, *J* = 7.7, 2H), 7.74 (t, *J* = 5.6 Hz, NH), 7.85, 7.86 (d, *J* = 7.9, 2H), 7.88 (s, 1H), 7.93 (s, 1H).

^{13}C NMR (126 MHz, DMSO) δ: 24.93, 27.87, 40.00, 46.91, 52.91, 64.85, 119.60, 125.30, 140.61, 143.51, 144.50, 146.76, 155.93, 156.63, 173.30.

1.2.5. Synthesis of Smoc-L-Arg(Pbf)-OH 6

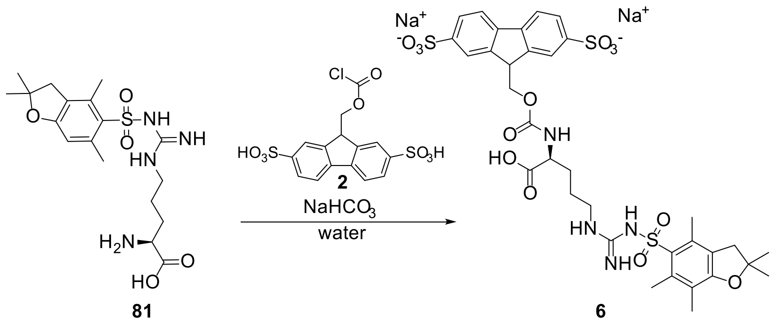

Figure S5: Synthesis of Smoc-L-Arg(Pbf)-OH **6**.

According to the general procedure, 3.00g (7.27 mmol, 1 eq.) L-Arg(Pbf)-OH **81** and 3.03g (7.27 mmol, 1 eq.) Smoc-Cl **2** were dissolved in 30ml water acetonitrile mixture (2:1) and sodium hydrogen carbonate was added to adjust the pH to 8.5. The reaction mixture was stirred at ambient temperature and the solvent was removed by lyophilization. The product was isolated by preparative RP-HPLC (gradient 10 to 100% B). After work up, 5.19g (85.1%) of Smoc-L-Arg(Pbf)-OH **6** were obtained as a slightly yellow powder.

RP-HPLC (10 to 100% B): t_R = 14.70 min.

ESI-MS calc. for $C_{33}H_{38}N_4O_{13}S_3$ m/z: 808.89, meas. 807.19 $[M-H]^-$; calc. 404.45, meas. 403.46 $[M-2H]^{2-}$.

HR-MS calc. for $C_{33}H_{38}N_4O_{13}S_3$ m/z: 809.18268, meas. 809.18227 $[M+H]^+$.

^{1}H NMR (500 MHz, DMSO-d_6) δ: 1,41 (s, 6H), 1.47 (m, 2H), 1.56 – 1.64 (m, 2H), 2.01 (s, 6H), 2.43 (s, 6H), 2.48 (s, 6H), 3.07 (q, *J* = 7.3 Hz, 2H), 2.97 (s, 2H), 3.96 (td, *J* = 9.1, 4.7 Hz, 1H), 4.3 (t, *J* = 5.5 Hz, 1H), 4.32 (m, 1H), 4.51 (m, 1H), 7.68 (d,d *J* = 7.8, 1.4 Hz, 2H), 7.52 (br, NH), 7.74 (t, *J* = 5.6 Hz, NH), 7.84 (d, *J* = 7.9, 2H), 7.88 (s, 1H), 7.91 (s, 1H).

^{13}C NMR (126 MHz, DMSO) δ: 12.22, 17.51, 18.91, 25.10, 27.80, 28.25, 39.71, 42.35; 46.92, 53.17, 64.98, 86.32, 116.34, 119.54, 122.03, 124.33, 125.28, 131.42, 133.90, 137.32, 140.55, 143.58, 144.39, 146.91, 155.96, 157.57, 166.74, 173.35.

1.2.6. Synthesis of Smoc-L-Asn-OH 7

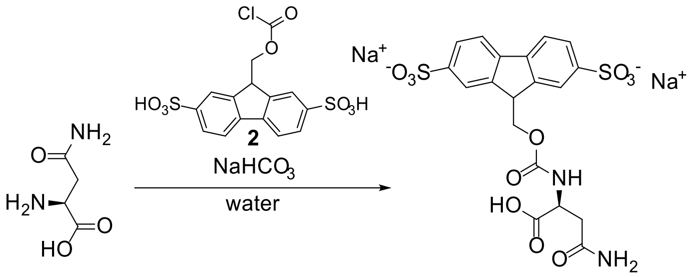

82

7

Figure S6: Synthesis of Smoc-L-Asn-OH **7**.

According to the general procedure, 3.37g (22.45 mmol, 1 eq.) L-asparagine **82** (mono hydrate) and 9.36g (22.45 mmol, 1 eq.) Smoc-Cl **2** were dissolved in 30ml water and sodium hydrogen carbonate was added to adjust the pH to 8.5. The reaction mixture was stirred at ambient temperature and the solvent was removed by lyophilization. The product was isolated by preparative RP-HPLC (gradient 0 to 40% B). After work up, 12.03g (90.4%) of Smoc-L-asparagine-OH **7** were obtained as a white powder.

RP-HPLC (0 to 40% B): t_R = 10.76 min.

ESI-MS calc. for $C_{19}H_{18}N_2O_{11}S_2$ m/z: 514.48, meas. 513.20 $[M-H]^-$; calc. 257.24, meas. 256.30 $[M-2H]^{2-}$.

HR-MS calc. for $C_{19}H_{18}N_2O_{11}S_2$ m/z: 515.04248, meas. 515.04298 $[M+H]^+$.

^{1}H NMR (500 MHz, DMSO-d_6) δ: 2.43 (dd, *J* = 15.2, 7.5 Hz, 1H), 2.54 (dd, *J* = 15.2, 5.8 Hz, 1H), 4.26 (t, 1H), 4.27 (m, 1H), 4.42 (m, 2H), 5.6 (br, NH), 7.40 (d, *J* = 8.2 Hz, 1H), 7.69 (d, *J* = 7.5 Hz, 2H), 7.84 (d, *J* = 7.9 Hz, 1H), 7.90 (s, 1H), 7.91 (s, 1H).

^{13}C NMR (126 MHz, DMSO) δ: 36.78, 46.06, 50.71, 65.25, 119.57, 122.11, 125.37, 140.59, 144.03, 144.14, 146.99, 155.83, 171.56, 172.83.

1.2.7. Synthesis of Smoc-L-Asp(OtBu)-OH 8

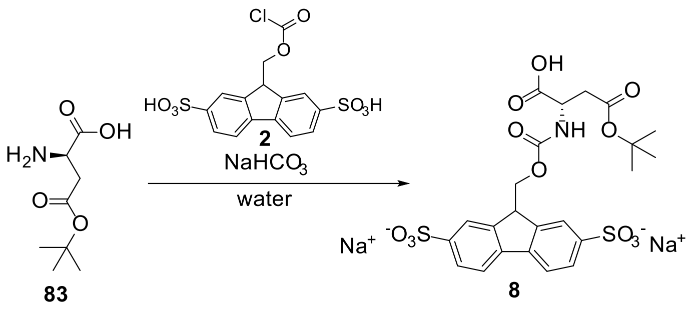

Figure S7: Synthesis of Smoc-L-Asp(OtBu)-OH **8**.

According to the general procedure, 4.00g (21.14 mmol, 1 eq.) L-Asp(OtBu)-OH **83** and 8.81g (21.14 mmol, 1 eq.) Smoc-Cl **2** were dissolved in 40ml water and sodium hydrogen carbonate was added to adjust the pH to 8.5. The reaction mixture was stirred at ambient temperature and the solvent was removed by lyophilization. The product was isolated by preparative RP-HPLC (gradient 0 to 60% B). After work up, 11.32g (86.7%) of Smoc- L-Asp(OtBu)-OH **8** were obtained as a white powder.

RP-HPLC (0 to 60% B): t_R = 15.14 min.

ESI-MS calc. for $C_{23}H_{25}NO_{12}S_2$ m/z: 571.57, meas. 570.08 $[M-H]^-$; calc. 285.79, meas. 284.84 $[M-2H]^{2-}$

HR-MS calc. for $C_{23}H_{25}NO_{12}S_2$ m/z: 610.04498, meas. 610.04568 $[M+H]^+$.

^{1}H NMR (500 MHz, DMSO-d_6) δ: 1.38 (s, 9H), 2.40 (dd, *J* = 14.7, 7.8 Hz, 1H), 2.59 (dd, *J* = 14.7, 5.4 Hz, 1H), 3.97 (dt, *J* = 7.5, 5.3 Hz, 1H), 4.22 (m, 2H), 4.23 (m, 1H), 6.80 (d, *J* = 7.5 Hz, NH), 7.69 (d, *J* = 7.7 Hz, 2H), 7.83 (d, *J* = 7.9 Hz, 2H),7.89 (s, 1H), 7.94 (s, 1H).

^{13}C NMR (126 MHz, DMSO) δ: 27.78, 39.54, 46.62, 52.92, 65.47, 79.10, 119.35, 122.31, 122.45, 125.25, 140.31, 143.78, 144.15, 147.26, 155.36, 170.72, 172.58.

1.2.8. Synthesis of Smoc-L-Cys(Trt)-OH 9

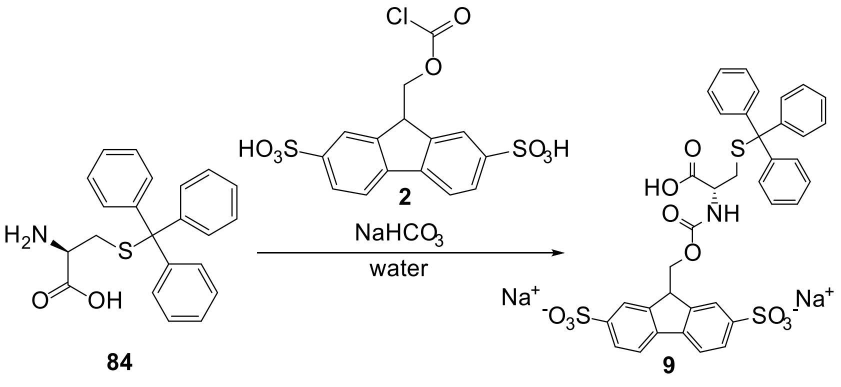

Figure S8: Synthesis of Smoc- L-Cys(Trt)-OH **9**.

According to the general procedure, 4.00g (11.00 mmol, 1 eq.) L-Cys(Trt)-OH **84** and 4.59g (11.00 mmol, 1 eq.) Smoc-Cl **2** were dissolved in 40ml water acetonitrile mixture (1:1) and sodium hydrogen carbonate was added to adjust the pH to 8.5. The reaction mixture was stirred at ambient temperature and the solvent was removed by lyophilization. The product was isolated by preparative RP-HPLC (30 to 100% B). After work up, 7.40g (85.1%) of Smoc-L-Cys(Trt)-OH **9** were obtained as a white powder.

RP-HPLC (10 to 100% B): t_R = 16.94 min.

ESI-MS calc. for $C_{37}H_{31}NO_{10}S_3$ m/z: 745.83, meas. 744.18 $[M-H]^-$; calc. 372.92, meas. 371.96 $[M-2H]^{2-}$

HR-MS calc. for $C_{37}H_{31}NO_{10}S$ m/z: 744.10373, meas. 744.10393 [M-H]$^-$.

^{1}H NMR (500 MHz, DMSO-d_6) δ: 2.38 (dd, J = 12.4, 5.0 Hz, 1H), 2.56 (dd, J = 12.6, 9.6 Hz, 1H), 3.77 (m, 1H), 4.37 (dd, J = 11.1; 5.8 Hz, 1H), 4.20 (t, J = 5.6 Hz, 1H), 4.41 (dd, J = 11.5, 6.5 Hz, 1H), 6.6 (d, J = 8.4 Hz, NH), 7.24 (t, J = 6.9 Hz, 3H), 7.3 (m, 12H), 7.68 (d, J = 7.9 Hz, 2H), 7.82 (d, J = 7.8 Hz, 2H), 7.90 (s, 1H), 7.91 (s, 1H).

^{13}C NMR (126 MHz, DMSO) δ: 32.52, 47.0, 53.58, 65.57, 66.28, 119.35, 122.0, 125.30, 126.67, 128.0, 129.0, 140.36, 143.97, 144.21, 147.4, 155.94, 171.63.

1.2.9. Synthesis of Smoc-L-Gln-OH 10

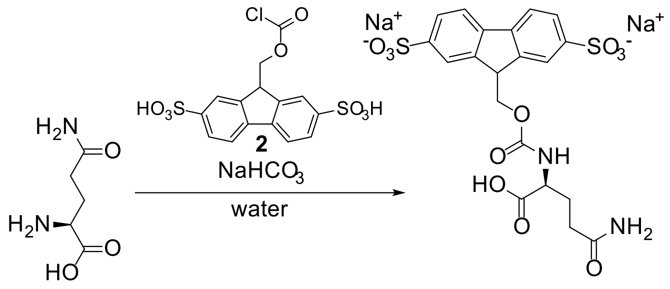

Figure S9: Synthesis of Smoc-L-Gln-OH **10**.

According to the general procedure, 3.28g (22.45 mmol, 1 eq.) L-glutamine **85** and 9.36g (22.45 mmol, 1 eq.) Smoc-Cl **2** were dissolved in 30ml water and sodium hydrogen carbonate was added to adjust the pH to 8.5. The reaction mixture was stirred at ambient temperature and the solvent was removed by lyophilization. The product was isolated by preparative RP-HPLC (gradient 0 to 40% B). After work up, 12.24g (90.8%) of Smoc-L-Gln-OH **10** were obtained as a white powder.

RP-HPLC (0 to 40% B): t_R = 11.38 min.

ESI-MS calc. for $C_{20}H_{20}N_2O_{11}S_2$ m/z: 528.50, meas. 527.20 [M-H]$^-$; calc. 264.25, meas. 263.30 [M-2H]$^{2-}$.

HR-MS calc. for $C_{20}H_{20}N_2O_{11}S_2$ m/z: 529.05813, meas. 529.05847 [M+H]$^+$.

^{1}H NMR (500 MHz, DMSO-d_6) δ: 1.68 (m, 1H), 1.99 (m, 1H), 2.16 (ddd, J = 9.5, 6.8, 3.2 Hz, 2H), 3.88 (dt, J = 10.4, 5.0 Hz, 1H), 4.27 (t, J = 6.0 Hz, 1H), 4.32 (dd, J = 10.7, 5.7 Hz, 1H), 4.45 (dd, J = 10.7, 6.8 Hz, 1H), 7.54 (d, J = 7.7 Hz, NH), 7.69 (d, J = 7.8 Hz, 2H), 7.84 (d, J = 7.9 Hz, 2H), 7.90 (s, 1H), 7.93 (s, 1H).

^{13}C NMR (126 MHz, DMSO) δ: 26.16, 31.23, 46.86, 53.47, 65.18, 119.48, 122.15, 125.32, 140.45, 140.50, 143.67, 144.32, 147.12, 156.12, 173.58, 173.68.

1.2.10. Synthesis of Smoc-L-Glu(OtBu)-OH 11

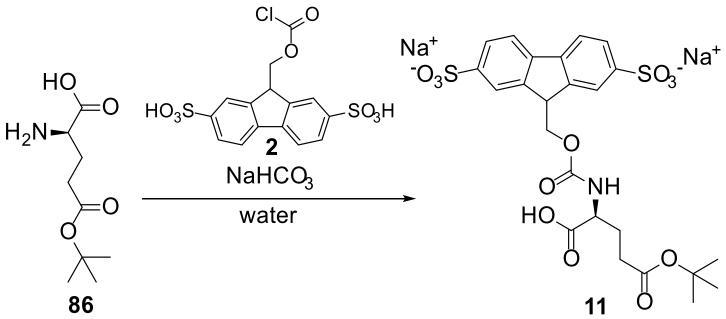

Figure S10: Synthesis of Smoc-L-Glu(OtBu)-OH **11**.

According to the general procedure, 4.50g (22.14 mmol, 1 eq.) L-Glu(OtBu)-OH **86** and 9.23g (22.14 mmol, 1 eq.) Smoc-Cl **2** were dissolved in 30ml water and sodium hydrogen carbonate was added to adjust the pH to 8.5. The reaction mixture was stirred at ambient temperature and the solvent was removed by lyophilization. The product was isolated by preparative RP-HPLC (gradient 0 to 60% B). After work up, 12.29g (88.2%) of Smoc-L-Glu(OtBu)-OH **11** were obtained as a white powder.

RP-HPLC (0 to 60% B): t_R = 16.31 min.

ESI-MS calc. for $C_{24}H_{27}NO_{12}S_2$ m/z: 585.60, meas. 584.08 $[M-H]^-$; calc. 292.80, meas. 291.85 $[M-2H]^{2-}$

HR-MS calc. for $C_{24}H_{27}NO_{12}S_2$ m/z: 608.08669, meas. 608.08709 $[M+H]^+$; calc. 624.06063 meas. 624.06093 $[M+K]^+$.

^{1}H NMR (500 MHz, DMSO-d_6) δ: 1.38 (s, 9H), 1.78 (tt, *J* = 12.1, 5.6, 1H), 1.90 (tt, *J* = 10.7, 5.2, 1H), 2.12 (ddd, *J* = 16.0, 10.8, 5.3 Hz, 1H), 2.23 (ddd, *J* = 16.1, 10.9, 5.5 Hz, 1H), 3.65 (q, *J* = 6.0 Hz, 1H), 4.23 (m, 3H), 6.63 (d, *J* = 6.7 Hz, NH), 7.69 (d, *J* = 8.0 Hz, 2H), 7.83 (d, *J* = 8.0 Hz, 2H), 7.89 (s, 1H), 7.94 (s, 1H).

^{13}C NMR (126 MHz, DMSO) δ: 27.73, 27.96, 30.73, 46.20, 54.80, 65.20, 79.13; 119.30, 122.30, 125.00, 140.31, 143.95, 147.20, 155.50, 172.40, 173.50.

1.2.11. Synthesis of Smoc-Gly-OH 12

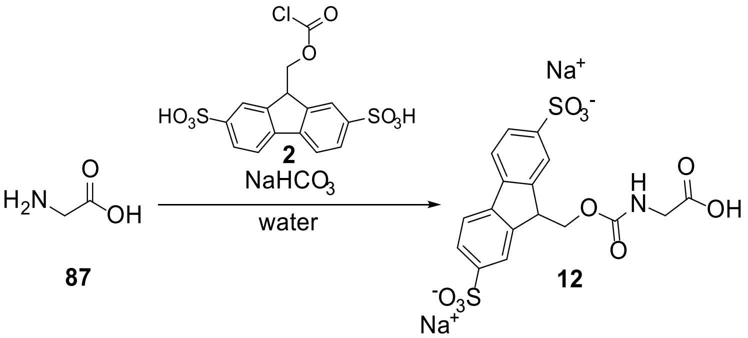

Figure S11: Synthesis of Smoc-Gly-OH **12**.

According to the general procedure, 1.69g (22.45 mmol, 1 eq.) glycine **87** and 9.36g (22.45 mmol, 1 eq.) Smoc-Cl **2** were dissolved in 30ml water and sodium hydrogen carbonate was added to adjust the pH to 8.5. The reaction mixture was stirred at ambient temperature and the solvent was removed by lyophilization. The product was isolated by preparative RP-HPLC (gradient 0 to 40% B). After work up, 10.55g (93.7%) of Smoc-Gly-OH **12** were obtained as a white powder.

RP-HPLC (0 to 40% B): t_R = 11.48 min.

ESI-MS calc. for $C_{17}H_{15}NO_{10}S_2$ m/z: 457.42, meas. 456.17 $[M-H]^-$; calc. 228.71, meas. 227.84 $[M-2H]^{2-}$

HR-MS calc. for $C_{17}H_{15}NO_{10}S_2$ m/z: 458.02101, meas. 458.02086 $[M+H]^+$.

^{1}H NMR (500 MHz, DMSO-d_6) δ: 3.61 (s, 2H), 4.25 (t, *J* = 5.7 Hz, 1H), 4.43 (d, *J* = 5.8 Hz, 2H), 7.49 (br, NH), 7.69 (d, *J* = 7.9, 2H), 7.85 (d, *J* = 7.9 Hz, 2H), 7.91 (s, 2H).

^{13}C NMR (126 MHz, DMSO) δ: 42.15, 46.97, 65.37, 119.56, 122.05, 125.36, 140.59, 144.11, 147.04, 156.52, 171.39.

1.2.12. Synthesis of Smoc-L-His-OH 13

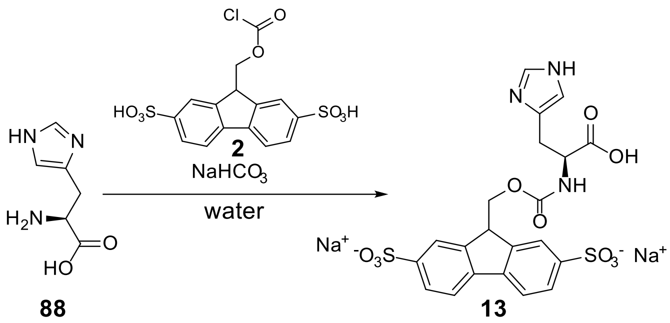

Figure S12: Synthesis of Smoc-L-His-OH **13**.

According to the general procedure, 3.48g (22.45 mmol, 1 eq.) L-histidine **88** and 9.36g (22.45 mmol, 1 eq.) Smoc-Cl **2** were dissolved in 30ml water and sodium hydrogen carbonate was added to adjust the pH to 8.5. The reaction mixture was stirred at ambient temperature and the solvent was removed by lyophilization. The product was isolated by preparative RP-HPLC (gradient 0 to 40% B). After work up, 12.06g (92.4%) of Smoc-L-His-OH **13** were obtained as a white powder.

RP-HPLC (0 to 40% B): t_R = 11.90 min.

ESI-MS calc. for $C_{21}H_{19}N_3O_{10}S_2$ m/z: 537.51, meas. 536.40 $[M-H]^-$; calc. 268.76, meas. 267.80 $[M-2H]^{2-}$.

HR-MS calc. for $C_{21}H_{19}N_3O_{10}S_2$ m/z: 538.05846, meas. 538.05888 $[M+H]^+$.

^1H NMR (500 MHz, DMSO-d_6) δ: 2.94 (dd, *J* = 15.0, 7.9 Hz, 1H), 3.13 (dd, *J* = 15.0, 4.4 Hz, 1H), 4.23 (m, 1H), 4.33 (dd, *J* = 10.8, 5.3 Hz, 1H), 4.41 (dd, *J* = 10.8, 6.8 Hz, 1H), 7.27 (s, 1H), 7.66 (d, *J* = 8.8 Hz, NH), 7.71 (d, *J* = 7.9 Hz, 2H), 7.84 (s, 1H), 7.85 (d, *J* = 7.9 Hz, 2H), 7.92 (s, 1H), 8.92 (s, 1H).

^{13}C NMR (126 MHz, DMSO) δ 26.03, 46.69, 52.73, 65.12, 117.38, 119.65, 121.90, 122.00, 125.36, 129.28, 133.71, 140.57, 140.66, 143.60, 144.34, 146.86, 155.78, 171.97.

1.2.13. Synthesis of Smoc-L-His(Trt)-OH 14

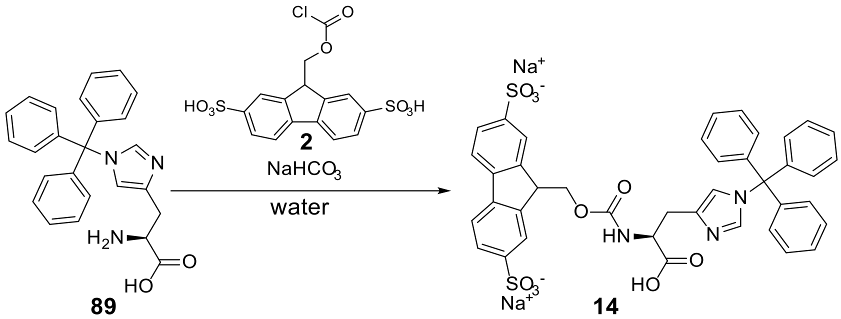

Figure S13: Synthesis of Smoc-L-His(Trt)-OH **14**.

According to the general procedure, 2.89g (7.27 mmol, 1 eq.) L-His(Trt)-OH **89** and 3.03g (7.27 mmol, 1 eq.) Smoc-Cl **2** were dissolved in 50ml water acetonitrile mixture (1:1) and sodium hydrogen carbonate was added to adjust the pH to 8.5. The reaction mixture was stirred at ambient temperature and the solvent was removed by lyophilization. The product was isolated by preparative RP-HPLC (30 to 100% B). After work up, 5.19g (86.6%) of Smoc-L-His(Trt)-OH **14** were obtained as a white powder.

RP-HPLC (10 to 100% B): t_R = 15.28 min.

ESI-MS calc. for $C_{40}H_{33}N_3O_{10}S_2$ m/z: 779.84, meas. 778.19 $[M-H]^-$; calc. 389.92, meas. 388.86 $[M-2H]^{2-}$.

HR-MS calc. for $C_{40}H_{33}N_3O_{10}S_2$ m/z: 780.16801, meas. 780.16788 $[M+H]^+$.

^{1}H NMR (500 MHz, DMSO-d_6) δ: 2.65 (dd, J = 14.9, 8.6 Hz, 1H), 2.99 (dd, J = 14.8, 4.6 Hz, 1H), 3.94 (m, 1H), 3.96 (m, 1H), 4.13 (m, 1H), 4.19 (t, J = 6.3 Hz, 1H), 6.97 (br, NH), 7.06 (m, 9H), 7.22 (br, 1H), 7.37 (m, 6H), 7.69 (d, J = 7.7 Hz, 2H), 7.84 (d, J = 7.7 Hz, 2H), 7.89 (s, 1H), 7.99 (s, 1H).

^{13}C NMR (126 MHz, DMSO) δ: 31.95, 46.9, 55.81, 65.23, 74.38, 118.80, 119.10, 122.30, 125.60, 127.50, 128.5, 128.9, 137.50, 138.30, 140.80, 142.40, 143.76, 144.00, 147.65, 156.0, 171.29.

1.2.14. Synthesis of Smoc-L-Ile-OH 15

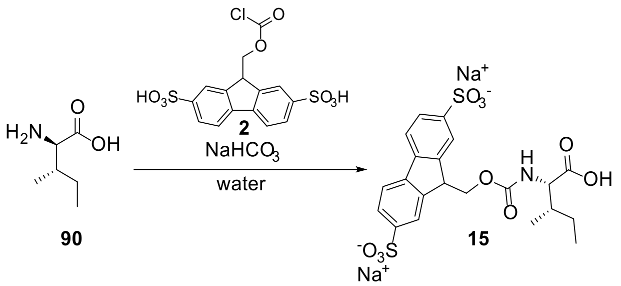

90 15

Figure S14: Synthesis of Smoc-L-Ile-OH **15**.

According to the general procedure, 2.94g (22.45 mmol, 1 eq.) L-isoleucine **90** and 9.36g (22.45 mmol, 1 eq.) Smoc-Cl **2** were dissolved in 30ml water acetonitrile mixture (3:1) and sodium hydrogen carbonate was added to adjust the pH to 8.5. The reaction mixture was stirred at ambient temperature and the solvent was removed by lyophilization. The product was isolated by preparative RP-HPLC (gradient 0 to 40% B). After work up, 11.13g (88.9%) of Smoc-L-isoleucine **15** were obtained as a white powder.

RP-HPLC (0 to 40% B): t_R = 17.12 min.

ESI-MS calc. for $C_{21}H_{23}NO_{10}S_2$ m/z: 513.53, meas. 512.17 $[M-H]^-$; calc. 256.77, meas. 255.84 $[M-2H]^{2-}$

HR-MS calc. for $C_{21}H_{23}NO_{10}S_2$ m/z: 514.08361, meas. 514.08407 $[M+H]^+$.

^{1}H NMR (500 MHz, DMSO-d_6) δ: 0.81 (d, J = 7.4 Hz, 3H), 0.84 (d, J = 6.9 Hz, 3H), 1.17 (ddq, J = 13.6, 8.9, 7.3 Hz, 1H), 1.39 (ddq, J = 13.6, 7.4, 4.5 Hz, 1H), 1.75 (m, 1H), 3.87 (t, J = 6.5 Hz, 1H), 4.23 (t, J = 5.8 Hz, 1H), 4.37 (dd, J = 10.9, 5.7 Hz, 1H), 4.86 (dd, J = 10.9, 5.9 Hz, 1H), 7.46 (d, J = 8.1 Hz, NH), 7.68 (dd, J = 7.9, 1.2 Hz, 2H), 7.83 (d, J = 7.9 Hz, 2H), 7.92 (s, 2H).

^{13}C NMR (126 MHz, DMSO) δ: 11.22, 15.48, 24.88, 35.72, 47.11, 58.68, 65.46, 119.44, 122.09, 125.31, 140.48, 144.03, 144.16, 147.15, 147.17, 156.44, 173.06.

1.2.15. Synthesis of Smoc-L-Leu-OH 16

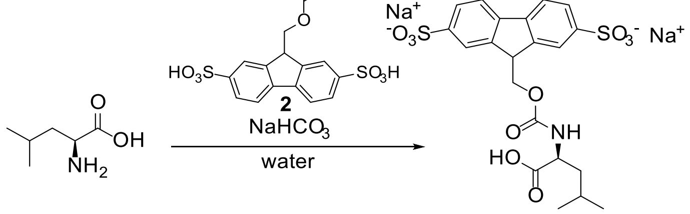

91 16

Figure S15: Synthesis of Smoc-L-Leu-OH **16**.

According to the general procedure, 2.94g (22.45 mmol, 1 eq.) L-leucine **91** and 9.36g (22.45 mmol, 1 eq.) Smoc-Cl **2** were dissolved in 30ml water acetonitrile mixture (3:1) and sodium hydrogen carbonate was added to adjust the pH to 8.5. The reaction mixture was stirred at ambient temperature and the

solvent was removed by lyophilization. The product was isolated by preparative RP-HPLC (gradient 0 to 40% B). After work up, 11.33g (90.6%) of Smoc-L-leucine **16** were obtained as a white powder.

RP-HPLC (0 to 40% B): t_R = 17.52 min.

ESI-MS calc. for $C_{21}H_{23}NO_{10}S_2$ m/z: 513.53, meas. 512.17 $[M-H]^-$; calc. 256.77, meas. 255.84 $[M-2H]^{2-}$

HR-MS calc. for $C_{21}H_{23}NO_{10}S_{22}$ m/z: 514.08361, meas. 514.08410 $[M+H]^+$.

^{1}H NMR (500 MHz, DMSO-d_6) δ: 0.83 (d, *J* = 6.5 Hz, 3H), 0.85 (d, *J* = 6.6 Hz, 3H), 1.41 (ddd, *J* = 13.5, 9.0, 4.9 Hz, 1H), 1.53 (ddd, *J* = 13.5, 10.2, 5.1 Hz, 1H), 1.61 (m, 1H), 3.93 (dt, *J* = 10.7, 5.4 Hz, 1H), 4.22 (t, *J* = 5.6 Hz, 1H), 4.38 (dd, *J* = 11.0, 5.6 Hz, 1H), 4.46 (dd, *J* = 11.0, 5.6 Hz, 1H), 7.51 (d, *J* = 7.9 Hz, NH), 7.68 (d, *J* = 8.0, 2H), 7.83 (d, *J* = 8.0 Hz, 2H), 7.90 (s, 1H), 7.91 (s, 1H).

^{13}C NMR (126 MHz, DMSO) δ: 21.28, 22.78, 24.17, 39.5, 47.06, 52.28, 65.35, 119.37, 122.00, 125.27, 140.42, 144.01, 144.09, 147.29, 156.29, 174.17.

1.2.16. Synthesis of Smoc-D-Leu-OH 17

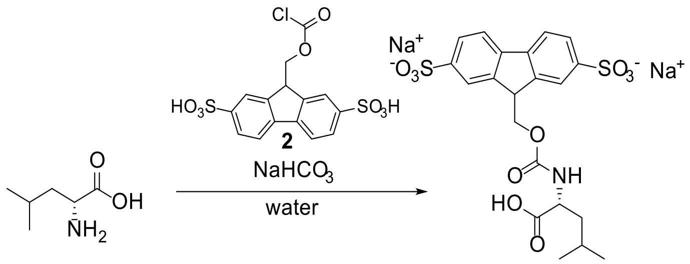

92 17

Figure S16: Synthesis of Smoc-D-Leu-OH **17**.

According to the general procedure, 2.94g (22.45 mmol, 1 eq.) D-leucine **92** and 9.36g (22.45 mmol, 1 eq.) Smoc-Cl **2** were dissolved in 30ml water acetonitrile mixture (3:1) and sodium hydrogen carbonate was added to adjust the pH to 8.5. The reaction mixture was stirred at ambient temperature and the solvent was removed by lyophilization. The product was isolated by preparative RP-HPLC (gradient 0 to 40% B). After work up, 11.10g (88.7%) of Smoc-D-leucine **17** were obtained as a white powder.

RP-HPLC (0 to 40% B): t_R = 17.70 min.

ESI-MS calc. for $C_{21}H_{23}NO_{10}S_2$ m/z: 513.53, meas. 512.07 $[M-H]^-$; calc. 256.77, meas. 255.84 $[M-2H]^{2-}$

HR-MS calc. for $C_{15}H_{11}ClO_8S_2$ m/z: 512.0691, meas. 512.0739 $[M-H]^-$.

^{1}H NMR (500 MHz, DMSO-d_6) δ: 0.82 (d, *J* = 6.6 Hz, 3H), 0.85 (d, *J* = 6.6 Hz, 3H), 1.41 (ddd, *J* = 13.7, 9.0, 4.9 Hz, 1H), 1.52 (ddd, *J* = 13.7, 10.1, 5.1 Hz, 1H), 1.61 (m, 1H), 3.92 (dd, *J* = 9.7, 4.8 Hz, 1H), 4.23 (t, *J* = 5.6 Hz, 1H), 4.39 (dd, *J* = 11.0, 5.6 Hz, 1H), 4.47 (dd, *J* = 11.0, 5.7 Hz, 1H), 7.51 (br, NH), 7.69 (d, *J* = 8.0, 2H), 7.85 (dd, *J* = 8.0, 1.5 Hz, 2H), 7.92 (s, 1H), 7.93 (s, 1H).

^{13}C NMR (126 MHz, DMSO) δ: 21.33, 22.83, 24.23, 39.56, 47.12, 52.32, 65.35, 119.56, 122.07, 125.36, 140.61, 144.14, 144.23, 146.96, 146.99, 156.33, 174.23.

1.2.17. Synthesis of Smoc-L-Lys(Boc)-OH 18

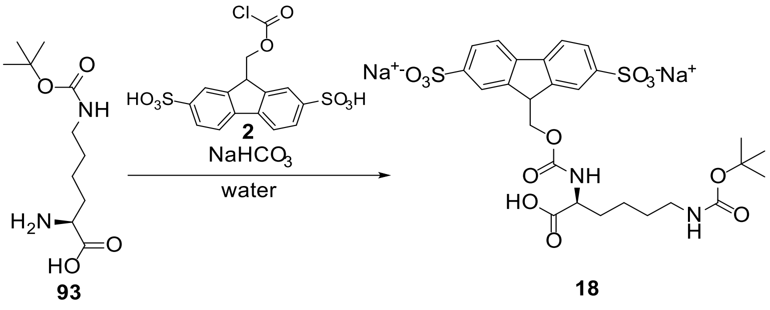

Figure S17: Synthesis of Smoc-L-Lys(Boc)-OH **18**.

According to the general procedure, 5.53g (22.45 mmol, 1 eq.) L-Lys(Boc)-OH **93** and 9.36g (22.45 mmol, 1 eq.) Smoc-Cl **2** were dissolved in 30ml water and sodium hydrogen carbonate was added to adjust the pH to 8.5. The reaction mixture was stirred at ambient temperature and the solvent was removed by lyophilization. The product was isolated by preparative RP-HPLC (0 to 60% B). After work up, 13.20g (87.4%) of Smoc-L-Lys(Boc)-OH **18** were obtained as a white powder.

RP-HPLC (0 to 60% B): t_R = 16.96 min.

ESI-MS calc. for $C_{26}H_{32}N_2O_{12}S_2$ m/z: 628.66, meas. 627.09 $[M-H]^-$; calc. 314.33, meas. 313.35 $[M-2H]^{2-}$.

HR-MS calc. for $C_{26}H_{32}N_2O_{12}S_2$ m/z: 629.14694, meas. 629.14704 $[M+H]^+$.

^{1}H NMR (500 MHz, DMSO-d_6) δ: 1.22 (m, 2H), 1.33 (m, 11H), 1.54 (m, 1H), 1.64 (dq, *J* = 10.3, 5.4 Hz, 1H), 2.86 (dt, *J* = 7.4, 6.9 Hz, 2H), 3.61 (q, *J* = 6.1 Hz, 1H), 4.23 (m, 2H), 4.24 (t, 1H), 6.56 (br, NH), 6.69 (br, NH), 7.68 (dd, *J* = 7.9, 1.4 Hz, 2H), 7.82 (d, *J* = 7.9 Hz, 2H), 7.88 (s, 1H), 7.93 (s, 1H).

^{13}C NMR (126 MHz, DMSO) δ: 22.53, 28.24, 29.60, 32.44, 40.03, 46.75, 55.58, 65.30, 77.14, 119.33, 122.26, 122.38, 125.22, 140.30, 143.92, 144.08, 147.24, 147.31, 155.24, 155.45, 173.58.

1.2.18. Synthesis of Smoc-L-Met-OH 19

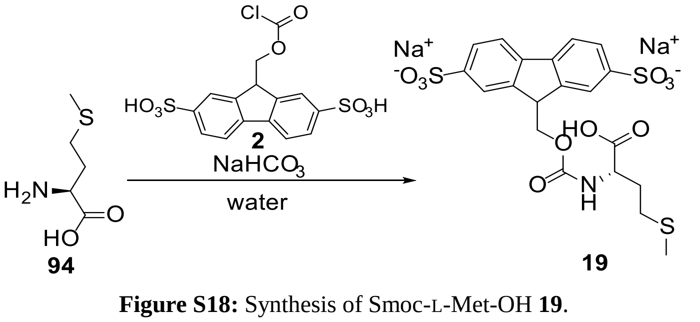

Figure S18: Synthesis of Smoc-L-Met-OH **19**.

According to the general procedure, 3.35g (22.45 mmol, 1 eq.) L-methionine **94** and 9.36g (22.45 mmol, 1 eq.) Smoc-Cl **2** were dissolved in 30ml water and sodium hydrogen carbonate was added to adjust the pH to 8.5. The reaction mixture was stirred at ambient temperature and the solvent was removed by lyophilization. The product was isolated by preparative RP-HPLC (gradient 0 to 40% B). After work up, 12.29g (95.1%) of Smoc-L-Met-OH **19** were obtained as a white powder.

RP-HPLC (0 to 40% B): t_R = 15.27 min.

ESI-MS calc. for $C_{20}H_{21}NO_{10}S_3$ m/z: 531.57, meas. 530.10 $[M-H]^-$; calc. 265.79, meas. 264.80 $[M-2H]^{2-}$

HR-MS calc. for $C_{20}H_{21}NO_{10}S_3$ m/z: 432.04004, meas. 432.04046 $[M+H]^+$.

^{1}H NMR (500 MHz, DMSO-d_6) δ: 1.87 (m, 2H), 2.01 (s, 3H), 2.47 (m, 2H), 4.03 (dt, J = 7.7, 5.6 Hz, 1H), 4.24 (t, J = 5.7 Hz, 1H), 4.38 (dd, J = 10.9, 5.6 Hz, 1H), 4.47 (dd, J = 10.9, 5.9 Hz, 1H), 7.60 (d, J = 7.6 Hz, NH), 7.69 (dd, J = 7.9, 1.2 Hz, 2H), 7.84 (d, J = 7.9 Hz, 2H), 7.91 (s, 1H), 7.92 (s, 1H).

^{13}C NMR (126 MHz, DMSO) δ: 14.53, 29.83, 30.31, 47.06, 52.93, 65.33, 119.53, 122.10, 125.36, 140.56, 144.02, 144.18, 156.33, 173.50.

1.2.19. Synthesis of Smoc-L-Phe-OH 20

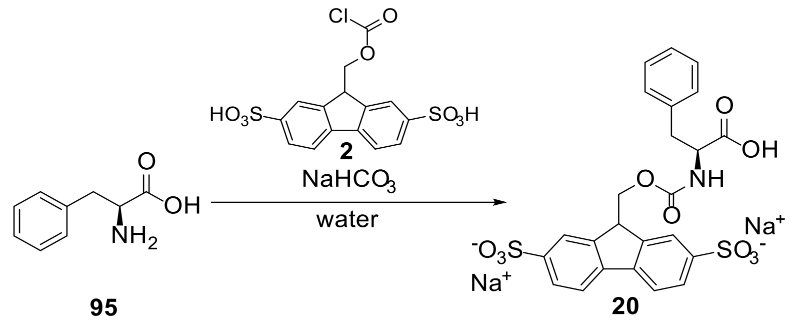

Figure S19: Synthesis of Smoc-L-Phe-OH **20**.

According to the general procedure, 3.71g (22.45 mmol, 1 eq.) L-Phe **95** and 9.36g (22.45 mmol, 1 eq.) Smoc-Cl **2** were dissolved in 30ml water and sodium hydrogen carbonate was added to adjust the pH to 8.5. The reaction mixture was stirred at ambient temperature and the solvent was removed by lyophilization. The product was isolated by preparative RP-HPLC (gradient 0 to 40% B). After work up, 12.44g (93.7%) of Smoc-L-Phe-OH **20** were obtained as a white powder.

RP-HPLC (0 to 40% B): t_R = 18.32 min.

ESI-MS calc. for $C_{24}H_{21}NO_{10}S_2$ m/z: 547.55, meas. 546.20 $[M-H]^-$; calc. 273.76, meas. 272.80 $[M-2H]^{2-}$

HR-MS calc. for $C_{24}H_{21}NO_{10}S_2$ m/z: 548.06796, meas. 548.06857 $[M+H]^+$.

^{1}H NMR (500 MHz, DMSO-d_6) δ: 2.88 (dd, J = 13.8, 9.5 Hz, 1H), 2.99 (dd, J = 13.8, 5.3 Hz, 1H), 4.12 (m, 1H), 4.16 (t, J = 5.5 Hz, 1H), 4.33 (dd, J = 11.0, 5.8 Hz, 1H), 4.38 (dd, J = 11.0, 5.7 Hz, 1H), 7.15 (t, J = 7.2 Hz, 1H), 7.22 (d, J = 7.4 Hz, 2H), 7.26 (t, J = 7.5 Hz, 2H), 7.61 (d, J = 8.1 Hz, NH), 7.69 (dd, J = 7.9, 1.2 Hz, 2H), 7.83 (d, J = 7.9 Hz, 2H), 7.90 (s, 2H).

^{13}C NMR (126 MHz, DMSO) δ: 36.43, 47.05, 55.65, 65.37, 119.43, 122.02, 122.08, 125.31, 126.22, 128.19, 129.06, 137.81, 140.44, 143.99, 144.05, 147.20, 156.05, 173.02.

1.2.20. Synthesis of Smoc-L-Pro-OH 21

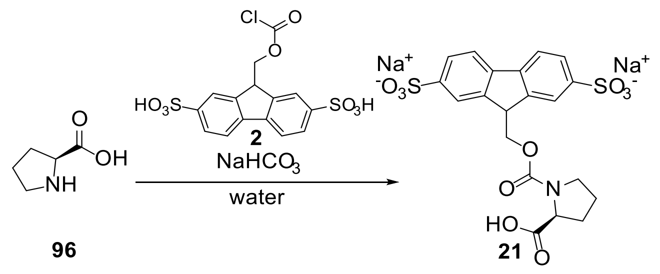

Figure S20: Synthesis of Smoc-L-Pro-OH **21**.

According to the general procedure, 2.58g (22.45 mmol, 1 eq.) L-Pro **96** and 9.36g (22.45 mmol, 1 eq.) Smoc-Cl **2** were dissolved in 30ml water and sodium hydrogen carbonate was added to adjust the pH to 8.5. The reaction mixture was stirred at ambient temperature and the solvent was removed by

lyophilization. The product was isolated by preparative RP-HPLC (gradient 0 to 40% B). After work up, 10.43g (85.8%) of Smoc-L-Pro-OH **21** were obtained as a white powder.

RP-HPLC (0 to 40% B): t_R = 13.50 min.

ESI-MS calc. for $C_{20}H_{19}NO_{10}S_2$ m/z: 497.49, meas. 496.17 $[M-H]^-$; calc. 248.75, meas. 247.84 $[M-2H]^{2-}$

HR-MS calc. for $C_{20}H_{19}NO_{10}S_2$ m/z: 498.05231, meas. 498.05248 $[M+H]^+$.

^{1}H NMR (500 MHz, DMSO-d_6) δ: 1.85 (m, 3H), 2.21 (m, 1H), 3.41 (m, 2H), 4.17 (dd, J = 8.6, 3.2 Hz, 1H), 4.20 – 4.30 (m, 2H), 4.29 (t, J = 5.6 Hz, 1H), 7.68 (dd, J = 7.9, 1.4 Hz, 2H), 7.84 (d, J = 7.9 Hz, 2H), 7.91 (d, J = 1.4 Hz, 1H), 7.92 (d, J = 1.4 Hz, 1H).

^{13}C NMR (126 MHz, DMSO) δ: 23.90, 29.34, 46.47, 58.85, 119.49, 122.42, 125.30, 140.43, 143.66, 143.90, 147.17, 153.84, 173.50.

1.2.21. Synthesis of Smoc-L-Ser-OH 22

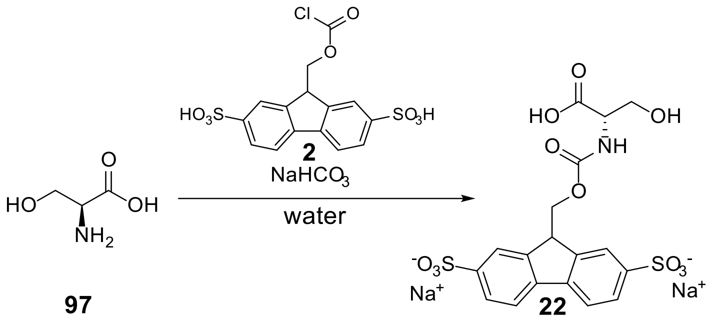

Figure S21: Synthesis of Smoc-L-Ser-OH **22**.

According to the general procedure, 2.36g (22.45 mmol, 1 eq.) L-Ser-OH **97** and 9.36g (22.45 mmol, 1 eq.) Smoc-Cl **2** were dissolved in 30ml water and sodium hydrogen carbonate was added to adjust the pH to 8.5. The reaction mixture was stirred at ambient temperature and the solvent was removed by lyophilization. The product was isolated by preparative RP-HPLC (gradient 0 to 40% B). After work up, 10.74g (90.0%) of Smoc-L-Ser-OH **22** were obtained as a white powder.

RP-HPLC (0 to 40% B): t_R = 11.30 min.

ESI-MS calc. for $C_{18}H_{17}NO_{11}S_2$ m/z: 487.45, meas. 486.20 $[M-H]^-$; calc. 243.73, meas. 242.80 $[M-2H]^{2-}$

HR-MS calc. for $C_{18}H_{17}NO_{11}S_2$ m/z: 488.03158, meas. 488.03176 $[M+H]^+$.

^{1}H NMR (500 MHz, DMSO-d_6) δ: 3.62 (dd, J = 11.3, 5.3 Hz, 1H), 3.65 (dd, J = 11.3, 4.2 Hz, 1H), 4.00 (t, J = 5.2 Hz, 1H), 4.01 (br, OH), 4.25 (t, J = 5.7 Hz, 1H), 4.41 (dd, J = 11.0, 5.6 Hz, 1H), 4.47 (dd, J = 11.0, 6.0 Hz, 1H), 7.14 (br, NH), 7.69 (dd, J = 7.9, 1.5 Hz, 2H), 7.84 (d, J = 7.9 Hz, 2H), 7.90 (s, 1H), 7.92 (s, 1H).

^{13}C NMR (126 MHz, DMSO) δ: 46.96, 56.72, 61.27, 65.29, 119.53, 122.00, 122.07, 125.32, 140.54, 143.92, 144.20, 147.10, 156.01, 171.94.

1.2.22. Synthesis of Smoc-L-Ser(tBu)-OH 23

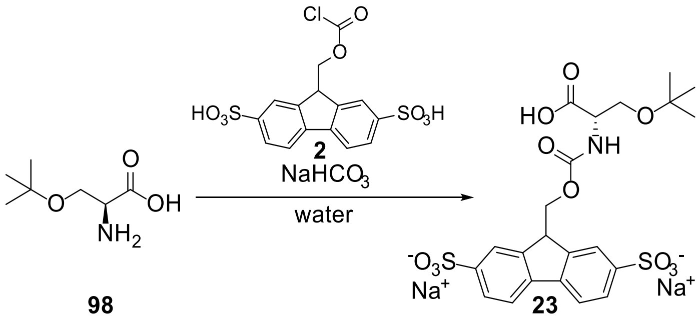

Figure S22: Synthesis of Smoc-L-Ser(tBu)-OH **23**.

According to the general procedure, 3.62g (22.45 mmol, 1 eq.) L-Ser(tBu)-OH **98** and 9.36g (22.45 mmol, 1 eq.) Smoc-Cl **2** were dissolved in 30ml water and sodium hydrogen carbonate was added to adjust the pH to 8.5. The reaction mixture was stirred at ambient temperature and the solvent was removed by lyophilization. The product was isolated by preparative RP-HPLC (gradient 0 to 60% B). After work up, 11.59g (87.9%) of Smoc-L-Ser(tBu)-OH **23** were obtained as a white powder.

RP-HPLC (0 to 60% B): t_R = 14.36 min.

ESI-MS calc. for $C_{22}H_{25}NO_{11}S_2$ m/z: 543.56, meas. 542.08 $[M-H]^-$; calc. 271.78, meas. 270.84 $[M-2H]^{2-}$

HR-MS calc. for $C_{22}H_{25}NO_{11}S$ m/z: 544.09418, meas. 544.09431 $[M+H]^+$.

^{1}H NMR (500 MHz, DMSO-d_6) δ: 1.10 (s, 9H), 3.54 (dd, J = 9.5, 4.9 Hz, 1H), 3.57 (dd, J = 9.5, 5.5 Hz, 1H), 4.08 (dt, J = 8.0, 5.2 Hz, 1H), 4.23 (t, J = 5.9 Hz, 1H), 4.37 (dd, J = 10.9, 5.9 Hz, 1H), 4.42 (dd, J = 10.9, 6.0 Hz, 1H), 7.17 (d, J = 8.1 Hz, NH), 7.69 (d, J = 7.9 Hz, 2H), 7.82 (d, J = 7.9 Hz, 2H), 7.92 (s, 2H).

^{13}C NMR (126 MHz, DMSO) δ: 27.14, 46.94, 54.90, 61.23, 65.60, 72.78, 119.32, 122.08, 125.29, 140.33, 143.87, 143.94, 147.43, 156.01, 171.79.

1.2.23. Synthesis of Smoc-L-Thr-OH 24

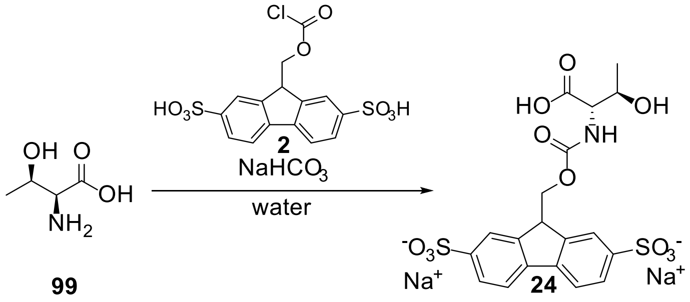

Figure S23: Synthesis of Smoc-L-Thr-OH **24**.

According to the general procedure, 2.67g (22.45 mmol, 1 eq.) L-Thr-OH **99** and 9.36g (22.45 mmol, 1 eq.) Smoc-Cl **2** were dissolved in 30ml water and sodium hydrogen carbonate was added to adjust the pH to 8.5. The reaction mixture was stirred at ambient temperature and the solvent was removed by lyophilization. The product was isolated by preparative RP-HPLC (0 to 40% B). After work up, 11.30 (92.2%) of Smoc-L-Thr-OH **24** were obtained as a white powder.

RP-HPLC (gradient 0 to 40% B): t_R = 12.07 min.

ESI-MS calc. for $C_{19}H_{19}NO_{11}S_2$ m/z: 501.48, meas. 500.07 $[M-H]^-$; calc. 250.74, meas. 249.84 $[M-2H]^{2-}$

HR-MS calc. for $C_{19}H_{19}NO_{11}S_2$ m/z: 502.04723, meas. 502.04749 $[M+H]^+$.

^{1}H NMR (500 MHz, DMSO-d_6) δ: 1.07 (d, J = 6.5 Hz, 3H), 3.92 (dd, J = 8.1, 3.8 Hz, 1H), 4.02 (qd, J = 6.5, 3.7 Hz, 1H), 4.25 (t, J = 6.0 Hz, 1H), 4.39 (dd, J = 10.9, 5.7 Hz, 1H), 4.46 (dd, J = 10.9, 6.1 Hz, 1H), 6.83 (d, J = 8.7 Hz, NH), 7.69 (d, J = 7.9 Hz, 2H), 7.83 (d, J = 7.9 Hz, 2H), 7.91 (s, 1H), 7.92 (s, 1H).

^{13}C NMR (126 MHz, DMSO) δ: 20.32, 46.95, 60.18, 65.52, 66.44, 119.43, 122.07, 125.30, 140.44, 143.86, 144.07, 147.22, 156.38, 172.09.

1.2.24. Synthesis of Smoc-L-Thr(tBu)-OH 25

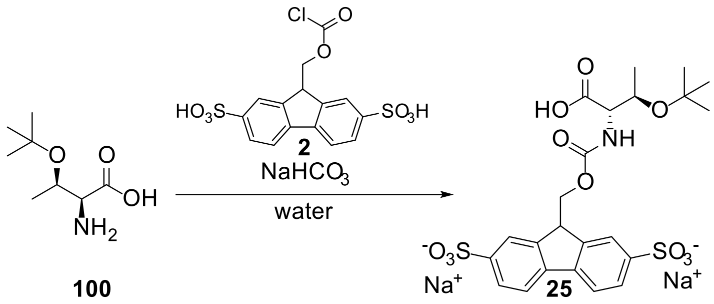

Figure S24: Synthesis of Smoc-L-Thr(tBu)-OH **25**.

According to the general procedure, 3.93g (22.45 mmol, 1 eq.) L-Thr(tBu)-OH **100** and 9.36g (22.45 mmol, 1 eq.) Smoc-Cl **2** were dissolved in 30ml water and sodium hydrogen carbonate was added to adjust the pH to 8.5. The reaction mixture was stirred at ambient temperature and the solvent was removed by lyophilization. The product was isolated by preparative RP-HPLC (0 to 60% B). After work up, 12.05 (89.2%) of Smoc-L-Thr(tBu)-OH **25** were obtained as a white powder.

RP-HPLC (0 to 60% B): t_R = 15.14 min.

ESI-MS calc. for $C_{23}H_{27}NO_{11}S_2$ m/z: 557.59, meas. 556.08 $[M-H]^-$; calc. 278.80, meas. 277.74 $[M-2H]^{2-}$

HR-MS calc. for $C_{23}H_{27}NO_{11}S_2$ m/z: 580.09177 meas. 580.09193 $[M+Na]^+$; m/z: calc. 596.06571 meas. 596.06589 $[M+K]^+$.

^{1}H NMR (500 MHz, DMSO-d_6) δ: 1.05 (d, J = 6.2 Hz, 3H), 1.10 (s, 9H), 3.59 (dd, J = 8.7, 3.3 Hz, 1H), 3.96 (dq, J = 6.2, 3.4 Hz, 1H), 4.23 (m, 3H), 6.18 (d, J = 8.7 Hz, NH), 7.69 (dd, J = 7.9, 1.5 Hz, 2H), 7.83 (d, J = 7.9 Hz, 2H), 7.90 (s, 1H), 7.96 (s, 1H).

^{13}C NMR (126 MHz, DMSO) δ: 21.08, 28.56, 46.54, 61.82, 65.58, 68.37, 72.43, 119.56, 122.60, 122.70, 125.55, 140.28, 143.95, 147.19, 156.10, 173.00.

1.2.25. Synthesis of Smoc-L-Trp-OH 26

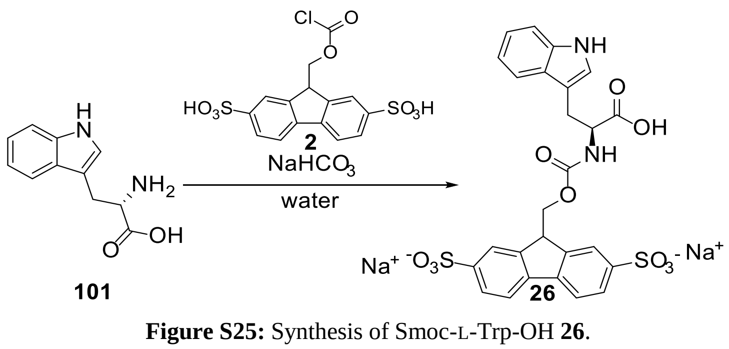

Figure S25: Synthesis of Smoc-L-Trp-OH **26**.

According to the general procedure, 4.48g (22.45 mmol, 1 eq.) L-Trp-OH **101** and 9.36g (22.45 mmol, 1 eq.) Smoc-Cl **2** were dissolved in 30ml water and sodium hydrogen carbonate was added to adjust the pH to 8.5. The reaction mixture was stirred at ambient temperature and the solvent was removed by

lyophilization. The product was isolated by preparative RP-HPLC (gradient 0 to 40% B). After work up, 12.84g (90.7%) of Smoc-Trp-OH **26** were obtained as a white powder.

RP-HPLC (0 to 40% B): t_R = 19.55 min.

ESI-MS calc. for $C_{26}H_{22}N_2O_{10}S_2$ m/z: 586.59, meas. 585.20 $[M-H]^-$; calc. 293.30, meas. 292.30 $[M-2H]^{2-}$.

HR-MS calc. for $C_{26}H_{22}N_2O_{10}S_2$ m/z: 587.07886, meas. 587.07914 $[M+H]^+$.

^{1}H NMR (500 MHz, DMSO-d_6) δ: 2.95 (dd, *J* = 14.6, 9.9 Hz, 1H), 3.16 (dd, *J* = 14.6, 4.5 Hz, 1H), 4.12 (dt, *J* = 9.0, 4.5 Hz, 1H), 4.21 (t, *J* = 5.8 Hz, 1H), 4.29 (dd, *J* = 10.8, 5.5 Hz, 1H), 4.40 (dd, *J* = 10.8, 6.1 Hz, 1H), 6.96 (t, *J* = 7.4 Hz, 1H), 7.03 (t, *J* = 7.5 Hz, 1H), 7.14 (s, 1H), 7.29 (d, *J* = 7.5 Hz, 1H), 7.48 (d, *J* = 7.8 Hz, 1H), 7.53 (d, *J* = 8.2 Hz, 1H), 7.69, 7.70 (dd, *J* = 7.9, 2.5 Hz, 2H), 7.82, 7.83 (dd, *J* = 7.9, 2.5 Hz, 2H), 7.90 (s, 1H), 7.93 (s, 1H), 10.87 (s, NH).

^{13}C NMR (126 MHz, DMSO) δ: 26.53, 45.18, 46.90, 65.28, 109.67, 111.33, 117.81, 118.18, 119.54, 120.64, 122.02, 124.31, 125.32, 125.35, 127.04, 135.84, 140.54, 140.57, 143.96, 144.25, 147.00, 147.07, 155.96, 173.47.

1.2.26. Synthesis of Smoc-L-Trp(Boc)-OH 27

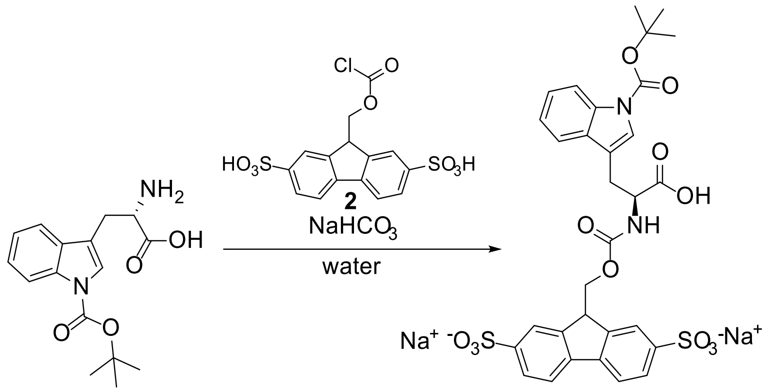

Figure S26: Synthesis of Smoc-L-Trp(Boc)-OH **27**.

According to the general procedure, 3.00g (9.86 mmol, 1 eq.) L-Trp(Boc)-OH **102** and 4.11g (9.86 mmol, 1 eq.) Smoc-Cl **2** were dissolved in 30ml water and sodium hydrogen carbonate was added to adjust the pH to 8.5. The reaction mixture was stirred at ambient temperature and the solvent was removed by lyophilization. The product was isolated by preparative RP-HPLC (gradient 0 to 80% B). After work up, 6.26g (86.9%) of Smoc-Trp(Boc)-OH **27** were obtained as a white powder.

RP-HPLC (0 to 100% B): t_R = 15.14 min.

ESI-MS calc. for $C_{31}H_{30}N_2O_{12}S_2$ m/z: 686.70, meas. 685.08 $[M-H]^-$; calc. 343.35, meas. 342.36 $[M-2H]^{2-}$.

HR-MS calc. for $C_{31}H_{30}N_2O_{12}S_2$ m/z: 685.11679, meas. 685.11674 $[M-H]^-$.

^{1}H NMR (500 MHz, DMSO-d_6) δ: 1.61 (s, 9H), 2.97 (dd, *J* = 14.6, 6.3 Hz, 1H), 3.16 (dd, *J* = 14.6, 5.4 Hz, 1H), 3.93 (q, *J* = 6.1 Hz, 1H), 4.18 (s, 3H), 6.72 (d, *J* = 6.8 Hz, 1H), 7.21 (t, *J* = 7.4 Hz, 2H), 7.26 (t, *J* = 7.7 Hz, 1H), 7.43 (s, 1H), 7.64 (d, *J* = 7.7 Hz, 1H), 7.69 (d, *J* = 7.9 Hz, 4H), 7.82 (d, *J* = 7.9 Hz, 3H), 7.87 (s, 1H), 7.94 (s, 1H), 8.00 (d, *J* = 8.2 Hz, 1H).

^{13}C NMR (126 MHz, DMSO) δ: 27.4, 27.69, 55.99, 65.18, 83.24, 110.23, 114.30, 118.13, 118.55, 119.29, 119.37, 122.21, 122.30, 122.5, 123.48, 123.91, 125.2, 130.90, 134.56, 137.32, 138.88, 142.08, 147.77, 149.10, 173.45.

1.2.27. Synthesis of Smoc-L-Tyr-OH 28

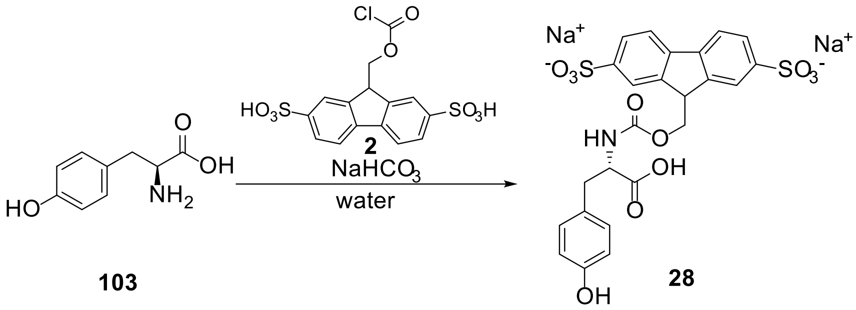

103

28

Figure S27: Synthesis of Smoc-L-Tyr-OH **28.**

According to the general procedure, 4.07g (22.45 mmol, 1 eq.) L-Tyr-OH **103** and 9.36g (22.45 mmol, 1 eq.) Smoc-Cl **2** were dissolved in 30ml water and sodium hydrogen carbonate was added to adjust the pH to 8.5. The reaction mixture was stirred at ambient temperature and the solvent was removed by lyophilization. The product was isolated by preparative RP-HPLC (gradient 0 to 40% B). After work up, 12.24g (89.7%) of Smoc-L-Tyr-OH **28** were obtained as a white powder.

RP-HPLC (0 to 40% B): t_R = 15.65 min.

ESI-MS calc. for $C_{24}H_{21}NO_{11}S_2$ m/z: 563.55, meas. 562.20 $[M-H]^-$; calc. 281.77, meas. 280.80 $[M-2H]^{2-}$

HR-MS calc. for $C_{24}H_{21}NO_{11}S_2$ m/z: 564.06288, meas. 564.06314 $[M+H]^+$; m/z: calc. 586.04482 meas. 586.04494 $[M+Na]^+$.

^{1}H NMR (500 MHz, DMSO-d_6) δ: 2.74 (dd, *J* = 13.9, 9.3 Hz, 1H), 2.86 (dd, *J* = 13.9, 5.4 Hz, 1H), 4.03 (q, *J* = 7.1 Hz, 1H), 4.18 (t, *J* = 5.7 Hz, 1H), 4.33 (dd, *J* = 11.0, 5.8 Hz, 1H), 4.39 (dd, *J* = 11.0, 5.7 Hz, 1H), 6.64 (d, *J* = 8.1 Hz, 2H), 6.98 (d, *J* = 8.0 Hz, 2H), 7.50 (d, *J* = 8.0 Hz, NH), 7.69 (d, *J* = 8.0 Hz, 2H), 7.84 (d, *J* = 7.9 Hz, 2H), 7.90 (s, 2H), 8.15 (s, OH).

^{13}C NMR (126 MHz, DMSO) δ: 35.74, 47.06, 56.00, 65.33, 115.05, 119.50, 122.05, 125.34, 127.80, 129.97, 140.52, 144.10, 147.06, 147.13, 155.77, 156.05, 173.19.

1.2.28. Synthesis of Smoc-L-Tyr(tBu)-OH 29

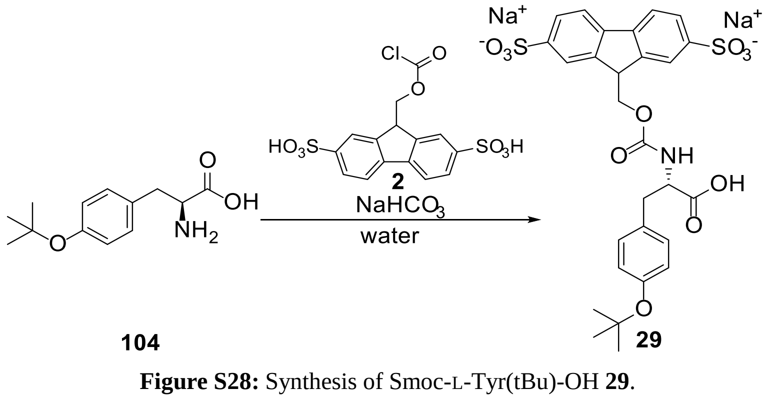

104

29

Figure S28: Synthesis of Smoc-L-Tyr(tBu)-OH **29**.

According to the general procedure, 5.33g (22.45 mmol, 1 eq.) L-Tyr(tBu)-OH **104** and 9.36g (22.45 mmol, 1 eq.) Smoc-Cl **2** were dissolved in 30ml water and sodium hydrogen carbonate was added to adjust the pH to 8.5. The reaction mixture was stirred at ambient temperature and the solvent was removed by lyophilization. The product was isolated by preparative RP-HPLC (gradient 0 to 60% B). After work up, 13.61g (91.4%) of Smoc-L-Tyr(tBu)-OH **29** were obtained as a white powder.

RP-HPLC (0 to 60% B): t_R = 17.95 min.

ESI-MS calc. for $C_{28}H_{29}NO_{11}S_2$ m/z: 619.66, meas. 618.09 $[M-H]^-$; calc. 309.83, meas. 308.85 $[M-2H]^{2-}$

HR-MS calc. for $C_{28}H_{29}NO_{11}S_2$ m/z: 620.12548, meas. 620.12539 $[M+H]^+$.

^{1}H NMR (500 MHz, DMSO-*d*$_6$) δ: 1.21 (s, 9H), 2.79 (dd, *J* = 13.6, 7.1 Hz, 1H), 3.00 (dd, *J* = 13.6, 4.9 Hz, 1H), 3.83 (q, *J* = 6.6 Hz, 1H), 4.14 (m, 3H), 6.51 (d, *J* = 7.2 Hz, NH), 6.78 (d, *J* = 8.0 Hz, 2H), 7.07 (d, *J* = 8.1 Hz, 2H), 7.68 (d, *J* = 8.0 Hz, 2H), 7.81 (d, *J* = 8.0 Hz, 2H), 7.86 (s, 1H), 7.92 (s, 1H).

^{13}C NMR (126 MHz, DMSO) δ: 28.54, 37.56, 46.53, 57.47, 65.34, 77.37, 119.48, 122.71, 123.09, 125.50, 129.71, 134.06, 140.24, 143.89, 147.78, 152.97, 155.31, 173.08.

1.2.29. Synthesis of Smoc-L-Val-OH 30

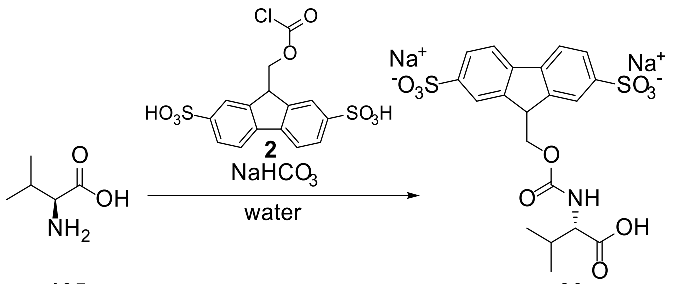

105 30

Figure S29: Synthesis of Smoc-L-Val-OH **30**.

According to the general procedure, 2.63g (22.45 mmol, 1 eq.) L-Val-OH **105** and 9.36g (22.45 mmol, 1 eq.) Smoc-Cl **2** were dissolved in 30ml water and sodium hydrogen carbonate was added to adjust the pH to 8.5. The reaction mixture was stirred at ambient temperature and the solvent was removed by lyophilization. The product was isolated by preparative RP-HPLC (gradient 0 to 40% B). After work up, 10.64g (87.2%) of Smoc-L-Val-OH **30** were obtained as a white powder.

RP-HPLC (0 to 40% B): t_R = 15.04 min.

ESI-MS calc. for $C_{20}H_{21}NO_{10}S_2$ m/z: 499.51, meas. 498.20 $[M-H]^-$; calc. 249.76, meas. 248.80 $[M-2H]^{2-}$

HR-MS calc. for $C_{20}H_{21}NO_{10}S_2$ m/z: 500.06796 meas. 500.06823 $[M+H]^+$.

^{1}H NMR (500 MHz, DMSO-*d*$_6$) δ: 0.87 (d, *J* = 6.8 Hz, 6H), 2.00 (oct, *J* = 13.9 Hz, 1H), 2.86 (dd, *J* = 13.9, 5.4 Hz, 1H), 3.81 (t, *J* = 6.7 Hz, 1H), 4.23 (t, *J* = 5.7 Hz, 1H), 4.38 (dd, *J* = 11.0, 5.7 Hz, 1H), 4.46 (dd, *J* = 11.0, 5.9 Hz, 1H), 7.46 (d, *J* = 8.3 Hz, NH), 7.68 (dd, *J* = 8.0, 1.3 Hz, 2H), 7.83 (d, *J* = 8.0 Hz, 2H), 7.92 (s, 1H), 7.93 (s, 1H).

^{13}C NMR (126 MHz, DMSO) δ: 18.40, 19.08, 29.41, 47.13, 59.82, 65.45, 119.45, 122.10, 125.31, 140.48, 144.02, 144.18, 147.13, 156.53, 173.07.

1.2.30. Synthesis of Smoc-β-Ala-OH 31

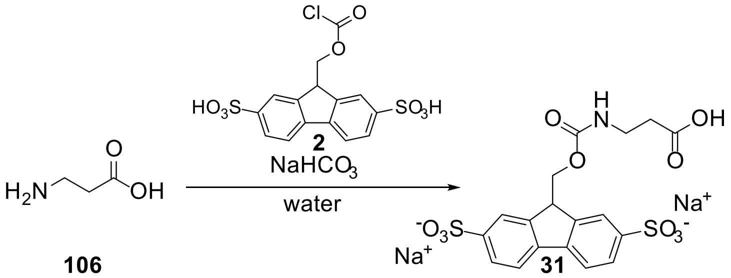

106 31

Figure S30: Synthesis of Smoc-β-Ala-OH **31**.

According to the general procedure, 2.03g (22.45 mmol, 1 eq.) β-Ala-OH **106** and 9.36g (22.45 mmol, 1 eq.) Smoc-Cl **2** were dissolved in 30ml water and sodium hydrogen carbonate was added to adjust the pH to 8.5. The reaction mixture was stirred at ambient temperature and the solvent was removed by lyophilization. The product was isolated by preparative RP-HPLC (gradient 0 to 40% B). After work up, 10.71g (92.5%) of Smoc-β-Ala-OH **31** were obtained as a white powder.

RP-HPLC (0 to 40% B): t_R = 12.12 min.

ESI-MS calc. for $C_{18}H_{17}NO_{10}S_2$ m/z: 471.45, meas. 470.20 $[M-H]^-$; calc. 235.73, meas. 234.80 $[M-2H]^{2-}$

HR-MS calc. for $C_{18}H_{17}NO_{10}S_2$ m/z: 470.02211, meas. 470.02236 $[M-H]^-$.

^{1}H NMR (500 MHz, DMSO-d_6) δ: 2.36 (t, *J* = 7.3 Hz, 2H), 3.14 (t, *J* = 7.4 Hz, 2H), 4.22 (t, *J* = 5.7 Hz, 1H), 4.42 (d, *J* = 5.7 Hz, 2H), 7.19 (br, NH), 7.67 (d, *J* = 8.0 Hz, 2H), 7.81 (d, *J* = 7.9 Hz, 2H), 7.87 (s, 2H).

^{13}C NMR (126 MHz, DMSO) δ: 34.01, 36.48, 47.00, 64.86, 119.34, 121.92, 125.21, 140.39, 144.01, 147.28, 155.99, 173.58.

1.2.31. Synthesis of Smoc-Aib-OH 32

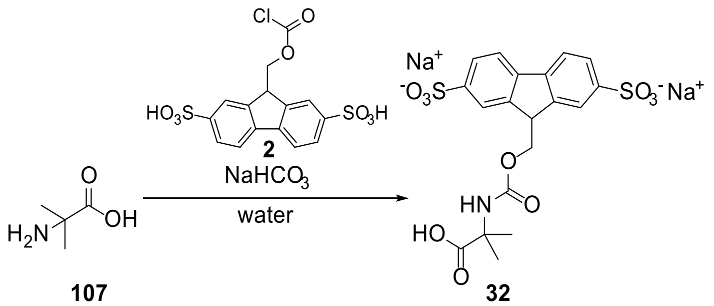

Figure S31: Synthesis of Smoc-Aib-OH **32**.

According to the general procedure, 1.00g (9.55 mmol, 1 eq.) 2-aminoisobutyric acid **107** and 3.98g (9.55 mmol, 1 eq.) Smoc-Cl **2** were dissolved in 30ml water acetonitrile mixture (1:1) and sodium hydrogen carbonate was added to adjust the pH to 8.5. The reaction mixture was stirred at ambient temperature and the solvent was removed by lyophilization. The product was isolated by preparative RP-HPLC (50 to 100% B). After work up, 2.93g (57.8%) of Smoc-Aib-OH **32** were obtained as a white powder.

RP-HPLC (0 to 40% B): t_R = 13.49 min.

ESI-MS calc. for $C_{19}H_{19}NO_{10}S_2$ m/z: 485.48, meas. 484.20 $[M-H]^-$; calc. 242.74, meas. 241.80 $[M-2H]^{2-}$

HR-MS calc. for $C_{19}H_{19}NO_{10}S_2$ m/z: 484.03776, meas. 484.03817 $[M-H]^-$.

^{1}H NMR (500 MHz, DMSO-d_6) δ: 1.29 (s, 6H), 4.21 (t, *J* = 5.5 Hz, 1H), 4.41 (d, *J* = 5.3 Hz, 2H), 7.44 (br, NH), 7.67 (d, *J* = 7.8 Hz, 2H), 7.82 (d, *J* = 7.8 Hz, 2H), 7.89 (s, 2H).

^{13}C NMR (126 MHz, DMSO) δ: 24.97, 47.05, 55.20, 64.80, 119.35, 121.97, 125.23, 140.43, 144.04, 147.23, 155.06, 175.60.

1.3. NMR studies

All NMR spectra were measured on a Bruker DRX 500 MHz spectrometer equipped with a room temperature 5 mm ATMA BBFO probe (Bruker Biospin, Karlsruhe, Germany) at 303 K. All samples were dissolved in deuterated DMSO purchased from Sigma Aldrich (Merck KGaA, Darmstadt, Germany) or deuterated CH_3CN purchased from Eurisotop (Gif-Sur-Yvette, France). The concentrations of the samples were ~ 10^{-2} mol/l. The solvent signals were used for referencing the ^{1}H-

and ^{13}C-NMR spectra at 2.5 ppm and 39.5 ppm respectively. Chemical shift assignment was achieved with ^{1}H-, ^{13}C- and ^{13}C-DEPT-135 1D spectra, 2D ^{1}H-^{1}H COSY (correlated spectroscopy), NOESY (nuclear overhauser enhancement spectroscopy), 2D ^{1}H-^{13}C HSQC (heteronuclear single quantum correlation) and 2D ^{1}H-^{13}C HMBC (heteronuclear multiple-bond correlation), using the Bruker pulse sequences zg30, zgpg30, dept135, cosygpmfqf, noesygptp, invietgpsi and inv4gplrl2ndqf, respectively. 1D spectra were recorded using an excitation pulse of 30° and a repetition time of 4.5 s (^{1}H) and 1.5 s (^{13}C). 32 scans (^{1}H) and 2.000 scans (^{13}C) were added and Fourier transformed with a final digital resolution of 0.08 Hz (1H, 0.26 Hz ^{13}C). The hetero-nuclear long-range correlation spectrum (HMBC) was recorded by a matrix of 1 k data points (f2, ^{1}H dimension) and 256 increments (data points in f1 ^{13}C dimension). The spectrum has been optimized for a heteronuclear coupling constant of 9 Hz. Raw data were processed with Topspin (Bruker Biospin, Karlsruhe, Germany) and 2D data were analyzed using MestReNova 11.0.3 (Mestrelab Research S.L., Spain). Assessment of NMR results is shown in **Table S1-Table S31**.

Table S1: Smoc-Cl **2** in MeCN-d3 (^{1}H NMR at 500 MHz, ^{13}C NMR at 126 MHz).

Position	^{1}H	#H	Multi-plicity	Coupling constant	^{13}C
1 and 8	8.18	2	s		124.48
2 and 7	-				140.82
3 and 6	8.0	2	d	8.1	128.13
4 and 5	8.08	2	d	8.1	122.98
4' and 5'	-				144.97
1' and 8'	-				145.68
9	4.55	1	t	5.7	47.8
10	4.9	2	d	5.7	72.67
11	-				151.05

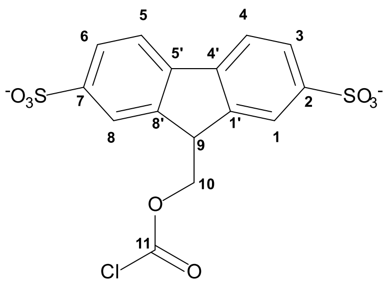

2

Table S2: Smoc-L-Ala-OH **3** in DMSO-d6 (^{1}H NMR at 500 MHz, ^{13}C NMR at 126 MHz).

Position	^{1}H	#H	Multi-plicity	Coupling constant	^{13}C
1 and 8	7.91; 7.89	2	s	-	121.97; 122.01
2 and 7	-	-	-	-	147.16
3 and 6	7.68	2	d	7.9	125.31
4 and 5	7.83	2	d	7.9	119.47
4' and 5'	-	-	-	-	140.52
1' and 8'	-	-	-	-	143.99; 144.17
9	4.23	1	t	5.5	47.02
10a	4.38	1	dd	10.9; 5.7	65.14
10b	4.49	1	dd	10.9; 5.4	
11	-	-	-	-	155.93
NH	7.52	1	br	-	-
12	3.95	1	q	7.3	49.27
13	-	-	-	-	174.27
14	1.22	3	d	7.4	16.83

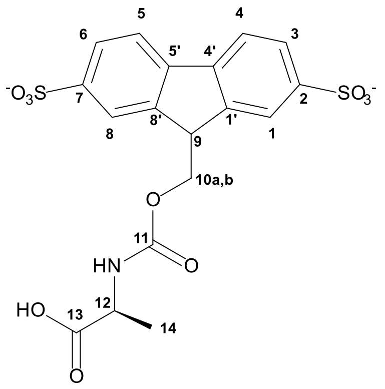

3

Table S3: Smoc-D-Ala-OH **4** in DMSO-d6 (^{1}H NMR at 500 MHz, ^{13}C NMR at 126 MHz).

Position	^{1}H	#H	Multi-plicity	Coupling constant	^{13}C
1 and 8	7.89; 7.91	2	s	-	121.94
2 and 7	-	-	-	-	147.19
3 and 6	7.68	2	d	8.2	125.28
4 and 5	7.83	2	d	7.9	119.43
4' and 5'	-	-	-	-	140.49
1' and 8'	-	-	-	-	143.97; 144.15
9	4.23	1	t	5.5	47.01
10a	4.37	1	dd	11.0; 5.7	65.11
10b	4.38	1	dd	10.9; 5.5	
11	-	-	-	-	155.91
NH	7.5	1	br	-	-
12	3.95	1	q	7.3	49.26
13	-	-	-	-	174.22
14	1.22	3	d	7.4	16.82

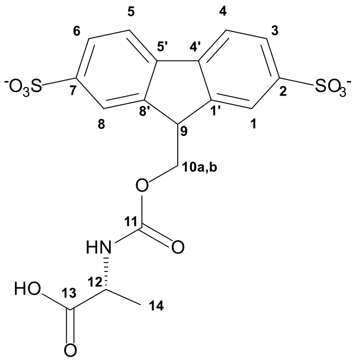

4

Table S4: Smoc-L-Arg-OH **5** in DMSO-d6 (^{1}H NMR at 500 MHz, ^{13}C NMR at 126 MHz).

Position	^{1}H	#H	Multi-plicity	Coupling constant	^{13}C
1 and 8	7.88; 7.93	2	s	-	122.01
2 and 7	-	-	-	-	146.76
3 and 6	7.7	2	d	7.7	125.3
4 and 5	7.85; 7.86	2	d	7.9	119.6
4' and 5'	-	-	-	-	140.61
1' and 8'	-	-	-	-	143.51; 144.50
9	4.32	1	t	5.5	46.91
10a	4.33	1	dd	10.0; 6.3	64.85
10b	4.54	1	dd	10.0; 6.3	
11	-	-	-	-	155.93
NH	7.52	1	d	8.5	-
12	3.96	1	dt	9.2; 4.3	52.91
13	-	-	-	-	173.3
14	1.56; 1.72	2	m	-	27.87
15	1.47	2	m	-	24.93
16a	3.02	1	dq	13.0; 6.5	40.0
16b	3.1	1	dq	13.0; 6.5	
NH	7.74	1	t	5.6	-
17	-	-	-	-	156.63

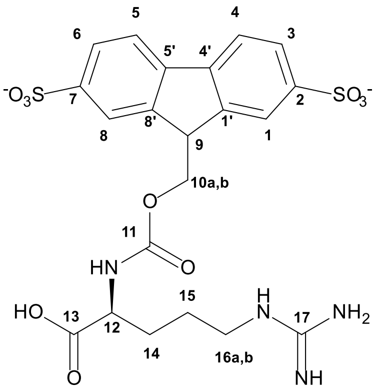

5

Table S5: Smoc-L-Arg(Pbf)-OH **6** in DMSO-d6 (^{1}H NMR at 500 MHz, ^{13}C NMR at 126 MHz).

Position	^{1}H	#H	Multi-plicity	Coupling constant	^{13}C
1 and 8	7.88; 7.91	2	d	1.4	122.03
2 and 7	-	-	-	-	146.91
3 and 6	7.68	2	dd	7.8; 1.4	125.28
4 and 5	7.84	2	d	7.9	119.54

4' and 5'	-	-	-	-	140.55
1' and 8'	-	-	-	-	143.58; 144.39
9	4.3	1	t	5.5	46.92
10a	4.32	1	m	-	64.98
10b	4.51	1	m	-	
11	-	-	-	-	155.96
NH	7.52	1	br	-	-
12	3.9	1	dt	9.1; 4.7	53.17
13	-	-	-	-	173.35
14	1.50; 1.63	2	m	-	27.8
15	1.37	2	m	-	25.1
16	3.07	2	q	7.3	39.71
NH	7.74	1	t	5.6	-
17	-	-	-	-	166.74
18	-	-	-	-	133.9
19	-	-	-	-	131.42
20	-	-	-	-	124.33
21	-	-	-	-	157.57
22	-	-	-	-	116.34
23	-	-	-	-	137.32
24	2.97	2	s	-	42.35
25	-	-	-	-	86.32
26	1.41	6	s	-	28.25
27	2.43	3	s	-	18.91
28	2.48	3	s	-	17.51
29	2.01	3	s	-	12.22

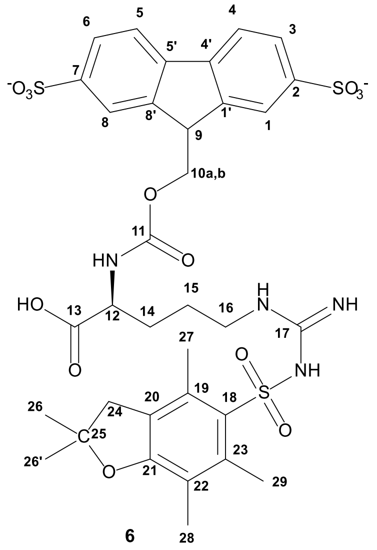

6

Table S6: Smoc-L-Asn-OH **7** in DMSO-d6 (^{1}H NMR at 500 MHz, ^{13}C NMR at 126 MHz).

Position	^{1}H	#H	Multi-plicity	Coupling constant	^{13}C
1 and 8	7.90; 7.91	2	s	-	122.11
2 and 7	-	-	-	-	146.99
3 and 6	7.69	2	d	7.5	125.37
4 and 5	7.84	2	d	7.9	119.57
4' and 5'	-	-	-	-	140.59
1' and 8'	-	-	-	-	144.03; 144.14
9	4.26	1	t	-	46.06
10	4.42	2	m	-	65.25
11	-	-	-	-	155.83
NH	7.4	1	d	8.2	-
12	4.27	1	m	-	50.71
13	-	-	-	-	172.83
14	2.43	1	dd	15.2; 7.5	36.78
14b	2.54	1	dd	15.2; 5.8	
15	-	-	-	-	171.56
NH_2	5.6	2	br	-	-

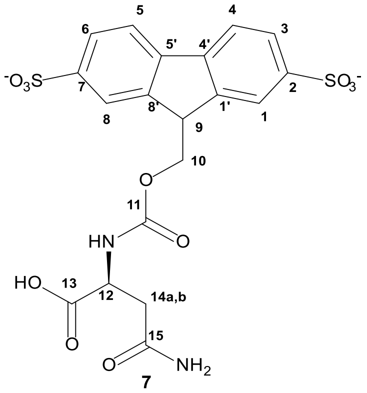

7

Table S7: Smoc-L-Asp(OtBu)-OH **8** in DMSO-d_6 (^{1}H NMR at 500 MHz, ^{13}C NMR at 126 MHz).

Position	^{1}H	#H	Multi-plicity	Coupling constant	^{13}C
1 and 8	7.89; 7.94	2	s	-	122.31; 122.45
2 and 7	-	-	-	-	147.26
3 and 6	7.69	2	d	7.7	125.25
4 and 5	7.83	2	d	7.9	119.35
4' and 5'	-	-	-	-	140.31
1' and 8'	-	-	-	-	143.78; 144.15
9	4.23	1	m	-	46.62
10	4.22	2	m	-	65.47
11	-	-	-	-	155.36
NH	6.8	1	d	7.5	-
12	3.97	1	dt	7.5; 5.3	52.92
13	-	-	-	-	170.72
14	2.4	1	dd	14.7; 7.8	39.54
14b	2.59	1	dd	14.7; 5.4	
15	-	-	-	-	172.58
16	-	-	-	-	79.1
17	1.38	9	s	-	27.78

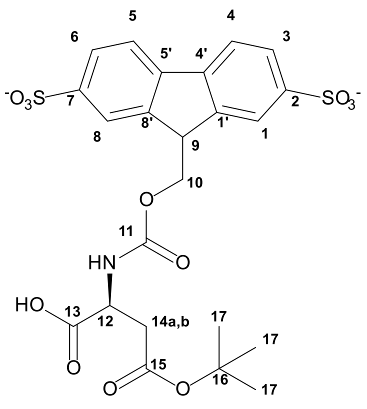

8

Table S8: Smoc-L-Cys(Trt)-OH **9** in DMSO-d_6 (^{1}H NMR at 500 MHz, ^{13}C NMR at 126 MHz).

Position	^{1}H	#H	Multi-plicity	Coupling constant	^{13}C
1 and 8	7.90; 7.91	2	s	-	122.0
2 and 7	-	-	-	-	147.4
3 and 6	7.68	2	d	7.9	125.3
4 and 5	7.82	2	d	7.8	119.35
4' and 5'	-	-	-	-	140.36
1' and 8'	-	-	-	-	143.97
9	4.2	1	t	5.6	47.0
10a	4.37	1	dd	11.1; 5.8	65.57
10b	4.41	1	dd	11.5; 6.5	
11	-	-	-	-	155.94
NH	6.6	1	d	8.4	
12	3.77	1	m	-	53.58
13	-	-	-	-	171.63
14	2.38	1	dd	12.4; 5.0	32.52
14b	2.56	1	dd	12.6; 9.4	
15	-	-	-	-	66.28
16	-	-	-	-	144.21
17	7.3	6	m	-	129.0
18	7.3	6	m	-	128.0
19	7.24	3	t	6.9	126.67

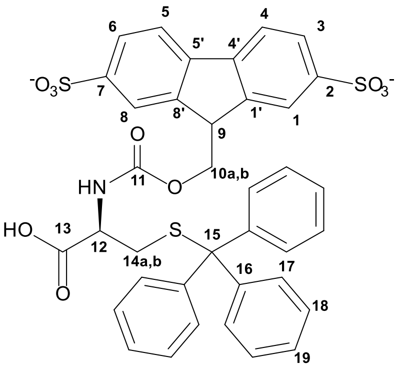

9

Table S9: Smoc-L-Gln-OH **10** in DMSO-d6 (^{1}H NMR at 500 MHz, ^{13}C NMR at 126 MHz).

Position	^{1}H	#H	Multi-plicity	Coupling constant	^{13}C
1 and 8	7.90; 7.93	2	s	-	122.15
2 and 7	-	-	-	-	147.12
3 and 6	7.69	2	d	7.8	125.32
4 and 5	7.84	2	d	7.8	119.48
4' and 5'	-	-	-	-	140.45; 140.50
1' and 8'	-	-	-	-	143.67; 144.32
9	4.27	1	t	6	46.86
10a	4.32	1	dd	10.7; 5.7	65.18
10b	4.45	1	dd	10.7; 6.8	
11	-	-	-	-	156.12
NH	7.54	1	d	7.7	-
12	3.88	1	dt	10.4; 5.0	53.47
13	-	-	-	-	173.68
14a	1.68	1	m	-	26.16
14b	1.99	1	m	-	
15	2.16	2	ddd	9.5; 6.8; 3.2	31.23
16	-	-	-	-	173.58

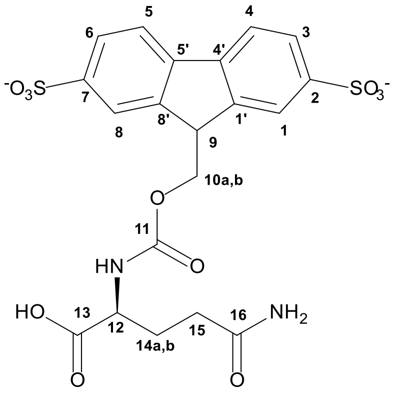

10

Table S10: Smoc-L-Glu(OtBu)-OH **11** in DMSO-d6 (^{1}H NMR at 500 MHz, ^{13}C NMR at 126 MHz).

Position	^{1}H	#H	Multi-plicity	Coupling constant	^{13}C
1 and 8	7.89; 7.94	2	s	-	122.3
2 and 7	-	-	-	-	147.2
3 and 6	7.69	2	d	8.0	125.0
4 and 5	7.83	2	d	8.0	119.3
4' and 5'	-	-	-	-	140.31
1' and 8'	-	-	-	-	143.95
9	4.23	1	m	-	46.2
10	4.23	2	m	-	65.2
11	-	-	-	-	155.5
NH	6.63	1	d	6.7	-
12	3.65	1	q	6.0	54.8
13	-	-	-	-	172.4
14	1.78	1	tt	12.1; 5.6	27.96
14b	1.9	1	tt	10.7; 5.2	
15a	2.12	1	ddd	16.0; 10.8; 5.3	30.73
15b	2.23	1	ddd	16.1; 10.9; 5.5	
16	-	-	-	-	173.5
17	-	-	-	-	79.13
18	1.38	9	s	-	27.73

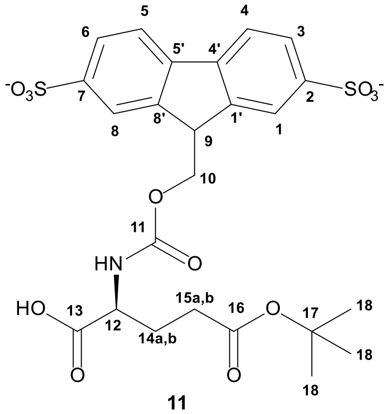

11

Table S11: Smoc-Gly-OH **12** in DMSO-d6 (^{1}H NMR at 500 MHz, ^{13}C NMR at 126 MHz).

Position	^{1}H	#H	Multi-plicity	Coupling constant	^{13}C
1 and 8	7.91	2	s	-	122.05
2 and 7	-	-	-	-	147.04
3 and 6	7.69	2	d	7.9	125.36
4 and 5	7.85	2	d	7.9	119.56
4' and 5'	-	-	-	-	140.59
1' and 8'	-	-	-	-	144.11
9	4.25	1	t	5.7	46.97
10	4.43	2	d	5.8	65.37
11	-	-	-	-	156.52
NH	7.49	1	br	-	-
12	3.61	2	s	-	42.15
13	-	-	-	-	171.39

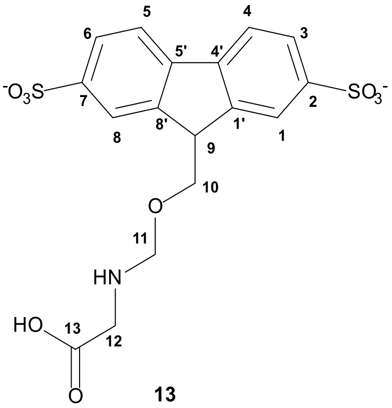

13

Table S12: Smoc-L-His-OH **13** in DMSO-d6 (^{1}H NMR at 500 MHz, ^{13}C NMR at 126 MHz).

Position	^{1}H	#H	Multi-plicity	Coupling constant	^{13}C
1 and 8	7.84; 7.92	2	s	-	121.9; 122.0
2 and 7	-	-	-	-	146.86
3 and 6	7.71	2	d	7.9	125.36
4 and 5	7.85	2	d	7.9	119.65
4' and 5'	-	-	-	-	140.57; 140.66
1' and 8'	-	-	-	-	143.60; 144.34
9	4.23	1	m	-	46.69
10a	4.33	1	dd	10.8; 5.3	65.12
10b	4.41	1	dd	10.8; 6.8	
11	-	-	-	-	155.78
NH	7.66	1	d	8.8	-
12	4.23	1	m	-	52.73
13	-	-	-	-	171.97
14	2.94	1	dd	15.0; 7.9	26.03
14b	3.13	1	dd	15.0; 4.4	
15	-	-	-	-	129.28
16	7.27	1	s	-	117.38
17	8.92	1	s	-	133.71

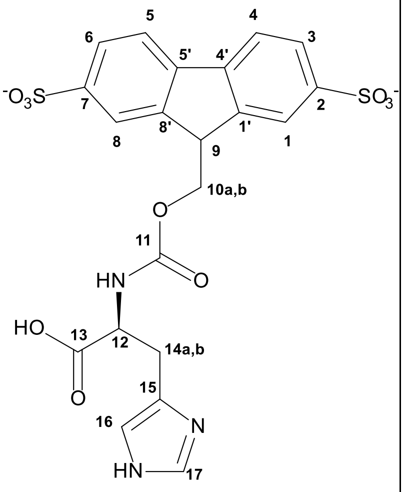

13

Table S13: Smoc-L-His(Trt)-OH **14** in DMSO-d$_6$ (^{1}H NMR at 500 MHz, ^{13}C NMR at 126 MHz).

Position	^{1}H	#H	Multi-plicity	Coupling constant	^{13}C
1 and 8	7.89; 7.99	2	s	-	122.3
2 and 7	-	-	-	-	147.65
3 and 6	7.69	2	d	7.7	125.6
4 and 5	7.84	2	d	7.7	119.1
4' and 5'	-	-	-	-	140.8
1' and 8'	-	-	-	-	143.76; 144.0
9	4.19	1	t	6.3	46.9
10a	3.94	1	m	-	65.23
10b	4.13	1	m	-	
11	-	-	-	-	156.0
NH	6.97	1	br	-	-
12	3.96	1	m	-	55.81
13	-	-	-	-	171.29
14	2.65	1	dd	14.9; 8.6	31.95
14b	2.99	1	dd	14.8; 4.6	
15	-	-	-	-	138.3
16	?*	-	-	-	118.8
17	7.22	-	br	-	137.5
18	-	-	-	-	74.38
19	-	-	-	-	142.4
20	7.06	6	m	-	128.9
21	7.37	6	m	-	128.5
22	7.06	3	m	-	127.5

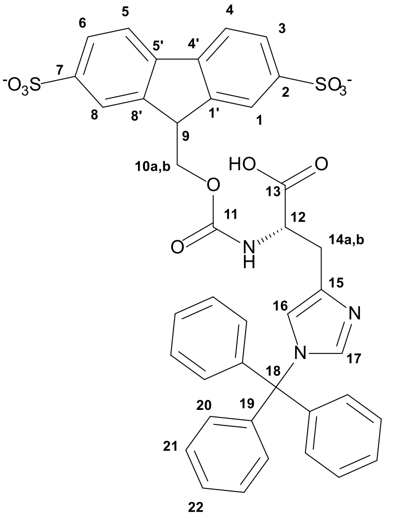

14

*: Signals have not been detected under the used conditions

Table S14: Smoc-L-Ile-OH **15** in DMSO-d$_6$ (^{1}H NMR at 500 MHz, ^{13}C NMR at 126 MHz).

Position	^{1}H	#H	Multi-plicity	Coupling constant	^{13}C
1 and 8	7.92	2	s	-	122.09
2 and 7	-	-	-	-	147.15; 147.17
3 and 6	7.68	2	dd	7.9; 1.2	125.31
4 and 5	7.83	2	d	7.9	119.44
4' and 5'	-	-	-	-	140.48
1' and 8'	-	-	-	-	144.03; 144.16
9	4.23	1	t	5.8	47.11
10a	4.37	1	dd	10.9; 5.7	65.46
10b	4.86	1	dd	10.9; 5.9	
11	-	-	-	-	156.44
NH	7.46	1	d	8.1	-
12	3.87	1	t	6.5	58.68
13	-	-	-	-	173.06
14	1.75	1	m	-	35.72
15a	1.17	1	ddq	13.6; 8.9; 7.3	24.88
15b	1.39	1	ddq	13.6; 7.4; 4.5	
16	0.81	3	t	7.4	11.22
17	0.84	3	d	6.9	15.48

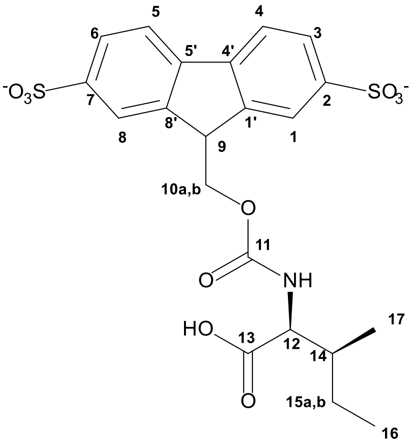

15

Table S15: Smoc-L-Leu-OH **16** in DMSO-d_6 (^{1}H NMR at 500 MHz, ^{13}C NMR at 126 MHz).

Position	^{1}H	#H	Multi-plicity	Coupling constant	^{13}C
1 and 8	7.90; 7.91	2	s	-	122.0
2 and 7	-	-	-	-	147.29
3 and 6	7.68	2	d	8	125.27
4 and 5	7.83	2	d	8	119.37
4' and 5'	-	-	-	-	140.42
1' and 8'	-	-	-	-	144.01; 144.09
9	4.22	1	t	5.6	47.06
10a	4.38	1	dd	11.0; 5.6	65.35
10b	4.46	1	dd	11.0; 5.6	
11	-	-	-	-	156.29
NH	7.51	1	d	7.9	-
12	3.93	1	dt	10.7; 5.4	52.28
13	-	-	-	-	174.17
14a	1.53	1	ddd	13.4; 10.2; 5.1	39.5
14b	1.41	1	ddd	13.5; 9.0; 4.9	
15	1.61	1	m	-	24.17
16	0.83	3	d	6.5	21.28
17	0.85	3	d	6.6	22.78

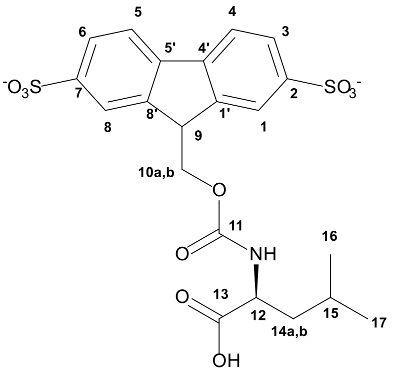

16

Table S16: Smoc-D-Leu-OH **17** in DMSO-d_6 (^{1}H NMR at 500 MHz, ^{13}C NMR at 126 MHz).

Position	^{1}H	#H	Multi-plicity	Coupling constant	^{13}C
1 and 8	7.92; 7.93	2	s	-	122.07
2 and 7	-	-	-	-	146.96; 146.99
3 and 6	7.69	2	dd	8; 1.5	125.36
4 and 5	7.85	2	d	8.0	119.56
4' and 5'	-	-	-	-	140.61
1' and 8'	-	-	-	-	144.14; 144.23
9	4.23	1	t	5.6	47.12
10a	4.39	1	dd	11.0; 5.6	65.35
10b	4.47	1	dd	11.0; 5.7	
11	-	-	-	-	156.33
NH	7.51	1	br	-	-
12	3.92	1	dd	9.7; 4.8	52.32
13	-	-	-	-	174.23
14a	1.52	1	ddd	13.7; 10.1; 5.1	39.56
14b	1.41	1	ddd	13.7; 9.0; 4.9	
15	1.61	1	m	-	24.23
16	0.82	3	d	6.6	21.33
17	0.85	3	d	6.6	22.83

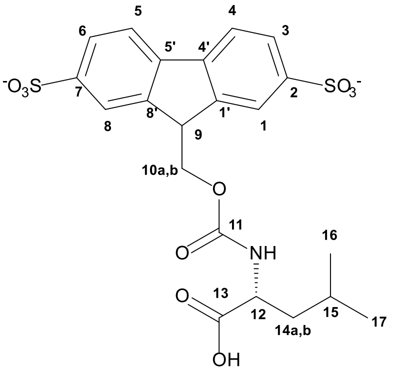

17

Table S17: Smoc-L-Lys(Boc)-OH **18** in DMSO-d6 (^{1}H NMR at 500 MHz, ^{13}C NMR at 126 MHz).

Position	^{1}H	#H	Multi-plicity	Coupling constant	^{13}C	
1 and 8	7.88; 7.93	2	s	-	122.26; 122.38	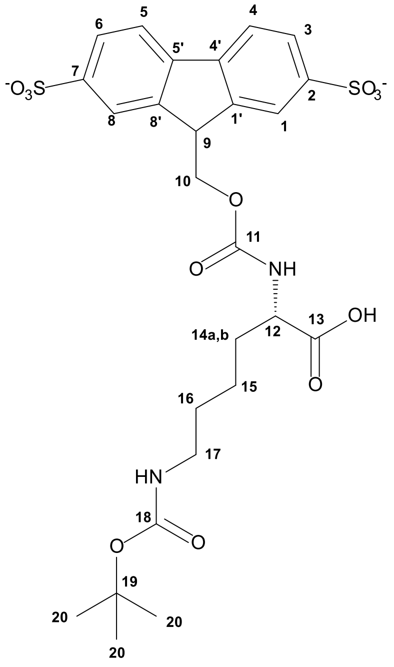 18
2 and 7	-	-	-	-	147.24; 147.31	
3 and 6	7.68	2	dd	7.9; 1.4	125.22	
4 and 5	7.82	2	d	7.9	119.33	
4' and 5'	-	-	-	-	140.3	
1' and 8'	-	-	-	-	143.92; 144.08	
9	4.24	1	t	-	46.75	
10	4.23	2	m	-	65.3	
11	-		-	-	155.45	
NH	6.56	1	br	-	-	
12	3.61	1	q	6.1	55.58	
13	-	-	-	-	173.58	
14a	1.54	1	m	-	32.44	
14b	1.64	1	dq	10.3; 5.4		
15	1.22	2	m	-	22.53	
16	1.33	2	m	-	29.6	
17	2.86	2	dt	7.4; 6.9	40.03	
NH	6.69	1	br	-	-	
18	-	-	-	-	155.24	
19	-	-	-	-	77.14	
20	1.33	9	s	-	28.24	

Table S18: Smoc-L-Met-OH **19** in DMSO-d6 (^{1}H NMR at 500 MHz, ^{13}C NMR at 126 MHz).

Position	^{1}H	#H	Multi-plicity	Coupling constant	^{13}C	
1 and 8	7.91; 7.92	2	s	-	122.1	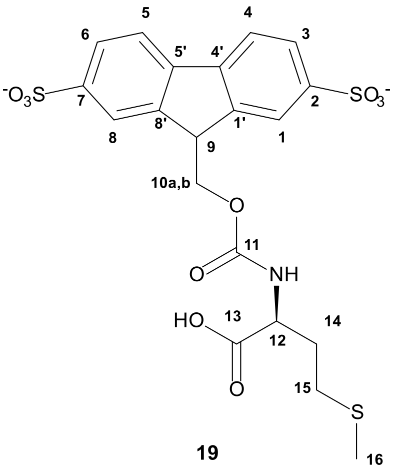
2 and 7	-	-	-	-	147.08	
3 and 6	7.69	2	dd	7.9; 1.2	125.36	
4 and 5	7.84	2	d	7.9	119.53	
4' and 5'	-	-	-	-	140.56	
1' and 8'	-	-	-	-	144.02; 144.18	
9	4.24	1	t	5.7	47.06	
10a	4.38	1	dd	10.9; 5.6	65.33	
10b	4.47	1	dd	10.9; 5.9		
11	-	-	-	-	156.33	
NH	7.6	1	d	7.6	-	
12	4.03	1	dt	7.7; 5.6	52.93	
13	-	-	-	-	173.5	
14	1.87	2	m	-	30.31	
15	2.47	2	m	-	29.83	
16	2.01	3	s	-	14.53	

Table S19: Smoc-L-Phe-OH **20** in DMSO-d6 (^{1}H NMR at 500 MHz, ^{13}C NMR at 126 MHz).

Position	^{1}H	#H	Multi-plicity	Coupling constant	^{13}C
1 and 8	7.9	2	s	-	122.02; 122.08
2 and 7	-	-	-	-	147.2
3 and 6	7.69	2	dd	7.9; 1.2	125.31
4 and 5	7.83	2	d	7.9	119.43
4' and 5'	-	-	-	-	140.44
1' and 8'	-	-	-	-	143.99; 144.05
9	4.16	1	t	5.5	47.05
10a	4.33	1	dd	11.0; 5.8	65.37
10b	4.38	1	dd	11.0; 5.7	
11	-	-	-	-	156.05
NH	7.61	1	d	8.1	-
12	4.12	1	m	-	55.65
13	-	-	-	-	173.02
14a	2.88	1	dd	13.8; 9.5	36.43
14b	2.99	1	dd	13.8; 5.3	
15	-	-	-	-	137.81
16	7.22	2	d	7.4	129.06
17	7.26	2	t	7.5	128.19
18	7.15	1	t	7.2	126.22

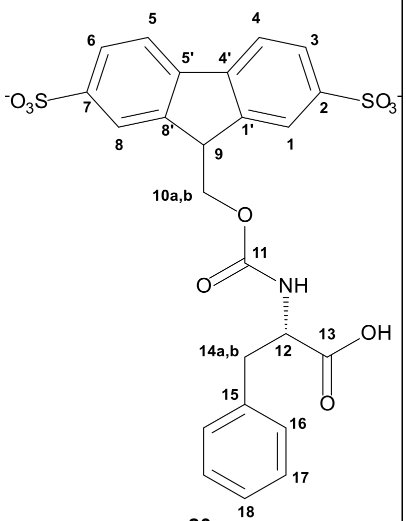

20

Table S20: Smoc-L-Pro-OH **21** in DMSO-d6 (^{1}H NMR at 500 MHz, ^{13}C NMR at 126 MHz).

Position	^{1}H	#H	Multi-plicity	Coupling constant	^{13}C
1 and 8	7.91; 7.92	2	d	1.4	122.42
2 and 7	-	-	-	-	147.17
3 and 6	7.68	2	dd	7.9; 1.4	125.3
4 and 5	7.84	2	d	7.9	119.49
4' and 5'	-	-	-	-	140.43
1' and 8'	-	-	-	-	143.66; 143.90
9	4.29	1	t	5.6	46.47
10	4.2-4.3	2	m	-	66.28
11	-	-	-	-	153.84
12	4.17	1	dd	8.6; 3.2	58.85
13	-	-	-	-	173.5
14a	1.85	1	m	-	29.34
14b	2.21	1	m	-	
15	1.85	2	m	-	23.9
16	3.41	2	m	-	46.01

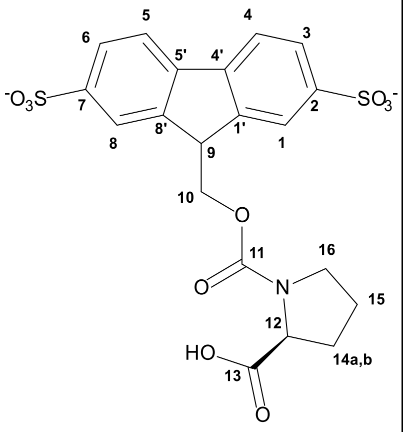

21

Table S21: Smoc-L-Ser-OH **22** in DMSO-d6 (^{1}H NMR at 500 MHz, ^{13}C NMR at 126 MHz).

Position	^{1}H	#H	Multi-plicity	Coupling constant	^{13}C
1 and 8	7.90; 7.92	2	s	-	122.0; 122.07
2 and 7	-	-	-	-	147.1
3 and 6	7.69	2	dd	7.9; 1.5	125.32
4 and 5	7.84	2	d	7.9	119.53
4' and 5'	-	-	-	-	140.54
1' and 8'	-	-	-	-	143.92; 144.20
9	4.25	1	t	5.7	46.96
10a	4.41	1	dd	11.0; 5.6	65.29
10b	4.47	1	dd	11.0; 6.0	
11	-	-	-	-	156.01
NH	7.14	1	br	-	-
12	4.0	1	t	5.2	56.72
13	-	-	-	-	171.94
14a	3.62	1	dd	11.3; 5.3	61.27
14b	3.65	1	dd	11.3; 4.2	
OH	4.01	1	br	-	-

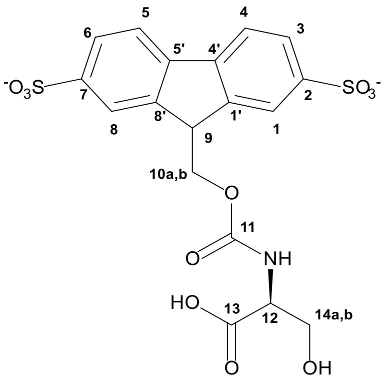

22

Table S22: Smoc-L-Ser(tBu)-OH **23** in DMSO-d6 (^{1}H NMR at 500 MHz, ^{13}C NMR at 126 MHz).

Position	^{1}H	#H	Multi-plicity	Coupling constant	^{13}C
1 and 8	7.92	2	s	-	122.08
2 and 7	-	-	-	-	147.43
3 and 6	7.69	2	d	7.9	125.29
4 and 5	7.82	2	d	7.9	119.32
4' and 5'	-	-	-	-	140.33
1' and 8'	-	-	-	-	143.87; 144.94
9	4.23	1	t	5.9	46.94
10a	4.37	1	dd	10.9; 5.9	65.6
10b	4.42	1	dd	10.9; 6.0	
11	-	-	-	-	156.01
NH	7.17	1	d	8.1	-
12	4.08	1	dt	8.0; 5.2	54.9
13	-	-	-	-	171.79
14a	3.54	1	dd	9.5; 4.9	61.23
14b	3.57	1	dd	9.5; 5.5	
15	-	-	-	-	72.78
16	1.1	9	s	-	27.14

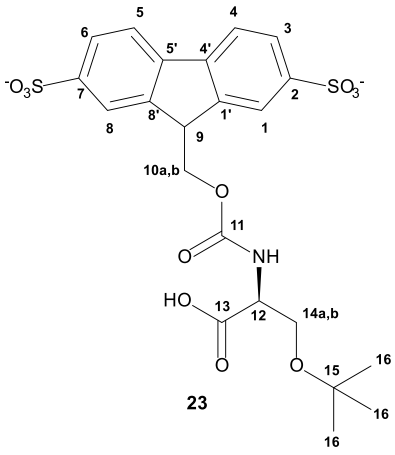

23

Table S23: Smoc-L-Thr-OH **24** in DMSO-d6 (^{1}H NMR at 500 MHz, ^{13}C NMR at 126 MHz).

Position	^{1}H	#H	Multi-plicity	Coupling constant	^{13}C
1 and 8	7.91; 7.92	2	s	-	122.07
2 and 7	-	-	-	-	147.22
3 and 6	7.69	2	d	7.9	125.3
4 and 5	7.83	2	d	7.9	119.43
4' and 5'	-	-	-	-	140.44
1' and 8'	-	-	-	-	143.86; 144.07
9	4.25	1	t	6.0	46.95
10a	4.39	1	dd	10.9; 5.7	65.52
10b	4.46	1	dd	10.9; 6.1	
11	-	-	-	-	156.38
NH	6.83	1	d	8.7	-
12	3.92	1	dd	8.1. 3;8	60.18
13	-	-	-	-	172.09
14	4.02	1	dq	6.5; 3.7	66.44
15	1.07	3	d	6.5	20.32

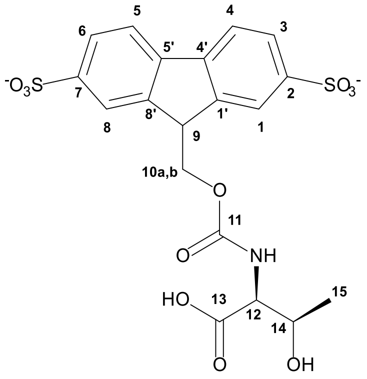

24

Table S24: Smoc-L-Thr(tBu)-OH **25** in DMSO-d6 (^{1}H NMR at 500 MHz, ^{13}C NMR at 126 MHz).

Position	^{1}H	#H	Multi-plicity	Coupling constant	^{13}C
1 and 8	7.90. 7.96	2	s	-	122.6. 122.7
2 and 7	-	-	-	-	147.19
3 and 6	7.69	2	dd	7.9; 15	125.55
4 and 5	7.83	2	d	7.9	119.56
4' and 5'	-	-	-	-	140.28
1' and 8'	-	-	-	-	143.95
9	4.23	1	m	-	46.54
10	4.23	2	m	-	65.58
11	-	-	-	-	156.1
NH	6.18	1	d	8.7	-
12	3.59	1	dd	8.7; 3.3	61.82
13	-	-	-	-	173
14	3.96	1	dq	6.2; 3.4	68.37
15	1.05	3	d	6.2	21.08
16	-	-	-	-	72.43
17	1.1	9	s	-	28.56

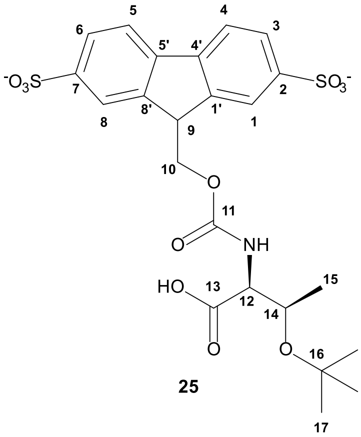

25

Table S25: Smoc-L-Trp-OH **26** in DMSO-d6 (^{1}H NMR at 500 MHz, ^{13}C NMR at 126 MHz).

Position	^{1}H	#H	Multi-plicity	Coupling constant	^{13}C
1 and 8	7.90; 7.93	2	s	-	122.02
2 and 7	-	-	-	-	147.00; 147.07
3 and 6	7.69; 7.70	2	dd	7.9. 1.5	125.32; 125.35
4 and 5	7.82; 7.83	2	d	7.9	119.54
4' and 5'	-	-	-	-	140.54; 140.57
1' and 8'	-	-	-	-	143.96; 144.25

9	4.21	1	t	5.8	46.9
10a	4.29	1	dd	10.8; 5.5	65.28
10b	4.4	1	dd	10.8; 6.1	
11	-	-	-	-	155.96
NH	7.53	1	d	8.2	-
12	4.12	1	dt	9; 4.5	45.18
13	-	-	-	-	173.47
14a	2.95	1	dd	14.6; 9.9	26.53
14b	3.16	1	dd	14.6; 4.5	
15	-	-	-	-	109.67
16	7.14	1	s	-	124.31
NH	10.87	1	s	-	-
17	-	-	-	-	135.84
18	7.29	1	d	7.5	111.33
19	7.03	1	t	7.5	120.64
20	6.96	1	t	7.4	118.18
21	7.48	1	d	7.8	117.81
22	-	-	-	-	127.04

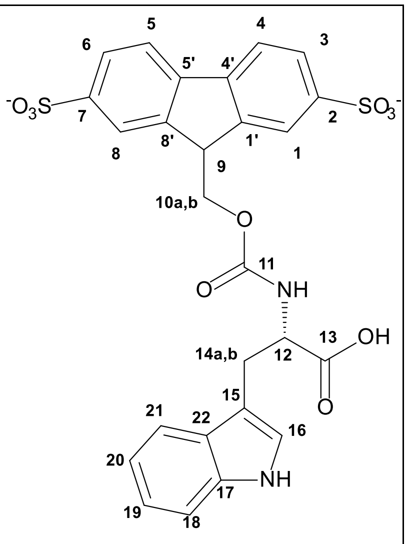

26

Table S26: Smoc-L-Trp(Boc)-OH **27** in DMSO-d6 (^{1}H NMR at 500 MHz, ^{13}C NMR at 126 MHz).

Position	^{1}H	#H	Multi-plicity	Coupling constant	^{13}C
1 and 8	7.87; 7.94	2	s	-	122.3; 122.5
2 and 7	-	-	-	-	147.77
3 and 6	7.69	2	d	7.9	125.2
4 and 5	7.82	2	d	7.9	119.3
4' and 5'	-	-	-	-	?*
1' and 8'	-	-	-	-	?*
9	4.18	1	m	-	46.27
10	4.18	2	m	-	65.18
11	-	-	-	-	?*
NH	6.72	1	d	6.8	-
12	3.93	1	q	6.1	55.19
13	-	-	-	-	173.45
14a	2.97	1	dd	14.6; 6.3	27.4
14b	3.16	1	dd	14.6; 5.4	
15	-	-	-	-	?*
16	7.43	1	s	-	?*
17	-	-	-	-	?*
18	8.0	1	d	8.2	114.3
19	7.26	1	t	7.7	123.9
20	7.21	1	t	7.4	122.2
21	7.64	1	d	7.7	119.3
22	-	-	-	-	?*
23	-	-	-	-	?*
24	-	-	-	-	83.24
25	1.61	9	s	-	27.69

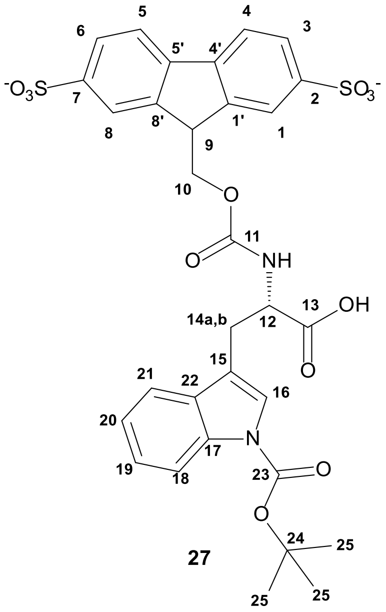

27

*: Signals have not been detected under the used conditions or assignment not possible.

Table S27: Smoc-L-Tyr-OH **28** in DMSO-d6 (^{1}H NMR at 500 MHz, ^{13}C NMR at 126 MHz).

Position	^{1}H	#H	Multi-plicity	Coupling constant	^{13}C
1 and 8	7.9	2	s	-	122.05
2 and 7	-	-	-	-	147.06; 147.13
3 and 6	7.69	2	d	8.0	125.34
4 and 5	7.84	2	d	8.0	119.5
4' and 5'	-	-	-	-	140.52
1' and 8'	-	-	-	-	144.1
9	4.18	1	t	5.7	47.06
10a	4.33	1	dd	11.0; 5.8	65.33
10b	4.39	1	dd	11.0; 5.7	
11	-	-	-	-	156.05
NH	7.5	1	d	8.0	-
12	4.03	1	q	7.1	56.0
13	-	-	-	-	173.19
14a	2.74	1	dd	13.9; 9.3	35.74
14b	2.86	1	dd	13.9; 5.4	
15	-	-	-	-	127.8
16	6.98	2	d	8.1	129.97
17	6.64	2	d	8.1	115.05
18	-	-	-	-	155.77
OH	8.15	1	s	-	-

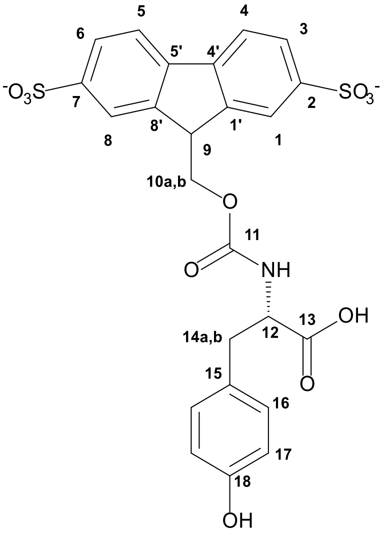

28

Table S28: Smoc-L-Tyr(tBu)-OH **29** in DMSO-d6 (^{1}H NMR at 500 MHz, ^{13}C NMR at 126 MHz).

Position	^{1}H	#H	Multi-plicity	Coupling constant	^{13}C
1 and 8	7.86; 7.92	2	s	-	122.71
2 and 7	-	-	-	-	147.78
3 and 6	7.67; 7.68	2	d	8.0	125.5
4 and 5	7.81	2	d	8.0	119.48
4' and 5'	-	-	-	-	140.24
1' and 8'	-	-	-	-	143.89
9	4.14	1	m	-	46.53
10	4.15	2	m	-	65.34
11	-	-	-	-	155.31
NH	6.51	1	d	7.2	-
12	3.83	1	q	6.6	57.47
13	-	-	-	-	173.08
14a	2.79	1	dd	13.6; 7.1	37.56
14b	3.0	1	dd	13.6; 4.9	
15	-	-	-	-	134.06
16	7.07	2	d	8.1	129.71
17	6.78	2	d	8.1	123.09
18	-	-	-	-	152.97
19	-	-	-	-	77.37
20	1.21	9	s	-	28.54

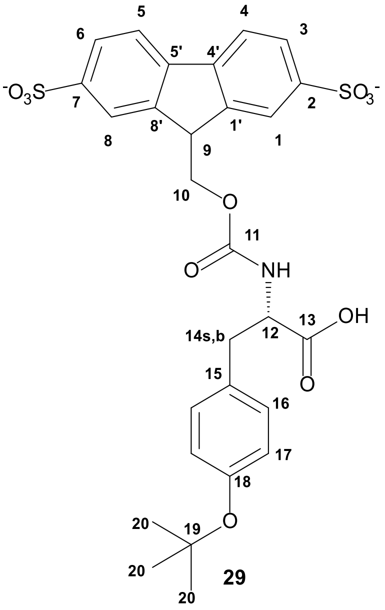

29

Table S29: Smoc-L-Val-OH **30** in DMSO-d_6 (^{1}H NMR at 500 MHz, ^{13}C NMR at 126 MHz).

Position	^{1}H	#H	Multi-plicity	Coupling constant	^{13}C
1 and 8	7.92; 7.93	2	s	-	122.1
2 and 7	-	-	-	-	147.13
3 and 6	7.68	2	dd	8.0; 1.3	125.31
4 and 5	7.83	2	d	8.0	119.45
4' and 5'	-	-	-	-	140.48
1' and 8'	-	-	-	-	144.02; 144.18
9	4.23	1	t	5.7	47.13
10a	4.38	1	dd	11.0; 5.7	65.45
10b	4.46	1	dd	11.0; 5.9	
11	-	-	-	-	156.53
NH	7.46	1	d	8.3	-
12	3.81	1	t	6.7	59.82
13	-	-	-	-	173.07
14a	2	1	oct	6.7	29.41
14b	2.86	1	dd	13.9; 5.4	
15	0.87	3	d	6.8	18.4
16	0.87	3	d	6.8	19.08

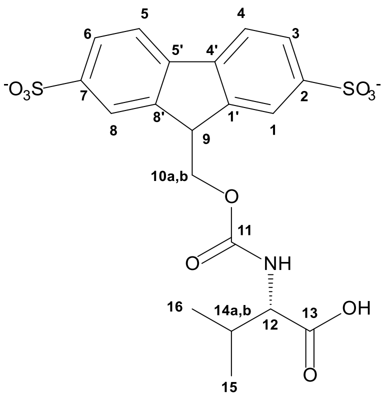

30

Table S30: Smoc-β-Ala-OH **31** in DMSO-d_6 (^{1}H NMR at 500 MHz, ^{13}C NMR at 126 MHz).

Position	^{1}H	#H	Multi-plicity	Coupling constant	^{13}C
1 and 8	7.87	2	s	-	121.92
2 and 7	-	-	-	-	147.28
3 and 6	7.67	2	d	8	125.21
4 and 5	7.81	2	d	7.9	119.34
4' and 5'	-	-	-	-	140.39
1' and 8'	-	-	-	-	144.01
9	4.22	1	t	5.7	47.0
10	4.42	2	d	5.7	64.86
11	-	-	-	-	155.99
NH	7.19	1	br	-	-
12	3.14	2	t	7.4	36.84
13	2.36	2	t	7.3	34.01
14	-	-	-	-	173.58

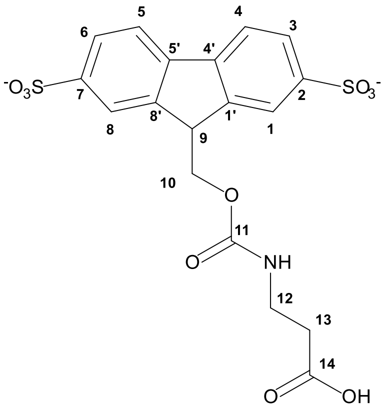

31

Table S31: Smoc-Aib-OH **32** in DMSO-d6 (^{1}H NMR at 500 MHz, ^{13}C NMR at 126 MHz).

Position	^{1}H	# H	Multi-plicity	Coupling constant	^{13}C
1 and 8	7.89	2	s	-	121.97
2 and 7	-	-	-	-	147.23
3 and 6	7.67	2	d	7.8	125.23
4 and 5	7.82	2	d	7.8	119.35
4' and 5'	-	-	-	-	140.43
1' and 8'	-	-	-	-	144.04
9	4.21	1	t	5.5	47.05
10	4.41	2	d	5.3	64.8
11	-	-	-	-	155.06
NH	7.44	1	br	-	-
12	-	-	-	-	55.2
13	-	-	-		175.6
14	1.29	6	s	-	24.97

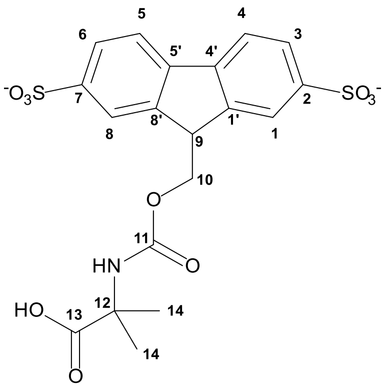

32

1.4. *N*α-Smoc deprotection

During the solid-phase peptide synthesis, the Nα-protecting group needs to be cleavable under mild conditions to prevent side chain deprotection, thus retaining the orthogonality. Smoc cleavage conditions were examined applying different aqueous bases or their solutions in polar solvents and the grade of deprotection after five minutes are summarized in **Table S32**.

Table S32: Summary of deprotection experiments.

Deprotection agent	Concentration	Solvent	Extent of deprotection after 5 min
**NaOH**	0.2 M	water	100%
**NaOH**	1 M	water<br>ethanol	100%<br>100%
**Ethanolamine**	10% (v/v)	water<br>ethanol	95%<br>75%
**Ethylenediamine**	10% (v/v)	water	100%
**Piperazine**	5% (w/v)	water	100%
**Ammonia**	10% (v/v)	water	100%
**Piperidine**	20% (v/v)	water	100%

After 5 min, the deprotection progress was monitored by HPLC. In general, two cleavage products (**33**, **34**) were observed for all used bases; in some cases, Smoc-base adducts were detected as well. Deprotection process is shown in **Figure S32**.

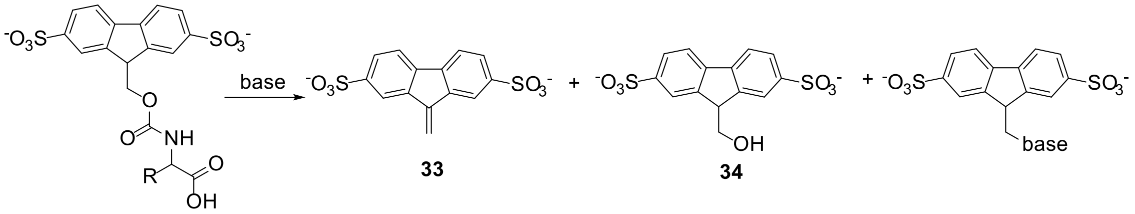

Figure S32: Deprotection of the Smoc protecting group leads to the products **33**, **34** as well as the possible base adduct.

1.5. Stability of Smoc-protected amino acids

After the conditions for the Smoc group deprotection have been thoroughly examined, the stability of N_{α}-Smoc amino acids during aqueous peptide synthesis was studied. Only with this knowledge Smoc could be considered as an N_{α}-protecting group. To that end, Smoc-Arg-OH **5**, Smoc-Ile-OH **15**, Smoc-Phe-OH **20**, Smoc-Pro-OH **21** and Smoc-Ser-OH **22** were dissolved in water with 3eq. $NaHCO_3$ and the resulting solutions were subjected to HPLC analysis after 7, 14 and 21 days; results are shown in **Figure S33**.

Interestingly, some of the impurities reduced over time, but the N_{α}-Smoc amino acids were stable under reaction conditions for 21 days, which is a sufficient time window. Stability might even be longer, but monitoring was stopped after 21 days.

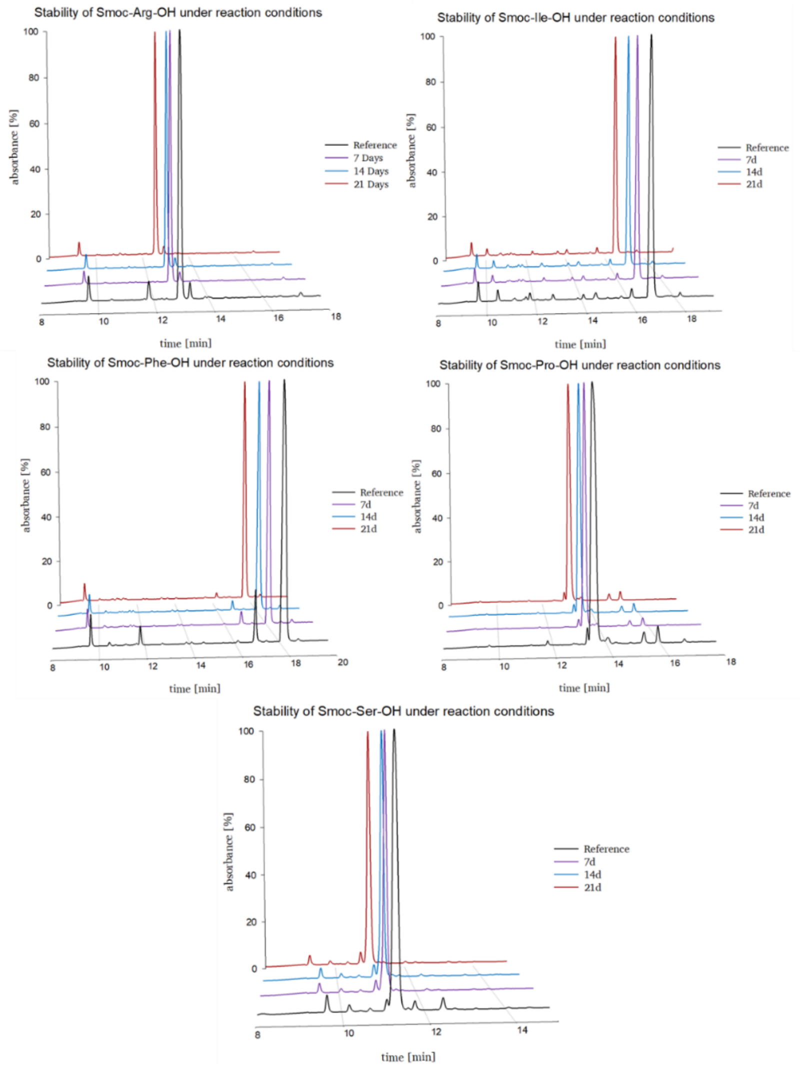

Figure S33: Stability studies of Smoc-Arg-OH **5** (top left), Smoc-Ile-OH **15** (top right), Smoc-Phe-OH **20** (middle left), Smoc-Pro-OH **21** (middle right) and Smoc-Ser-OH **22** (bottom). Reference is shown in black, 7 days in purple , 14 days in blue and 21 day in red. The shown area is reduced to the relevant range of the HPLC runs. Absorption values were normalised from 0 to 100%. HPLC traces were monitored at λ=220 nm with a gradient of 0to40 MeCN, see section **2.3** for details.

1.6. Coupling efficiency in water-based systems

Being one of the most fundamental chemical bonds in nature, an amide bond is the major constituent of protein backbone and a dominant motive in many natural products, biopolymers, and pharmaceuticals. To date, a vast repertoire of synthetic approaches towards amide bonds has been developed.

From the early years of peptide synthesis, it has been generally accepted that the formation of amide/peptide bonds implies anhydrous coupling conditions. Indeed, in the presence of water, the condensation equilibrium is shifted towards starting reagents and the active esters are hydrolysed (**Figure S34)**. However, the acidity of carboxylic acids in water is increased compared to the polar aprotic solvents commonly applied in peptide synthesis.[1] Therefore, the acidic proton of the carboxylic group is transferred to a water molecule, resulting in an increased reactivity of the carboxylate ion towards carbodiimides.[2]

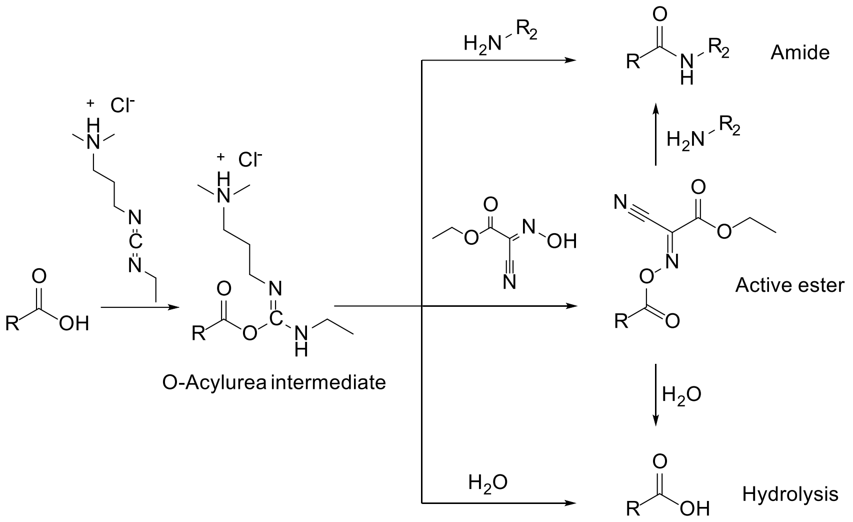

Figure S34: EDC-HCl **37** based activation of a carboxylic acid, followed by direct amide bond formation (top), formation of an Oxyma **39** active ester (middle) or hydrolysis of the activated species (bottom). (R1, R2: residues).

In order to investigate how active esters are formed under aqueous conditions and how these activated species participate in the amide bond formation, series of experiments with N_α-Smoc amino acids were performed.

Initially, different coupling reagents or active ester-forming compounds were evaluated in the synthesis of a dipeptide **36** at room temperature; the synthesis scheme is shown in **Figure S35**.

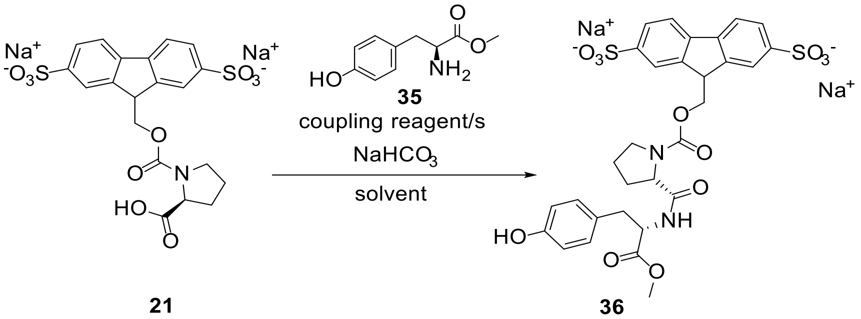

21 **36**

Figure S35: Synthesis of Smoc-L-Pro-L-Tyr-OMe **36**.

This experiment was performed using 1 eq. Smoc-Pro-OH **21**, 1.2 eq. H-Tyr-OMe **35**, 1eq. $NaHCO_3$ as base and 1.5 eq. of the coupling reagents **37**, **42**, **43** or **44** (**Table S33**) were dissolved in 3 mL of the following solvent systems: water, 30% aq. MeCN, 30% aq. ethanol, 30% aq. isopropanol, 10% aq. Me-THF, 30% Me-THF/water (biphasic) and 30% ethyl acetate/water (biphasic). After that time, the reaction progress was monitored by RP-HPLC.

Table S33: Summary of the used coupling additives and active ester-forming compounds.

Compound	Coupling reagents	Structure
**37**	1-ethyl-3-(3-dimethylaminopropyl)carbodiimide hydrochloride (EDC-HCl)	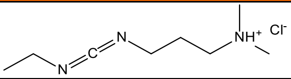
**38**	*N*-hydroxysuccinimide (NHS)	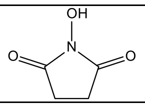

**39**	Oxyma	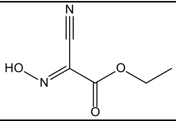
**40**	1-hydroxy-2-pyridone (HOPO)	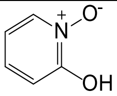
**41**	*N*-hydoxybicyclo[2.2.1]hept-5-ene-2,3-dicarboximide (HONB)	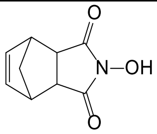
**42**	*N*-ethoxycarbonyl-2-ethoxy-1,2-dihydroquinoline(EEDQ)	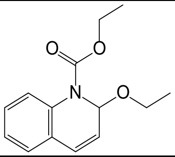
**43**	4-(4,6-dimethoxy-1,3,5-triazin-2-yl)-4-methyl-morpholinium chloride (DMT-MM)	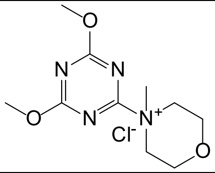
**44**	(1-Cyano-2-ethoxy-2-oxoethylidenaminooxy)-dimethylamino-morpholino-carbenium hexafluorophosphate (COMU)	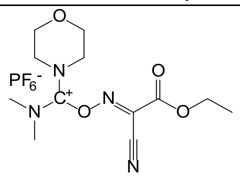

The HPLC analysis of aqueous reaction mixtures with the respective coupling reagents is shown in **Figure S36**.

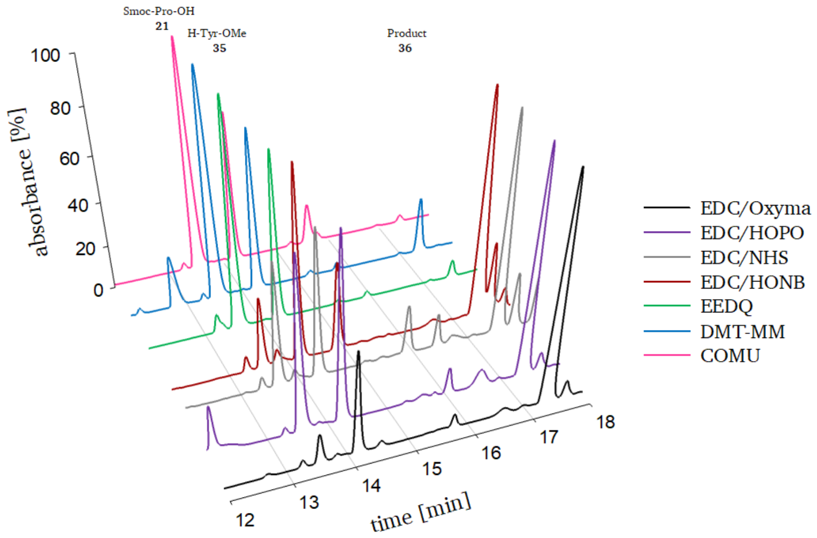

Figure S36: Synthesis of Smoc-L-Pro-L-Tyr-OMe **36** in water. The shown area is reduced to the relevant range of the HPLC runs. Absorption values were normalised from 0 to 100%. A direct comparison between the HPLC runs in terms of product formation is not possible. HPLC traces were monitored at λ=220 nm with a gradient of 0to40 MeCN, see section **2.3** for details.

The HPLC section shown is selected in such a way, that the resulting product and the starting reagents are visible. The graphs were normalized to 0-100%. A direct graphical comparison of the product peaks is not possible, since no additional normalization on the respective maxima was carried out. The product Smoc-L-Pro-L-Tyr-OMe **36** has retention time of ~17.2 minutes, the educt Smoc-Pro-OH **21** - 13.5, and H-Tyr-OMe **35** - 14.2 minutes. The complete HPLC data is shown in section **3.5.3**; the reference data of the educts **21** and **35**, coupling reagents **37-44** and product **36** is shown in section **3.5.1**.

The obtained data showed that the product formation in water was most efficient using EDC-HCl **37** activation with active ester reagents. Usage of EEDQ **42**, DMT-MM **43** and COMU **44** resulted only in a minor product formation after 25 minutes. Product formation *via* activation with DMT-MM and EEDQ increased with longer reaction times, but these are not suiTable Sfor peptide synthesis with repetitive coupling steps. In addition, EEDQ has a low solubility in water, which further reduces its efficacy. To summarize, Oxyma **39** appeared the most efficient additive, followed by HOPO **40**. HONB **41** is less active, this may be caused by its poorer solubility compared to the other additives. HPLC studies showed the formation of Smoc-Pro-NHS **45** and Smoc-Pro-HONB **46**. These esters are quite stable under the reaction conditions, both of them could be isolated by HPLC, structures are shown in **Figure S37**.

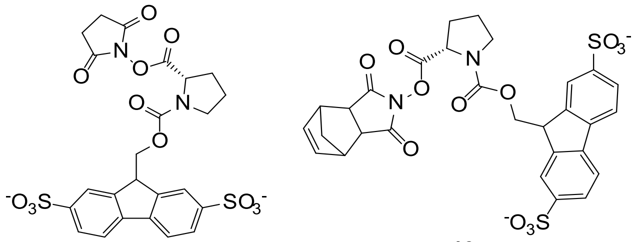

Figure S37: Structures of the isolated Smoc-Pro-NHS **45** and Smoc-Pro-HONB **46** ester.

NHS activation was found quite fast, but the formed NHS ester appeared rather stable and less reactive under aqueous conditions, which resulted in a reduced product formation. Interestingly, NHS ester activation was associated with a number of side products, which were not formed with the other additives. The following **Table S34** summarizes the efficiency of the used coupling reagents in water after 25 minutes. In general, the coupling efficiency increased along with reaction time (up to 45 minutes).

Table S34: Summary of coupling efficiency of the different reagents in water after 25 minutes.

Coupling reagent	Coupling efficiency*	Active ester side products
EDC-HCl **37**/Oxyma **39**	90.7%	Not observed
EDC-HCl **37**/HOPO **40**	58.3%	Not observed
EDC-HCl **37**/NHS **38**	54%	5.7% NHS ester
EDC-HCl **37**/HONB **41**	57.8%	28.9% HONB ester
EEDQ **42**	6.7%	not applicable
DMT-MM **43**	17.3%	not applicable
COMU **44**	3.2%	Not observed

* The coupling efficiency was calculated based on the area under the HPLC curve of the product, the starting materials and detected active ester derivatives.

The same experiment was performed in 30% aq. acetonitrile, to examine if the addition of small amounts of an organic solvent can influence amide bond formation. The obtained HPLC data for different coupling reagents after 25 minutes coupling in water are shown in **Figure S38**. The complete HPLC data are shown in section **3.5.4**; the reference data of the educts **21** and **35**, coupling reagents **37-44** and product **36** - in section **3.5.1**.

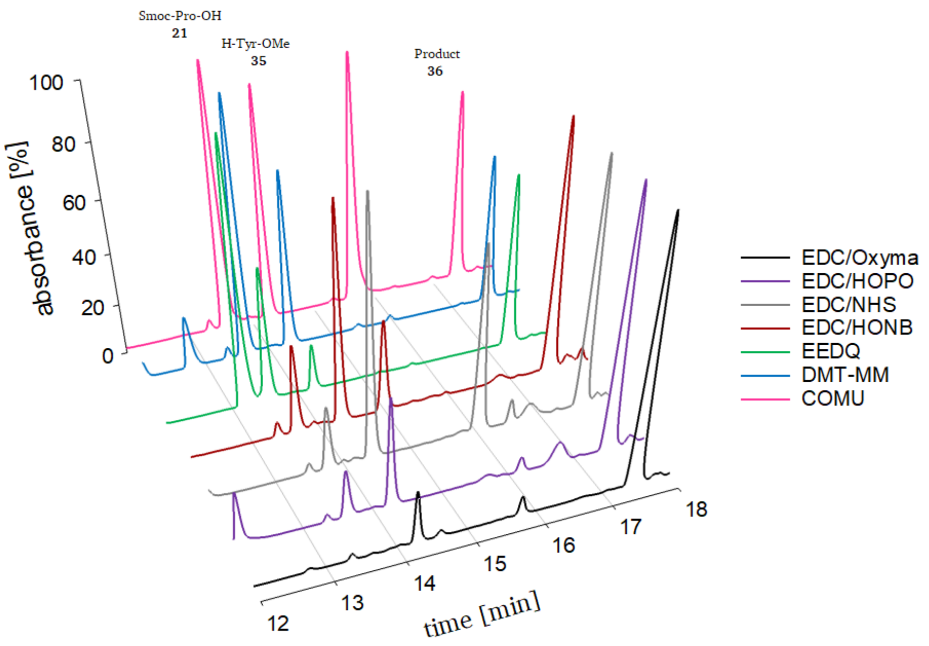

Figure S38: Synthesis of Smoc-L-Pro-L-Tyr-OMe **36** in 30% aq. MeCN. The shown area is reduced to the relevant range of the HPLC runs. Absorption values were normalised from 0 to 100%. A direct comparison between the HPLC runs in terms of product formation is not possible. HPLC traces were monitored at λ=220 nm with a gradient of 0to40 MeCN, see section **2.3** for details.

Generally, the efficiency of all used coupling reagents significantly increased in 30% aq. MeCN. As expected, the general trend was maintained, but a clear increase in the performance of HONB **40** and EEDQ **42** possessing low solubility in pure water was observed. The following **Table S35** summarizes the coupling efficiency of the used reagents in 30% aq. MeCN within 25 minutes. It is interesting to note that combination of EDC-HCl **37**/Oxyma **39** under these conditions allows an almost quantitative conversion. Starting Smoc-Pro-OH **21** was not observed in HPLC and LC-MS.

Table S35: Summary of coupling efficiency of the different reagents in 30% aq. MeCN after 25 minutes.

Coupling reagents	Coupling efficiency*	Active ester side products
EDC-HCl **37**/Oxyma **39**	99.8%	Not observed
EDC-HCl **37**/HOPO **40**	84.2%	Not observed
EDC-HCl **37**/NHS **38**	48.8%	38.9% NHS ester
EDC-HCl **37**/HONB **41**	47.6%	37.09% HONB ester
EEDQ **42**	40.5%	not applicable
DMT-MM **43**	37.3%	not applicable
COMU **44**	41.9%	Not observed

* The coupling efficiency was calculated based on the area under the HPLC curve of the product, the starting materials and detected active ester derivatives.

The same experimental setup was applied to other organic solvents. Results for 30% aq. ethanol are shown **Figure S39**. The complete HPLC data shown in section **3.5.6**; the reference data of the educts **21** and **35**, coupling reagents **37-44** and product **36** - in section **3.5.1**.

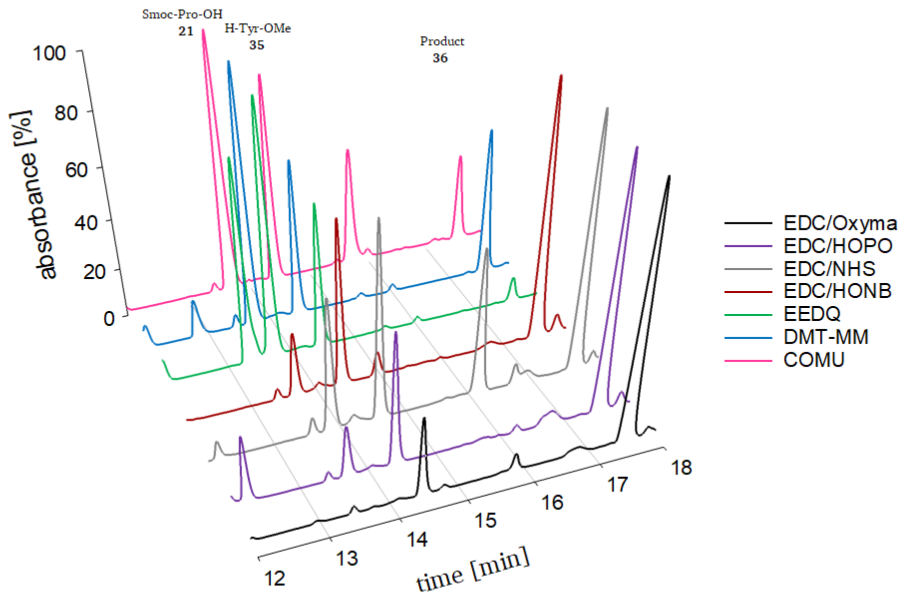

Figure S39: Synthesis of Smoc-L-Pro-L-Tyr-OMe **36** in 30% aq. ethanol. The shown area is reduced to the relevant range of the HPLC runs. Absorption values were normalised from 0 to 100%. A direct comparison between the HPLC runs in terms of product formation is not possible. HPLC traces were monitored at λ=220 nm with a gradient of 0to40 MeCN, see section **2.3** for details.

The direct comparison of ethanol and MeCN shows similar results in terms of EDC-HCl **37** and active ester-forming additives **38-41**, but a significant reduction in efficiency for the coupling reagents **42-44**. Compared to water, an increase in efficiency can be observed for all compounds. The following **Table S36** summarizes the coupling efficiency of the used reagents in 30% aq. ethanol after 25 minutes.

Table S36: Summary of coupling efficiency of the different reagents in 30% aq. ethanol after 25 minutes.

Coupling reagents	Coupling efficiency*	Active ester side products
EDC-HCl **37**/Oxyma **39**	99.0%	Not observed
EDC-HCl **37**/HOPO **40**	83.6%	Not observed
EDC-HCl **37**/NHS **38**	46.7%	28.9% NHS ester
EDC-HCl **37**/HONB **41**	50.5%	37.2% HONB ester
EEDQ **42**	8%	not applicable
DMT-MM **43**	36.5%	not applicable
COMU **44**	24.3%	Not observed

* The coupling efficiency was calculated based on the area under the HPLC curve of the product, the starting materials and detected active ester derivatives.

The formation of ethanol esters as side products was observed in LC-MS, but it was significantly reduced compared to pure alcohols. By extending the reaction time, the amount of ethanol ester increased, but was still less than 4% in our experiments and therefore in an acceptable range for the application in SPPS.

The similar experiments were performed with 30% aq. isopropanol; the results are presented in **Figure S40**. The complete HPLC data are shown in section **3.5.7**; the reference data of the educts **21** and **35**, coupling reagents **37-44** and product **36** shown in section **3.5.1**.

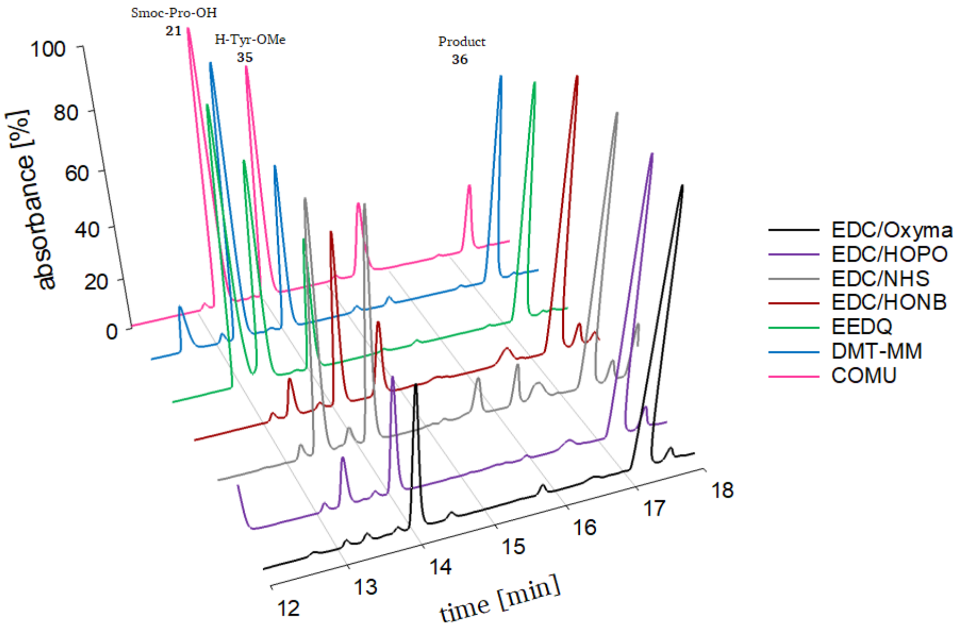

Figure S40: Synthesis of Smoc-L-Pro-L-Tyr-OMe **36** in 30% aq. isopropanol. The shown area is reduced to the relevant range of the HPLC runs. Absorption values were normalised from 0 to 100%. A direct comparison between the HPLC runs in terms of product formation is not possible. HPLC traces were monitored at λ=220 nm with a gradient of 0to40 MeCN, see section **2.3** for details.

Using 30% aq. isopropanol delivered the similar results as for ethanol-based system, with EEDQ coupling efficiency clearly improved due to the better solubility. The following **Table S37** summarizes the coupling efficiency of the used coupling reagents in 30% isopropanol after 25 minutes.

Table S37: Summary of coupling efficiency of the different reagents in 30% aq. isopropanol after 25 minutes.

Coupling reagents	Coupling efficiency*	Active ester side products
EDC-HCl **37**/Oxyma **39**	98.8%	Not observed
EDC-HCl **37**/HOPO **40**	83.6%	Not observed
EDC-HCl **37**/NHS **38**	47.8%	8.8% NHS ester
EDC-HCl **37**/HONB **41**	62.2%	30.4% HONB ester
EEDQ **42**	53.2%	not applicable
DMT-MM **43**	45.1%	not applicable
COMU **44**	25.6%	Not observed

* The coupling efficiency was calculated based on the area under the HPLC curve of the product, the starting materials and detected active ester derivatives.

An additional experiment was performed with 10% 2-methyltetrahydrofuran (Me-THF) as an additive using the same reaction setting as before. The obtained HPLC data are shown in **Figure S41**. The complete HPLC data shown in section **3.5.9**; the reference data of the educts **21** and **35**, coupling reagents **37-44** and product **36** - in section **3.5.1**.

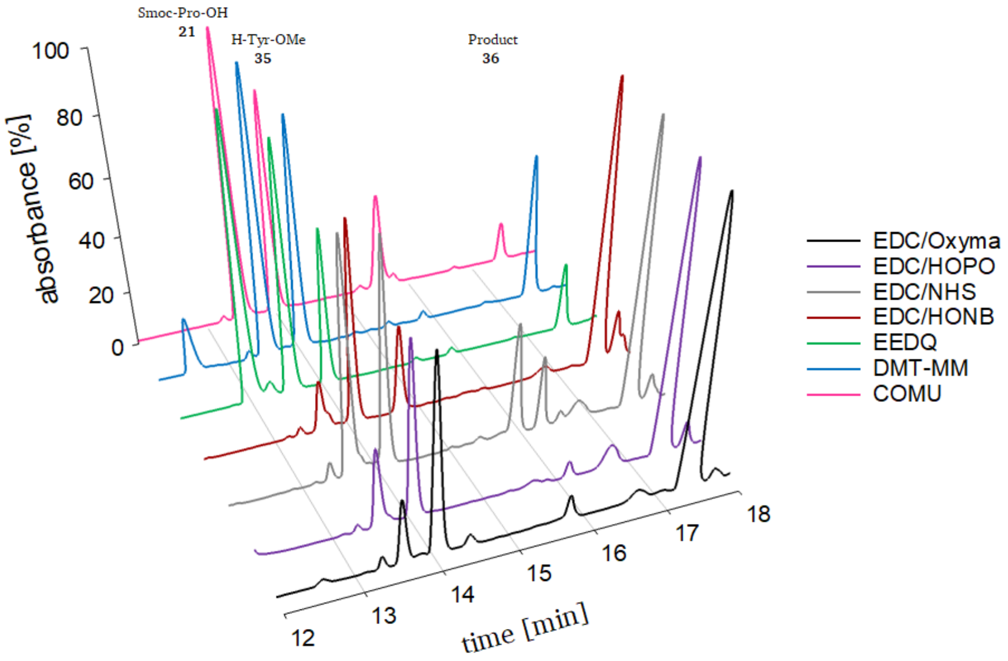

Figure S41: Synthesis of Smoc-L-Pro-L-Tyr-OMe **36** in 10% aq. Me-THF. The shown area is reduced to the relevant range of the HPLC runs. Absorption values were normalised from 0 to 100%. A direct comparison between the HPLC runs in terms of product formation is not possible. HPLC traces were monitored at λ=220 nm with a gradient of 0to40 MeCN, see section **2.3** for details.

Coupling efficiency in 10% aq. Me-THF was even lower than in pure water. Therefore, the usage of 10%-MeTHF was discarded for SPPS experiments. The following **Table S38** summarizes the coupling efficiency of the used coupling reagents in 10% Me-THF after 25 minutes.

Table S38: Summary of coupling efficiency of the different reagents in 10% aq. Me-THF after 25 minutes.

Coupling reagents	Coupling efficiency*	Active ester side products
EDC-HCl **37**/Oxyma **39**	88.7%	Not observed
EDC-HCl **37**/HOPO **40**	79.4%	Not observed
EDC-HCl **37**/NHS **38**	44.6%	17.4% NHS ester
EDC-HCl **37**/HONB **41**	61.5%	26.2% HONB ester
EEDQ **42**	34.9%	not applicable
DMT-MM **43**	39.9%	not applicable
COMU **44**	11.2%	Not observed

* The coupling efficiency was calculated based on the area under the HPLC curve of the product, the starting materials and detected active ester derivatives.

The same experiment was repeated with 30% aq. Me-THF. However, at this concentration phase separation occurred and the reaction was carried out in an emulsion. The obtained HPLC data are shown in **Figure S42**. The complete HPLC data shown in section **3.5.8**; the reference data of the educts **21** and **35**, coupling reagents **37-44** and product **36** - in section **3.5.1**.

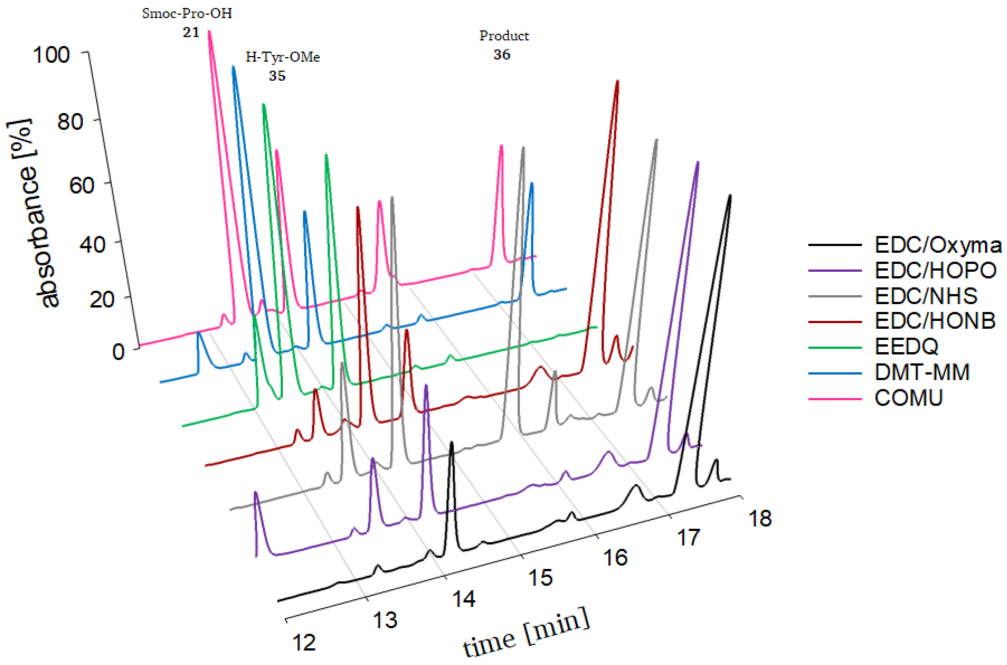

Figure S42: Synthesis of Smoc-L-Pro-L-Tyr-OMe **36** in 30% Me-THF in water (biphasic). The shown area is reduced to the relevant range of the HPLC runs. Absorption values were normalised from 0 to 100%. A direct comparison between the HPLC runs in terms of product formation is not possible. HPLC traces were monitored at λ=220 nm with a gradient of 0to40 MeCN, see section **2.3** for details.

Interestingly, in a biphasic system higher coupling yields were observed compared to those for the 10% aq. Me-THF. Though most of the coupling systems demonstrated increased efficiency, EDC-HCl **37**/HONB **41** and DMT-MM **43** showed decreased coupling yields, and EEDQ **42** did not lead to detectable product formation at all. Due to their hydrophobic properties, these reagents seem to be located exclusively in the organic layer, and phase-transfer catalyst is presumably required to make this system viable. The following **Table S39** summarizes the coupling efficiency of the used reagents in 30% aq. Me-THF after 25 minutes.

Table S39: Summary of coupling efficiency of the different reagents in 30% Me-THF/water (biphasic) after 25 minutes.

Coupling reagents	Coupling efficiency*	Active ester side products
EDC-HCl **37**/Oxyma **39**	99.8%	Not observed
EDC-HCl **37**/HOPO **40**	81.5%	Not observed
EDC-HCl **37**/NHS **38**	66.6%	23.3% NHS ester
EDC-HCl **37**/HONB **41**	57.4%	35.2% HONB ester
EEDQ **42**	0%	not applicable
DMT-MM **43**	28.6%	not applicable
COMU **44**	29.8%	Not observed

* The coupling efficiency was calculated based on the area under the HPLC curve of the product, the starting materials and detected active ester derivatives.

Based on the results of the biphasic Me-THF-water system, the similar experiment was performed with 30% ethyl acetate in water. As before, a phase separation was observed and the reaction was performed in an emulsion. The obtained HPLC data are shown in **Figure S43**. The complete HPLC data shown in section **3.5.5**; the reference data of the educts **21** and **35**, coupling reagents **37-44** and product **36** - in section **3.5.1**.

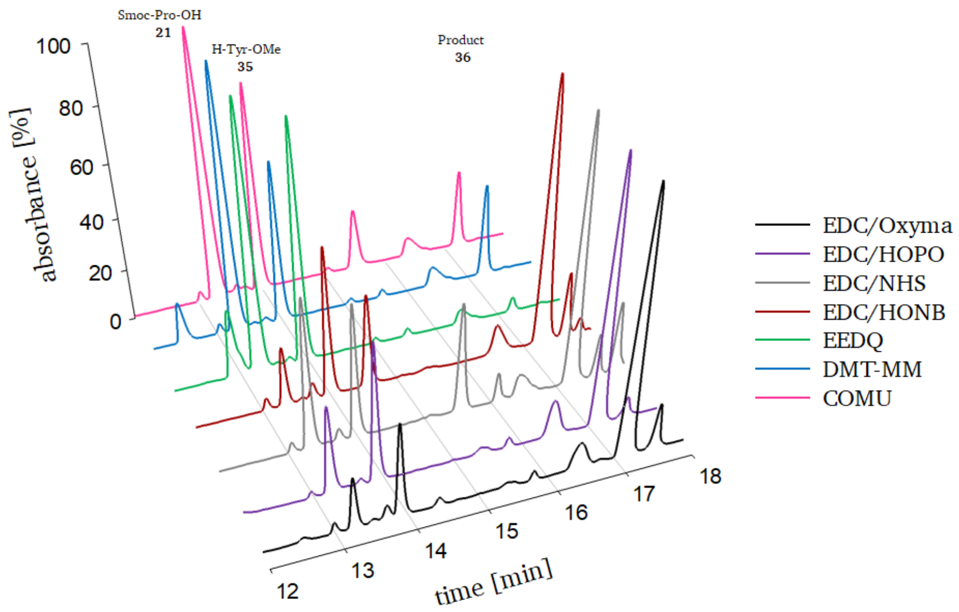

Figure S43: Synthesis of Smoc-L-Pro-L-Tyr-OMe **36** in 30% EtOAc in water (biphasic). The shown area is reduced to the relevant range of the HPLC runs. Absorption values were normalised from 0 to 100%. A direct comparison between the HPLC runs in terms of product formation is not possible. HPLC traces were monitored at λ=220 nm with a gradient of 0to40 MeCN, see section **2.3** for details.

The results are similar to those observed for 30% MeTHF in water, the product formation rates are slightly lower. Due to the difficult handling and the low efficiency of the biphasic systems in comparison to MeCN or alcohol-water mixtures, no SPPS reaction was carried out under these conditions. The following **Table S40** summarizes the efficiency of the used coupling reagents in 30% EtOAc after 25 minutes.

Table S40: Summary of coupling efficiency of the different reagents in 30% EtOAc/water (biphasic) after 25 minutes.

Coupling reagents	Coupling efficiency*	Active ester side products
EDC-HCl **37**/Oxyma **39**	87.3%	Not observed
EDC-HCl **37**/HOPO **40**	76.9%	Not observed
EDC-HCl **37**/NHS **38**	53%	21.5% NHS ester
EDC-HCl **37**/HONB **41**	64.9%	24.5% HONB ester
EEDQ **42**	4.4%	not applicable
DMT-MM **43**	29.9%	not applicable
COMU **44**	21.1%	Not observed

* The coupling efficiency was calculated based on the area under the HPLC curve of the product, the starting materials and detected active ester derivatives.

The coupling efficiency of the used coupling systems are summarized for easy comparability in the following **Table S41**. In summary, the EDC-HCl **37**/Oxyma **39** and EDC-HCl **37**/HOPO **40** mixtures have proven to be the most suitable candidates for water-based peptide synthesis applying sodium bicarbonate as a general base. Therefore, these reagents were used for SPPS test reactions with a short test peptide.

Table S41: Summary of coupling efficiency of the different reagents after 25 minutes.

Coupling reagents	Coupling efficiency*						
	water	30% MeCN	30% EtOH	30% *i*PrOH	10% Me-THF	30% Me-THF**	30% EtOAc**
EDC-HCl **37**/Oxyma **39**	90.7%	99.8%	99.0%	98.8%	88.7%	99.8%	87.3%
EDC-HCl **37**/HOPO **40**	58.3%	84.2%	83.6%	83.6%	79.4%	81.5%	76.9%
EDC-HCl **37**/NHS **38**	54%	48.8%	46.7%	47.8%	44.6%	66.6%	53%
EDC-HCl **37**/HONB **41**	57.8%	47.6%	50.5%	62.2%	61.5%	57.4%	64.9%
EEDQ **42**	6.7%	40.5%	8%	53.2%	34.9%	0%	4.4%
DMT-MM **43**	17.3%	37.3%	36.5%	45.1%	39.9%	28.6%	29.9%
COMU **44**	3.2%	41.9%	24.3%	25.6%	11.2%	29.8%	21.1%

* The coupling efficiency was calculated based on the area under the HPLC curve of the product, the starting materials and detected active ester derivatives; ** Biphasic

Following the previous experiments in solution, coupling experiments were carried out on the solid phase. The two most promising candidates, EDC-HCl **37**/Oxyma **39** and EDC-HCl **37**/HOPO **40**, were used for the synthesis of the tetrapeptide Smoc-**LAGV**-NH_2 **47**.

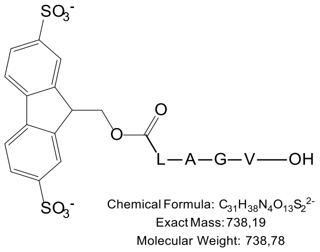

Figure S44: Smoc-LAGV-NH_2 **47** synthesis with EDC-HCl **37** and Oxyma **39**/HOPO **40**.

Peptide synthesis was carried out in a plastic syringe attached to a vacuum manifold to remove excess of reagents and solvents rapidly. The synthesis was carried out on 100 mg ChemMatrix H-Rink amide resin (loading capacity 0.4-0-6 mmol/g, 0.05 mmol; calculated on average loading). The resin was swollen for 2h in either water or 30% $MeCN_{(aq)}$ and loaded in double coupling (2×25min) using a solution of N_α-Smoc amino acid (3 eq.), EDC-HCl **37** (5.5 eq.), Oxyma **39** (3 eq.) or HOPO **40** (3 eq.) and $NaHCO_3$ (3 eq.) as base in 6 mL water or 30% $MeCN_{(aq)}$. Then the resin was washed with water twice followed by Smoc deprotection in 1M NaOH for 5 and 10 minutes with subsequent wash with water (2 times 5 mL). The coupling step of the next N_α-Smoc amino acid followed. After the chain assembly, the peptide was cleaved from the resin (without drying step) with TFA:H_2O (98:2) at ambient temperature for 2 h. The crude peptides **47** were precipitated in ice-cold diethyl ether, dried, dissolved in water and isolated by lyophilization. Analysis was performed by LC-MS and reverse-phase HPLC.

RP-HPLC (0 to 40% B): t_R = 15.3 min.
ESI-MS calc. for $C_{31}H_{40}N_4O_{13}S_2$ m/z: 740.80 meas. 738.18 $[M-2H]^-$, calc. 370.40 meas. 368.86 $[M-2H]^{2-}$.

The following **Table S42** summarizes the synthetic yields.

Table S42: Summary of the SPPS yields for Oxyma **39** and HOPO **40** based peptide synthesis in water or 30% aq. MeCN.

Coupling reagents	Solvent	Yield (calculated from average loading)
EDC-HCl **37**/Oxyma **39**	water	19mg (51%)
EDC-HCl **37**/Oxyma **39**	30% $MeCN_{(aq)}$	24mg (65%)
EDC-HCl **37**/HOPO **40**	water	18mg (49%)
EDC-HCl **37**/HOPO 40	30% $MeCN_{(aq)}$	17mg (46%)

The yields of the synthesized peptides showed that the syntheses with EDC-HCl **37**/Oxyma **39** is resulting in slightly higher yields compared to EDC-HCl **37**/HOPO **40**. This corresponds to the results obtained for solution-based peptide synthesis before. The usage of 30% organic solvents such as MeCN or alcohols showed a significant increase of the yield. Therefore, we assume that the addition of organic solvents seems to slow down the hydrolysis of the activated species and leads to a higher product formation. The surprisingly low performance of EDC-HCl **37**/HOPO **40** seems to be caused by a mistake during the synthesis or precipitation problem, because it contradicts all the other synthetic outcomes of the previous experiments.

The synthesis for all subsequent peptides was thus performed with EDC-HCl **37**/Oxyma **39** as standard coupling mixture.

1.7. Aqueous SPPS (ASPPS) of model peptides

1.7.1. General procedure for peptide synthesis

Peptide synthesis was carried out in a plastic syringe attached to a vacuum manifold to remove excess of reagents and solvents rapidly. The synthesis was conducted either on ChemMatrix H-Rink amide resin (loading capacity 0.4-0-6 mmol/g), HMPB-ChemMatrix resin (loading capacity 0.3-0.65 mmol/g) or on a commercially available preloaded HMPB resin; all resins were swollen for 2h in water. The first amino acid was attached by double coupling (2×25min) using a solution of N_{α}-Smoc amino acid (3 eq.), EDC-HCl **37** (5.5 eq.), Oxyma **39** (3 eq.) and $NaHCO_3$ (3 eq.) as base in 6 mL water or its mixture with organic solvent. The resin was washed with water twice followed by Smoc deprotection with either 1M NaOH, 25% aq. ethanolamine or 5-10% aq. piperazine for 5 and 10 minutes (depending on the used resin). Afterwards the resin was washed twice with water followed by coupling of the next amino acid until the desired peptide was completed. If not otherwise mentioned, the peptide was cleaved from the resin upon treatment with TFA:H_2O:TES (96:2:2) at RT for 1 h. The crude peptide was precipitated in ice-cold diethyl ether, dried, dissolved in water or aqueous acetonitrile and isolated by lyophilization. Analysis was performed by LC MS and reverse-phase HPLC.

The yields of synthesized peptides are generally consistent with those observed during the synthesis of chosen peptides in organic solvents. For example, for Leu-Enkephalin 72 % yield has been reported, and for vasopressin it was 30 % after cystine formation (to compare: we observed 71,8, respectively 39,7 %). [30, 31]

1.7.2. Synthesis of H-AGELS-NH_2 (Pentapeptide-31) 48

H—A-G-E-L-S—NH_2

Chemical Formula: $C_{19}H_{34}N_6O_8$
Exact Mass: 474,24
Molecular Weight: 474,52

Figure S45: H-AGELS-NH_2 (Pentapeptide-31) **48**.

Peptide synthesis was carried out according to the general procedure. Synthesis was carried out on 500 mg H-Rink amide-ChemMatrix (0.40-0.60 mmol/g, 0.25 mmol; calculated on average loading) in water. Preloading and coupling were performed according to the general procedure for 45 min at ambient temperature, deprotection of the Smoc protecting group was performed with 1M $NaOH_{(aq)}$. The crude peptide **48** was precipitated in ice-cold diethyl ether, dried, dissolved in water and isolated by lyophilization. Analysis was performed by LC MS and reverse-phase HPLC.

Yield: 13.72 mg (57.71% calculated on average loading)

RP-HPLC (0 to 60% B): t_R = 11.18 min.
ESI-MS calc. for $C_{19}H_{34}N_6O_8$ m/z: 474.52 meas. 475.37 $[M+H]^+$.

1.7.3. Synthesis of H-GPQGPQ-OH (Hexapeptide-9) 49

H—G-P-Q-G-P-Q—OH

Chemical Formula: $C_{24}H_{38}N_8O_9$
Exact Mass: 582,28
Molecular Weight: 582,62

Figure S46: H-GPQGPQ-OH (Hexapeptide-9) **49**.

Peptide synthesis was carried out according to the general procedure. Synthesis was carried out on 200 mg preloaded H-Gln-HMPB-ChemMatrix resin (0.30-0.70 mmol/g, 0.09 mmol; calculated on average loading) in water. Coupling was performed according to the general procedure for 45 min at ambient temperature, deprotection of the Smoc protecting group was performed with 25% ethanolamine$_{(aq)}$. Smoc-glutamine **10** was used without side-chain protecting group. The crude peptide **49** was precipitated in ice-cold diethyl ether, dried, dissolved in water and isolated by lyophilization. Analysis was performed by LC MS and reverse-phase HPLC.

Yield: 9.22 mg (38.7% calculated on average loading)

RP-HPLC (0 to 60% B): t_R = 11.09 min.
ESI-MS calc. for $C_{24}H_{38}N_8O_9$ m/z: 582.62 meas. 581.37 $[M-H]^-$.

1.7.4. Synthesis of H-EEMQRR-NH2 (Hexapeptide 3) 50

H—E-E-M-Q-R-R—NH_2

Chemical Formula: $C_{32}H_{58}N_{14}O_{11}S$
Exact Mass: 846,41
Molecular Weight: 846,96

Figure S47: H-EEMQRR-NH2 (Hexapeptide 3) **50**.

Peptide synthesis was carried out according to the general procedure. Synthesis was carried out on 100 mg H-Rink amide-ChemMatrix (0.40-0.60 mmol/g, 0.05 mmol; calculated on average loading) in water. Preloading and coupling were performed according to the general procedure for 45 min at ambient temperature, deprotection of the Smoc protecting group was performed with 1M NaOH$_{(aq)}$. Smoc-glutamine **10** and Smoc-arginine **5** were used without side-chain protecting groups. The crude peptide **50** was precipitated in ice-cold diethyl ether, dried, dissolved in water and isolated by lyophilization. Analysis was performed by LC MS and reverse-phase HPLC.

Yield: 21.73 mg (51.31% calculated on average loading).

RP-HPLC (0 to 40% B): t_R = 9.24 min.
ESI-MS calc. for $C_{32}H_{58}N_{14}O_{11}S$ m/z: 846.96 meas. 847.60 $[M+H]^+$.

1.7.5. Synthesis of Ac-EEMQRR-NH2 (Acetyl-Hexapeptide 3) 51

Ac—E-E-M-Q-R-R—NH_2

Chemical Formula: $C_{34}H_{60}N_{14}O_{12}S$
Exact Mass: 888,42
Molecular Weight: 889,00

Figure S48: Ac-EEMQRR-NH_2 (Acetyl-Hexapeptide 3) **51**.

Peptide synthesis was carried out according to the general procedure. Synthesis was carried out on 100 mg H-Rink amide-ChemMatrix (0.40-0.60 mmol/g, 0.05 mmol; calculated on average loading) in water. Preloading and coupling were performed according to the general procedure for 45 min at ambient temperature, deprotection of the Smoc protecting group was performed with 1M NaOH$_{(aq)}$. Smoc-glutamine **10** and Smoc-arginine **5** were used without side-chain protecting groups. The crude peptide

51 was precipitated in ice-cold diethyl ether, dried, dissolved in water and isolated by lyophilization. Analysis was performed by LC MS and reverse-phase HPLC.

Yield: 18.74 mg (42.16% calculated on average loading).

RP-HPLC (0 to 60% B): t_R = 9.28 min.
ESI-MS calc. for $C_{34}H_{60}N_{14}O_{12}S$ m/z: 889.00 meas. 889.70 $[M+H]^+$; calc. 444.50 meas. 445.60 $[M+2H]^{2+}$**.**

1.7.6. Synthesis of Leu-Enkephalin amide 52

H—Y-G-G-F-L—NH_2

Chemical Formula: $C_{28}H_{38}N_6O_6$
Exact Mass: 554,29
Molecular Weight: 554,65

Figure S49: Leu-Enkephalin amide **52.**

Peptide synthesis was carried out according to the general procedure. Synthesis was carried out on 100 mg H-Rink amide-ChemMatrix (0.40-0.60 mmol/g, 0.05 mmol; calculated on average loading) in water. Preloading and coupling were performed according to the general procedure for 45 min at ambient temperature, deprotection of the Smoc protecting group was performed with 1M $NaOH_{(aq)}$. Smoc-tyrosine **28** was used without side-chain protecting group. The crude peptide **52** was precipitated in ice-cold diethyl ether, dried, dissolved in water and isolated by lyophilization. Analysis was performed by LC MS and reverse-phase HPLC.

Yield: 19.65mg (70.86% calculated on average loading).

RP-HPLC (0 to 40% B): t_R = 17.98 min.
ESI-MS calc. for $C_{28}H_{38}N_6O_6$ m/z: 554.65 meas. 555.47 $[M+H]^+$.

1.7.7. Synthesis of Met-Enkephalin: H-YGGFM-OH 53

H—Y-G-G-F-M—OH

Chemical Formula: $C_{27}H_{35}N_5O_7S$
Exact Mass: 573,23
Molecular Weight: 573,67

Figure S50: Met-Enkephalin **53**.

Peptide synthesis was carried out according to the general procedure. Synthesis was carried out on 50 mg preloaded H-Met-HMPB-ChemMatrix resin (0.30-0.70 mmol/g, 0.025 mmol; calculated on average loading) in a water MeCN mixture (70:30). Coupling was performed according to the general procedure for 45 min at ambient temperature, deprotection of the Smoc protecting group was performed with 25% $ethanolamine_{(aq)}$. Peptide **53** was cleaved from solid support using 20% HFIP (in DCM)[3] to prevent oxidation of the Met-residue for 120 min, solvent was removed by rotary evaporator, dissolved in water and isolated by lyophilization. Analysis was performed by LC MS and reverse-phase HPLC.

Yield: 9 mg (63% calculated on average loading)

RP-HPLC (0 to 60% B): t_R = 17.77 min.
ESI-MS calc. for $C_{27}H_{35}N_5O_7S$ m/z: 573.67 meas. 574.18 $[M+H]^+$.

1.7.8. Synthesis of Leu-Enkephalin: H-YGGFL-OH 54

H—Y-G-G-F-L—OH

Chemical Formula: $C_{28}H_{37}N_5O_7$
Exact Mass: 555,27
Molecular Weight: 555,63

Figure S51: Leu-Enkephalin **54**.

Peptide synthesis was carried out according to the general procedure. Synthesis was carried out on 50 mg preloaded H-Leu-HMPB-ChemMatrix resin (0.30-0.70 mmol/g, 0.025 mmol; calculated on average loading) in a water MeCN mixture (70:30). Coupling was performed according to the general procedure for 45 min at ambient temperature, deprotection of the Smoc protecting group was performed with 25% ethanolamine$_{(aq)}$. Smoc-tyrosine **28** was used without side-chain protecting group. Peptide **54** was cleaved from solid support using HFIP (0.1% HCl)[4] for 120 min, solvent was removed by rotary evaporator, dissolved in water and isolated by lyophilization. Analysis was performed by LC MS and reverse-phase HPLC.

Yield: 10.3 mg (71.82% calculated on average loading)

RP-HPLC (0 to 60% B): t_R = 19.46 min.
ESI-MS calc. for $C_{28}H_{37}N_5O_7$ m/z: 555.63 meas. 556.37 $[M+H]^+$.

1.7.9. Synthesis of Acyl-Carrier-Protein (ACP) 65-74 peptide: H-VQAAIDYING-OH 55

H—V-Q-A-A-I-D-Y-I-N-G—OH

Chemical Formula: $C_{47}H_{75}N_{13}O_{15}$
Exact Mass: 1061,55
Molecular Weight: 1062,19

Figure S52: Acyl-Carrier-Protein (ACP) 65-74: H-VQAAIDYING-OH **55.**

Peptide synthesis was carried out according to the general procedure. Synthesis was carried out on 100 mg preloaded H-Gly-HMPB-ChemMatrix resin (0.30-0.65 mmol/g, 0.045 mmol; calculated on average loading) in a water MeCN mixture (50:50). Coupling was performed according to the general procedure for 25 min at ambient temperature as double coupling step, deprotection of the Smoc protecting group was performed with 5% piperazine$_{(aq)}$. Smoc-tyrosine **28**, Smoc-asparagine **7** and Smoc-glutamine **10** were used without side-chain protecting groups. Peptide **55** was cleaved from solid support using HFIP with 0.1% HCl[4] for 120 min. The crude peptide **55** was precipitated in ice-cold diethyl ether, dried, dissolved in water and isolated by lyophilization. Analysis was performed by LC MS and reverse-phase HPLC.

Yield: 18 mg (36% calculated on average loading)

RP-HPLC (10 to 100% B): t_R = 11.66 min.
ESI-MS calc. for $C_{47}H_{75}$N13O_{15} m/z: 1062.19 meas. 1063.67 $[M+H]^+$, calc. 531.5 meas. 532.55 $[M+2H]^{2+}$.

1.7.10. Synthesis of Acyl-Carrier-Protein (ACP) 65-74: H-VQAAIDYING-NH_2 56

H—V-Q-A-A-I-D-Y-I-N-G—NH_2

Chemical Formula: $C_{47}H_{74}N_{12}O_{16}$
Exact Mass: 1062,53
Molecular Weight: 1063,18

Figure S53: Acyl-Carrier-Protein (ACP) 65-74: H-VQAAIDYING-NH_2 **56**.

Peptide synthesis was carried out according to the general procedure. Synthesis was carried out on 100 mg H-Rink amide-ChemMatrix (0.40-0.60 mmol/g, 0.05 mmol; calculated on average loading) in a water MeCN mixture (50:50). Preloading and coupling were performed according to the general procedure for 25 min at ambient temperature as double coupling step, deprotection of the Smoc protecting group was performed with 5% piperazine$_{(aq)}$. Smoc-tyrosine **28**, Smoc-asparagine **7** and Smoc-glutamine **10** were used without side-chain protecting groups. Peptide **56** was cleaved from solid support using TFA:H_2O:TES (95:2.5:2.5) for 60 min. The crude peptide **56** was precipitated in ice-cold diethyl ether, dried, dissolved in water and isolated by lyophilization. Analysis was performed by LC MS and reverse-phase HPLC.

Yield: 23 mg (46% calculated on average loading)

RP-HPLC (10 to 100% B): t_R = 11.57 min.
ESI-MS calc. for $C_{47}H_{74}N_{12}O_{16}$ m/z: 1063.18 meas. 1062.67 $[M+H]^+$, calc. 531.59 meas. 532.05 $[M+2H]^{2+}$.

1.7.11. Synthesis of H-GPRP-OH 57

H—G-P-R-P—OH
Chemical Formula: $C_{18}H_{31}N_7O_5$
Exact Mass: 425,24
Molecular Weight: 425,49

Figure S54: H-GPRP-OH **57**.

Peptide synthesis was carried out according to the general procedure. Synthesis was carried out on 100 mg preloaded H-Gly-HMPB-ChemMatrix resin (0.30-0.65 mmol/g, 0.045 mmol; calculated on average loading) in water. Coupling was performed according to the general procedure for 45 min at ambient temperature, deprotection of the Smoc protecting group was performed with 25% ethanolamine$_{(aq)}$. Peptide **57** was cleaved from solid support using HFIP with 0.1% HCl[4] for 120 min. The crude peptide **57** was precipitated in ice-cold diethyl ether, dried, dissolved in water or aqueous acetonitrile and isolated by lyophilization. Analysis was performed by LC MS and reverse-phase HPLC.

Yield: 8 mg (38% calculated on average loading)

RP-HPLC (0 to 40% B): t_R = 13.29 min.
ESI-MS calc. for $C_{18}H_{31}N_7O_8$ m/z: 425.49 meas. 426.40 $[M+H]^+$, calc. 212.75 meas. 214.00 $[M+2H]^{2+}$.

1.7.12. Synthesis of Smoc-VVIA-NH_2 58

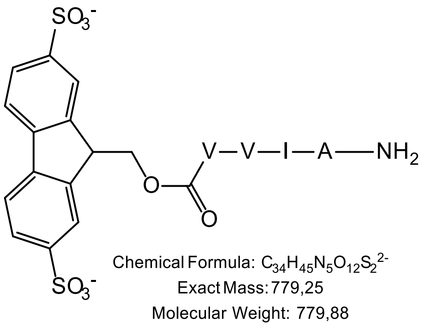

Figure S55: Smoc-VVIA amide **58**.

Peptide synthesis was carried out according to the general procedure. Synthesis was carried out on 100 mg H-Rink amide-ChemMatrix (0.40-0.60 mmol/g, 0.05 mmol; calculated on average loading) in water. Coupling was performed according to the general procedure for 45 min at ambient temperature, deprotection of the Smoc protecting group was performed with 1M NaOH$_{(aq)}$. The final N_α-Smoc

protecting group was left on the peptide for an easier HPLC detection, Smoc-peptide **58** was cleaved from solid support using TFA:H_2O (95:5). The crude Smoc-peptide **58** was precipitated in ice-cold diethyl ether, dried, dissolved in water and isolated by lyophilization. Analysis was performed by LC MS and reverse-phase HPLC.

Yield: 26 mg (67% calculated on average loading)

RP-HPLC (0 to 40% B): t_R = 16.45 min.
ESI-MS calc. for $C_{34}H_{47}N_5O_{12}S_2$ m/z: 781.89 meas. 780.40 $[M-H]^-$, calc. 390.20 meas. 390.00 $[M-2H]^{2-}$

1.7.13. Synthesis of Smoc-DIIW-OH 59

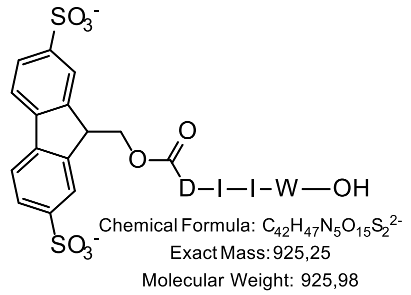

Figure S56: Smoc-DIIW-OH **59**.

Peptide synthesis was carried out according to the general procedure. Synthesis was carried out on 100 mg preloaded H-Trp-HMPB-ChemMatrix resin (0.30-0.65 mmol/g, 0.045 mmol; calculated on average loading) in water. Coupling was performed according to the general procedure for 45 min at ambient temperature, deprotection of the Smoc protecting group was performed with 1M $NaOH_{(aq)}$. The final N_α-Smoc protecting group was left on the peptide for an easier HPLC detection, Smoc-peptide **59** was cleaved from solid support using HFIP with 0.1% HCl[4] for 120 min. The crude Smoc-peptide **59** was precipitated in ice-cold diethyl ether, dried, dissolved in water and isolated by lyophilization. Analysis was performed by LC MS and reverse-phase HPLC.

Yield: 22 mg (50% calculated on average loading)

RP-HPLC (0 to 40% B): t_R = 16.32 min.
ESI-MS calc. for $C_{42}H_{49}N_5O_{15}S_2$ m/z: 927.99 meas. 928.40 $[M-H]^-$, calc. 464.20 meas. 463.97 $[M-2H]^2$.

1.7.14. Synthesis of Smoc-E(OtBu)K(Boc)R(Pbf)S(tBu)C(Trt)-OH 60 as model for a fully protected peptide.

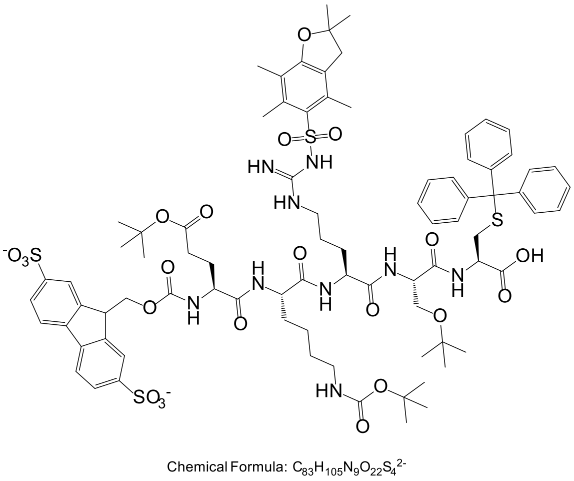

Figure S57: Structure of Smoc-E(OtBu)K(Boc)R(Pbf)S(tBu)C(Trt)-OH **60**.

Peptide synthesis was carried out according to the general procedure. Synthesis was carried out on 50 mg preloaded H-Cys(Trt)-HMPB-ChemMatrix resin (0.30-0.65 mmol/g, 0.045 mmol; calculated on average loading) in a water MeCN mixture (50:50). Preloading and coupling were performed according to the general procedure for 25 min at ambient temperature as double coupling step, deprotection of the Smoc protecting group was performed with 5% piperazine$_{(aq)}$. The fully protected Peptide **60** was cleaved from solid support using 20% HFIP (in DCM) for 120 min and solvent removed by rotary evaporator. The crude peptide **60** was dried, dissolved in aqueous acetonitrile and isolated by lyophilization. Analysis was performed by LC MS and reverse-phase HPLC.

Yield: 18 mg (44% calculated on average loading)

RP-HPLC (50 to 100% B): t_R = 18.83 min.
ESI-MS calc. for $C_{19}H_{19}NO_{10}S_2$ m/z: 855.53 meas. 854.30 $[M-2H]^{2-}$.

1.7.15. Synthesis of model peptides 61,62 for racemization tests

H—C-Y-E-I-S—NH$_2$
Chemical Formula: $C_{26}H_{40}N_6O_9S$
Exact Mass: 612,26
Molecular Weight: 612,70

H—A-N-K-P-G—NH$_2$
Chemical Formula: $C_{20}H_{36}N_8O_6$
Exact Mass: 484,28
Molecular Weight: 484,56

Figure S58: Model peptides H-CYEIS-NH_2 **61** and H-ANKPG-NH_2 **62** for racemization tests.

Peptide synthesis was carried out according to the general procedure. Synthesis was carried out on 100 mg H-Rink amide-ChemMatrix (0.40-0.60 mmol/g, 0.05 mmol; calculated on average loading) in a water MeCN mixture (70:30). Preloading and Coupling was performed according to the general procedure for 25 min at ambient temperature as double coupling step, deprotection of the Smoc protecting group was performed with 1M NaOH$_{(aq)}$. Smoc-asparagine **7** and Smoc-tyrosine **28** were used without side-chain protecting groups. Peptides **61**, **62** were cleaved from solid support using TFA:H_2O:TES (95:2.5:2.5) for 60 min. The crude peptides **61, 62** were precipitated in ice-cold diethyl ether, dried, dissolved in water or aqueous acetonitrile and isolated by lyophilization. Analysis was performed by LC MS and reverse-phase HPLC.

H-CYEIS-NH_2 61
Yield: 15mg (48.99% calculated on average loading)
RP-HPLC (50 to 100% B): t_R = 19.21 min.
ESI-MS calc. for $C_{26}H_{40}N_6O_9S$ m/z: 612.70 meas. 613.28 $[M+H]^+$.

H-ANKPG-NH_2 62
Yield: 13mg (53.66% calculated on average loading)
RP-HPLC (0 to 40% B): t_R = 9.94 min.
ESI-MS calc. for $C_{20}H_{36}N_8O_9$ m/z: 484.56 meas. 485.37 $[M+H]^+$.

Samples **61**, **62** as well as Smoc-Asn-OH **7** were sent to *C.A.T. GmbH & Co Chromatographie und Analysentechnik KG* (Tübingen, Germany) for analysis of amino acid racemization.

1.7.16. Synthesis of Pal-GHK-OH 63

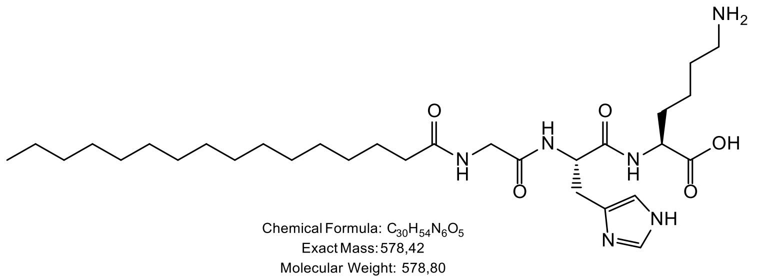

Figure S59: Structure of Pal-GHK-OH **63**.

Peptide synthesis was carried out according to the general procedure. Synthesis was carried out on 100 mg preloaded H-Lys(Boc)-HMPB-ChemMatrix resin (0.30-0.60 mmol/g, 0.045 mmol; calculated on average loading) in a water ethanol mixture (80:20). Coupling was performed according to the general procedure for 30 min at ambient temperature, deprotection of the Smoc protecting group was performed with 1M $NaOH_{(aq)}$. Palmitoylation was performed with palmitic acid (3 eq.), EDC-HCl **37** (5.5 eq.), Oxyma **39** (3 eq.) and $NaHCO_3$ (3 eq.) in ethanol water (70:30) at pH 7.2 for 45 min. Washing was performed twice with ethanol water (70:30) at pH 7.2 and twice with diethyl ether. Peptide **63** was cleaved from solid support using 95% $TFA_{(aq)}$ for 60 min, solvent was removed by lyophilisation.

Yield: 14.12 mg (54.23% calculated on average loading)

RP-HPLC (10 to 60% B): t_R = 20.34 min.
ESI-MS calc. for $C_{30}H_{54}N_6O_5$ m/z: 578.80 meas. 579.57 $[M+H]^+$.

1.7.17. Synthesis of Pal-GQPR-OH 64

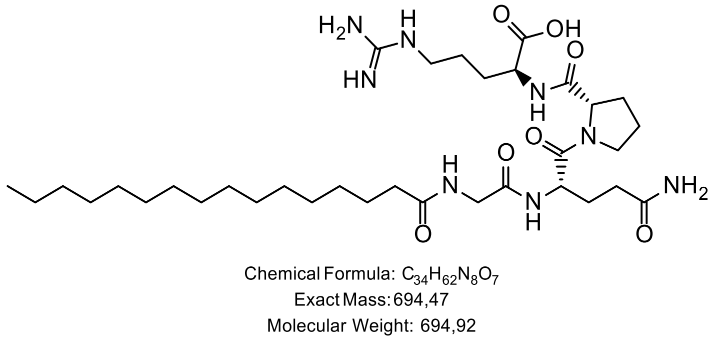

Figure S60: Structure of Pal-GQPR-OH **64**.

Peptide synthesis was carried out according to the general procedure. Synthesis was carried out on 100 mg preloaded H-Arg-HMPB-ChemMatrix resin (0.30-0.60 mmol/g, 0.045 mmol; calculated on average loading) in a water ethanol mixture (80:20). Coupling was performed according to the general procedure for 30 min at ambient temperature, deprotection of the Smoc protecting group was performed with 1M $NaOH_{(aq)}$. Smoc-Glutamine **10** was used without side-chain protecting group. Palmitoylation was performed with palmitic acid (3 eq.), EDC-HCl **37** (5.5 eq.), Oxyma **39** (3 eq.) and $NaHCO_3$ (3 eq.) in ethanol water (70:30) at pH 7.2 for 45 min. Washing was performed twice with ethanol water (70:30) at pH 7.2 and twice with diethyl ether. Peptide **64** was cleaved from solid support using 95% $TFA_{(aq)}$ for 60 min, solvent was removed by lyophilisation.

Yield: 18.68 mg (59.74% calculated on average loading)

RP-HPLC (0 to 60% B): t_R = 20.26 min.
ESI-MS calc. for $C_{34}H_{62}N_8O_7$ m/z: 694.92 meas. 695.66 $[M+H]^+$.

1.7.18. Synthesis of H-GPRPA-NH_2 Vialox (Pentapeptide-3) 65

H—G-P-R-P-A—NH_2

Chemical Formula: $C_{21}H_{37}N_9O_5$

Exact Mass: 495,29

Molecular Weight: 495,59

Figure S61: H-GPRPA-NH_2 Vialox (Pentapeptide-3) **65**.

Peptide synthesis was carried out according to the general procedure. Synthesis was carried out on 100 mg H-Rink amide-ChemMatrix (0.40-0.60 mmol/g, 0.05 mmol; calculated on maximal loading) in a water MeCN mixture (70:30). Preloading and coupling were performed according to the general procedure for 25 min at ambient temperature, deprotection of the Smoc protecting group was performed with 1M $NaOH_{(aq)}$. Peptide **65** was cleaved from solid support using TFA:H_2O:TES (95:2.5:2.5) for 60 min. The crude peptide **65** was precipitated in ice-cold diethyl ether, dried, dissolved in water and isolated by lyophilization. Analysis was performed by LC MS and reverse-phase HPLC.

Yield: 12 mg (48.43% calculated on average loading)

RP-HPLC (0 to 40% B): t_R = 11.35 min.
ESI-MS calc. for $C_{21}H_{37}N_9O_5$ m/z: 495.59 meas. 496.37 $[M+H]^+$, calc. 247.80 meas. 248.95 $[M+2H]^{2+}$.

1.7.19. Synthesis of Oxytocin 66

H—C-Y-I-Q-N-C-P-L-G—NH_2

Chemical Formula: $C_{43}H_{66}N_{12}O_{12}S_2$

Exact Mass: 1006,44

Molecular Weight: 1007,19

Figure S62: Oxytocin **66**.

Peptide synthesis was carried out according to the general procedure. Synthesis was carried out on 100 mg H-Rink amide-ChemMatrix (0.40-0.60 mmol/g, 0.05 mmol; calculated on average loading) in a water MeCN mixture (70:30). Preloading and coupling were performed according to the general procedure for 25 min at ambient temperature as double coupling step, deprotection of the Smoc protecting group was performed with 1M $NaOH_{(aq)}$. Smoc-tyrosine **28**, Smoc-asparagine **7** and Smoc-glutamine **10** were used without side-chain protecting groups. Peptide **66** was cleaved from solid support using TFA:TES:H_2O:DTT (90:2.5:2.5:5) for 60 min. The crude peptide **66** was precipitated in ice-cold diethyl ether, dried, dissolved in water and isolated by lyophilization. The disulfide bond was closed in 0.1M NH_4HCO_3 buffer pH 8 in a concentration 1 mg/mL for 3 days at room temperature. Analysis was performed by LC MS and reverse-phase HPLC.

Yield: 18 mg (35.75% calculated on average loading)

RP-HPLC (10 to 100% B): t_R = 11.82 min.
ESI-MS calc. for $C_{43}H_{66}N_{12}O_{12}S_2$ m/z: 1007.19 meas. 1007.67 $[M+H]^+$, calc. 503.60 meas. 504.55 $[M+2H]^{2+}$.

1.7.20. Synthesis of Vasopressin (peptide hormone) 67

H—C-Y-F-Q-N-C-P-R-G—NH_2

Chemical Formula: $C_{46}H_{65}N_{15}O_{12}S_2$

Exact Mass: 1083,44

Molecular Weight: 1084,24

Figure S63: Vasopressin **67**.

Peptide synthesis was carried out according to the general procedure. Synthesis was carried out on 100 mg H-Rink amide-ChemMatrix (0.40-0.60 mmol/g, 0.05 mmol; calculated on average loading) in a water MeCN mixture (70:30). Preloading and coupling were performed according to the general procedure for 25 min at ambient temperature as double coupling step, deprotection of the Smoc protecting group was performed with 1M $NaOH_{(aq)}$. Smoc-tyrosine **28**, Smoc-asparagine **7** and Smoc-glutamine **10** were used without side-chain protecting groups. Peptide **67** was cleaved from solid support using TFA:TES:H_2O:DTT (90:2.5:2.5:5) for 60 min. The crude peptide **67** was precipitated in ice-cold diethyl ether, dried, dissolved in water and isolated by lyophilization. The disulfide bond was closed in 0.1M NH_4HCO_3 buffer pH 8 in a concentration 1 mg/mL for 3 days at room temperature. Analysis was performed by LC MS and reverse-phase HPLC.

Yield: 20 mg (39.71% calculated on average loading)

RP-HPLC (10 to 100% B): t_R = 10.47 min.
ESI-MS calc. for $C_{46}H_{65}N_{15}O_{12}S_2$ m/z: 1084.24 meas. 1084.57 $[M+H]^+$, calc. 542.12 meas. 543.05 $[M+2H]^{2+}$.

1.7.21. Synthesis of heptaarginine cell-penetrating peptide 68

H—R-R-R-R-R-R-R—NH_2

Chemical Formula: $C_{42}H_{87}N_{29}O_7$

Exact Mass: 1109,73

Molecular Weight: 1110,35

Figure S64: Heptaarginine cell-penetrating peptide **68**.

Peptide synthesis was carried out according to the general procedure. Synthesis was carried out on 100 mg H-Rink amide-ChemMatrix (0.40-0.60 mmol/g, 0.05 mmol; calculated on average loading) in a water MeCN mixture (70:30). Preloading and coupling were performed according to the general procedure for 45 min at ambient temperature, deprotection of the Smoc protecting group was performed with 1M $NaOH_{(aq)}$. Smoc-arginine **5** was used without side-chain protecting groups. Peptide **68** was cleaved from solid support using TFA:H_2O: (95:5) for 60 min. The crude peptide **68** was precipitated in ice-cold diethyl ether, dried, dissolved in water or aqueous acetonitrile and isolated by lyophilization. Analysis was performed by LC MS and reverse-phase HPLC.

Yield: 23 mg (41.43% calculated on average loading)

RP-HPLC (0 to 40% B): t_R = 13.86 min. (Smoc-heptaarginine for easier detection)
ESI-MS calc. for $C_{42}H_{87}N_{29}O_7$ m/z: 1110.35 meas. 1113.27 $[M+H]^+$, calc. 555.18 meas. 557.15 $[M+2H]^{2+}$.

1.7.22. Synthesis of H-Y(D-A)GFL-OH Leuphasyl 69

H—Y–a–G–F–L—OH

Chemical Formula: $C_{29}H_{39}N_5O_7$

Exact Mass: 569,28

Molecular Weight: 569,66

Figure S65: Leuphasyl **69.**

Peptide synthesis was carried out according to the general procedure. Synthesis was carried out on 100 mg preloaded H-Leu-HMPB-ChemMatrix resin (0.30-0.60 mmol/g, 0.045 mmol; calculated on average loading) in a water MeCN mixture (80:20). Coupling was performed according to the general procedure for 45 min at ambient temperature, deprotection of the Smoc protecting group was performed with 1M NaOH(aq). Peptide **69** was cleaved from solid support using 1% TFA for 60 min. The crude peptide **69**

was precipitated in ice-cold diethyl ether, dried, dissolved in water or aqueous acetonitrile and isolated by lyophilization. Analysis was performed by LC MS and reverse-phase HPLC.

Yield: 17.3 mg (57.85% calculated on average loading)

RP-HPLC (0 to 60% B): t_R = 18.7 min.
ESI-MS calc. for $C_{29}H_{39}N_5O_7$ m/z: 569.66 meas. 570.27 $[M+H]^+$.

1.8. Purification by affinity chromatography

Preparative RP-HPLC is currently the industrial state-of-art method for peptide purification allowing one to remove impurities in the crude product. Productivity, yield, and solvent consumption have to be considered for each purification process. Unfortunately, these three factors influence each other.[28] A general trend in peptide synthesis is to optimize the chemical synthesis (upstream process) and then accept up to 50% loss during the HPLC isolation (downstream) due to severe purification problems and in order to keep solvent consumption in acceptable ranges.[28]

The purification system proposed by Merrifield and Bach and their 2-sulfo-Fmoc derivate[29] for product purification could of course be applied to the Smoc derivatives (**Figure S66**).

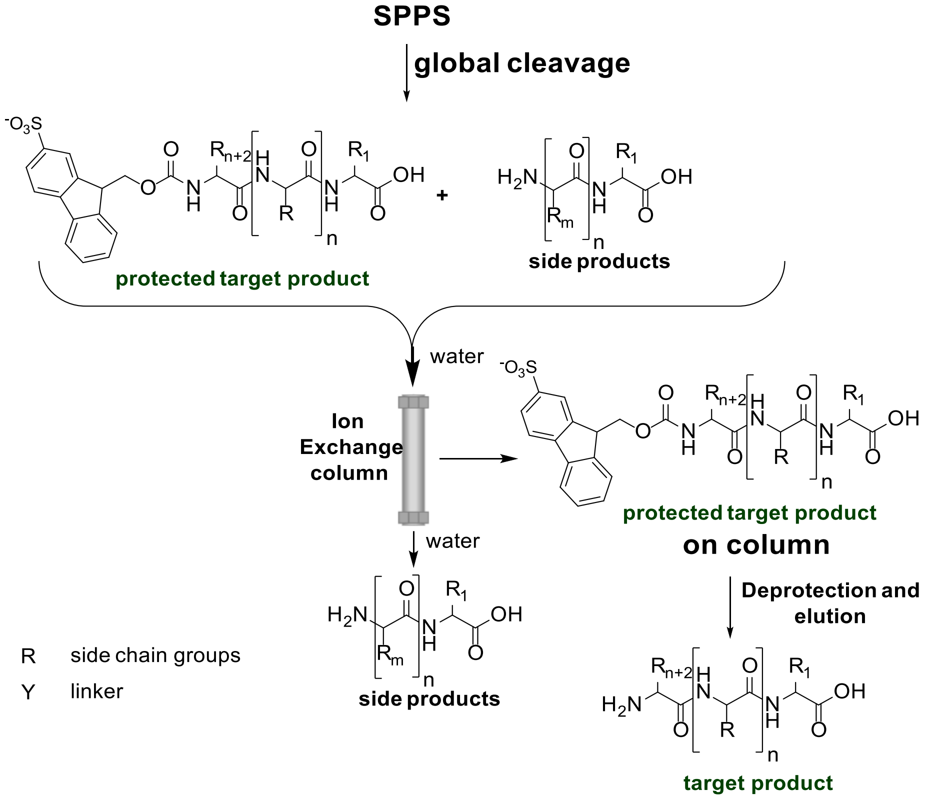

Figure S66: Purification method by Merrifield and Bach.[29] After global cleavage, mixture is put on an ion-exchange column and the protected target peptide stays on the column. This is followed by an on-column deprotection of the respective 2-sulfo-Fmoc derivate and the elution of the target peptide in the deprotection cocktail.

However, this strategy is quite labour intensive as an additional deprotection step after the purification is necessary. As a consequence, we aimed of modifying the purification concept for the ASPPS strategy. In order to simplify the peptide isolation/purification process, we developed the capping strategy based on *Sulfo-tags*. This allows a large number of by-products to be removed using IEC. The advantages of the IEC method are high capacity, low time effort, application of water as solvent and easy automation. Moreover, compared to HPLC purification, the IEC is quite inexpensive.

The capping method is based on Sulfo-carboxylic compounds, e.g. 4-sulfobenzoic acid **75** or sulfoacetic acid **76**. Structures are shown in **Figure S67**. After each coupling step, capping with **75**, **76** or similar compounds is performed to label all free amine residues. After cleavage from solid support, the target peptide, all labelled side products and the cleaved side chain protecting groups are in solution. This mixture is subjected to IEC. Only the

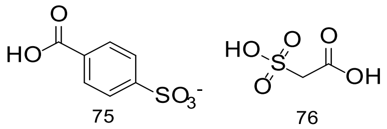

75 76

Figure S67: Structures of 4-sulfobenzoic acid **75** and sulfoacetic acid **76** as examples for capping reagents.

target peptide is able to run through, all labelled impurities stay on the column. The strategy is shown in **Figure S68**.

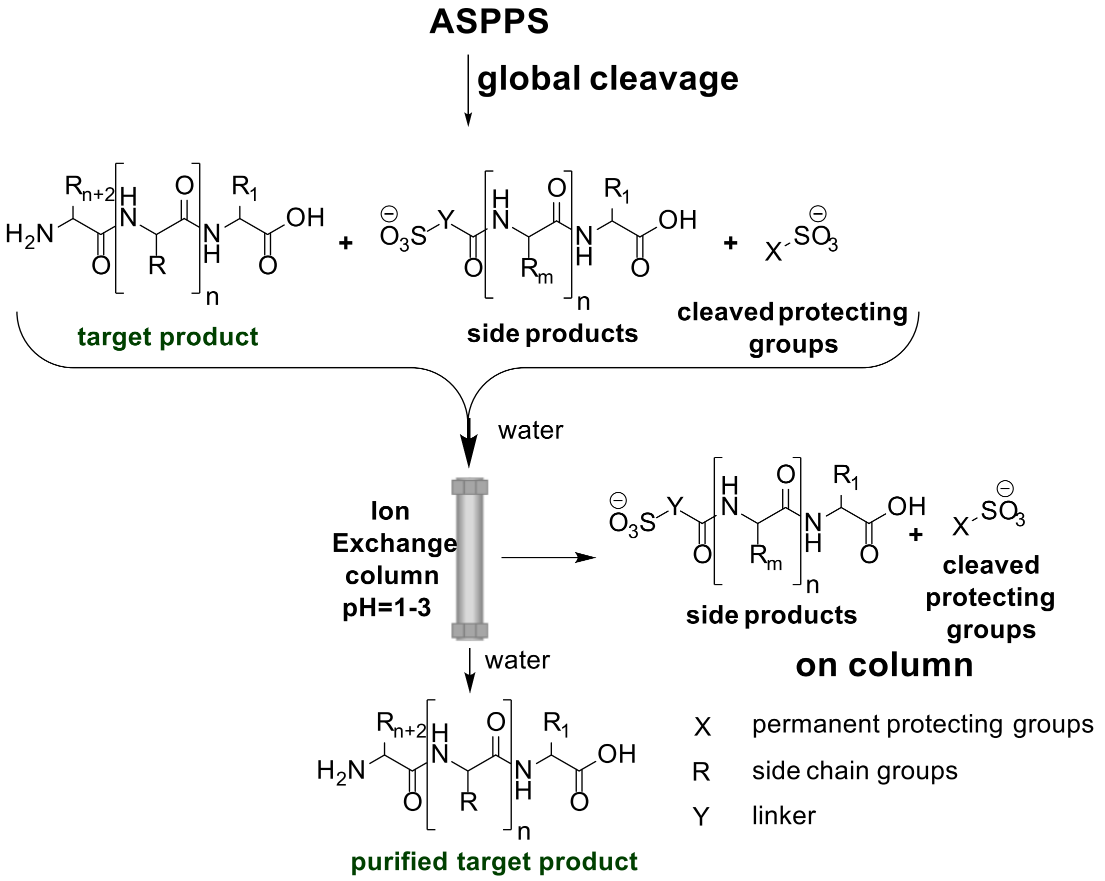

Figure S68: Purification of peptide by IEC using the *Sulfo-tag* method.

As proof-of-concept, two peptides have been synthesized, applying amino acid building blocks in deficiency to increase side-product formation for purification tests. Hexapeptide-9 **49** has been synthesized in water with 0.95 eq. of N_α-Smoc amino acids, compared to prior coupling, in order to maximize by-product formation. Capping was performed with sulfoacetic acid **76** in water.

H—G-P-Q-G-P-Q—OH

Chemical Formula: $C_{24}H_{38}N_8O_9$
Exact Mass: 582,28
Molecular Weight: 582,62

Figure S69: H-GPQGPQ-OH (Hexapeptide-9) **49** for capping experiments.

Peptide synthesis was carried out according to the general procedure. Synthesis was performed on 100 mg preloaded H-Gln-HMPB-ChemMatrix resin (0.30-0.70 mmol/g, 0.05 mmol; calculated on average loading) in water. Coupling was performed with 0.95 eq. of N_α-Smoc amino acid compared to prior coupling in order to maximize by-product formation, EDC-HCl **37** (2 eq.), Oxyma **39** (1 eq.) and $NaHCO_3$ (1 eq.) as base in 6 mL water for 25 min at ambient temperature. Resin was washed thrice with water followed by capping with sulfoacetic acid **76** (50 eq.), EDC-HCl **37** (60 eq.), Oxyma **39** (50 eq.) and $NaHCO_3$ (100 eq.) as base for 15 min (capping mixture was reused). Deprotection of the Smoc protecting group was performed with 5% piperazine$_{(aq)}$. Smoc-glutamine **10** was used without sidechain protecting group. Peptide **49** was cleaved from solid support using TFA:H_2O (95:5) for 120 min. The crude mixture was analysed by analytical HPLC. Afterwards, the crude mixture was put on a DEAE Sephadex A-25 ion-exchange column for purification. The column was washed twice with water and an additional HPLC of the purified peptide was monitored.

After cleavage from solid support, an HPLC of the crude peptide was performed. Afterwards the pH of the solution was adjusted to pH=2 and the solution was transferred to a DEAE Sephadex A-25 ion-exchange column. After washing the column with additional water, a HPLC of the mixture was performed.

The second peptide was synthesized in a similar way. Adrenorphin **77** was been synthesized in DMF with 0.95 eq. of an N_α-Fmoc-amino acid compared to prior coupling in order to maximize by-product formation. Capping was performed with a 4-sulfobenzoic acid **75** salt in DMF.

H—Y-G-G-F-M-R-R-V—NH_2

Chemical Formula: $C_{44}H_{69}N_{15}O_9S$

Exact Mass: 983,51

Molecular Weight: 984,19

Figure S70: H-YGGFMRRV-NH_2 Adrenorphin **77** for capping experiments in DMF.

Synthesis was performed on 100 mg ChemMatrix rink amide resin (0.4-0.6 mmol/g, 0.05 mmol; calculated on average loading) by manual standard Fmoc-based SPPS in DMF. Coupling was performed with 0.95 eq. of Fmoc-amino acid compared to prior coupling in order to maximize by-product formation, HBTU (0.95 eq. to the amino acid) and DIPEA (2 eq. to the amino acid) as base in 6 mL DMF for 25 min at ambient temperature. Resin was washed thrice with DMF followed by capping with an organic 4-sulfobenzoic acid **75** salt in DMF (50 eq.), HBTU (49.8 eq.) and DIPEA (100 eq.) as base for 15 min (capping mixture was reused). Deprotection of the Fmoc-protecting group was performed with 20% piperidine in DMF. Peptide **77** was cleaved from solid support using TFA:H_2O (95:5) for 120 min. An analytical HPLC of the crude mixture was monitored. Afterwards, the crude mixture was put on a DEAE Sephadex A-25 ion-exchange column for purification. The column was washed twice with water and an additional HPLC of the purified peptide was monitored.

After cleavage from solid support, an HPLC of the crude peptide was performed. Afterwards the pH of the solution was adjusted to pH=1 and the solution was applied to a DEAE Sephadex A-25 ion-exchange column. After washing the column with additional water, a HPLC of the mixture was performed.

Both proof-of-concept methods show good results for the peptide purification process. Most of the side products are removed by IEC in around 1 minute. Interestingly, this method could be used for ASPPS as well as for classic DMF-based SPPS processes.

Purification by IEC could be used as only purification step if a certain level of purity is required. For pharmaceutical peptides, an additional HPLC would be necessary of course, but as most of the side-products are removed prior to the HPLC purification, the separation should be more efficient and reduced amounts of organic solvents should be assumed. This method is also applicable as pre-purification system or as part of multicolumn chromatography (MCC) systems.

The same concept could be used in another way. As proof-of-concept an excess of Smoc-Gly-OH **12** was applied to a DEAE Sephadex A-25 ion-exchange column. After the ion-exchange chromatography, the HPLC chromatogram showed no detectable amounts of Smoc-Gly-OH **12** in the solution. After elution from the column, the excess of Smoc-Gly-OH **12** was detected in HPLC again. This could be used to remove all Sulfo-tagged impurities from the wastewater or for a possible regeneration of N_α-Smoc amino acids, used in excess, after coupling steps. Results are shown in **Figure S71**.

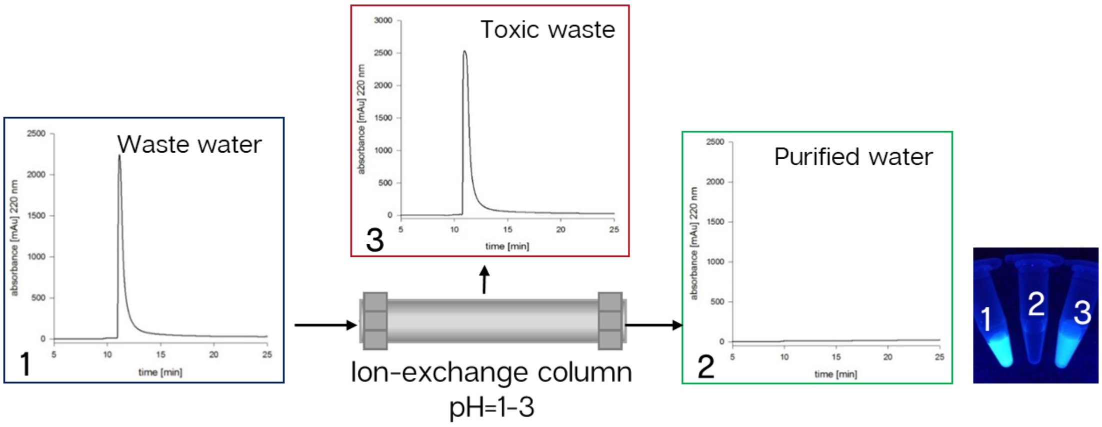

Figure S71: Removal of N_{α}-Smoc amino acid excess from wastewater by IEC. 1 shows the analytical HPLC of the wastewater, 2 the HPLC after the IEC, 3 shows the elution from the IEC. The picture on the right shows the fluorescence of 1-3 upon UV-irradiation as additional analytical method. HPLC traces were monitored at λ=220 nm with a gradient of 0to40 MeCN, see section 7.2.3 for details.

1.9. Racemization studies

Two model peptides were synthesized by ASPPS to investigate potential racemization behaviour during the synthesis. The two peptide sequences are shown in the following **Figure S72**.

H—C-Y-E-I-S—NH_2 Chemical Formula: $C_{26}H_{40}N_6O_9S$

H—A-N-K-P-G—NH_2 Chemical Formula: $C_{20}H_{36}N_8O_6$

Figure S72: Model peptides H-CYEIS-NH_2 **61** and H-ANKPG-NH_2 **62** for racemization tests.

The synthesis of both peptides was performed on ChemMatrix H-Rink amide resins that was loaded in double coupling (2×25min) with a solution of N_{α}-Smoc amino acids (3 eq.), EDC-HCl **37** (5.5 eq.), Oxyma **39** (3 eq.) and $NaHCO_3$ (3 eq.). Smoc deprotection was carried out with 1M NaOH for 5 and 10 minutes. NaOH was used as base, as this is the strongest base used in the ASPPS process. If there are base catalyzed side-reactions during the synthesis process, it should increase the amount of side products. Coupling of the following amino acids was performed with 3 eq. N_{α}-Smoc amino acids, 5.5 eq. EDC-HCl **37**, 3 eq. Oxyma **39** and 3 eq. $NaHCO_3$ as base. Smoc-Asn-OH **7** and Smoc-Tyr-OH **29** were used without side-chain protecting groups. Peptide samples of **61** and **62** were sent to *C.A.T. GmbH & Co Chromatographie und Analysentechnik KG (Tübingen, Germany)* for the analysis of enantiomeric purity. The obtained results are shown in **Table S43**. Since the measured racemization for Asn appeared to be quite high, an additional sample Smoc-Asn-OH **7** was send to *C.A.T. GmbH & Co Chromatographie und Analysentechnik KG (Tübingen, Germany)* for analysis.

Table S43: Determined racemization levels of the amino acids in both test peptides

Amino acid	Enantiomeric composition
**Isoleucine**	>99.7% L-Isoleucine
	<0.10% D-Isoleucine
	<0.10% L-*allo*-Isoleucine
	<0.10% D-*allo*-Isoleucine
**Serine**	<0.10% D-Enantiomer
**Cysteine**	<0.10% D-Enantiomer
**Glutamine/Glutamic acid**	<0.10% D-Enantiomer
**Tyrosine**	0.25% D-Enantiomer
**Alanine**	0.50% D-Enantiomer
**Proline**	0.43% D-Enantiomer
**Asparagine/Aspartic acid**	2.04% D-Enantiomer
**Lysine**	0.11% D-Enantiomer
**Smoc-Asn-OH 7**	0.30% D-Enantiomer

The racemization of the amino acids during the ASPPS corresponds to those expected within the framework of a Fmoc-based SPPS in DMF. The slightly increased values for proline and alanine can be attributed to the synthesis process of amino acids, which was therefore easily adapted for future syntheses. The elevated level of racemization in the case of Asn cannot be explained by the used N_α-Smoc amino acid, which contains 0.30% D-enantiomer. This indicates a base-catalyzed side reaction during the synthesis process. As the other amino acids showed no anomalies, and the used base concentration of $NaHCO_3$ during the coupling process is too low to cause racemization it can only be a result of Smoc deprotection with NaOH. Therefore, additional aspartimide formation studies have been performed as this was suspected to be the source of racemization of Asn during the synthesis process.

1.10. Aspartimide formation studies

Aspartimide formation in Fmoc-SPPS is a well-documented side reaction occurring frequently at Asn-R or Asp-R where R is an amino acid such as Gly, Ala or Ser.[5-17]

The treatment of the peptides containing Asp/Asn with bases such as piperidine results in the formation of the cyclic D/L aspartimide intermediate. Depending on the following reaction, multiple possible side products are formed. Hydrolysis of the D/L aspartimide intermediate could result in the formation of the D/L-α- or the D/L-β-peptides. Nucleophilic attack of the base or other amines could result in the corresponding D/L-α- or the D/L-β-peptide adducts (shown in **Figure S73**).[8-10, 18]

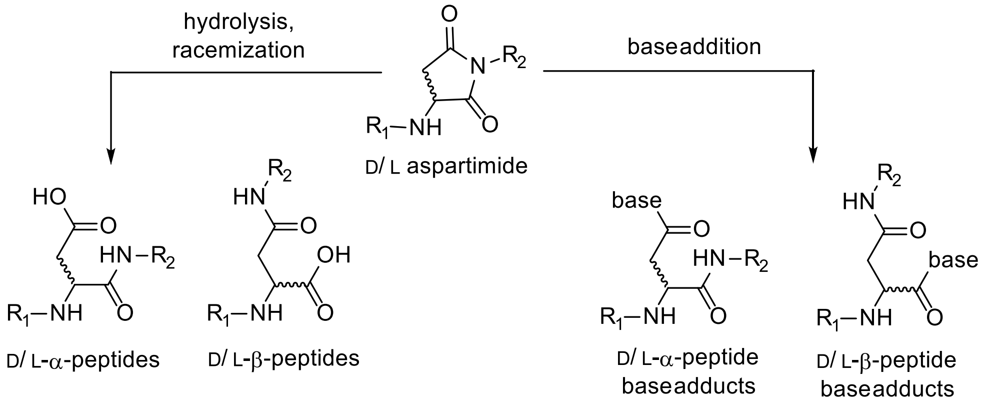

Figure S73: Aspartimide formation under basic conditions and the possible side products. (R_1, R_2, R_3: residues).

To clarify whether aspartimide formation during ASPPS is a frequent side reaction, four model peptides derived from peptide scorpion toxin II (H-VKDGYI-NH_2 **70**, H-VK(D-D)GYI-NH_2 **71**, H-VKNGYI-NH_2 **72** and H-VK(β-D)GYI-NH_2 **73**) were synthesized.

H—V-K-D-G-Y-I—NH_2	H—V-K-N-G-Y-I—NH_2	H—V-K-d-G-Y-I—NH_2	H—V-K·bD·G-Y-I—NH_2
Chemical Formula: $C_{32}H_{52}N_8O_9$	Chemical Formula: $C_{32}H_{53}N_9O_8$	Chemical Formula: $C_{32}H_{52}N_8O_9$	Chemical Formula: $C_{32}H_{52}N_8O_9$
Exact Mass: 692,39	Exact Mass: 691,40	Exact Mass: 692,39	Exact Mass: 692,39
Molecular Weight: 692,82	Molecular Weight: 691,83	Molecular Weight: 692,82	Molecular Weight: 692,82

Figure S74: Model peptides for aspartimide formation H-VKDGYI-NH_2 **70**, H-VKNGYI-NH_2 **72**, H-VK(D-D)GYI-NH_2 **71** and H-VK(β-D)GYI-NH_2 **73**.

Synthesis was performed on 200 mg ChemMatrix Rink amide resin (0.4-0.6 mmol/g, 0.23 mmol; calculated on average loading) by manual standard Fmoc-based SPPS. Afterwards the peptide-resins 70-73 were split into 15 equal parts and incubated for 3h and 16h with the following bases: 1M $NaOH_{(aq)}$, 1M NaOH in EtOH, 5% piperazine in DMF, 5% $piperazine_{(aq)}$, 20% piperidine in DMF and 10% $ethanolamine_{(aq)}$. As a reference, a non-incubated resin was subjected to global cleavage. After the respective incubation time, the samples were cleaved from the resin and analyzed by RP-HPLC and LC-MS. Aspartic acid was added as internal standard to all HPLC runs to achieve comparable HPLC retention times.

Afterwards the peptide-resins **70-73** were split into 15 equal parts and incubated for 3h and 16 h with the following bases: 1M $NaOH_{(aq)}$, 1M NaOH in EtOH, 5% piperazine in DMF, 5% $piperazine_{(aq)}$, 20% piperidine in DMF and 10% $ethanolamine_{(aq)}$. As a reference, a non-incubated resin was subjected to global cleavage. After the respective incubation time, the samples were cleaved from the resin and analyzed by RP-HPLC and LC-MS. The results of the 3h and 16h incubation of H-VKDGYI-NH_2 **70** are shown in **Figure S75**. As expected and described in detail in the literature, aspartimide formation depends on the used bases and the incubation time. Using 20% piperidine in DMF results in several side products after 16 hours. The main observed side products are the corresponding base adducts. Using 5% piperazine in DMF results in less side products after 16 hours, as previously described by Wade *et al.*[19]

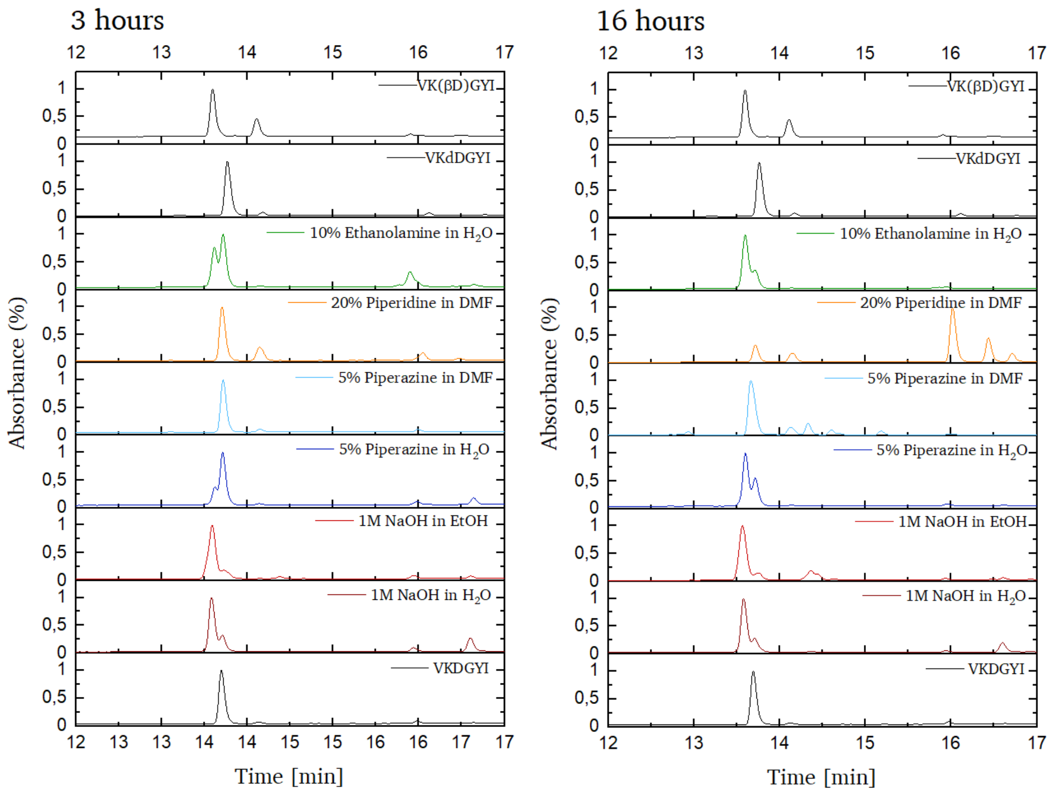

Figure S75: Summary of HPLC data of the incubation of H-VKDGYI-NH_2 **70** with different bases for 3h (left) and for 16h (right) and the resulting aspartimide side product formation caused by the used bases. Retention times were calibrated using ascorbic acid as internal standard; absorbance was normalized between 0 and 1. HPLC traces were monitored at λ=220 nm with a gradient of 0to40 MeCN, see section 7.2.3 for details.

The formation of the D-product and the β–product was not observed in organic solvents. Interestingly, using NaOH in either water or alcohol mainly results in the formation of the β–product, the D-product was not observed. Using ethanolamine in water results mainly in the formation of the β–product. The usage of 5% piperazine in water results in the formation of the β–product in a ratio of 1:3 (β–product:α-product) after 3h, after 16h incubation time the ratio is switched to 3:2 (β–product: α-product). To sum it up, in organic solvents the observed side products are mainly the corresponding L/D-α-base adducts or L/D-β-base adducts, the formation of the β–product was not observed. In water-based synthesis mainly the β–product is formed as side product. The ratio of the β-product formation corresponds to the D/L-β-peptides and the D/L-α-peptide ratio described in the literature for the aspartimide formation of proteins under *in vivo* conditions (3:1 ,observed in amyloid beta-protein,[20] and 2:1[21]). Using stronger bases like NaOH in water results in an inreased amount of β–product formation. This could depend on the base strenght but is more likely based on the nearly instantious cleavage of the *tert*-butyl ester side side chain of Asp, resulting in a high amount of reactive species. Therefore, NaOH could not be used in ASPPS if esters are applied as linkers or side chain protecting groups.

The results of the 3h and 16h incubation of H-VK(D-D)GYI-NH_2 **71** are shown in **Figure S76**. As expected, the incubation of H-VK(D-D)GYI-NH_2 **71** for 3h and 16h yielded the same results as the incubation of H-VKDGYI-NH_2 **70**. Main side products in organic solvents are the corresponding L/D-α-base or L/D-β-base adducts, the formation of the β–product was not observed as well. In water-based synthesis mainly the β–product is formed as side product in the same ratio as observed during the H-VKDGYI-NH_2 **71** incubation. Interestingly, the incubation with 10% ethanolamine in water also results in the formation of L-product; this was the only time when racemization was observed. After 16h, a ratio of 2.3:1:1 (β-peptide: D-peptide:L-peptide) was observed.

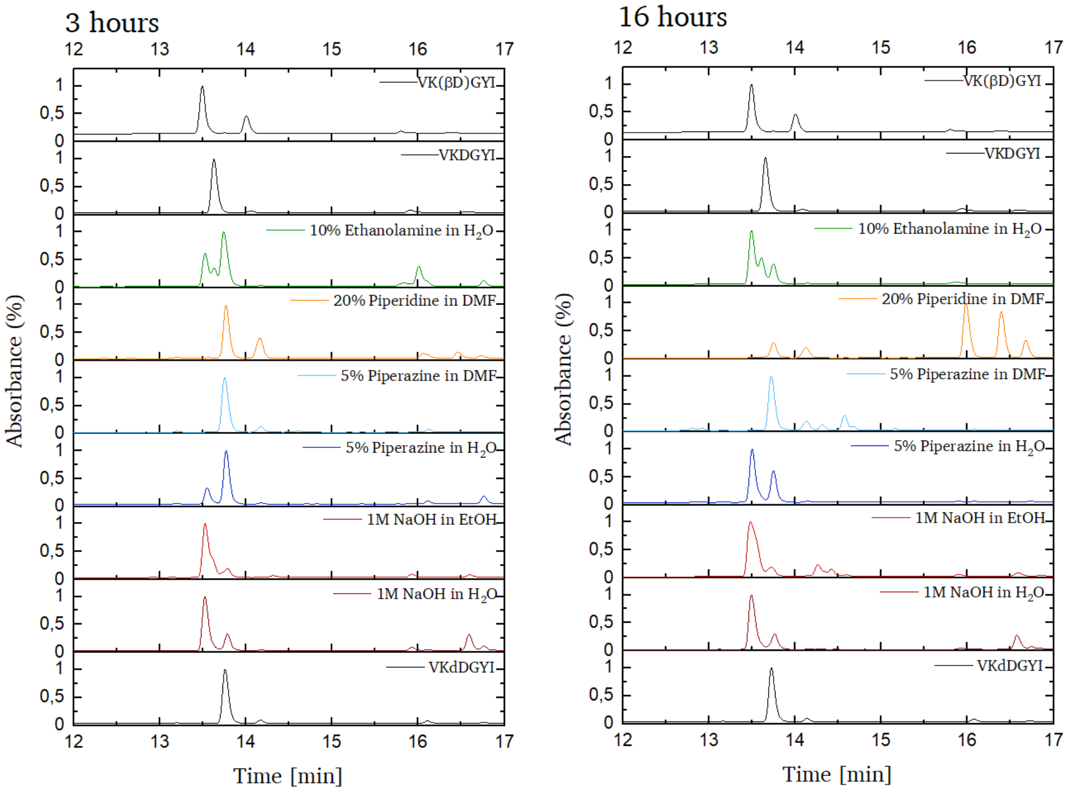

Figure S76: Summary of HPLC data of the incubation of H-VK(D-D)GYI-NH_2 **71** with different bases for 3h (left) and for 16h (right) and the resulting aspartimide side product formation caused by the used bases. Retention times were calibrated using ascorbic acid as internal standard; absorbance was normalized between 0 and 1. HPLC traces were monitored at λ=220 nm with a gradient of 0to40 MeCN, see section 7.2.3 for details.

As Asn is prone to aspartimide formation as well, the same experiments were performed with H-VKNGYI-NH_2 **72**, the results of the 3h and 16h incubation are shown in **Figure S77**. Using 20% piperidine and 5% piperazine is DMF results in a minimal amount of side products after 16h of incubation. 5% piperazine is water delivers the results similar to those obtained for the DMF-based incubation after 16h, after 3h there seem to be a side product formation that was not observed after 16h. The usage of 10% ethanolamine results in the formation of minor side products after 3h and 16h. Usage of NaOH in water or ethanol results in the formation of side products. With increasing time, the incubation with NaOH in ethanol seems to increase a possible deamination of Asn. Interestingly, this was not observed with other bases in water or DMF. The formation of a side product observed upon NaOH incubation is not time-dependent, but its isolation and LC-MS identification was not possible. In summary, using 5% piperazine for Smoc deprotection of aspartimide-prone sequences is advised. In comparison with 20% piperidine in DMF, less side product formation is observed, but compared to 5% piperazine in DMF there is a significant increase of side product formation. This β-peptide side product seems to be caused less by the base itself but by the aspartimide formation in water itself, as observed by proteins degradation under *in vivo* conditions.

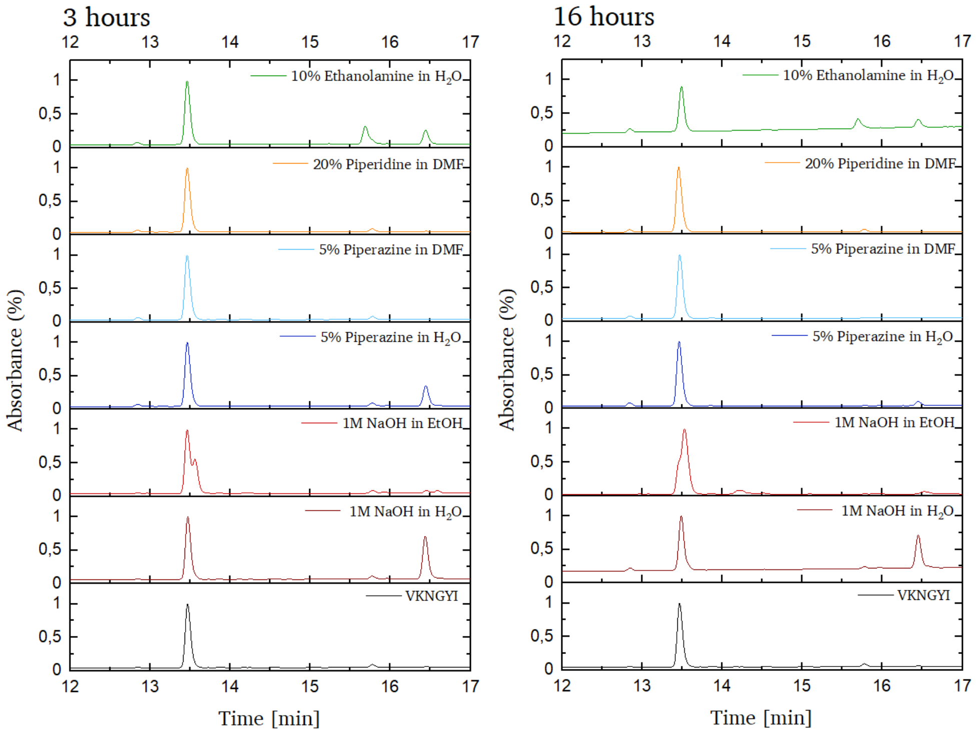

Figure S77: Summary of HPLC data of the incubation of H-VKNGYI-NH_2 **72** with different bases for 3h (left) and for 16h (right) and the resulting aspartimide side product formation caused by the used bases. Retention times were calibrated using ascorbic acid as internal standard; absorbance was normalized between 0 and 1. HPLC traces were monitored at λ=220 nm with a gradient of 0to40 MeCN, see section 7.2.3 for details.

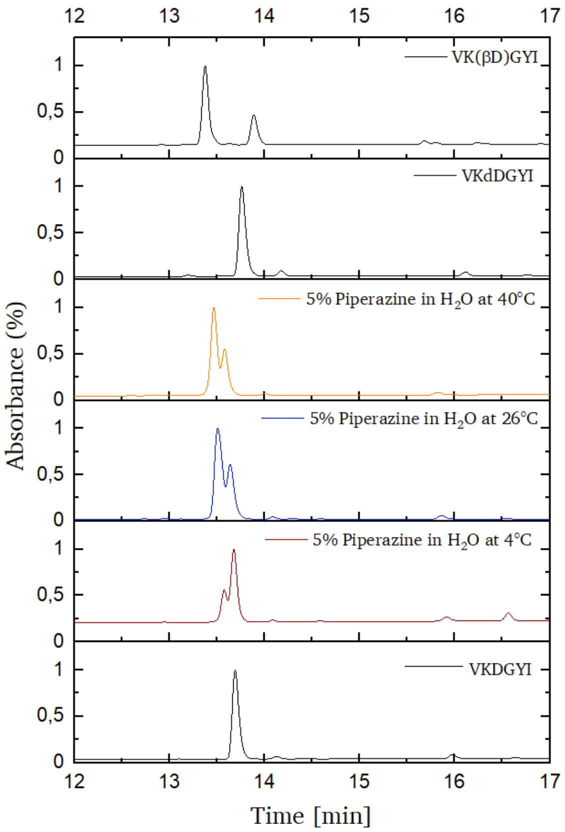

Figure S78: Summary of HPLC data of the incubation of the temperature dependent formation of H-VK(β-D)GYI-NH_2 **73** with 5% Piperazine in water after 16 h. Retention times were calibrated using ascorbic acid as internal standard; absorbance was normalized between 0 and 1. HPLC traces were monitored at λ=220 nm with a gradient of 0to40 MeCN, see section 7.2.3 for details.

Based on the literature, the protein degradation should increase at higher temperatures and decrease at the lower ones. Therefore, an additional incubation of H-VKDGYI-NH_2 **70** with 5% piperazine in water was performed at 4°C, RT and 40°C respectively. The temperature-dependent formation of H-VK(β-D)GYI-NH_2 **73** with 5% piperazine in water after 16h is shown in **Figure S78**.

The obtained results show that the aspartimide formation in water is temperature dependent. At 4°C the (β–product:α-product) ratio is 1:3 and with increasing temperature shifted at 40°C to a ratio of 2.5:1. Therefore, deprotection of aspartimide-prone sequences in water should be performed at 4°C or below this temperature to reduce the amount of aspartimide side products.

1.11. Fluorescent properties of the Smoc group

Due to its particular electronic structure, the Smoc group adsorbs in UV/Vis spectral area and possesses fluorescent properties, being therefore detectable by respective analytical methods. It is spectrally active and fluorescent both as N_{α}-Smoc amino acids and in its cleaved form. This is a significant advantage over the classical Fmoc group, which can only be observed after deprotection,[22] and allows to establish a real-time monitoring of the synthesis process. **Figure S79** shows a direct comparison of Smoc-Gly-OH **12** with Fmoc-Gly-OH upon UV irradiation.

A 100nM aqueous solutions of all *Nα*-Smoc amino acids were prepared and transferred to a Greiner Bio-One (Kremsmünster, Austria) UV-STAR®, flat-bottom, black 96 well plate for fluorescent measurements. Absorbance and fluorescent measurements were performed with a CLARIOstar (BMG LABTECH GmbH, Ortenberg, Germany), absorbance was measured in the range of 220-700nm and fluorescence was measured in the range of 300-400nm.

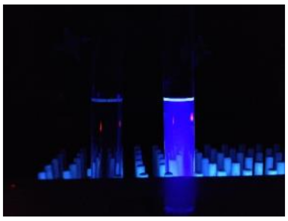

Figure S79: A sample of Smoc-Gly-OH **12** (right) and Fmoc-Gly-OH (left) as a reference, illuminated at 254nm.

As an example, the absorption spectra and the fluorescence spectra of a 100 nM solution of Smoc-Ala-OH were recorded between 300 nm and 400 nm, results are shown in **Figure S80**. The experimental data shows excitation maxima of 281 nm and emission maxima of 338 nm, resulting in a Stokes shift of 57 nm.

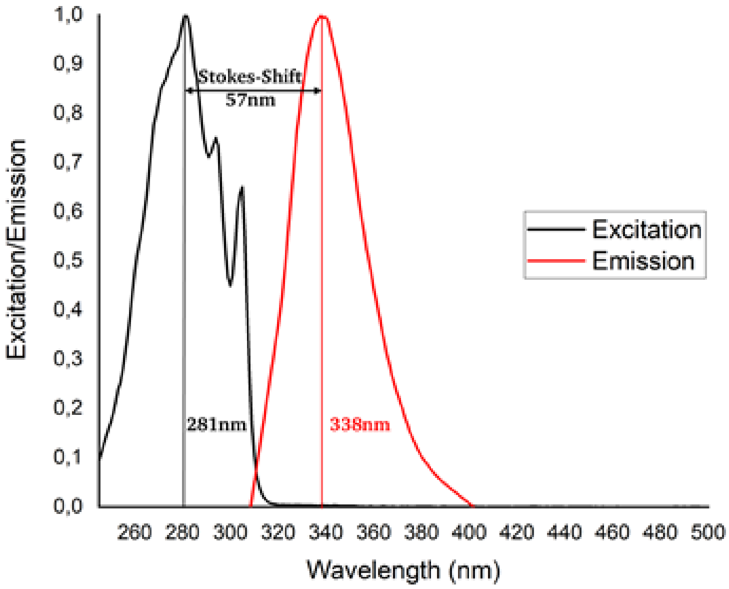

Figure S80: Emission and excitation spectra of Smoc-Ala-OH **3**. Excitation and emission have been normalized.

The determined spectral data of all synthesized N_{α}-Smoc amino acids are listed in **Table S44**. Excitation spectra of all N_{α}-Smoc amino acids were measured between 220 nm and 700 nm. All N_{α}-Smoc amino

acids show absorbance between 200nm and 220 nm (due to technical limitations of the used equipment this is not shown here) but only the range of 245-310 nm was identified as excitation area.

Table S44: Excitation and emission maxima of all synthesized N_α-Smoc amino acids and their Stokes shift in water.

N_α-Smoc amino acid	Excitation maxima	Emission maxima	Stokes shift
Smoc-L-Ala-OH **3**	281 nm	338 nm	57 nm
Smoc-D-Ala-OH **4**	281 nm	340 nm	59 nm
Smoc-L-Arg-OH **5**	282 nm	340 nm	58 nm
Smoc-L-Arg(Pbf)-OH **6**	282 nm (Abs max. 220nm)	338 nm	56 nm
Smoc-L-Asn-OH **7**	281 nm	340 nm	59 nm
Smoc-L-Asp(OtBu)-OH **8**	282 nm	340 nm	58 nm
Smoc-L-Cys(Trt)-OH **9**	282 nm (Abs max. 220nm)	340 nm	58 nm
Smoc-L-Gln-OH **10**	281 nm	338 nm	57 nm
Smoc-L-Glu(OtBu)-OH **11**	282 nm	340 nm	58 nm
Smoc-Gly-OH **12**	281 nm	338 nm	57 nm
Smoc-L-His-OH **13**	281 nm	338 nm	57 nm
Smoc-L-His(Trt)-OH **14**	282 nm (Abs max. 220nm)	338 nm	57 nm
Smoc-L-Ile-OH **15**	282 nm	340 nm	58 nm
Smoc-L-Leu-OH **16**	281 nm	340 nm	59 nm
Smoc-D-Leu-OH **17**	282 nm	340 nm	58 nm
Smoc-L-Lys(Boc)-OH **18**	281 nm	338 nm	57 nm
Smoc-L-Met-OH **19**	281 nm	340 nm	59 nm
Smoc-L-Phe-OH **20**	281 nm	338 nm	57 nm
Smoc-L-Pro-OH **21**	281 nm	340 nm	59 nm
Smoc-L-Ser-OH **22**	281 nm	340 nm	59 nm
Smoc-L-Ser(tBu)-OH **23**	281 nm	340 nm	59 nm
Smoc-L-Thr-OH **24**	281 nm	340 nm	59 nm
Smoc-L-Thr(tBu)-OH **25**	281 nm	340 nm	59 nm
Smoc-L-Trp-OH **26**	281 nm (Abs max. 220, 225nm)	341 nm	60 nm
Smoc-L-Trp(Boc)-OH **27**	281 nm (Abs max. 220nm)	340 nm	59 nm
Smoc-L-Tyr-OH **28**	282 nm	341 nm	59 nm
Smoc-L-Tyr(tBu)-OH **29**	281 nm	340 nm	59 nm
Smoc-L-Val-OH **30**	281 nm	340 nm	59 nm
Smoc-β-Ala-OH **31**	281 nm	340 nm	59 nm
Smoc-Aib-OH **32**	281 nm	338 nm	57 nm

Fluorescence was measured between 300 nm and 500 nm. As the results show, excitation maxima for Smoc was around 280 nm and the emission maxima - around 340 nm. The minor differences between the N_α-Smoc amino acids seem to be caused by the limited measuring accuracy of the used device. Quantum efficiency is critical for fluorescence measurements, however in this work the quantum efficiency of all N_α-Smoc amino acids could not be determined due to a lack of required equipment. Generally, a decreased fluorescence was observed with aromatic amino acids tyrosine and tryptophan as well as with aromatic side chain protecting groups like trityl (Cys, His) or Pbf (Arg). This might be caused by an inter- or intramolecular quenching effect.

In case of Cys the problem of decreased fluorescence can be avoided by using different side chain protecting groups, Arg does not need side-chain protection at all. As proof-of-concept, the loading of a resin and the synthesis of a model peptide were monitored by fluorescence measurements.

1.12. Fluorescence monitoring of resin loading and coupling status during ASPPS

Monitoring of the reaction progress during SPPS requires the usage of additional markers and detection reactions or it is only possible after the Fmoc group has been cleaved off as only the formed dibenzofulvene product is spectrally active.[23-27] The Smoc protecting group allows for detection either by UV/Vis measurements using a photometer (this generally known method is therefore not explicitly discussed here) or by fluorescence monitoring. The fluorescence properties of the Smoc group allow the detection in solution or even if bound to the solid support, thus enabling a real-time monitoring of the coupling and deprotection process for the first time in the history of solid-phase peptide synthesis.

Smoc-Gly-OH **12** was loaded in two different concentrations onto a water-compatible 2-CTC resin with DMSO as solvent. The resin was washed thrice with water, suspended in water and split into three equal portions that were transferred to a Greiner Bio-One (Kremsmünster, Austria) UV-STAR®, flat-bottom, black 96 well plate for fluorescent measurements. The fluorescence was measured at the excitation maxima of 280 nm and emission maxima of 340 nm.

The choice of a solvent for this step was stipulated by the fact that the loading of this resin is hydrolysis-prone under aqueous conditions. The fluorescence was measured at the excitation maxima of 280 nm and emission maxima of 340 nm. The obtained data (shown in **Table S45**) clearly show a dependence between the amounts of coupled amino acid and fluorescence measured.

Table S45: Fluorescence intensity of different on-resin concentrations of Smoc-Gly-OH **12** measured on resin.

Experiment	DMSO	2-CTC resin	Smoc-Gly-resin 0.08mM loading	Smoc-Gly-resin 0.04mM loading
**1**	8	255	6493	2742
**2**	13	235	6363	3212
**3**	6	249	6923	3540
**4**	7	222	6240	3471
**mean value**	8.5	240.25	6504.75	3241.25
**standard deviation**	3.10	14.77	297.35	361.54

To show the real-time monitoring of fluorescence is possible during the ASPPS process, a model peptide with the sequence H-L-V-A-I-G-NH_2 **74** was synthesised on a Rink amide PEGA resin in water.

H—L-V-A-I-G—OH

Chemical Formula: $C_{22}H_{41}N_5O_6$

Exact Mass: 471,31

Molecular Weight: 471,60

Figure S81: H-LVAIG-NH_2 **74.**

H-LVAIG-NH_2 **74** was synthesized on a Rink amide PEGA resin in water according to the general procedure. After each respective coupling or deprotection step, the resin was washed thrice with water, suspended in water and split into three equal portions that were transferred to a *Greiner Bio-One (Kremsmünster, Austria)* UV-STAR®, flat-bottom, black 96 well plate for fluorescent measurements. After the measurements, the resin was reunited and the synthesis continued. After each synthesis step, the fluorescence of the peptide-resin was measured.

The fluorescence measurement was performed by excitation at 280 nm and emission at 340 nm. The fluorescence allows distinguishing between the reaction steps. After Smoc deprotection, the fluorescence adjusts itself on a baseline value with a specific auto-fluorescence. After coupling of an N_α-Smoc amino acid, the fluorescence increases. The amino acids show intrinsically different fluorescence properties. This could be compensated by a normalisation that takes the quantum yield of each amino acid into account. The experiment shows that measuring the fluorescence on the solid phase

to monitor the course of the reaction is basically possible. However, since it is easier to apply, fluorescence detection in solution of the coupling and deprotection mixture is more likely to be used.

Although the fluorescence measurements are possible, the absorption measurements with the Smoc group are probably the easiest way to monitor the reaction in real time.

2. Analytical Methods

2.1. Mass spectrometry

Electrospray ionization mass spectroscopy (ESI-MS) spectra were obtained by using a Shimadzu LCMS-2020 mass spectrometer equipped with a *Phenomenex* Synergi™ Hydro-RP LC Column (4 u, 80 Å, 100 x 3 mm). Eluent system consisted of 0.1% aq. formic acid, LC-MS grade (eluent A) and 100% acetonitrile containing 0.1% formic acid, LC-MS grade (eluent B).

2.2. HR-MS

HR-MS electron ionization were obtained by using a Bruker Impact II.

2.3. Liquid chromatography

Analytical reversed-phase high performance liquid chromatography (RP-HPLC) was performed on a Agilent 1100 series HPLC equipped with a Interchim US5C18HQ-250/046 (5u, 250×4.6mm) at a flow rate of 1 mL/min. Eluent A: 0.1% aq. trifluoroacetic acid (TFA), eluent B: MeCN with 0.1% TFA. 4 min of isocratic flow (starting concentration of eluent B) was followed by 20 min of gradient flow. Absorption was measured by UV/VIS detector at 220 nm and 280 nm.

For isolation of peptides or N_α-Smoc amino acids on a semi-preparative *Interchim* PuriFlash 4250 equipped with a preparative C_{18} column (*Interchim* US5C18HQ-250/212 (5u, 250×21.2mm) was used at a flow rate of 18 mL/min. Eluent A: 0.1% aq. trifluoroacetic acid (TFA), eluent B: MeCN with 0.1% TFA. 5 min of isocratic flow (starting concentration of eluent B) was followed by 20 min of gradient flow. Absorption was measured by UV/VIS detector at 220 nm and 280 nm.

2.4. NMR

^{1}H, ^{13}C, ^{13}C DEPT and the 2D NMR spectra ^{1}H-^{13}C HMBC, ^{1}H-^{1}H NOESY, ^{1}H-^{1}H COSY were recorded with a 500 MHz NMR Spectrometer DRX 500 (Bruker BioSpin GmbH, Karlsruhe) equipped with a 5mm ATMA BBFO probe . All Samples were dissolved in deuterated DMSO d6 purchased from *Sigma Aldrich* (*Merck KGaA*, Darmstadt, Germany) or deuterated MeCN purchased from *Euriso Top* (Gif-Sur-Yvette, France).

3. Analytical data

3.1. Smoc-Chloride 2

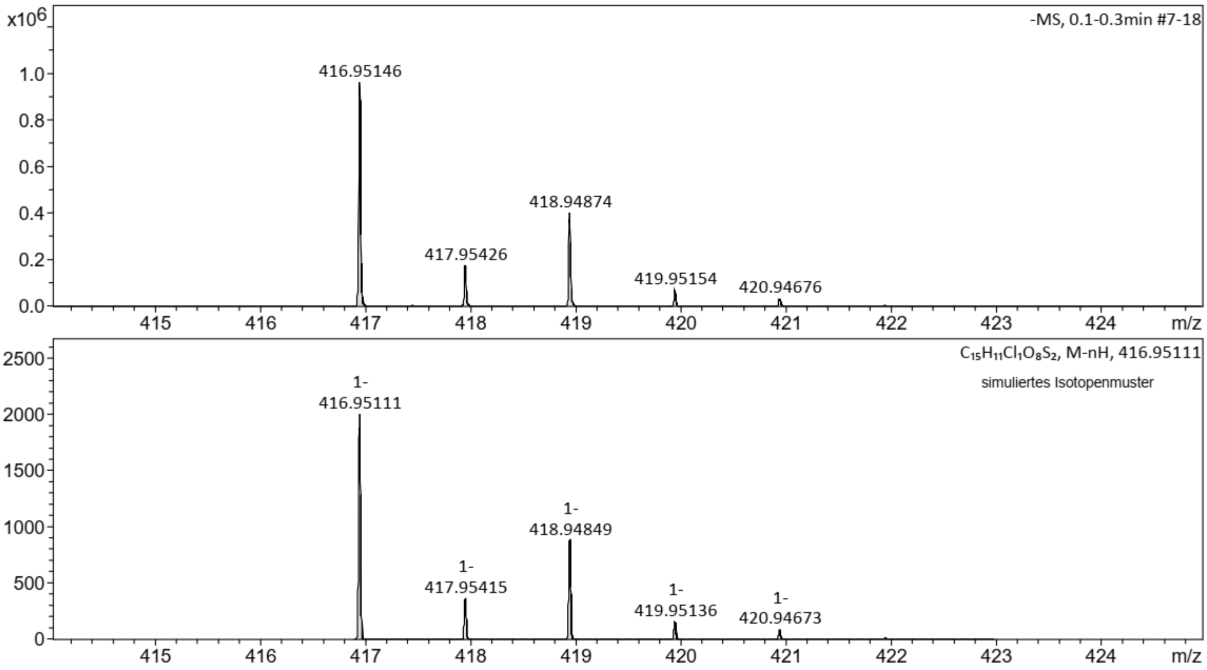

Figure S82: HR-MS of Smoc-chloride **2** (M measured=416.95146 $[M-H]^-$, M calc.=416.95111).

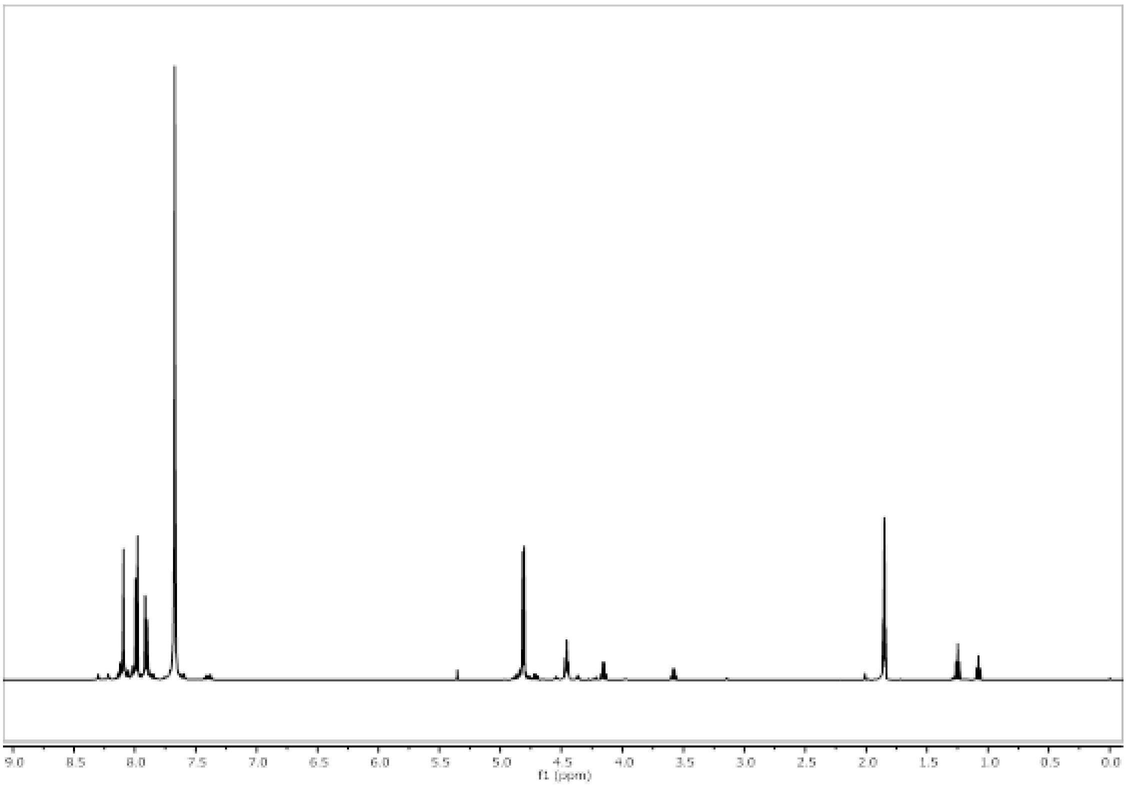

Figure S83: ^{1}H-NMR of Smoc-Cl **2.**

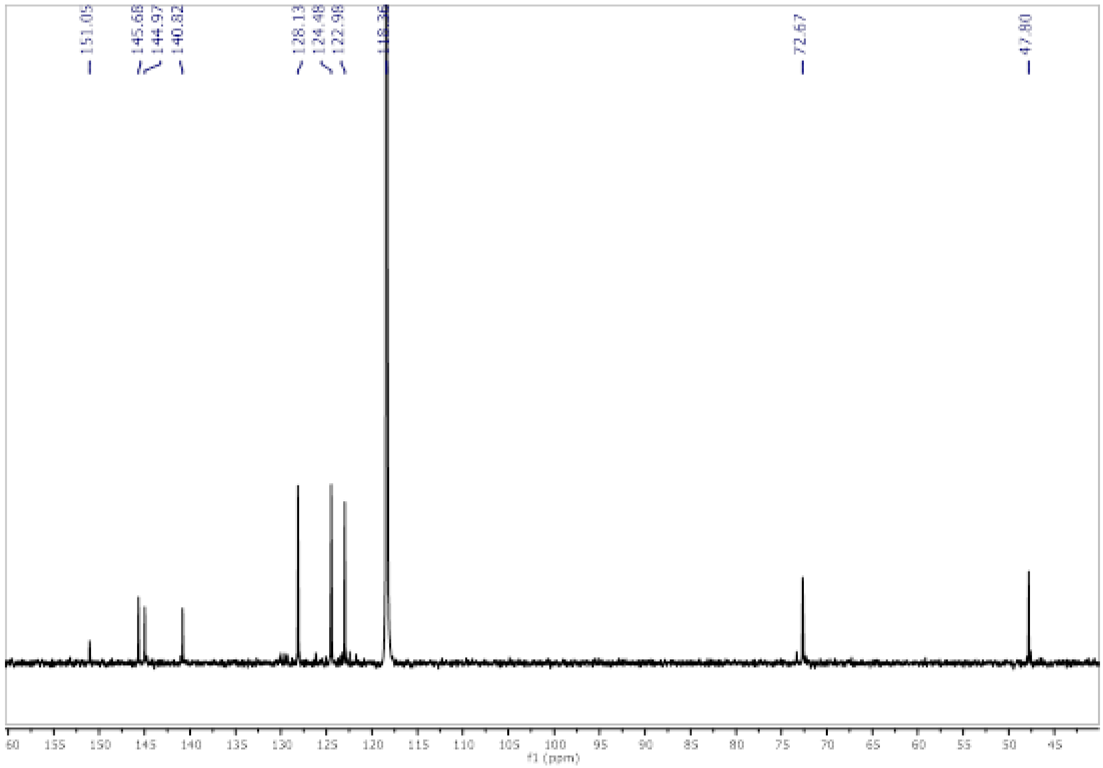

Figure S84: ^{13}C-NMR of Smoc-Cl **2.**

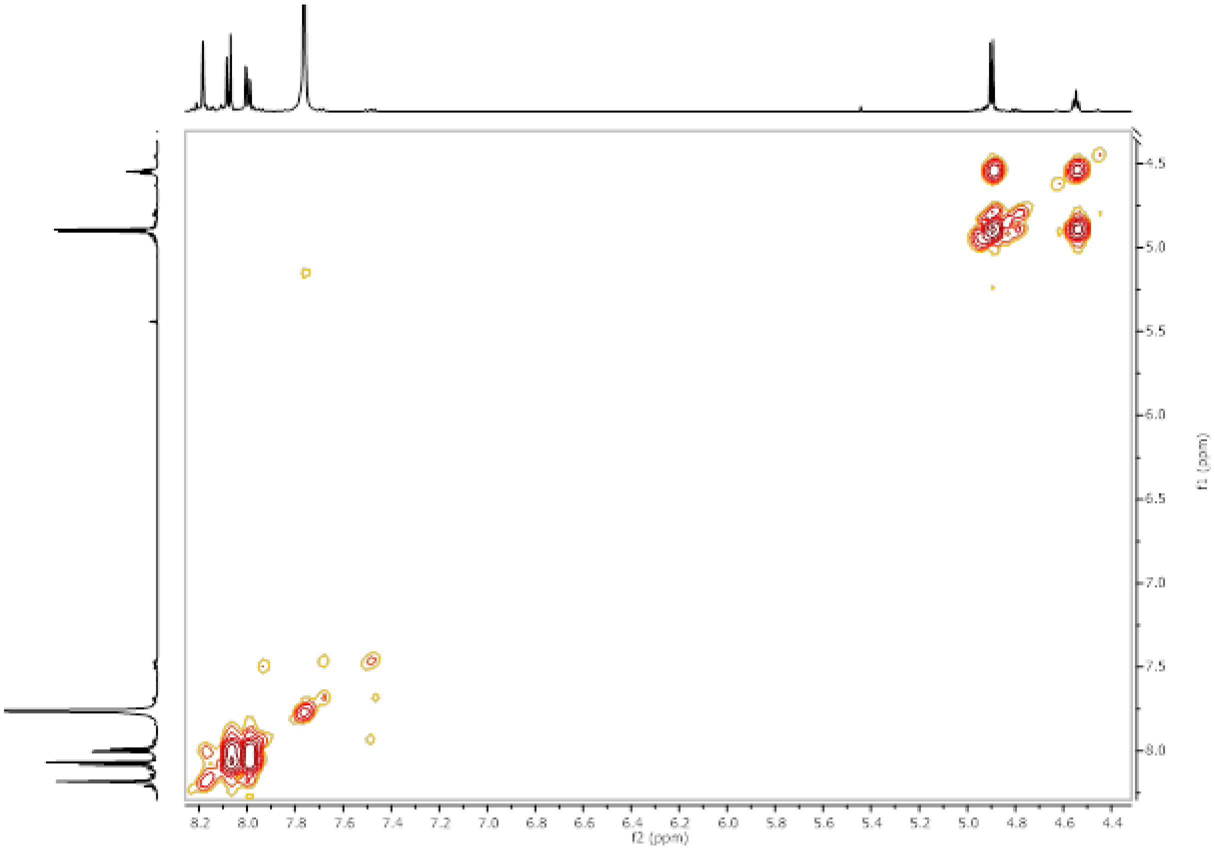

Figure S85: ^{1}H-^{1}H COSY-NMR of Smoc-Cl **2.**

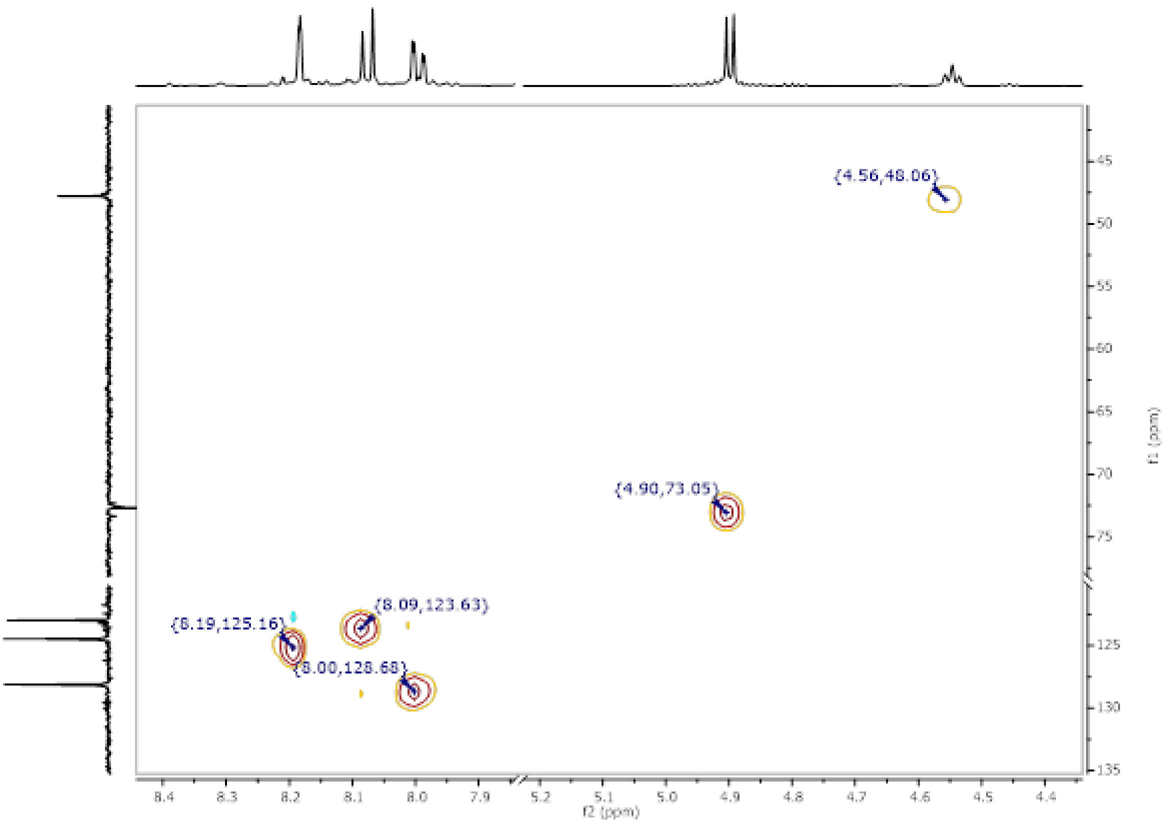

Figure S86: ^{1}H-^{13}C HSQC-NMR of Smoc-Cl **2.**

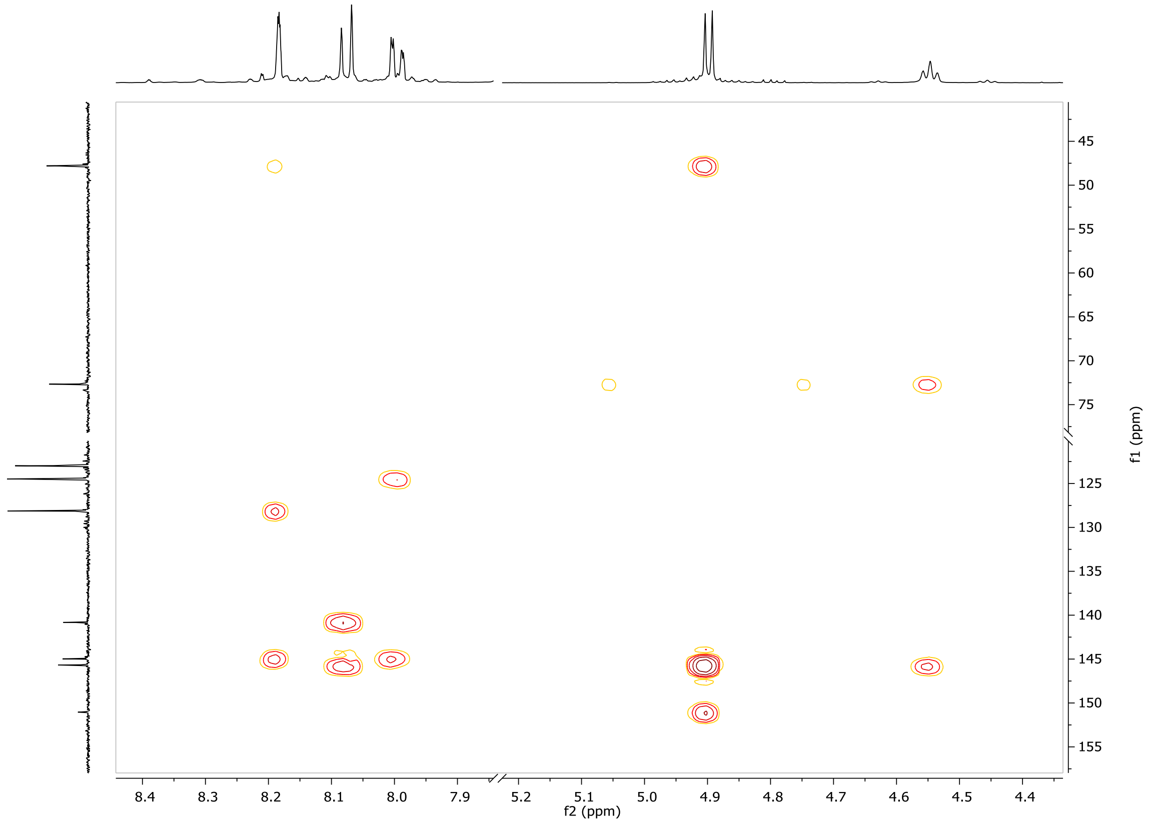

Figure S87: ^{1}H-^{13}C HMBC-NMR of Smoc-Cl **2.**

3.2. Amino Acids

3.2.1. Analytical data of Smoc-L-Ala-OH 3

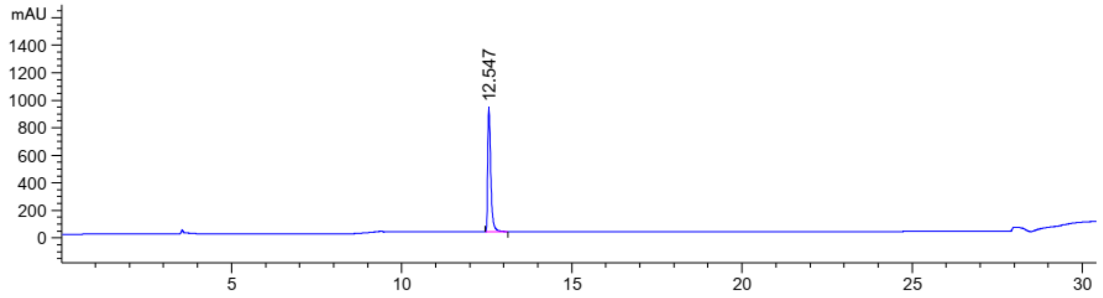

Figure S88: HPLC chromatogram of Smoc-L-Ala-OH **3** at λ=220 nm (0to40 MeCN).

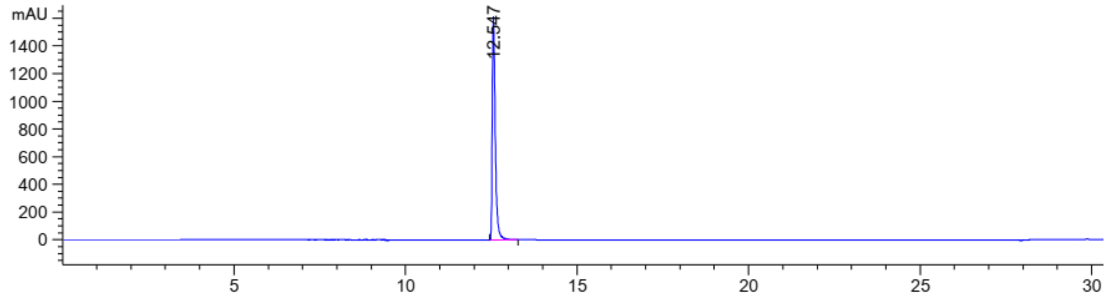

Figure S89: HPLC chromatogram of Smoc-L-Ala-OH **3** at λ=280 nm (0to40 MeCN).

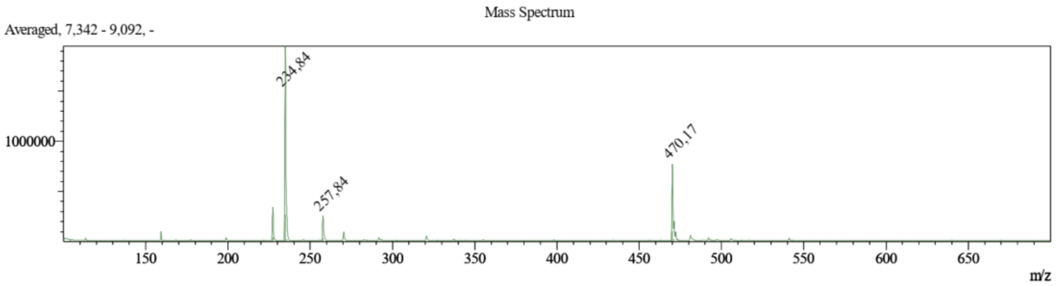

Figure S90: ESI-MS of Smoc-L-Ala-OH **3** (M measured=470.17[M-H]$^-$, M calc.=471.45).

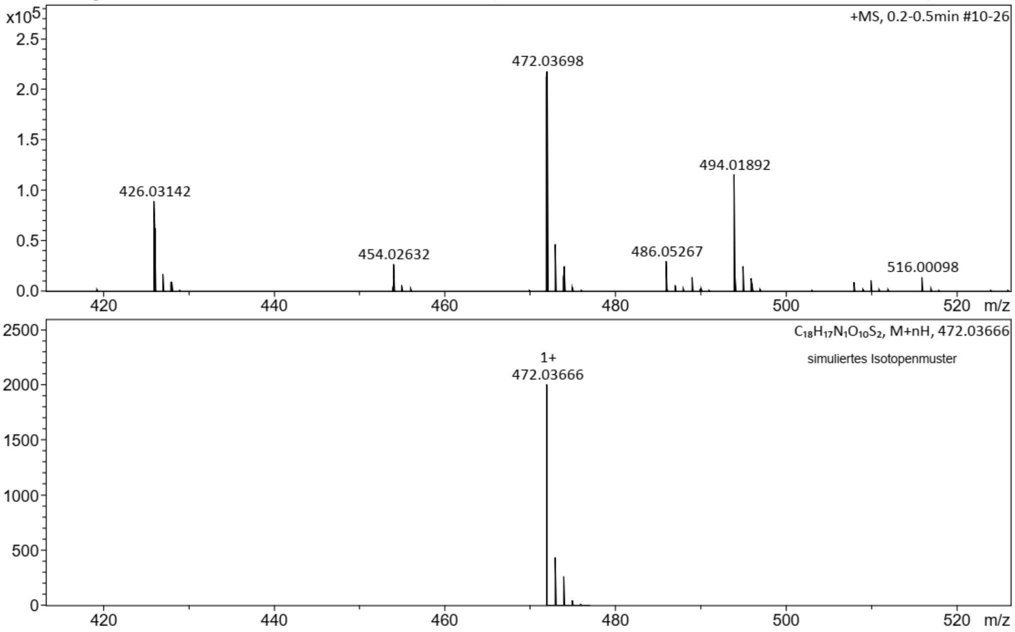

Figure S91: HR-MS of Smoc-L-Ala-OH 3 (M measured=472.03698 [M+H]$^+$, M calc.=472.03666)

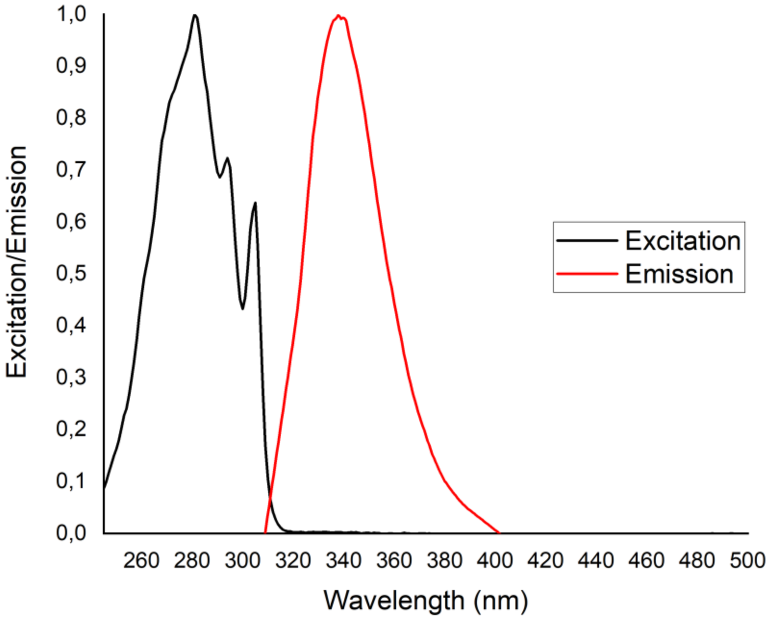

Figure S92: Excitation and emission spectra of Smoc-L-Ala-OH **3**, excitation and emission have been normalized between 0 and 1 for illustration.

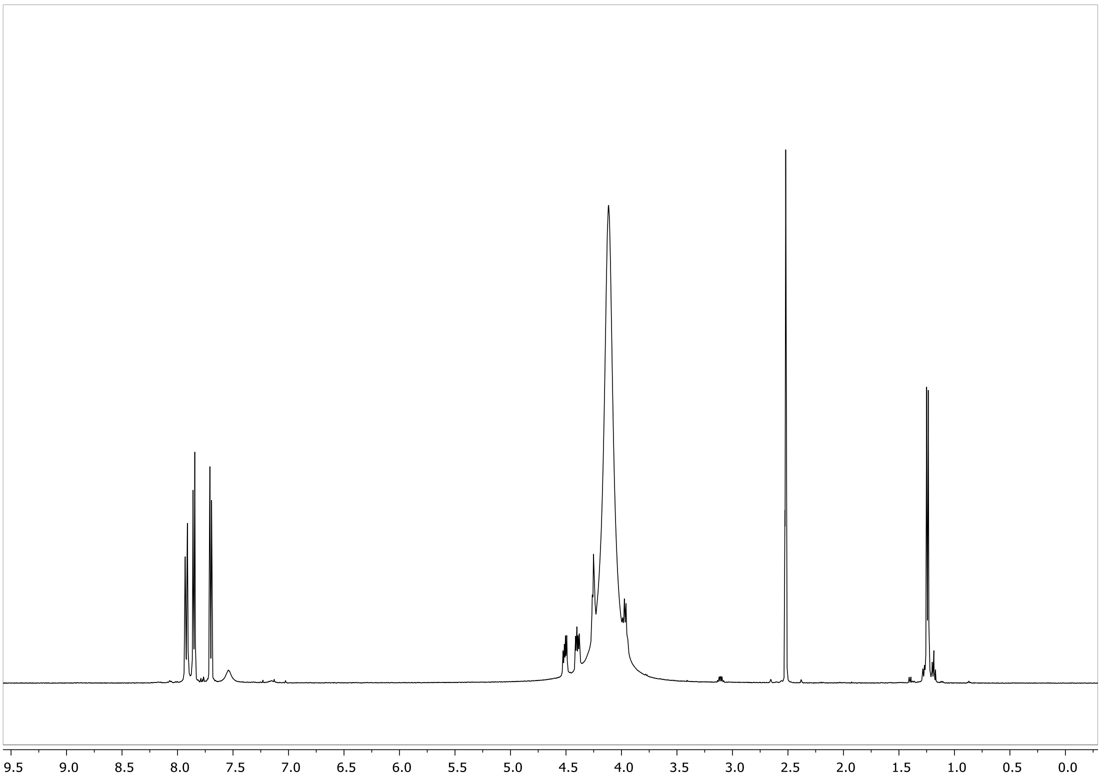

Figure S93: ^{1}H-NMR of Smoc-L-Ala-OH **3**.

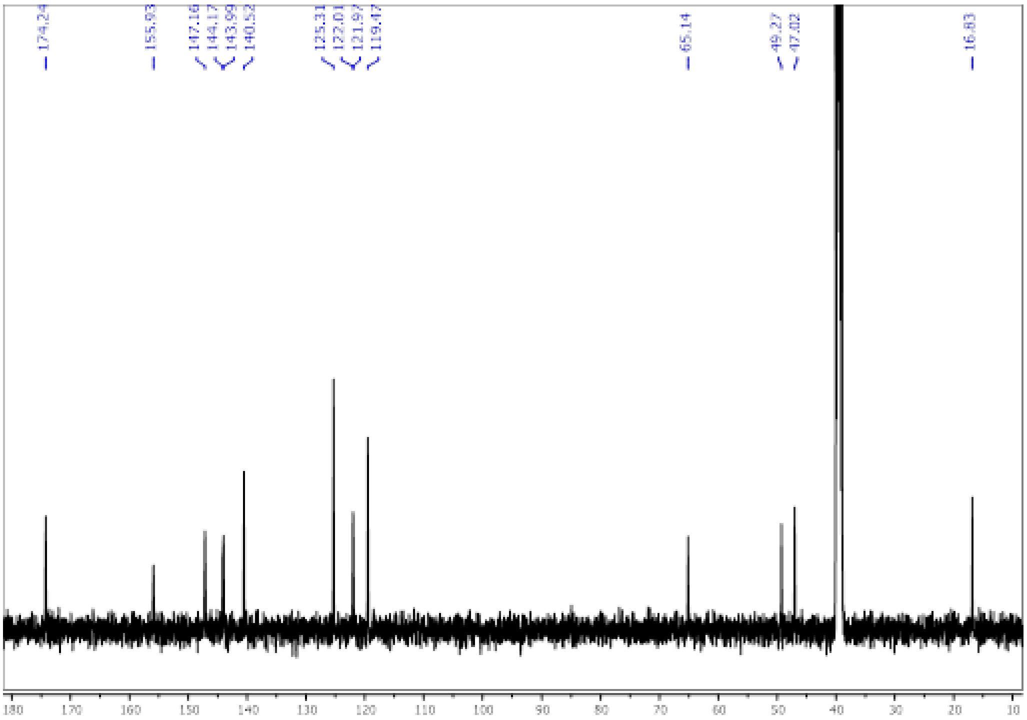

Figure S94: ^{13}C NMR of Smoc-L-Ala-OH **3**.

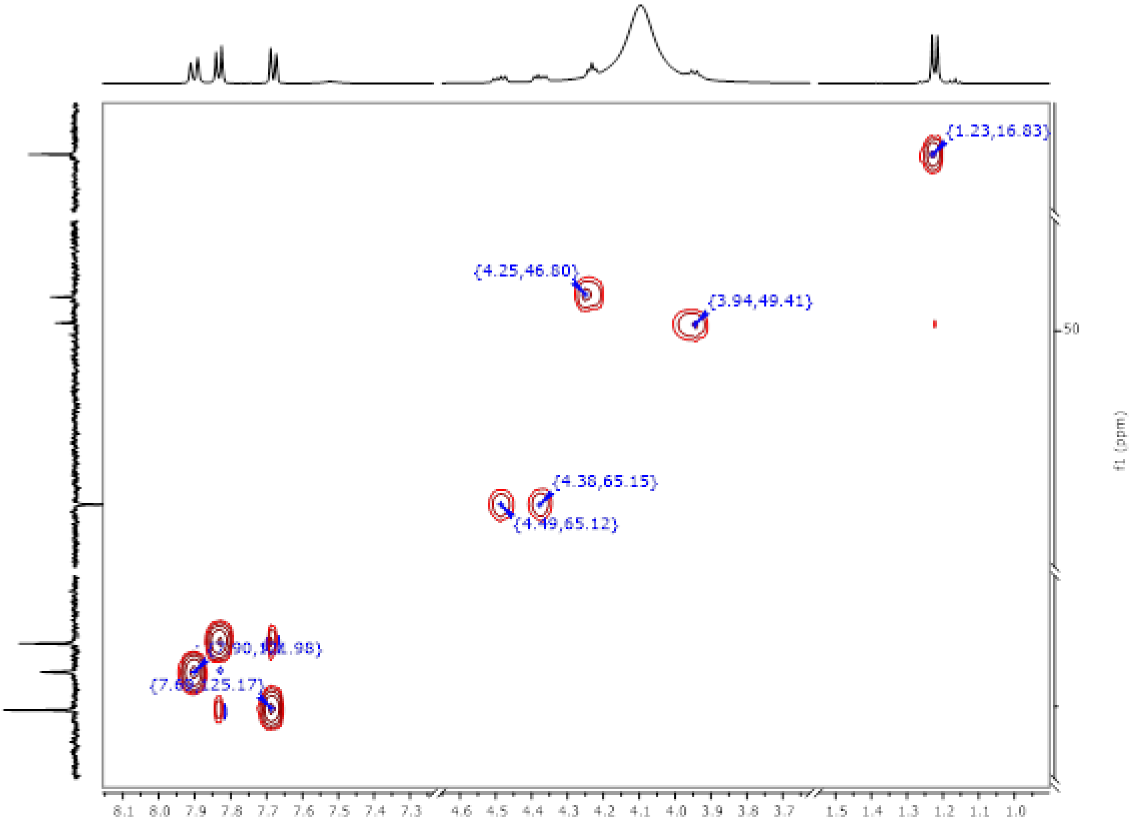

Figure S95: ^{1}H-^{13}C HSQC-NMR of Smoc-L-Ala-OH **3**.

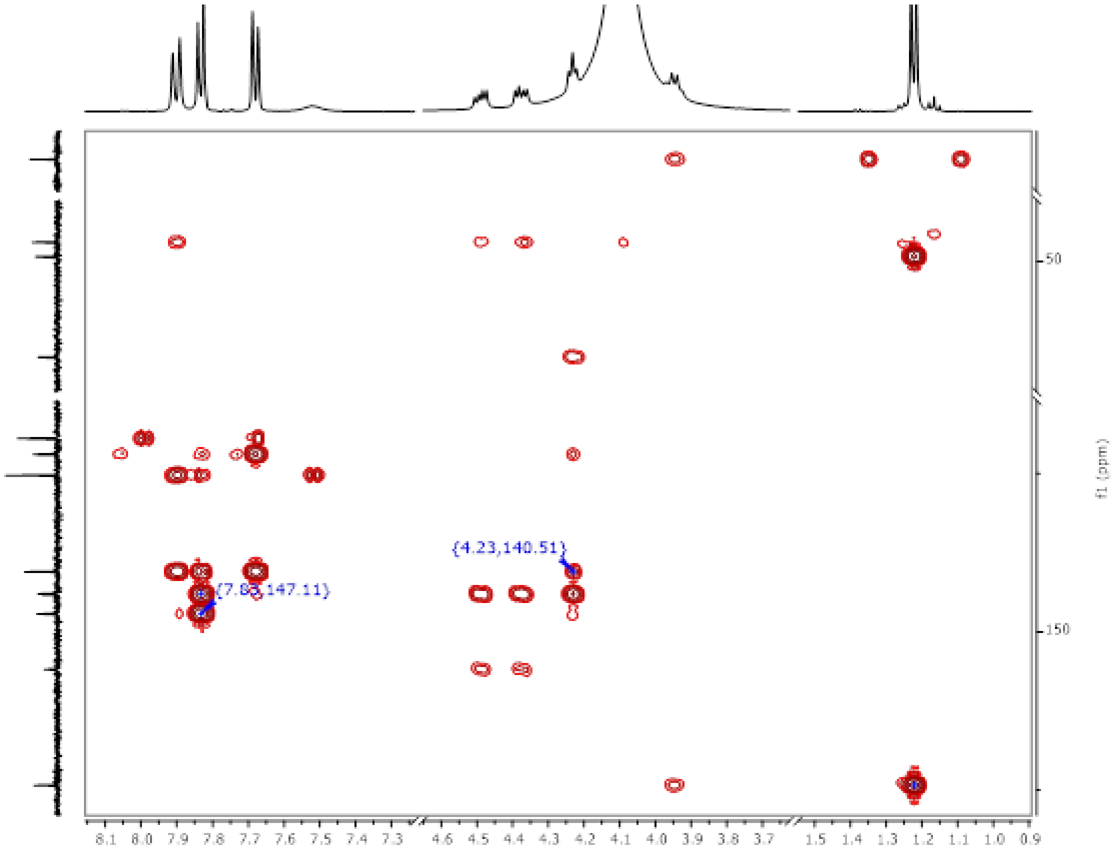

Figure S96: ^{1}H-^{13}C HMBC-NMR of Smoc-L-Ala-OH **3**.

3.2.2. Analytical data of Smoc-D-Ala-OH 4

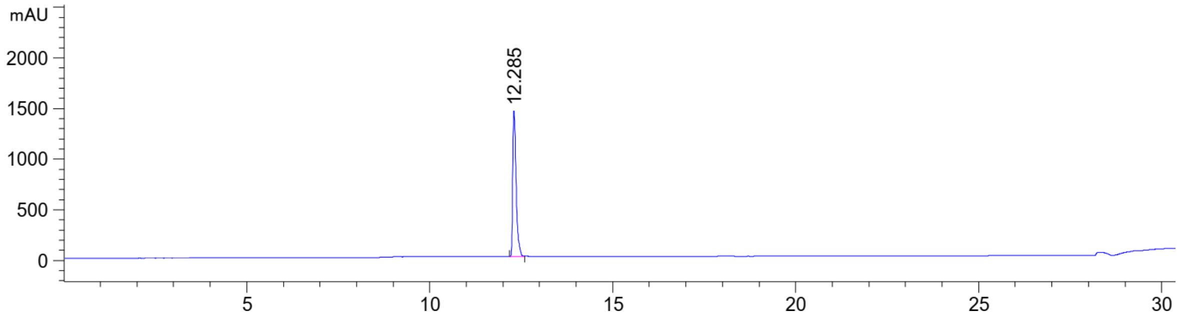

Figure S97: HPLC chromatogram of Smoc-D-Ala-OH **4** at λ=220 nm (0to40 MeCN).

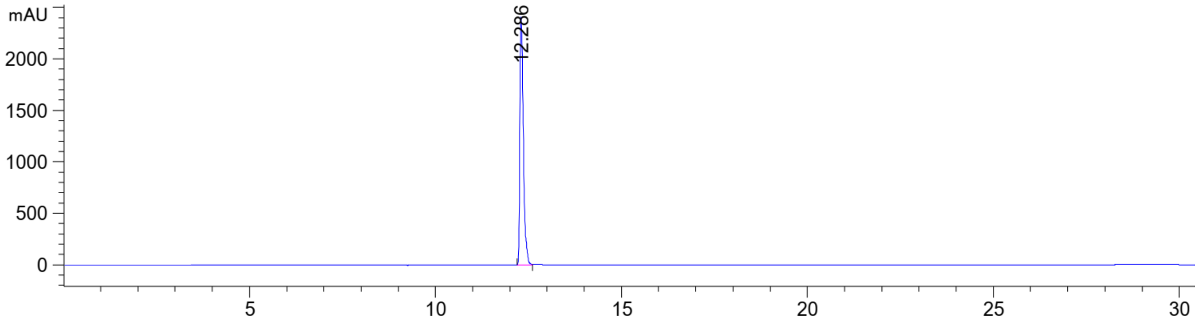

Figure S**98**: HPLC chromatogram of Smoc-D-Ala-OH **4** at λ=280 nm (0to40 MeCN).

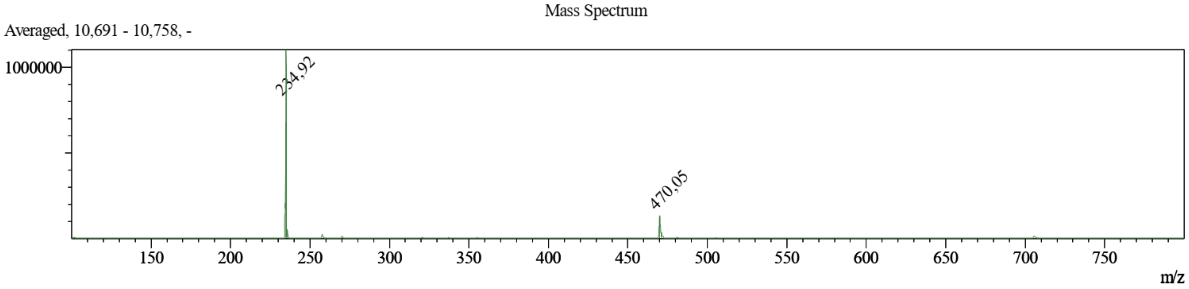

Figure S99: ESI-MS of Smoc-D-Ala-OH **4** (M measured=470.05[M-H]-, M calc.=471.45).

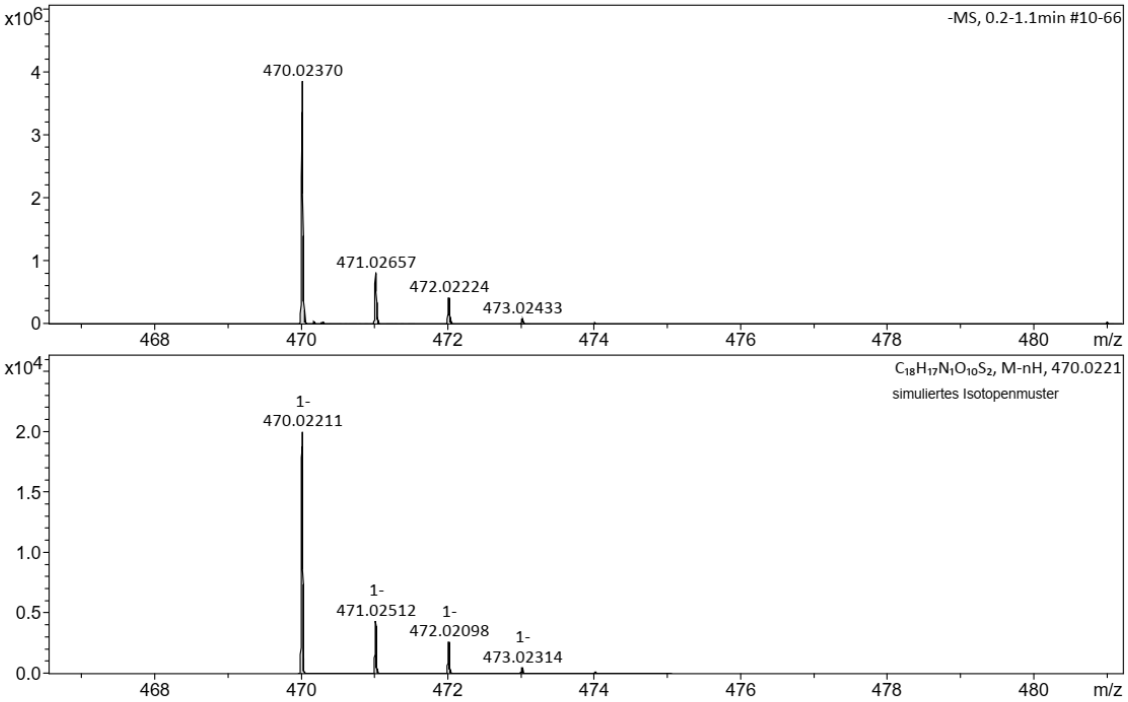

Figure S100: HR-MS of Smoc-D-Ala-OH **4** (M measured=470.02370 [M-H]-, M calc.=470.02211).

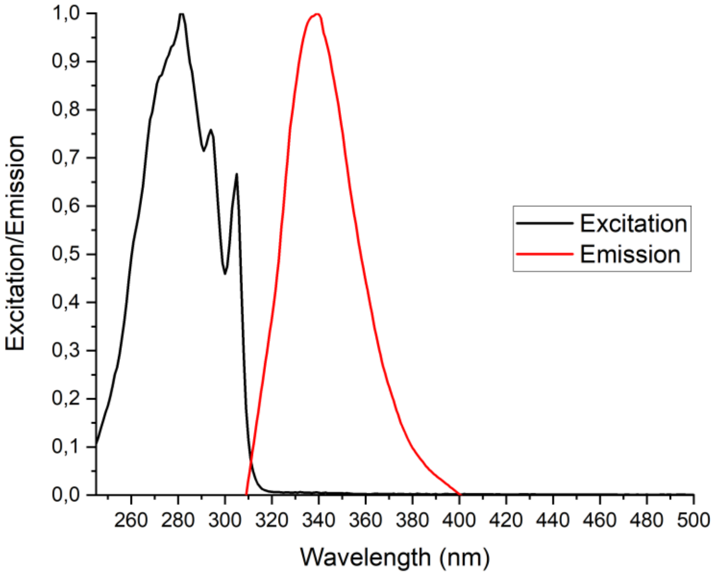

Figure S101: Excitation and emission spectra of Smoc-D-Ala-OH **4**, excitation and emission have been normalized between 0 and 1 for illustration.

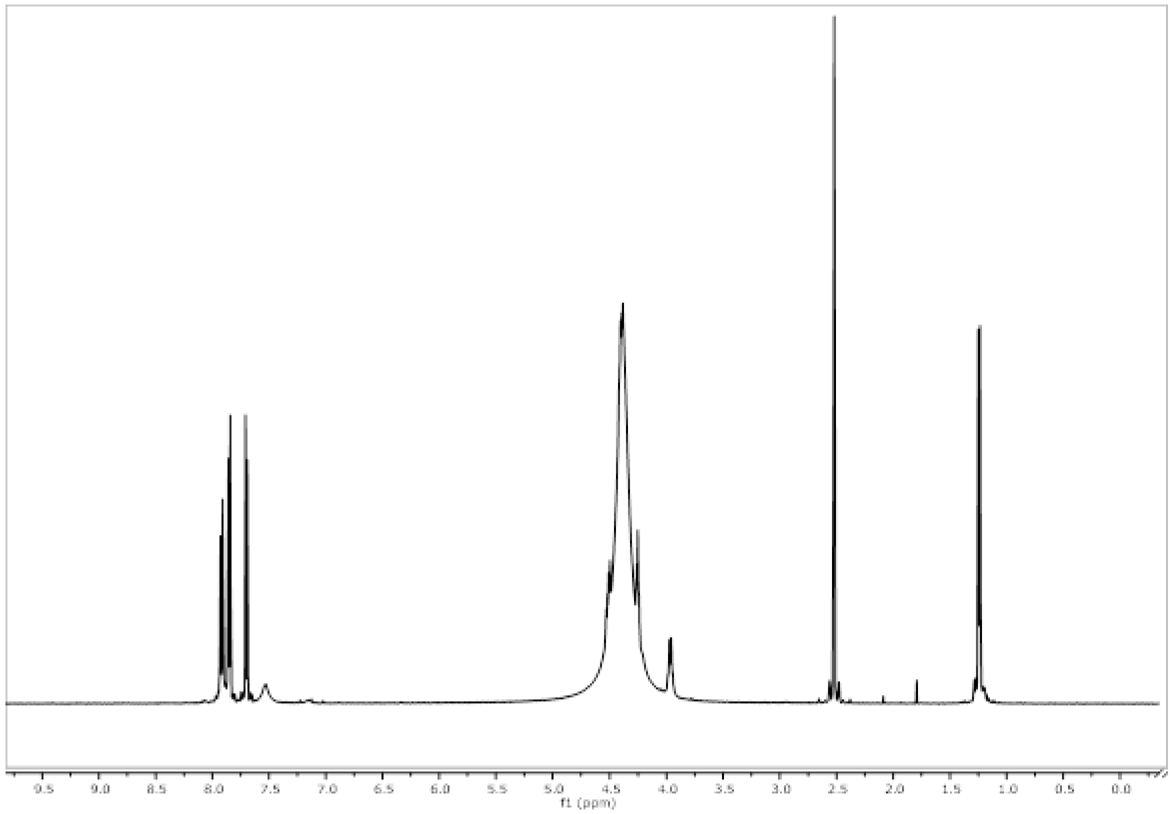

Figure S102: ^{1}H-NMR of Smoc-D-Ala-OH **4**.

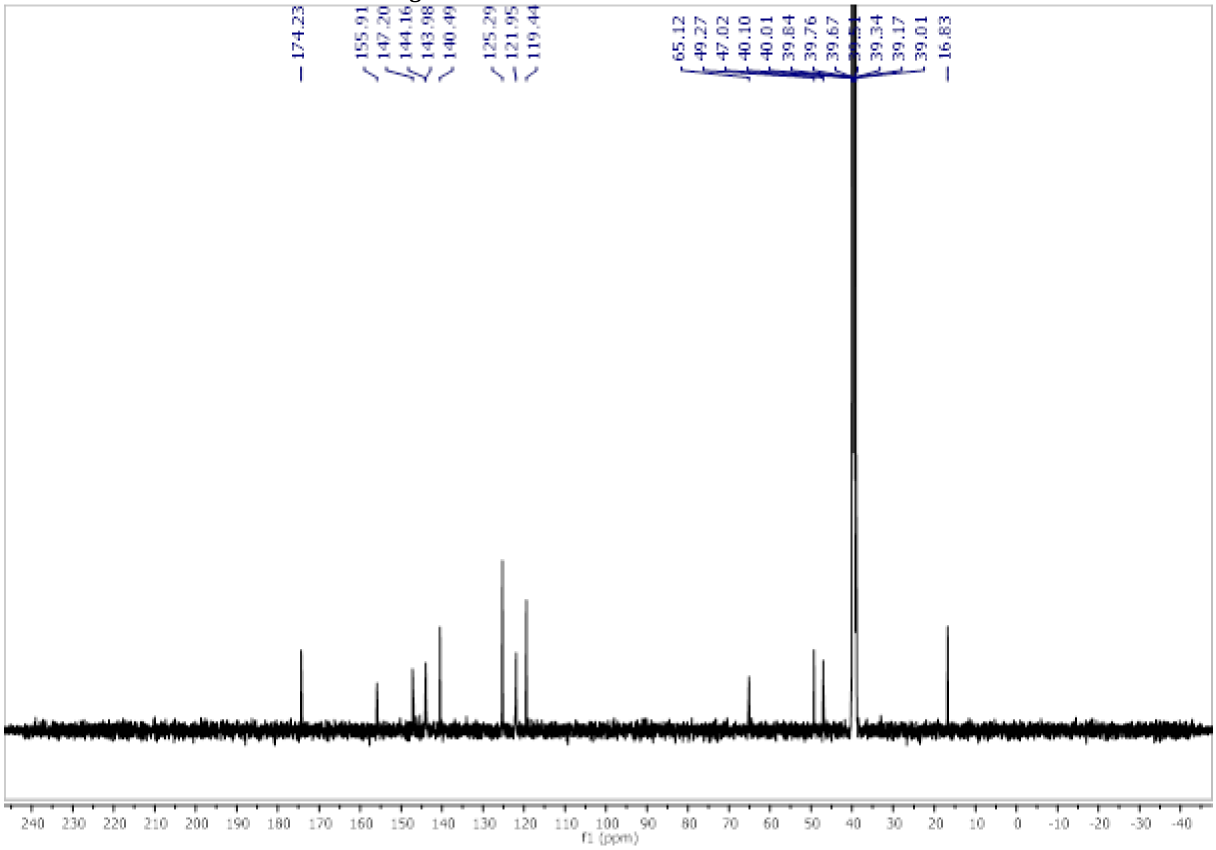

Figure S103: ^{13}C-NMR of Smoc-D-Ala-OH **4**.

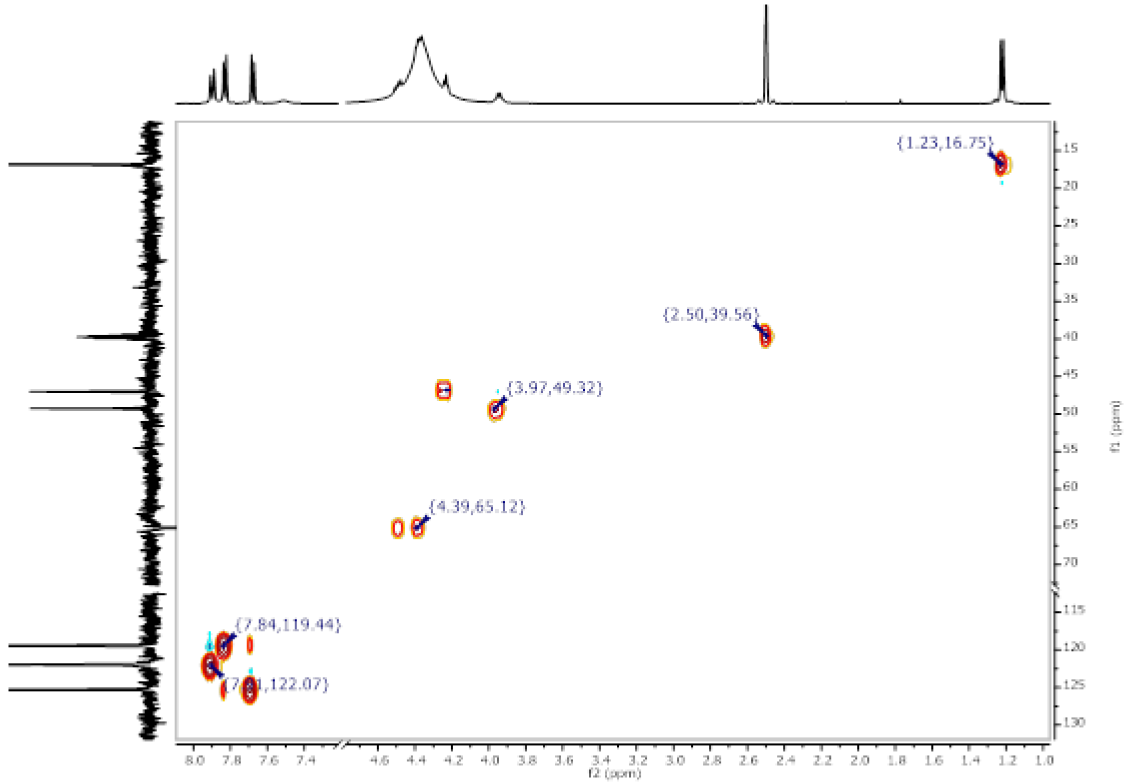

Figure S104: ^{1}H-^{13}C HSQC-NMR of Smoc-D-Ala-OH **4**.

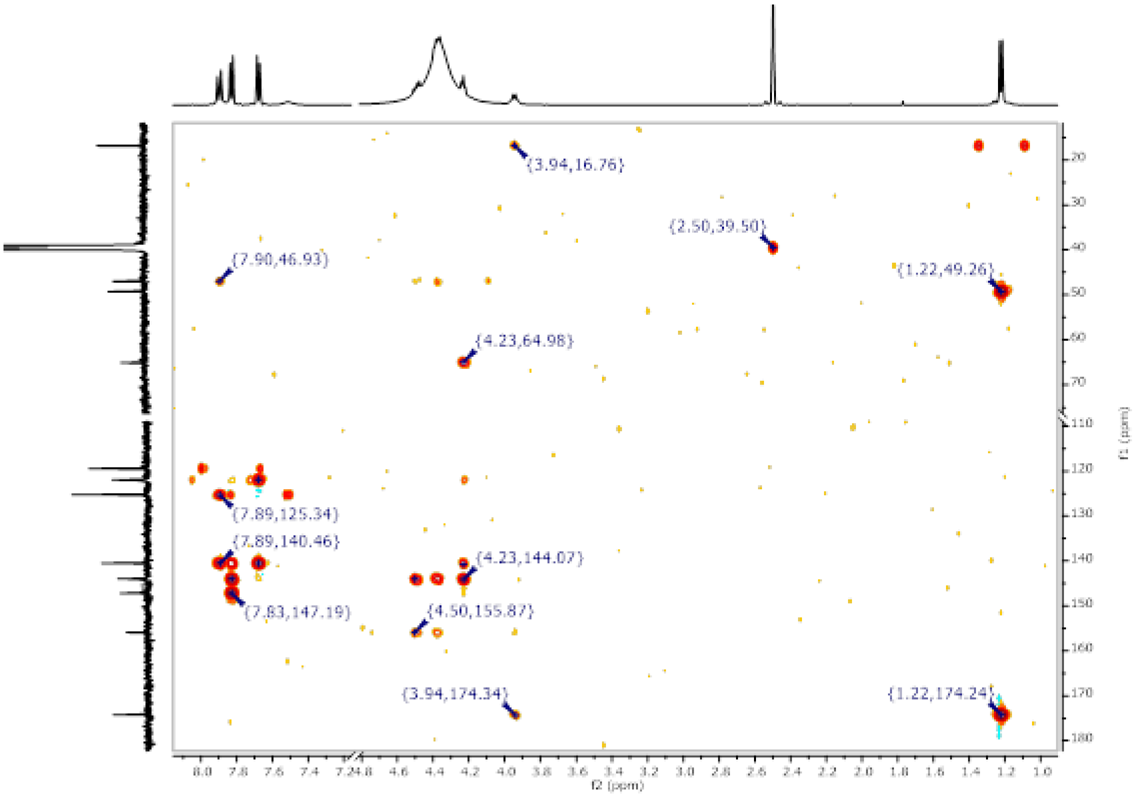

Figure S105: ^{1}H-^{13}C HMBC-NMR of Smoc-D-Ala-OH **4**.

3.2.3. Analytical data of Smoc-L-Arg-OH 5

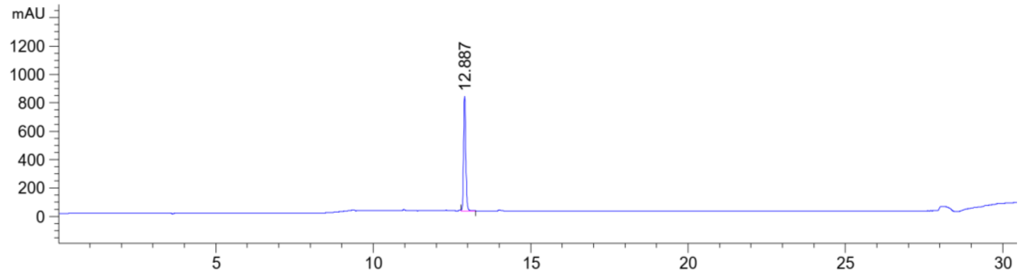

Figure S106: HPLC chromatogram of Smoc-L-Arg-OH **5** at λ=220 nm (0to40 MeCN).

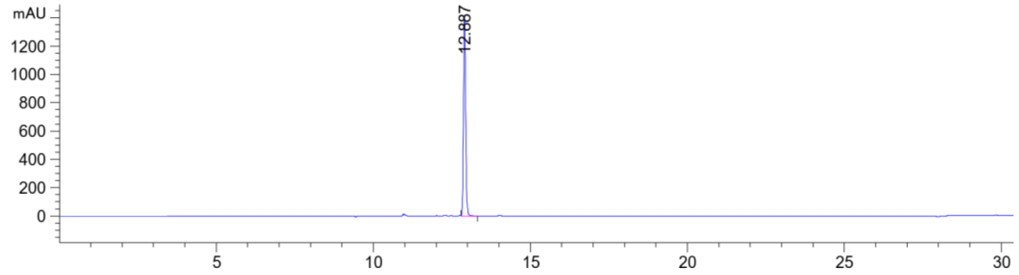

Figure S107: HPLC chromatogram of Smoc-L-Arg-OH **5** at λ=280 nm (0to40 MeCN).

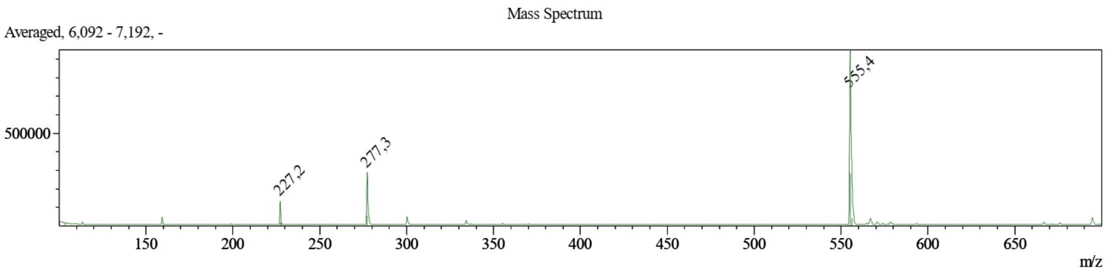

Figure S108: ESI-MS of Smoc-L-Arg-OH **5** (M measured=555.40 [M-H]$^-$, M calc.=556.56).

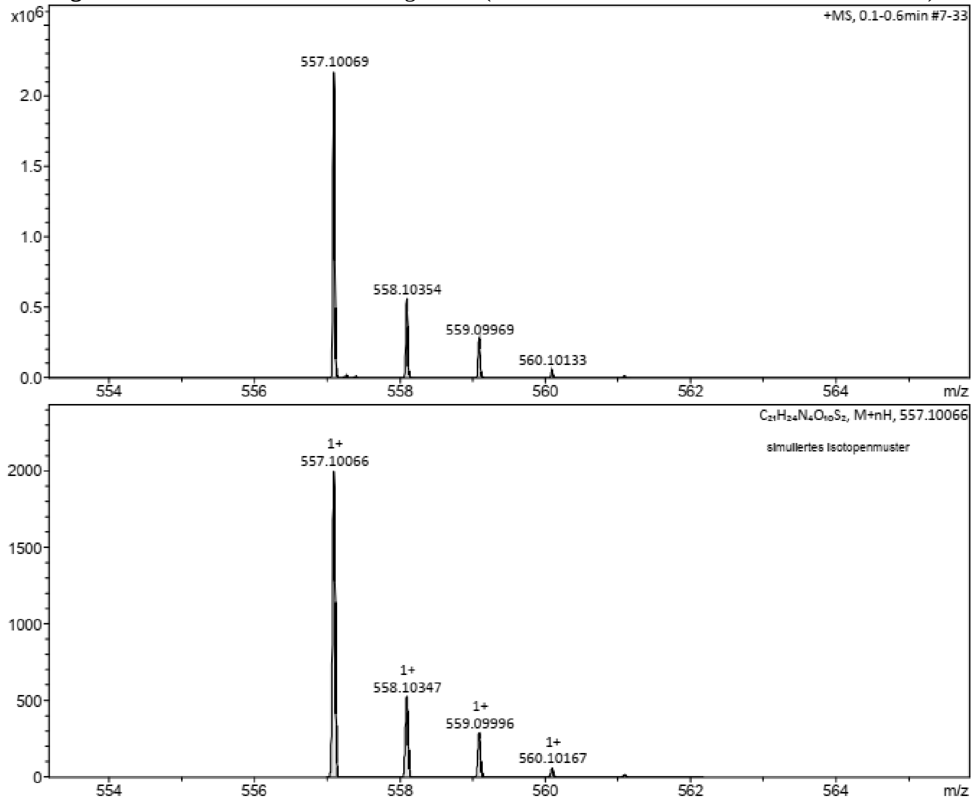

Figure S109: HR-MS of Smoc-L-Arg-OH **5** (M measured=557.10069 [M+H]$^+$, M calc.=557.010066).

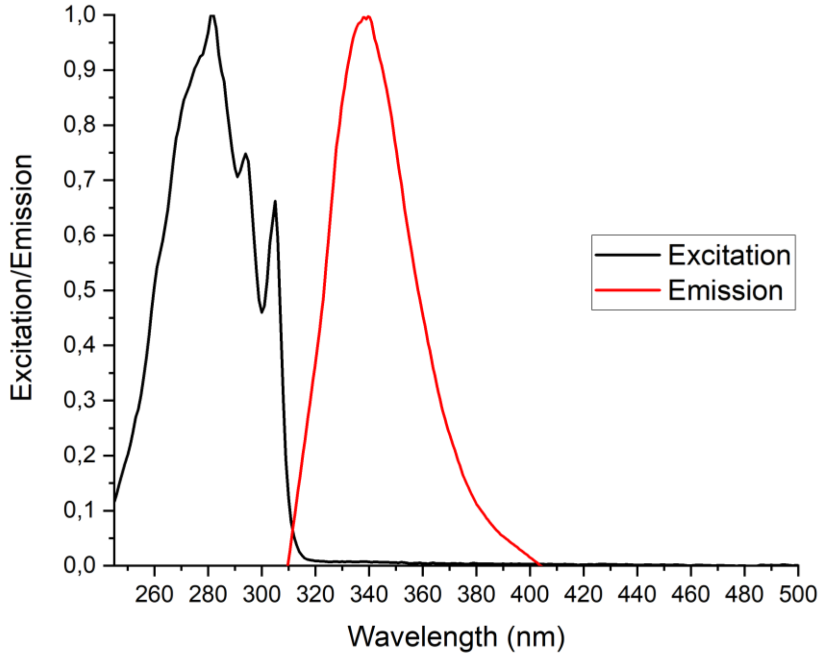

Figure S110: Excitation and emission spectra of Smoc-L-Arg-OH **5**, excitation and emission have been normalized between 0 and 1 for illustration.

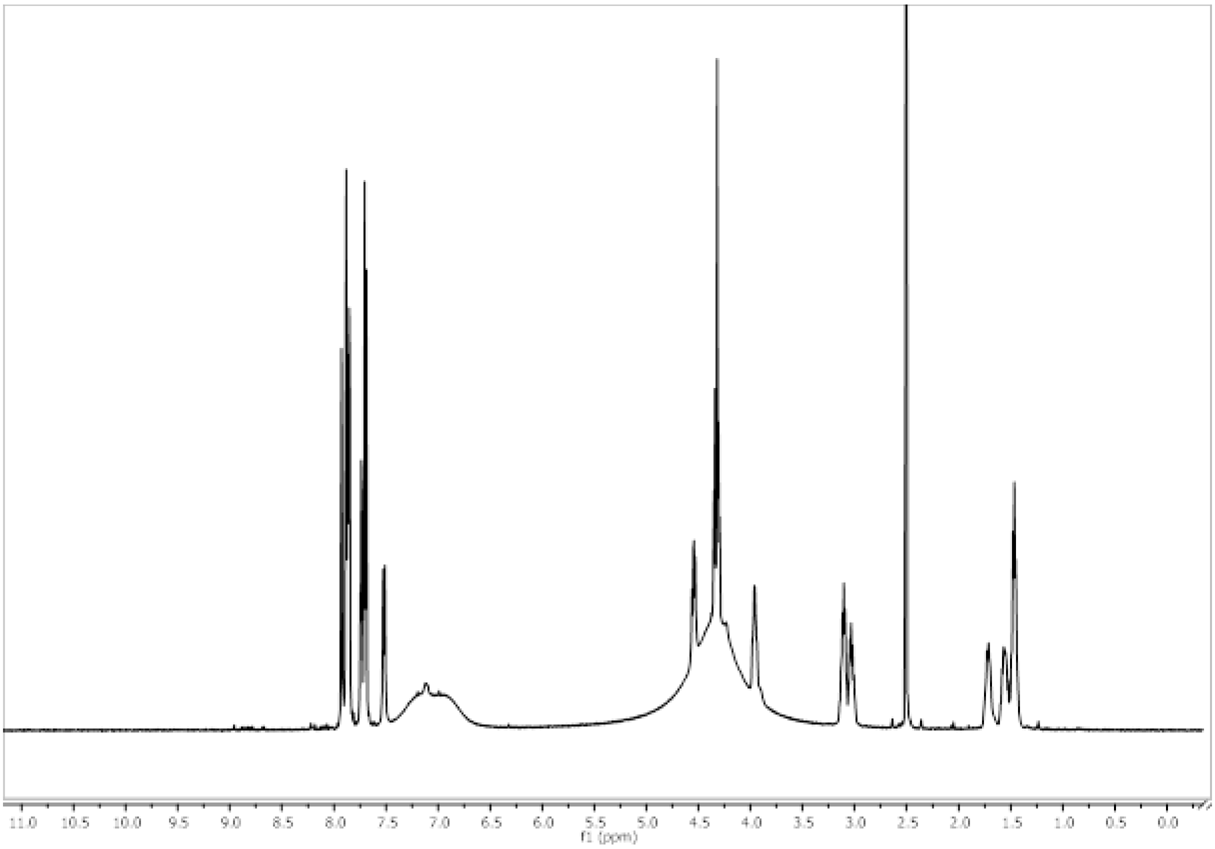

Figure S111: ^{1}H-NMR of Smoc-L-Arg-OH **5**.

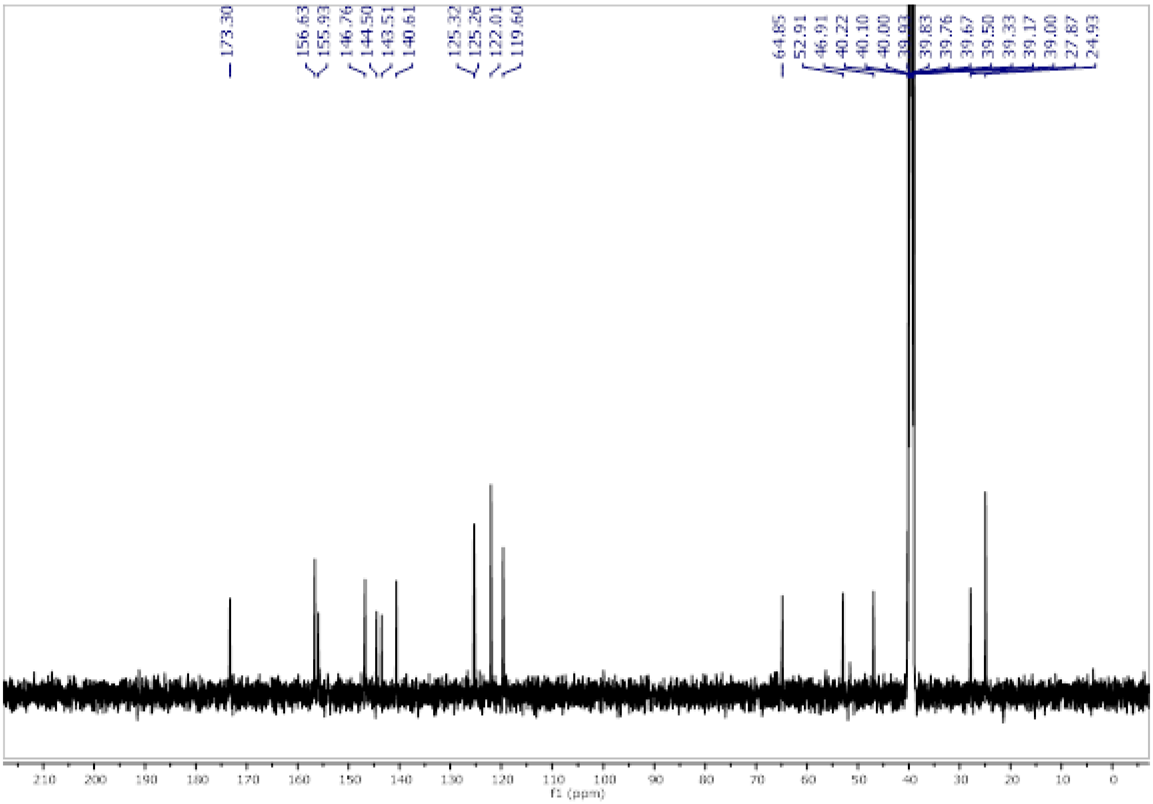

Figure S112: ^{13}C-NMR of Smoc-L-Arg-OH **5**.

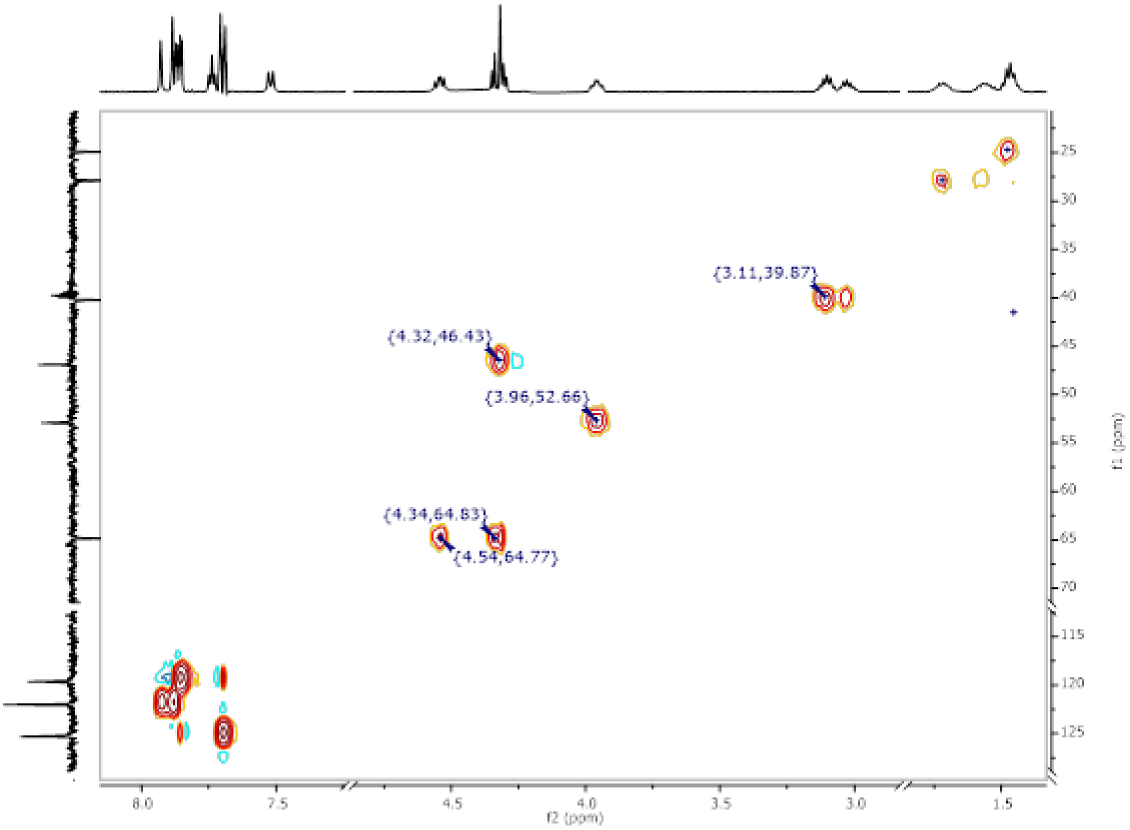

Figure S113: ^{1}H-^{13}C HSQC-NMR of Smoc-L-Arg-OH **5**.

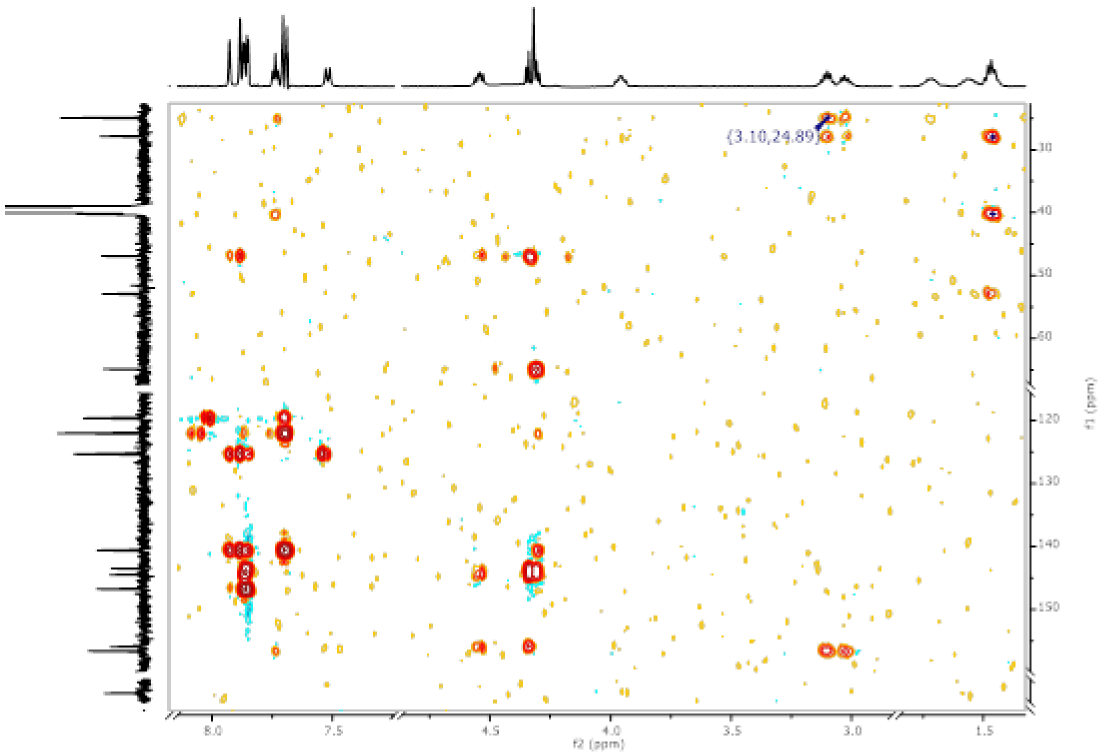

Figure S114: ^{1}H-^{13}C HMBC-NMR of Smoc-L-Arg-OH **5**.

3.2.4. Analytical data of Smoc-L-Arg(Pbf)-OH 6

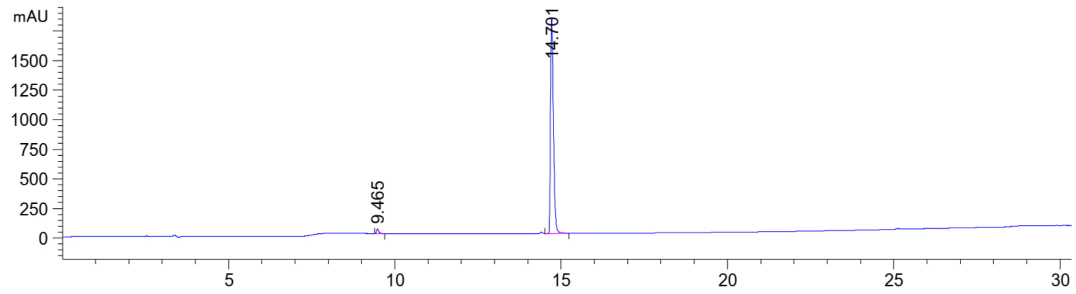

Figure S115: HPLC chromatogram of Smoc-L-Arg(Pbf)-OH **6** at λ=220 nm (10to100 MeCN).

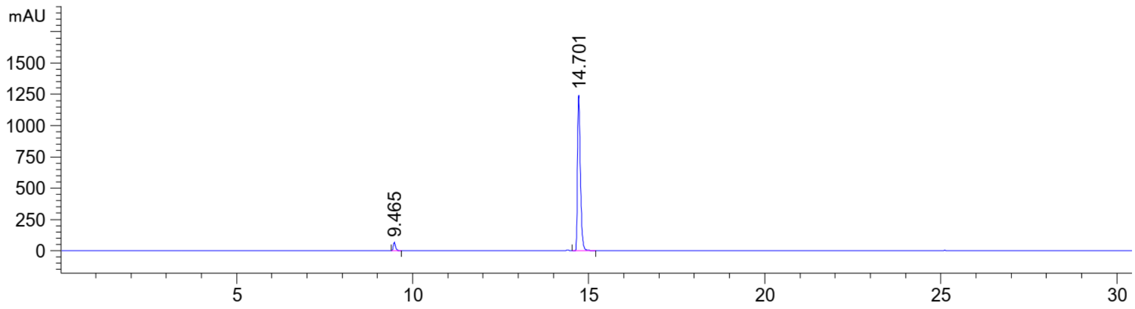

Figure S116: HPLC chromatogram of Smoc-L-Arg(Pbf)-OH **6** at λ=280 nm (10to100 MeCN).

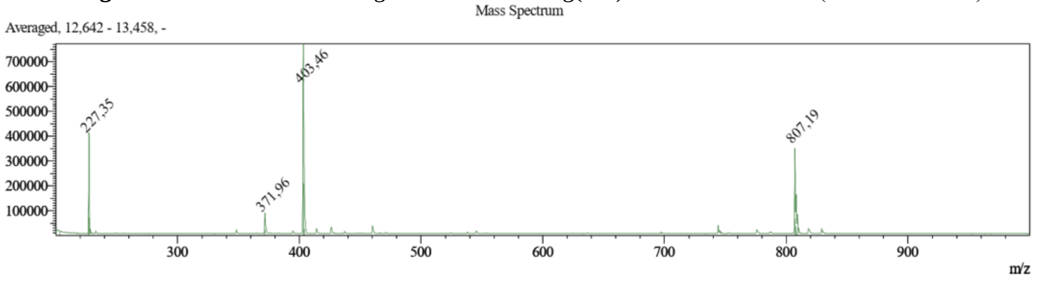

Figure S117: ESI-MS of Smoc-L-Arg(Pbf)-OH **6** (M measured=807.19 [M-H]$^-$, M calc.=808.89).

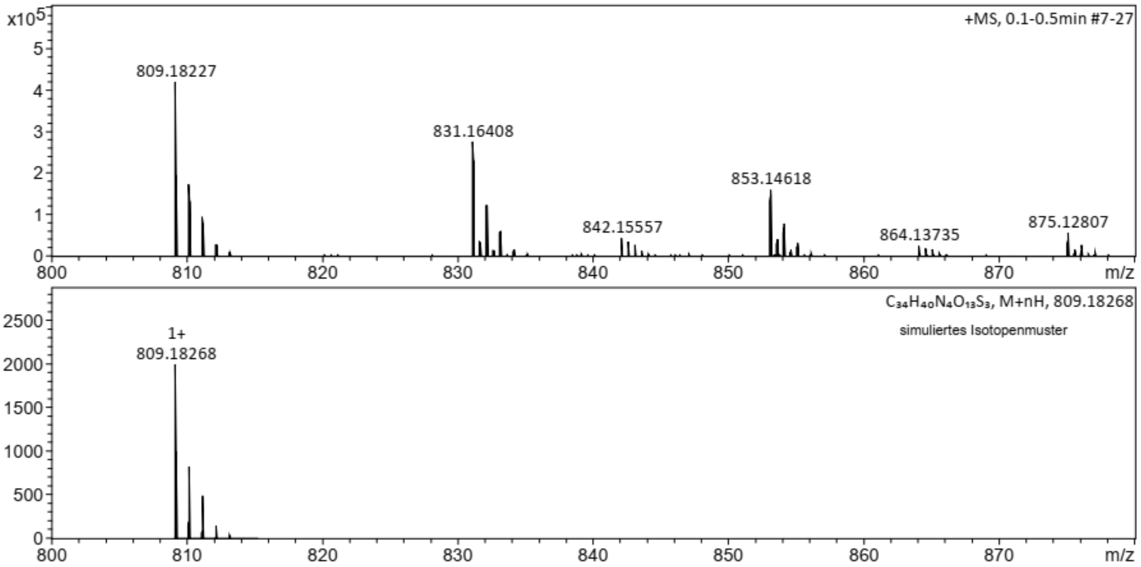

Figure S118: HR-MS of Smoc-L-Arg(Pbf)-OH **6** (M measured=809.18227 [M+H]$^+$, M calc.=809.18268).

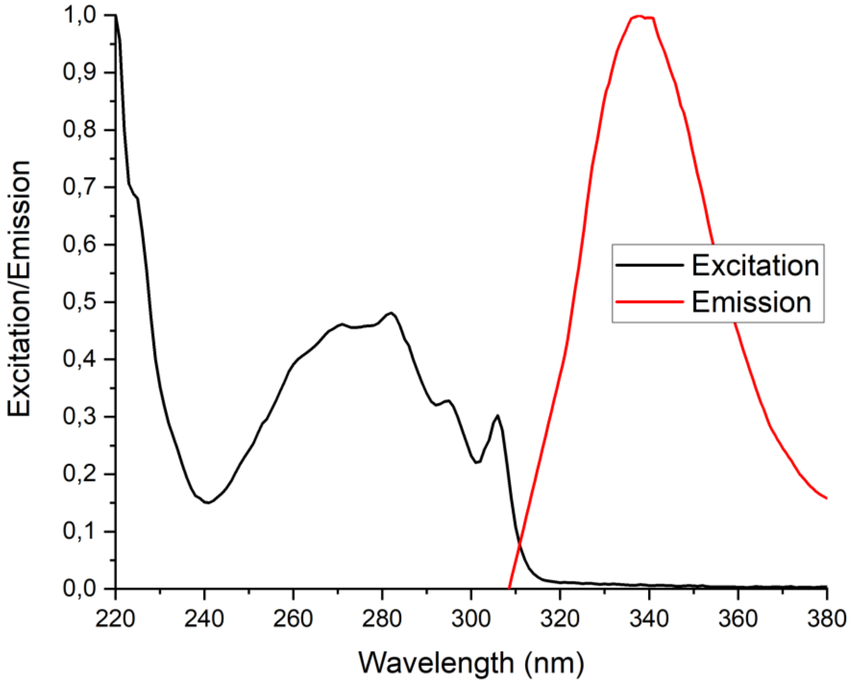

Figure S119: Excitation and emission spectra of Smoc-L-Arg(Pbf)-OH **6**, excitation and emission have been normalized between 0 and 1 for illustration.

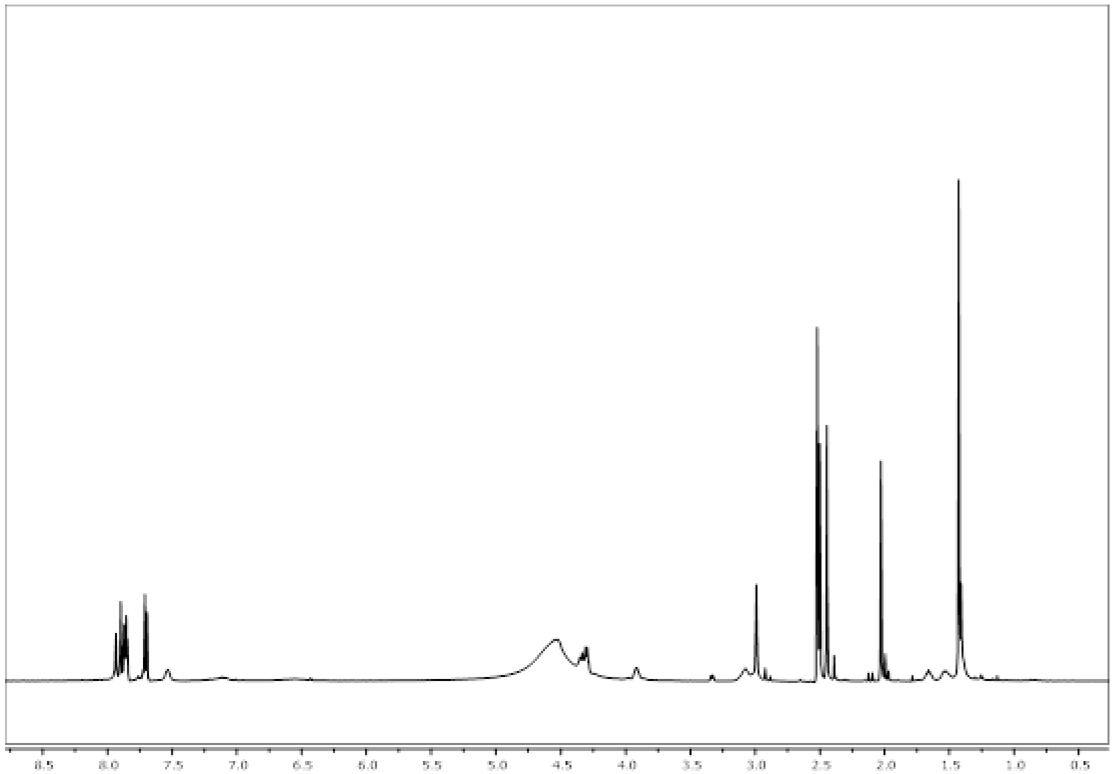

Figure S120: ^{1}H-NMR of Smoc-L-Arg(Pbf)-OH **6**.

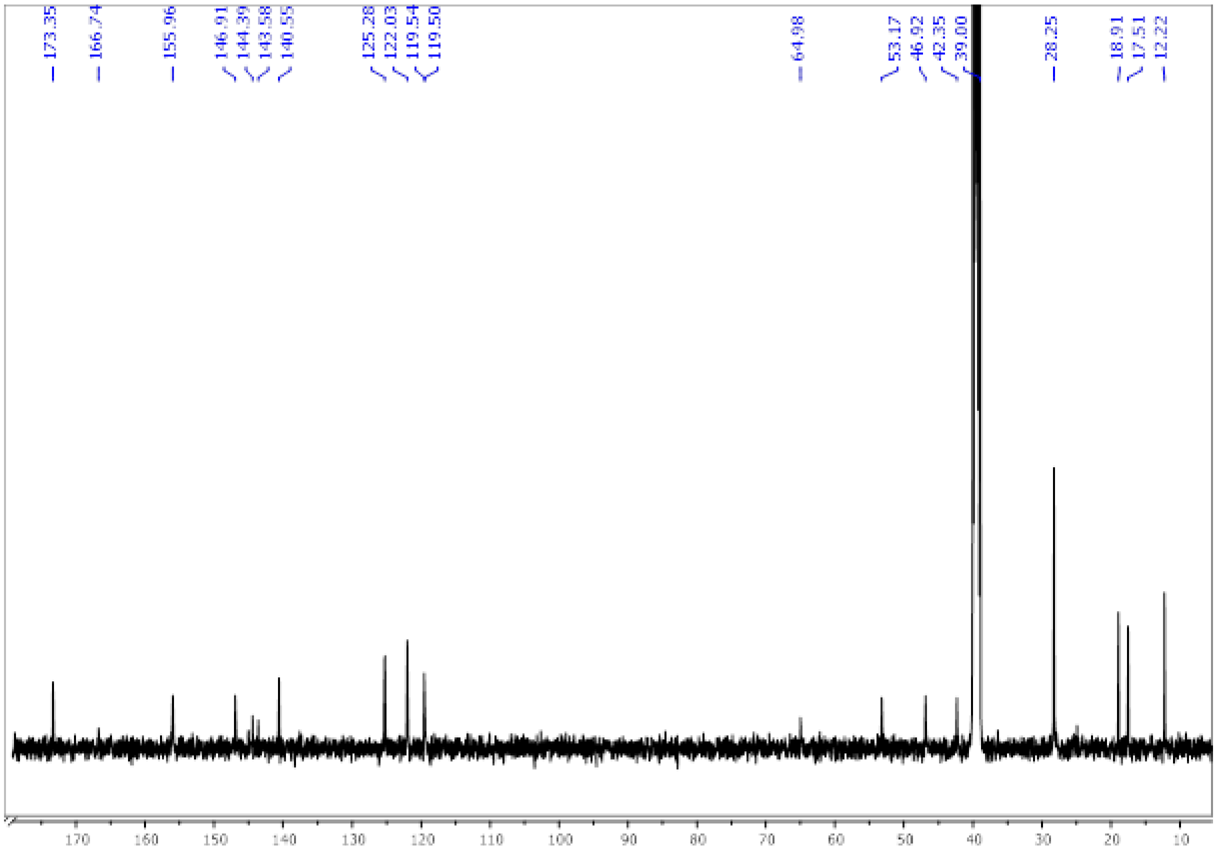

Figure S121: ^{13}C-NMR of Smoc-L-Arg(Pbf)-OH **6**.

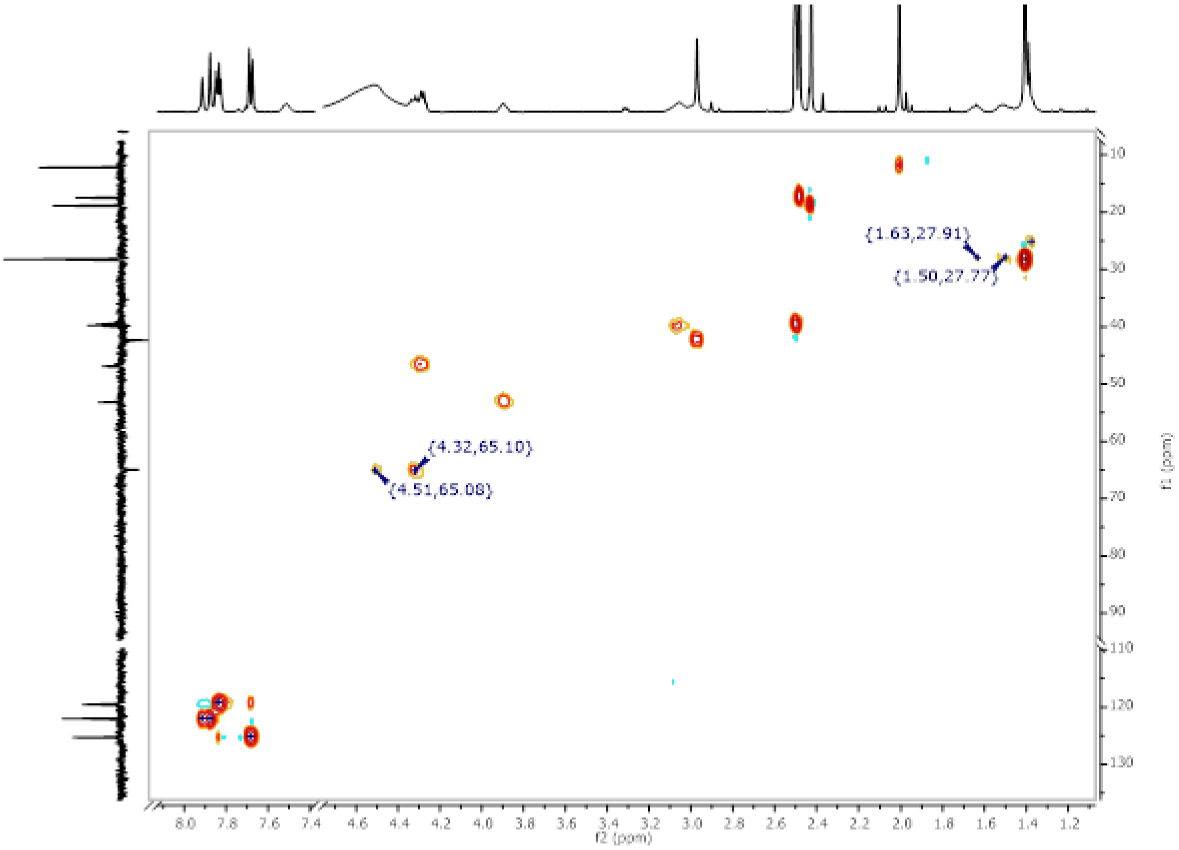

Figure S122: ^{1}H-^{13}C HSQC-NMR of Smoc-L-Arg(Pbf)-OH **6**.

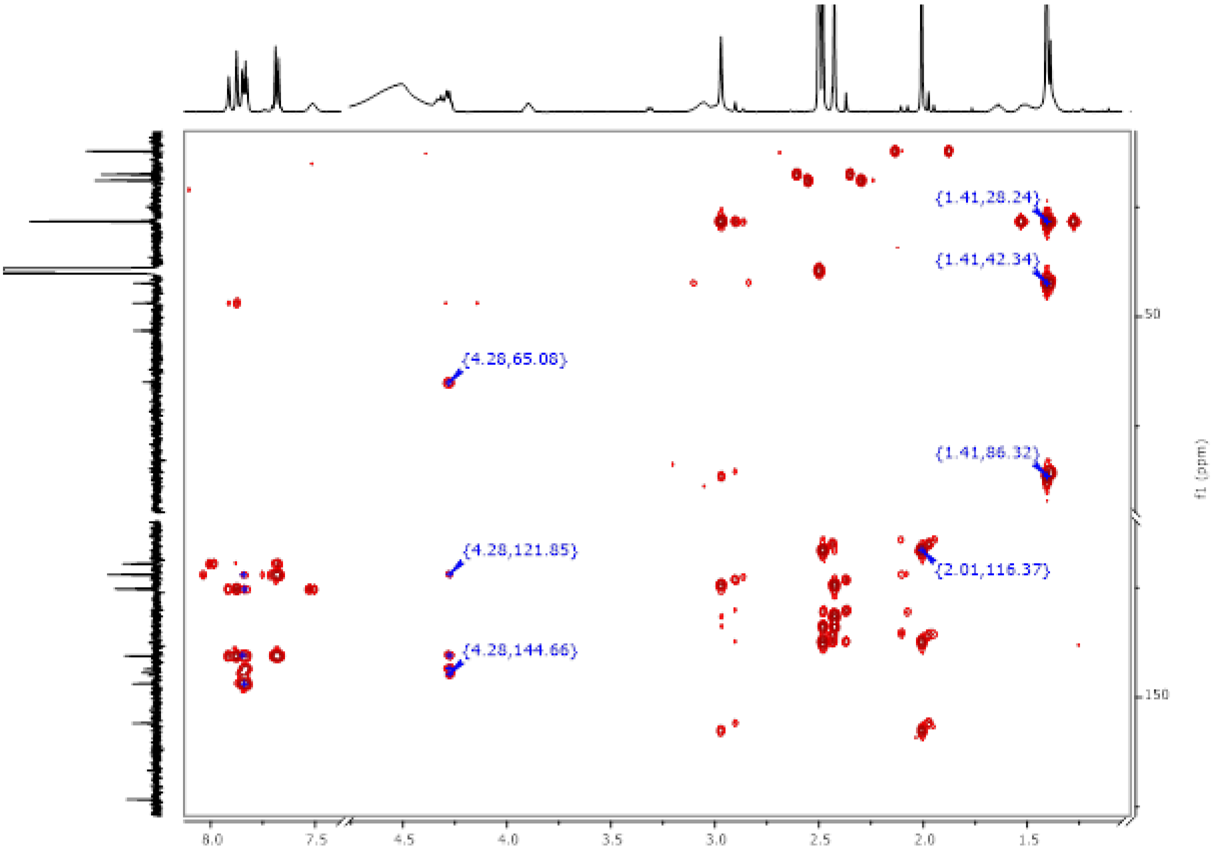

Figure S123: ^{1}H-^{13}C HMBC-NMR of Smoc-L-Arg(Pbf)-OH **6**.

3.2.5. Analytical data of Smoc-L-Asn-OH 7

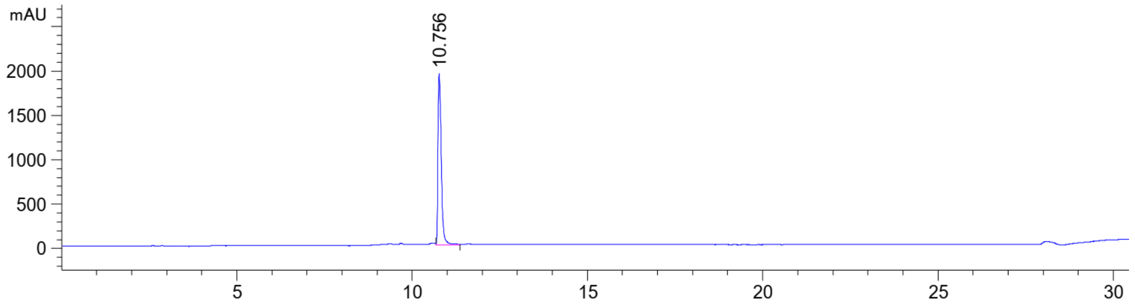

Figure S124: HPLC chromatogram of Smoc-L-Asn-OH **7** at λ=220 nm (0to40 MeCN).

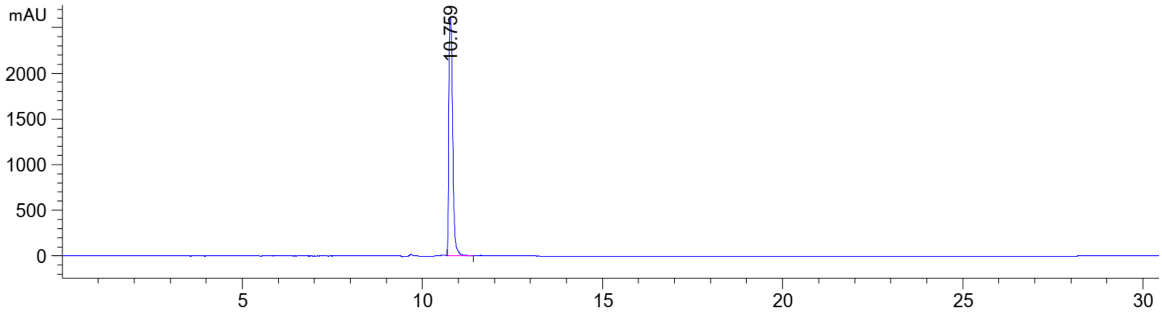

Figure S125: HPLC chromatogram of Smoc-L-Asn-OH **7** at λ=280 nm (0to40 MeCN).

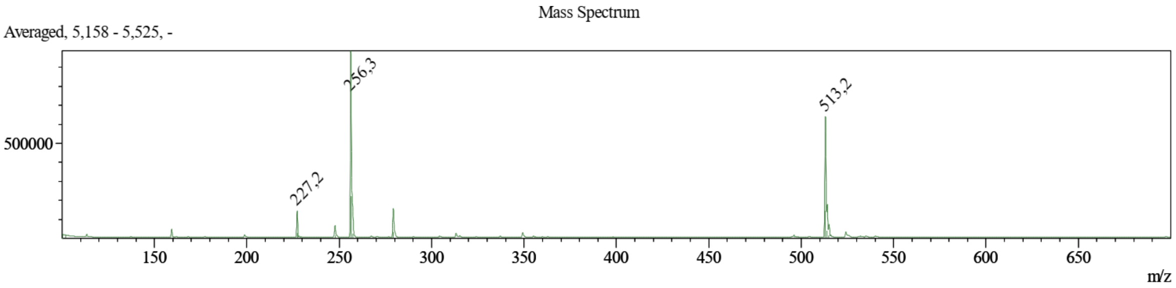

Figure S126: ESI-MS of Smoc-L-Asn-OH **7** (M measured=513.20 $[M-H]^-$, M calc.=514.48).

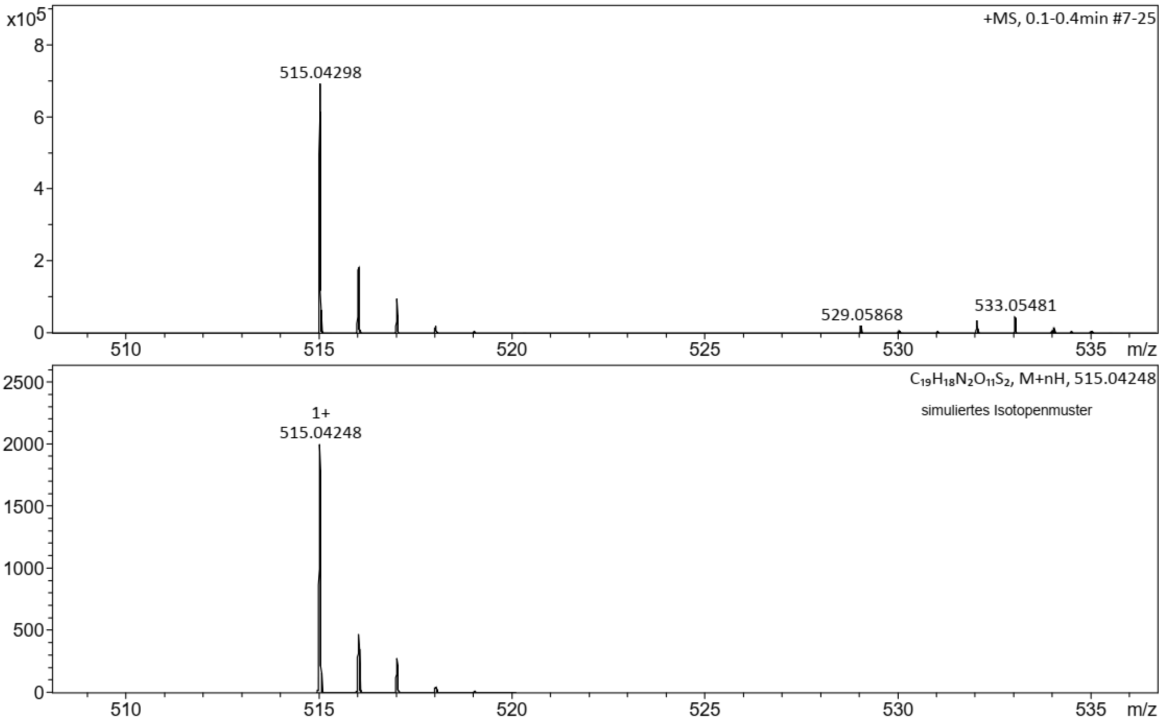

Figure S127: HR-MS of Smoc-L-Asn-OH **7** (M measured=515.04298 $[M+H]^+$, M calc.=515.04248).

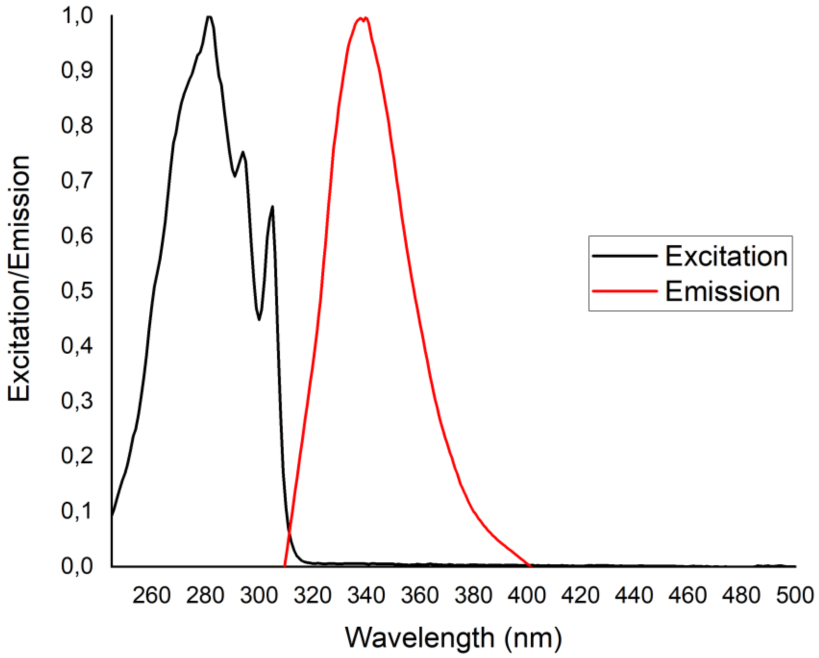

Figure S128: Excitation and emission spectra of Smoc-L-Asn-OH **7**, excitation and emission have been normalized between 0 and 1 for illustration.

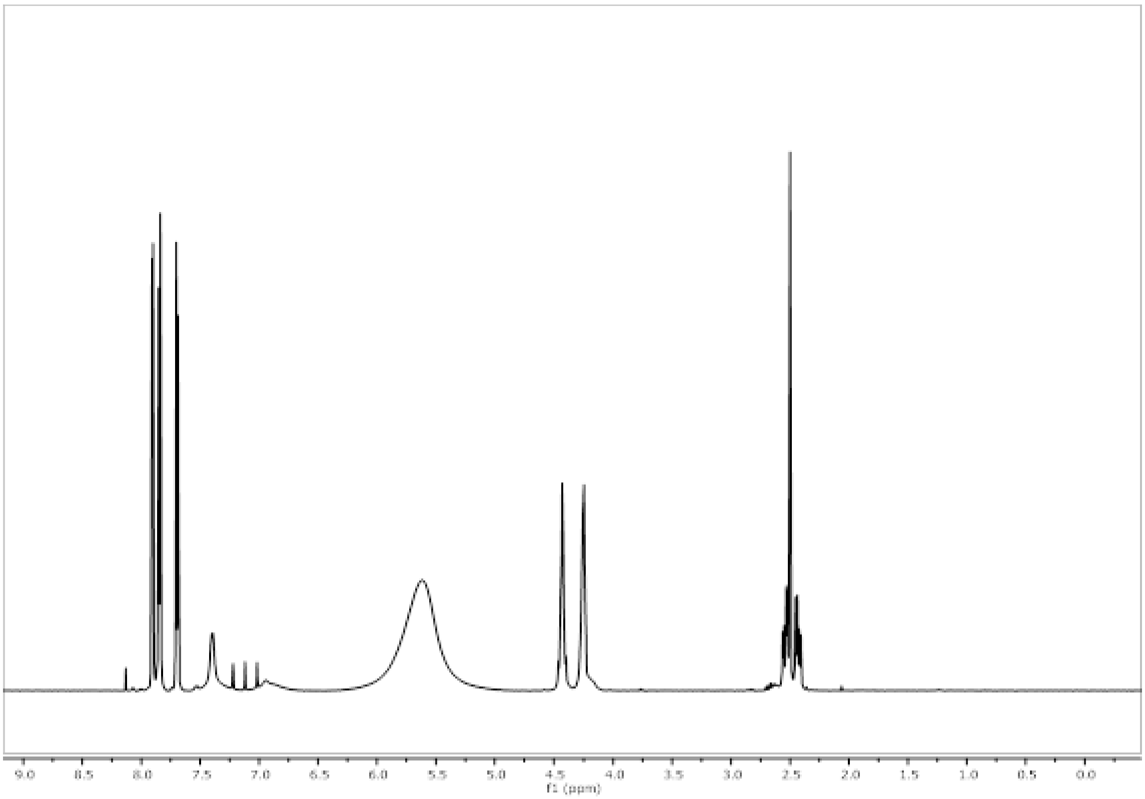

Figure S129: ^{1}H-NMR of Smoc-L-Asn-OH **7**.

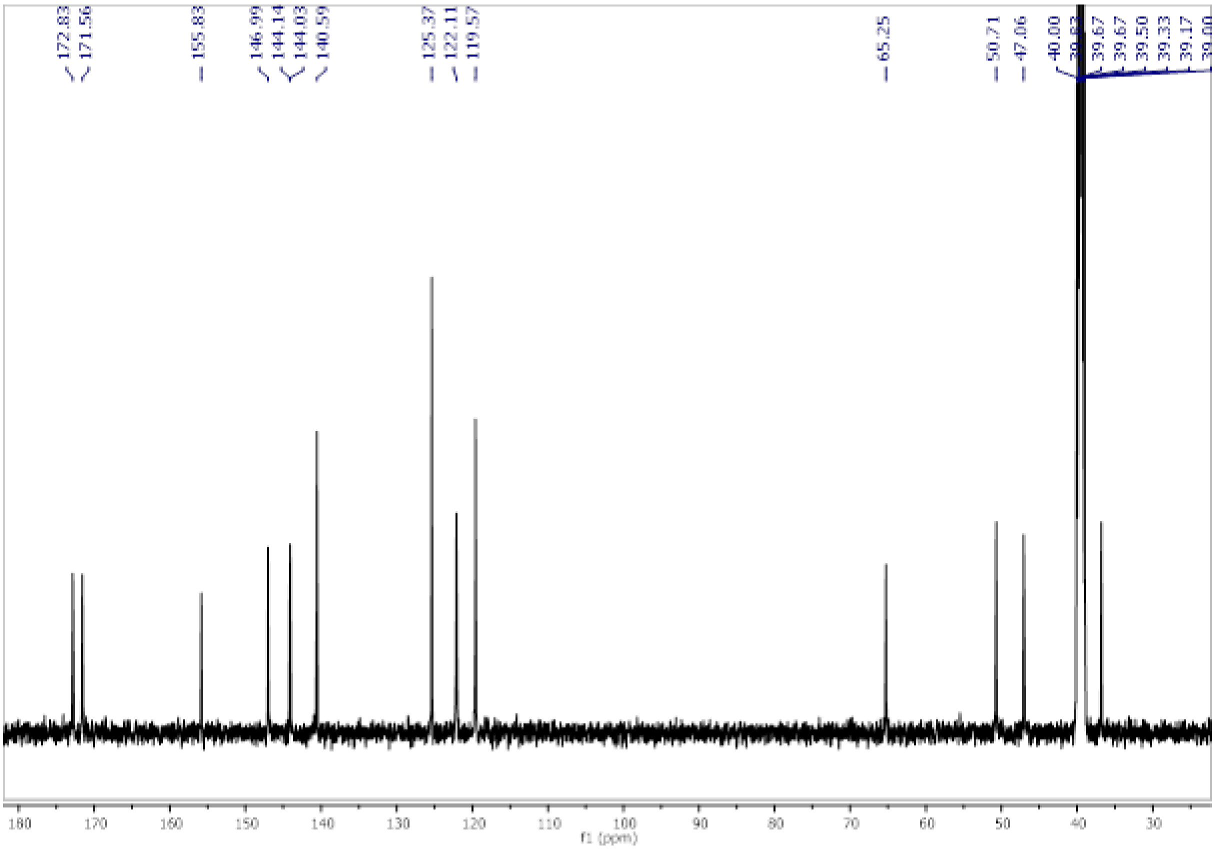

Figure S130: ^{13}C-NMR of Smoc-L-Asn-OH **7**.

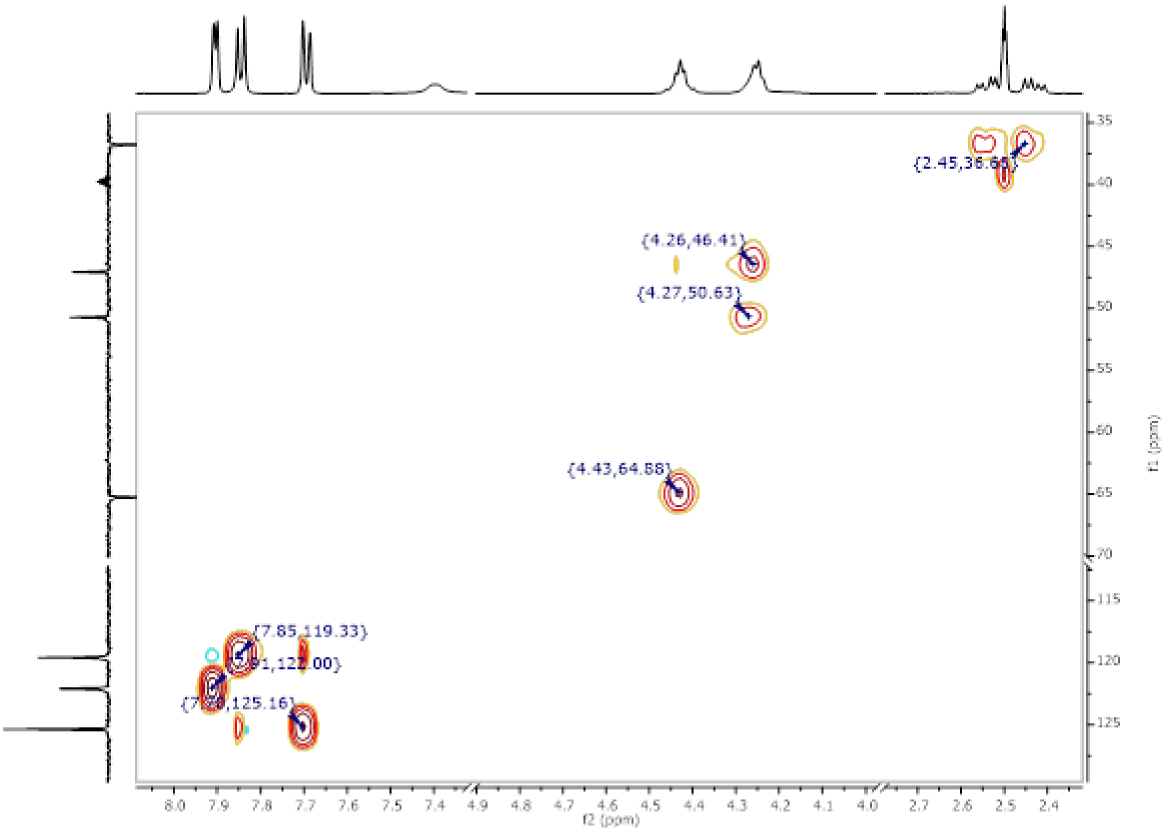

Figure S131: ^{1}H-^{13}C HSQC-NMR of Smoc-L-Asn-OH **7**.

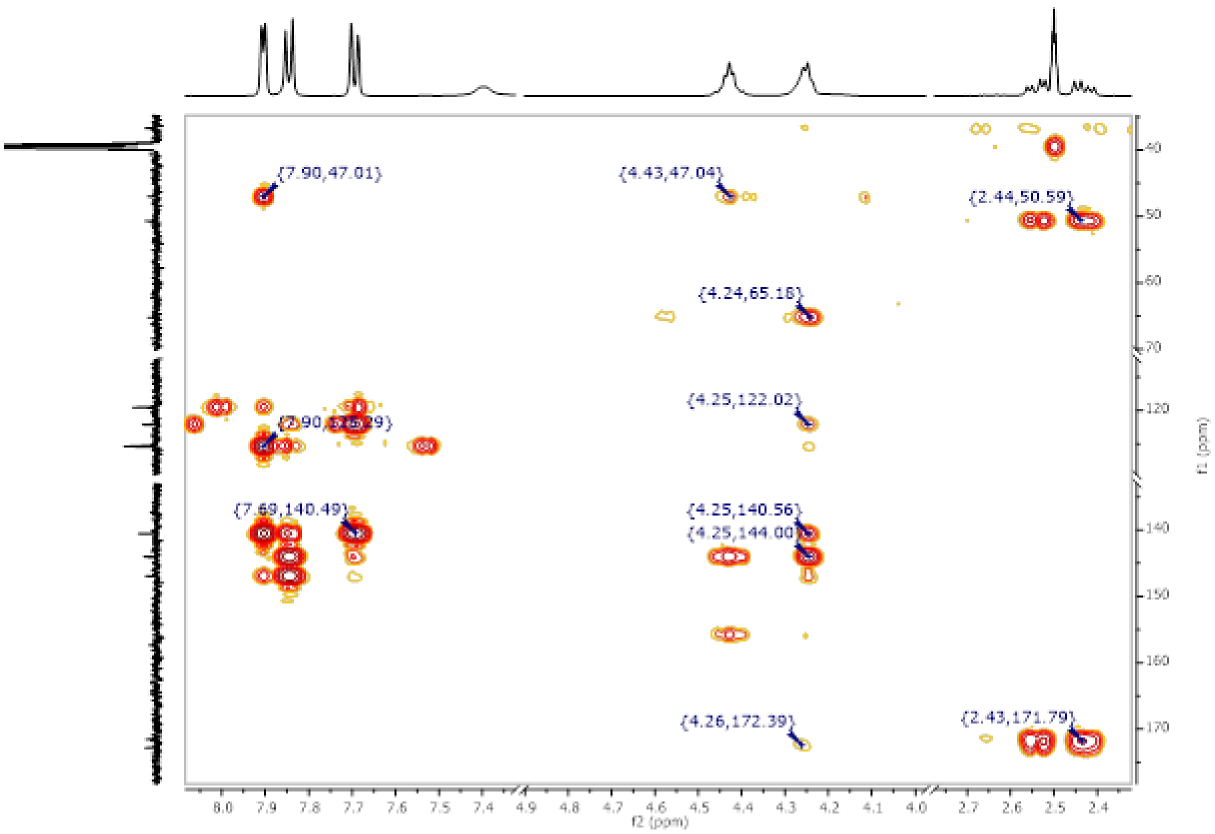

Figure S132: ^{1}H-^{13}C HMBC-NMR of Smoc-L-Asn-OH **7**.

3.2.6. Analytical data of Smoc-L-Asp(OtBu)-OH 8

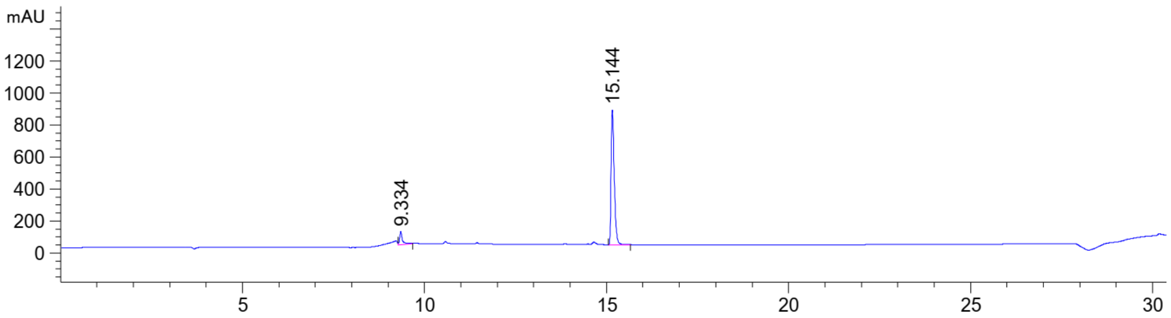

Figure S133: HPLC chromatogram of Smoc-L-Asp(OtBu)-OH **8** at λ=220 nm (0to60 MeCN).

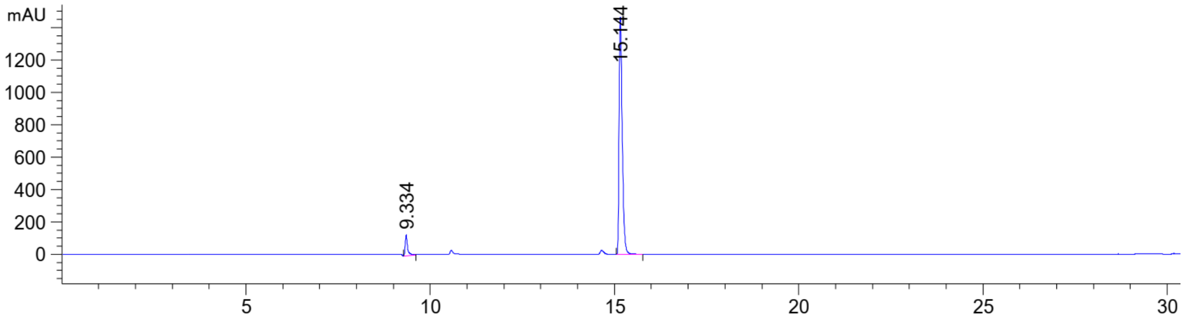

Figure S134: HPLC chromatogram of Smoc-L-Asp(OtBu)-OH **8** at λ=280 nm (0to60 MeCN).

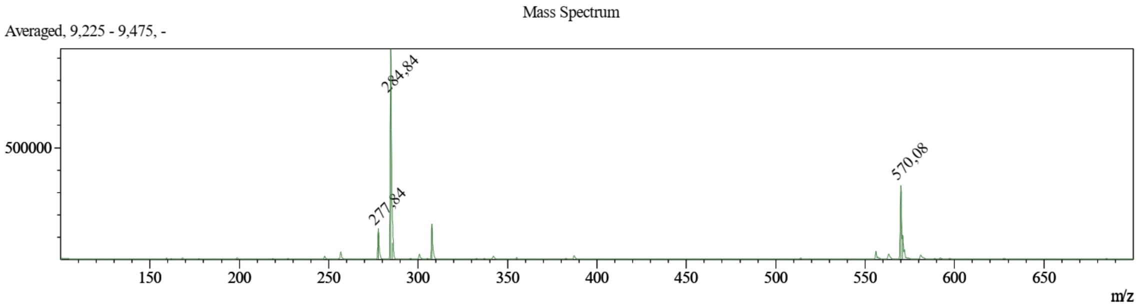

Figure S135: ESI-MS of Smoc-L-Asp(OtBu)-OH **8** (M measured=570.08 [M-H]$^-$, M calc.= 571.57).

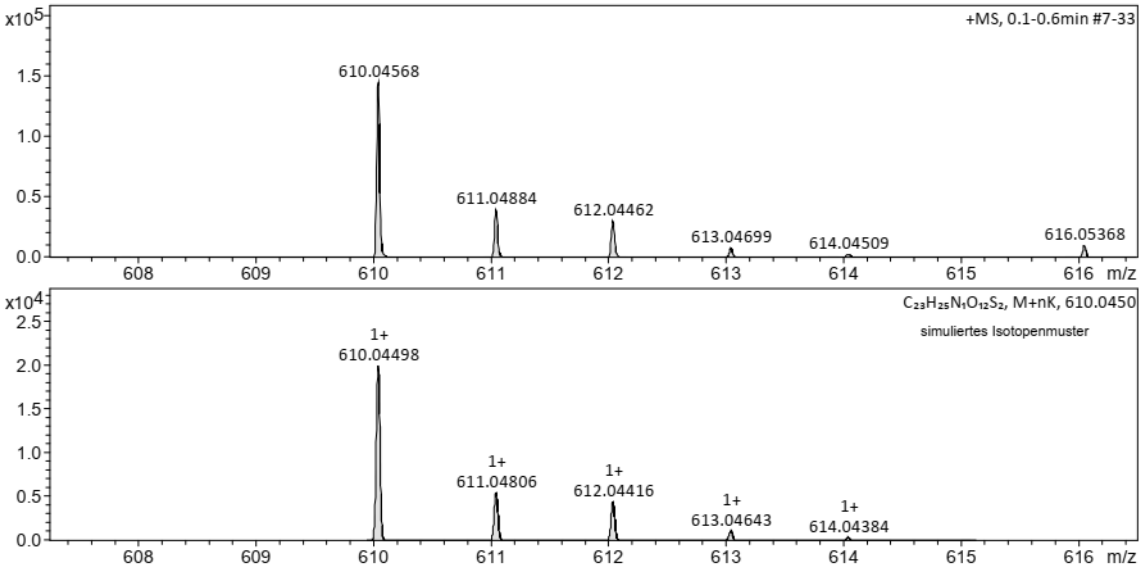

Figure S136: HR-MS of Smoc-L-Asp(OtBu)-OH **8** (M measured=610.04568 [M+H]$^+$, M calc.=610.04498).

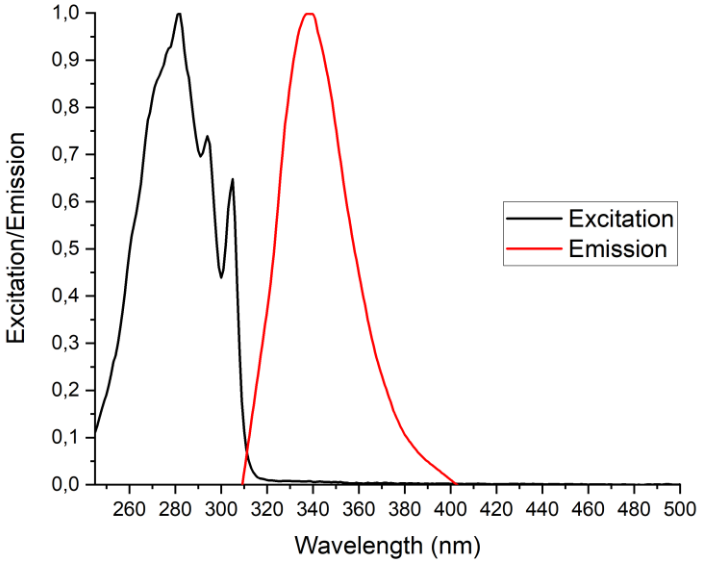

Figure S137: Excitation and emission spectra of Smoc-L-Asp(OtBu)-OH **8**, excitation and emission have been normalized between 0 and 1 for illustration.

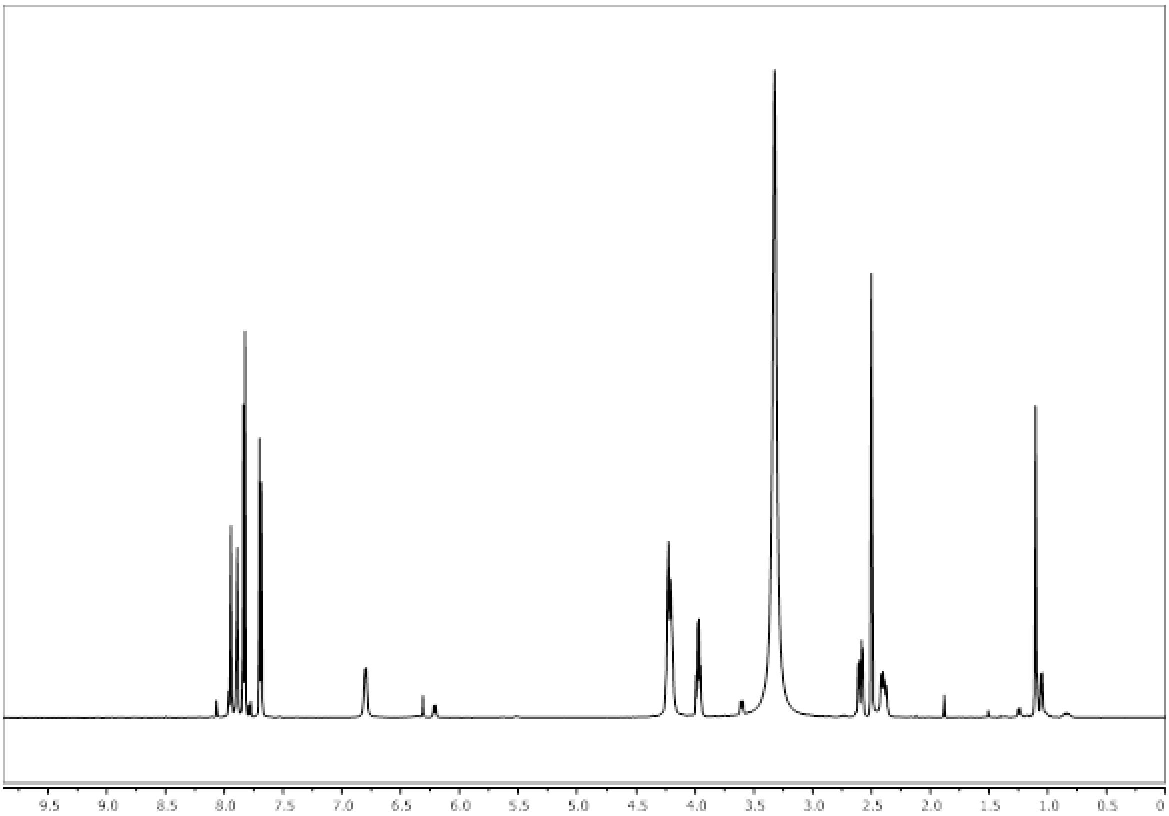

Figure S138: ^{1}H-NMR of Smoc-L-Asp(OtBu)-OH **8**.

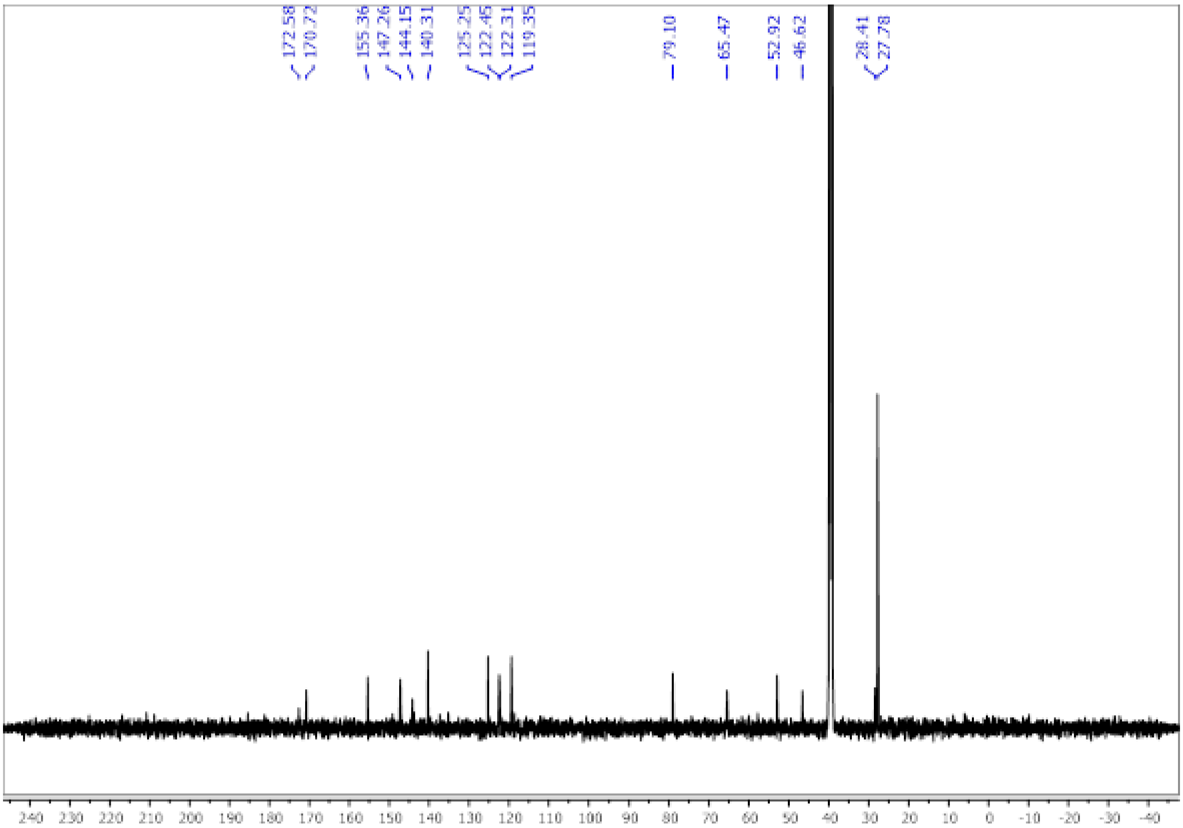

Figure S139: ^{13}C-NMR of Smoc-L-Asp(OtBu)-OH **8**.

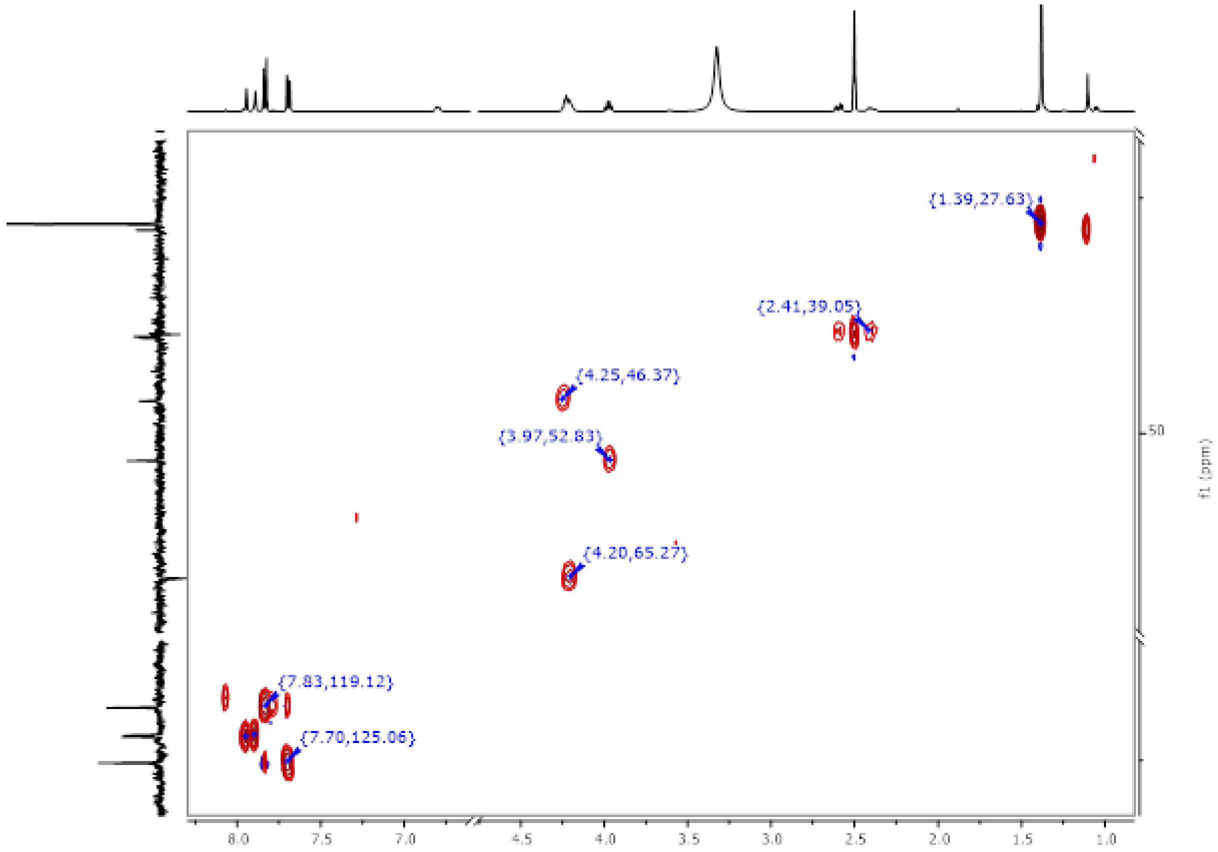

Figure S140: ^{1}H-^{13}C HSQC-NMR of S

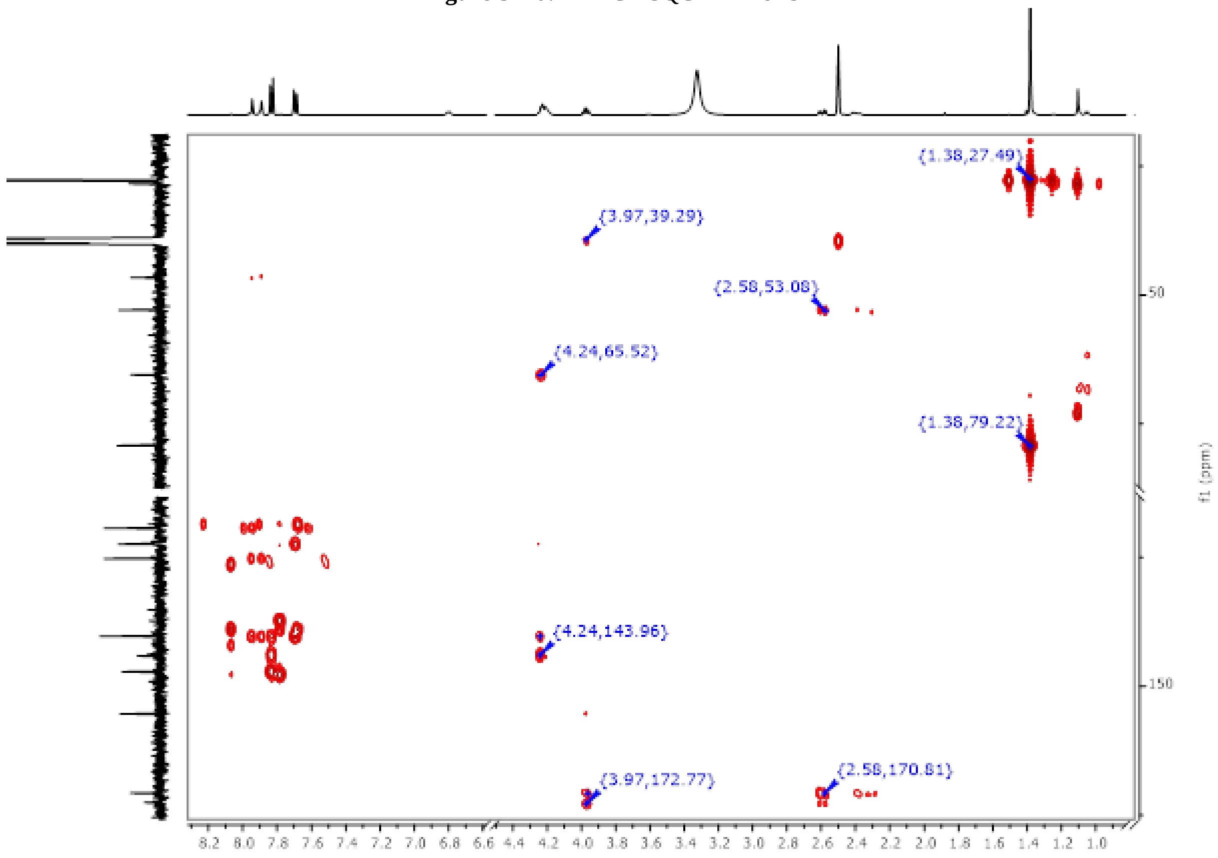

moc-L-Asp(OtBu)-OH **8**.

Figure S141: ^{1}H-^{13}C HMBC-NMR of Smoc-L-Asp(OtBu)-OH **8**.

3.2.7. Analytical data of Smoc-L-Cys(Trt)-OH 9

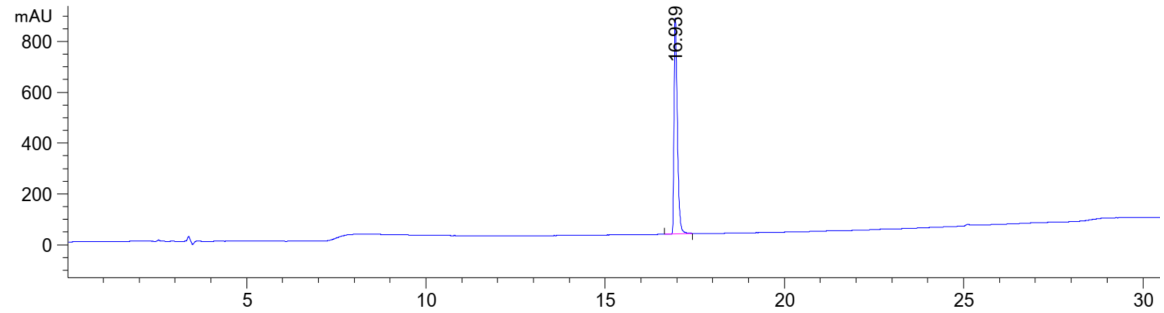

Figure S142: HPLC chromatogram of Smoc-L-Cys(Trt)-OH **9** at λ=220 nm (10to100 MeCN).

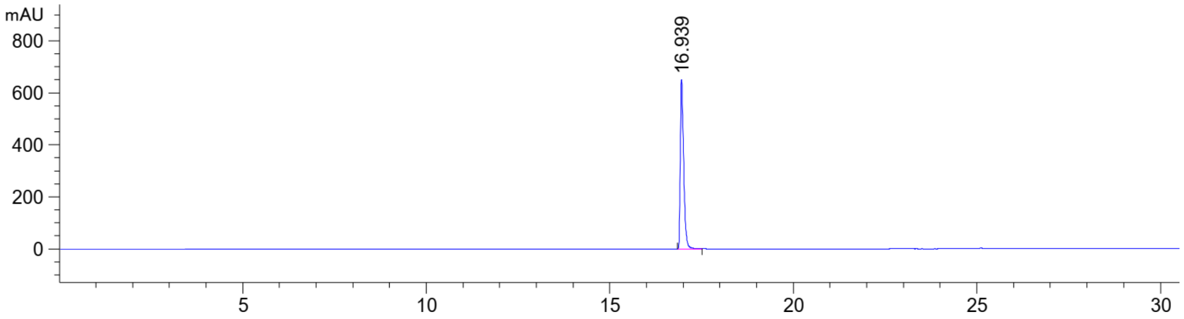

Figure S143: HPLC chromatogram of Smoc-L- Cys(Trt)-OH **9** at λ=280 nm (10to100 MeCN).

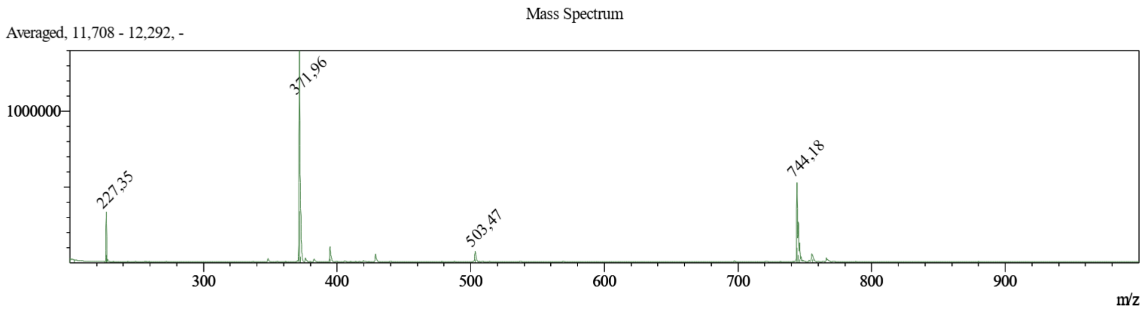

Figure S144: ESI-MS of Smoc-L- Cys(Trt)-OH **9** (M measured=744.18 [M-H]$^-$, M calc.=745.83).

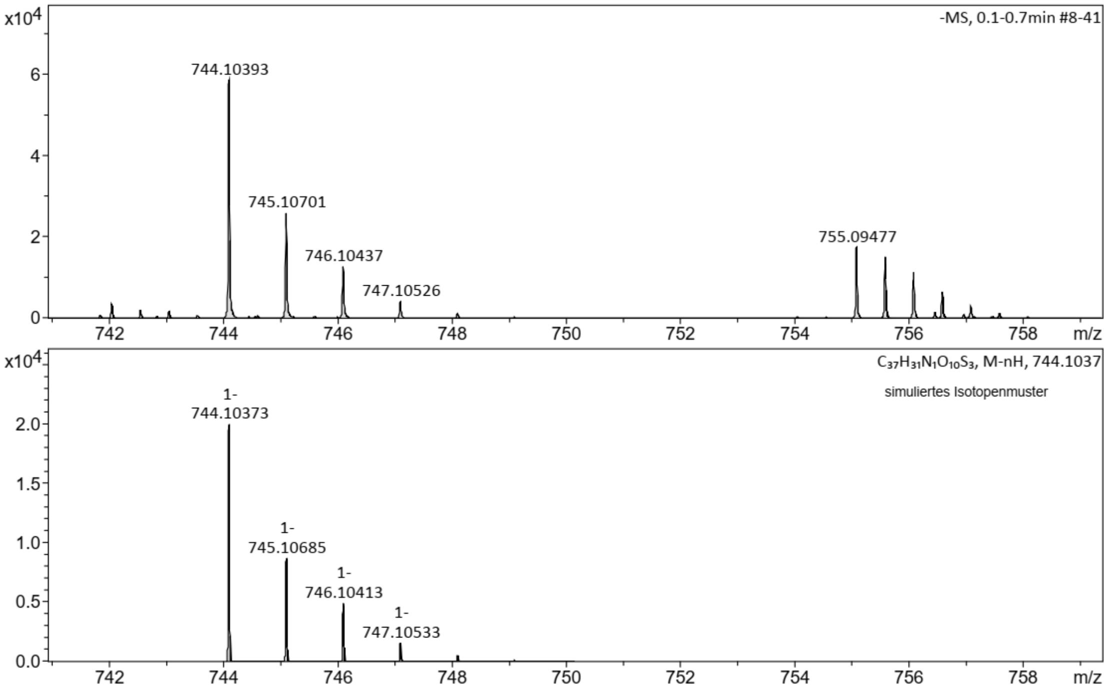

Figure S145: HR-MS of Smoc-L-Cys(Trt)-OH **9** (M measured=744.10393 [M-H]$^-$, M calc.=744.10373).

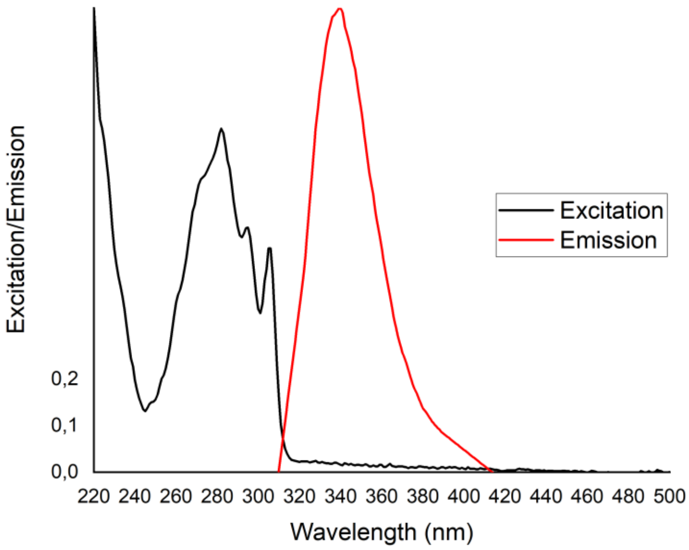

Figure S146: Excitation and emission spectra of Smoc-L-Cys(Trt)-OH **9**, excitation and emission have been normalized between 0 and 1 for illustration.

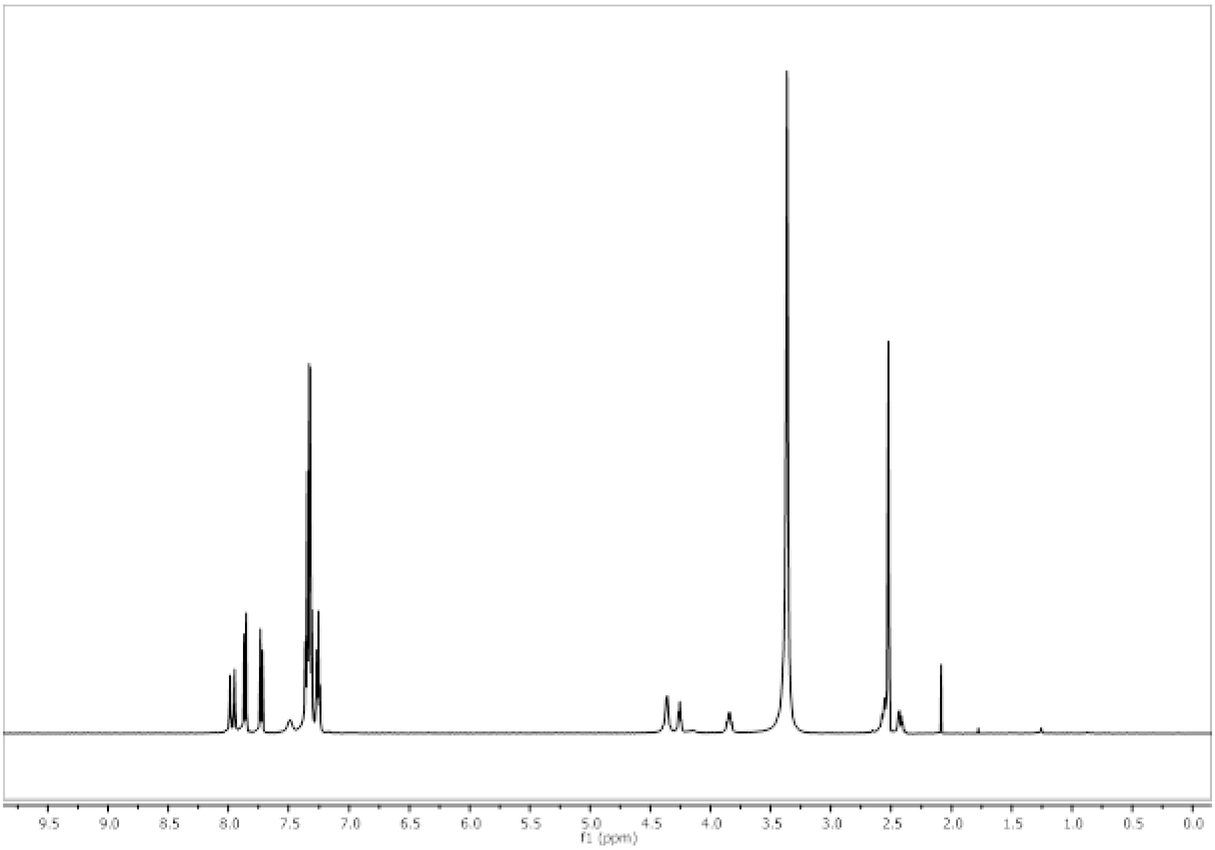

Figure S147: ^{1}H-NMR of Smoc-L-Cys(Trt)-OH **9**.

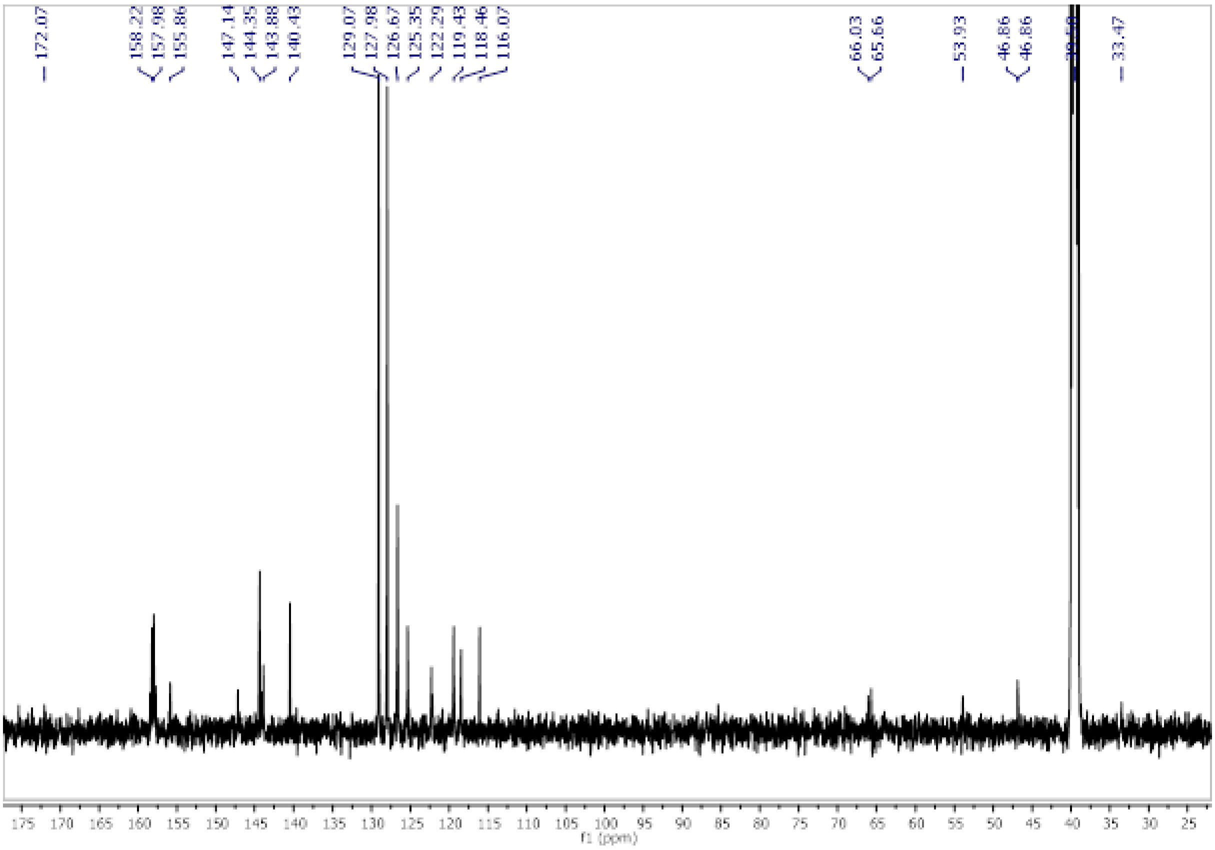

Figure S148: ^{13}C-NMR of Smoc-L-Cys(Trt)-OH **9**.

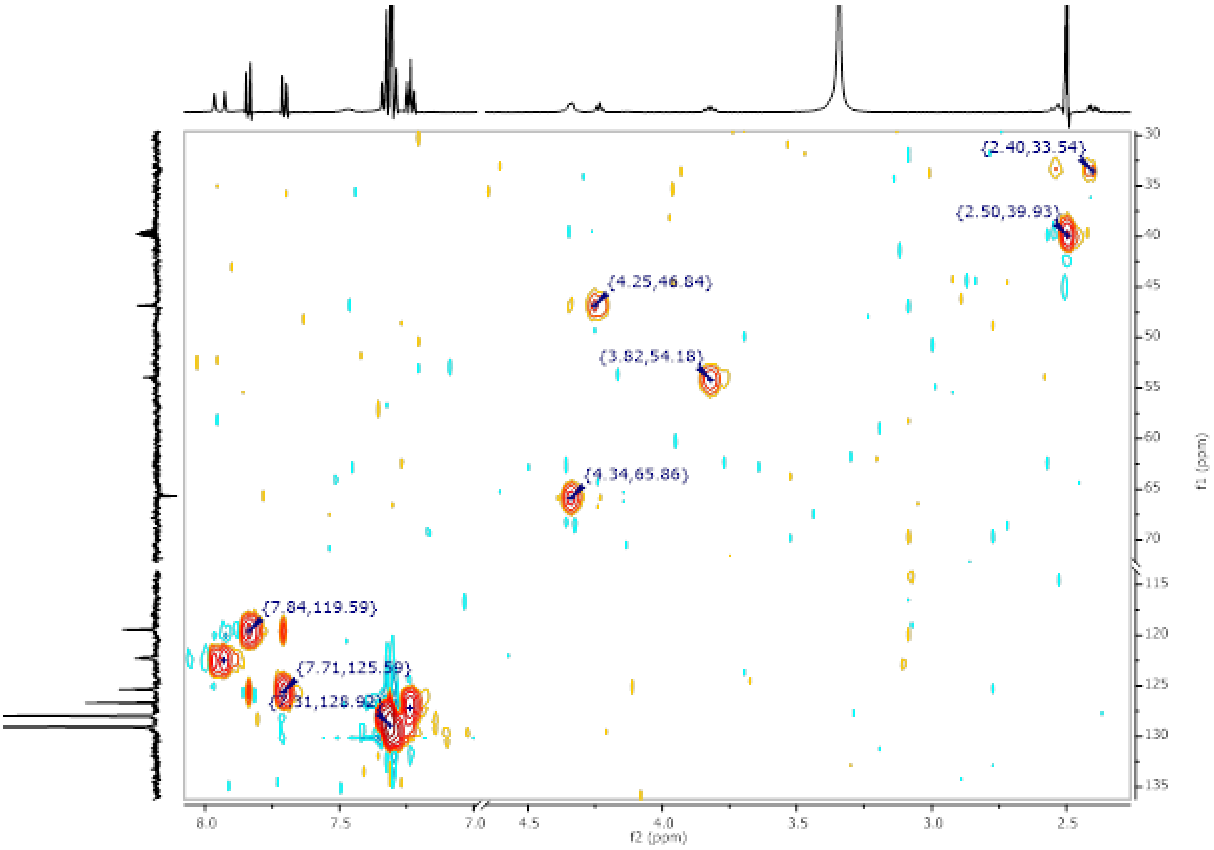

Figure S149: ^{1}H-^{13}C HSQC-NMR of Smoc-L-Cys(Trt)-OH **9**.

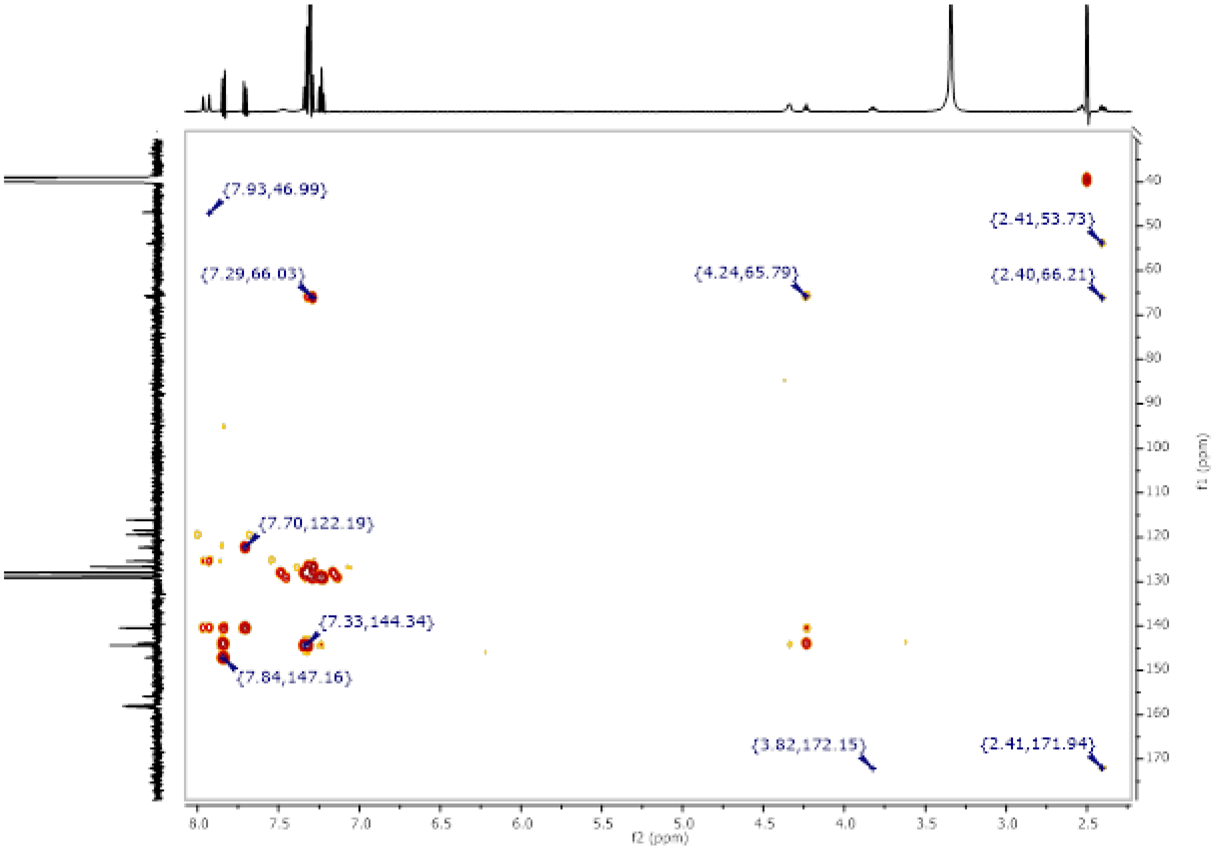

Figure S150: ^{1}H-^{13}C HMBC-NMR of Smoc-L-Cys(Trt)-OH **9**.

3.2.8. Analytical data of Smoc-L-Gln-OH 10

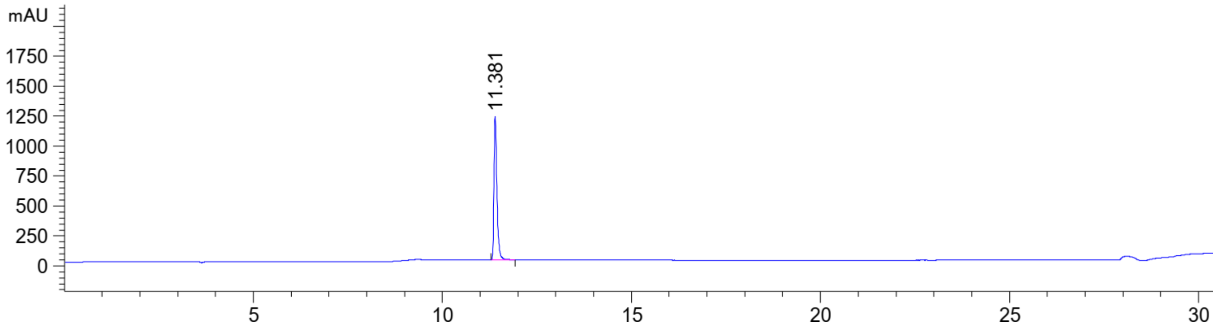

Figure S151: HPLC chromatogram of Smoc-L-Gln-OH **10** at λ=220 nm (0to40 MeCN).

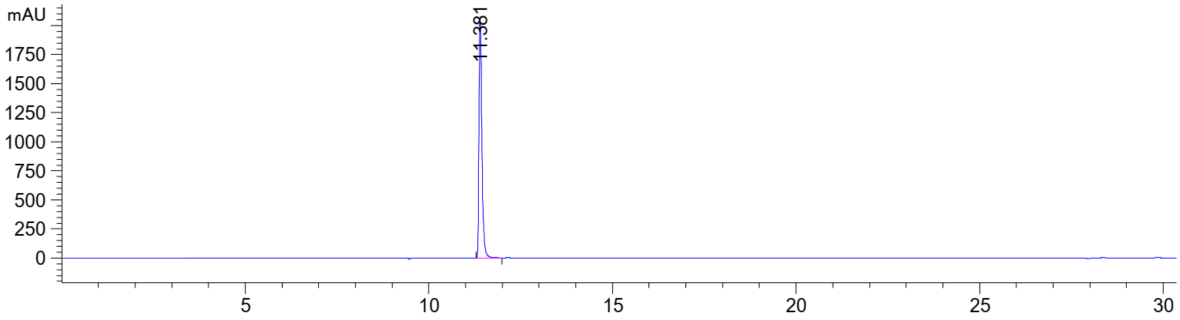

Figure S152: HPLC chromatogram of Smoc-L- Gln-OH **10** at λ=280 nm (0to40 MeCN).

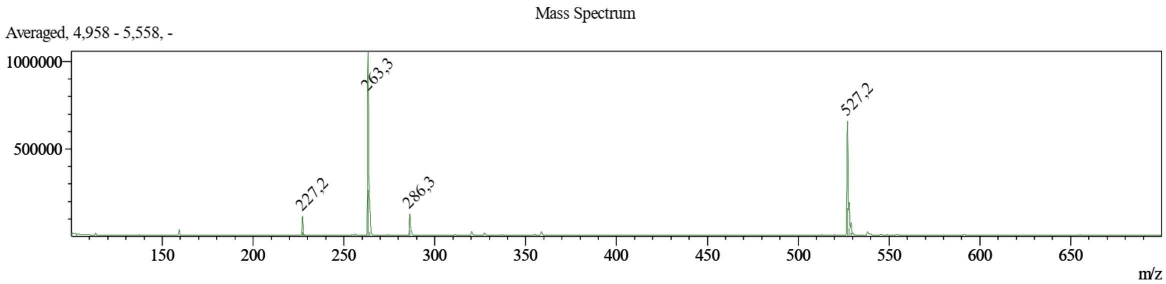

Figure S153: ESI-MS of Smoc-L- Gln-OH **10** (M measured=527.20 $[M-H]^-$, M calc.=528.50).

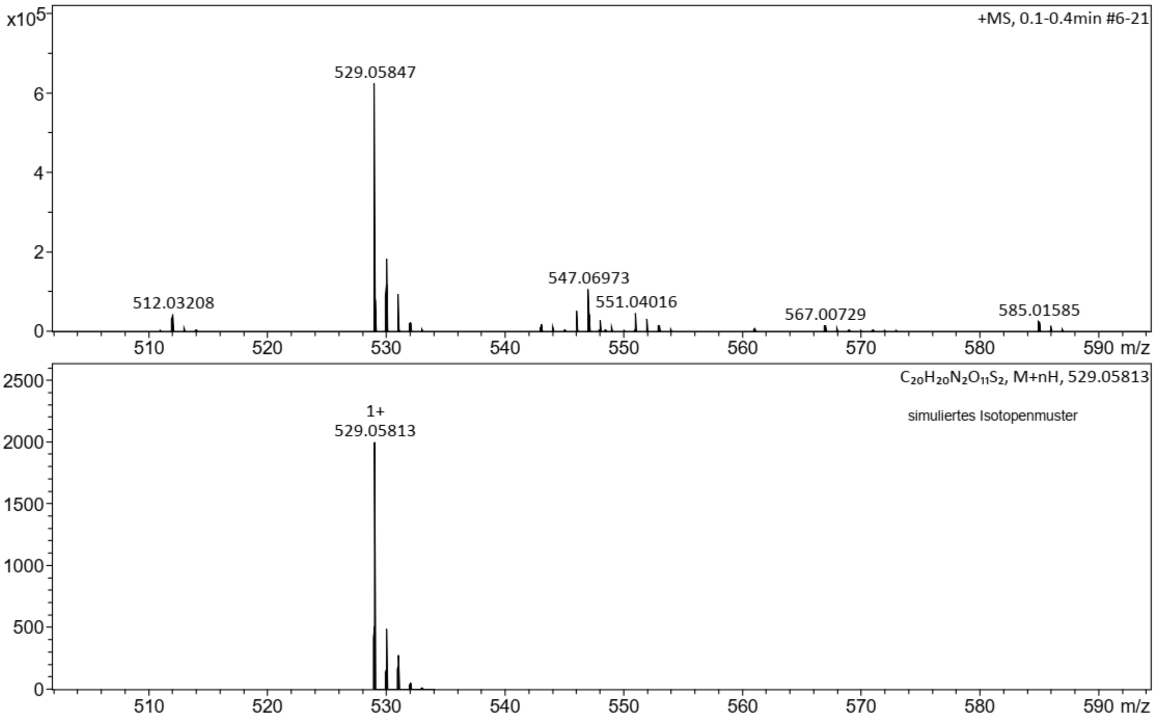

Figure S154: HR-MS of Smoc-L-Gln-OH **10** (M measured=529.05847 $[M+H]^+$, M calc.=529.05813).

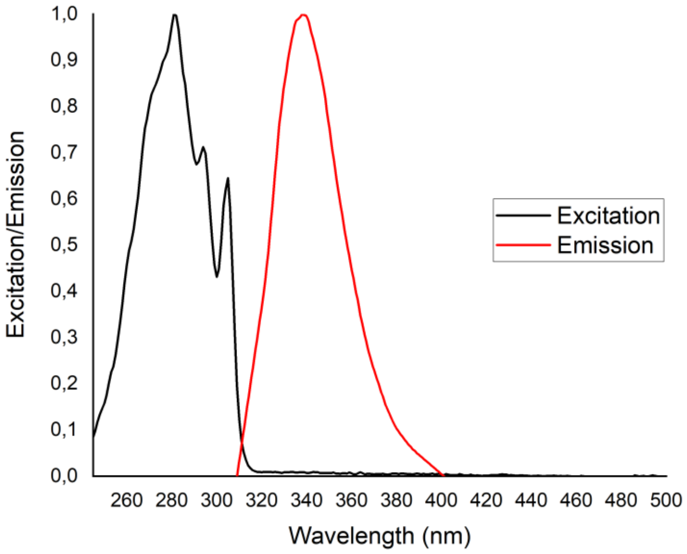

Figure S155: Excitation and emission spectra of Smoc-L-Gln-OH **10**, excitation and emission have been normalized between 0 and 1 for illustration.

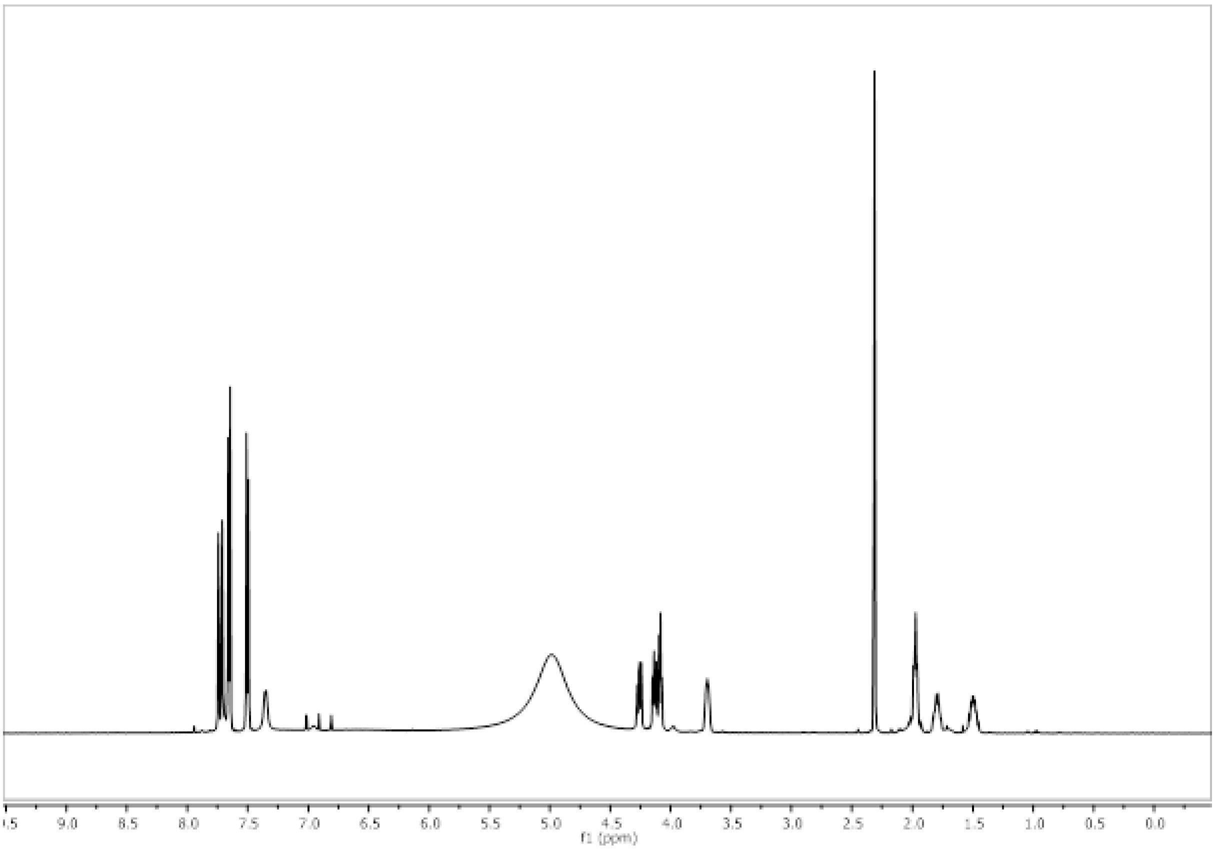

Figure S156: ^{1}H-NMR of Smoc-L- Gln-OH **10**.

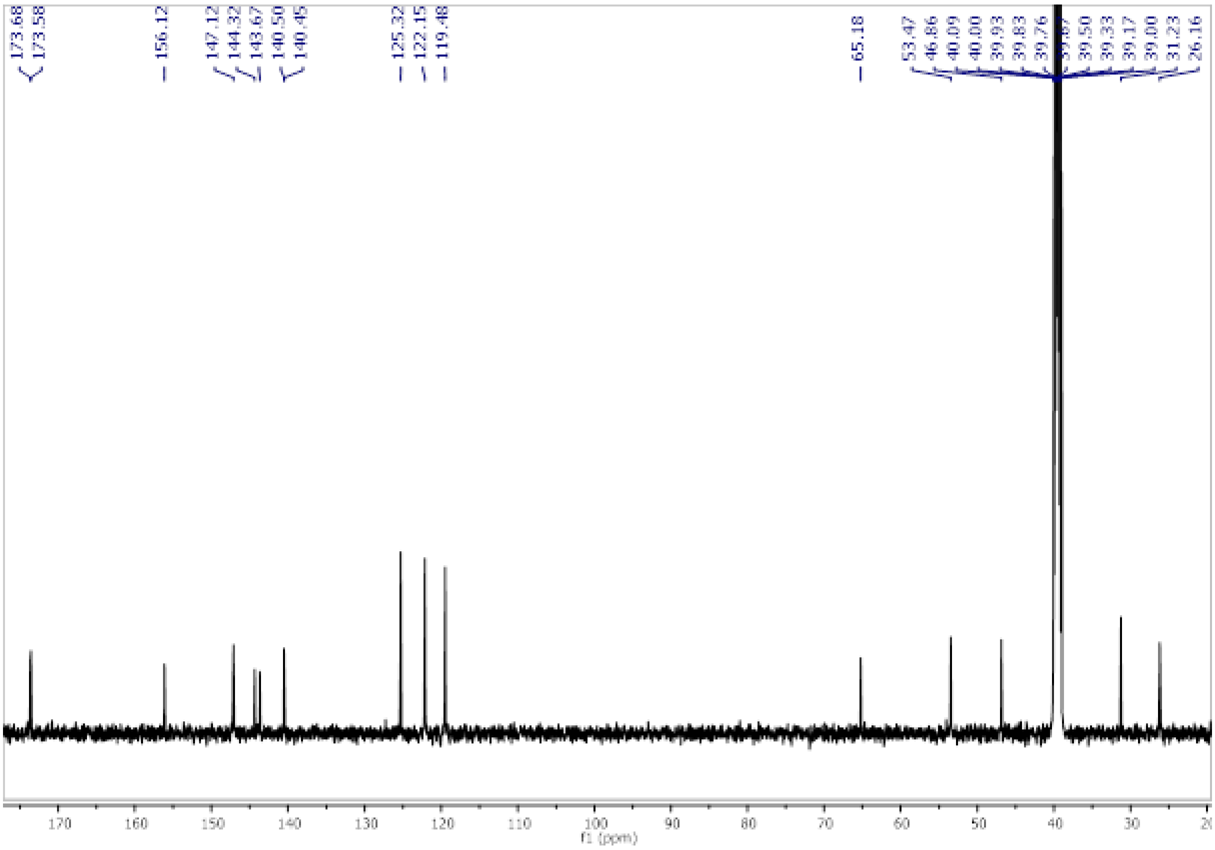

Figure S157: ^{13}C-NMR of Smoc-L- Gln-OH **10**.

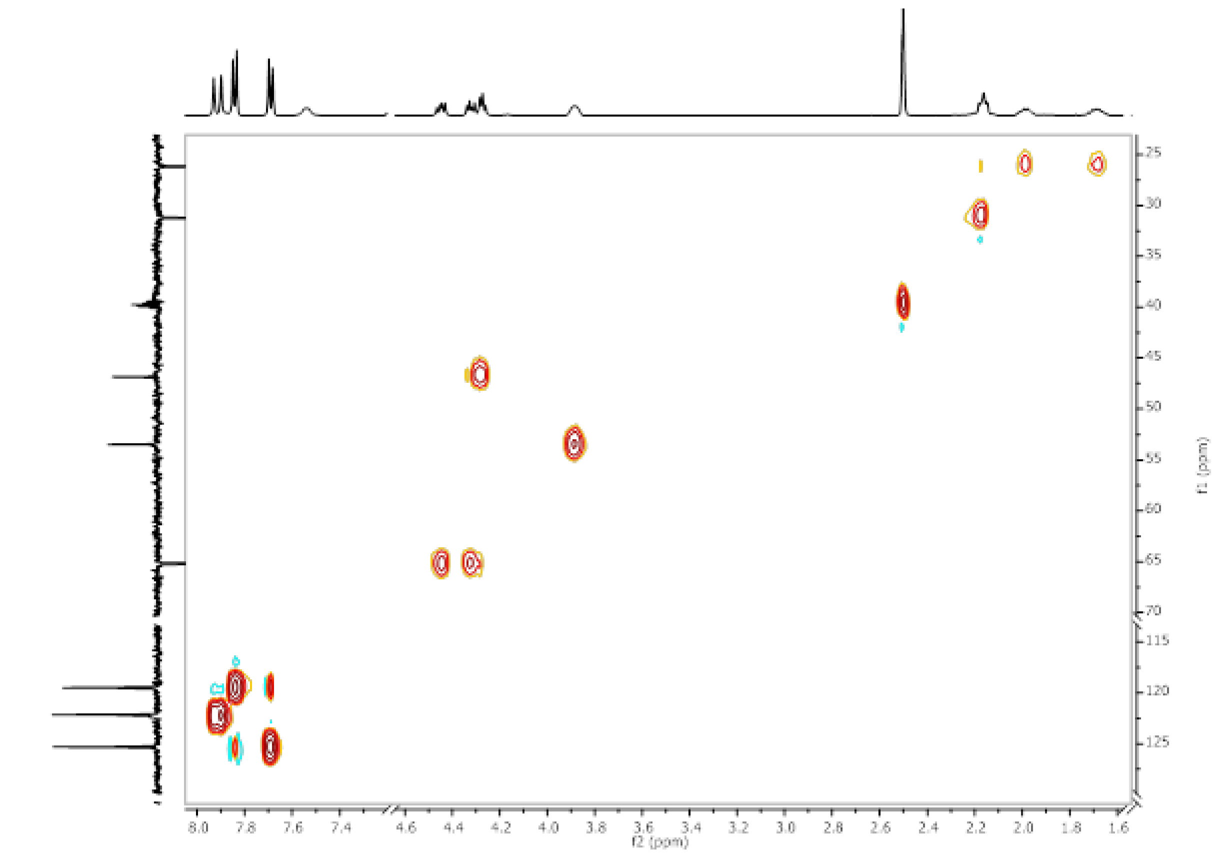

Figure S158: ^{1}H-^{13}C HSQC-NMR of Smoc-L- Gln-OH **10**.

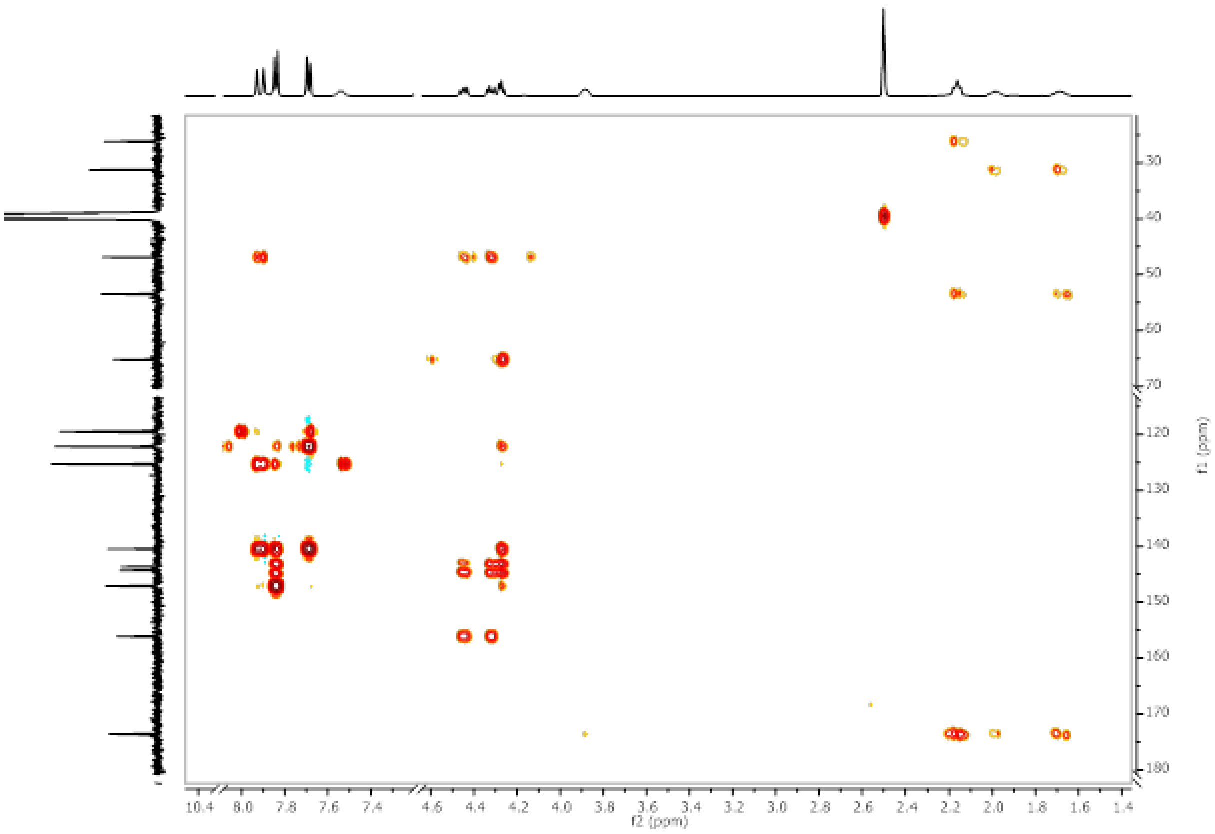

Figure S159: ^{1}H-^{13}C HMBC-NMR of Smoc-L- Gln-OH **10**.

3.2.9. Analytical data of Smoc-L-Glu(OtBu)-OH 11

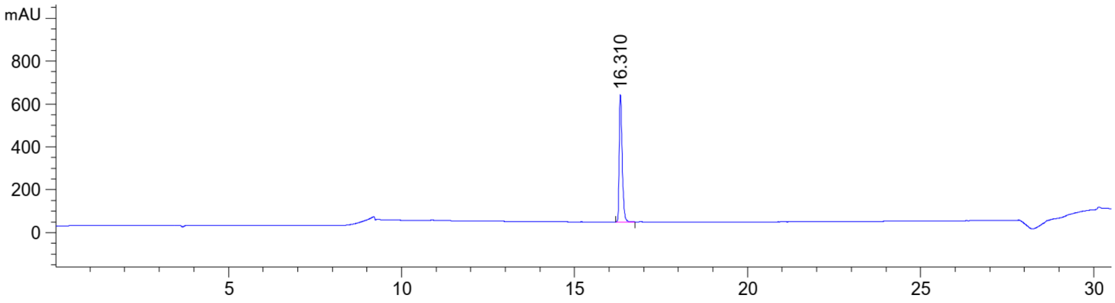

Figure S160: HPLC chromatogram of Smoc-L-Glu(OtBu)-OH **11** at λ=220 nm (0to60 MeCN).

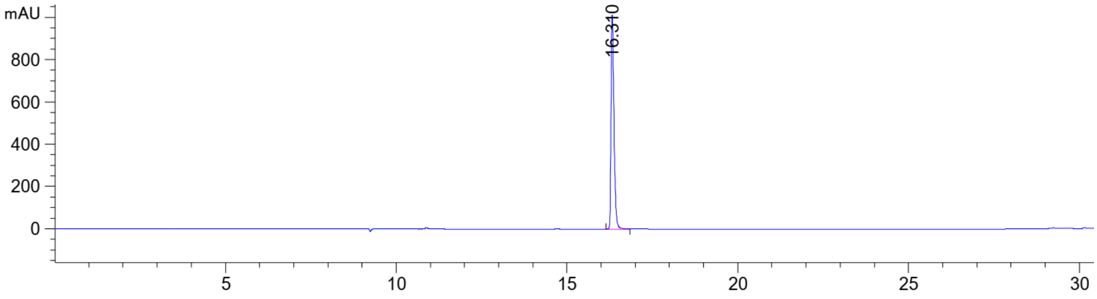

Figure S161: HPLC chromatogram of Smoc-L-Glu(OtBu)-OH **11** at λ=280 nm (0to60 MeCN).

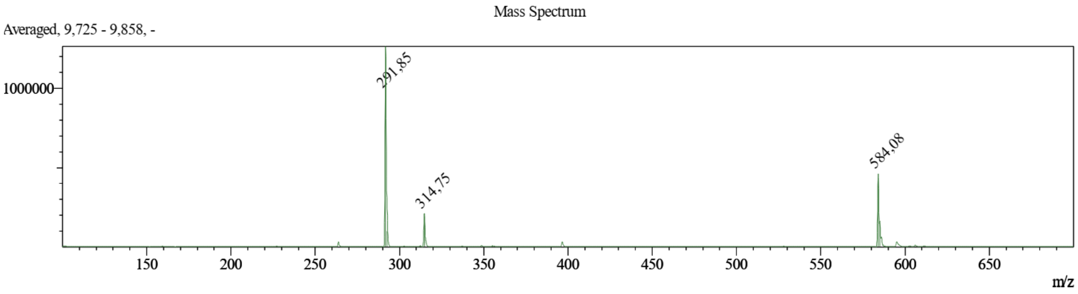

Figure S162: ESI-MS of Smoc-L-Glu(OtBu)-OH **11** (M measured=584.08 [M-H]$^-$, M calc.=585.60).

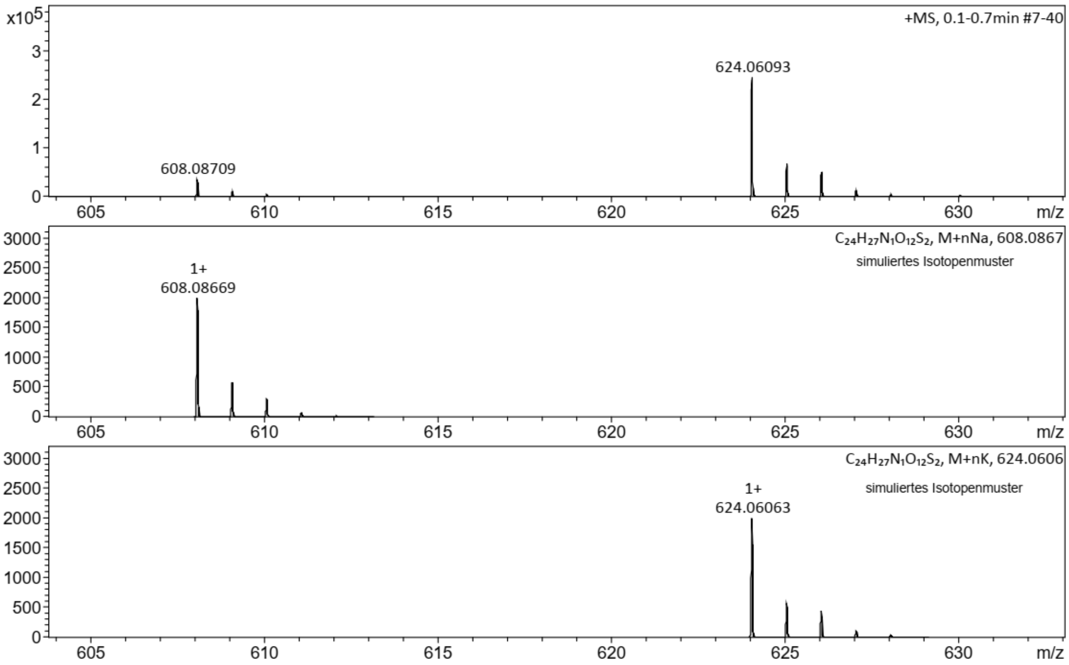

Figure S163: HR-MS of Smoc-L-Glu(OtBu)-OH **11** (M measured=608.08669 [M+H]$^+$, M calc.=608.08709).

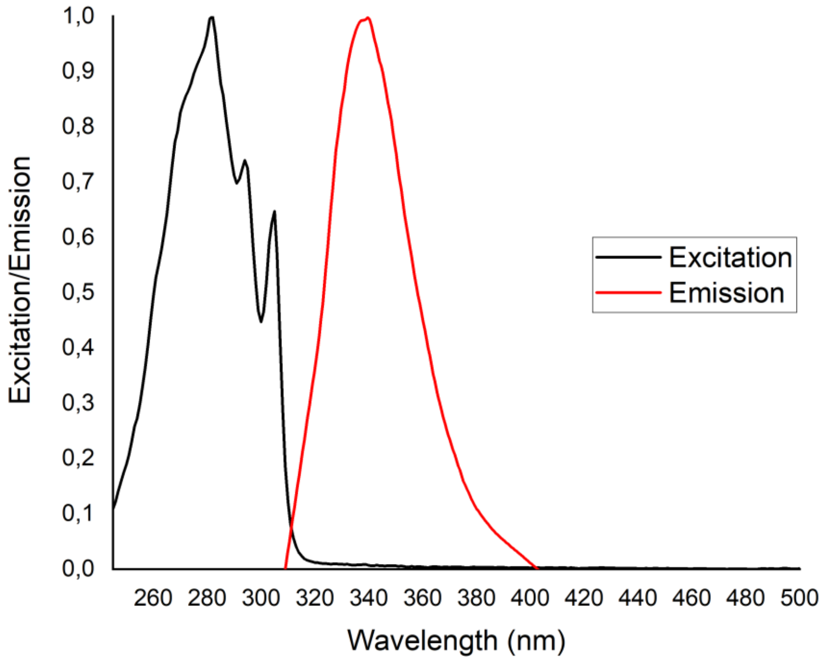

Figure S164: Excitation and emission spectra of Smoc-L-Glu(OtBu)-OH **11**, excitation and emission have been normalized between 0 and 1 for illustration.

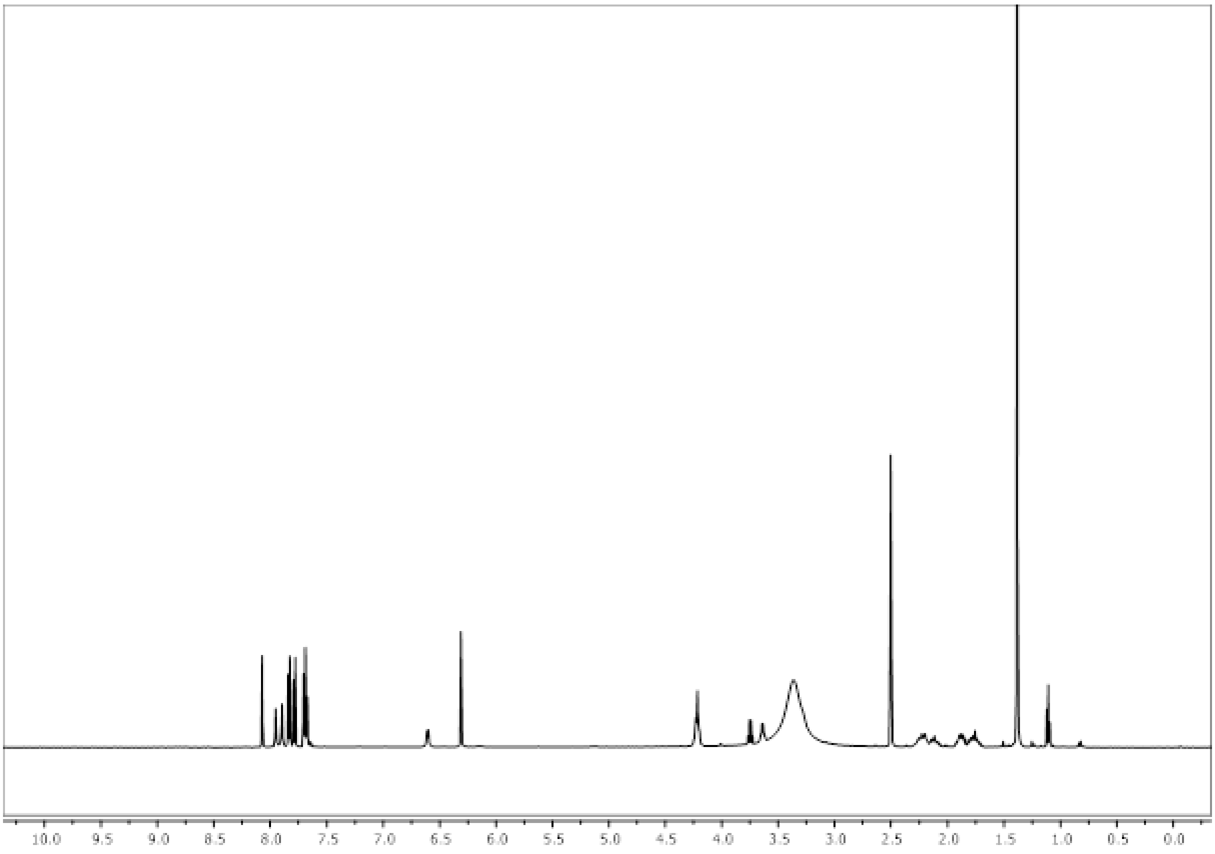

Figure S165: ^{1}H-NMR of Smoc-L-Glu(OtBu)-OH **11**.

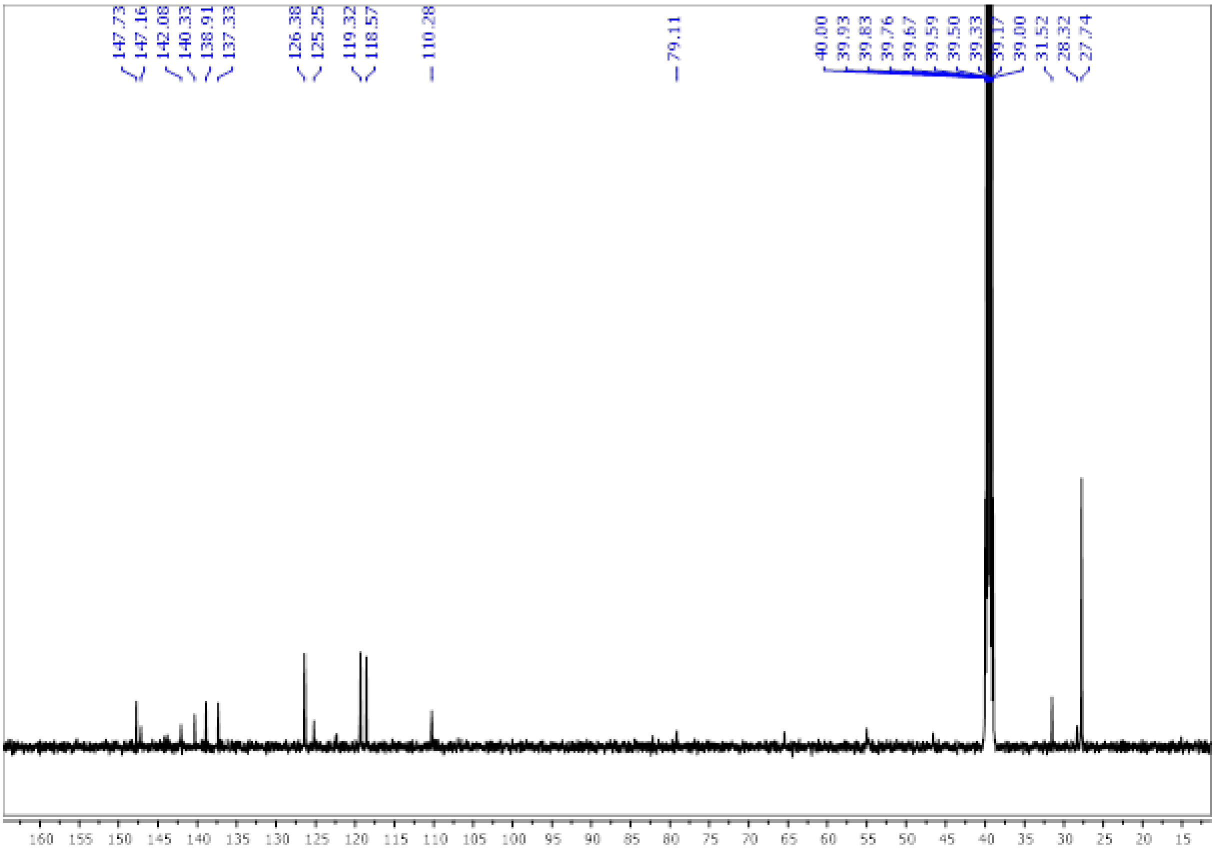

Figure S166: ^{13}C-NMR of Smoc-L-Glu(OtBu)-OH **11**.

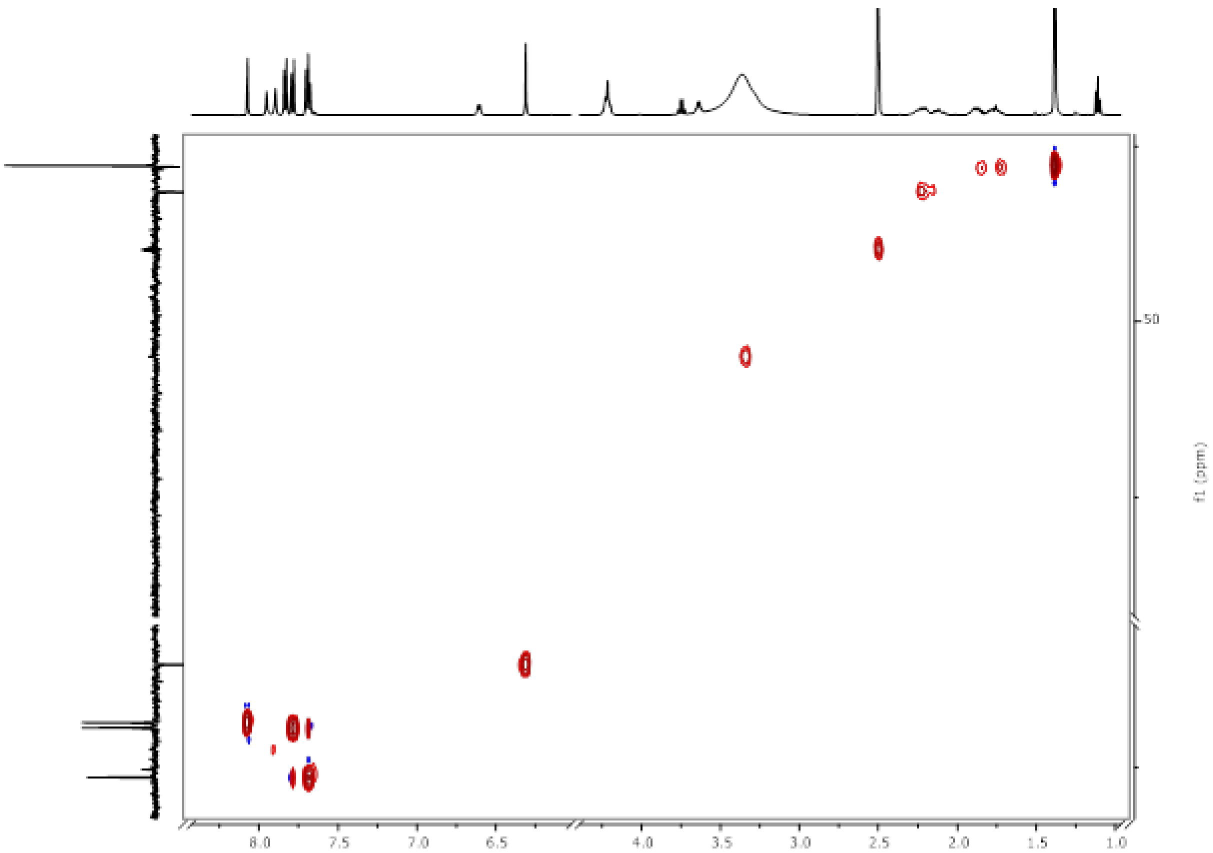

Figure S167: ^{1}H-^{13}C HSQC-NMR of Smoc-L-Glu(OtBu)-OH **11**.

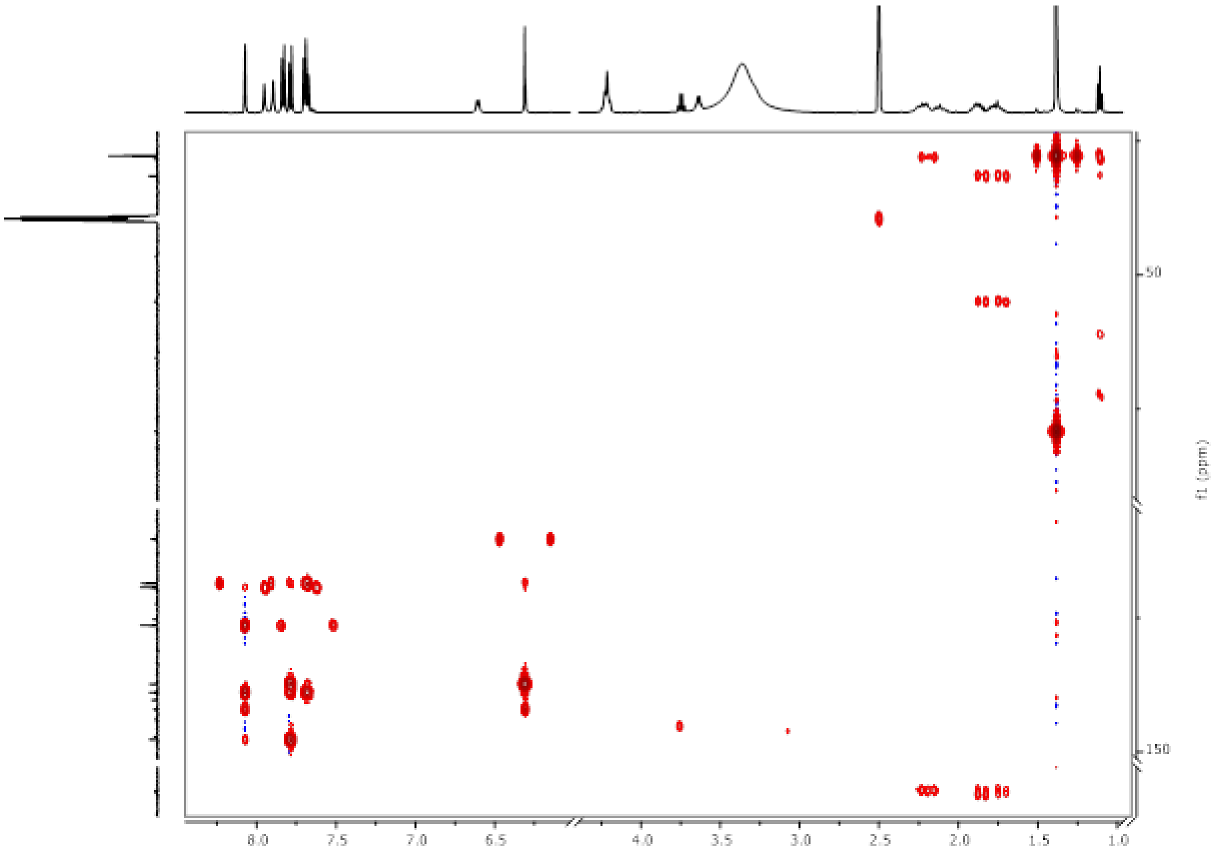

Figure S168: ^{1}H-^{13}C HMBC-NMR of Smoc-L-Glu(OtBu)-OH **11**.

3.2.10. Analytical data of Smoc-Gly-OH 12

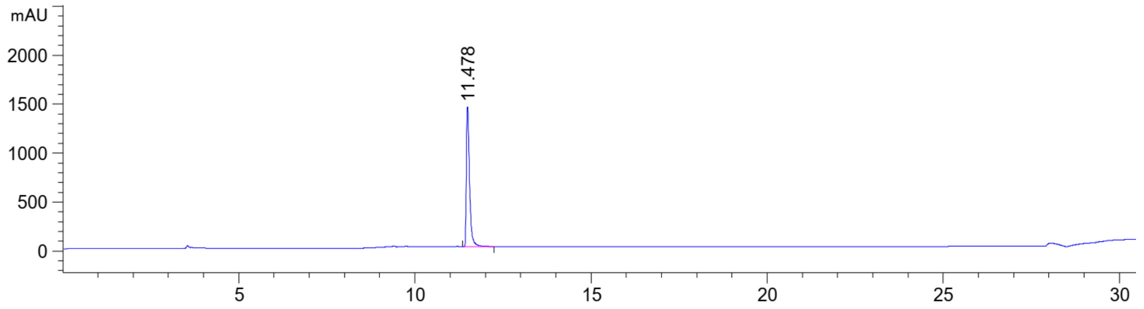

Figure S169: HPLC chromatogram of Smoc-Gly-OH **12** at λ=220 nm (0to40 MeCN).

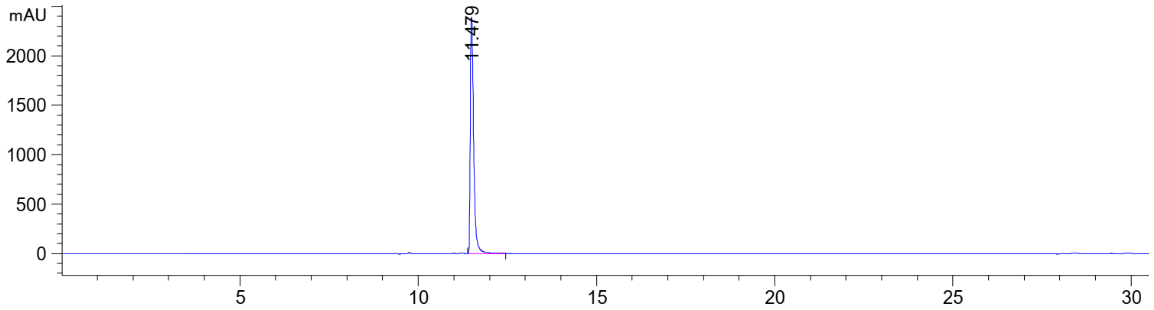

Figure S170: HPLC chromatogram of Smoc-Gly-OH **12** at λ=280 nm (0to40 MeCN).

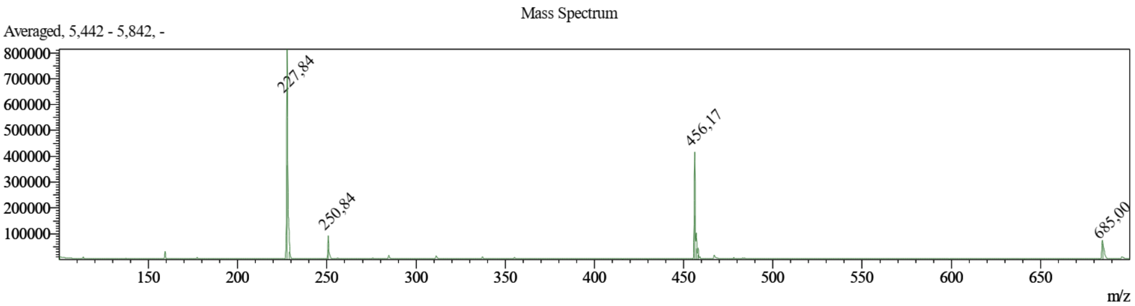

Figure S171: ESI-MS of Smoc-Gly-OH **12** (M measured=456.17 [M-H]$^-$, M calc.=457.42).

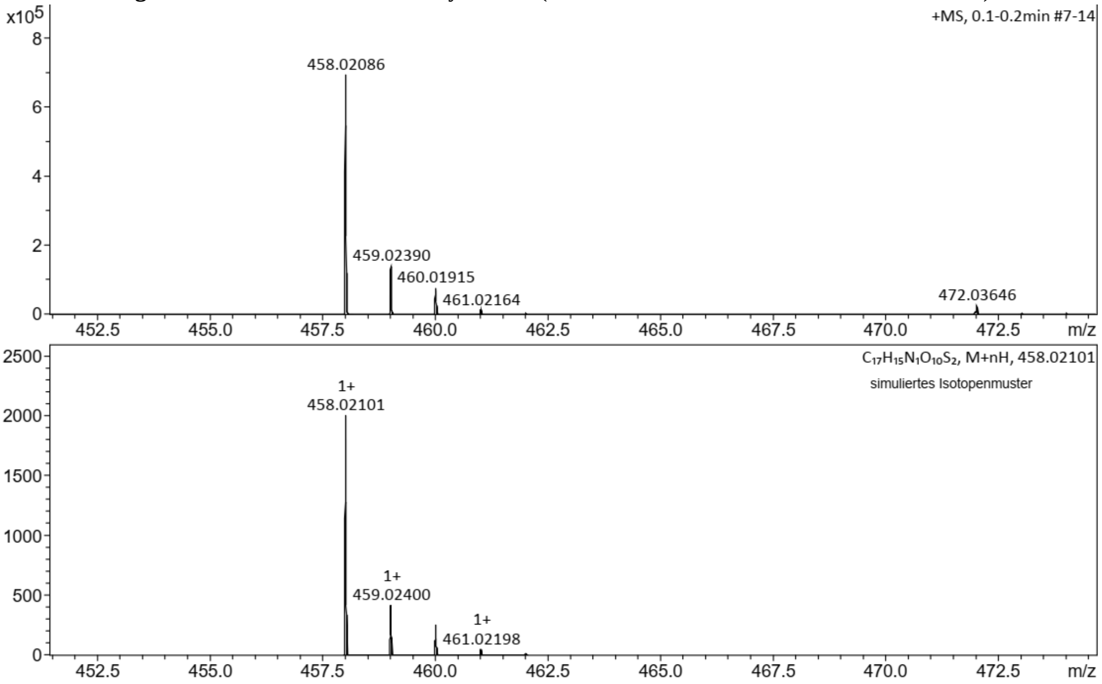

Figure S172: HR-MS of Smoc-Gly-OH **12** (M measured=458.02086 [M+H]$^+$, M calc.=458.02101).

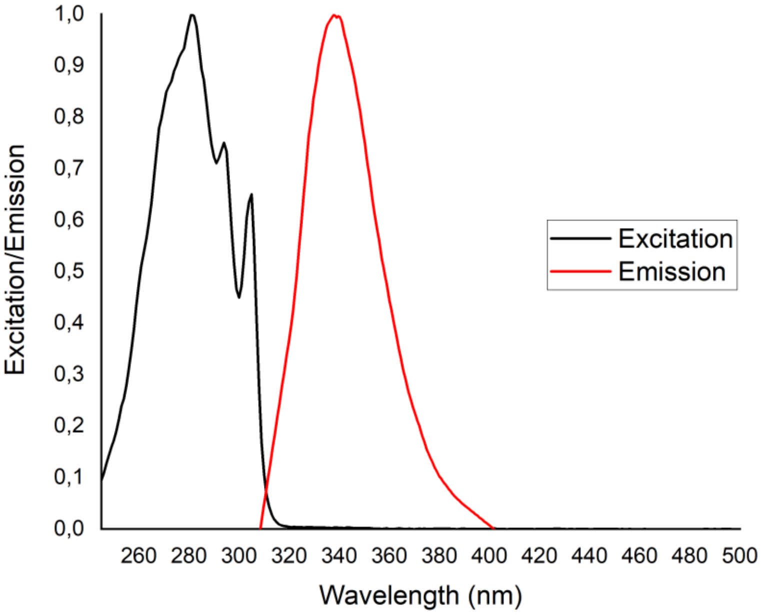

Figure S173: Excitation and emission spectra of Smoc-Gly-OH **12**, excitation and emission have been normalized between 0 and 1 for illustration.

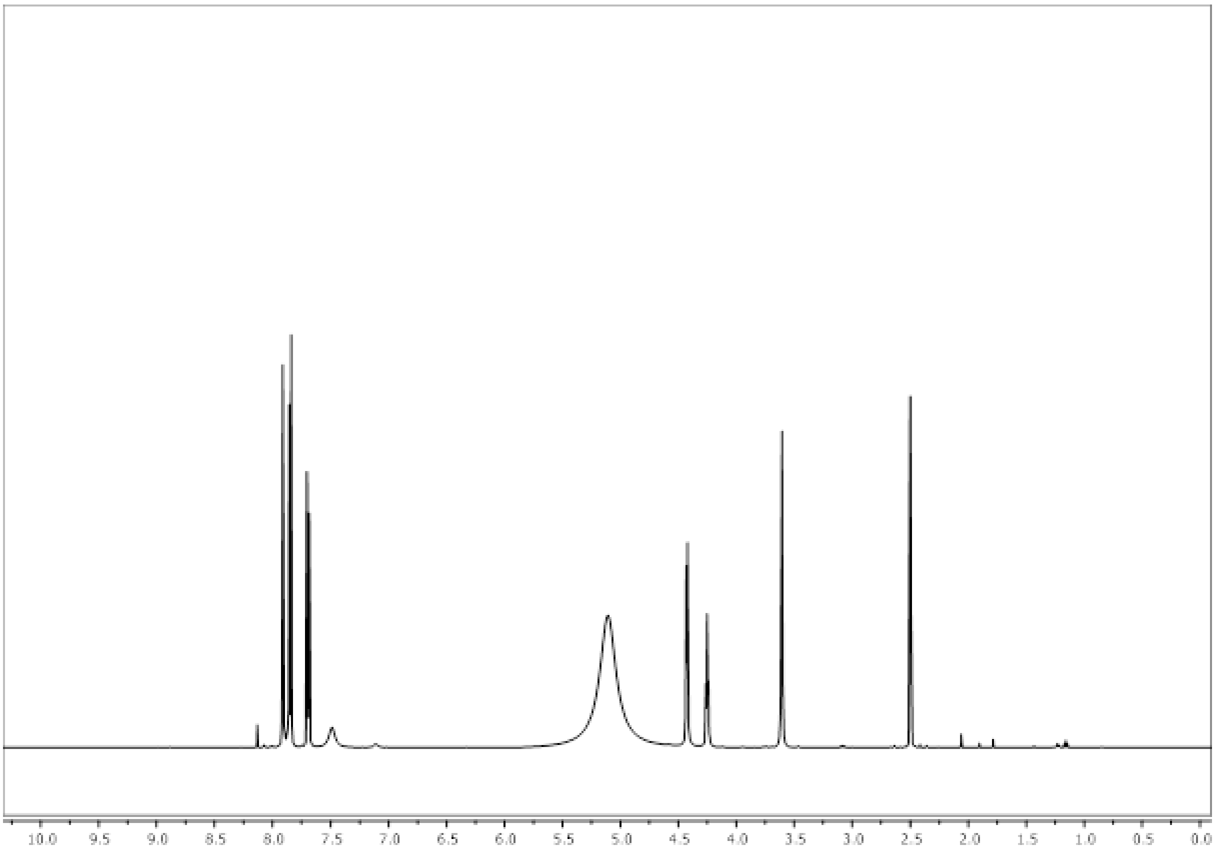

Figure S174: ^{1}H-NMR of Smoc-Gly-OH **12**.

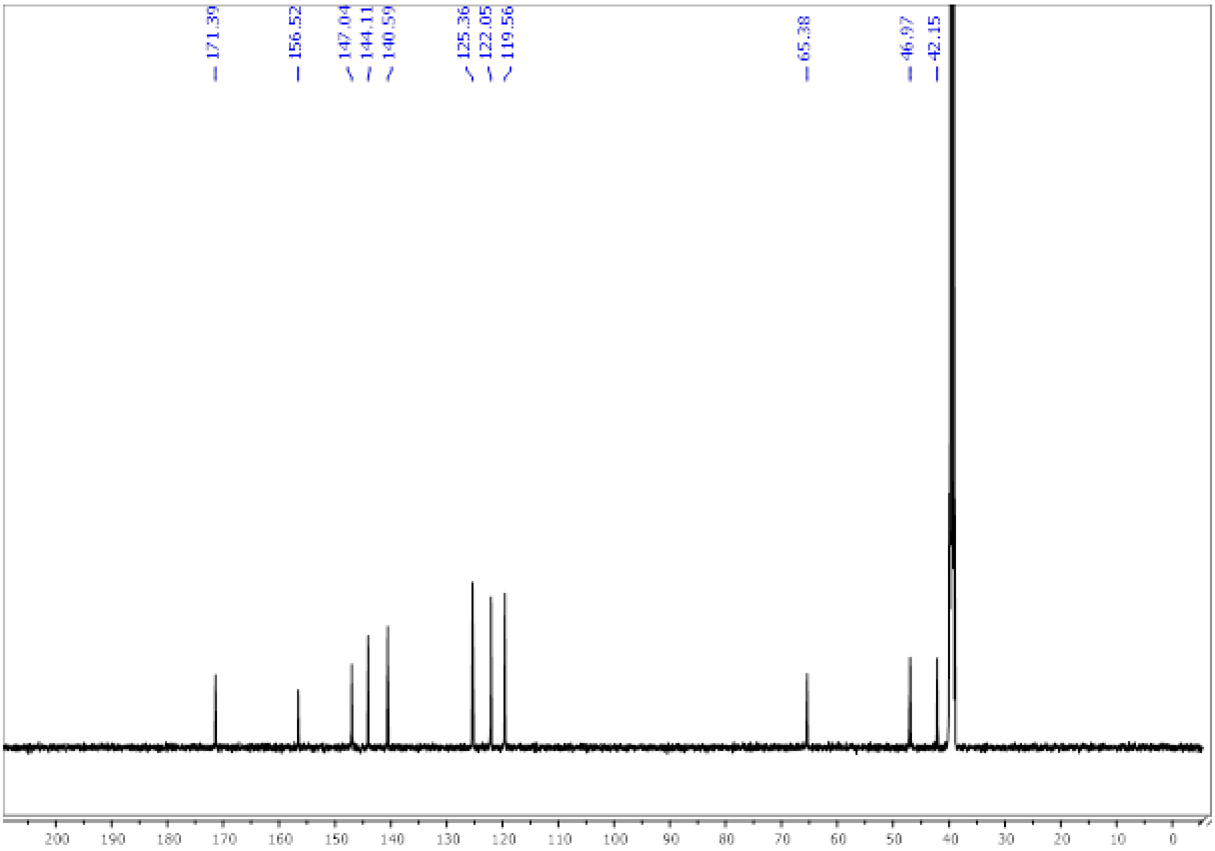

Figure S175: ^{13}C-NMR of Smoc-Gly-OH **12**.

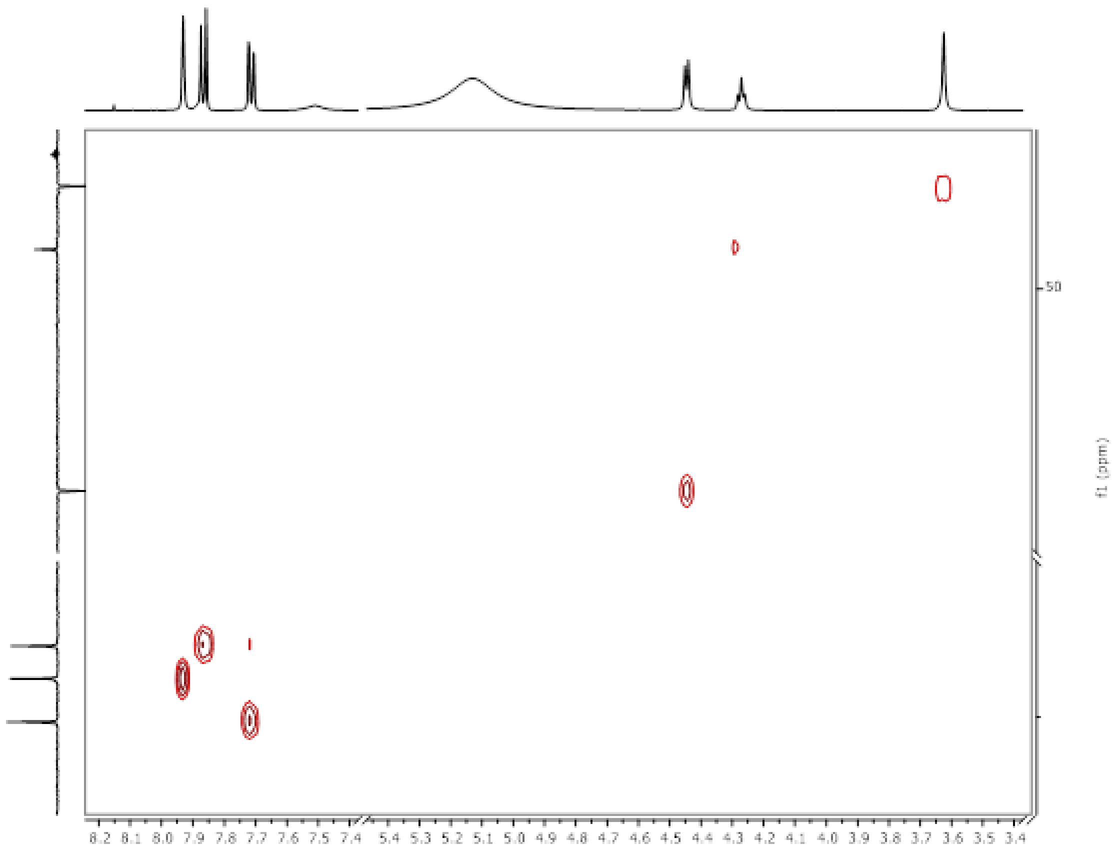

Figure S176: ^{1}H-^{13}C HSQC-NMR of Smoc-Gly-OH **12**.

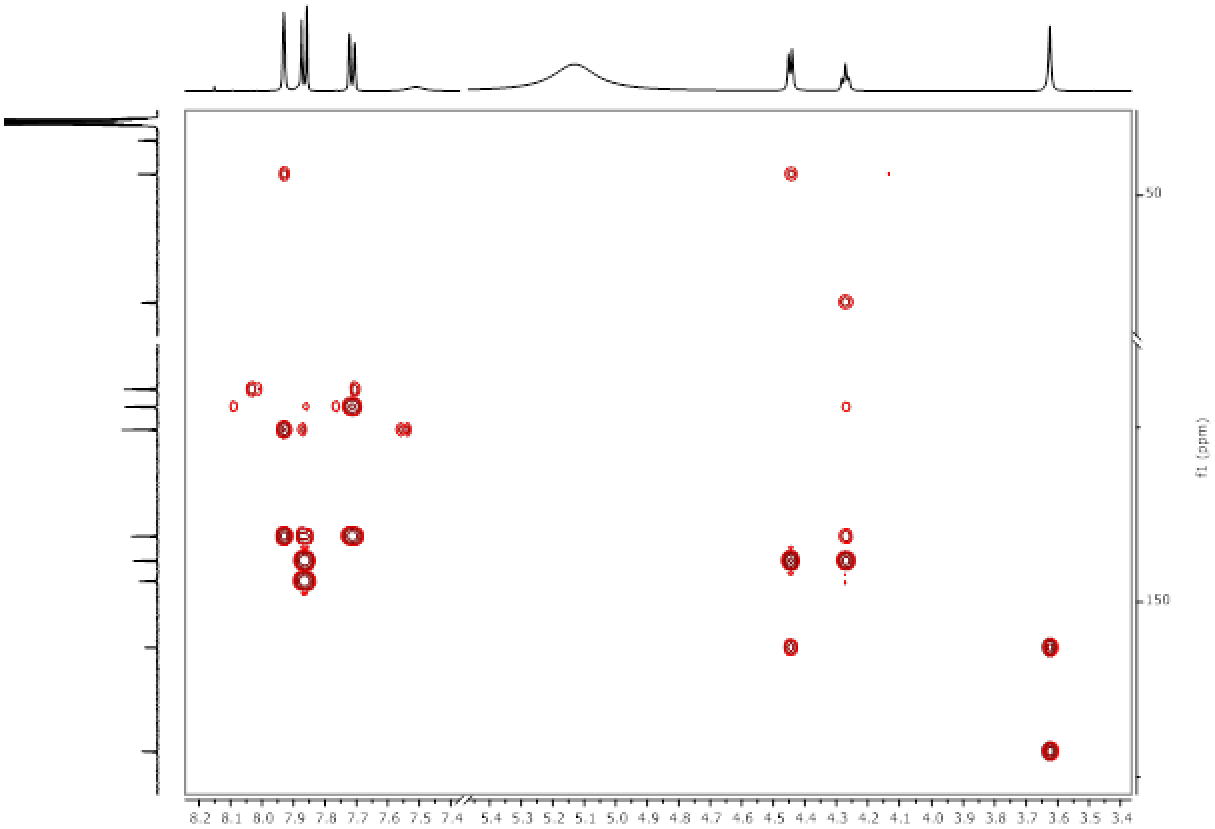

Figure S177: ^{1}H-^{13}C HMBC-NMR of Smoc-Gly-OH **12**.

3.2.11. Analytical data of Smoc-L-His-OH 13

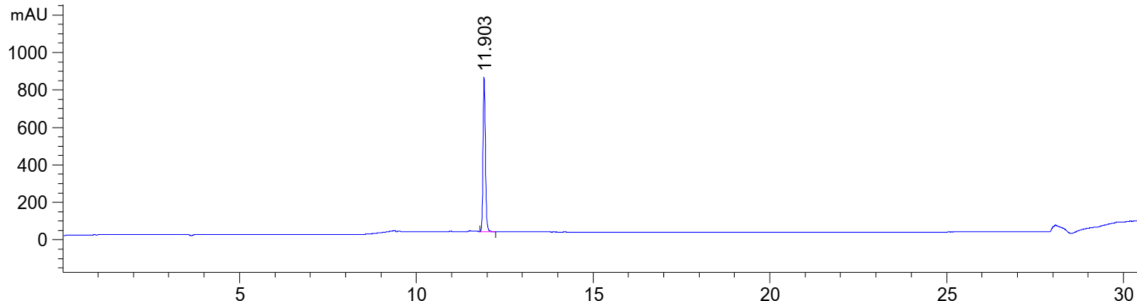

Figure S178: HPLC chromatogram of Smoc-L-His-OH **13** at λ=220 nm (0to40 MeCN).

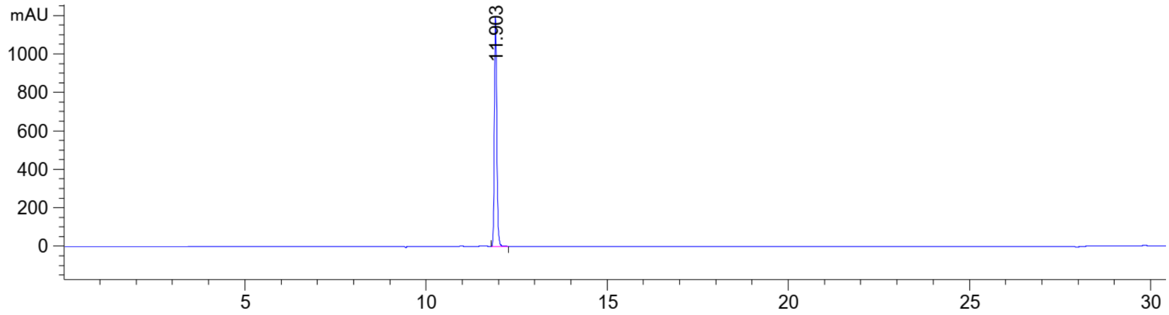

Figure S179: HPLC chromatogram of Smoc-L-His-OH **13** at λ=280 nm (0to40 MeCN).

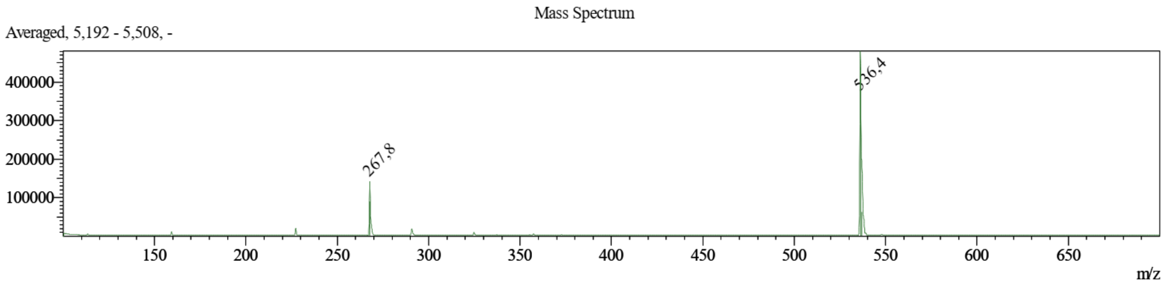

Figure S180: ESI-MS of Smoc-L-His-OH **13** (M measured=534.40 [M-H]$^-$, M calc.=537.51).

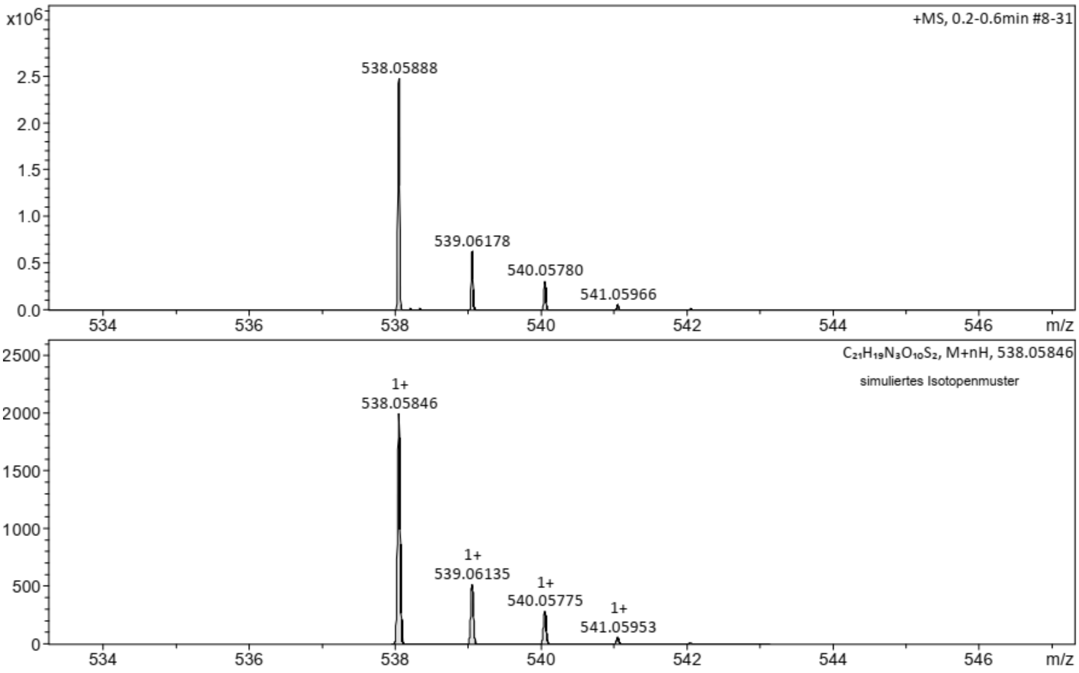

Figure S181: HR-MS of Smoc-L-His-OH **13** (M measured=538.05888 [M+H]$^+$, M calc.=538.05846).

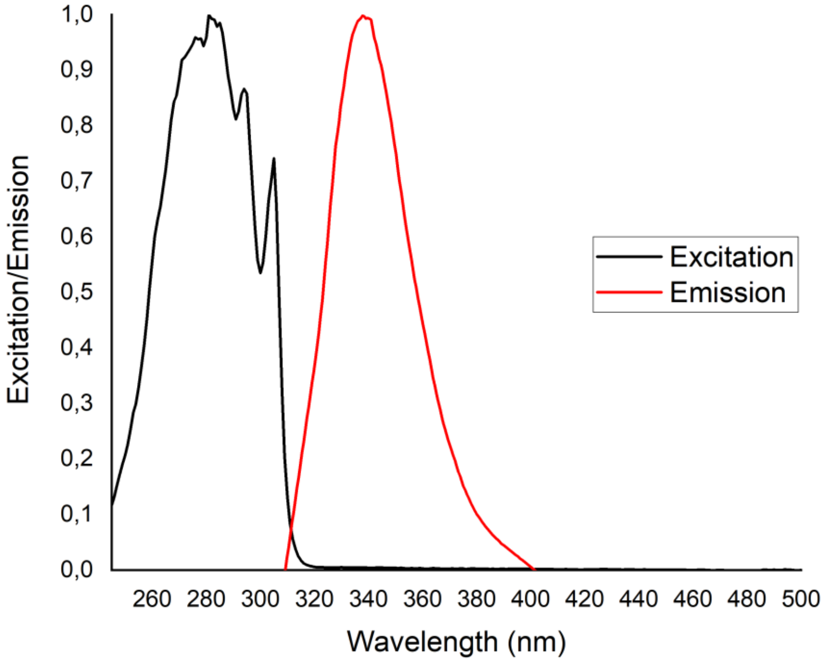

Figure S182: Excitation and emission spectra of Smoc-L-His-OH **13**, excitation and emission have been normalized between 0 and 1 for illustration.

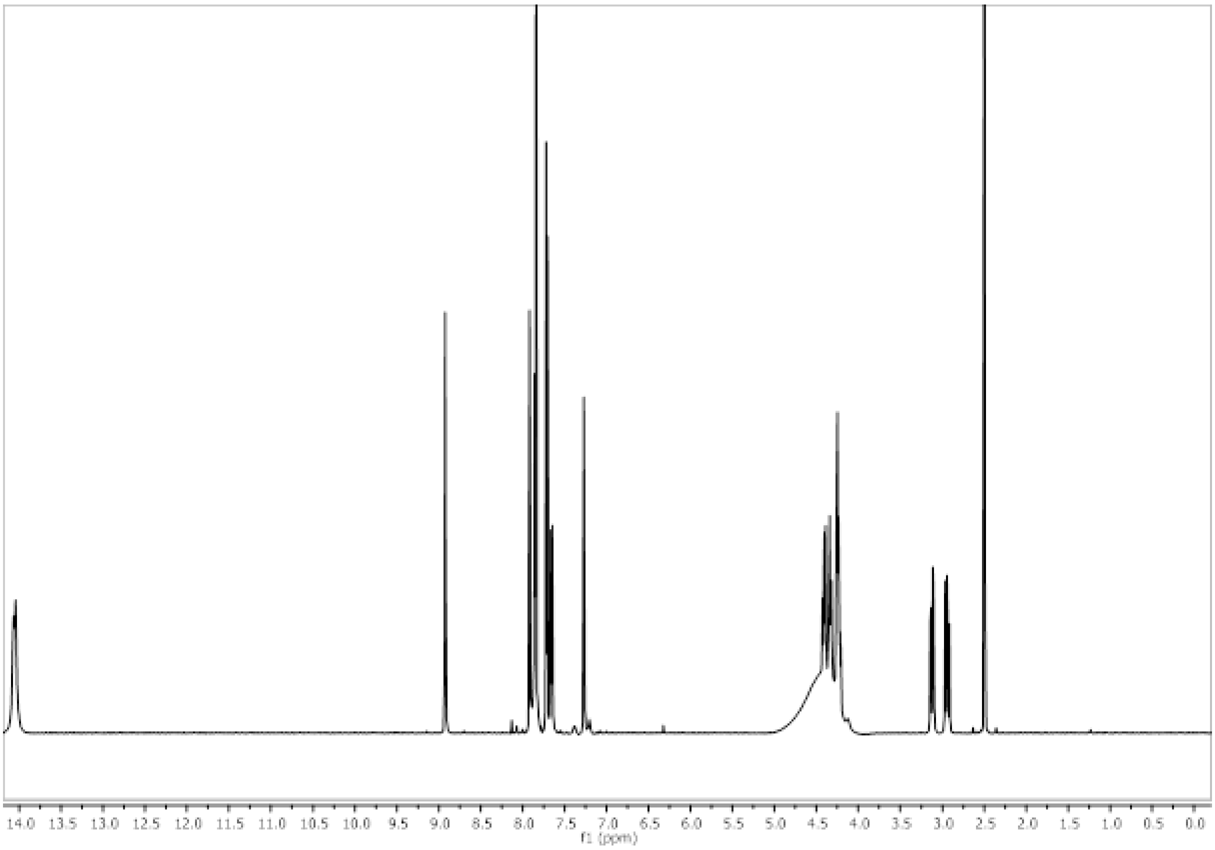

Figure S183: ^{1}H-NMR of Smoc-L-His-OH **13**.

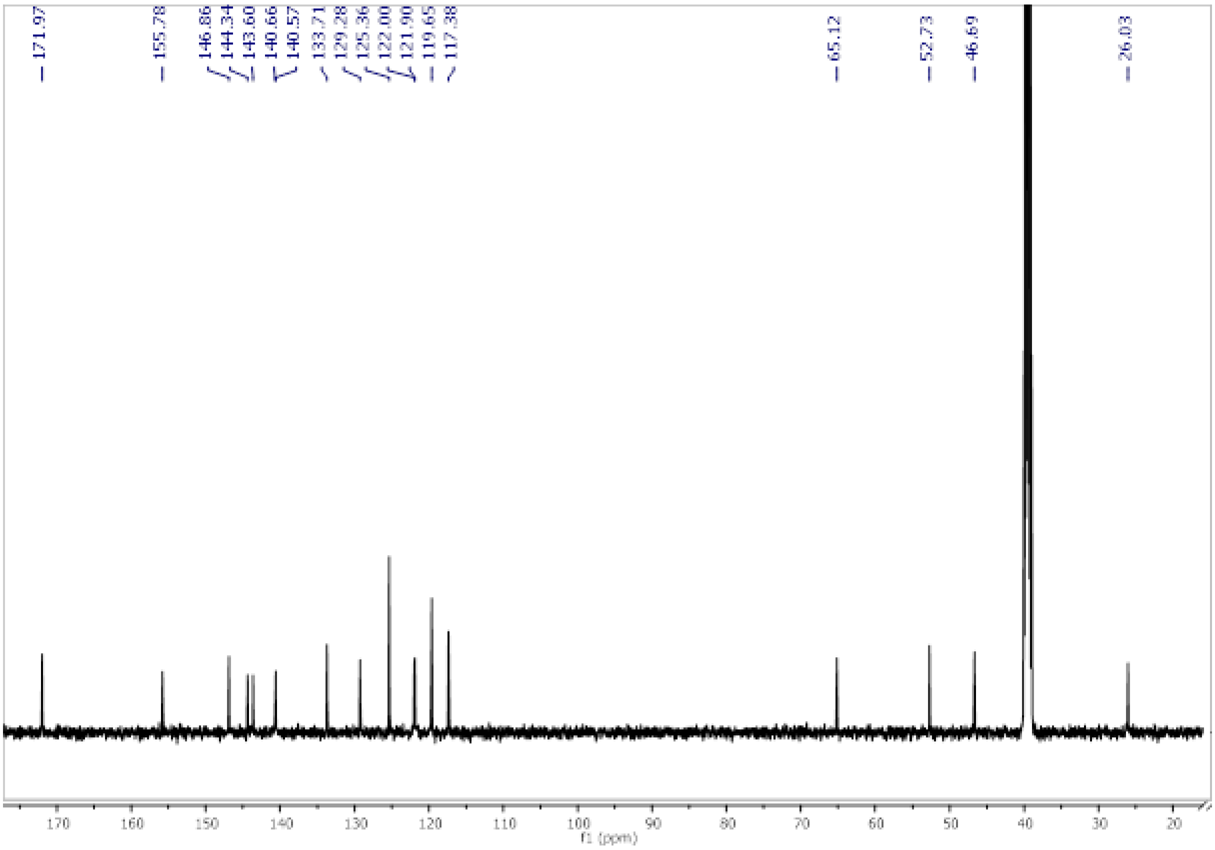

Figure S184: ^{13}C-NMR of Smoc-L-His-OH **13**.

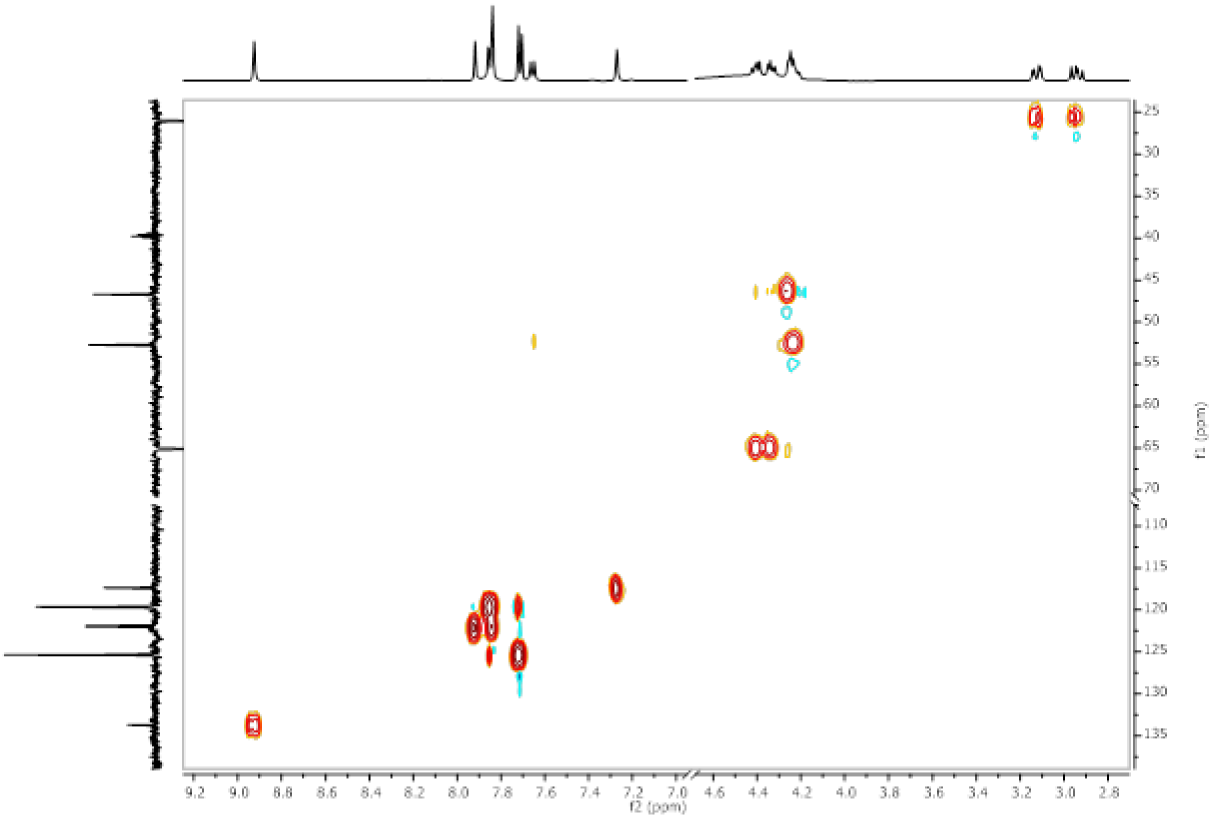

Figure S185: ^{1}H-^{13}C HSQC-NMR of Smoc-L-His-OH **13**.

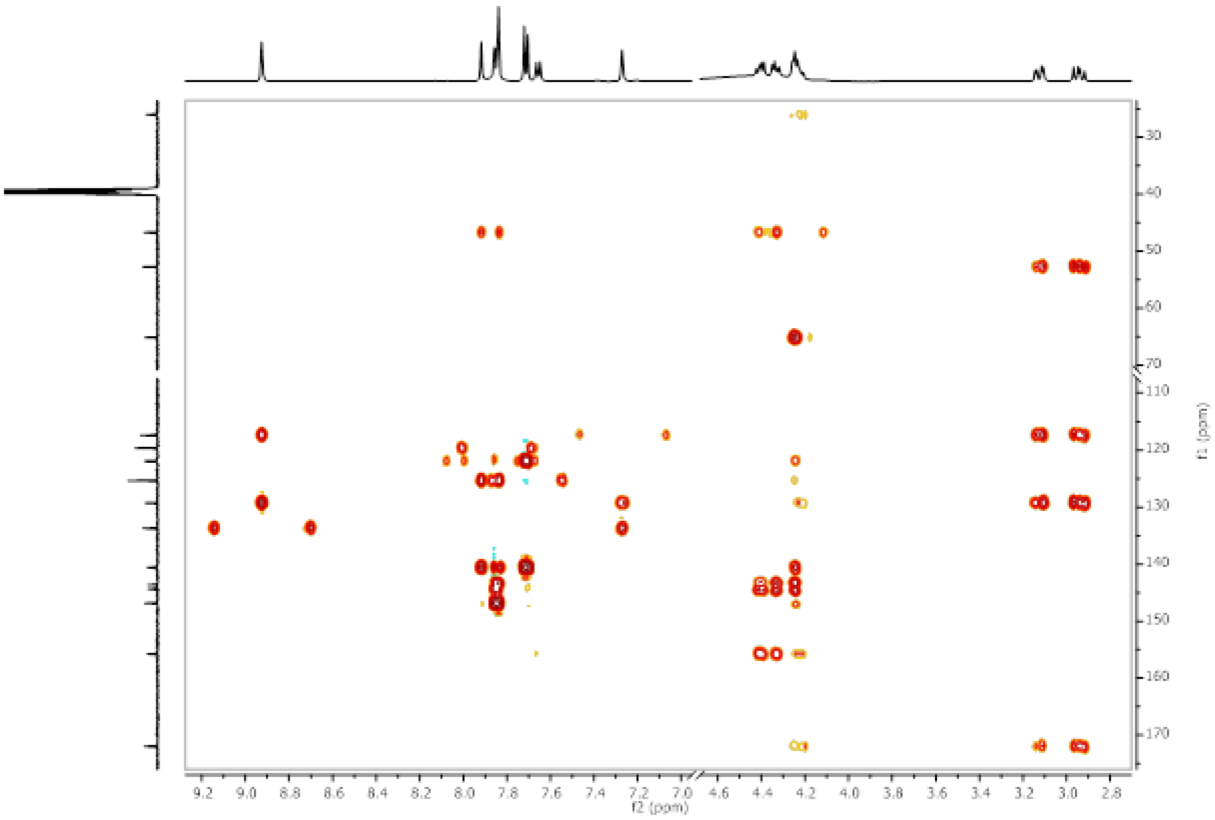

Figure S186: ^{1}H-^{13}C HMBC-NMR of Smoc-L-His-OH **13**.

3.2.12. Analytical data of Smoc-L-His(Trt)-OH 14

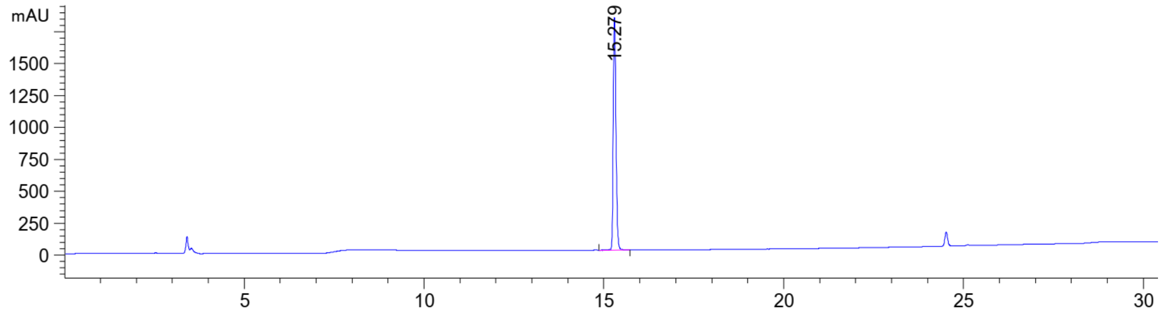

Figure S187: HPLC chromatogram of Smoc-L-His(Trt)-OH **14** at λ=220 nm (10to100 MeCN).

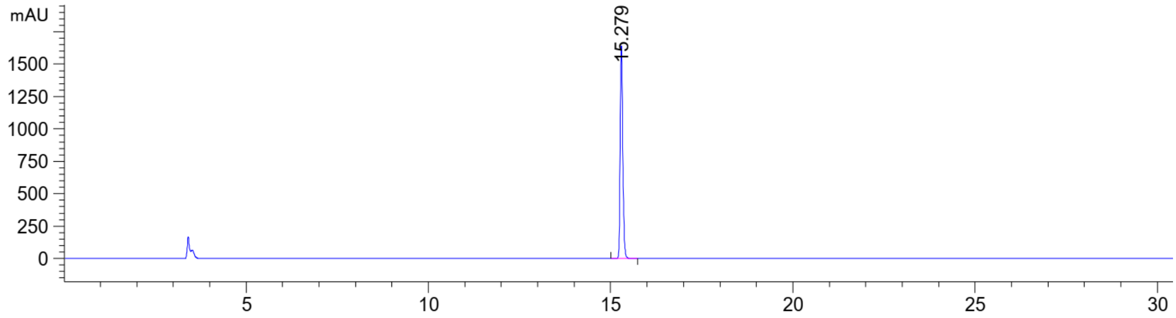

Figure S188: HPLC chromatogram of Smoc-L-His(Trt)-OH **14** at λ=280 nm (10to100 MeCN).

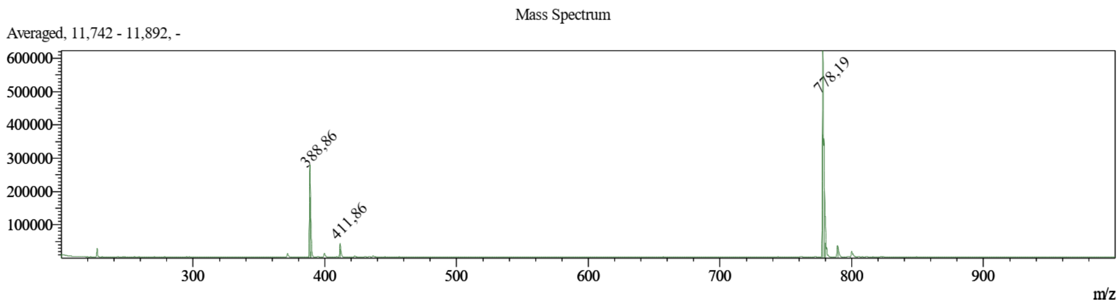

Figure S189: ESI-MS of Smoc-L-His(Trt)-OH **14** (M measured=778.19 [M-H]$^{-}$, M calc.=779.84).

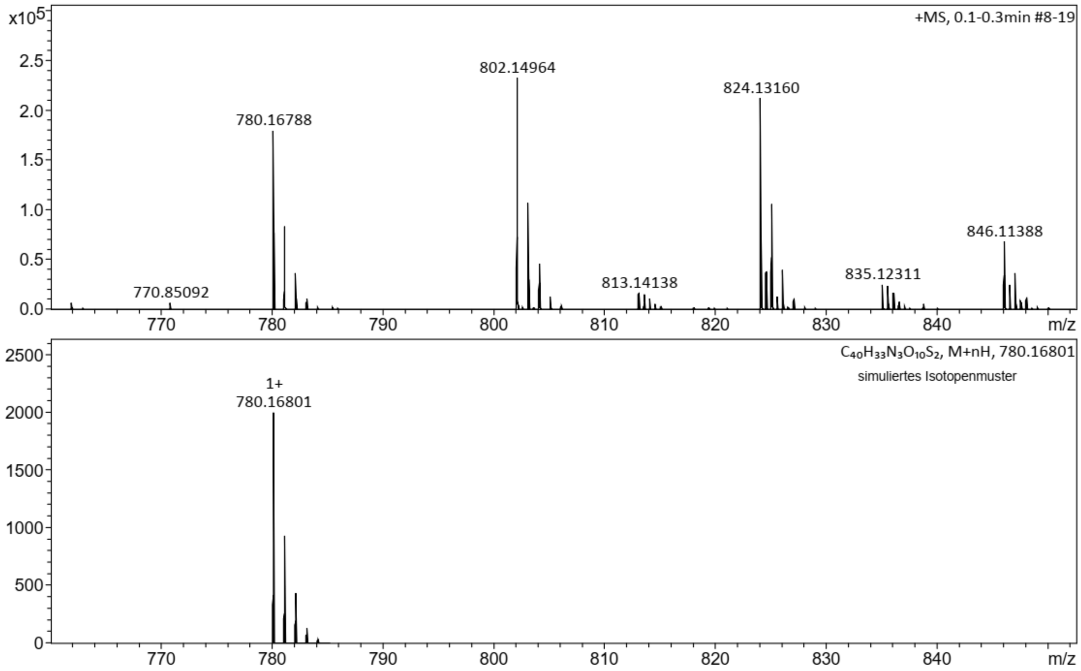

Figure S190: HR-MS of Smoc-L-His(Trt)-OH **14** (M measured=780.16788 [M+H]$^{+}$, M calc.=780.16801).

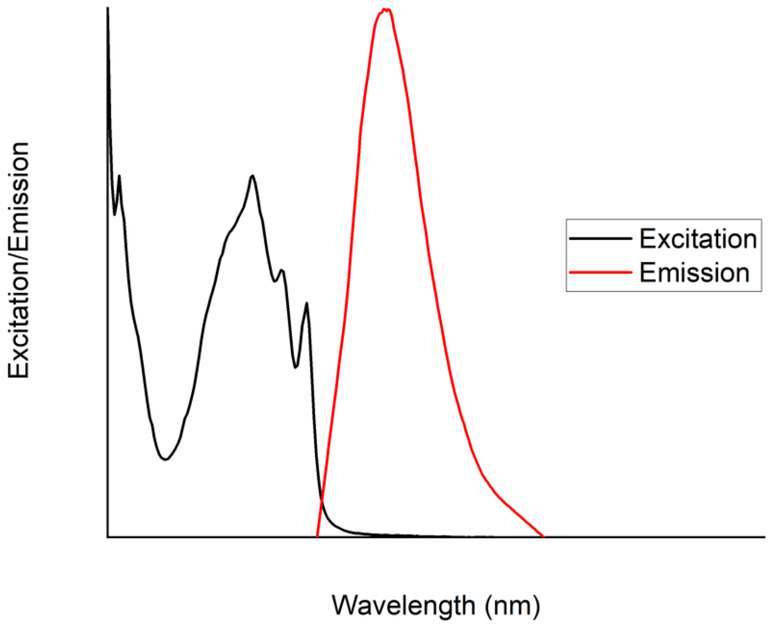

Figure S191: Excitation and emission spectra of Smoc-L-His(Trt)-OH **14**, excitation and emission have been normalized between 0 and 1 for illustration.

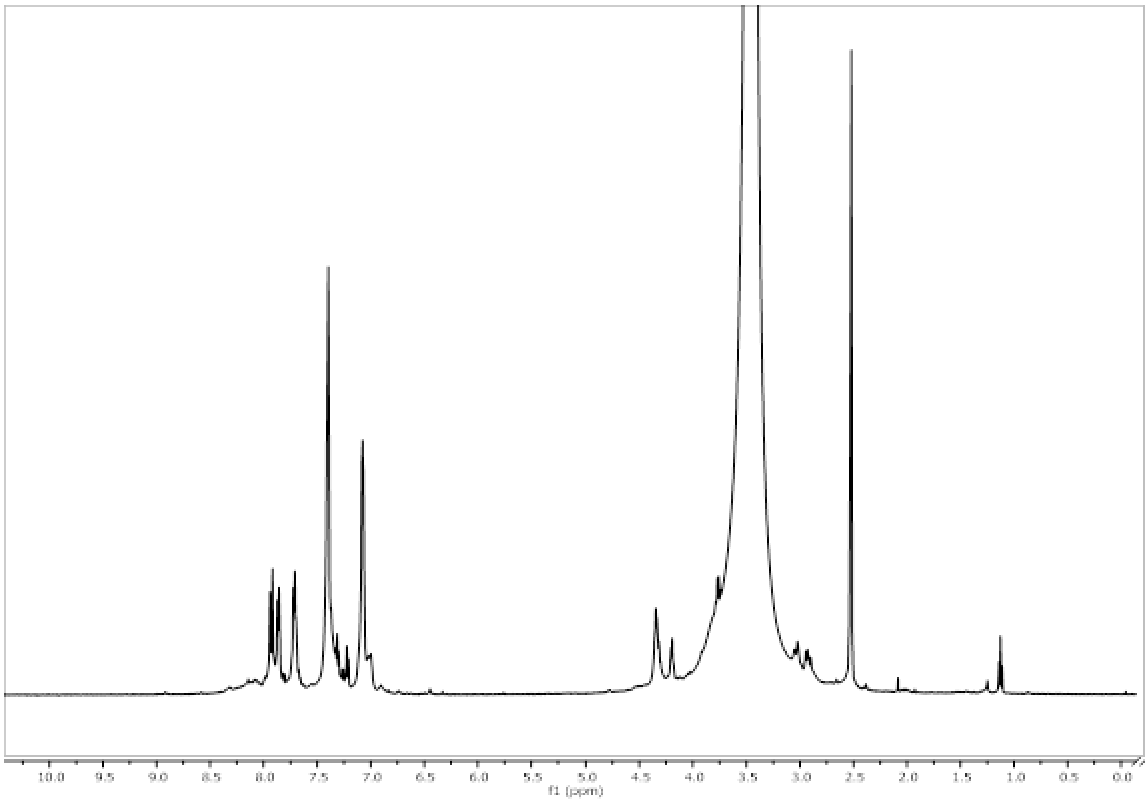

Figure S192: ^{1}H-NMR of Smoc-L-His(Trt)-OH **14**.

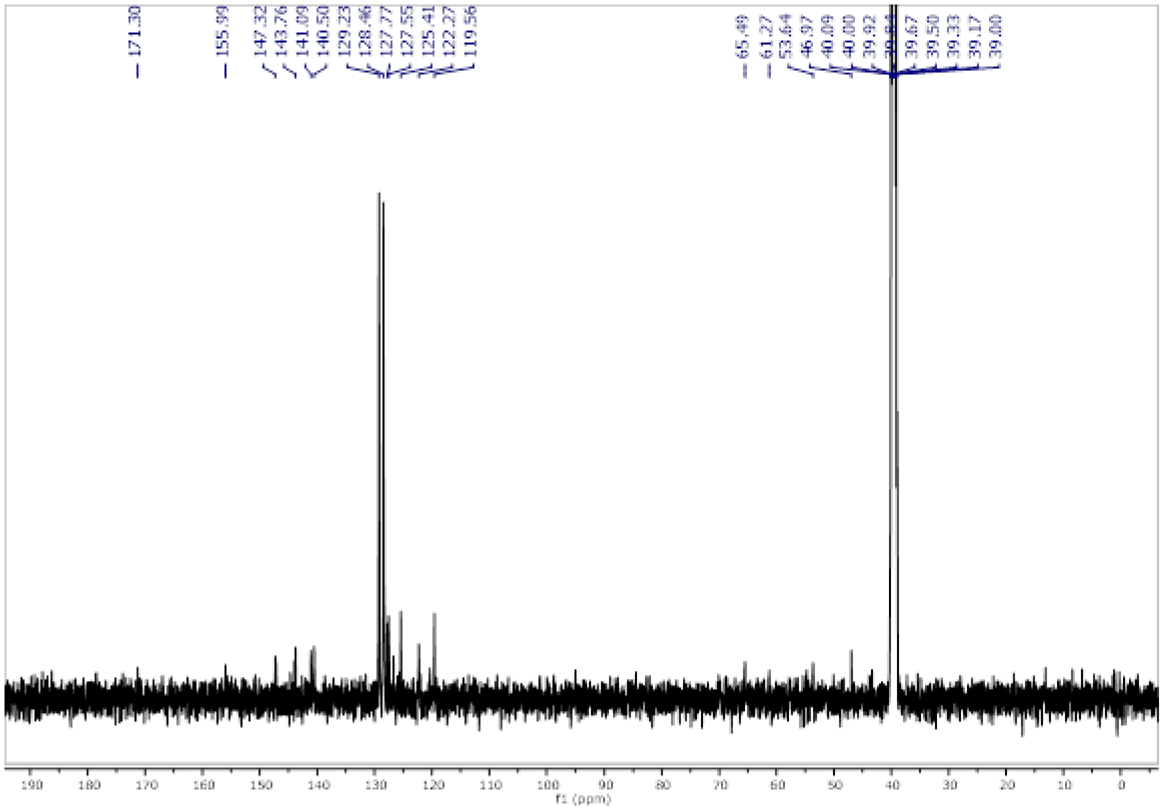

Figure S193: ^{13}C-NMR of Smoc-L-His(Trt)-OH **14**.

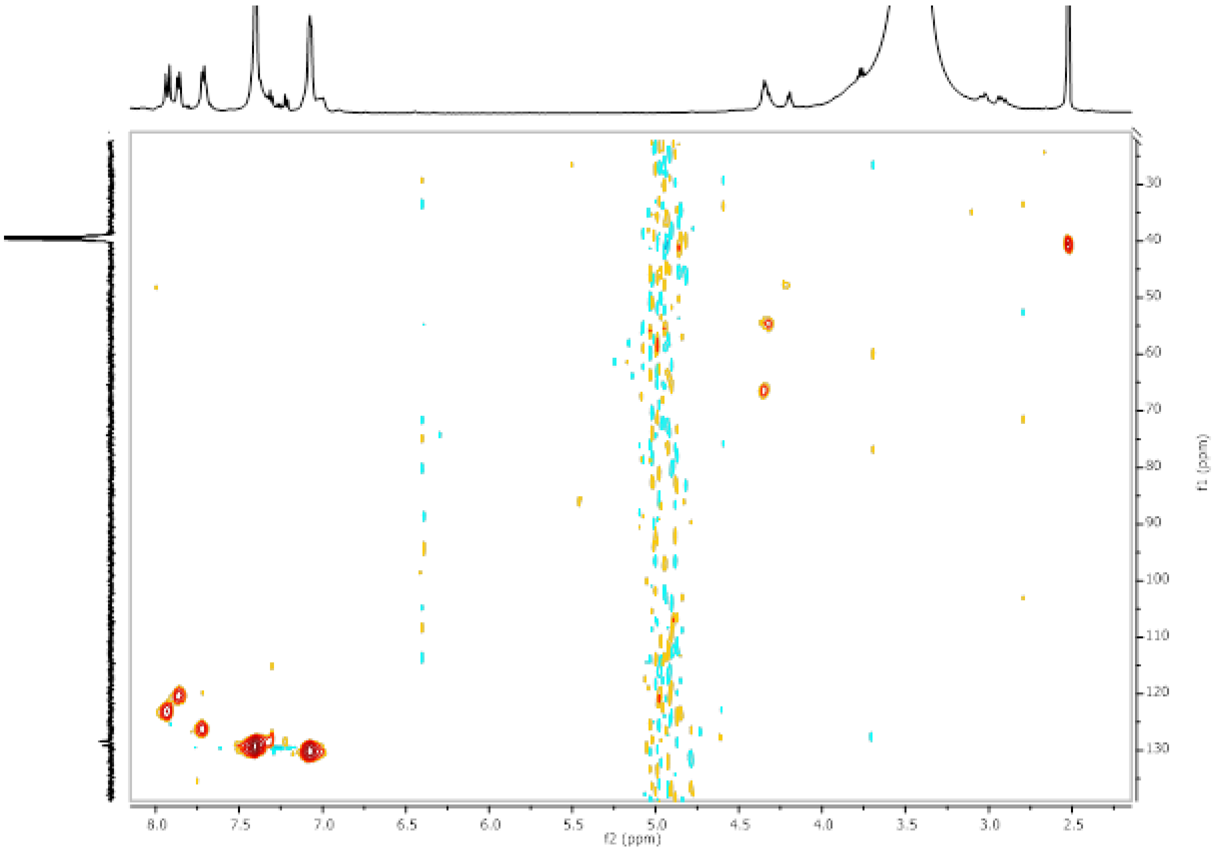

Figure S194: ^{1}H-^{13}C HSQC-NMR of Smoc-L-His(Trt)-OH **14**.

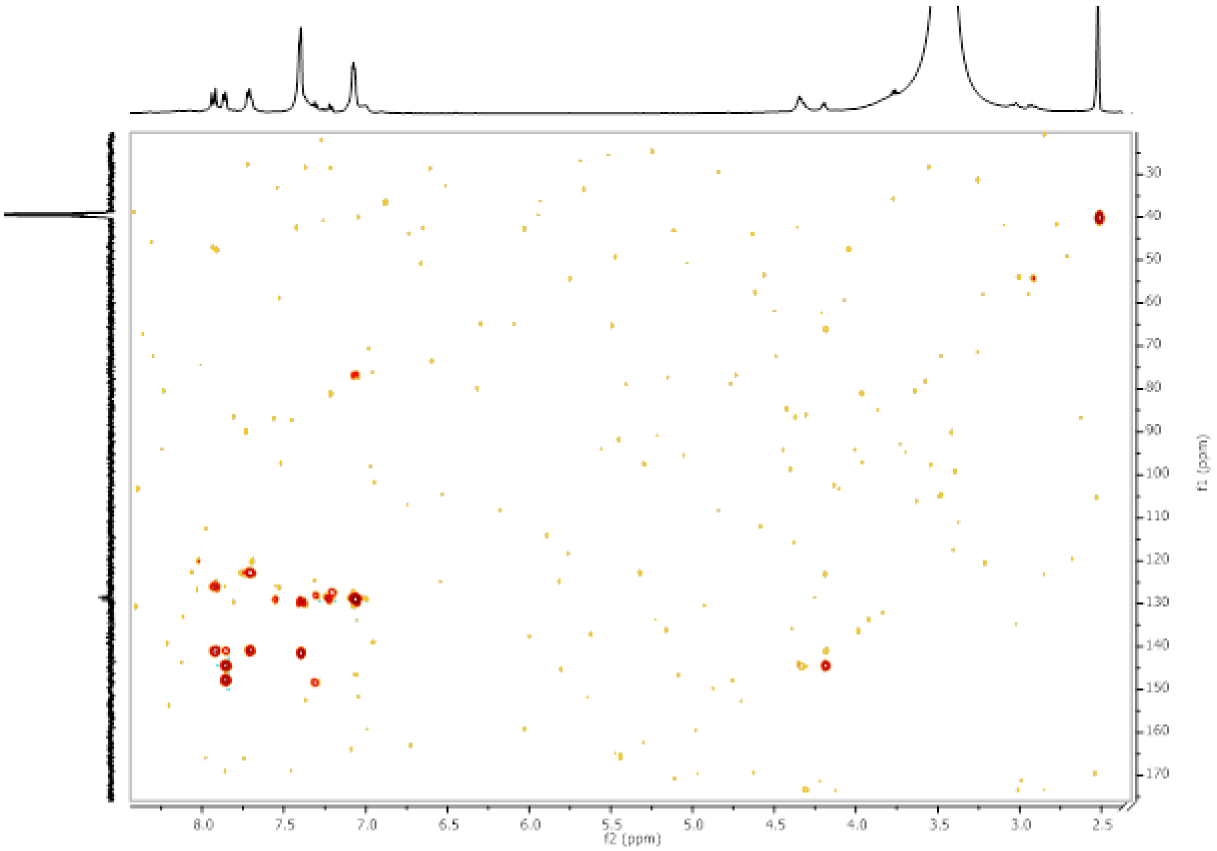

Figure S195: ^{1}H-^{13}C HMBC-NMR of Smoc-L-His(Trt)-OH **14**.

3.2.13. Analytical data of Smoc-L-Ile-OH 15

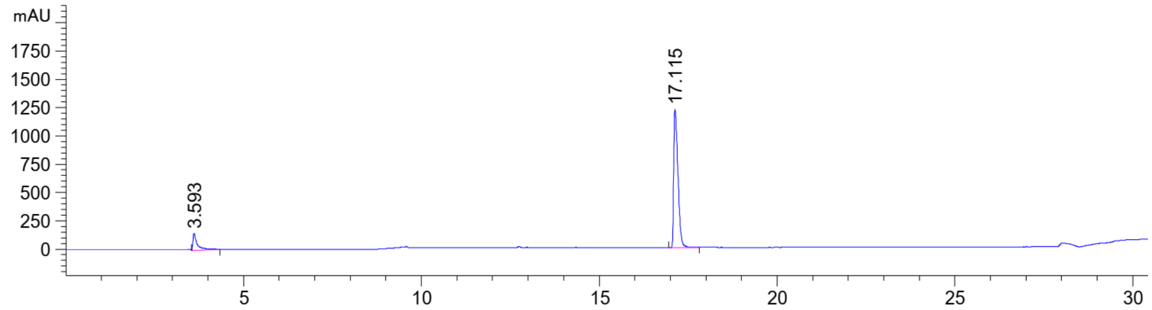

Figure S196: HPLC chromatogram of Smoc-L-Ile-OH **15** at λ=220 nm (0to40 MeCN).

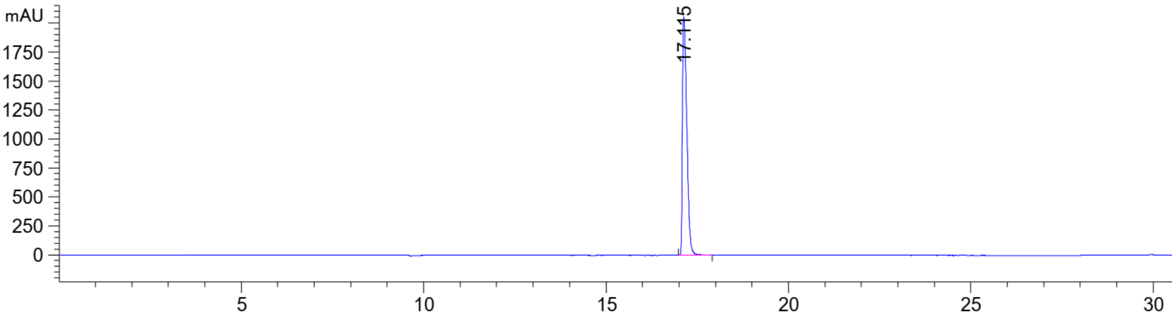

Figure S197: HPLC chromatogram of Smoc-L-Ile-OH **15** at λ=280 nm (0to40 MeCN).

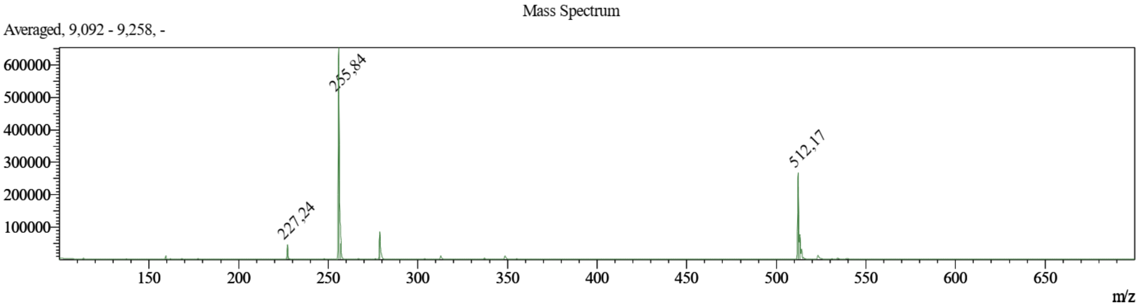

Figure S198: ESI-MS of Smoc-L-Ile-OH **15** (M measured=512.17 [M-H]$^-$, M calc.=513.53).

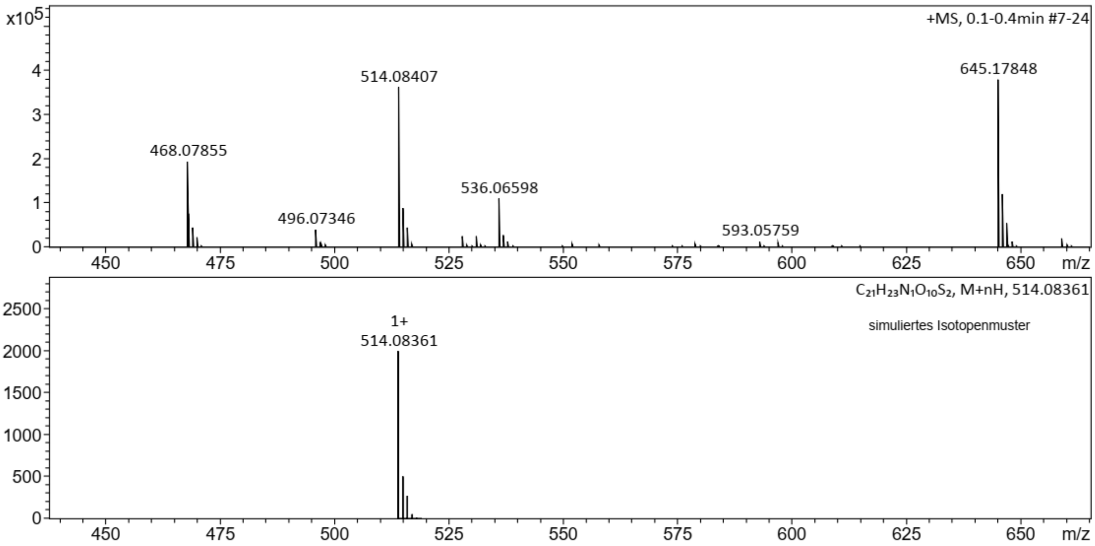

Figure S199: HR-MS of Smoc-L-Ile-OH **15** (M measured=514.08407 [M+H]$^+$, M calc.=514.08361).

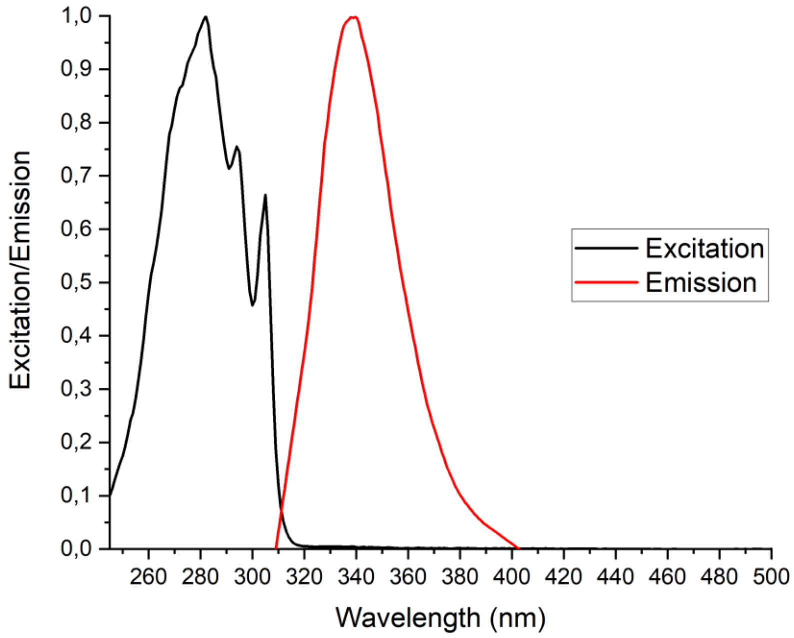

Figure S200: Excitation and emission spectra of Smoc-L-Ile-OH **15**, excitation and emission have been normalized between 0 and 1 for illustration.

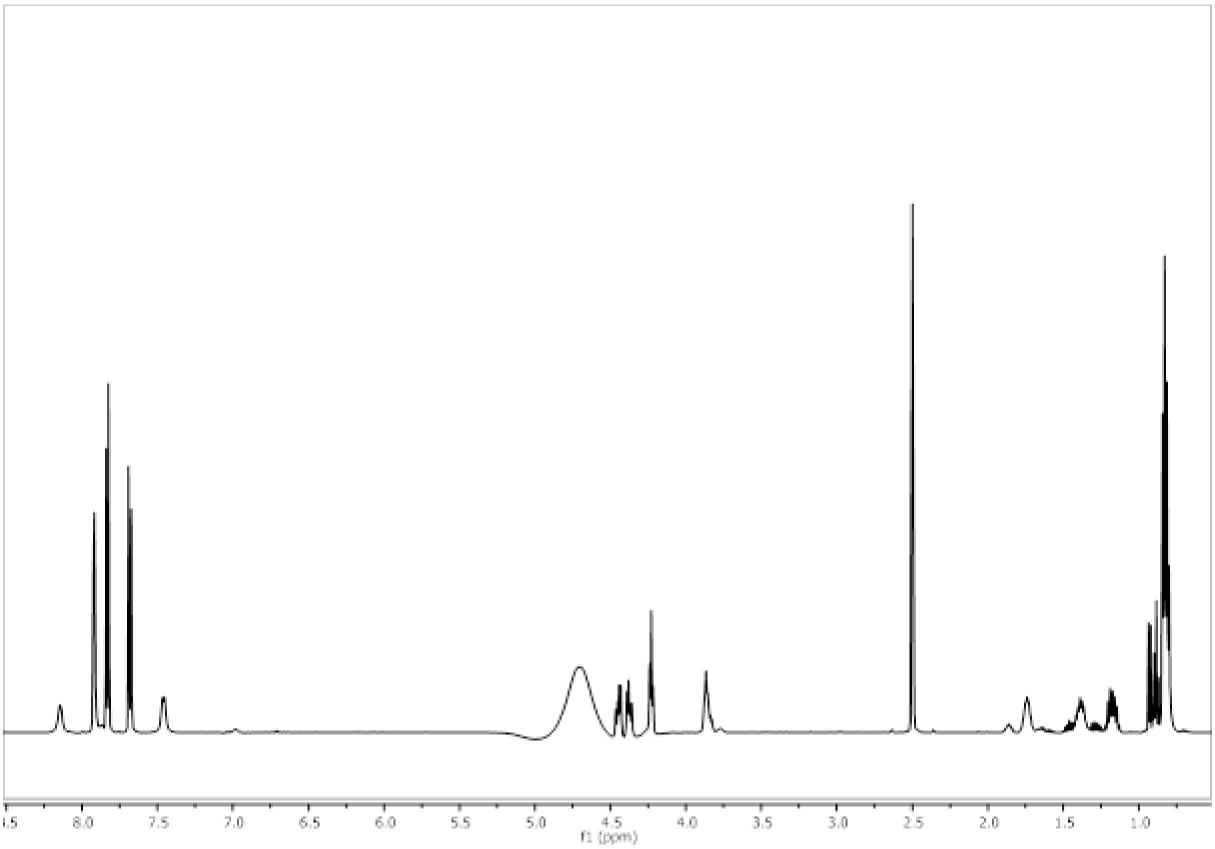

Figure S201: ^{1}H-NMR of Smoc-L-Ile-OH **15**.

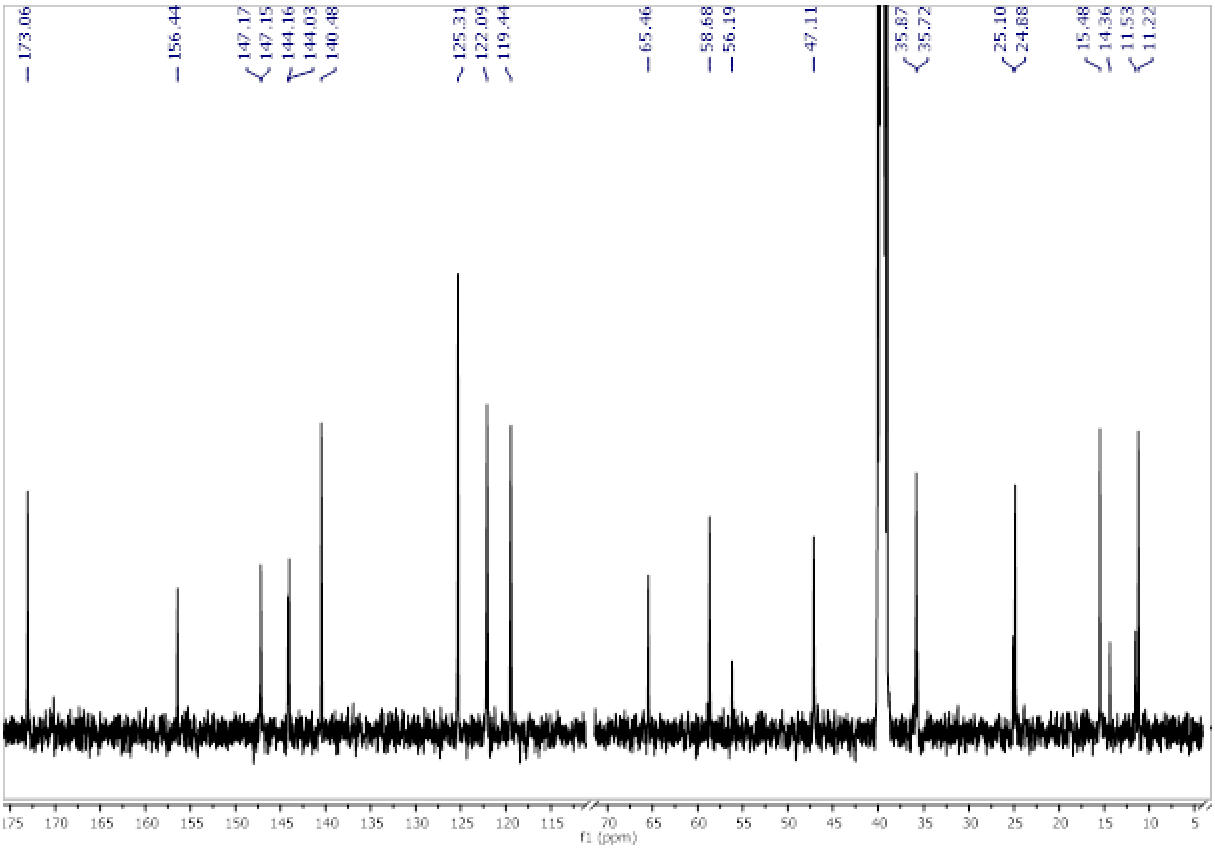

Figure S202: ^{13}C-NMR of Smoc-L-Ile-OH **15**.

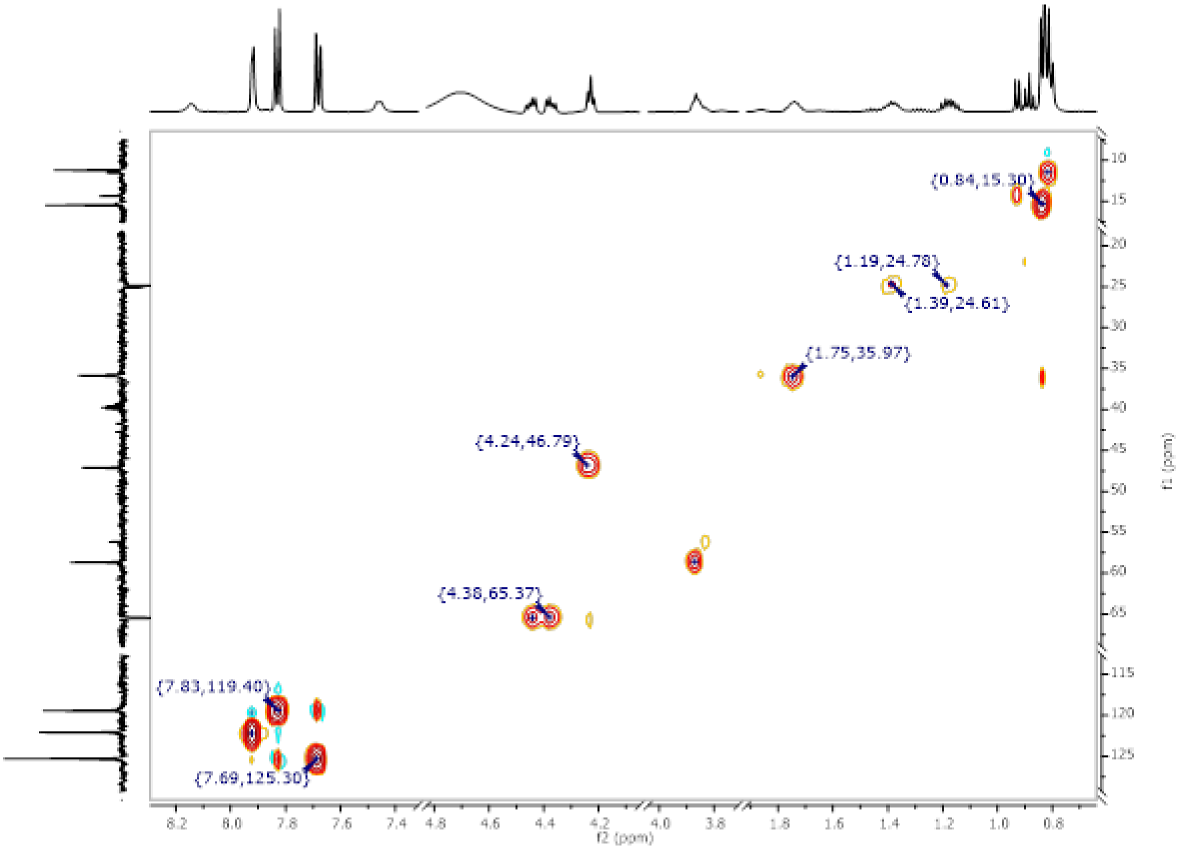

Figure S203: ^{1}H-^{13}C HSQC-NMR of Smoc-L-Ile-OH **15**.

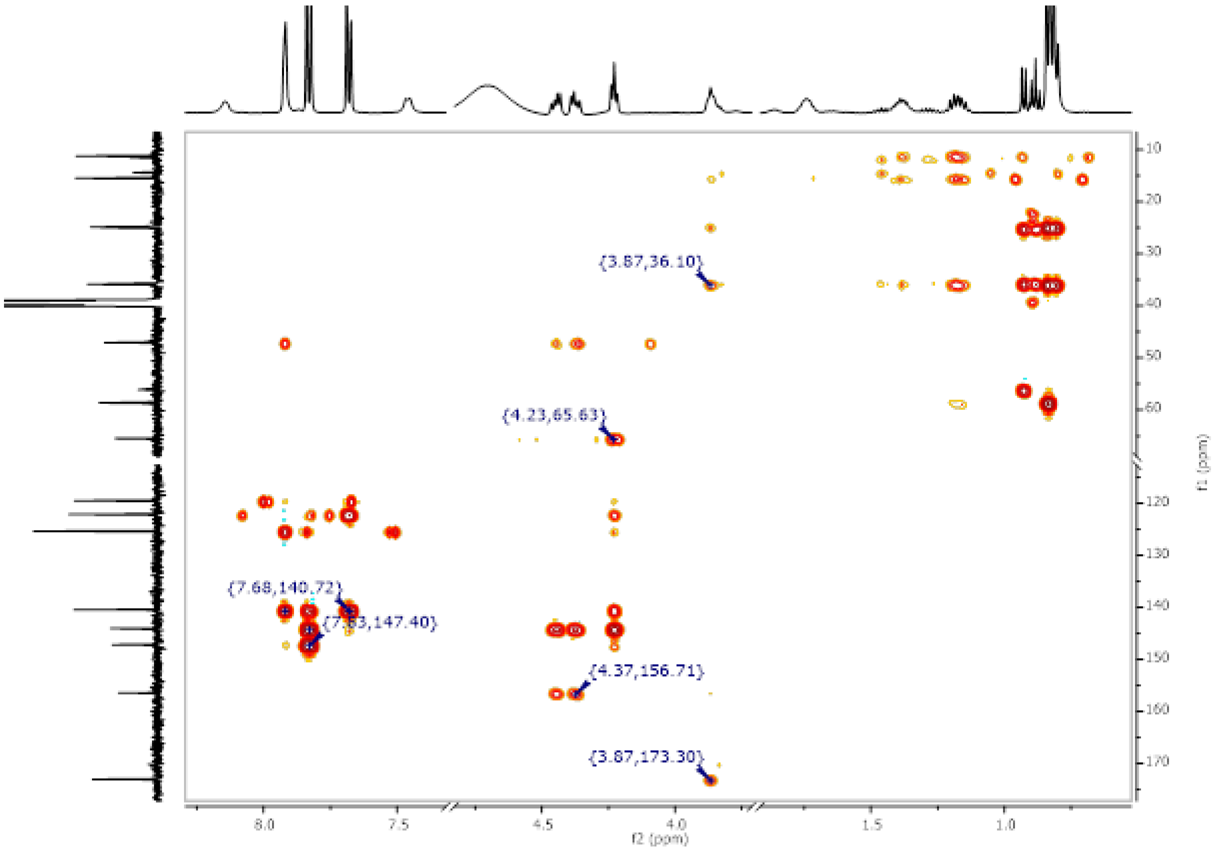

Figure S204: ^{1}H-^{13}C HMBC-NMR of Smoc-L-Ile-OH **15**.

3.2.14. Analytical data of Smoc-L-Leu-OH 16

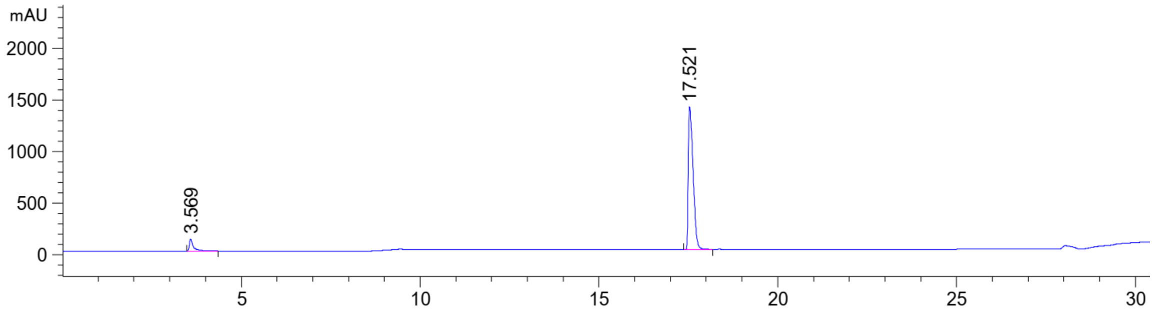

Figure S205: HPLC chromatogram of Smoc-L-Leu-OH **16** at λ=220 nm (0to40 MeCN).

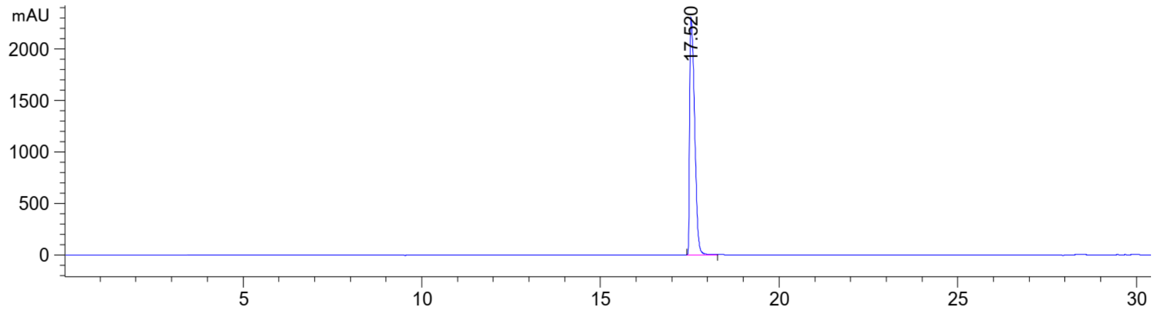

Figure S206: HPLC chromatogram of Smoc-L-Leu-OH **16** at λ=280 nm (0to40 MeCN).

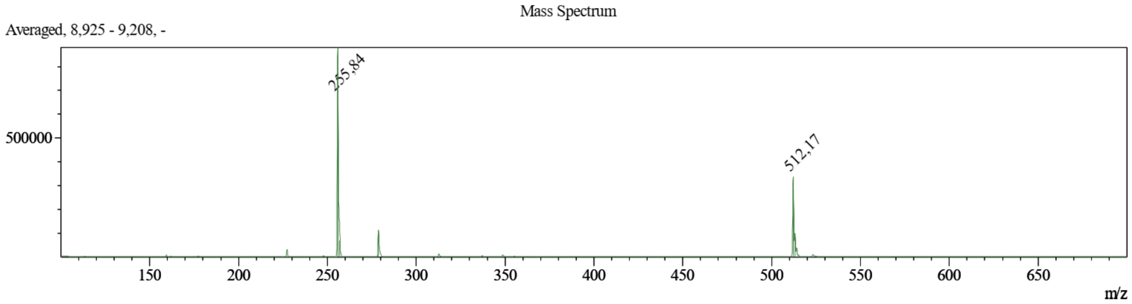

Figure S207: ESI-MS of Smoc-L-Leu-OH **16** (M measured=512.17 [M-H]-, M calc.=513.53).

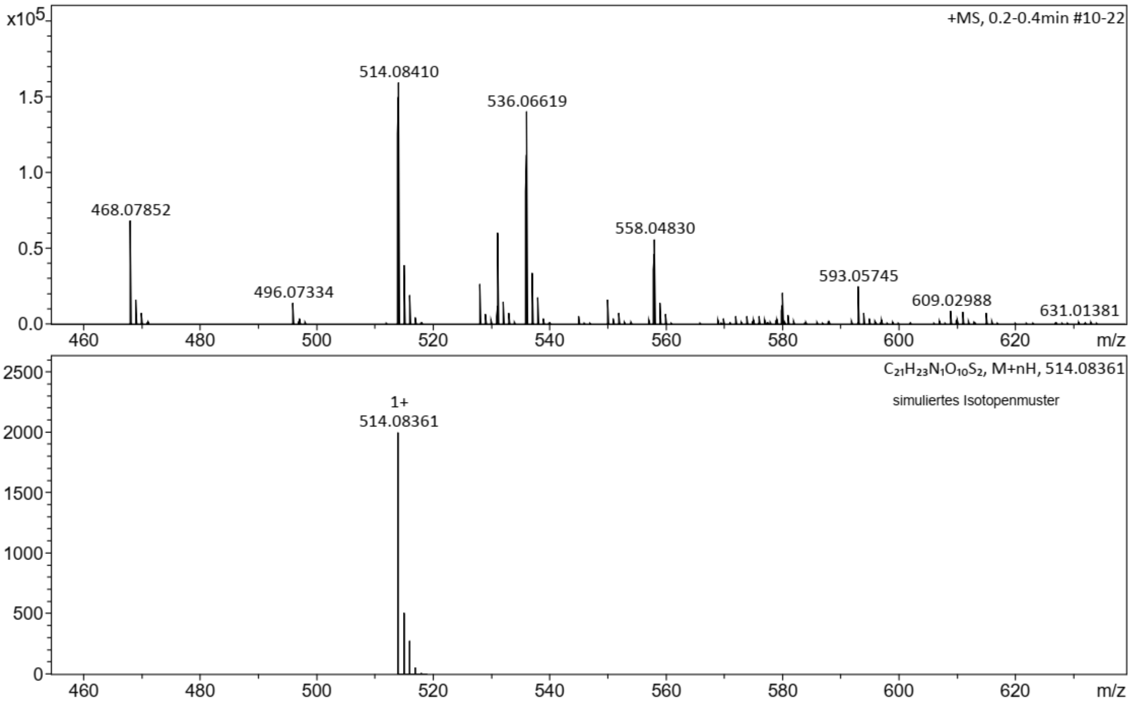

Figure S208: HR-MS of Smoc-L-Leu-OH **16** (M measured=514.08410 [M+H]+, M calc.=514.08361).

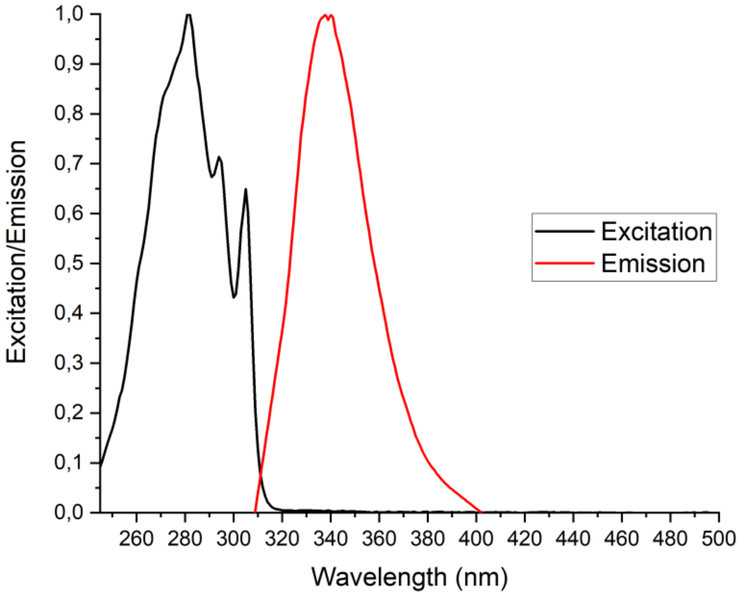

Figure S209: Excitation and emission spectra of Smoc-L-Leu-OH **16**, excitation and emission have been normalized between 0 and 1 for illustration.

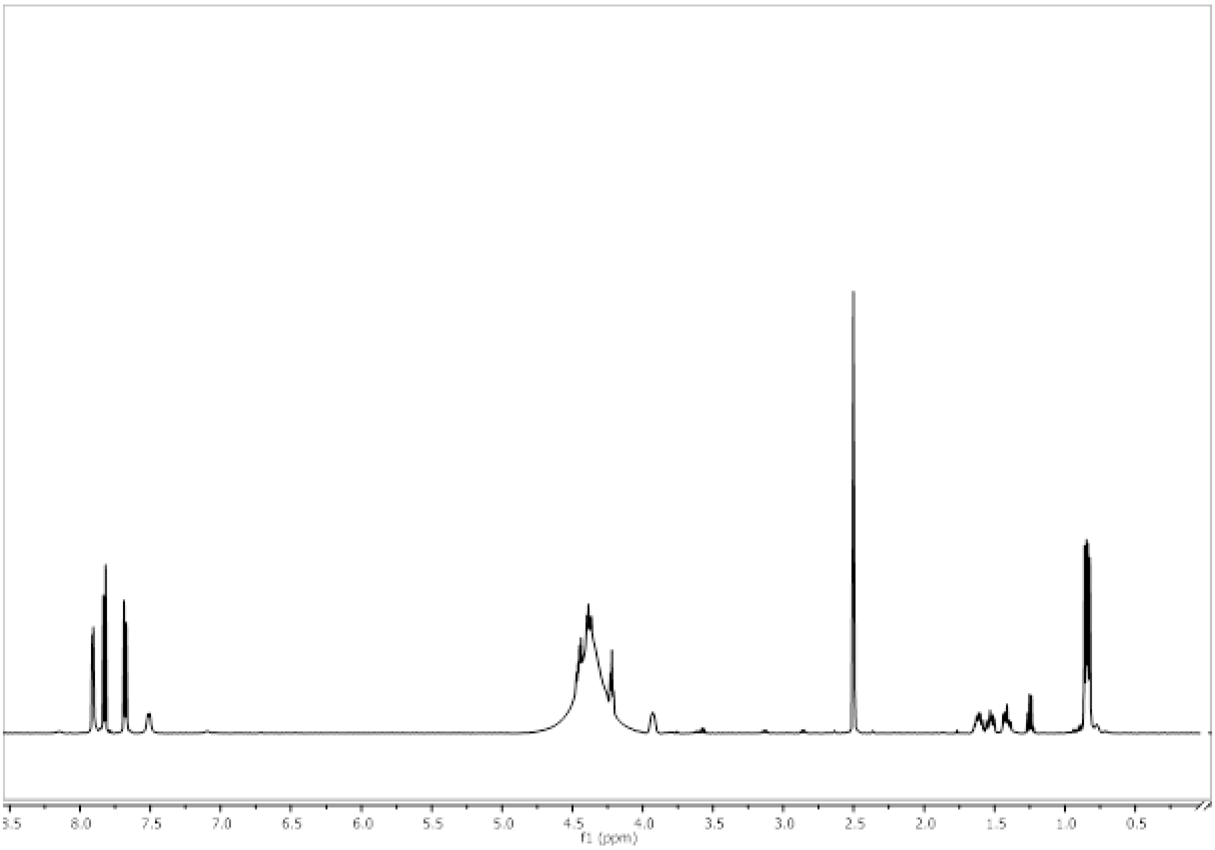

Figure S210: ^{1}H-NMR of Smoc-L-Leu-OH **16**.

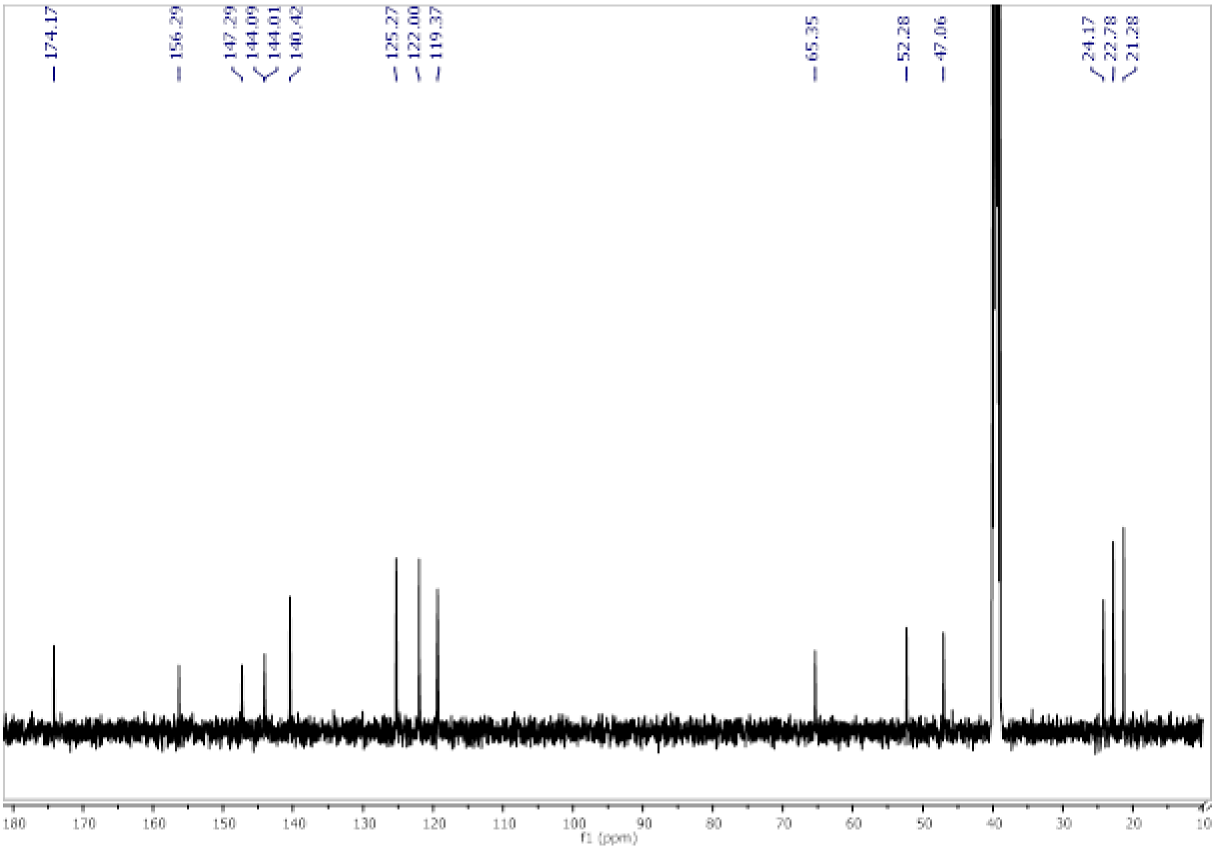

Figure S211: ^{13}C-NMR of Smoc-L-Leu-OH **16**.

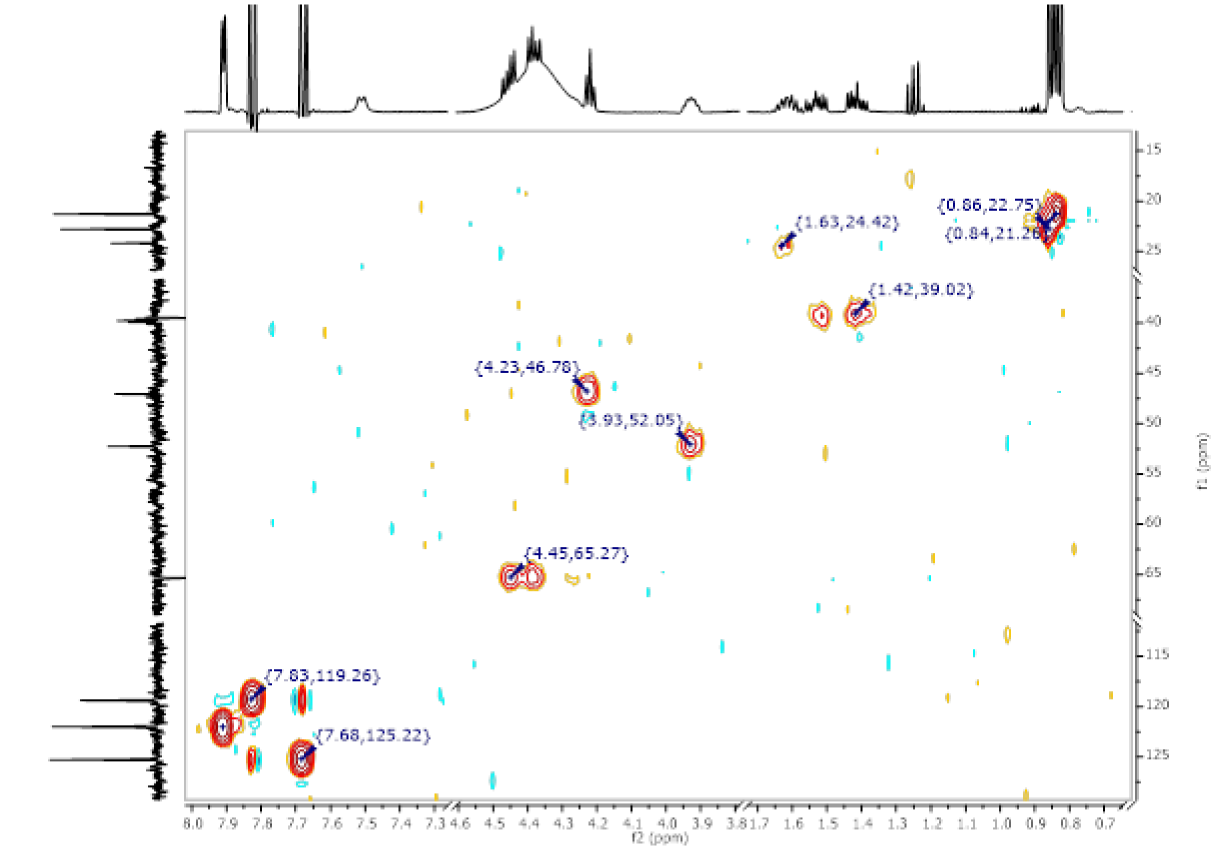

Figure S212: ^{1}H-^{13}C HSQC-NMR of Smoc-L-Leu-OH **16**.

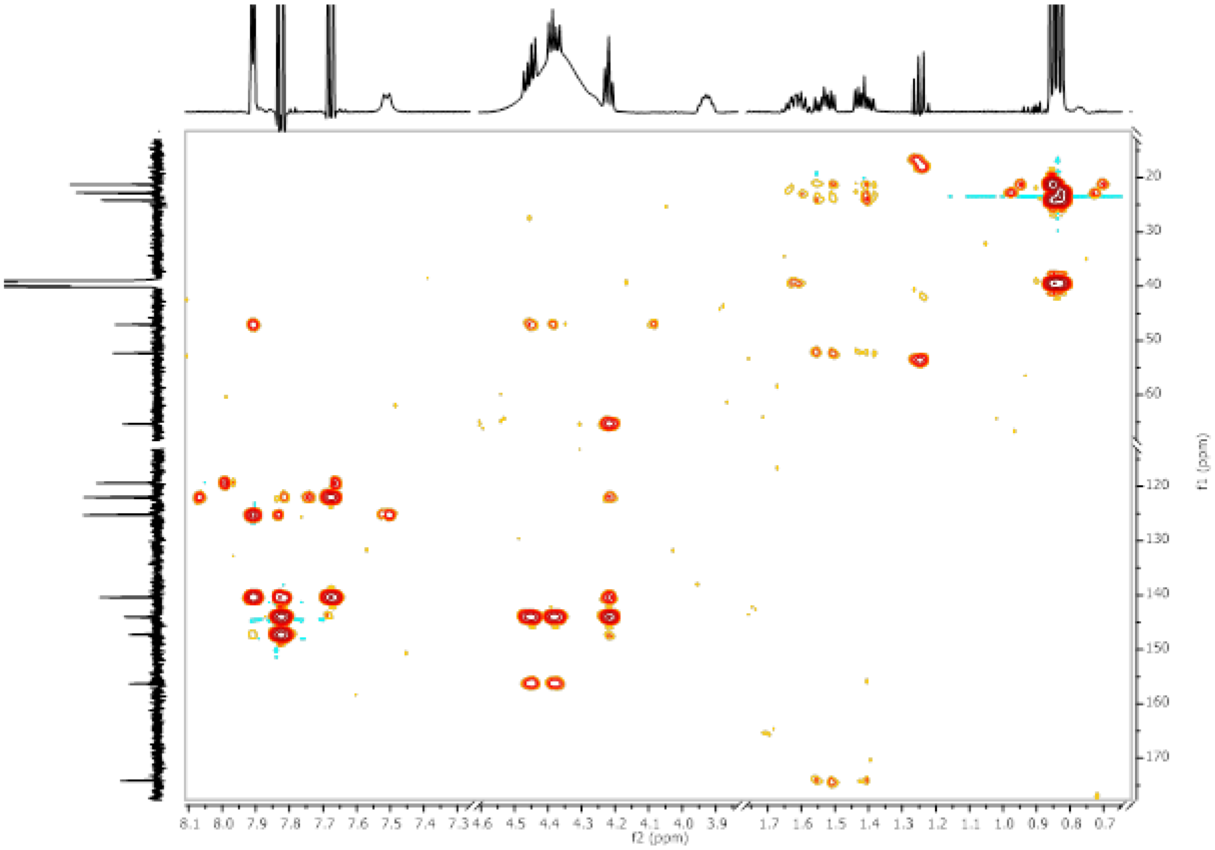

Figure S213: ^{1}H-^{13}C HMBC-NMR of Smoc-L-Leu-OH **16**.

3.2.15. Analytical data of Smoc-D-Leu-OH 17

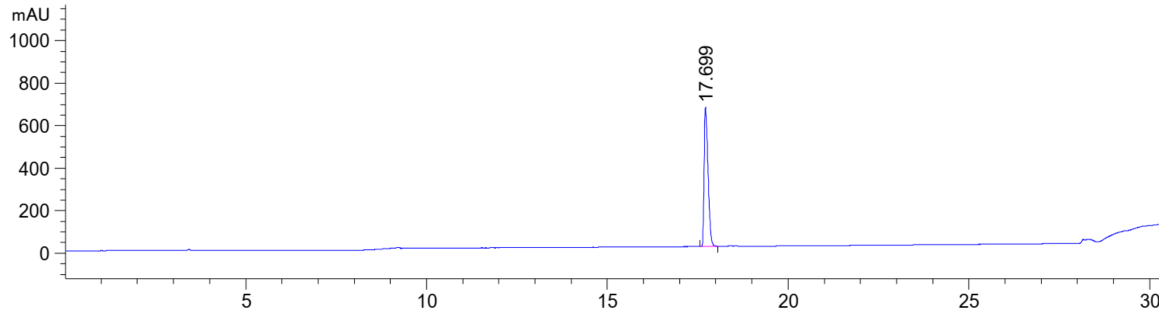

Figure S214: HPLC chromatogram of Smoc-D-Leu-OH **17** at λ=220 nm (0to40 MeCN).

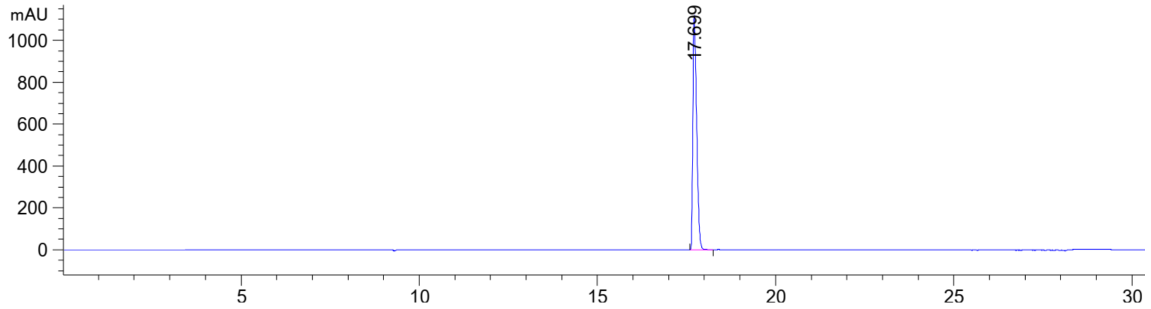

Figure S215: HPLC chromatogram of Smoc-D-Leu-OH **17** at λ=280 nm (0to40 MeCN).

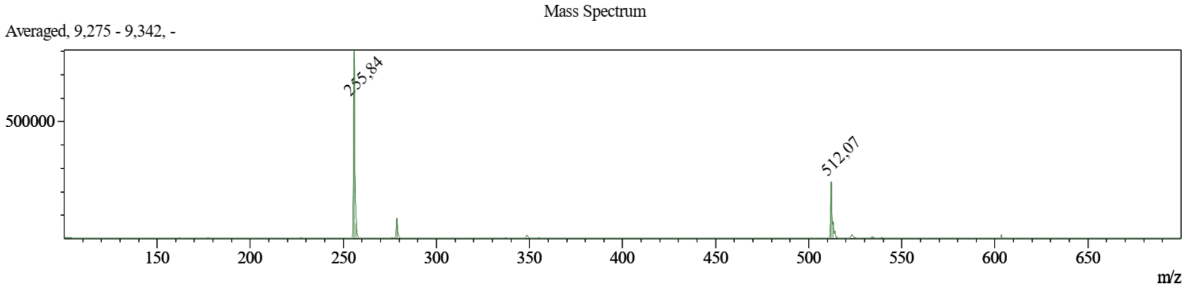

Figure S216: ESI-MS of Smoc-D-Leu-OH **17** (M measured=512.07 [M-H]$^-$, M calc.=513.53).

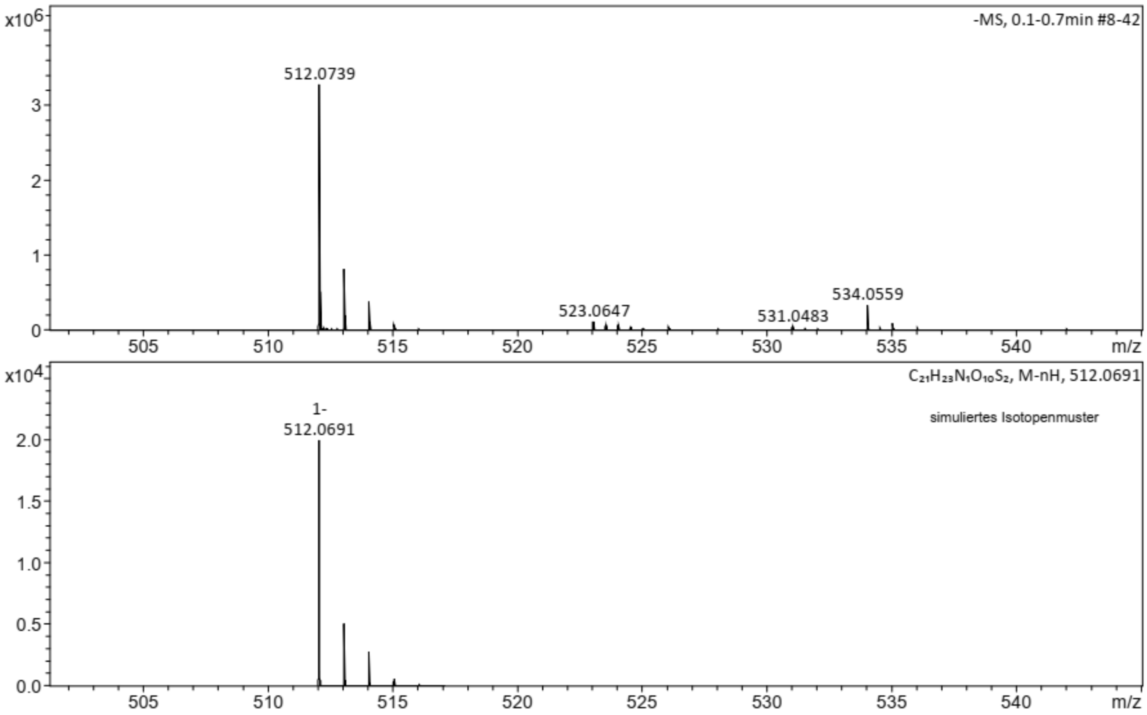

Figure S217: HR-MS of Smoc-D-Leu-OH **17** (M measured=512.0739 [M+H]$^+$, M calc.=512.0691).

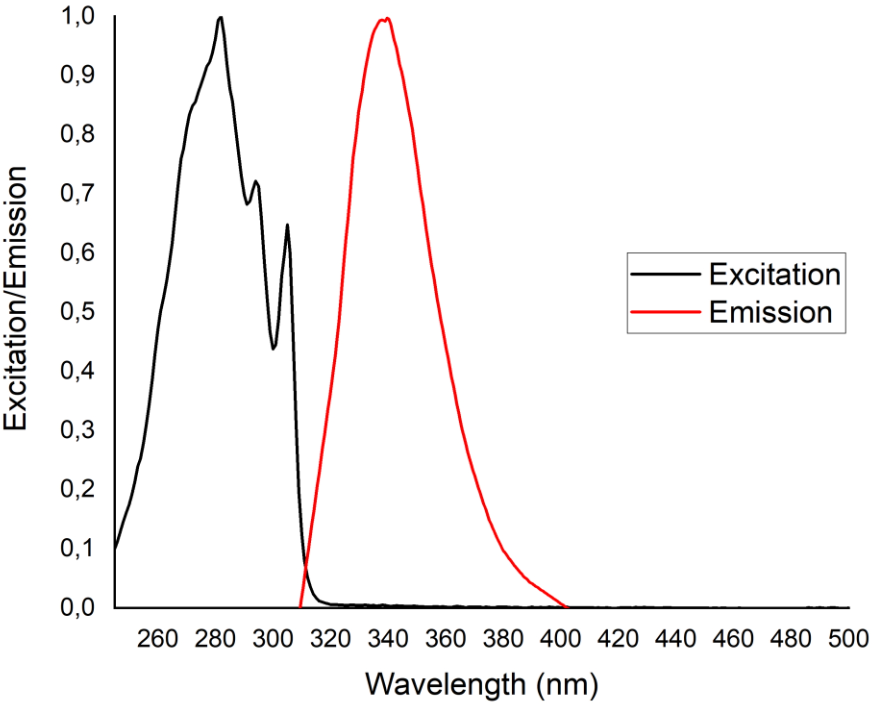

Figure S218: Excitation and emission spectra of Smoc-D-Leu-OH **17**, excitation and emission have been normalized between 0 and 1 for illustration.

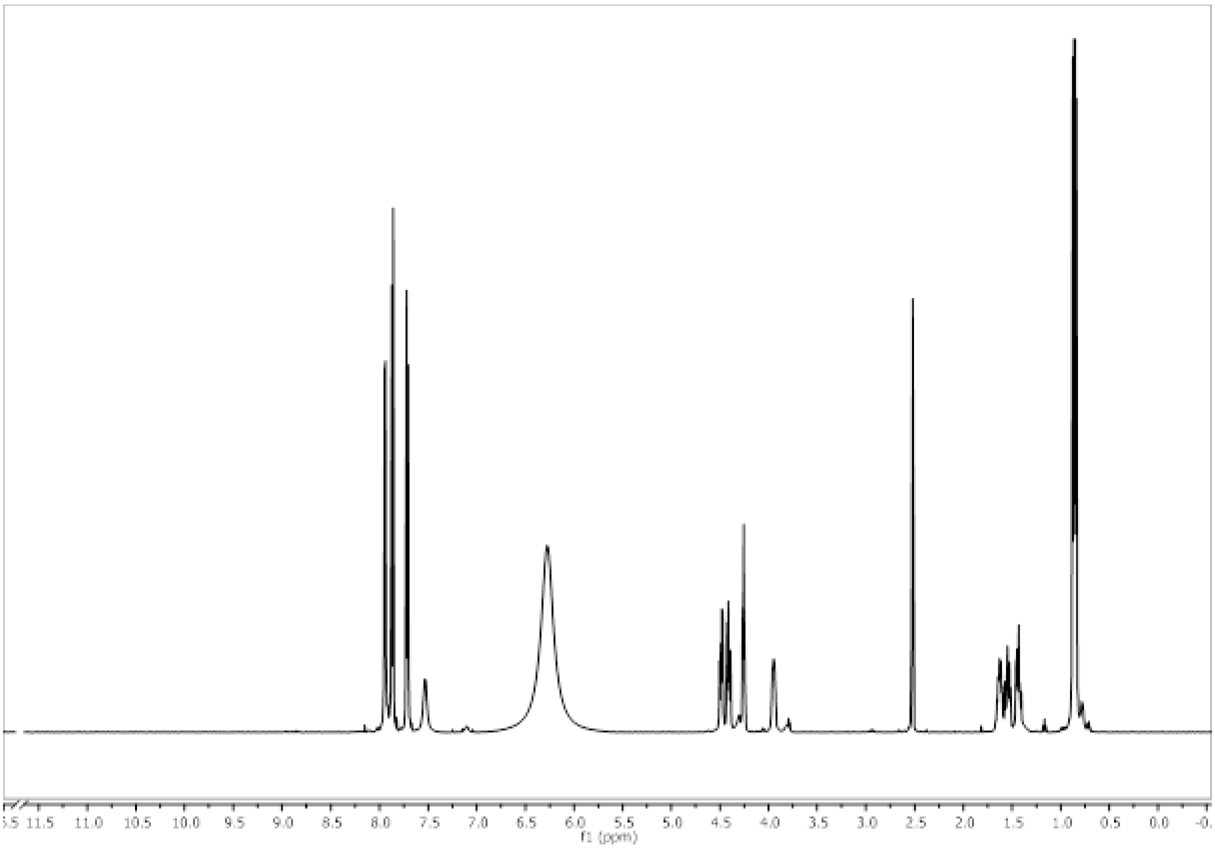

Figure S219: ^{1}H-NMR of Smoc-D-Leu-OH **17**.

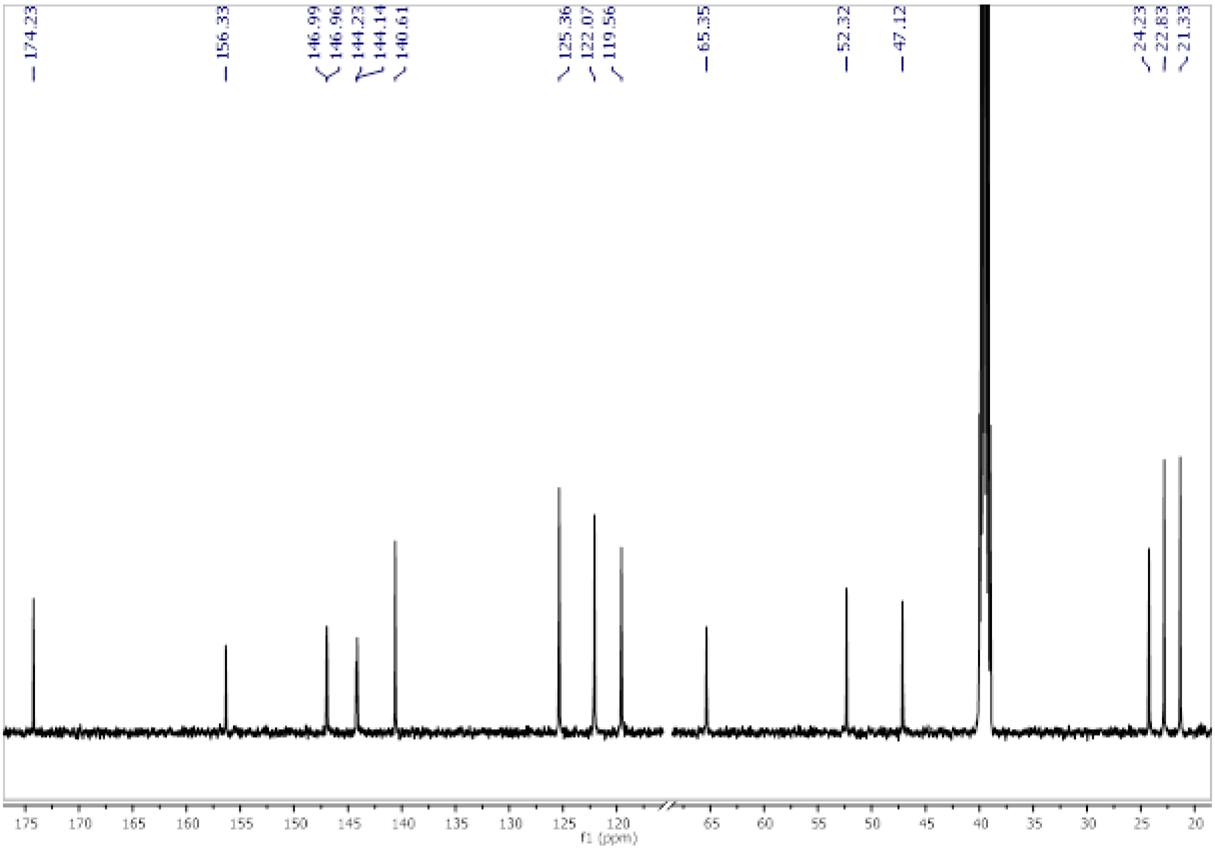

Figure S220: ^{13}C-NMR of Smoc-D-Leu-OH **17**.

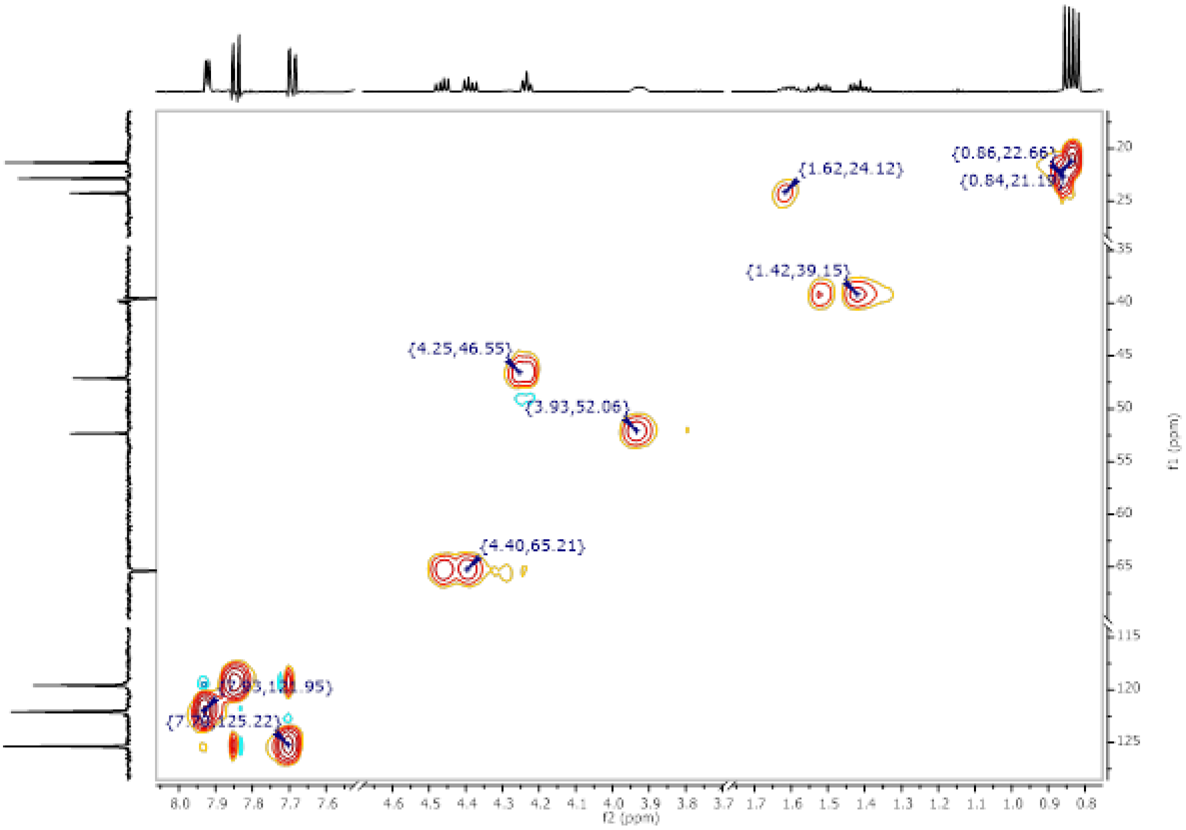

Figure S221: ^{1}H-^{13}C HSQC-NMR of Smoc-D-Leu-OH **17**.

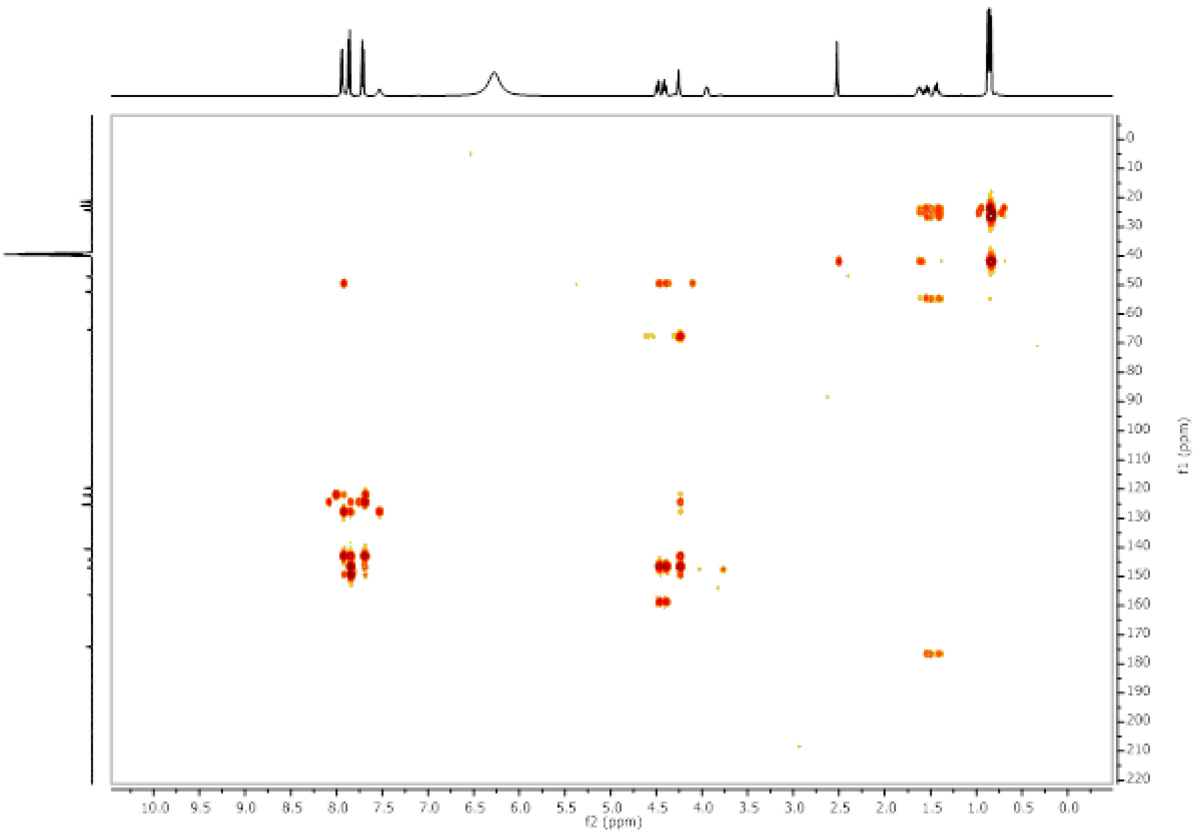

Figure S222: ^{1}H-^{13}C HMBC-NMR of Smoc-D-Leu-OH **17**.

3.2.16. Analytical data of Smoc-L-Lys(Boc)-OH 18

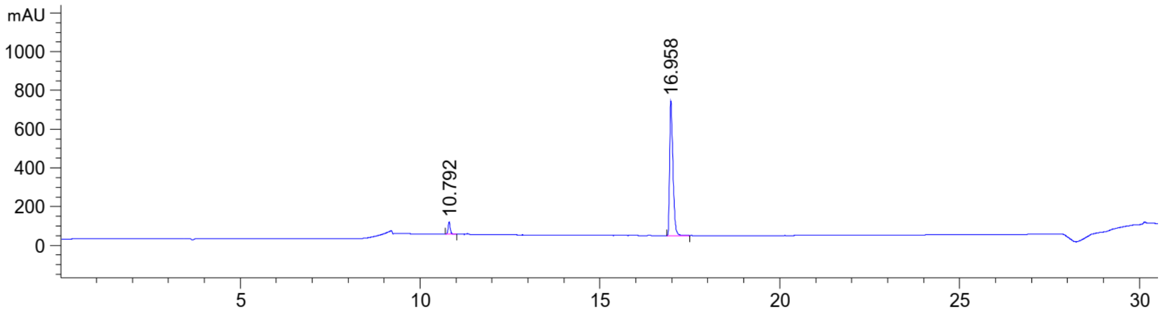

Figure S223: HPLC chromatogram of Smoc-L-Lys(Boc)-OH **18** at λ=220 nm (0to60 MeCN).

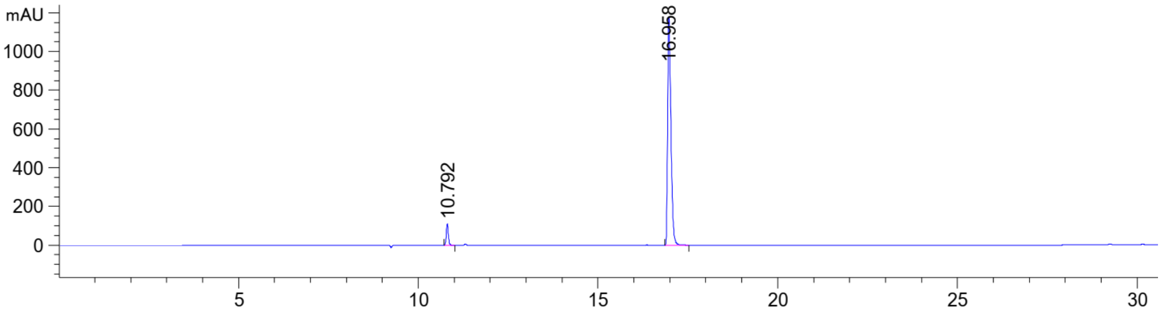

Figure S224: HPLC chromatogram of Smoc-L-Lys(Boc)-OH **18** at λ=280 nm (0to60 MeCN).

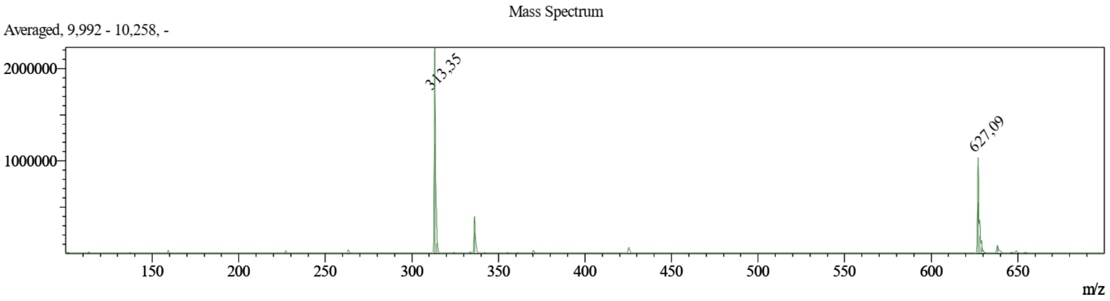

Figure S225: ESI-MS of Smoc-L-Lys(Boc)-OH **18** (M measured=627.09 [M-H]$^-$, M calc.=628.66).

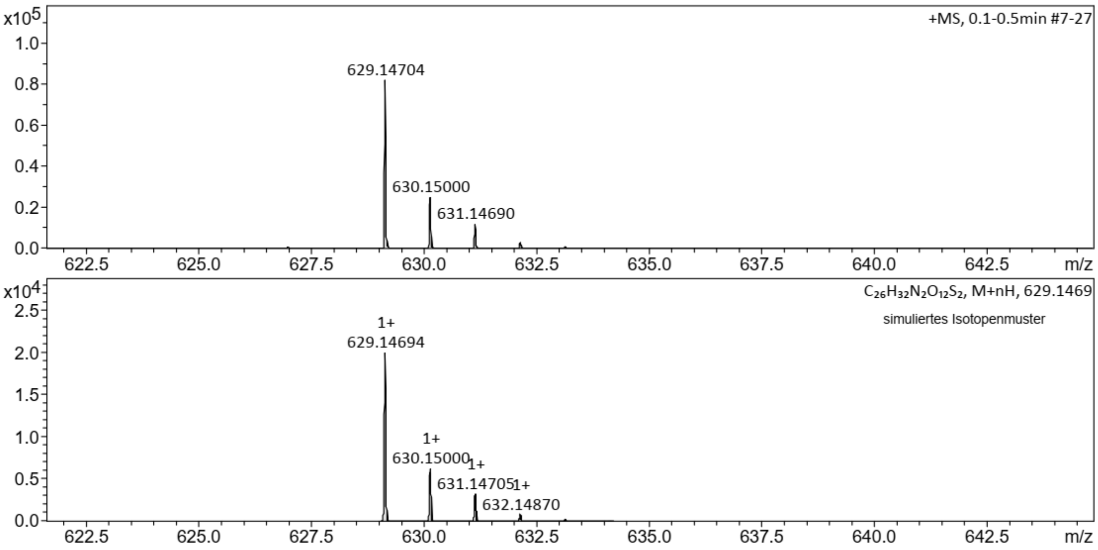

Figure S226: HR-MS of Smoc-L-Lys(Boc)-OH **18** (M measured= 629.14704 [M+H]$^+$, M calc.=629.14694).

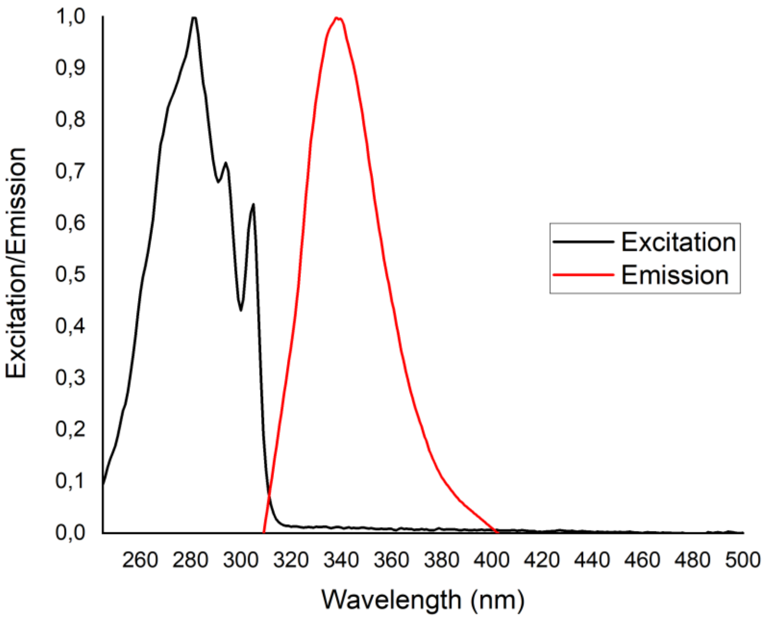

Figure S227: Excitation and emission spectra of Smoc-L-Lys(Boc)-OH **18**, excitation and emission have been normalized between 0 and 1 for illustration.

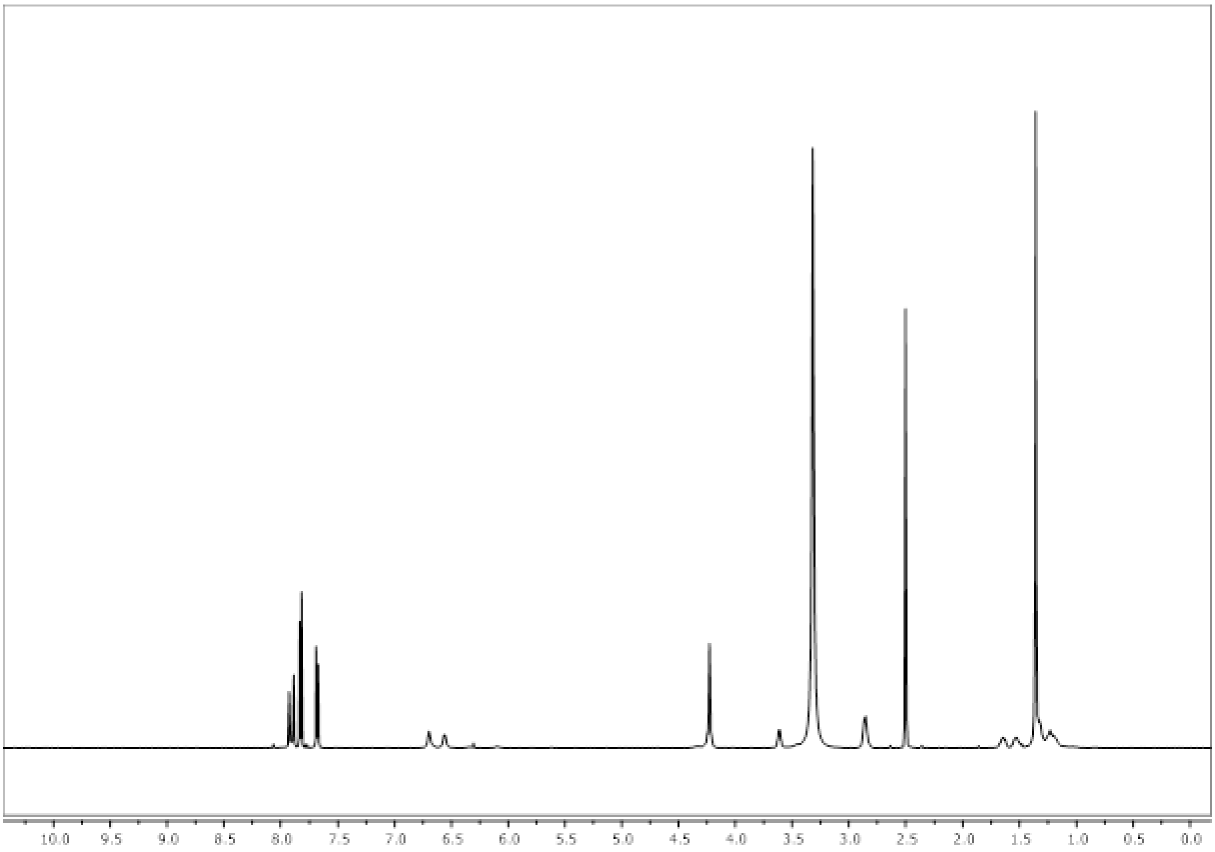

Figure S228: ^{1}H-NMR of Smoc-L-Lys(Boc)-OH **18**.

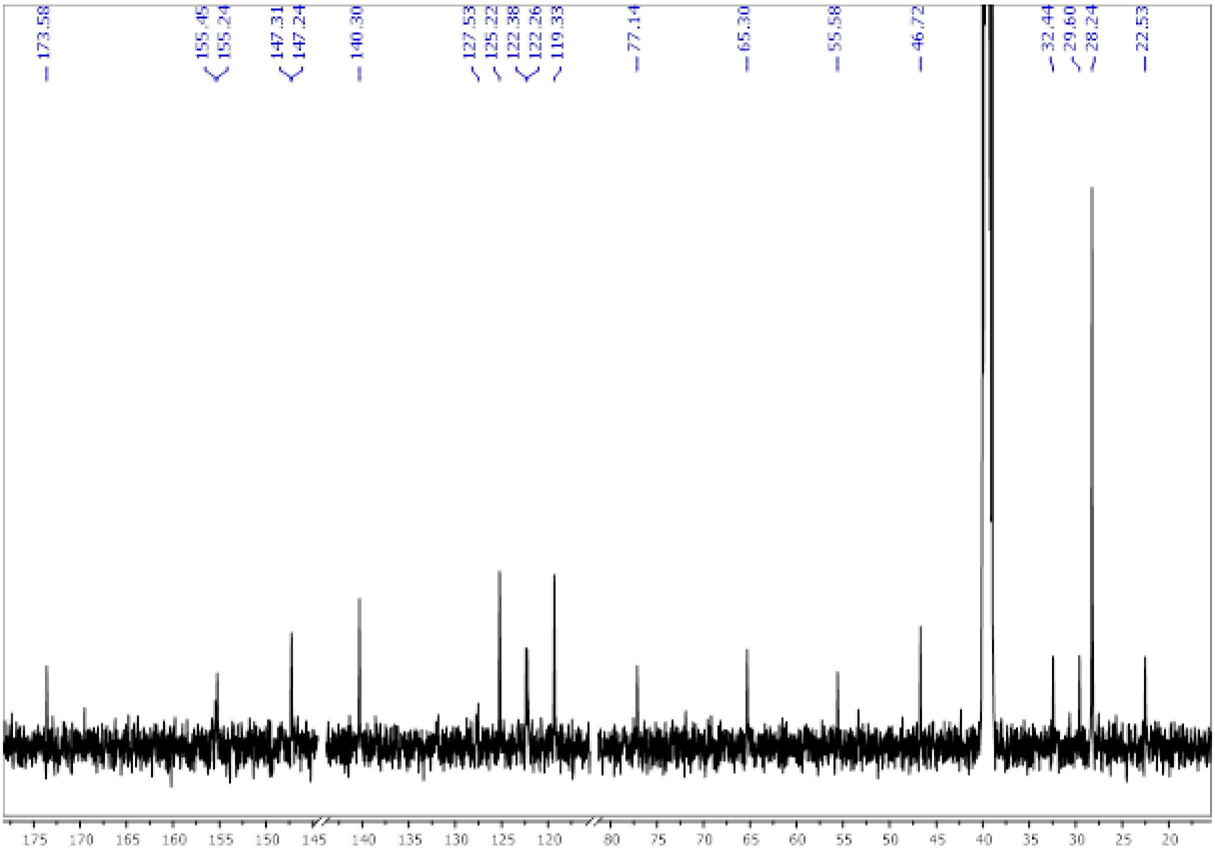

Figure S229: ^{13}C-NMR of Smoc-L-Lys(Boc)-OH **18**.

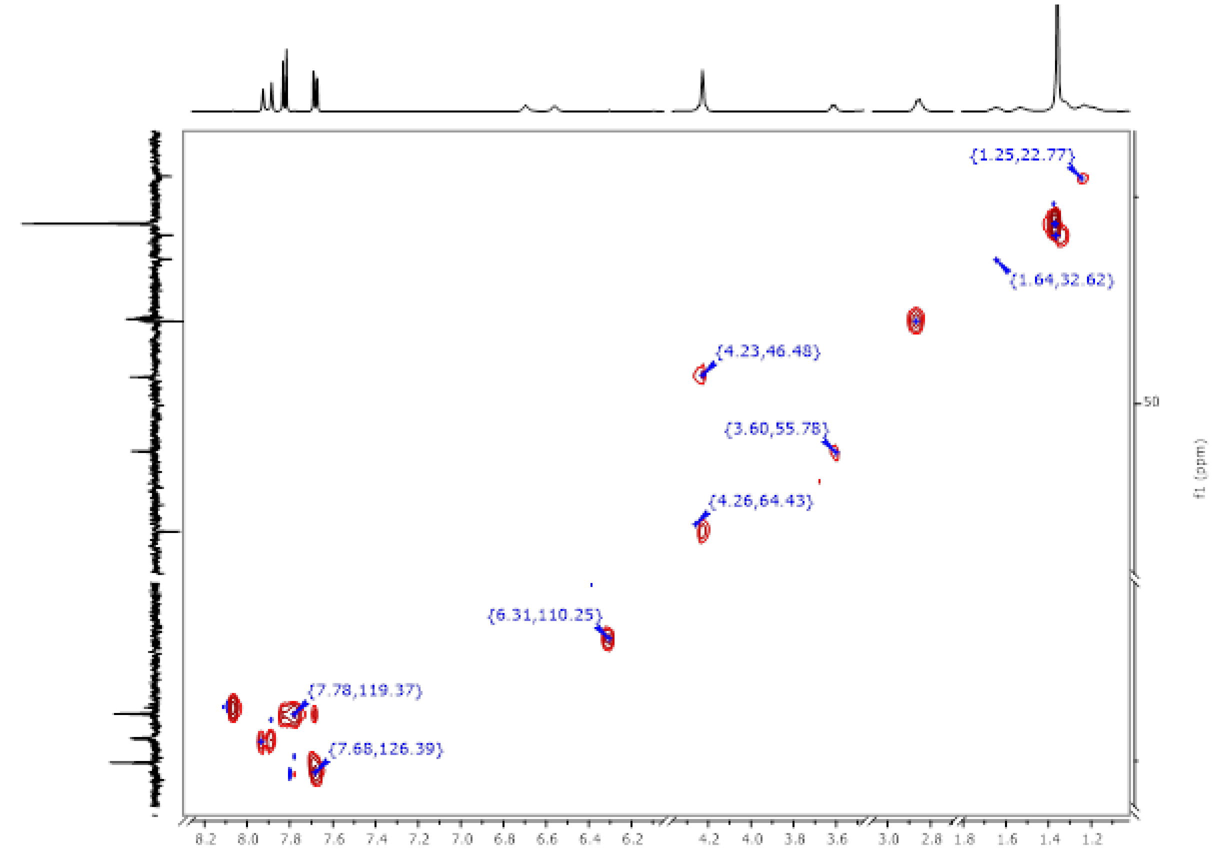

Figure S230: ^{1}H-^{13}C HSQC-NMR of Smoc-L-Lys(Boc)-OH **18**.

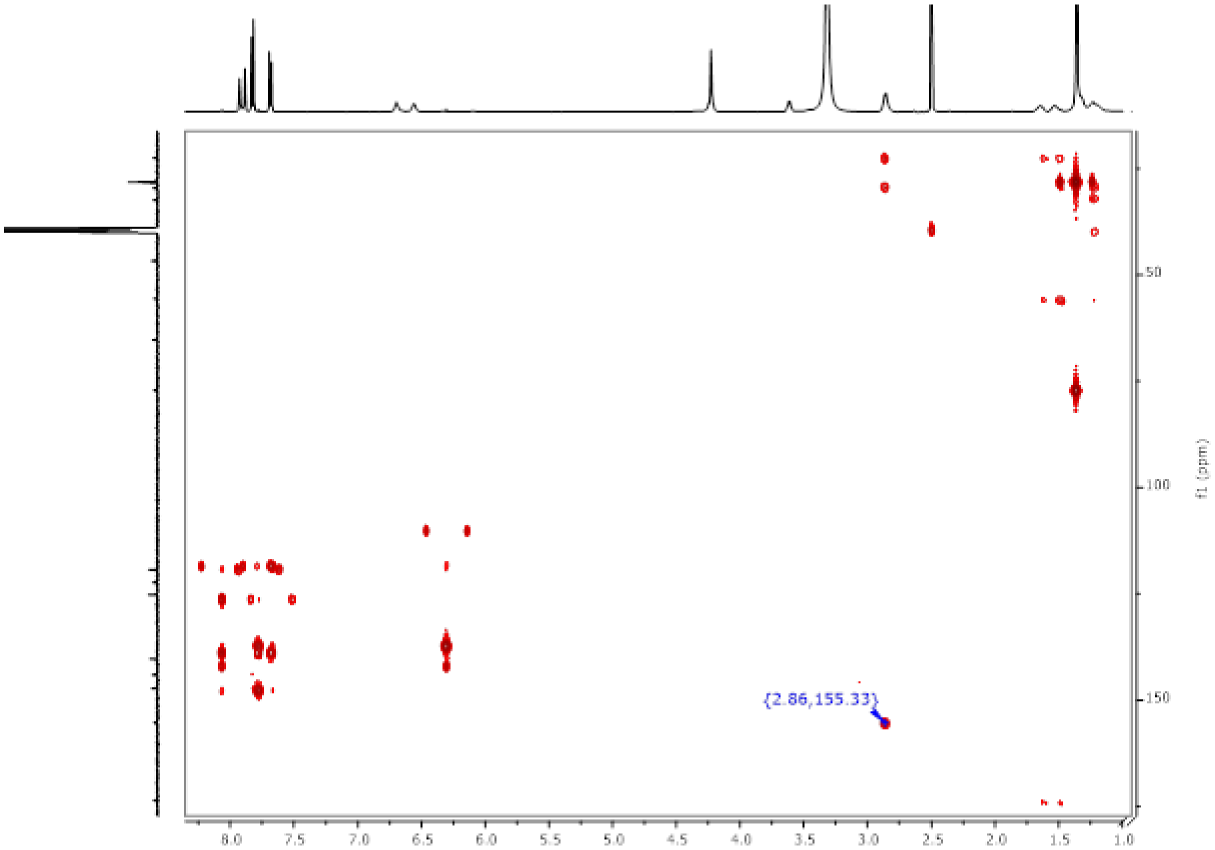

Figure S231: ^{1}H-^{13}C HMBC-NMR of Smoc-L-Lys(Boc)-OH **18**.

3.2.17. Analytical data of Smoc-L-Met-OH 19

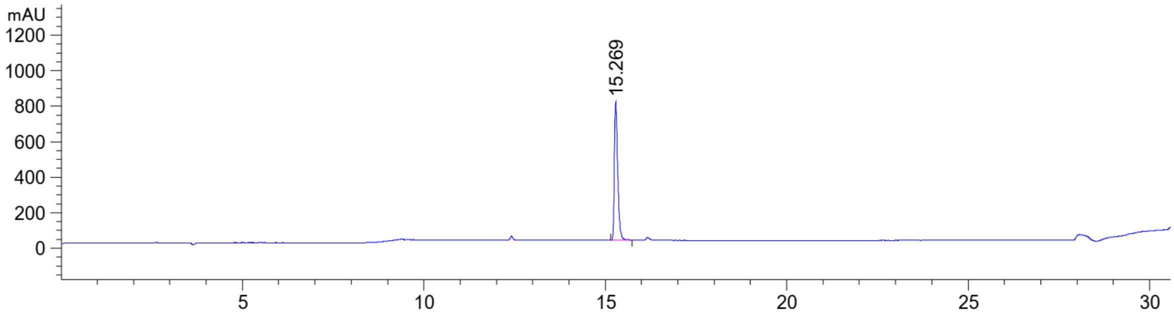

Figure S232: HPLC chromatogram of Smoc-L-Met-OH **19** at λ=220 nm (0to40 MeCN).

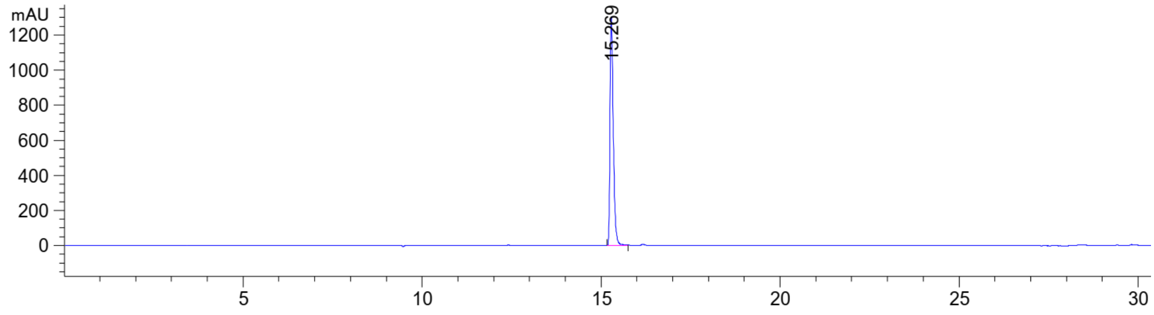

Figure S233: HPLC chromatogram of Smoc-L-Met-OH **19** at λ=280 nm (0to40 MeCN).

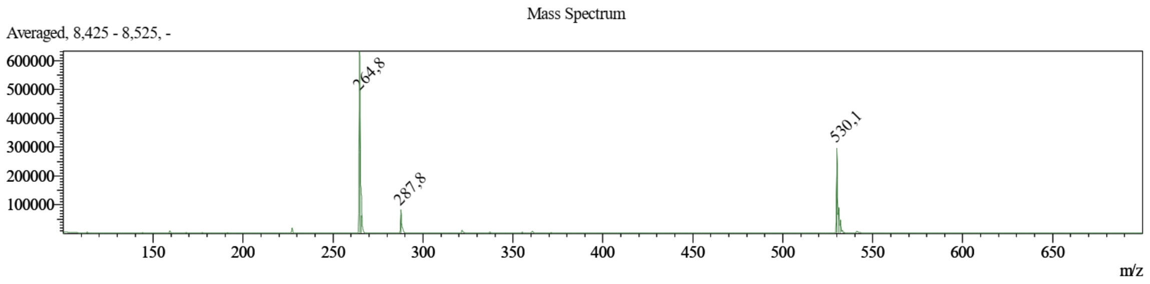

Figure S234: ESI-MS of Smoc-L-Met-OH **19** (M measured=530.10 [M-H]$^-$, M calc.=531.57).

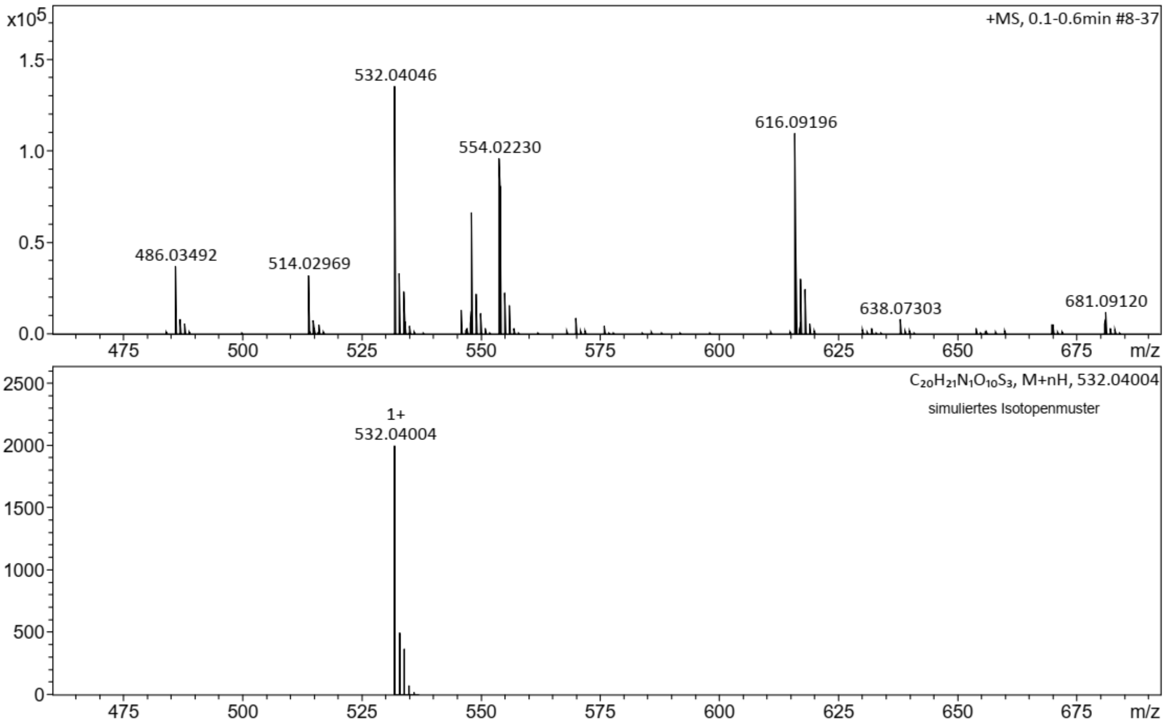

Figure S235: HR-MS of Smoc-L-Met-OH **19** (M measured=532.04046 [M+H]$^+$, M calc.=532.04004).

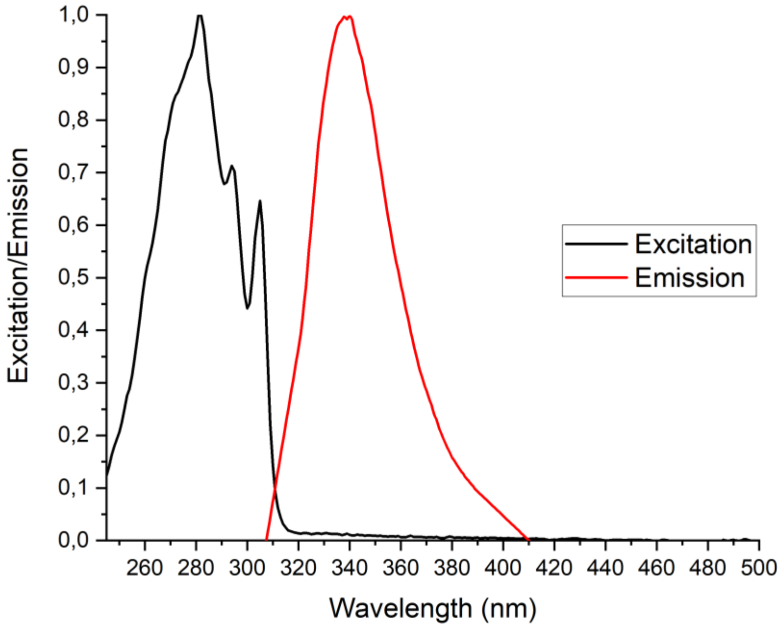

Figure S236: Excitation and emission spectra of Smoc-L-Met-OH **19**, excitation and emission have been normalized between 0 and 1 for illustration.

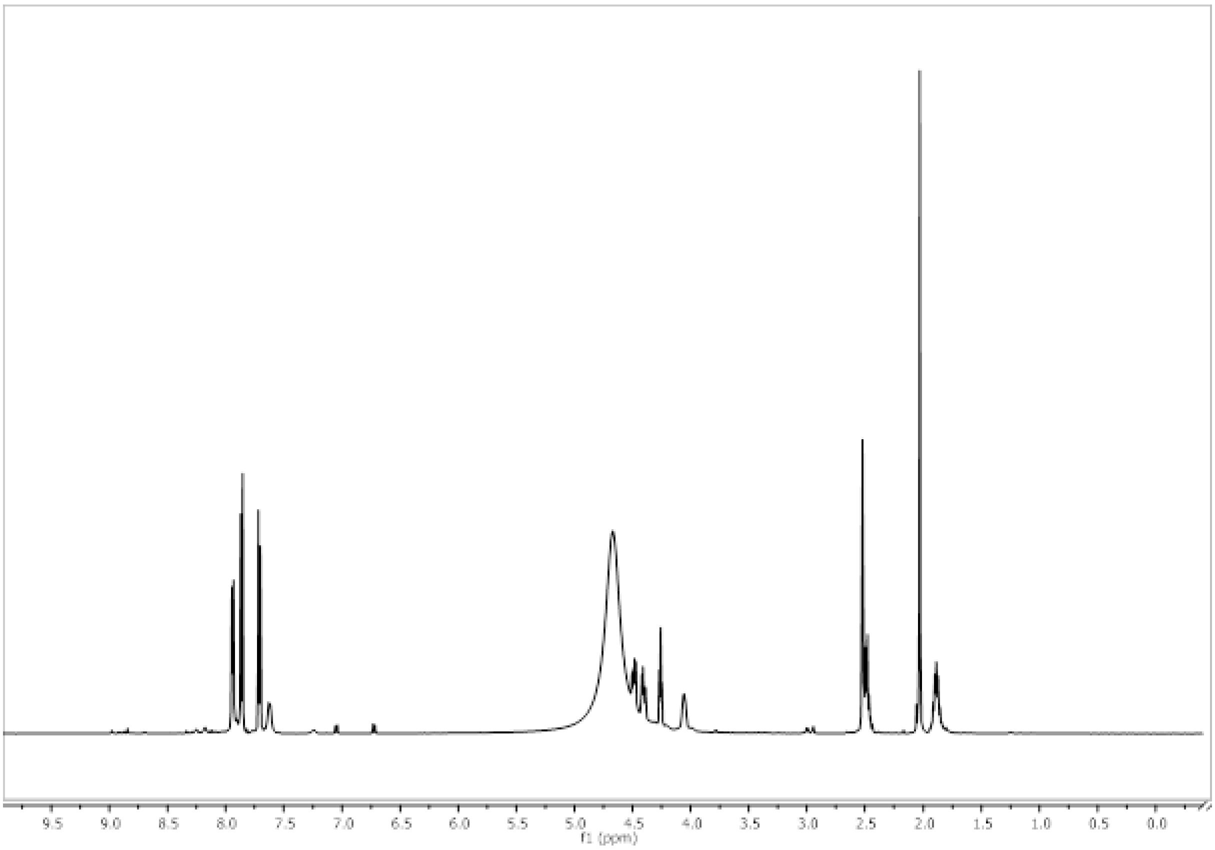

Figure S237: ^{1}H-NMR of Smoc-L-Met-OH **19**.

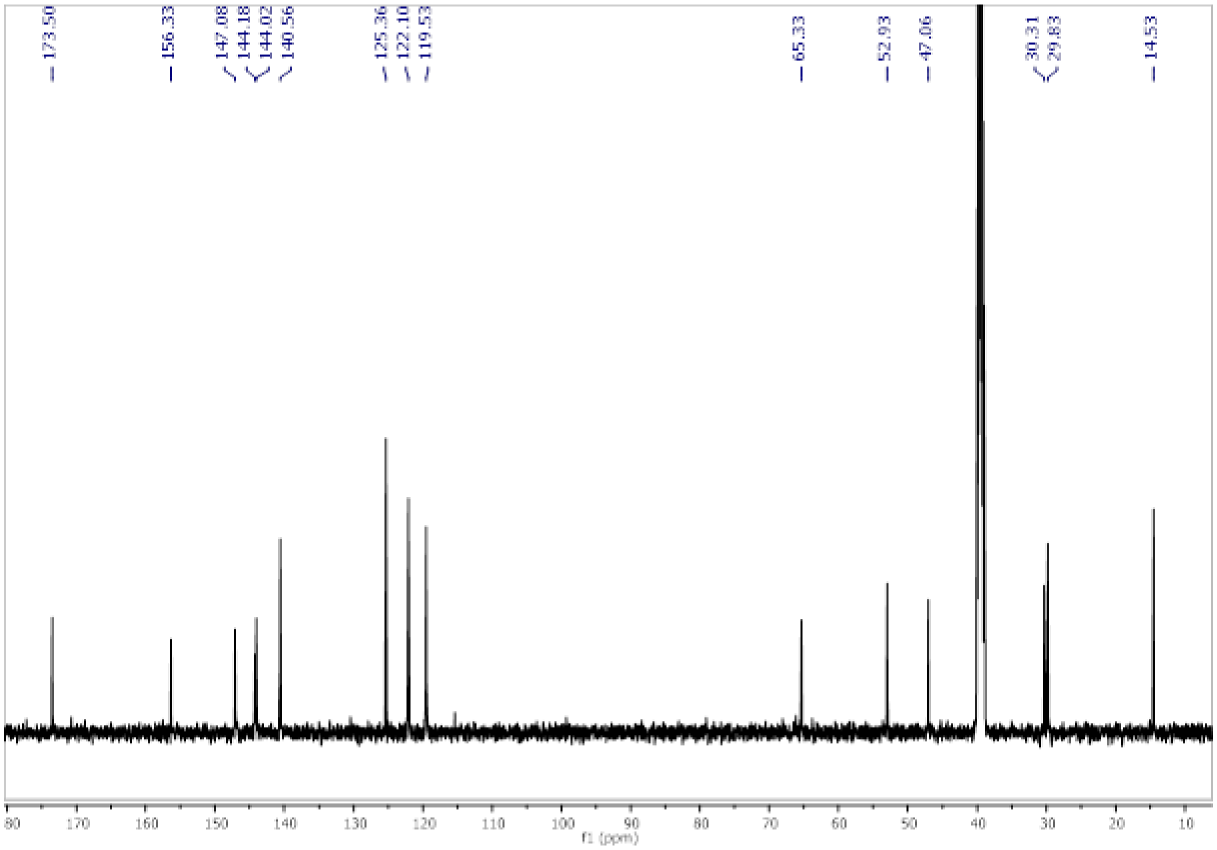

Figure S238: ^{13}C-NMR of Smoc-L-Met-OH **19**.

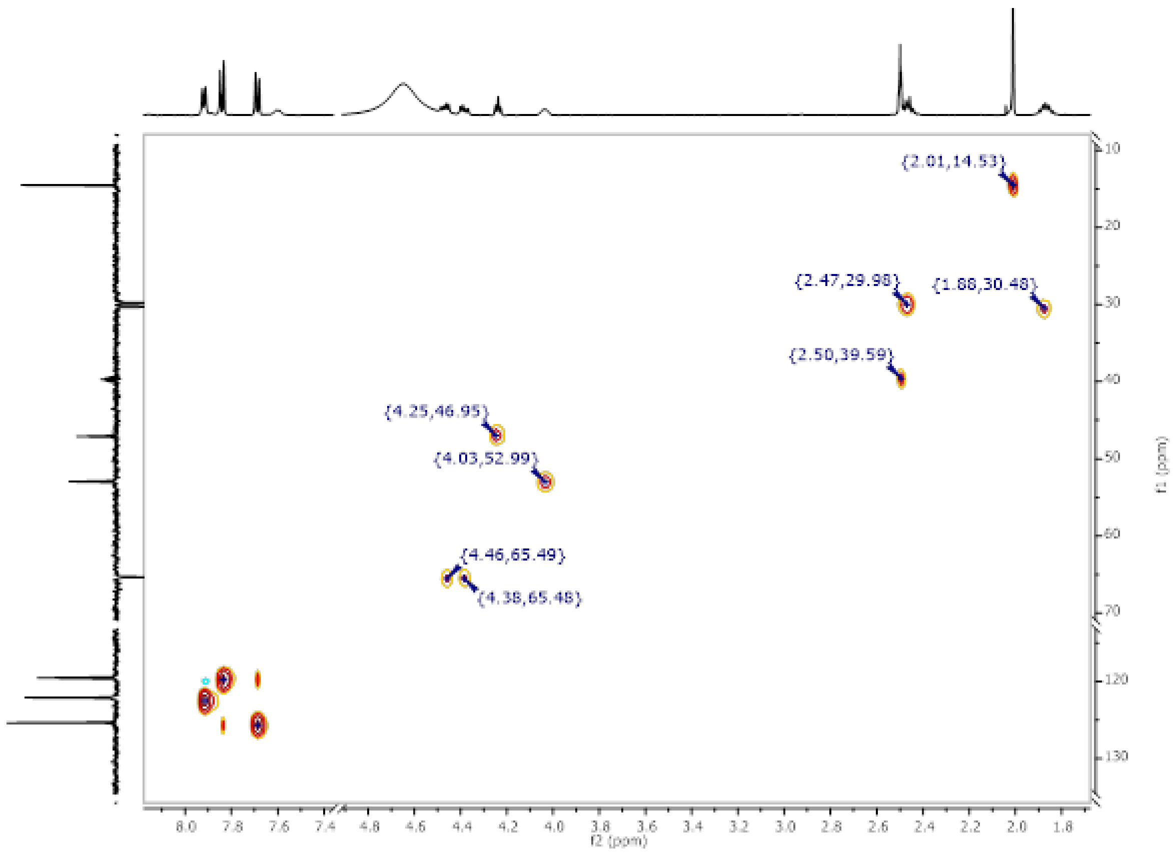

Figure S239: ^{1}H-^{13}C HSQC-NMR of Smoc-L-Met-OH **19**.

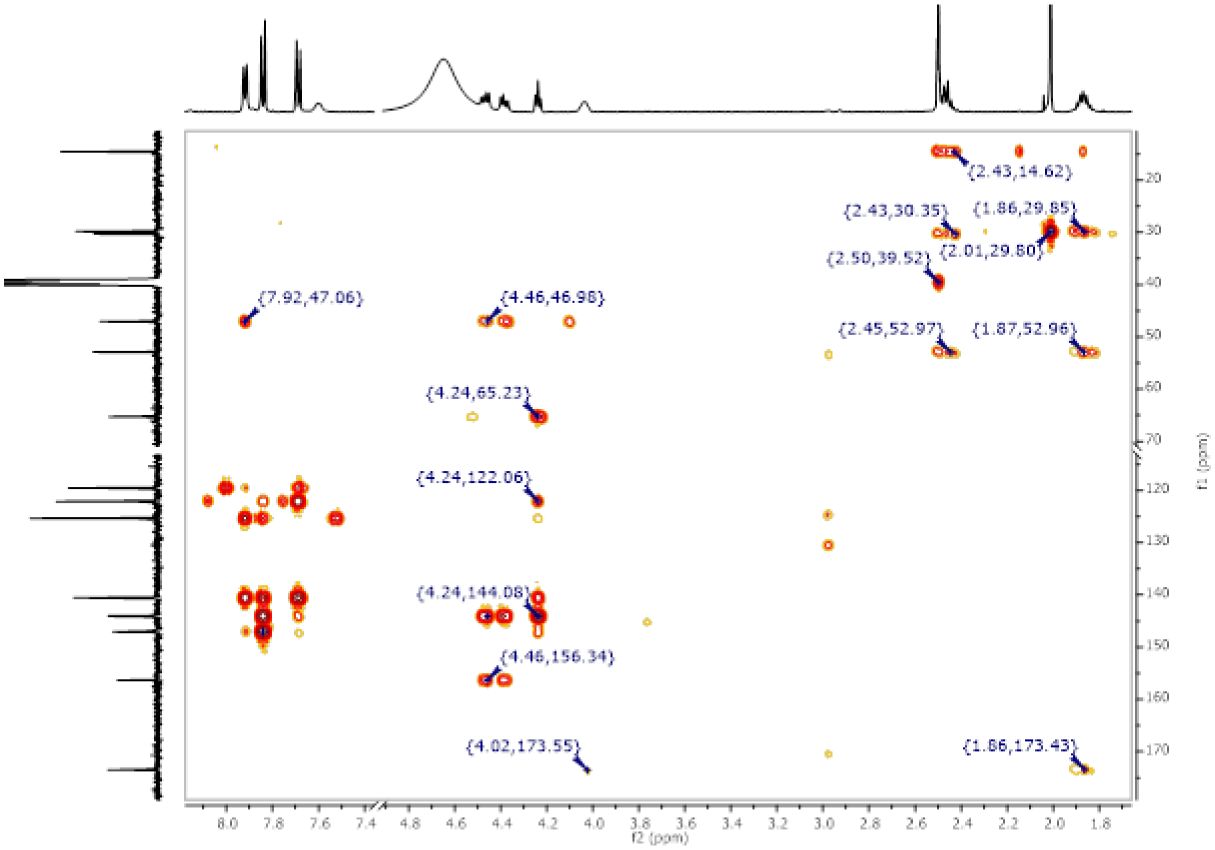

Figure S240: ^{1}H-^{13}C HMBC-NMR of Smoc-L-Met-OH **19**.

3.2.18. Analytical data of Smoc-L-Phe-OH 20

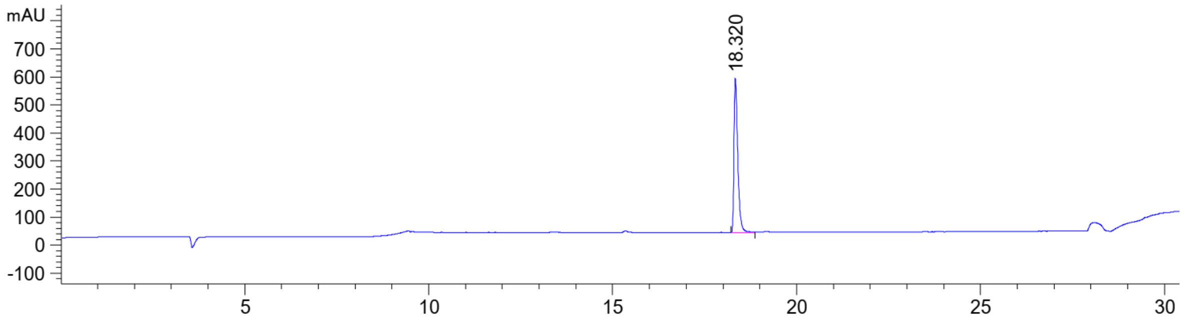

Figure S241: HPLC chromatogram of Smoc-L-Phe-OH **20** at λ=220 nm (0to40 MeCN).

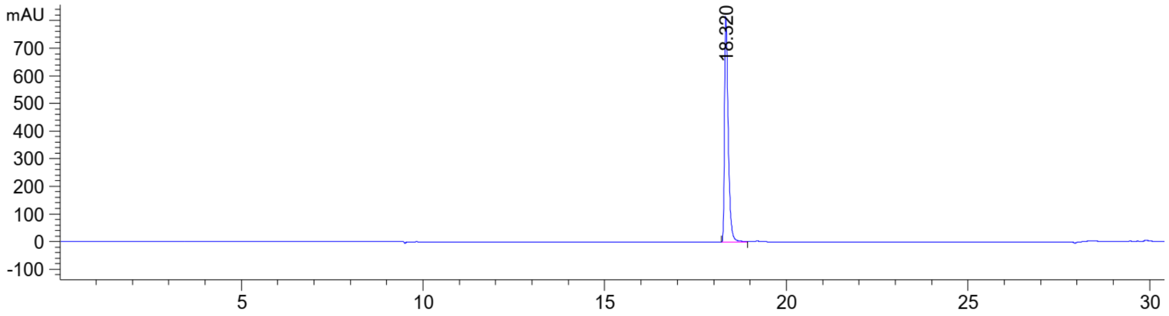

Figure S242: HPLC chromatogram of Smoc-L-Phe-OH **20** at λ=280 nm (0to40 MeCN).

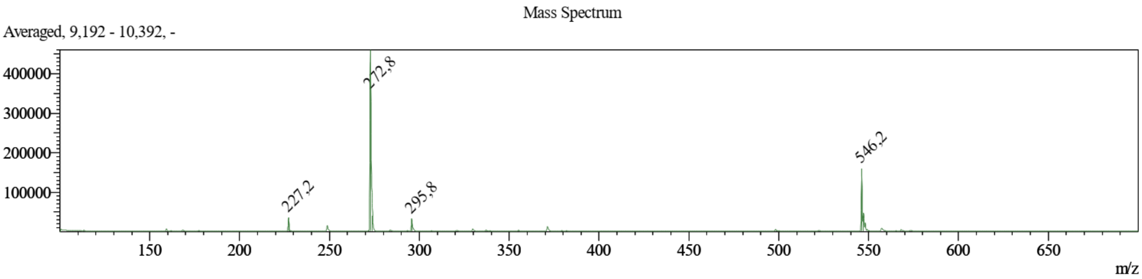

Figure S243: ESI-MS of Smoc-L-Phe-OH **20** (M measured=546.20 [M-H]$^-$, M calc.=547.55).

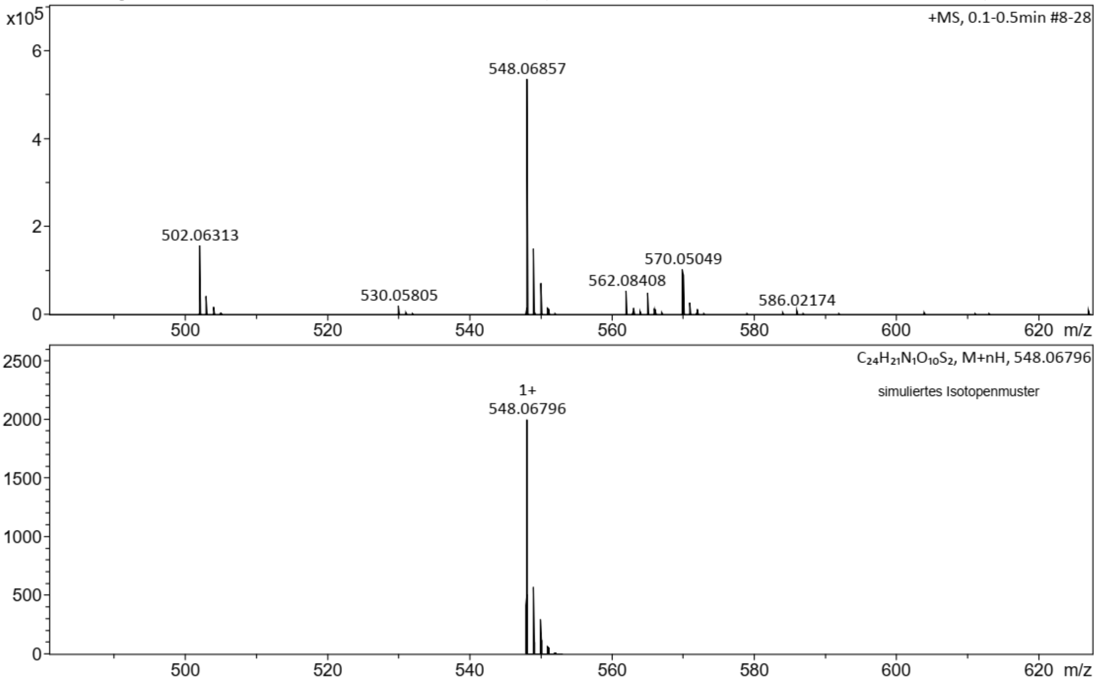

Figure S244: HR-MS of Smoc-L-Phe-OH **20** (M measured=548.06857 [M+H]$^+$, M calc.=548.006796).

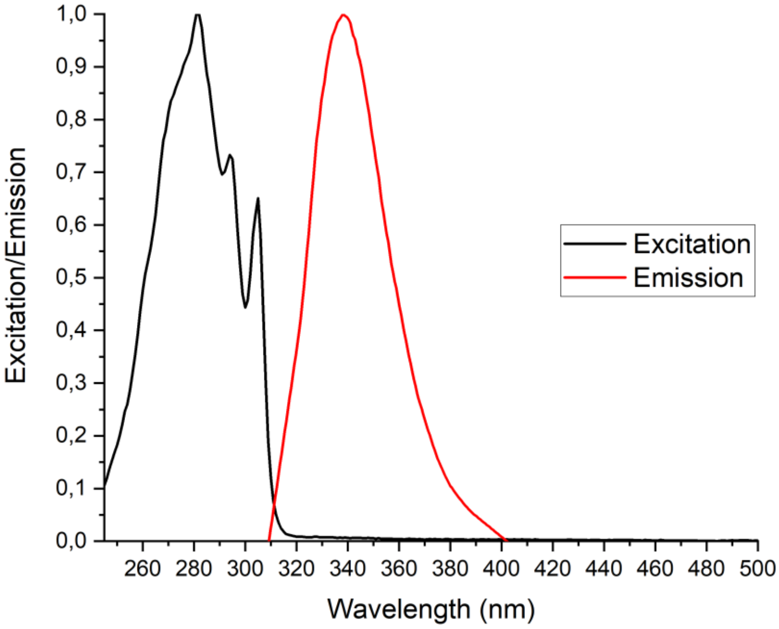

Figure S245: Excitation and emission spectra of Smoc-L-Phe-OH **20**, excitation and emission have been normalized between 0 and 1 for illustration.

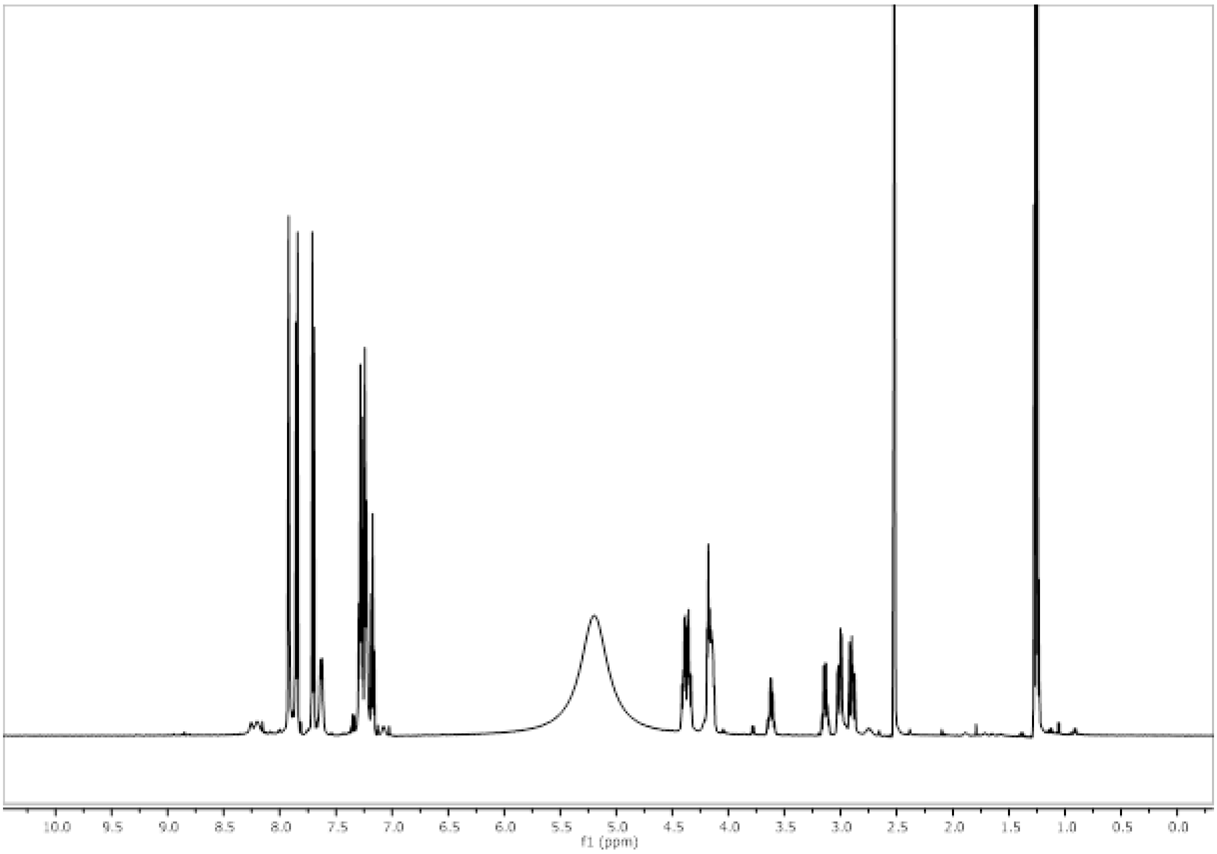

Figure S246: ^{1}H-NMR of Smoc-L-Phe-OH **20**.

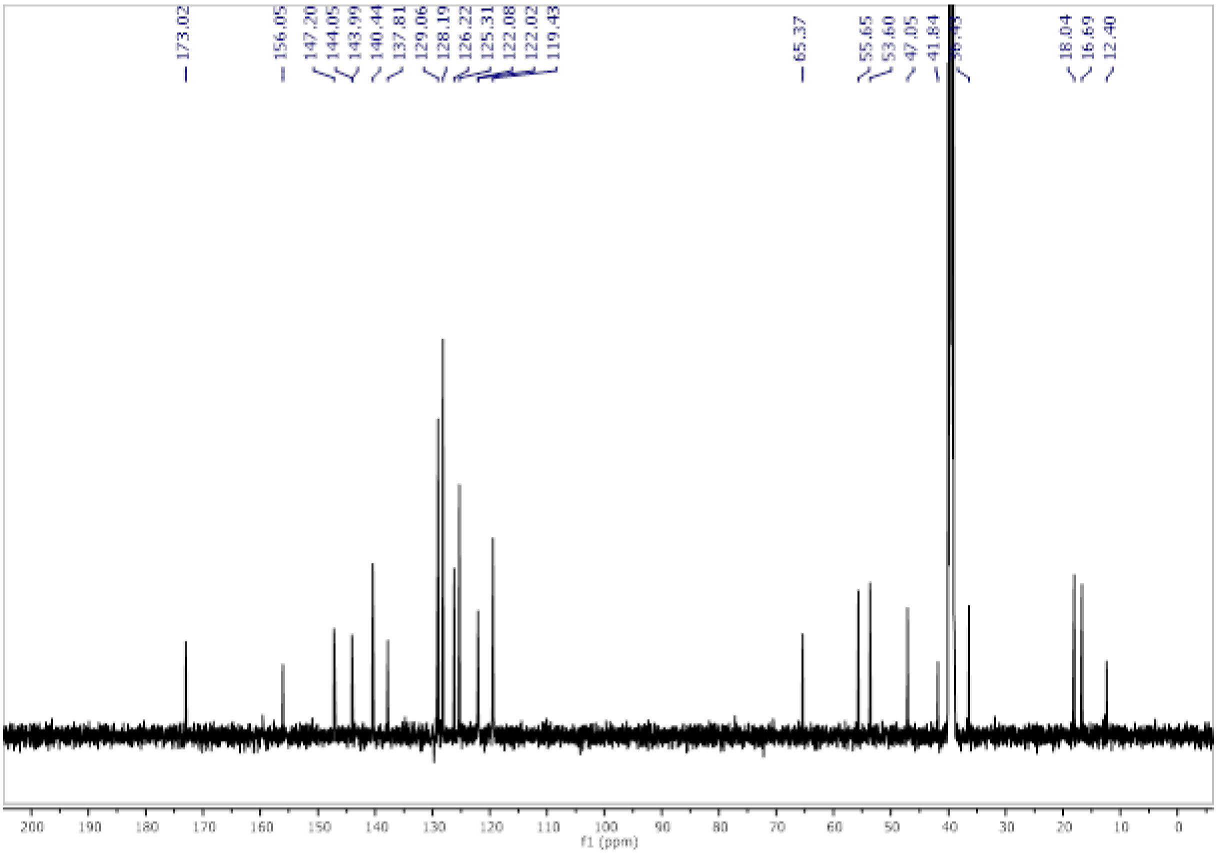

Figure S247: ^{13}C-NMR of Smoc-L-Phe-OH **20**.

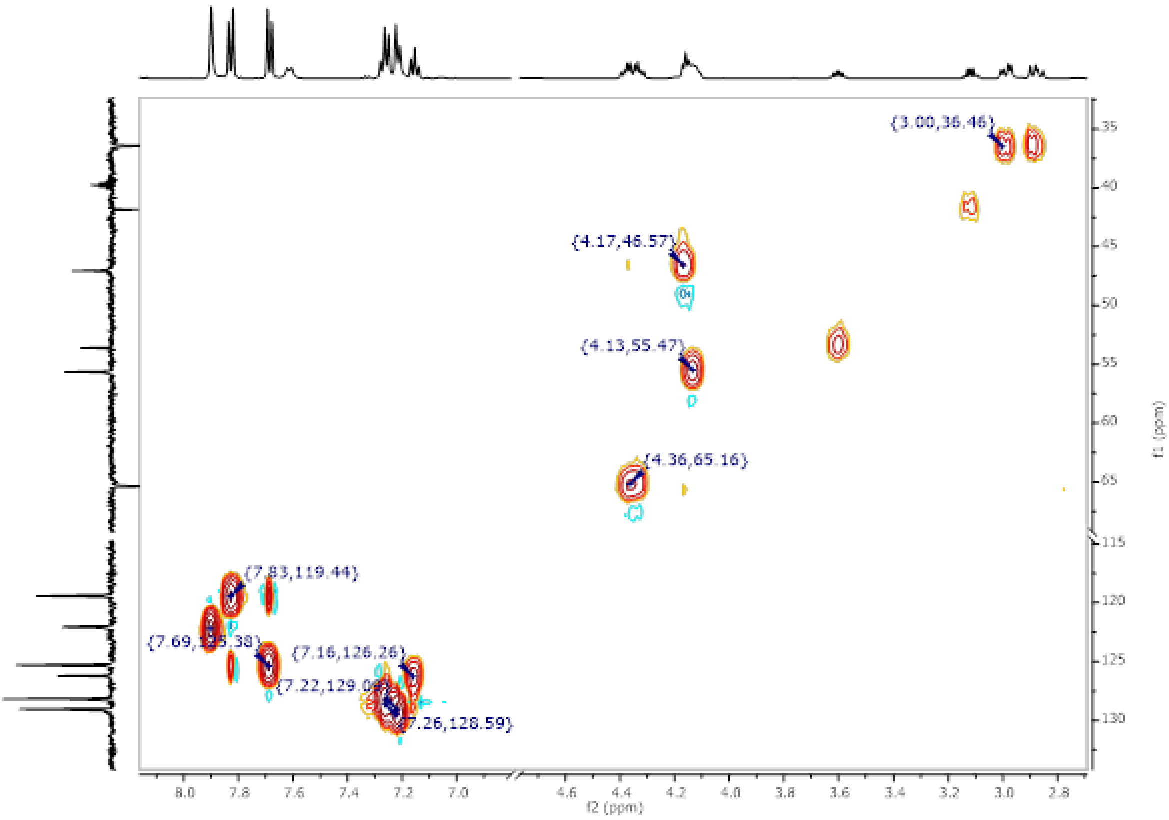

Figure S248: ^{1}H-^{13}C HSQC-NMR of Smoc-L-Phe-OH **20**.

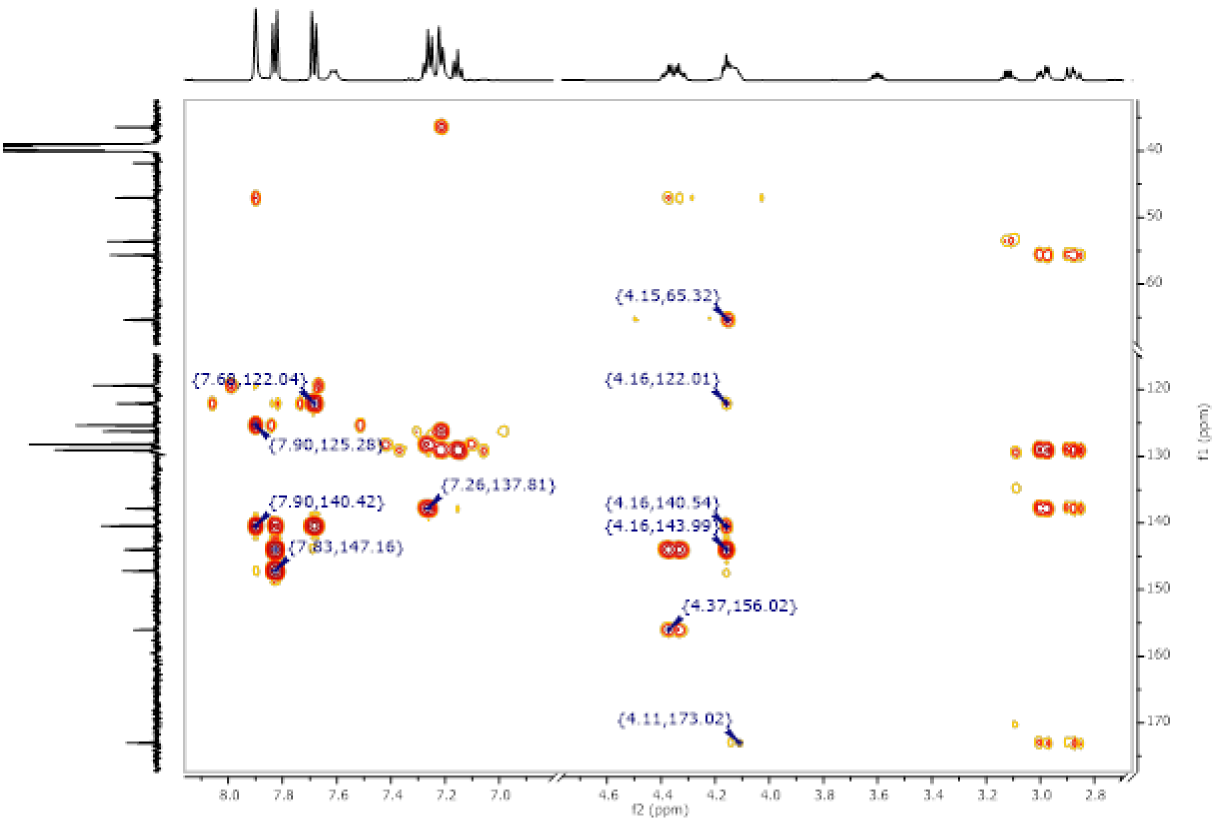

Figure S249: ^{1}H-^{13}C HMBC-NMR of Smoc-L-Phe-OH **20**.

3.2.19. Analytical data of Smoc-L-Pro-OH 21

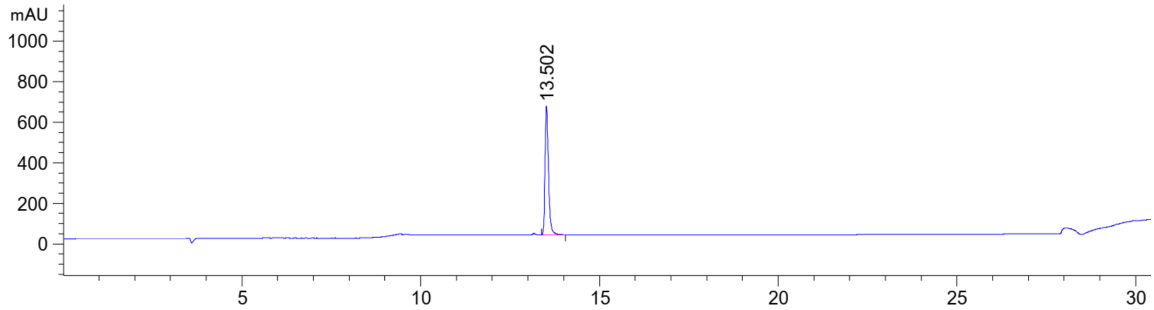

Figure S250: HPLC chromatogram of Smoc-L-Pro-OH **21** at λ=220 nm (0to40 MeCN).

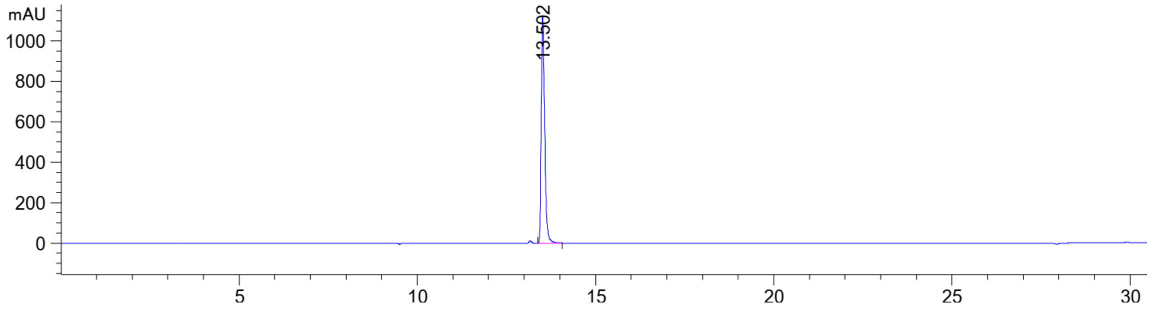

Figure S251: HPLC chromatogram of Smoc-L-Pro-OH **21** at λ=280 nm (0to40 MeCN).

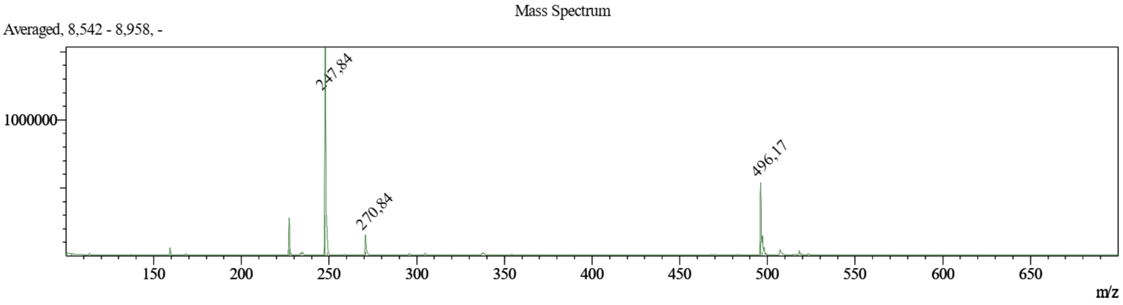

Figure S252: ESI-MS of Smoc-L-Pro-OH **21** (M measured=496.17 $[M-H]^-$, M calc.=497.49).

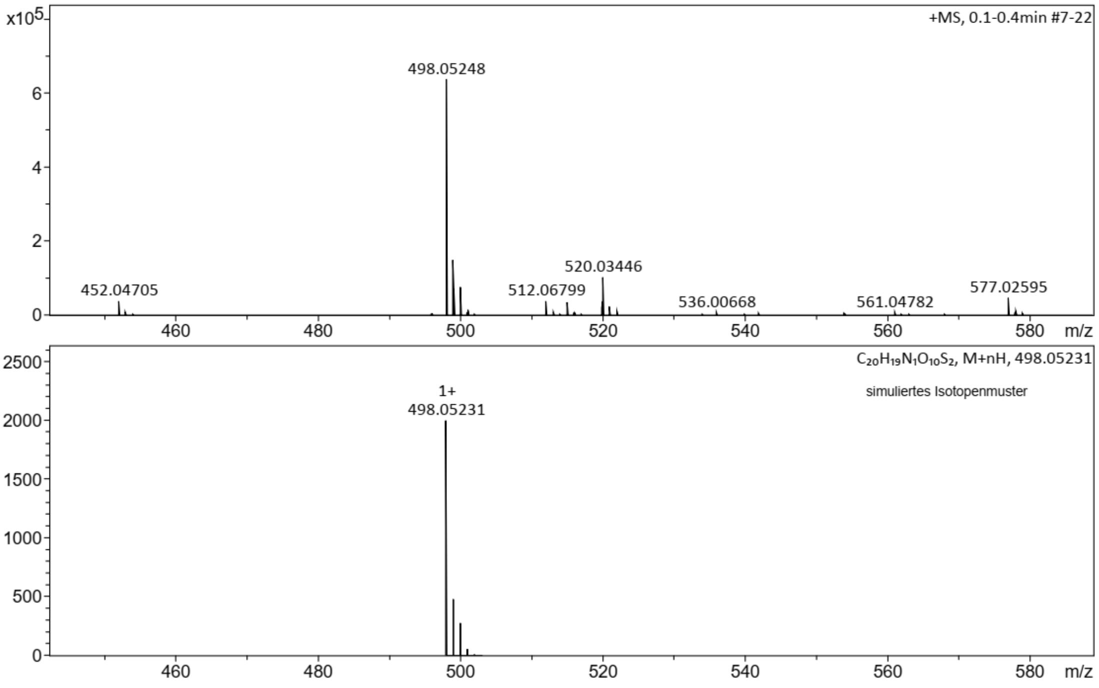

Figure S253: HR-MS of Smoc-L-Pro-OH **21** (M measured=498.05248 $[M+H]^+$, M calc.=498.05231).

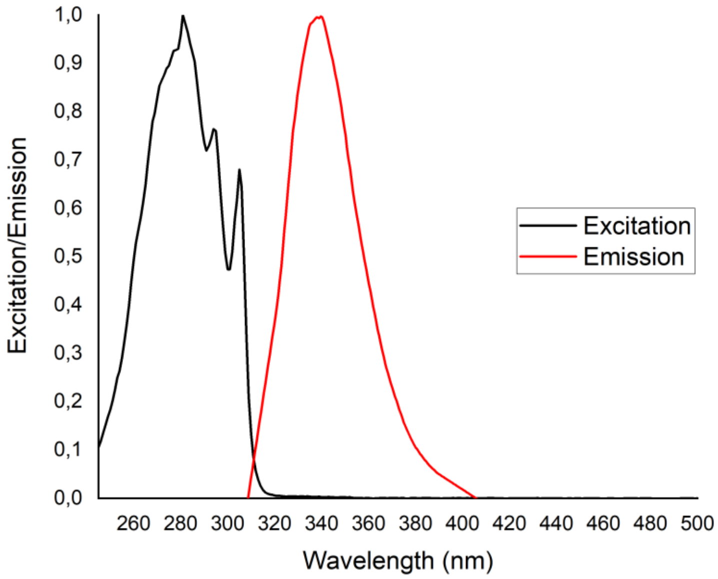

Figure S254: Excitation and emission spectra of Smoc-L-Pro-OH **21**, excitation and emission have been normalized between 0 and 1 for illustration.

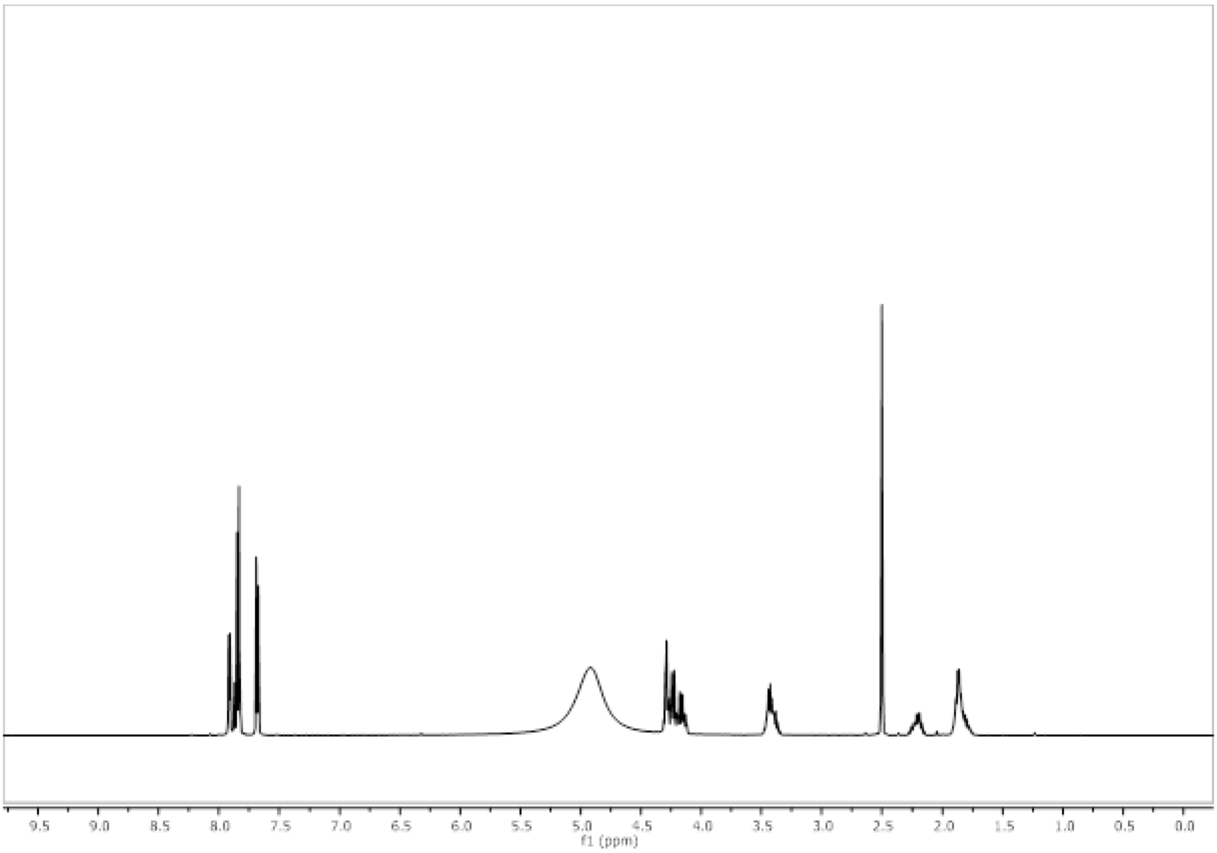

Figure S255: ^{1}H-NMR of Smoc-L-Pro-OH **21**.

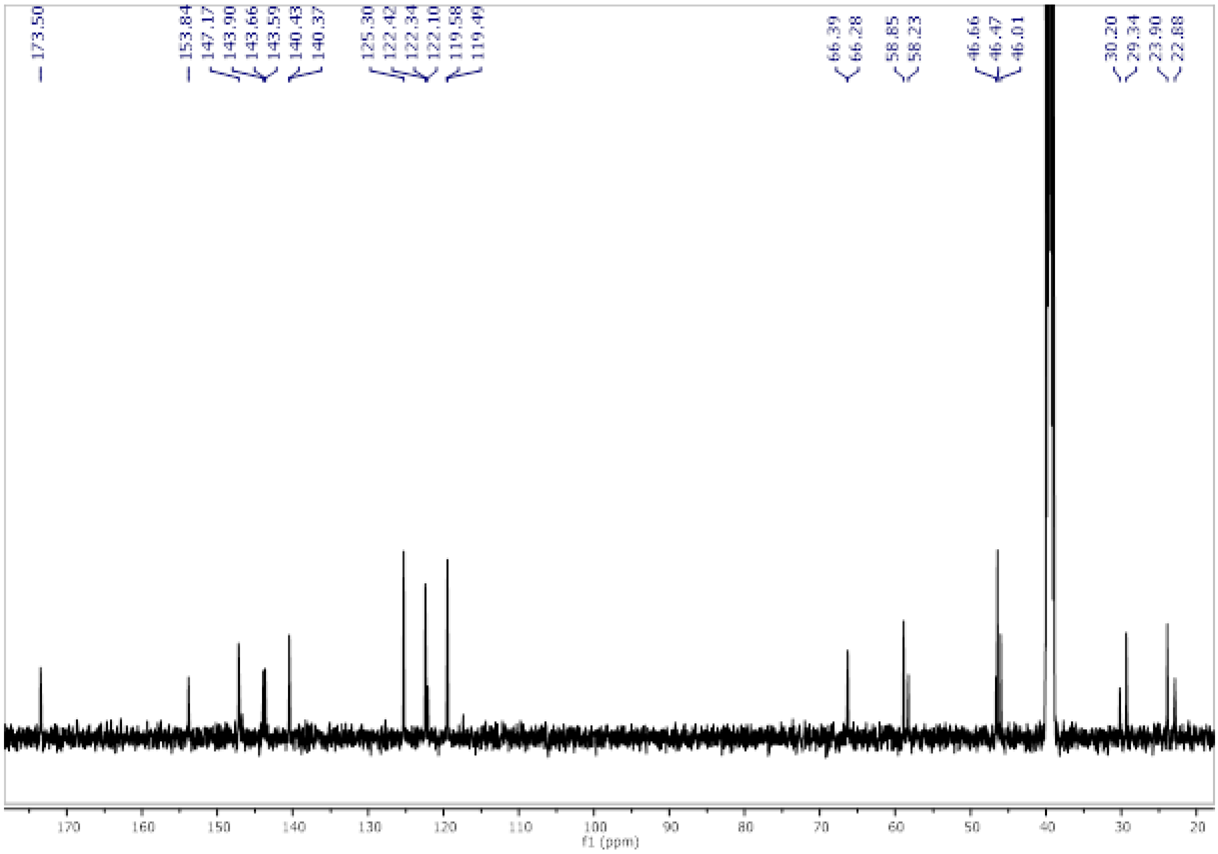

Figure S256: ^{13}C-NMR of Smoc-L-Pro-OH **21**.

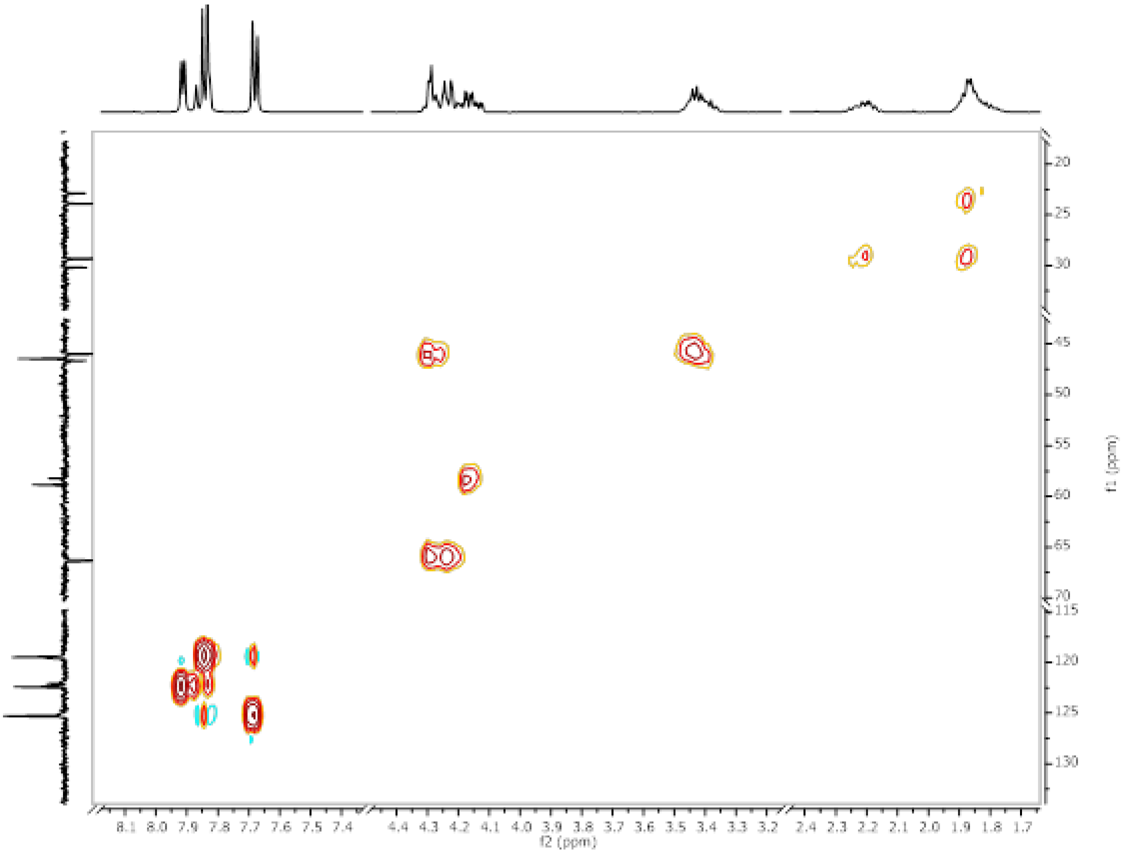

Figure S257: ^{1}H-^{13}C HSQC-NMR of Smoc-L-Pro-OH **21**.

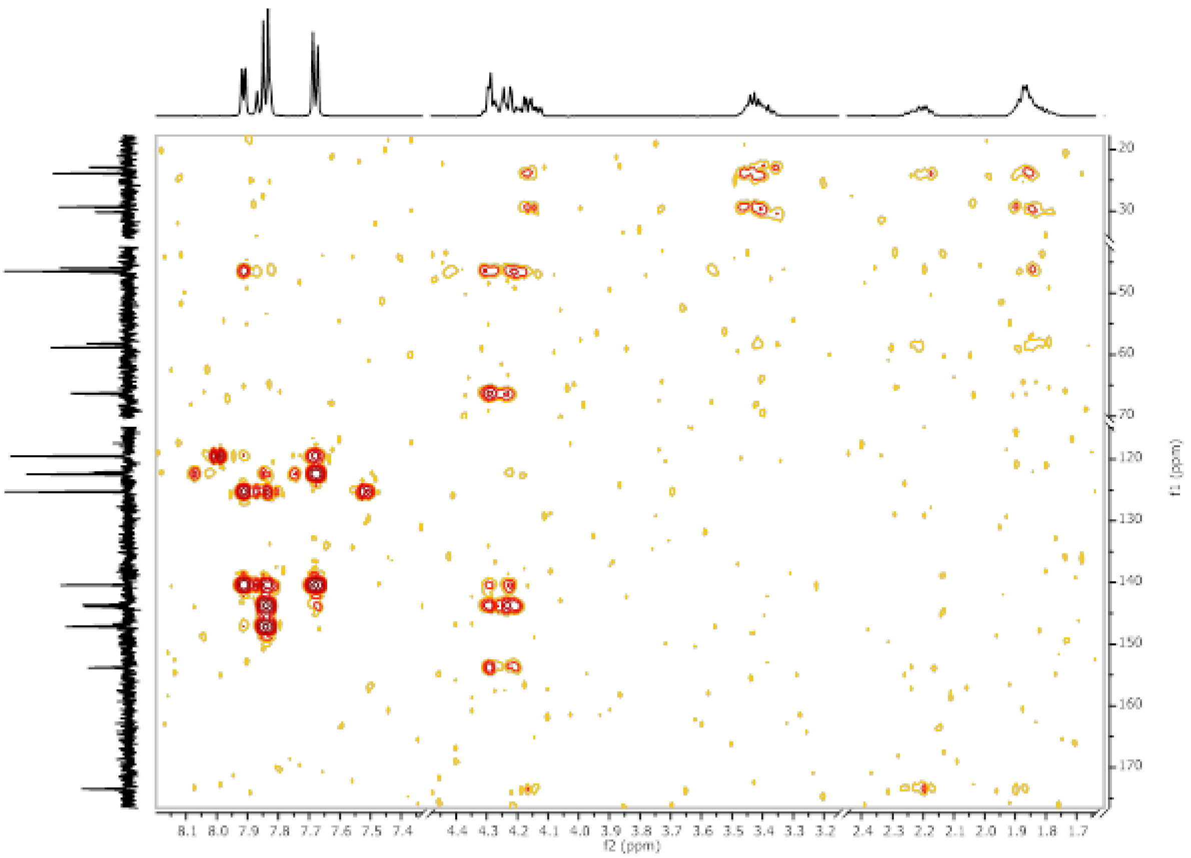

Figure S258: ^{1}H-^{13}C HMBC-NMR of Smoc-L-Pro-OH **21**.

3.2.20. Analytical data of Smoc-L-Ser-OH 22

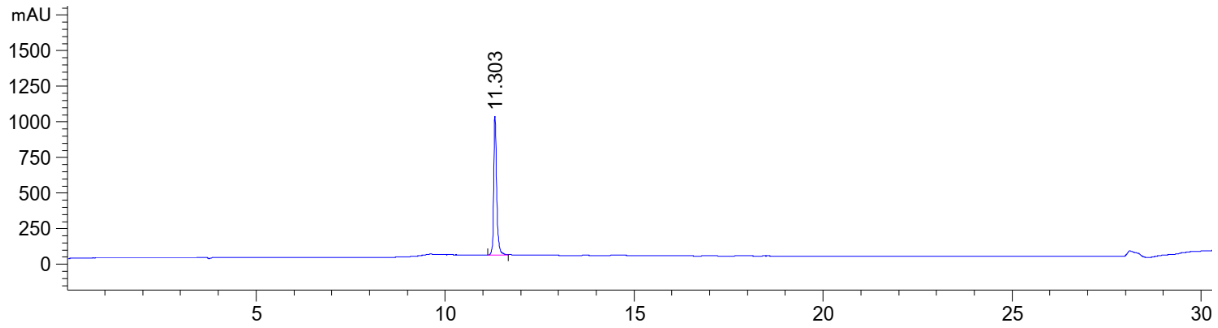

Figure S259: HPLC chromatogram of Smoc-L-Ser-OH **22** at λ=220 nm (0to40 MeCN).

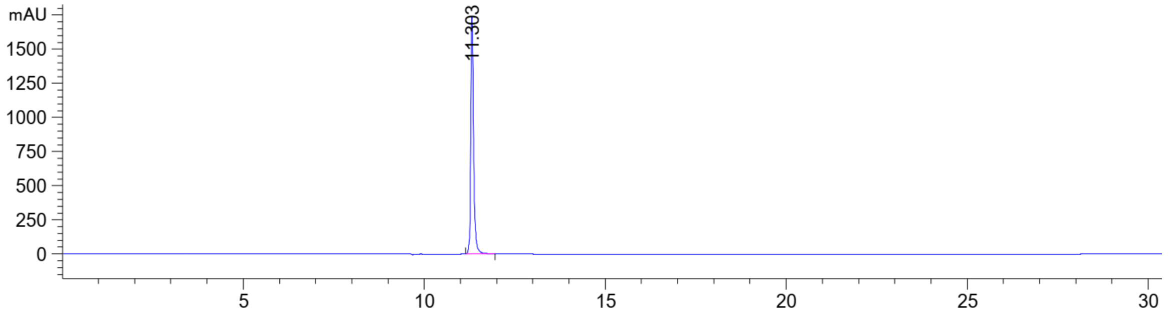

Figure S260: HPLC chromatogram of Smoc-L-Ser-OH **22** at λ=280 nm (0to40 MeCN).

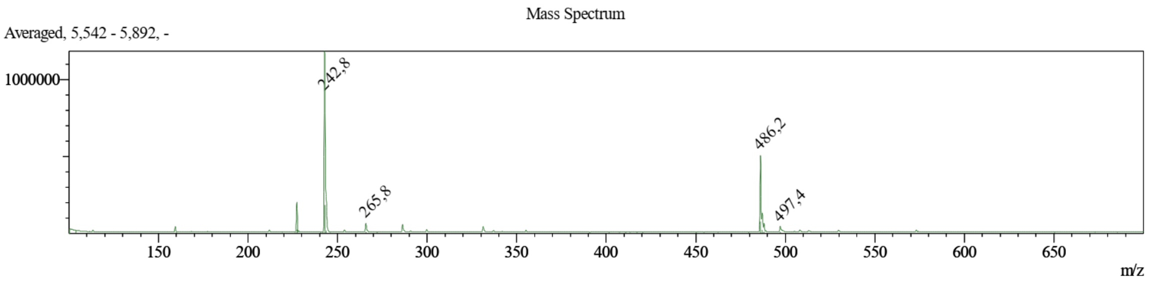

Figure S261: ESI-MS of Smoc-L-Ser-OH **22** (M measured=486.20 [M-H]$^-$, M calc.=487.45).

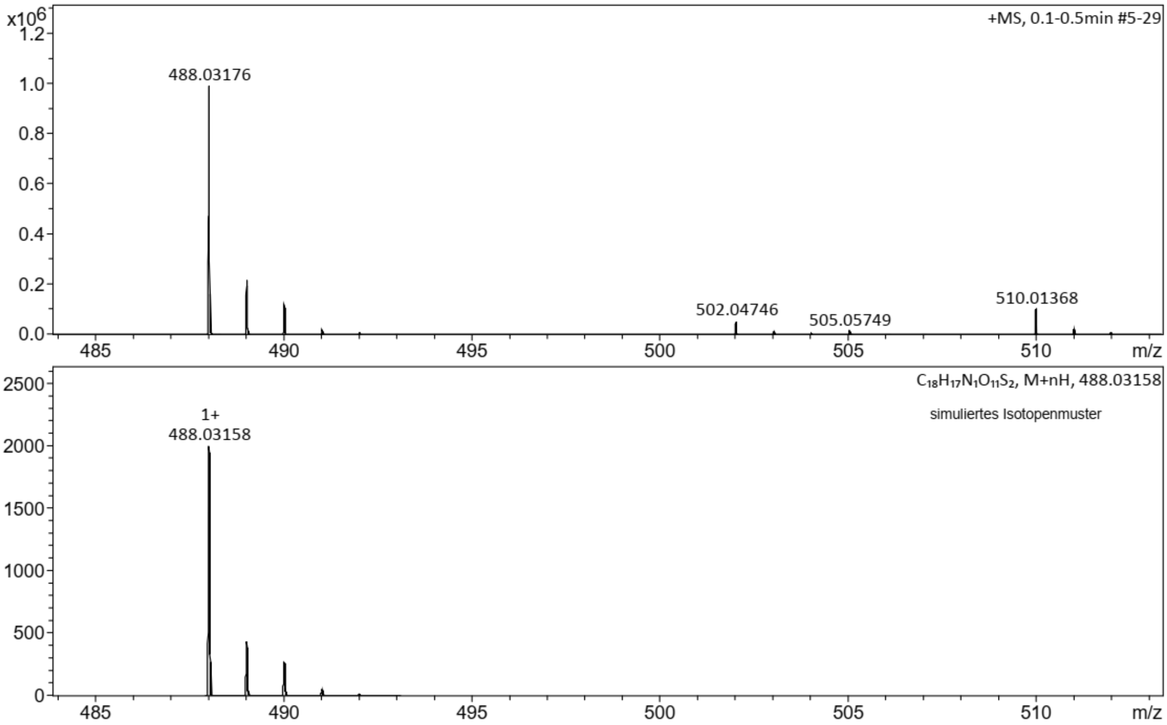

Figure S262: HR-MS of Smoc-L-Ser-OH **22** (M measured=488.03176 [M+H]$^+$, M calc.=488.03158).

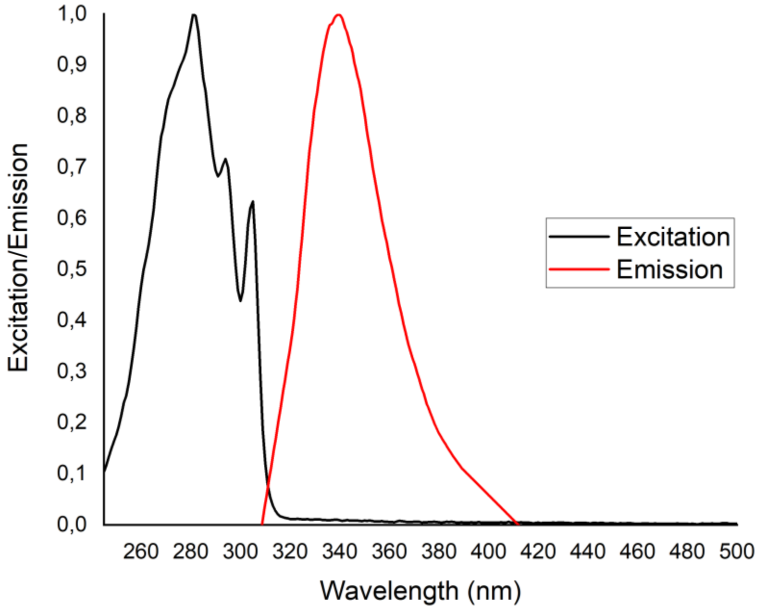

Figure S263: Excitation and emission spectra of Smoc-L-Ser-OH **22**, excitation and emission have been normalized between 0 and 1 for illustration.

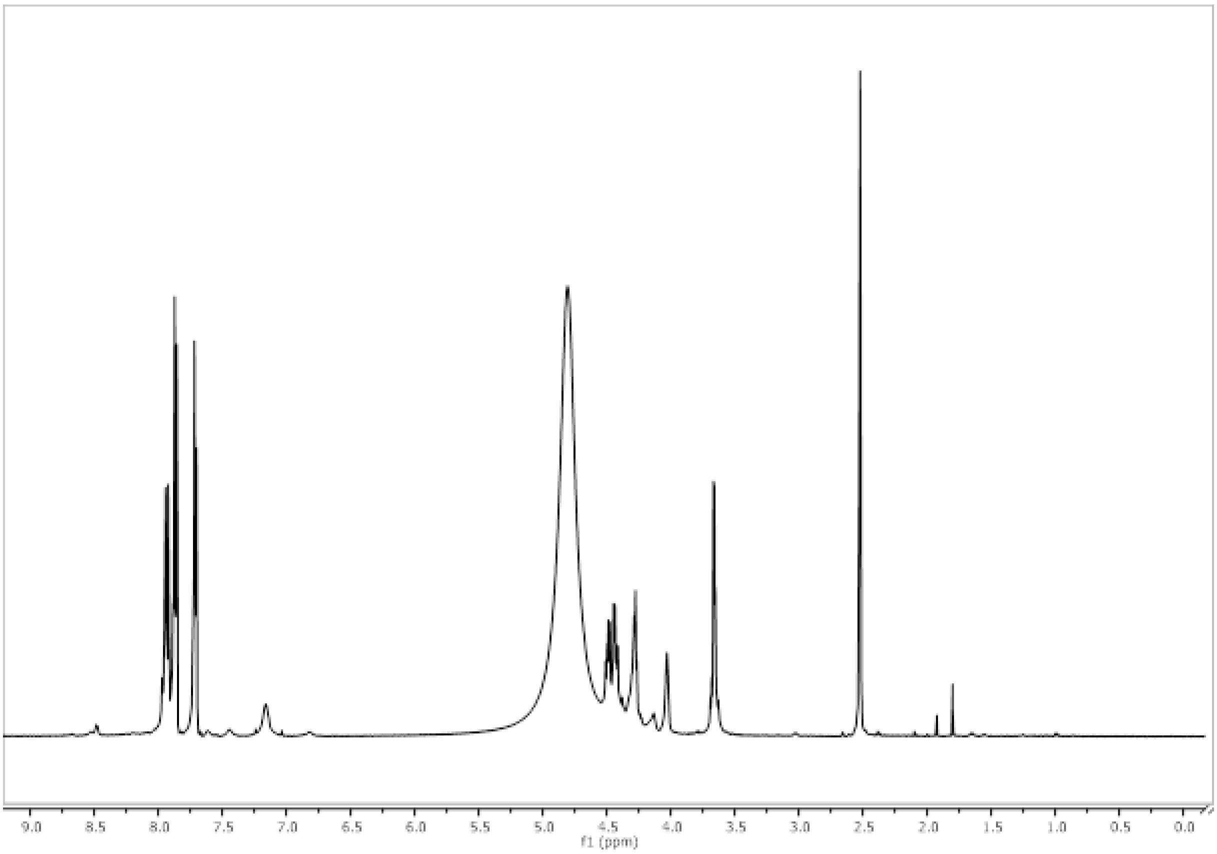

Figure S264: ^{1}H-NMR of Smoc-L-Ser-OH **22**.

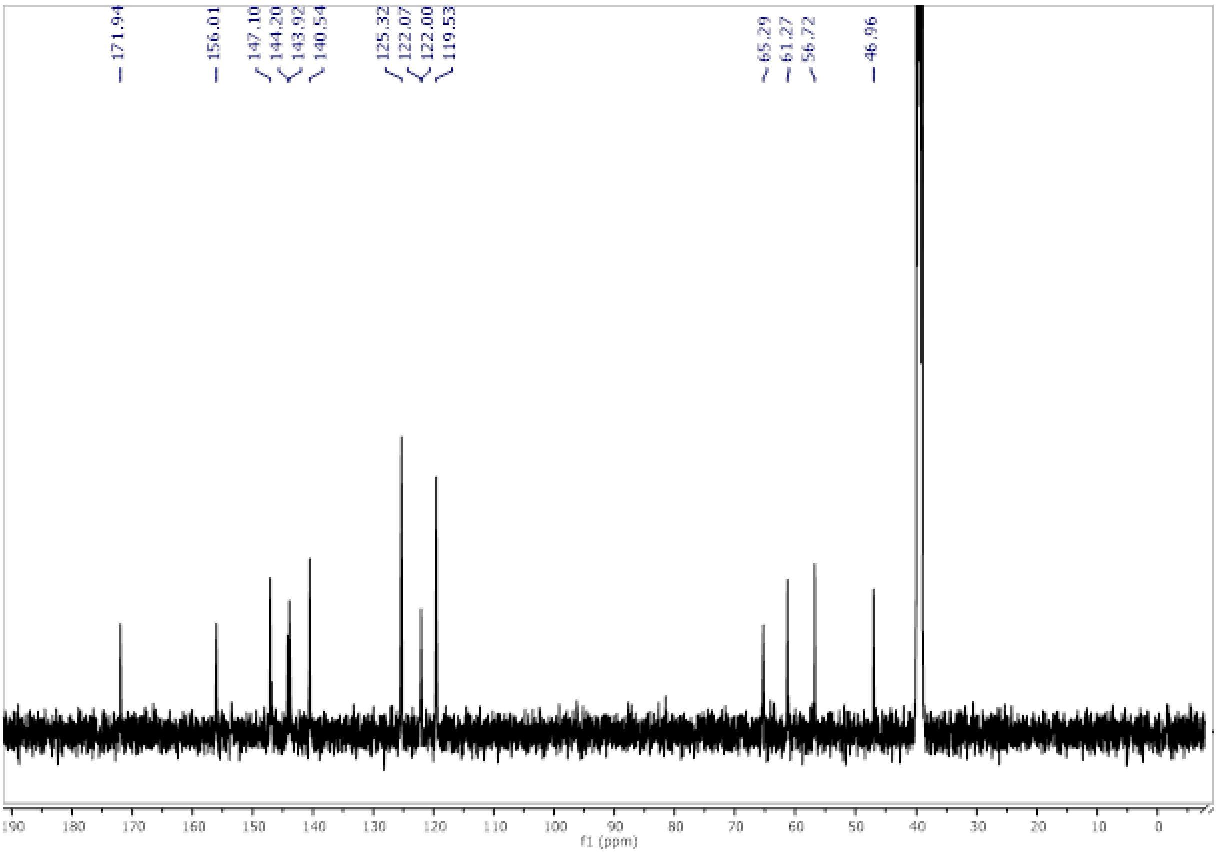

Figure S265: ^{13}C-NMR of Smoc-L-Ser-OH **22**.

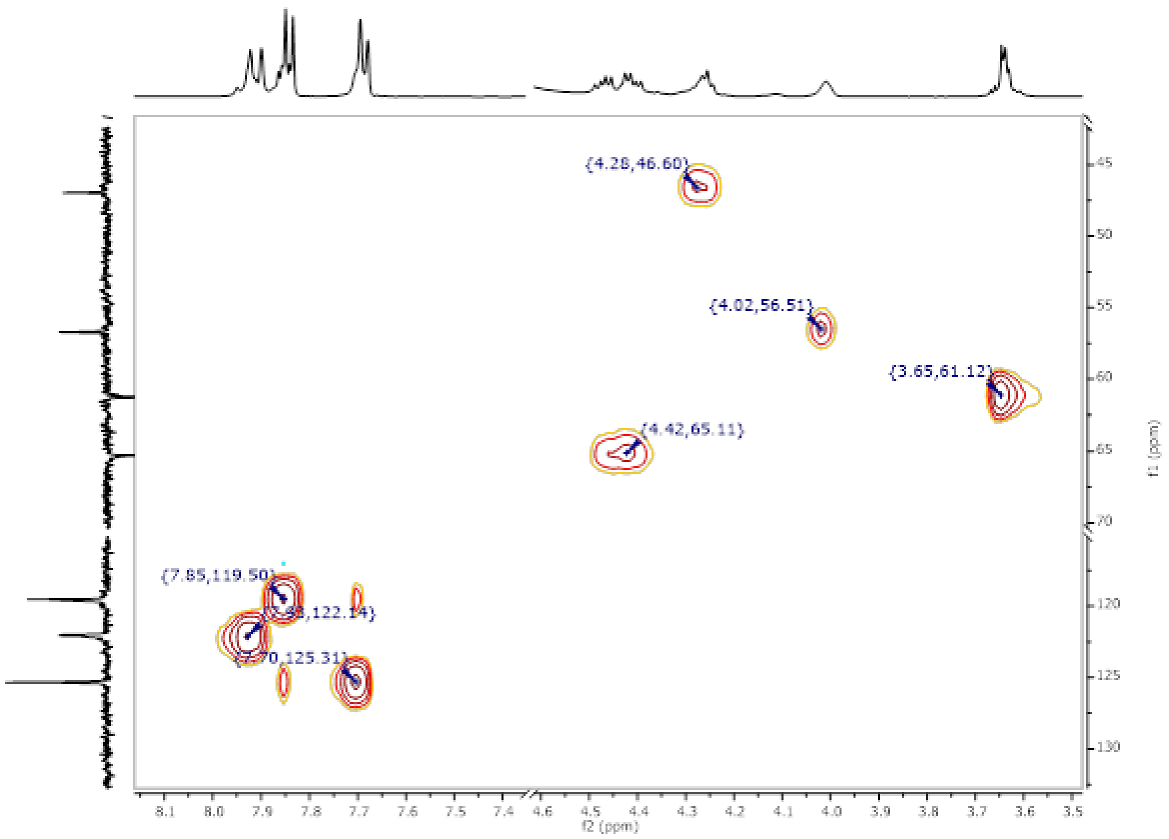

Figure S266: ^{1}H-^{13}C HSQC-NMR of Smoc-L-Ser-OH **22**.

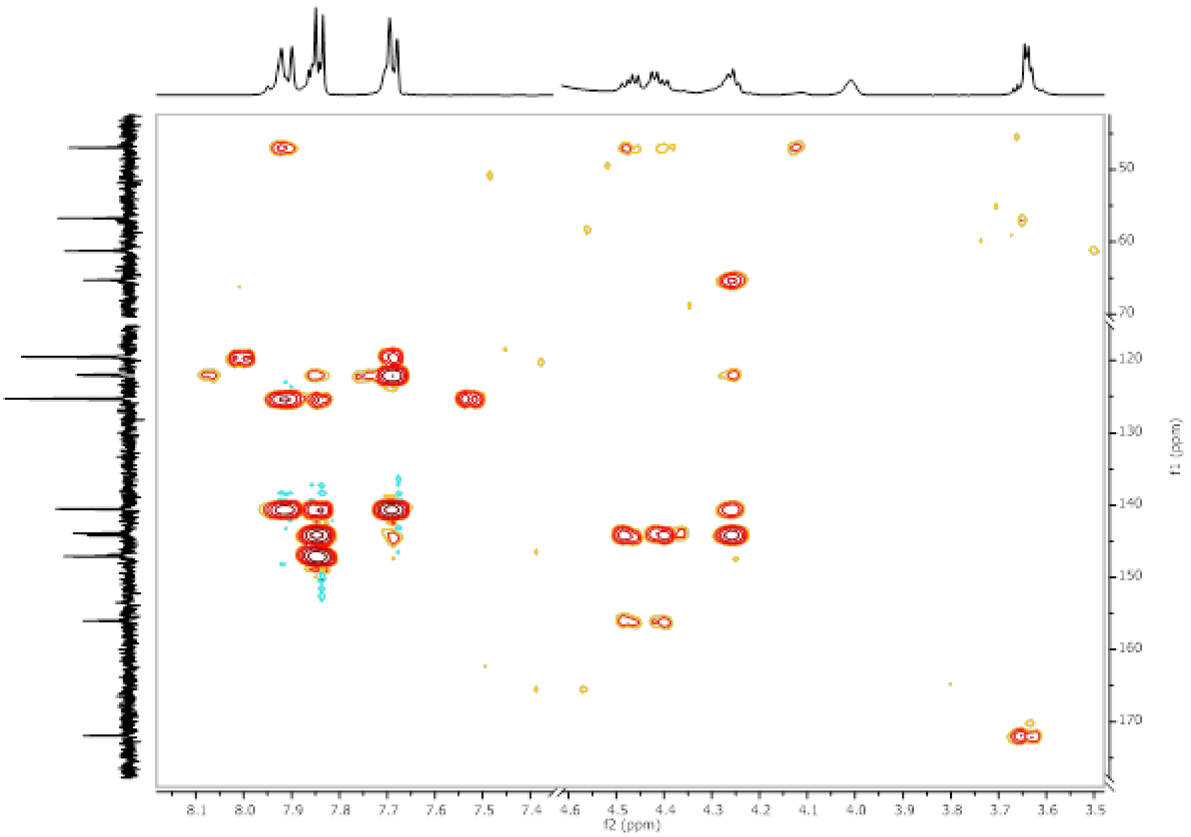

Figure S267: ^{1}H-^{13}C HMBC-NMR of Smoc-L-Ser-OH **22**.

3.2.21. Analytical data of Smoc-L-Ser(tBu)-OH 23

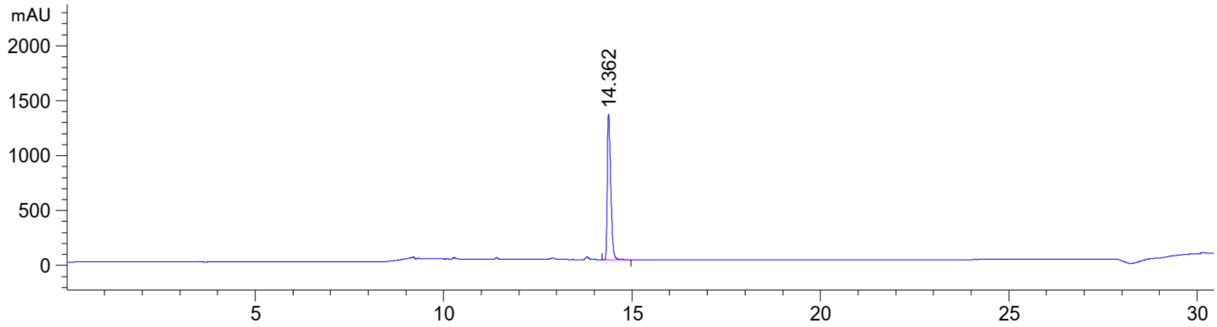

Figure S268: HPLC chromatogram of Smoc-L-Ser(tBu)-OH **23** at λ=220 nm (0to60 MeCN).

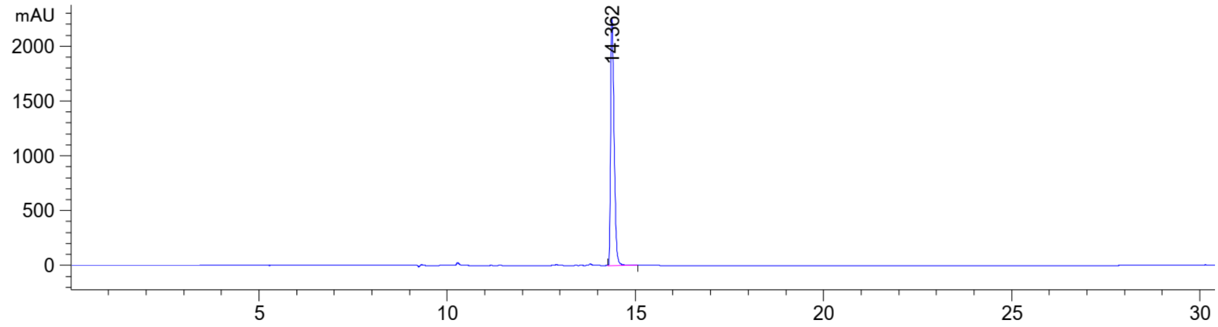

Figure S269: HPLC chromatogram of Smoc-L-Ser(tBu)-OH **23** at λ=280 nm (0to60 MeCN).

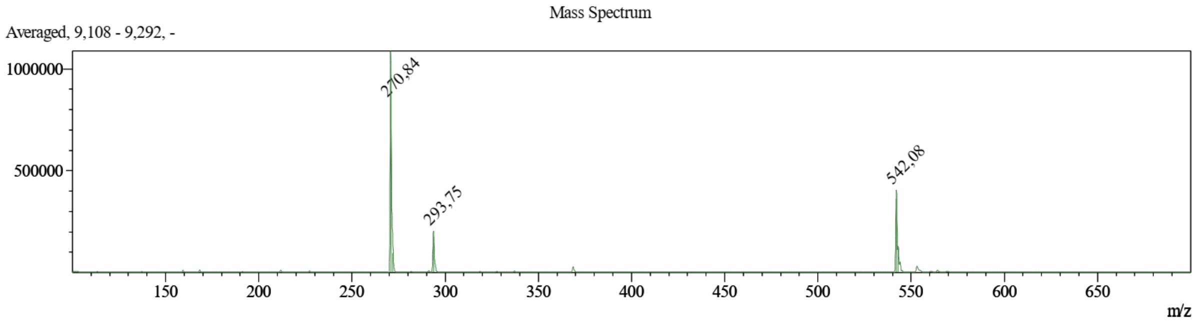

Figure S270: ESI-MS of Smoc-L-Ser(tBu)-OH **23** (M measured=542.08 $[M-H]^-$, M calc.=543.56).

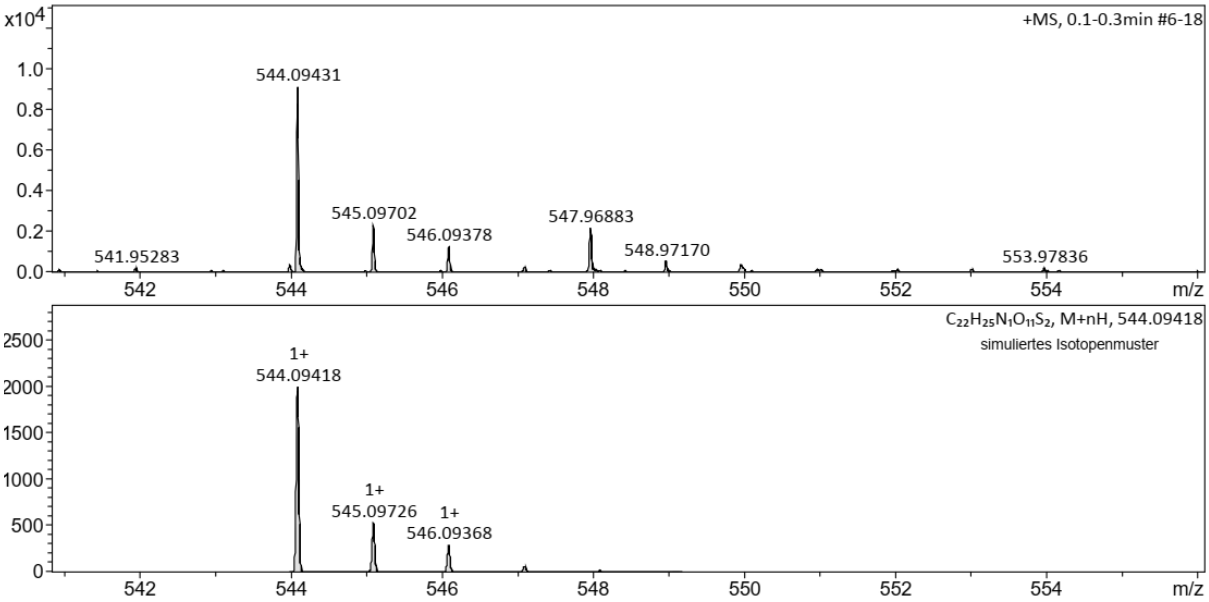

Figure S271: HR-MS of Smoc-L-Ser(tBu)-OH **23** (M measured=544.09431 $[M+H]^+$, M calc.=544.09418).

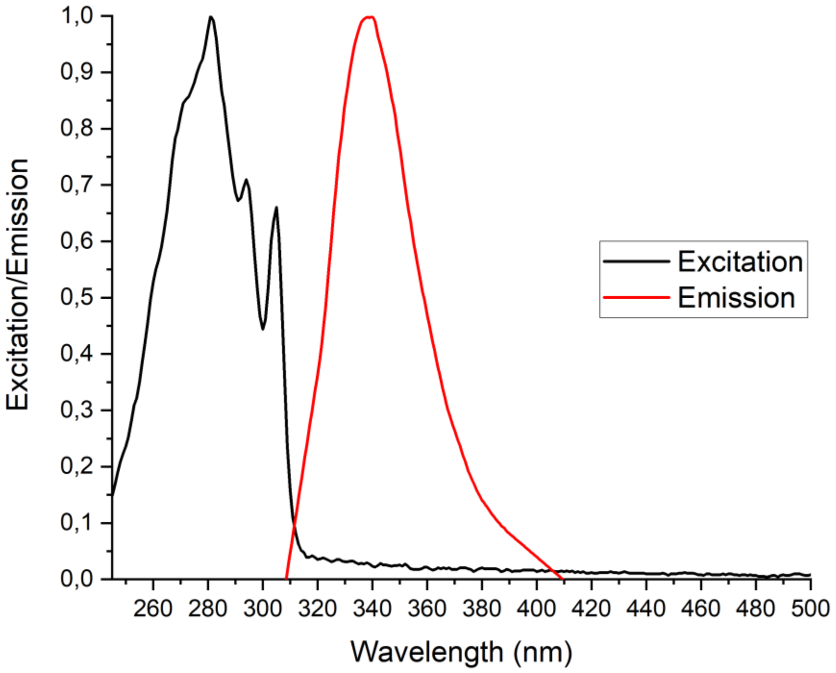

Figure S272: Excitation and emission spectra of Smoc-L-Ser(tBu)-OH **23**, excitation and emission have been normalized between 0 and 1 for illustration.

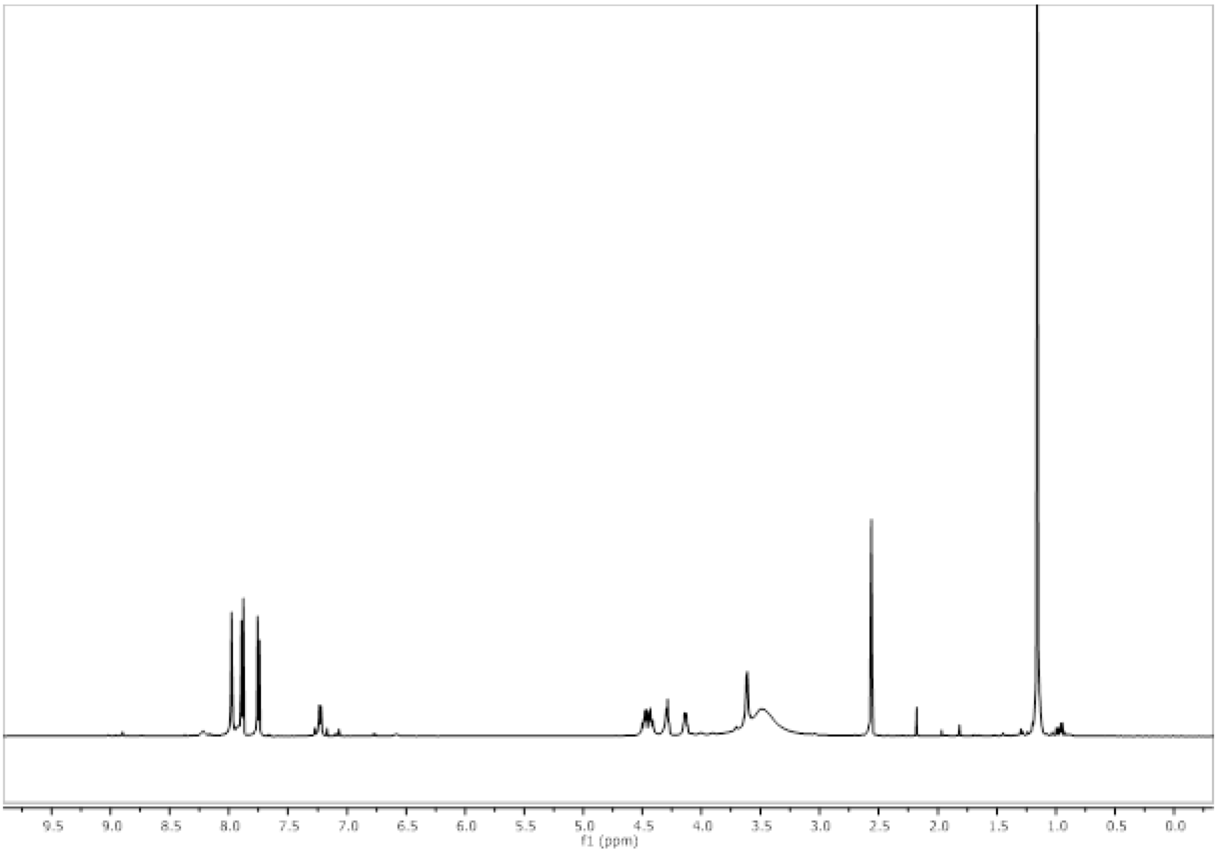

Figure S273: ^{1}H-NMR of Smoc-L-Ser(tBu)-OH **23**.

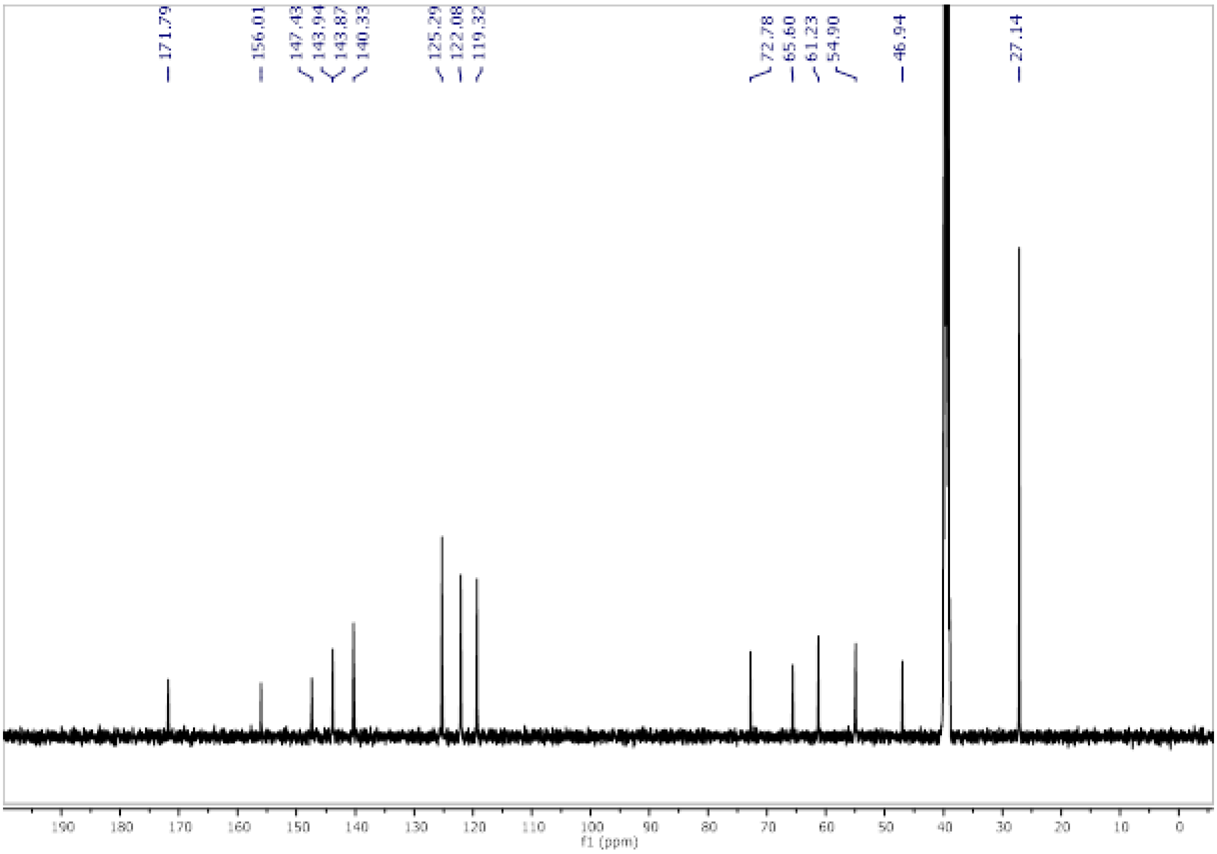

Figure S274: ^{13}C-NMR of Smoc-L-Ser(tBu)-OH **23**.

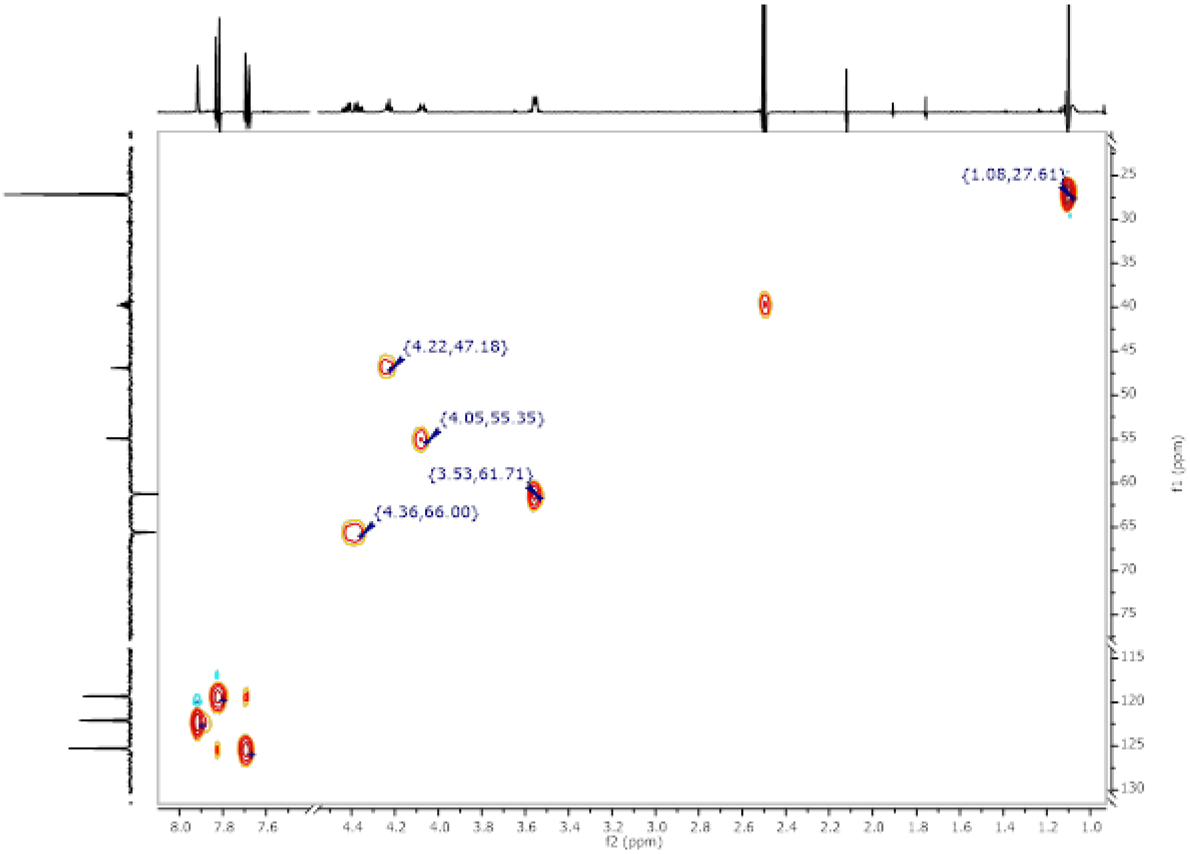

Figure S275: ^{1}H-^{13}C HSQC-NMR of Smoc-L-Ser(tBu)-OH **23**.

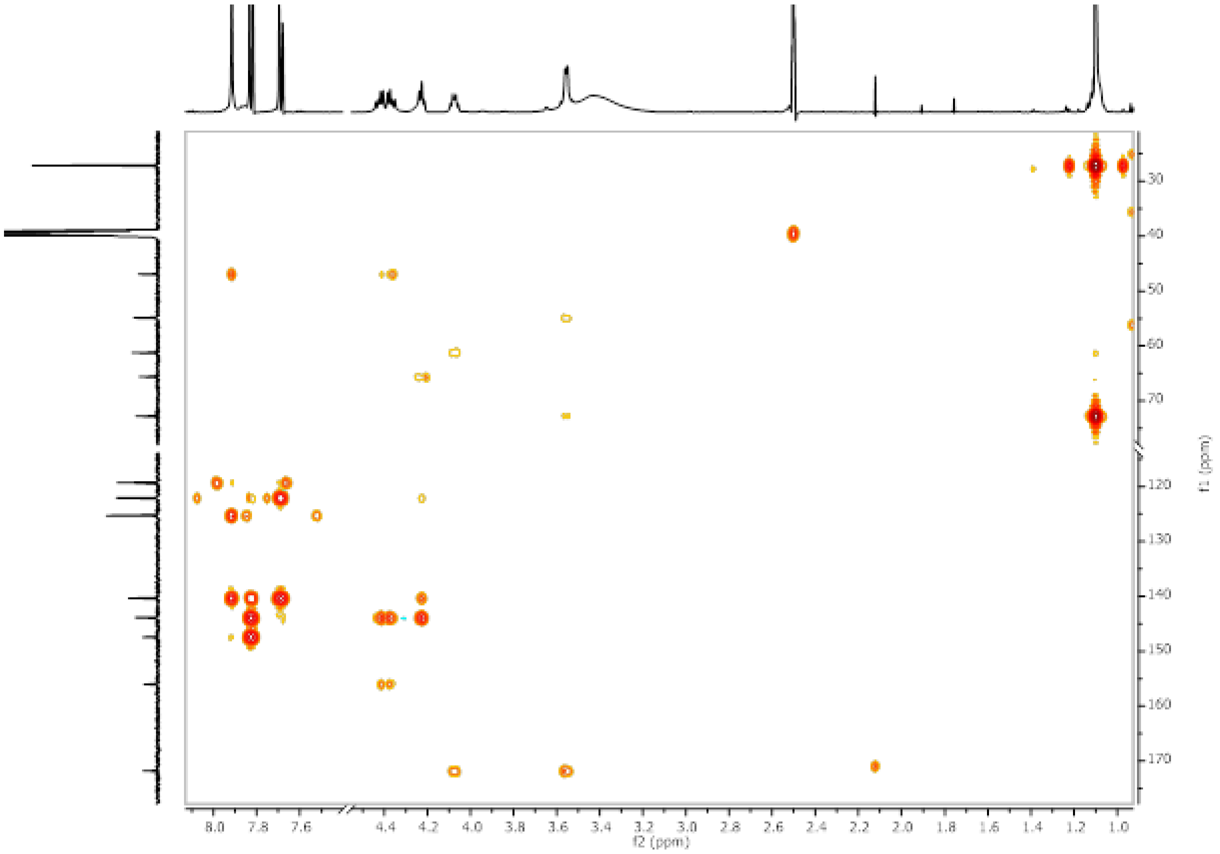

Figure S276: ^{1}H-^{13}C HMBC-NMR of Smoc-L-Ser(tBu)-OH **23**.

3.2.22. Analytical data of Smoc-L-Thr-OH 24

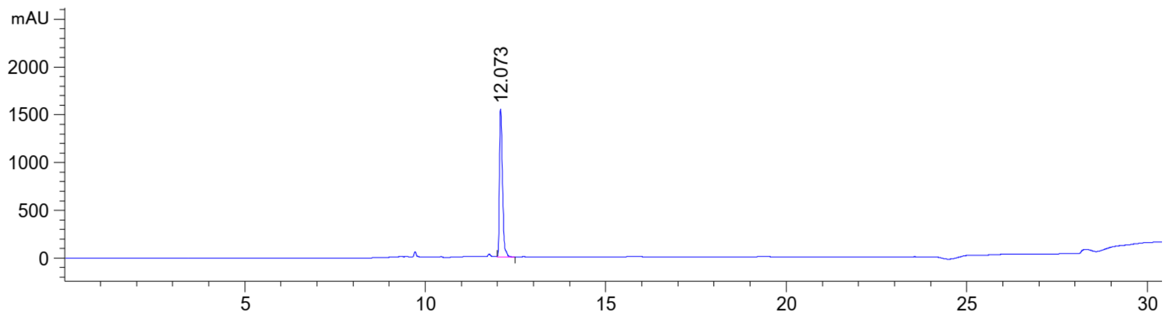

Figure S277: HPLC chromatogram of Smoc-L-Thr-OH **24** at λ=220 nm (0to40 MeCN).

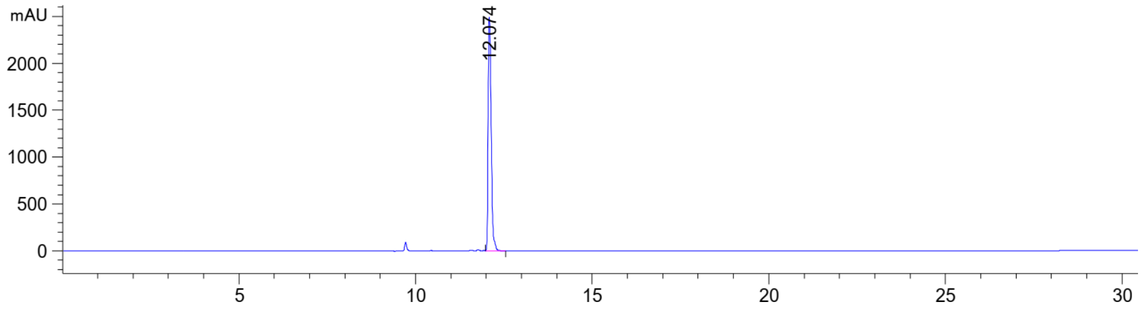

Figure S278: HPLC chromatogram of Smoc-L-Thr-OH **24** at λ=280 nm (0to40 MeCN).

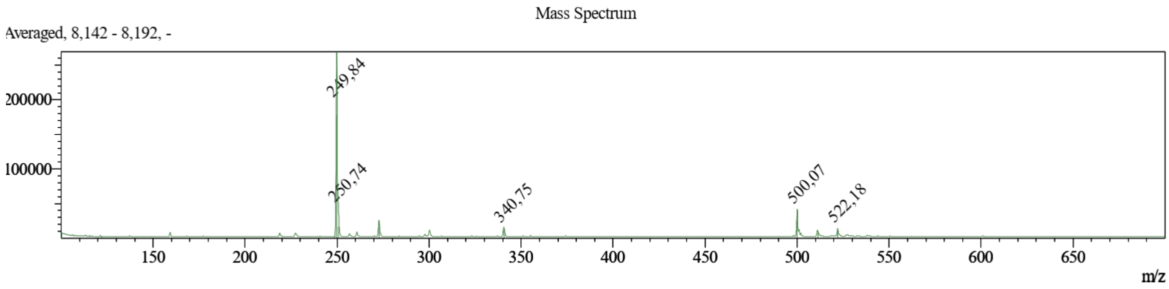

Figure S279: ESI-MS of Smoc-L-Thr-OH **24** (M measured=500.07 [M-H]$^{-}$, M calc.=501.48).

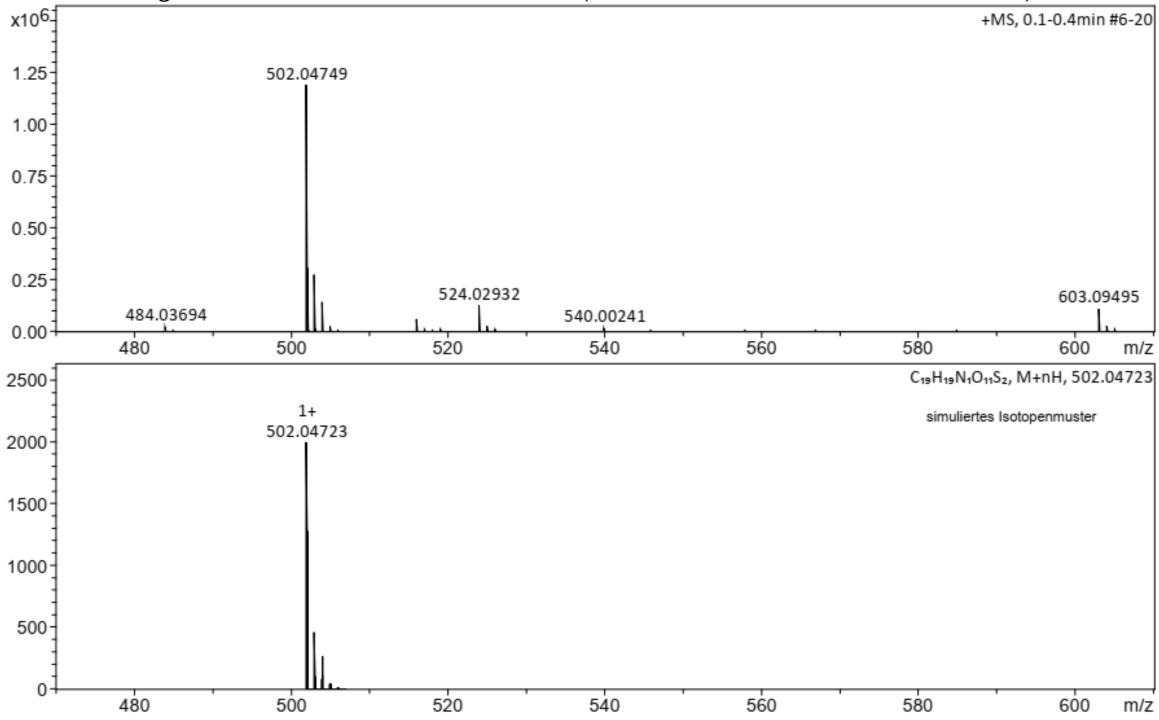

Figure S280: HR-MS of Smoc-L-Thr-OH **24** (M measured=502.04749 [M+H]$^{+}$, M calc.=502.04723).

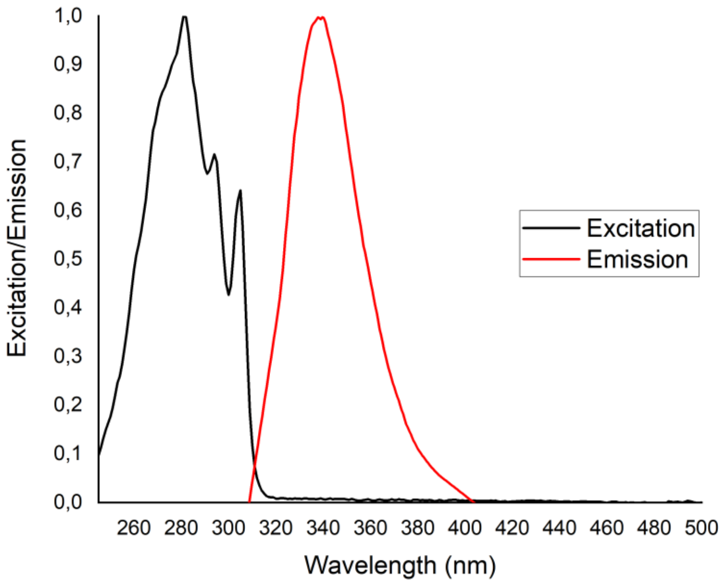

Figure S281: Excitation and emission spectra of Smoc-L-Thr-OH **24**, excitation and emission have been normalized between 0 and 1 for illustration.

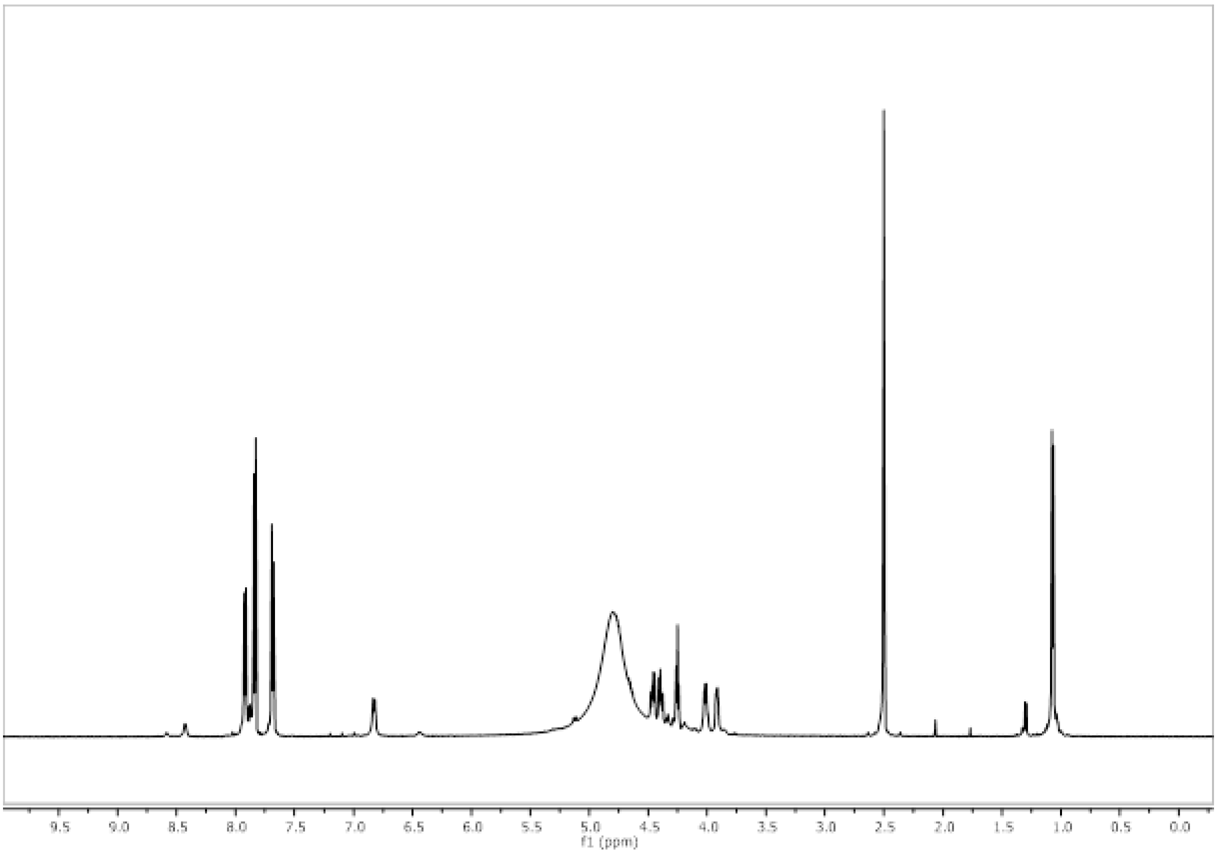

Figure S282: ^{1}H-NMR of Smoc-L-Thr-OH **24**.

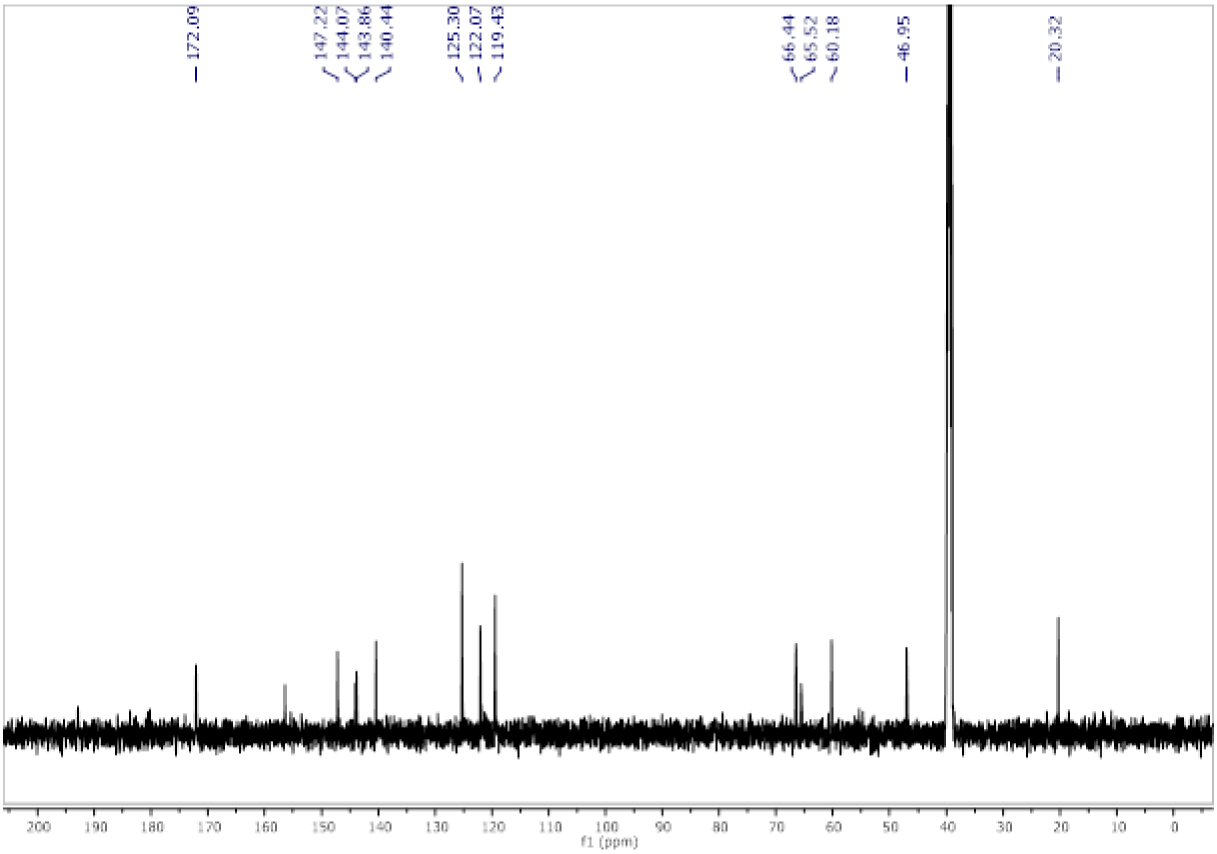

Figure S283: ^{13}C-NMR of Smoc-L-Thr-OH **24**.

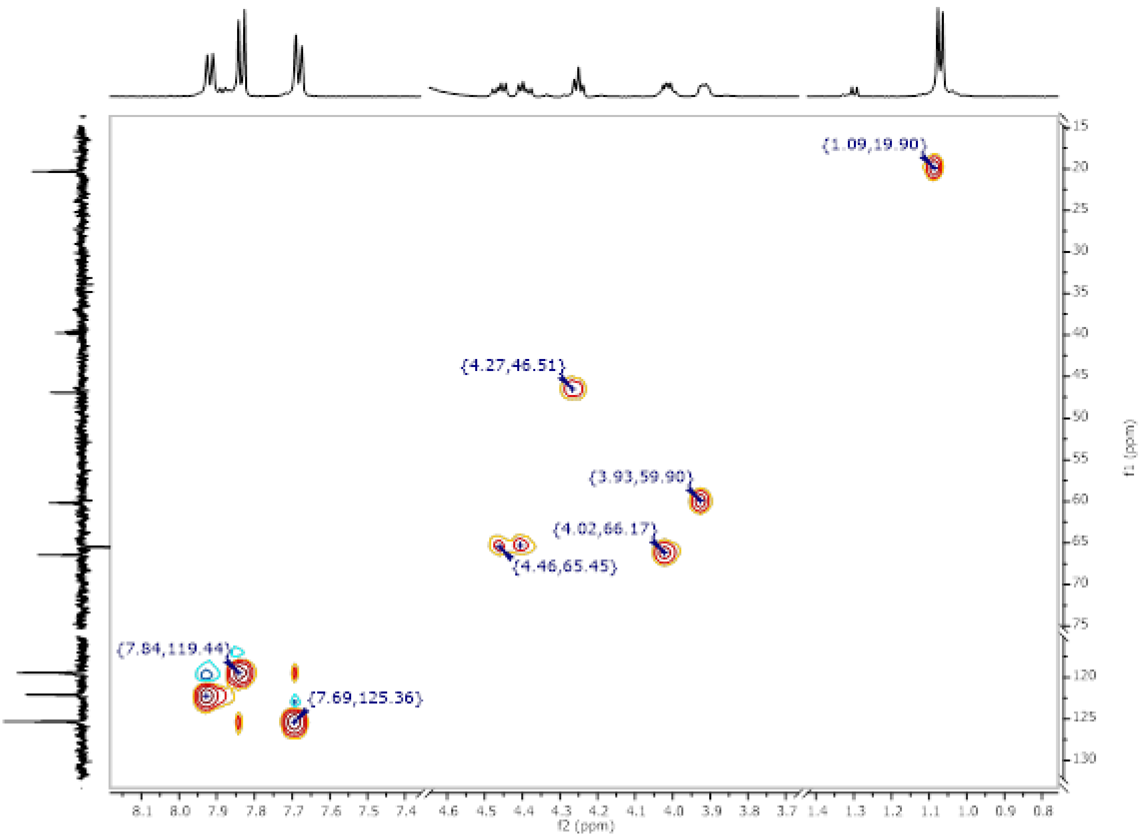

Figure S284: ^{1}H-^{13}C HSQC-NMR of Smoc-L-Thr-OH **24**.

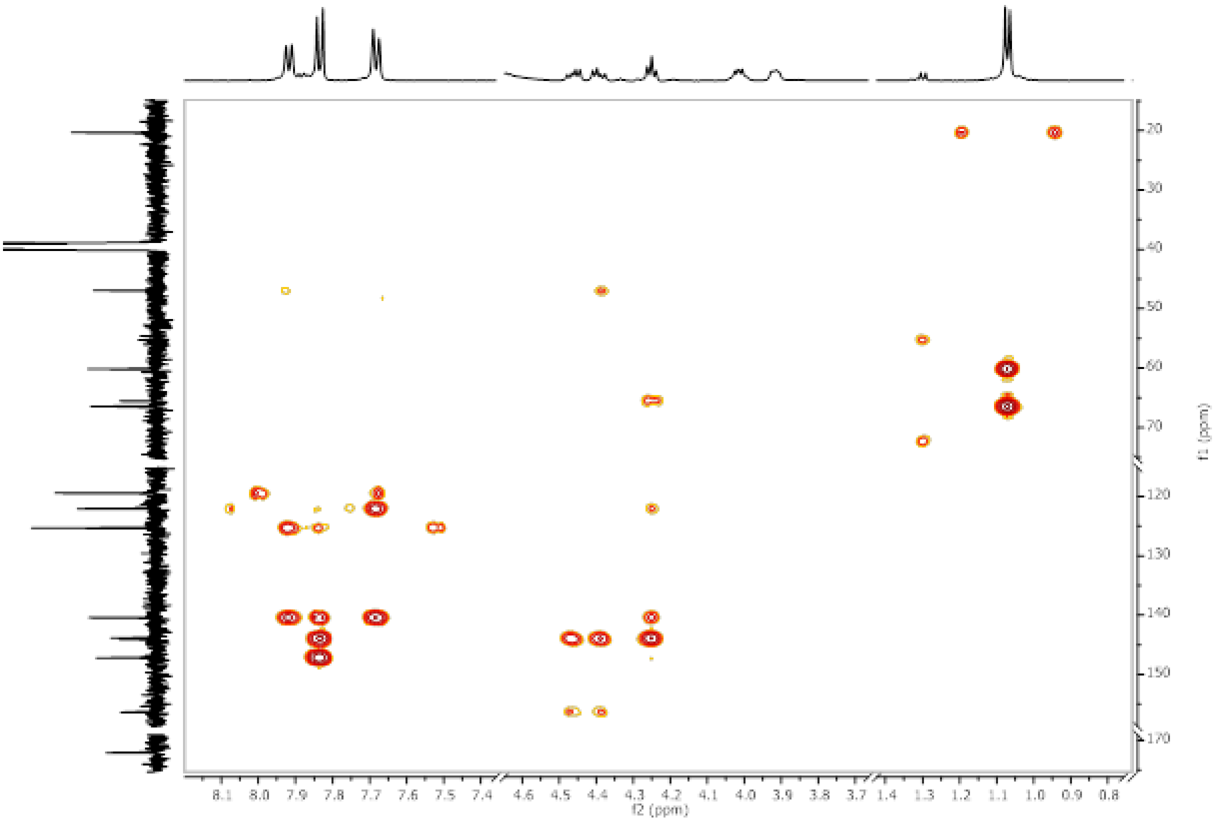

Figure S285: ^{1}H-^{13}C HMBC-NMR of Smoc-L-Thr-OH **24**.

3.2.23. Analytical data of Smoc-L-Thr(tBu)-OH 25

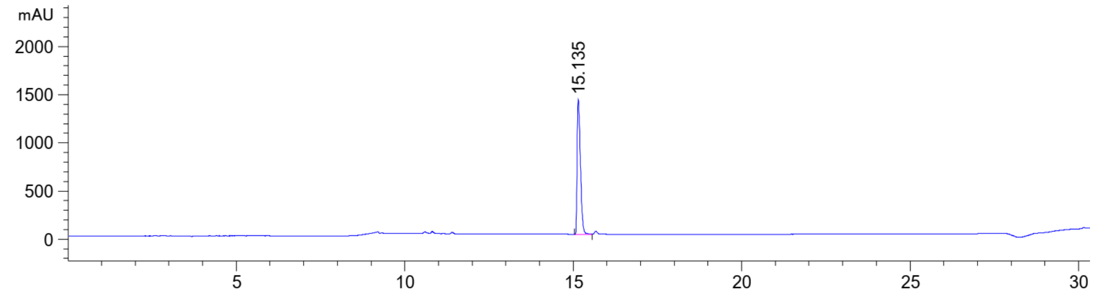

Figure S286: HPLC chromatogram of Smoc-L-Thr(tBu)-OH **25** at λ=220 nm (0to60 MeCN).

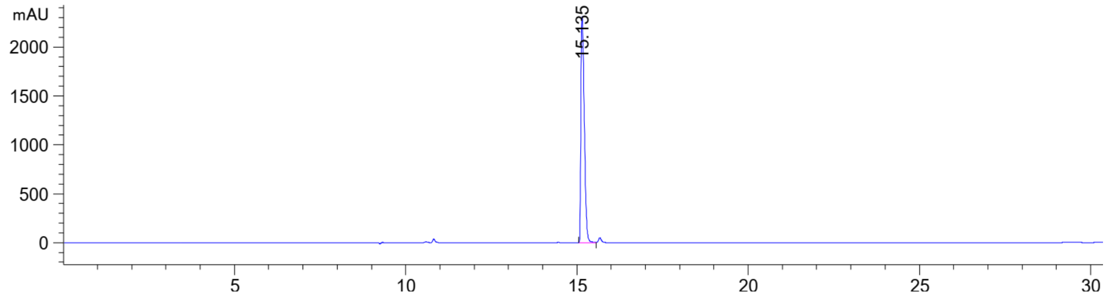

Figure S287: HPLC chromatogram of Smoc-L-Thr(tBu)-OH **25** at λ=280 nm (0to60 MeCN).

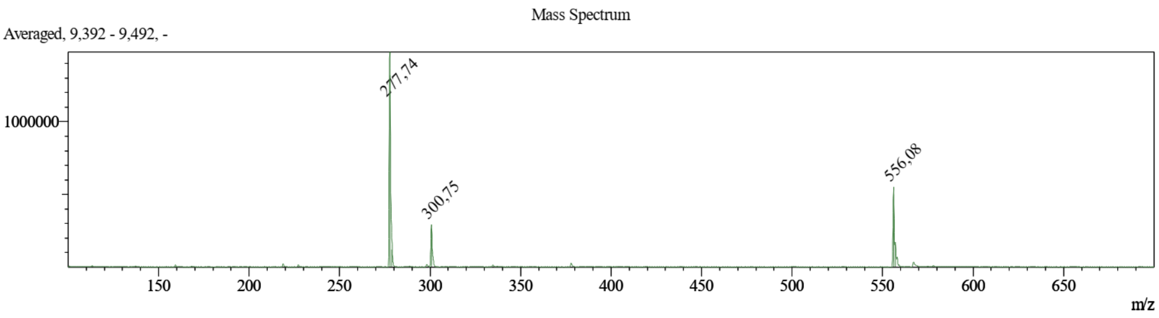

Figure S288: ESI-MS of Smoc-L-Thr(tBu)-OH **25** (M measured=556.08 [M-H]$^-$, M calc.=557.59).

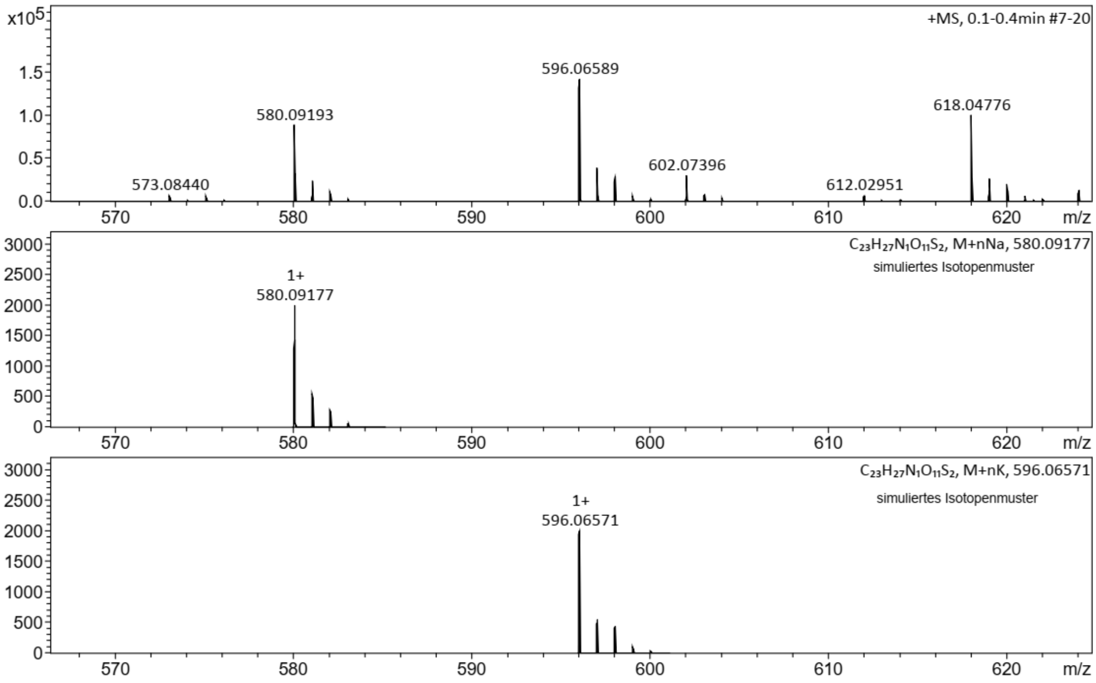

Figure S289: HR-MS of Smoc-L-Thr(tBu)-OH **25** (M measured=580.09193 [M+Na]$^+$, M calc.=580.09177; M measured=596.06589 [M+K]$^+$, M calc.=596.06571).

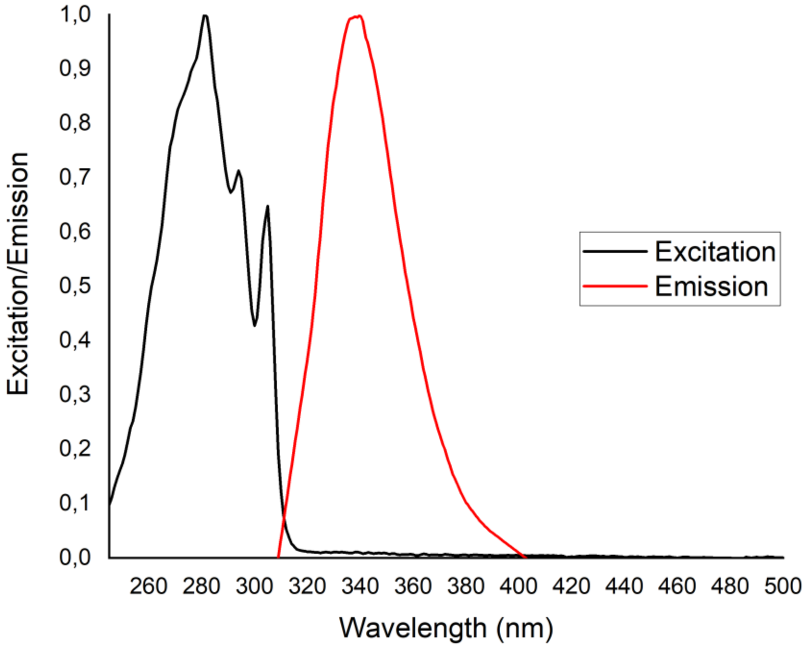

Figure S290: Excitation and emission spectra of Smoc-L-Thr(tBu)-OH **25**, excitation and emission have been normalized between 0 and 1 for illustration.

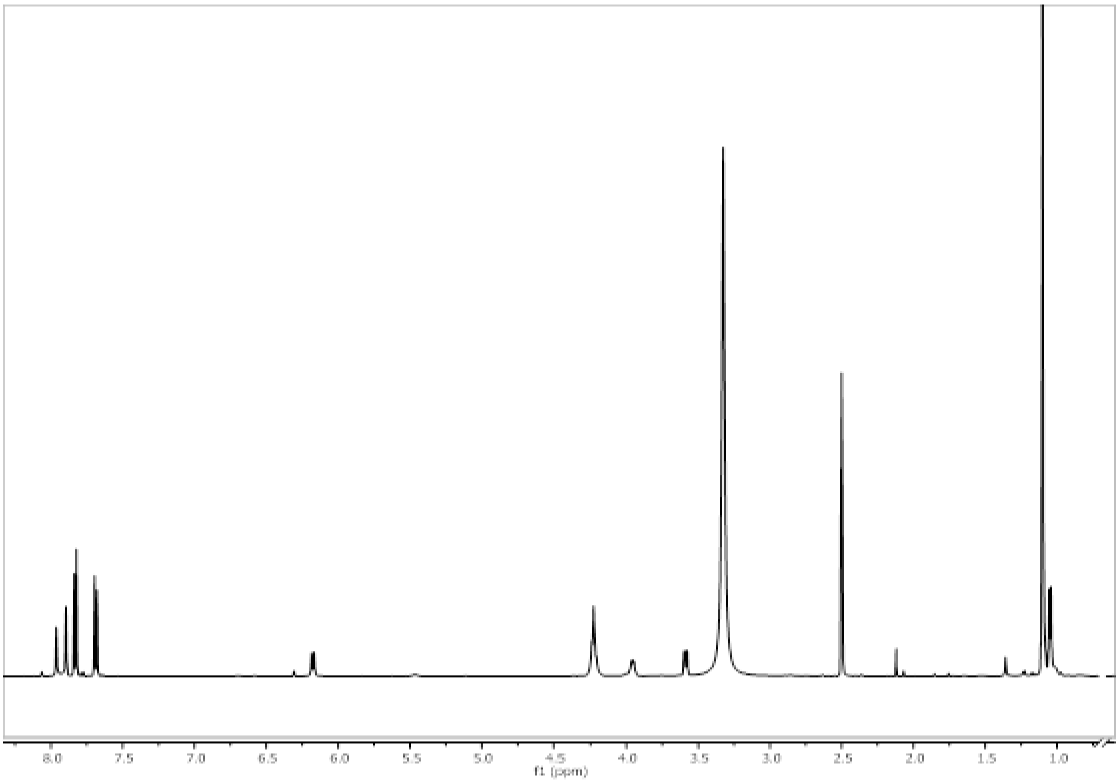

Figure S291: ^{1}H-NMR of Smoc-L-Thr(tBu)-OH **25**.

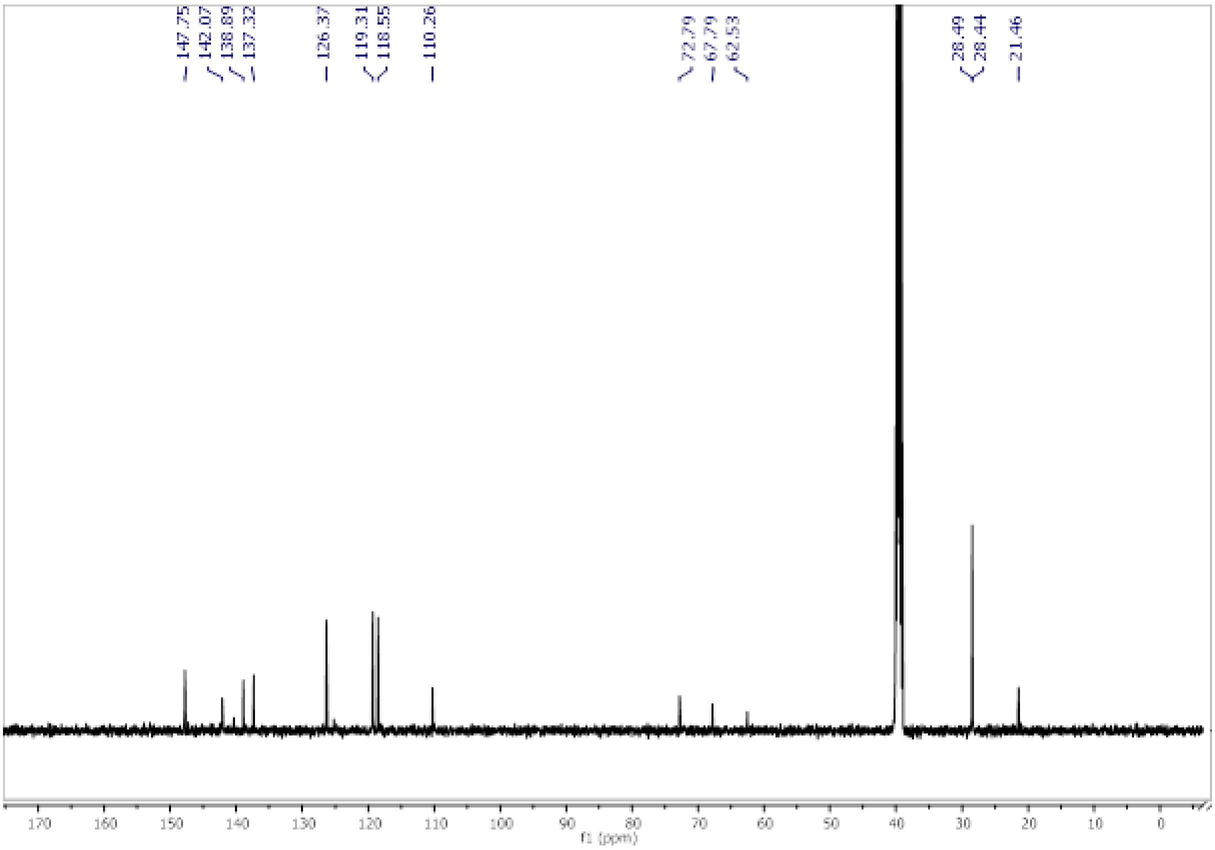

Figure S292: ^{13}C-NMR of Smoc-L-Thr(tBu)-OH **25**.

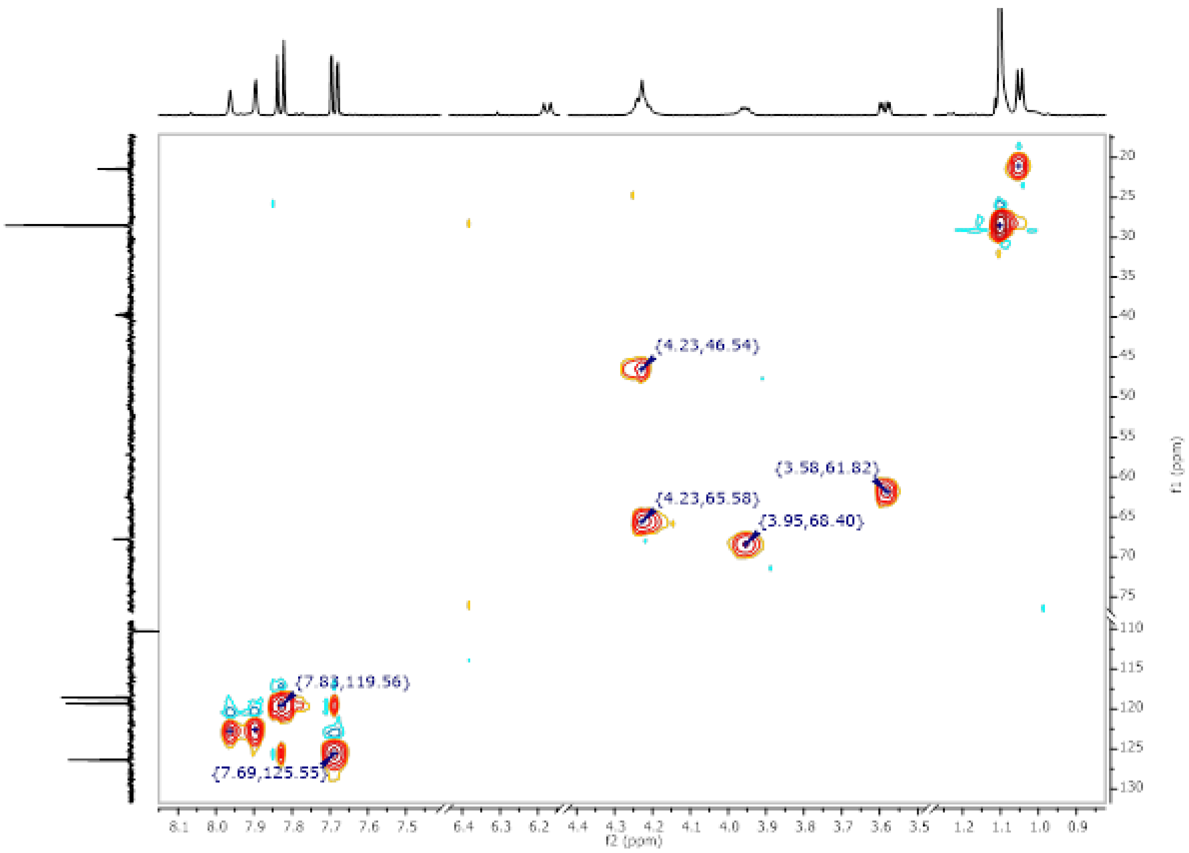

Figure S293: ^{1}H-^{13}C HSQC-NMR of Smoc-L-Thr(tBu)-OH **25**.

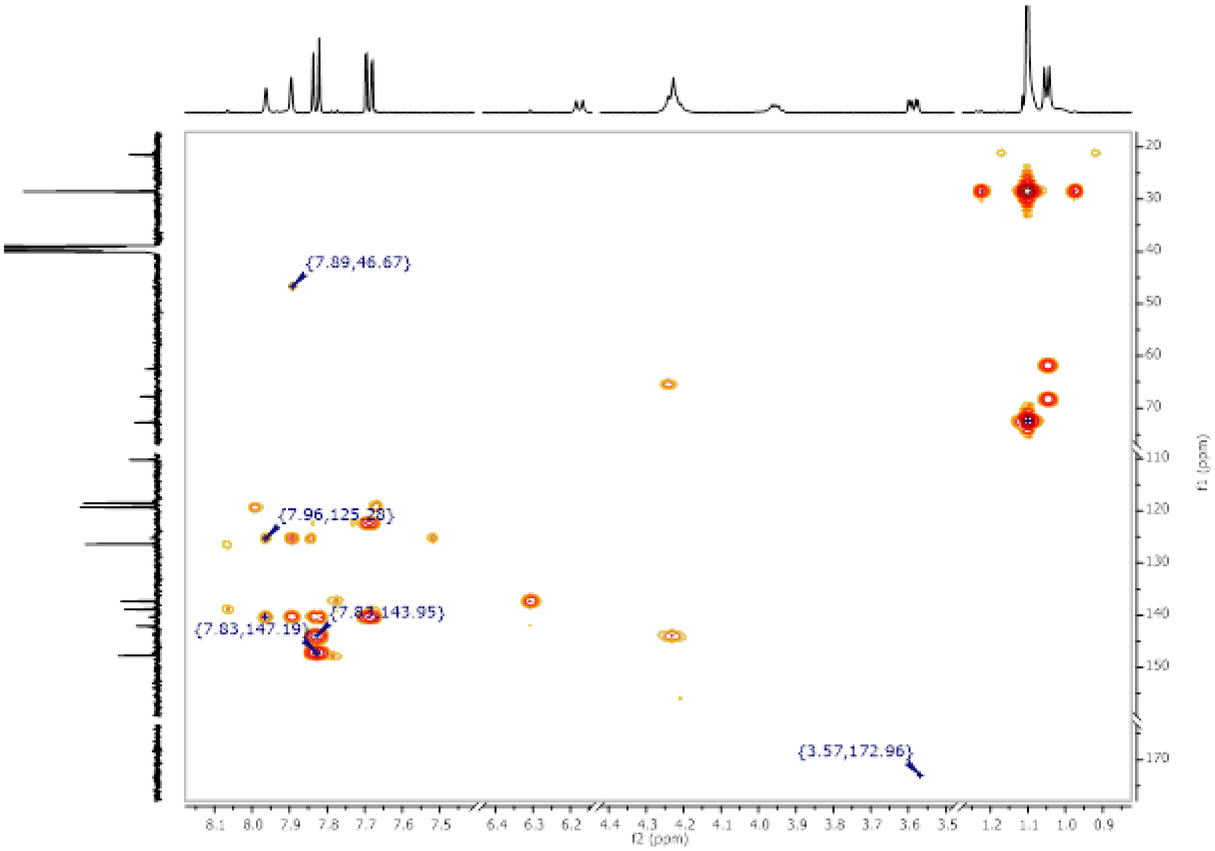

Figure S294: ^{1}H-^{13}C HMBC-NMR of Smoc-L-Thr(tBu)-OH **25**.

3.2.24. Analytical data of Smoc-L-Trp-OH 26

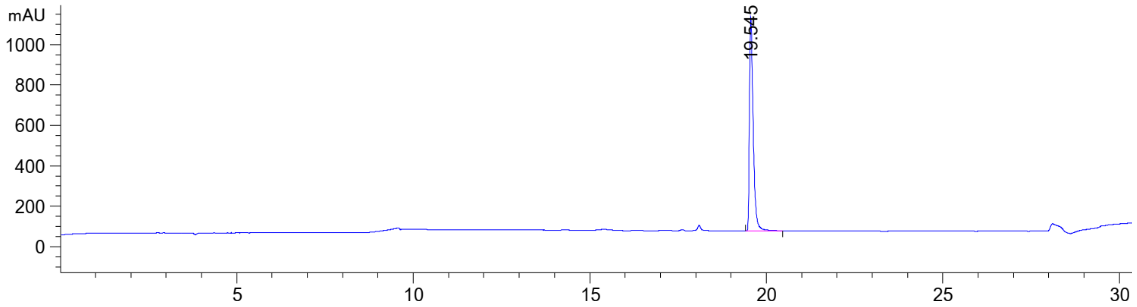

Figure S295: HPLC chromatogram of Smoc-L-Trp-OH **26** at λ=220 nm (0to40 MeCN).

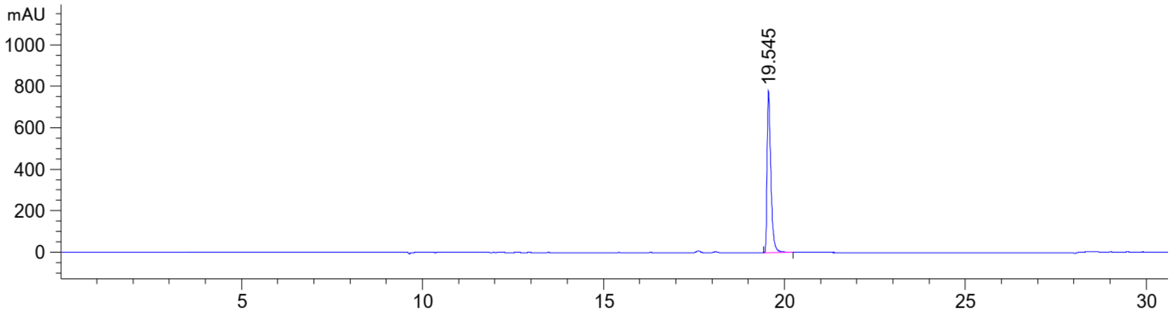

Figure S296: HPLC chromatogram of Smoc-L-Trp-OH **26** at λ=280 nm (0to40 MeCN).

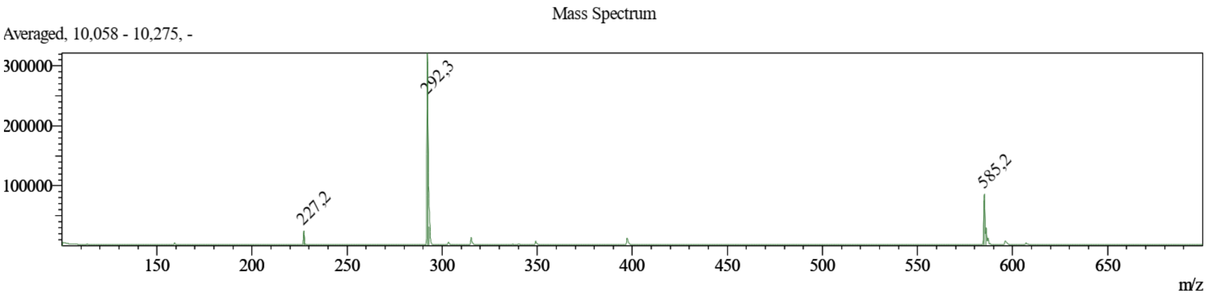

Figure S297: ESI-MS of Smoc-L-Trp-OH **26** (M measured=585.20 [M-H]$^-$, M calc.=586.59).

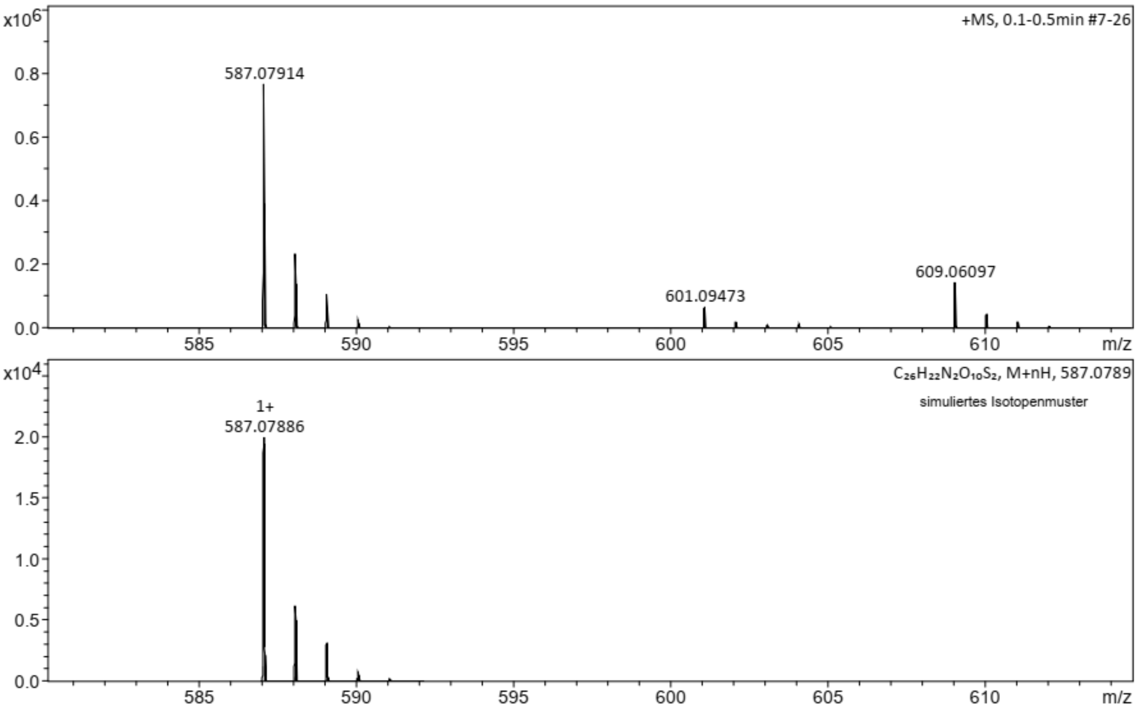

Figure S298: HR-MS of Smoc-L-Trp-OH **26** (M measured=587.07914 [M+H]$^+$, M calc.=587.07886).

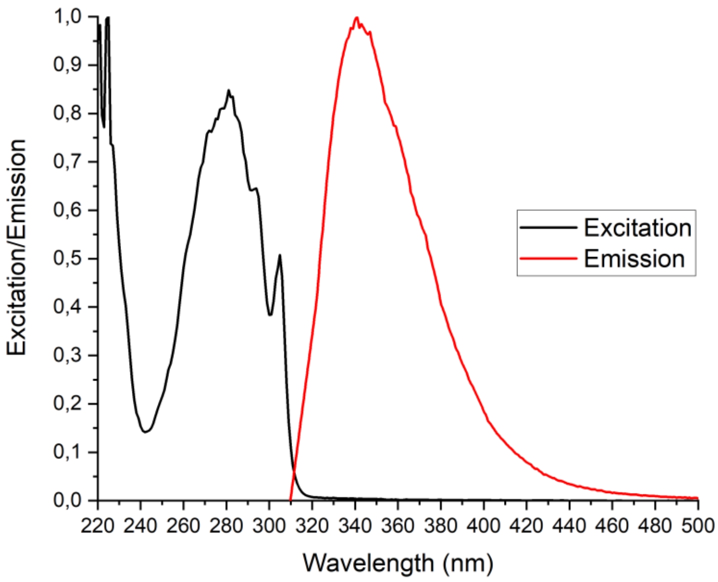

Figure S299: Excitation and emission spectra of Smoc-L-Trp-OH **26**, excitation and emission have been normalized between 0 and 1 for illustration.

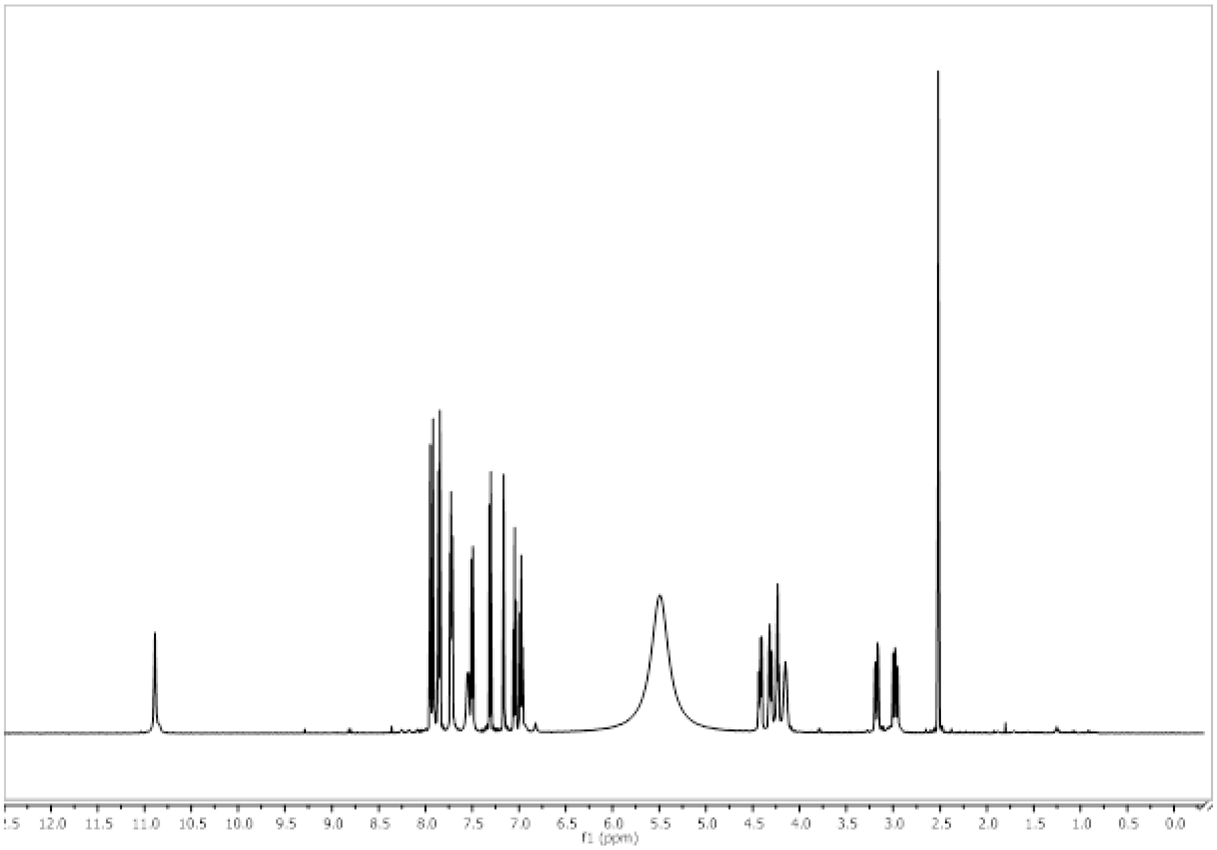

Figure S300: ^{1}H-NMR of Smoc-L-Trp-OH **26**.

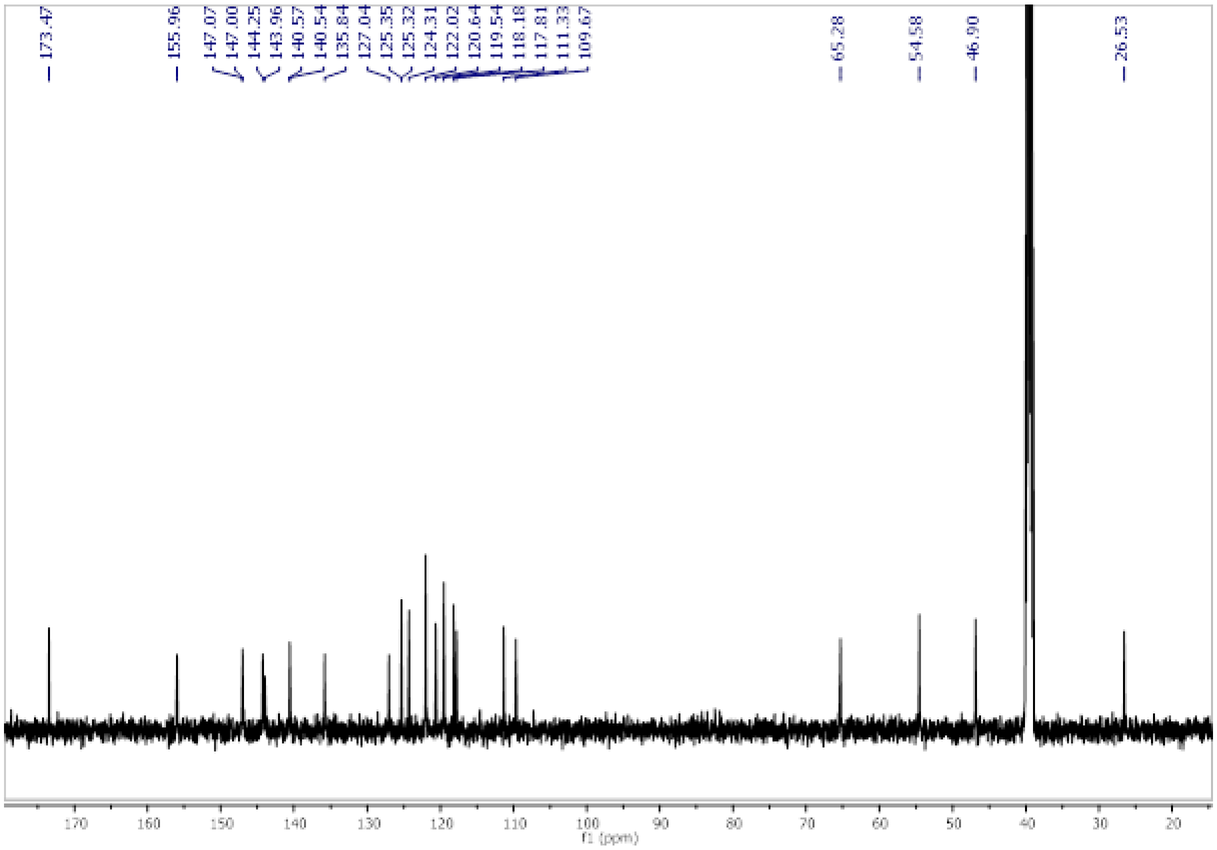

Figure S301: ^{13}C-NMR of Smoc-L-Trp-OH **26**.

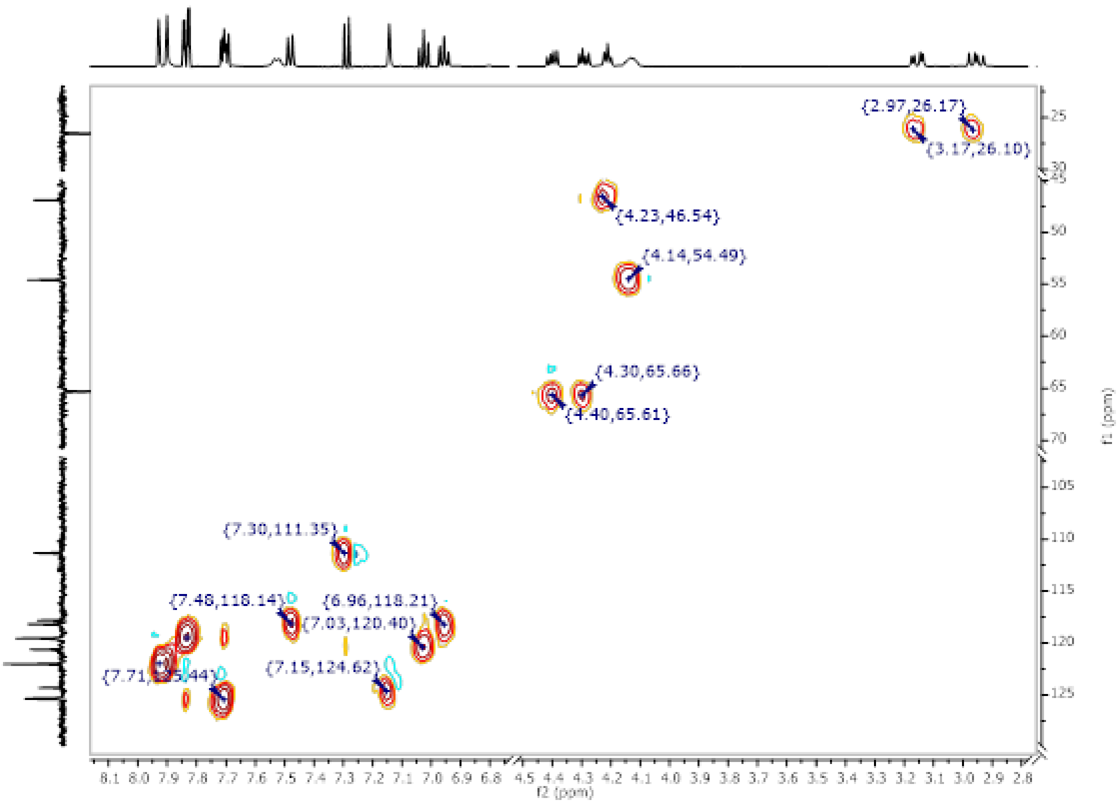

Figure S302: ^{1}H-^{13}C HSQC-NMR of Smoc-L-Trp-OH **26**.

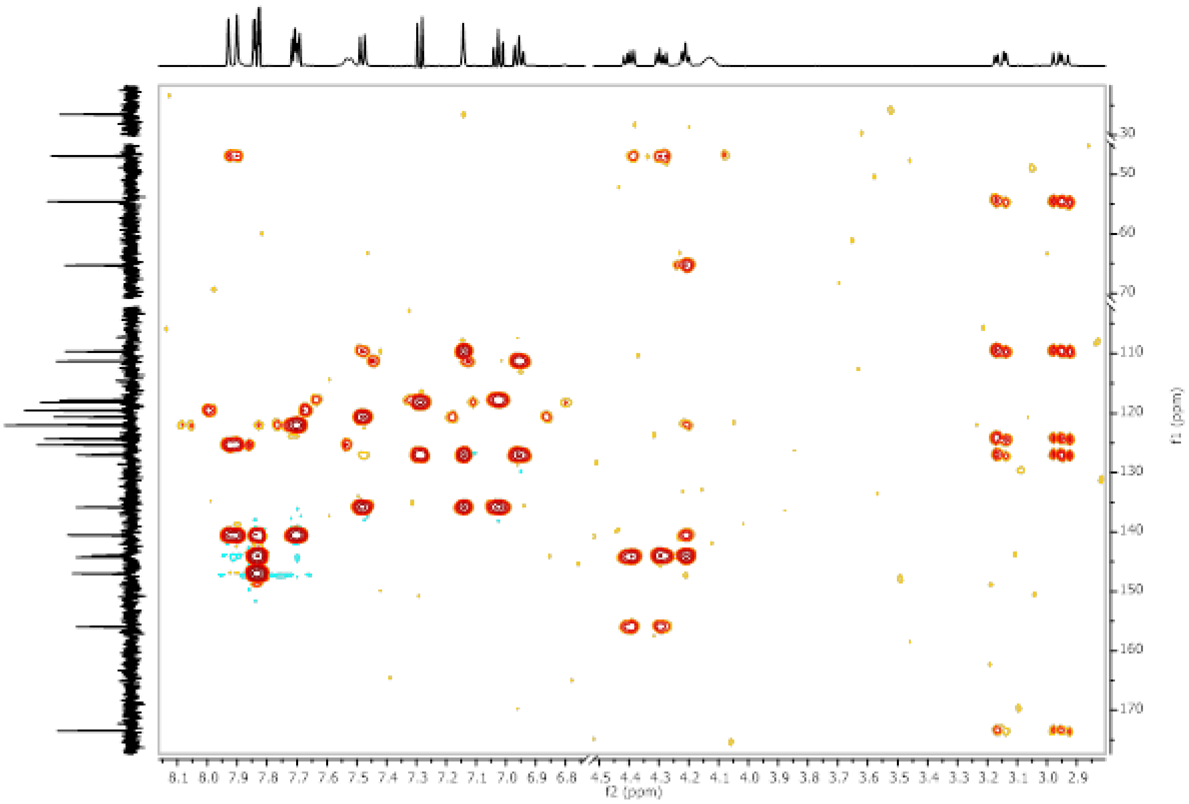

Figure S303: ^{1}H-^{13}C HMBC-NMR of Smoc-L-Trp-OH **26**.

3.2.25. Analytical data of Smoc-L-Trp(Boc)-OH 27

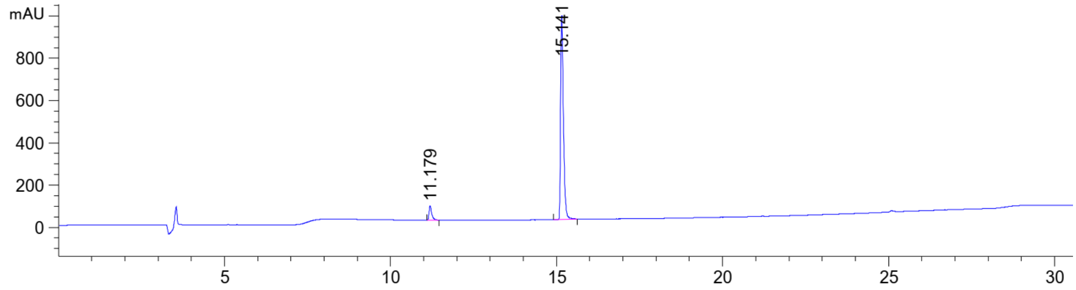

Figure S304: HPLC chromatogram of Smoc-L-Trp(Boc)-OH **27** at λ=220 nm (0to100 MeCN).

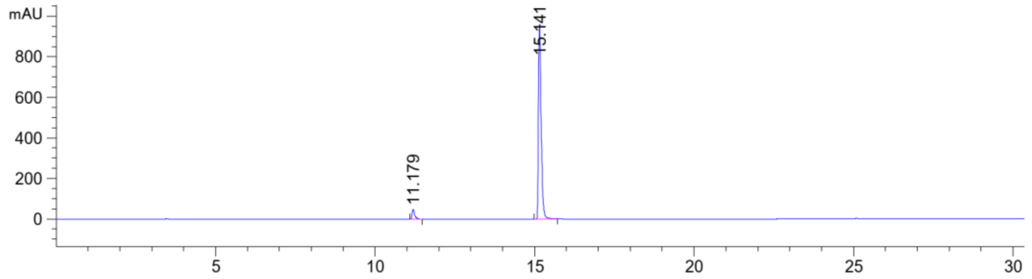

Figure S305: HPLC chromatogram of Smoc-L-Trp(Boc)-OH **27** at λ=280 nm (0to100 MeCN).

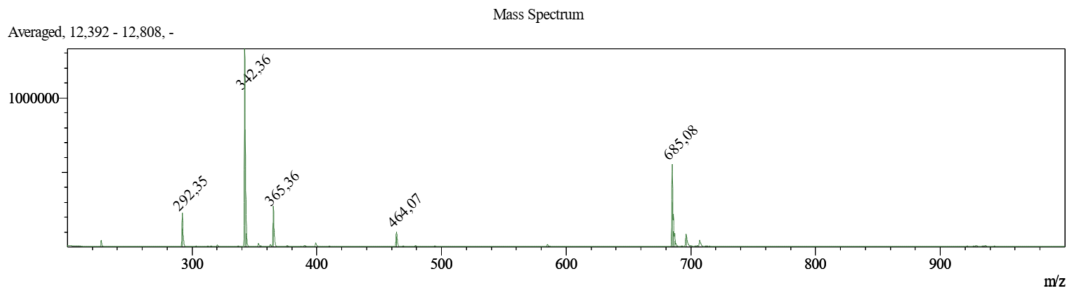

Figure S306: ESI-MS of Smoc-L-Trp(Boc)-OH **27** (M measured=685.08 [M-H]$^-$, M calc.=686.70).

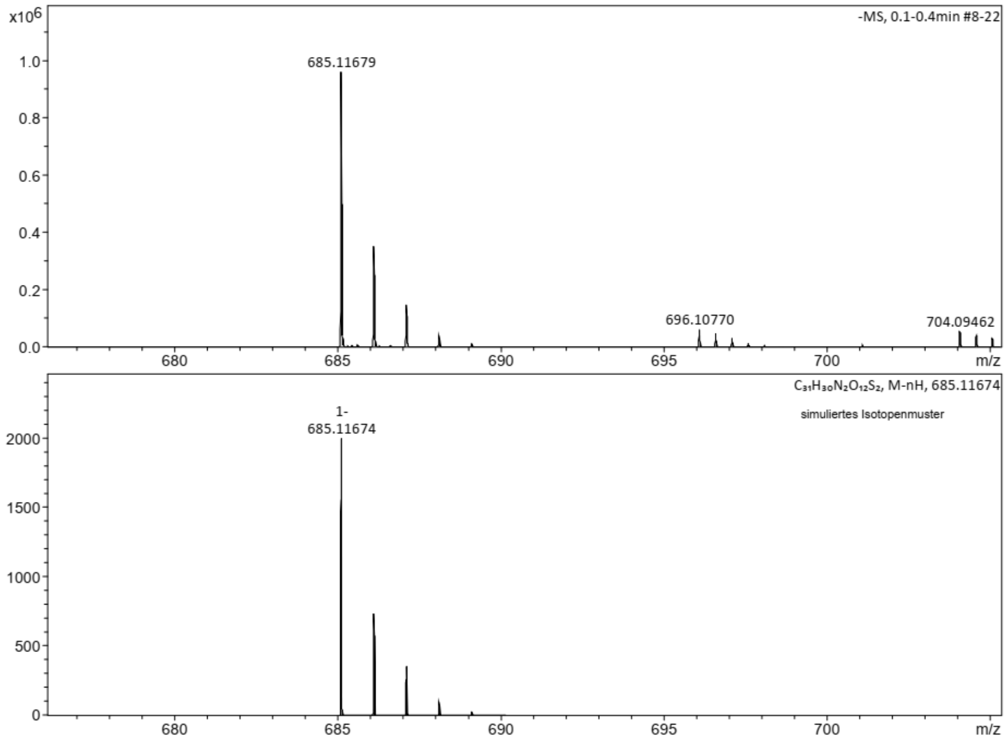

Figure S307: HR-MS of Smoc-L-Trp(Boc)-OH **27** (M measured=685.11679 [M-H]$^-$, M calc.=685.11674).

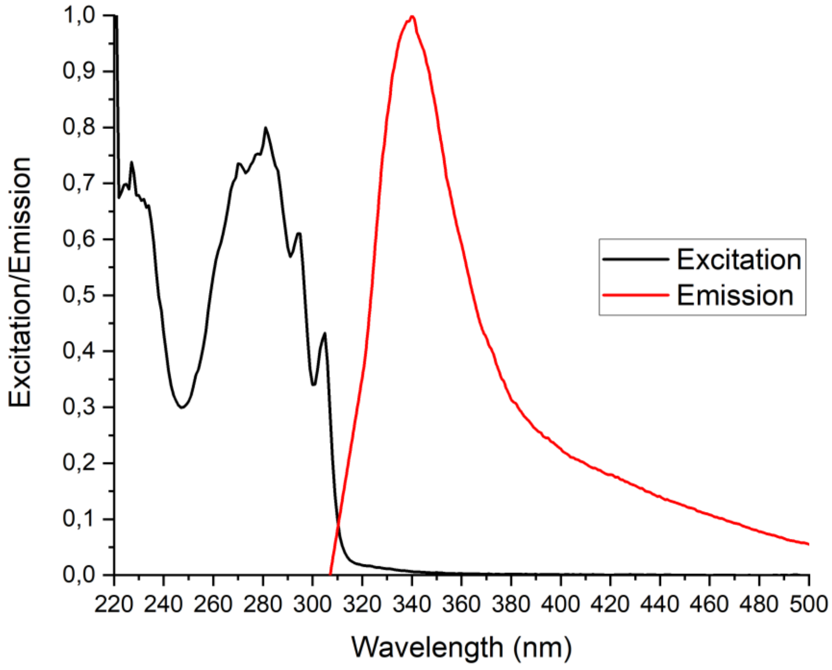

Figure S308: Excitation and emission spectra of Smoc-L-Trp(Boc)-OH **27**, excitation and emission have been normalized between 0 and 1 for illustration.

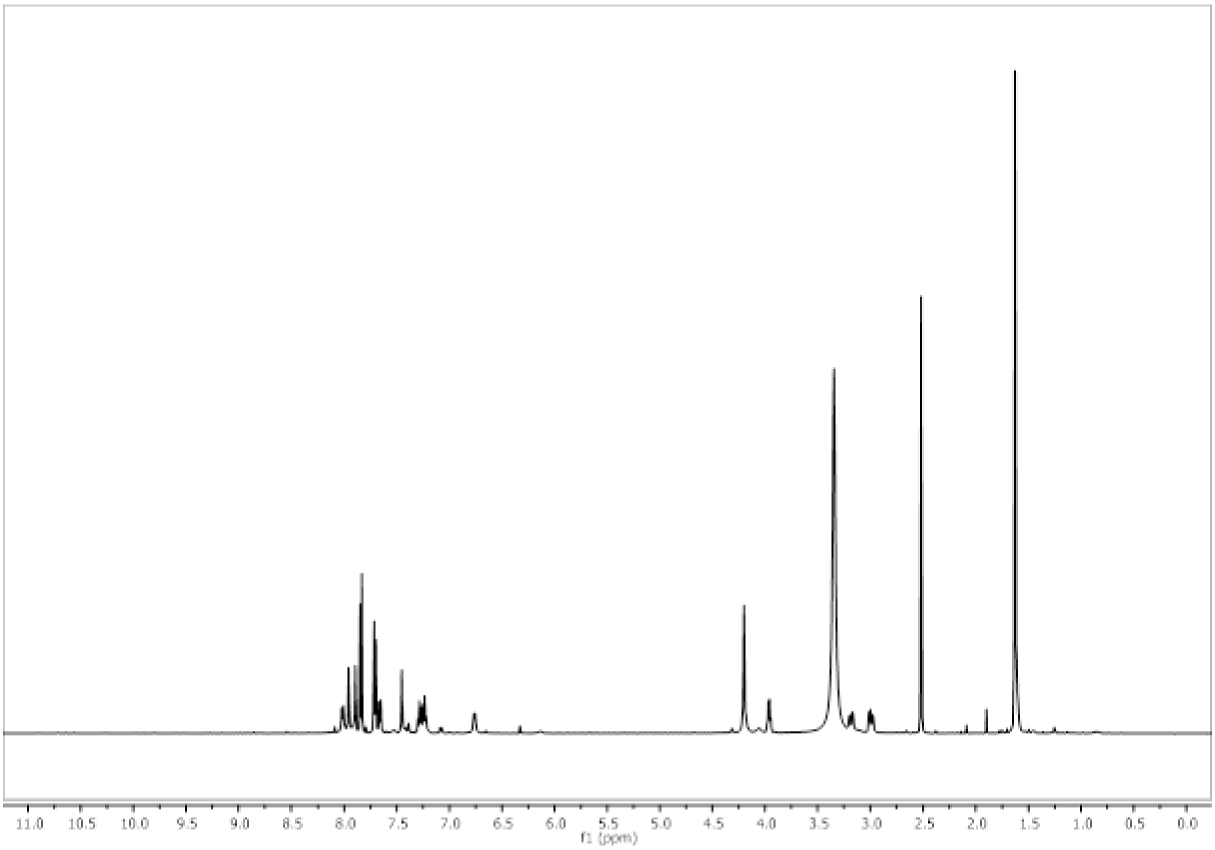

Figure S309: ^{1}H-NMR of Smoc-L-Trp(Boc)-OH **27**.

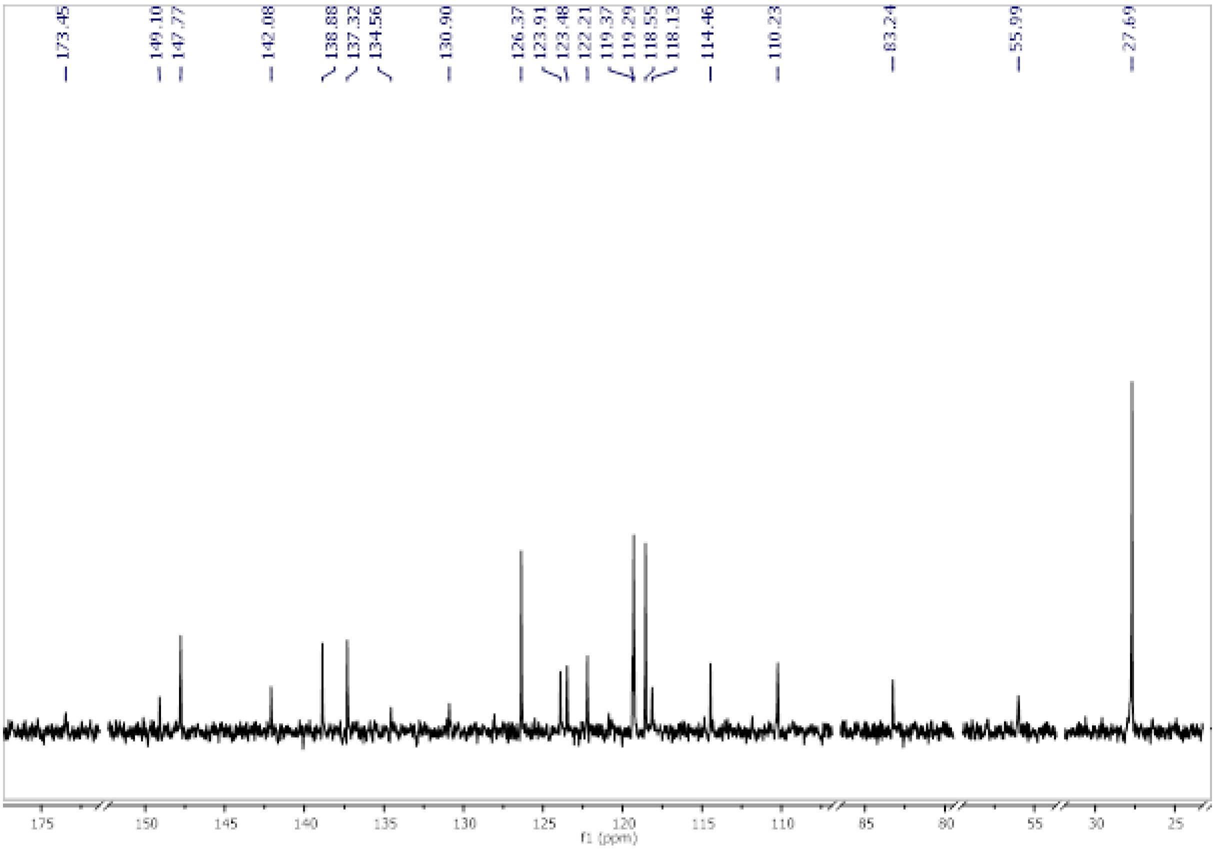

Figure S310: ^{13}C-NMR of Smoc-L-Trp(Boc)-OH **27**.

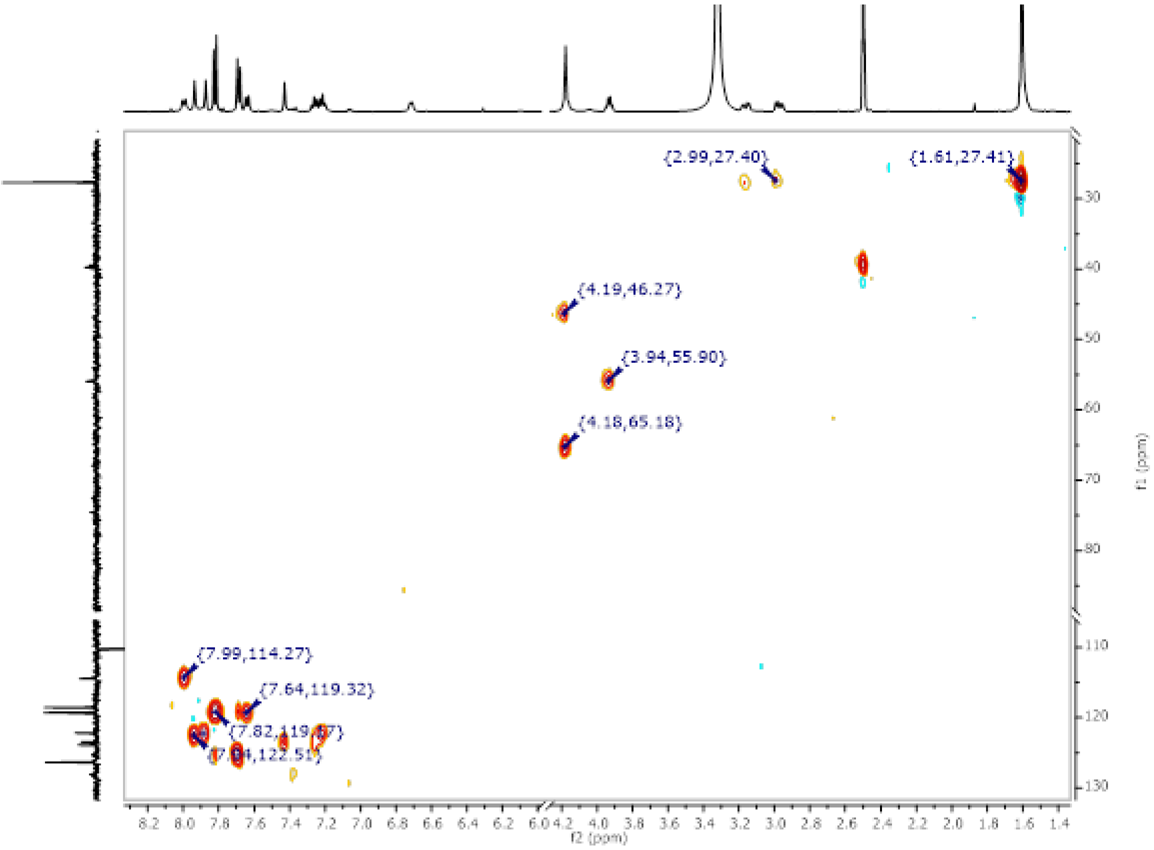

Figure S311: ^{1}H-^{13}C HSQC-NMR of Smoc-L-Trp(Boc)-OH **27**.

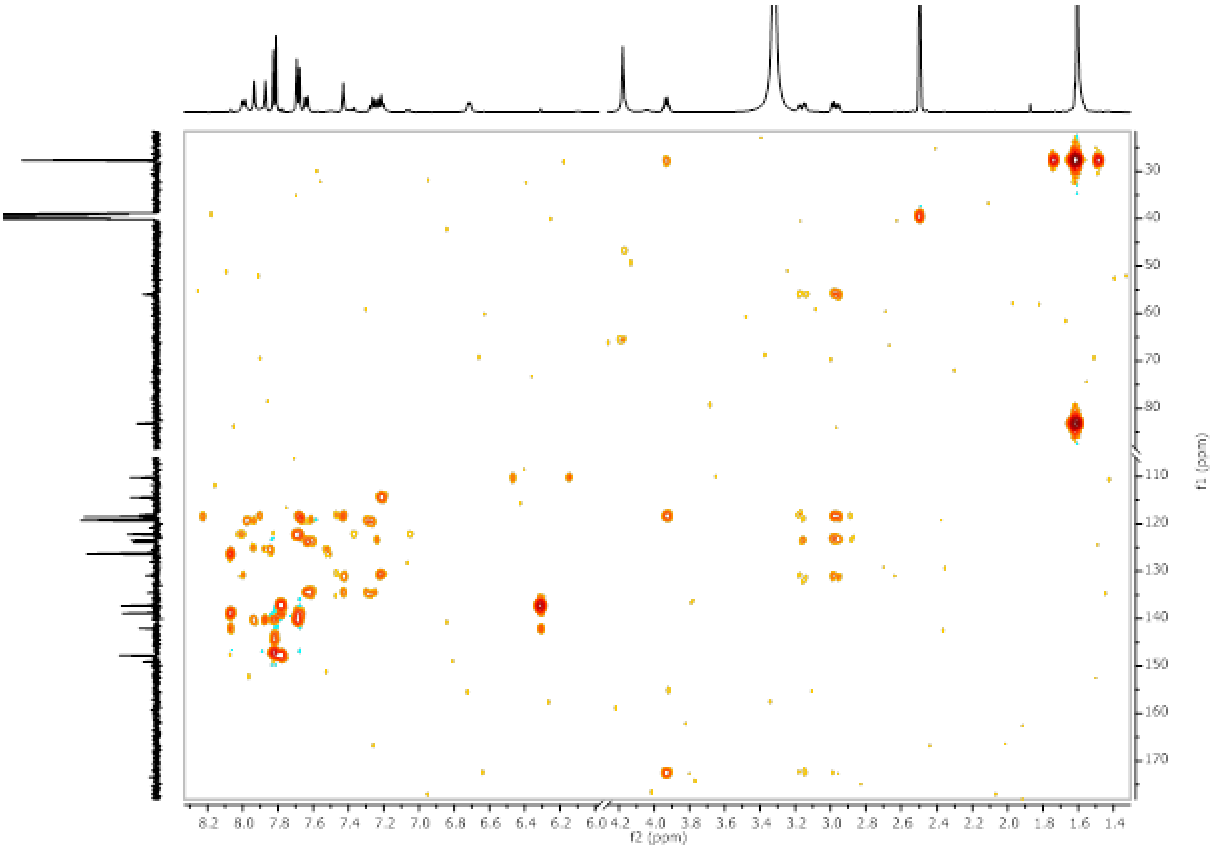

Figure S312: ^{1}H-^{13}C HMBC-NMR of Smoc-L-Trp(Boc)-OH **27**.

3.2.26. Analytical data of Smoc-L-Tyr-OH 28

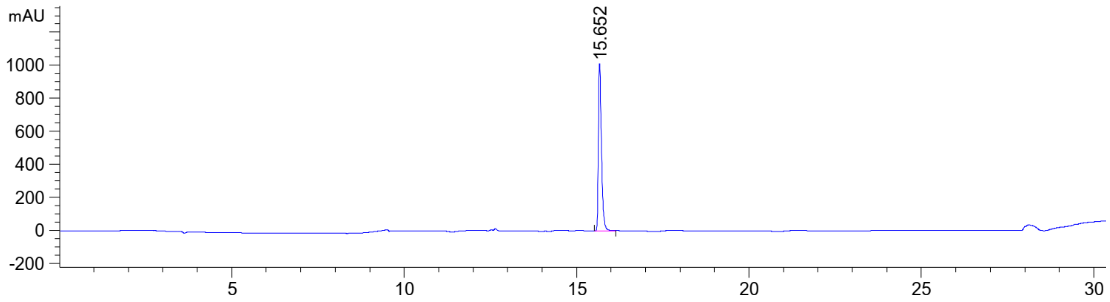

Figure S313: HPLC chromatogram of Smoc-L-Tyr-OH **28** at λ=220 nm (0to40 MeCN).

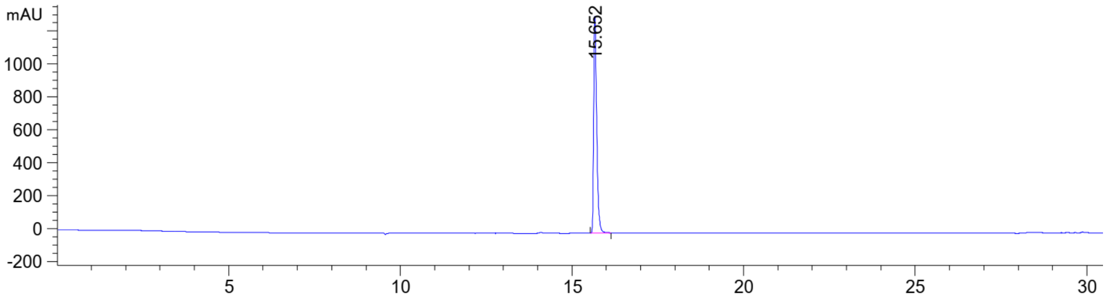

Figure S314: HPLC chromatogram of Smoc-L-Tyr-OH **28** at λ=280 nm (0to40 MeCN).

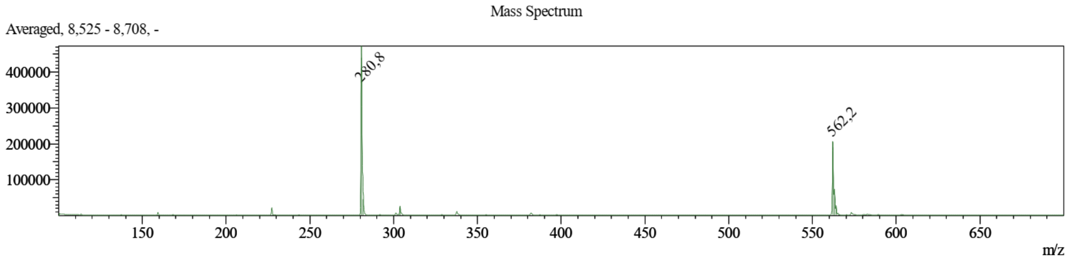

Figure S315: ESI-MS of Smoc-L-Tyr-OH **28** (M measured=562.20 [M-H]$^-$, M calc.=563.55).

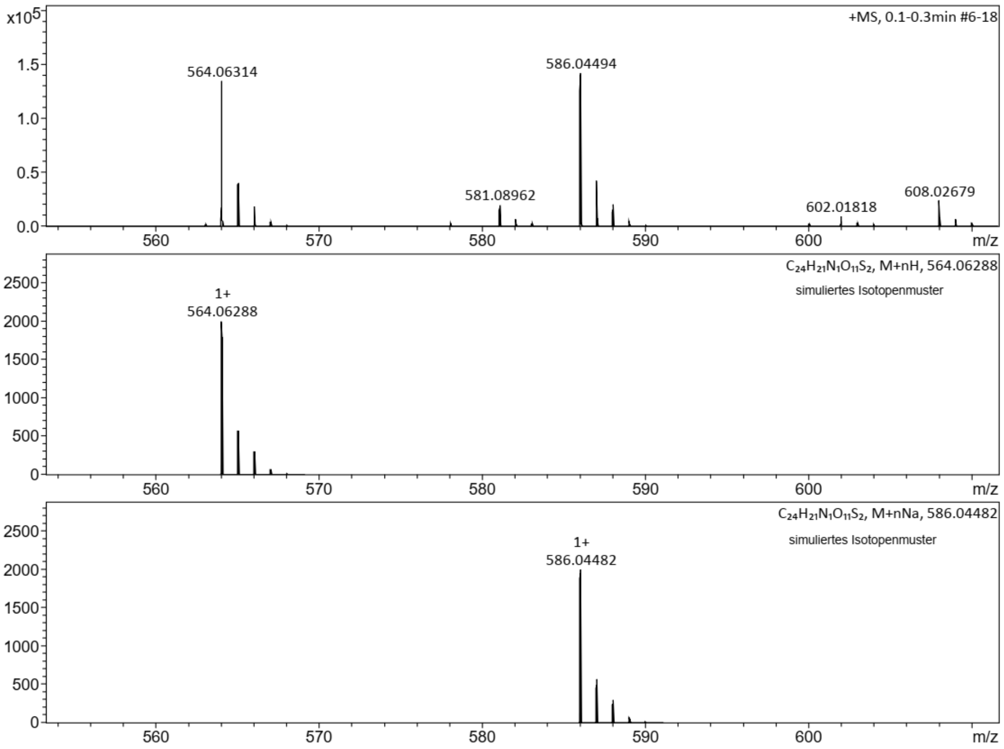

Figure S316: HR-MS of Smoc-L-Tyr-OH **28** (M measured=564.06314 [M+H]$^+$, M calc.=564.06288).

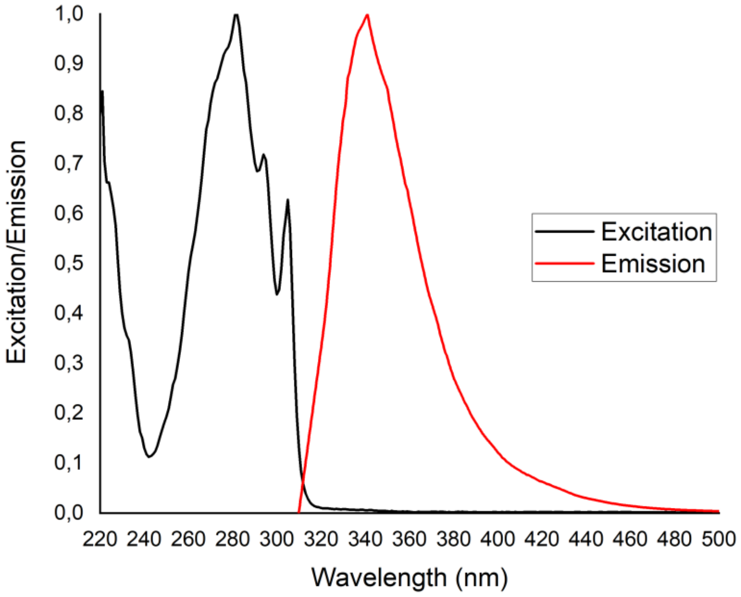

Figure S317: Excitation and emission spectra of Smoc-L-Tyr-OH **28**, excitation and emission have been normalized between 0 and 1 for illustration.

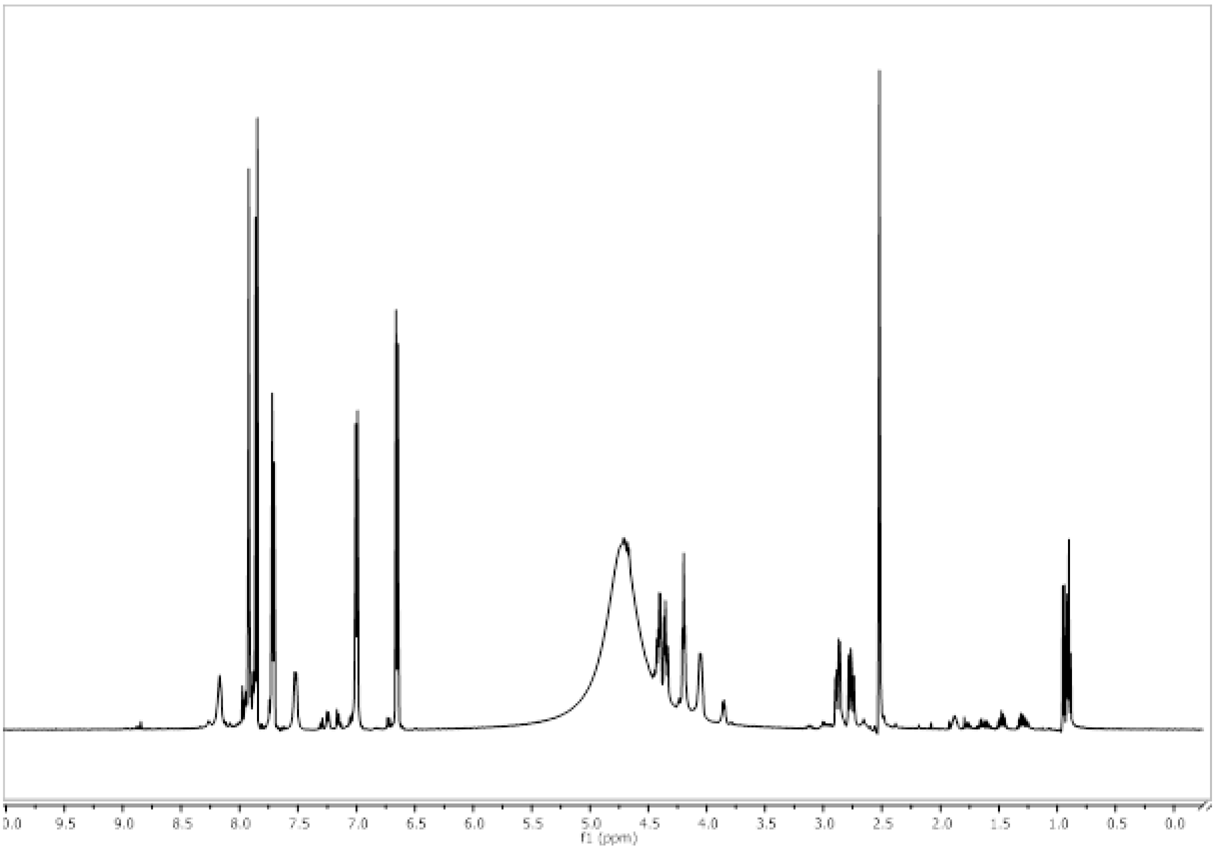

Figure S318: ^{1}H-NMR of Smoc-L-Tyr-OH **28**.

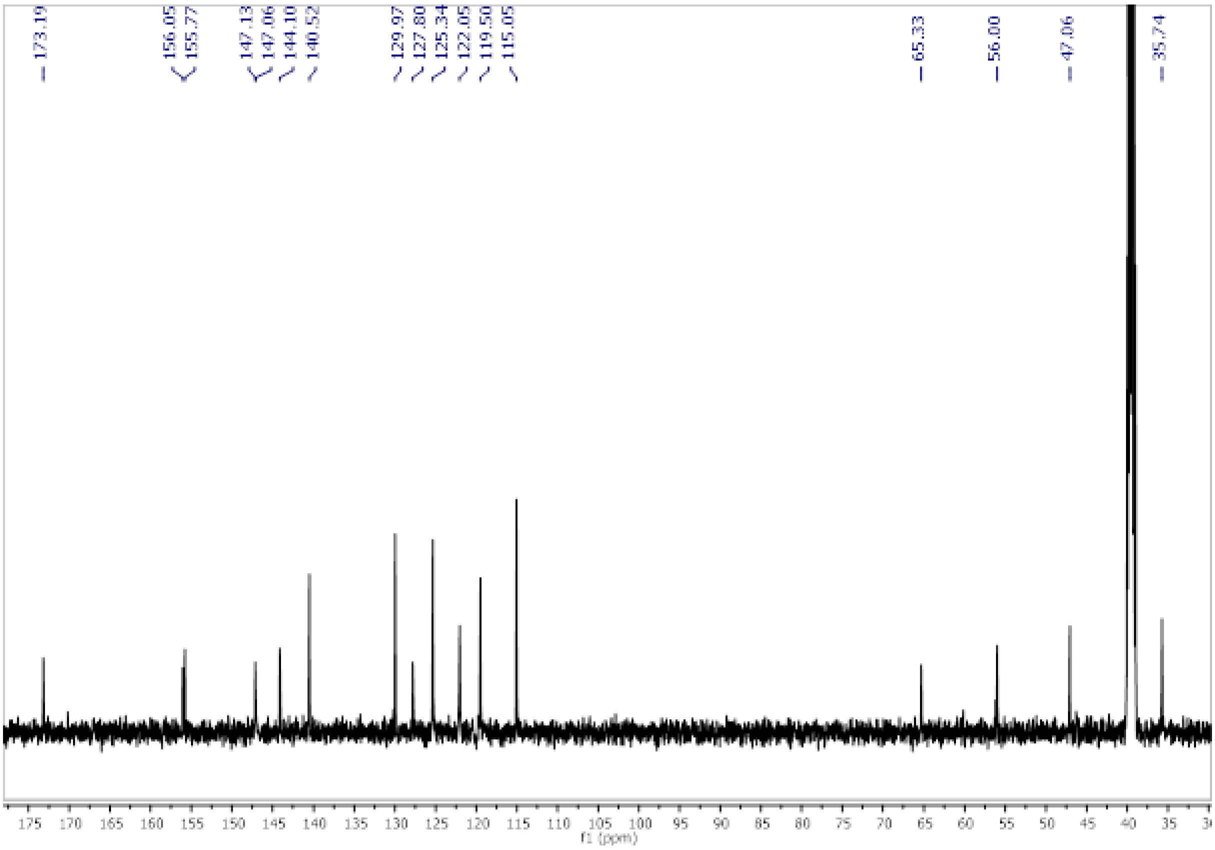

Figure S319: ^{13}C-NMR of Smoc-L-Tyr-OH **28**.

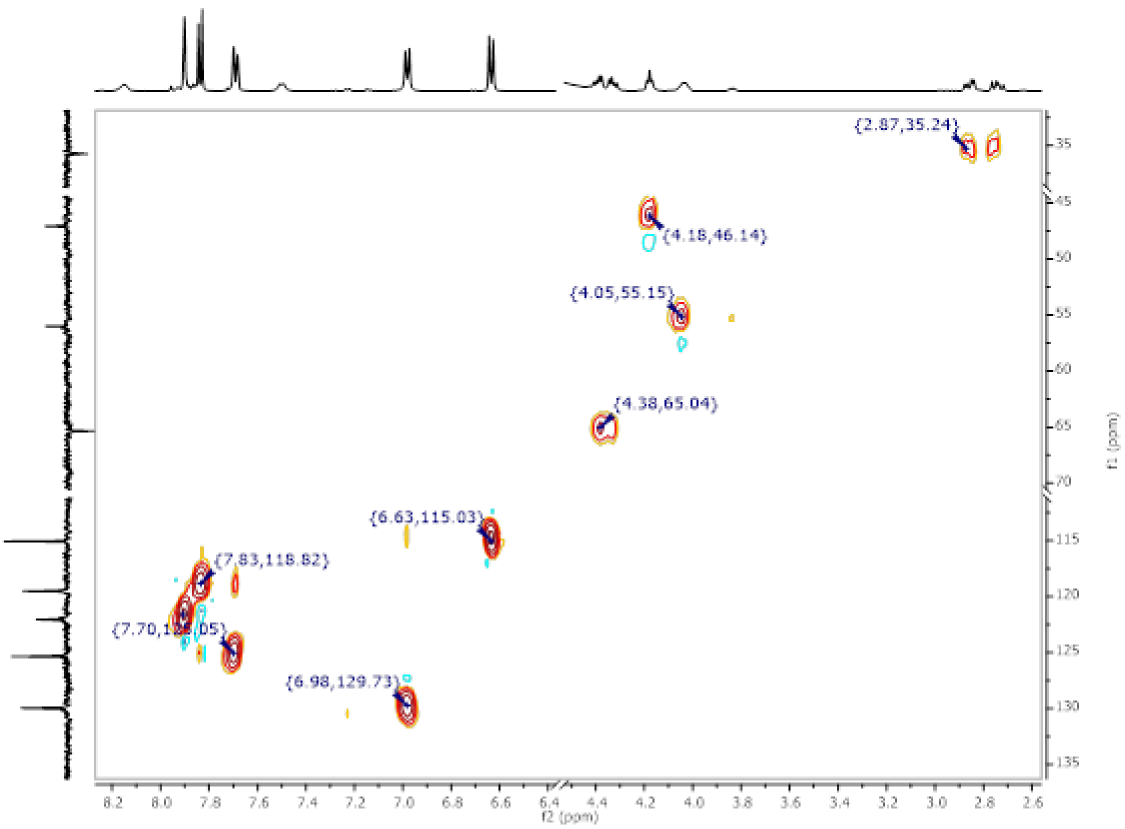

Figure S320: ^{1}H-^{13}C HSQC-NMR of Smoc-L-Tyr-OH **28**.

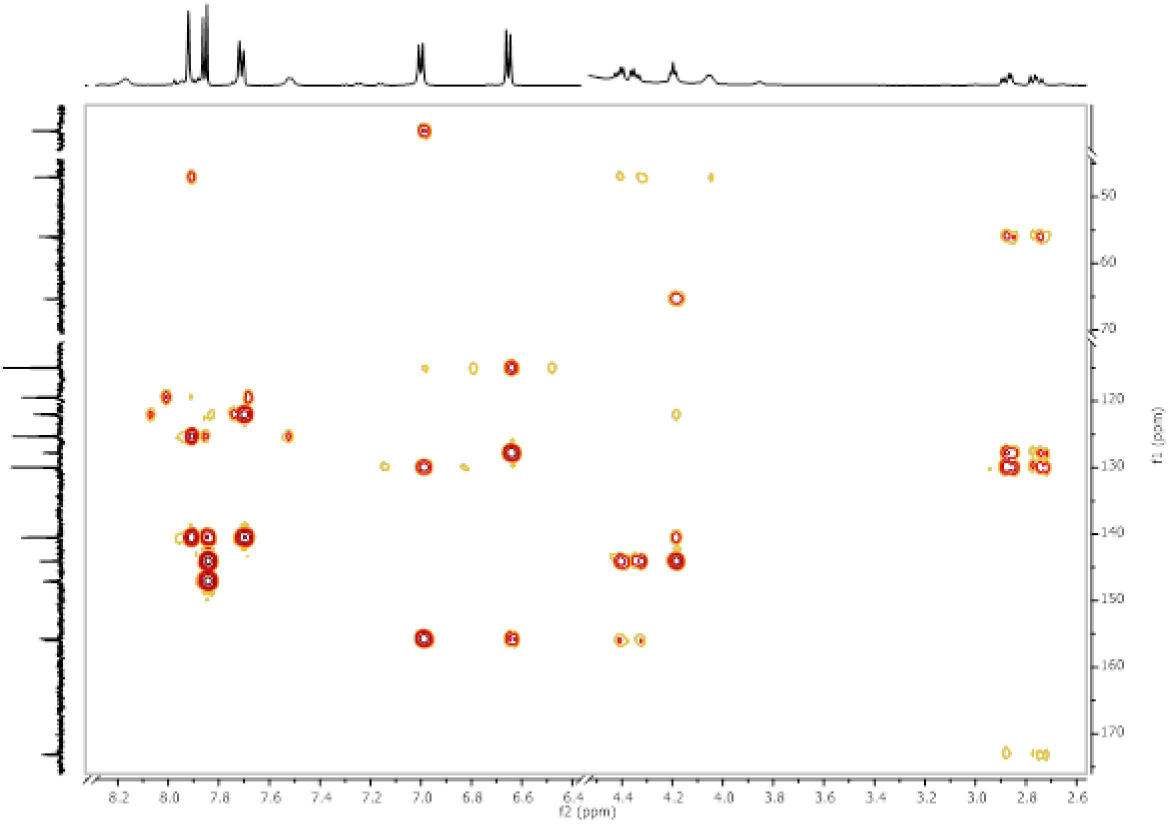

Figure S321: ^{1}H-^{13}C HMBC-NMR of Smoc-L-Tyr-OH **28**.

3.2.27. Analytical data of Smoc-L-Tyr(tBu)-OH 29

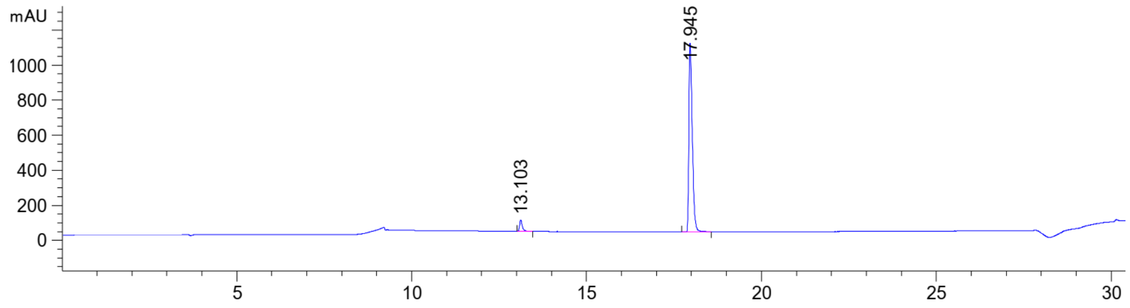

Figure S322: HPLC chromatogram of Smoc-L-Tyr(tBu)-OH **29** at λ=220 nm (0to60 MeCN).

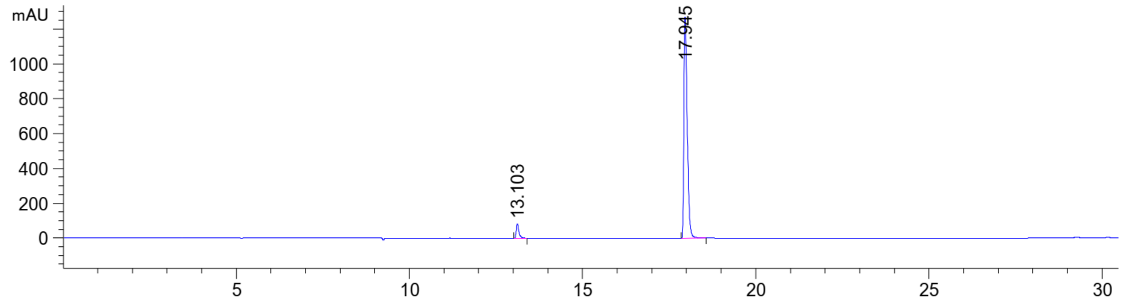

Figure S323: HPLC chromatogram of Smoc-L-Tyr(tBu)-OH **29** at λ=280 nm (0to60 MeCN).

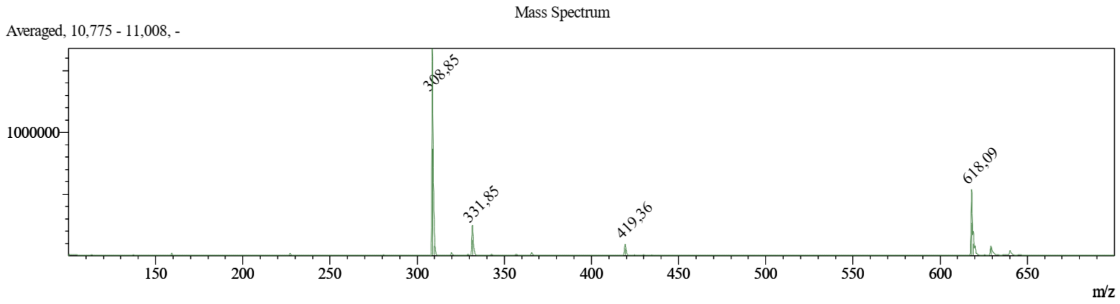

Figure S324: ESI-MS of Smoc-L-Tyr(tBu)-OH **29** (M measured=618.09 [M-H]$^{-}$, M calc.=619.66).

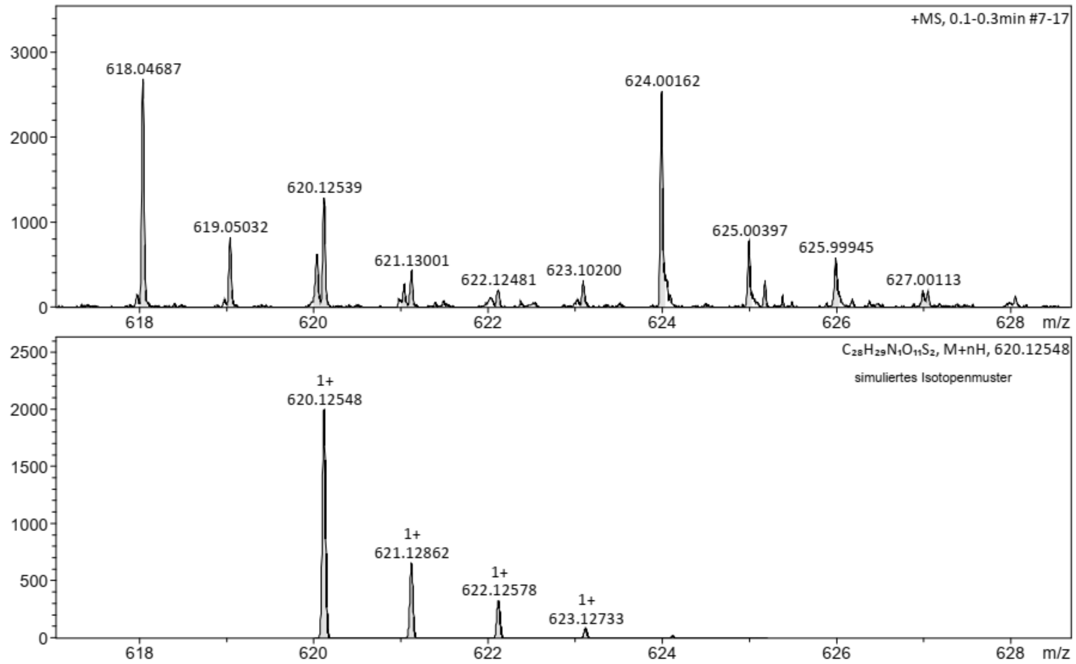

Figure S325: HR-MS of Smoc-L-Tyr(tBu)-OH **29** (M measured=620.12539 [M+H]$^{+}$, M calc.=620.12548).

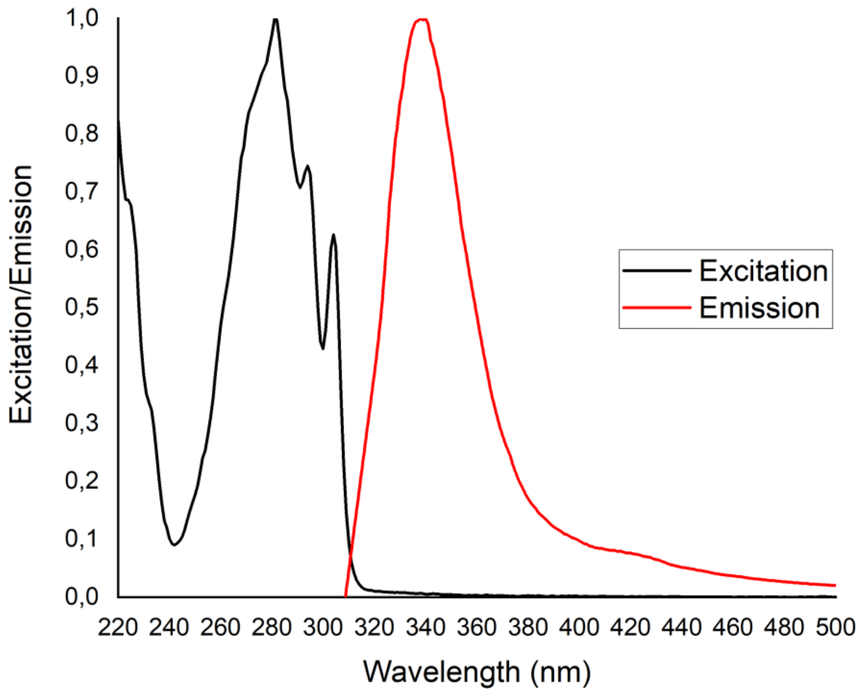

Figure S326: Excitation and emission spectra of Smoc-L-Tyr(tBu)-OH **29**, excitation and emission have been normalized between 0 and 1 for illustration.

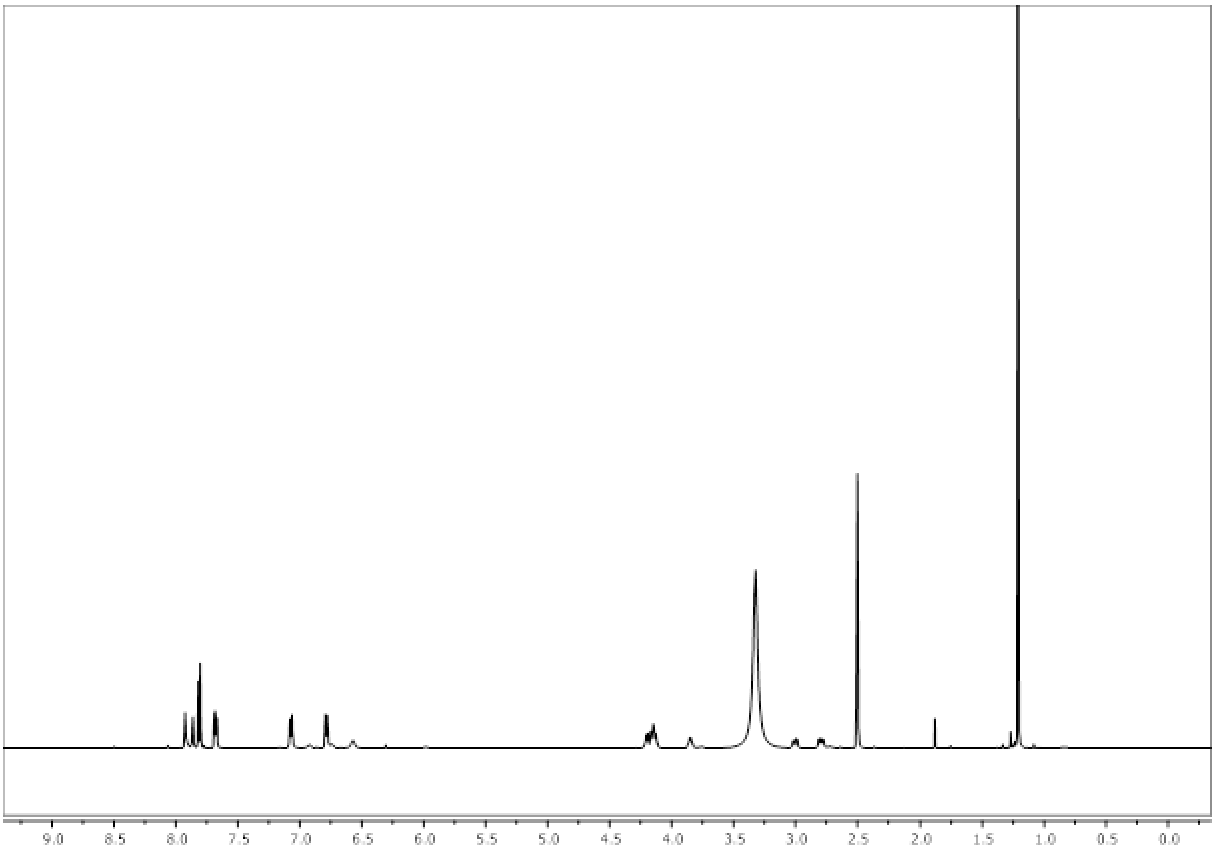

Figure S327: ^{1}H-NMR of Smoc-L-Tyr(tBu)-OH **29**.

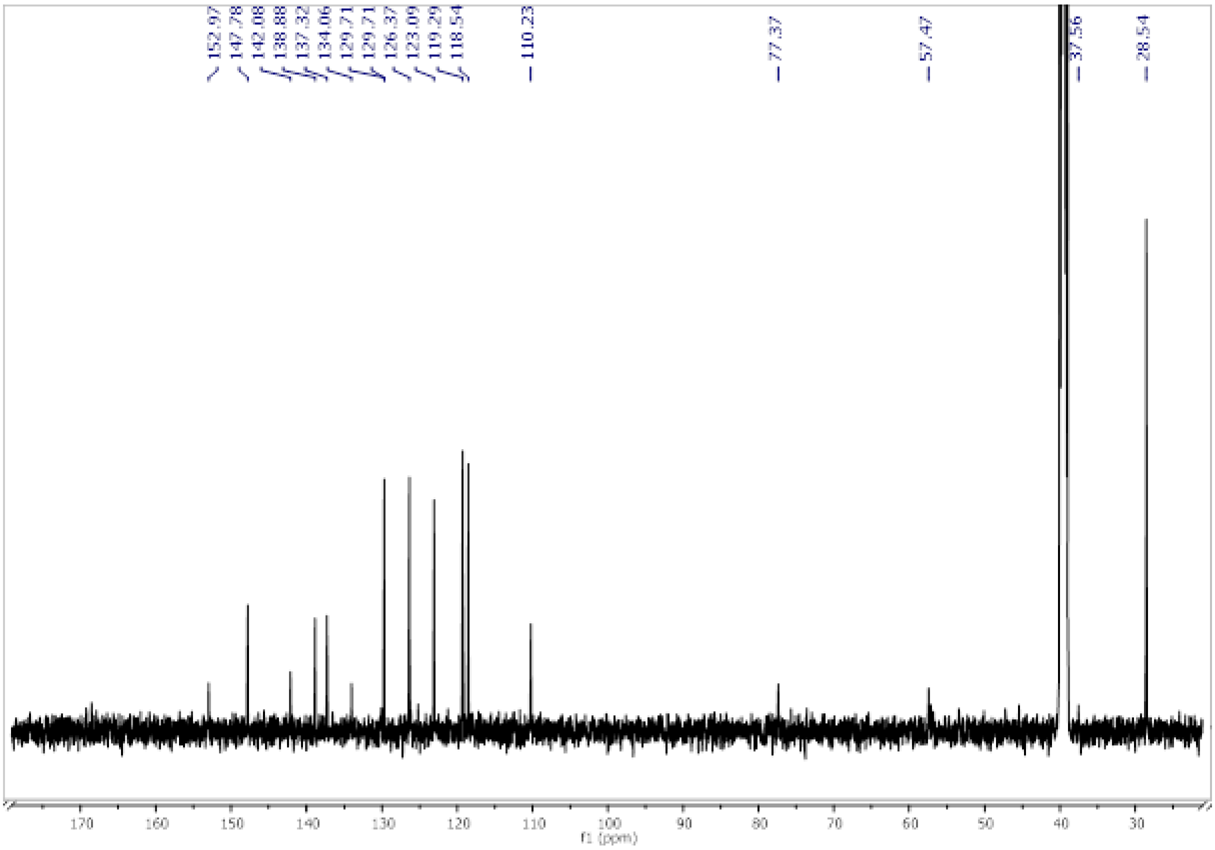

Figure S328: ^{13}C-NMR of Smoc-L-Tyr(tBu)-OH **29**.

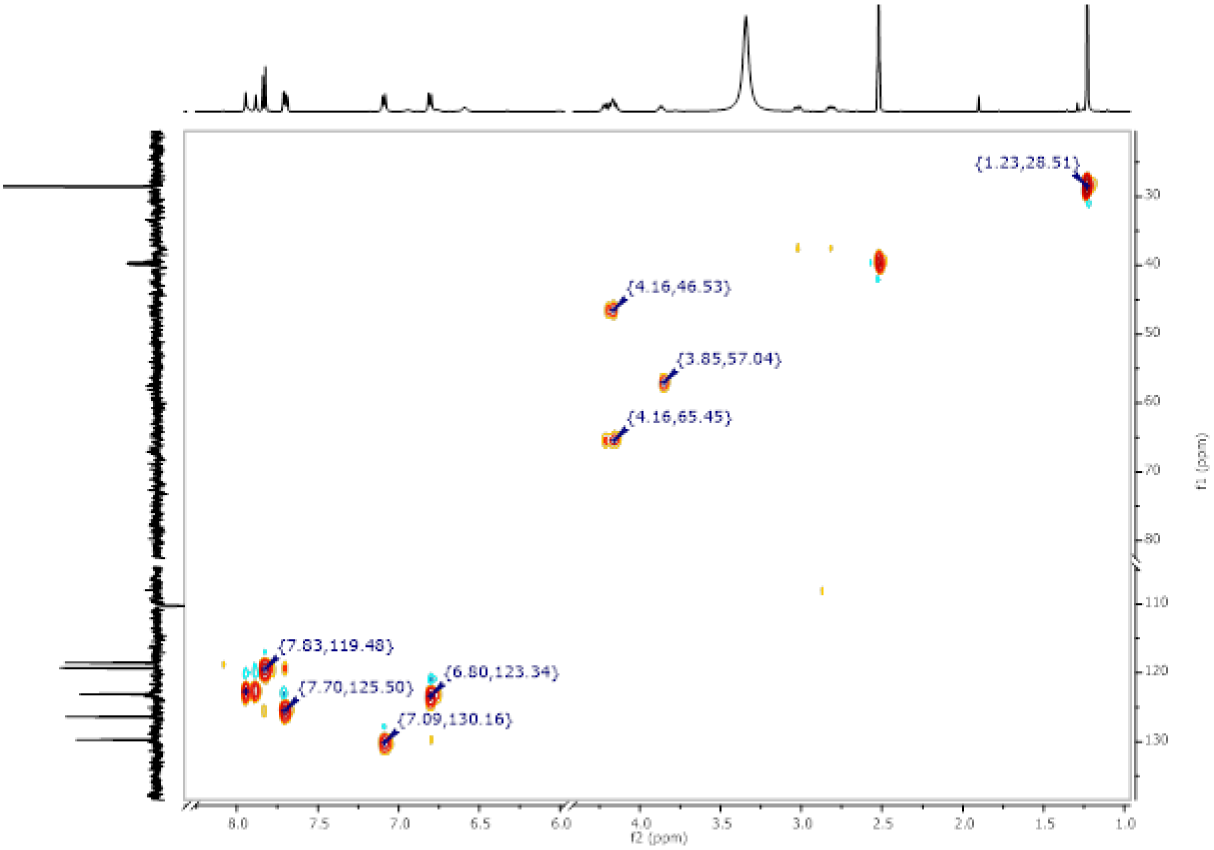

Figure S329: ^{1}H-^{13}C HSQC-NMR of Smoc-L-Tyr(tBu)-OH **29**.

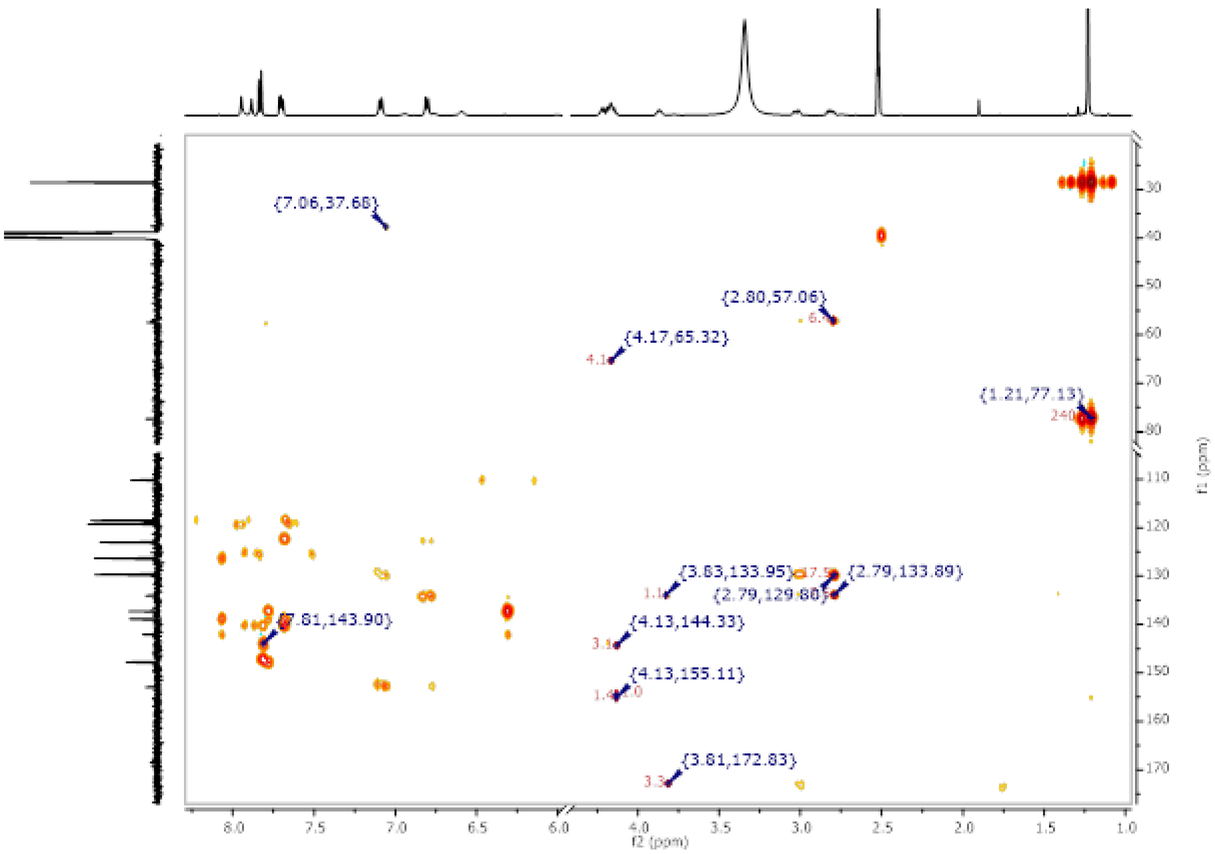

Figure S330: ^{1}H-^{13}C HMBC-NMR of Smoc-L-Tyr(tBu)-OH **29**.

3.2.28. Analytical data of Smoc-L-Val-OH 30

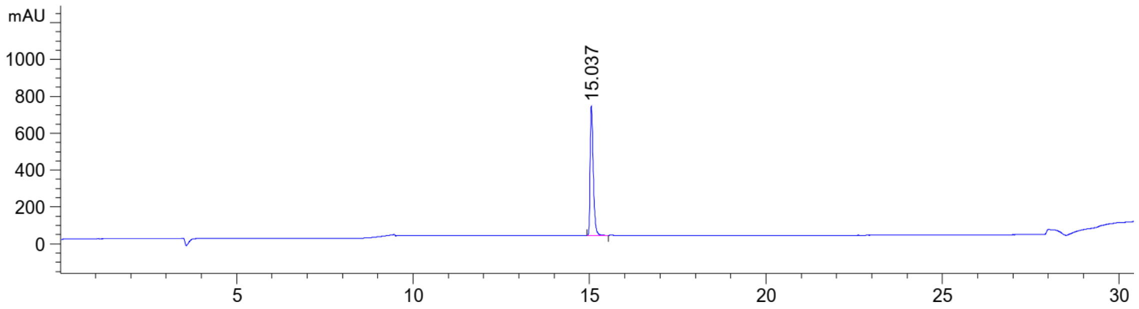

Figure S331: HPLC chromatogram of Smoc-L-Val-OH **30** at λ=220 nm (0to40 MeCN).

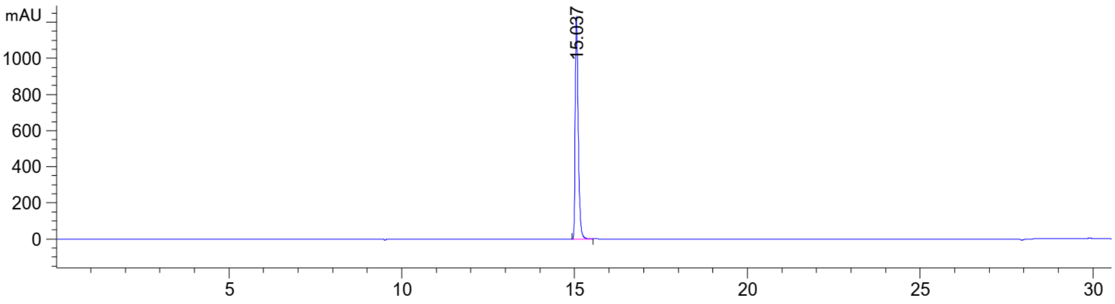

Figure S332: HPLC chromatogram of Smoc-L-Val-OH **30** at λ=280 nm (0to40 MeCN).

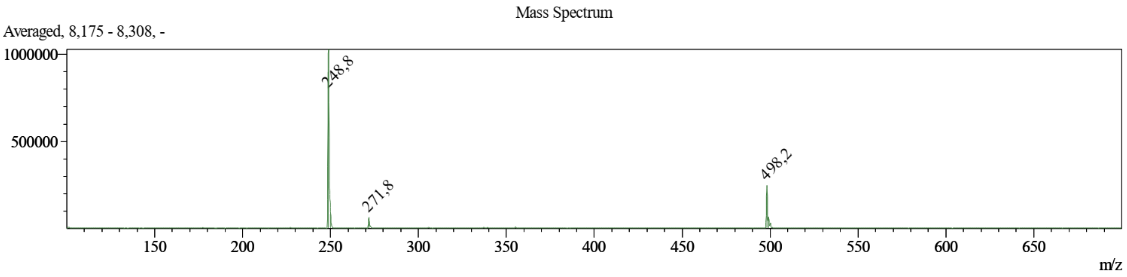

Figure S333: ESI-MS of Smoc-L-Val-OH **30** (M measured=498.20 [M-H]$^-$, M calc.=499.51).

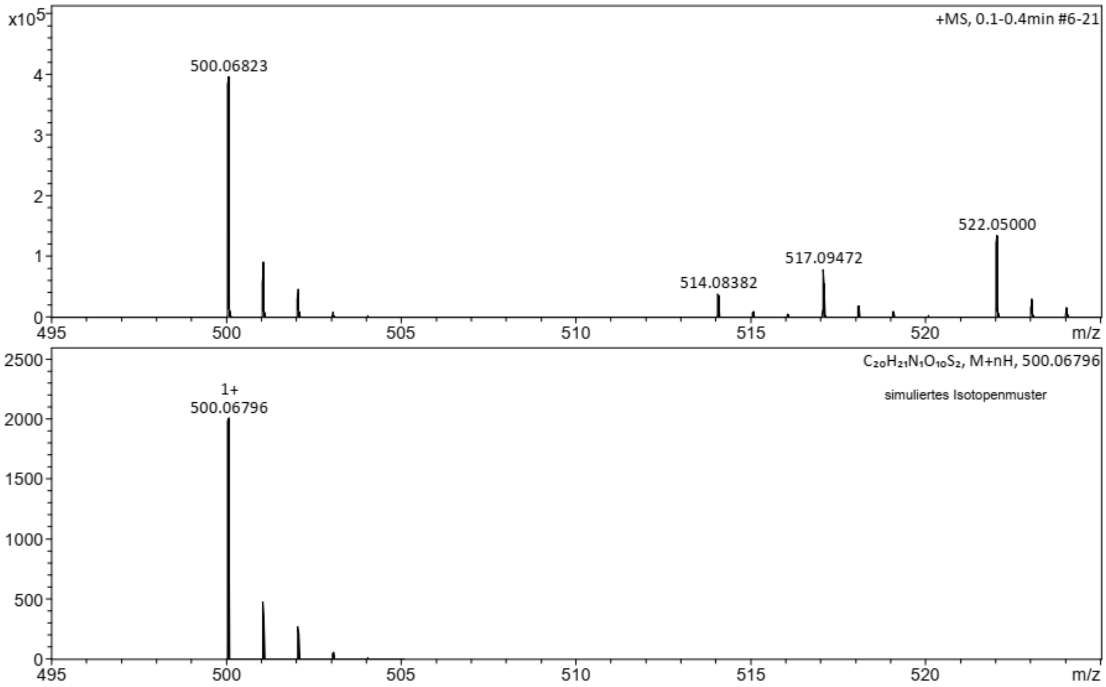

Figure S334: HR-MS of Smoc-L-Val-OH **30** (M measured=500.06823 [M+H]$^+$, M calc.=500.06796).

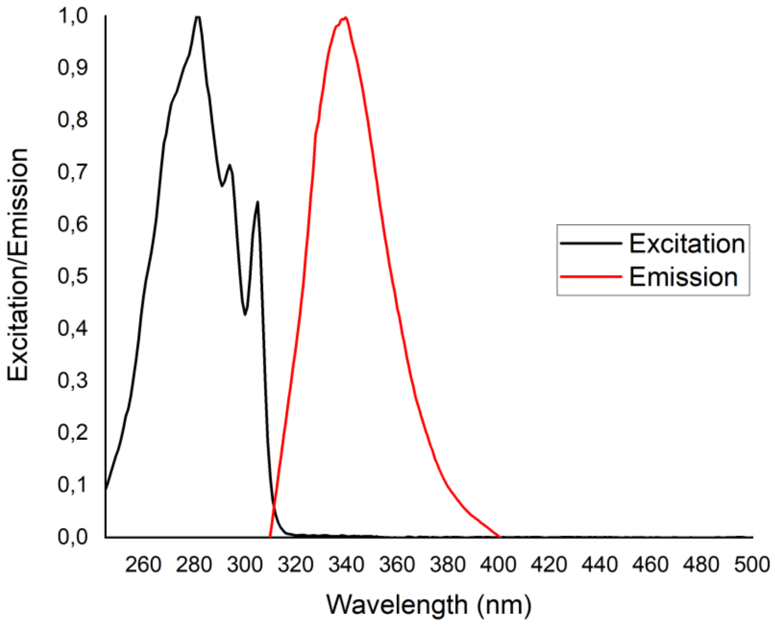

Figure S335: Excitation and emission spectra of Smoc-L-Val-OH **30**, excitation and emission have been normalized between 0 and 1 for illustration.

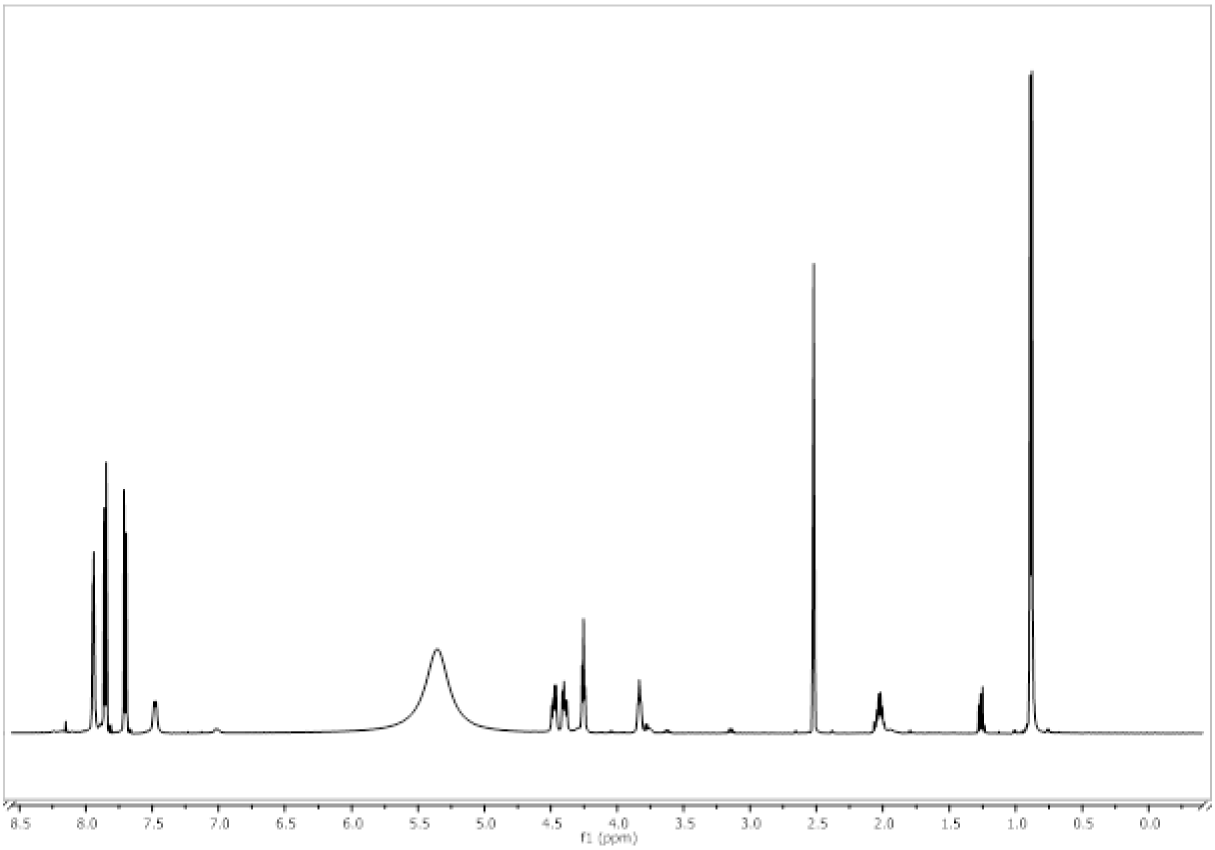

Figure S336: ^{1}H-NMR of Smoc-L-Val-OH **30**.

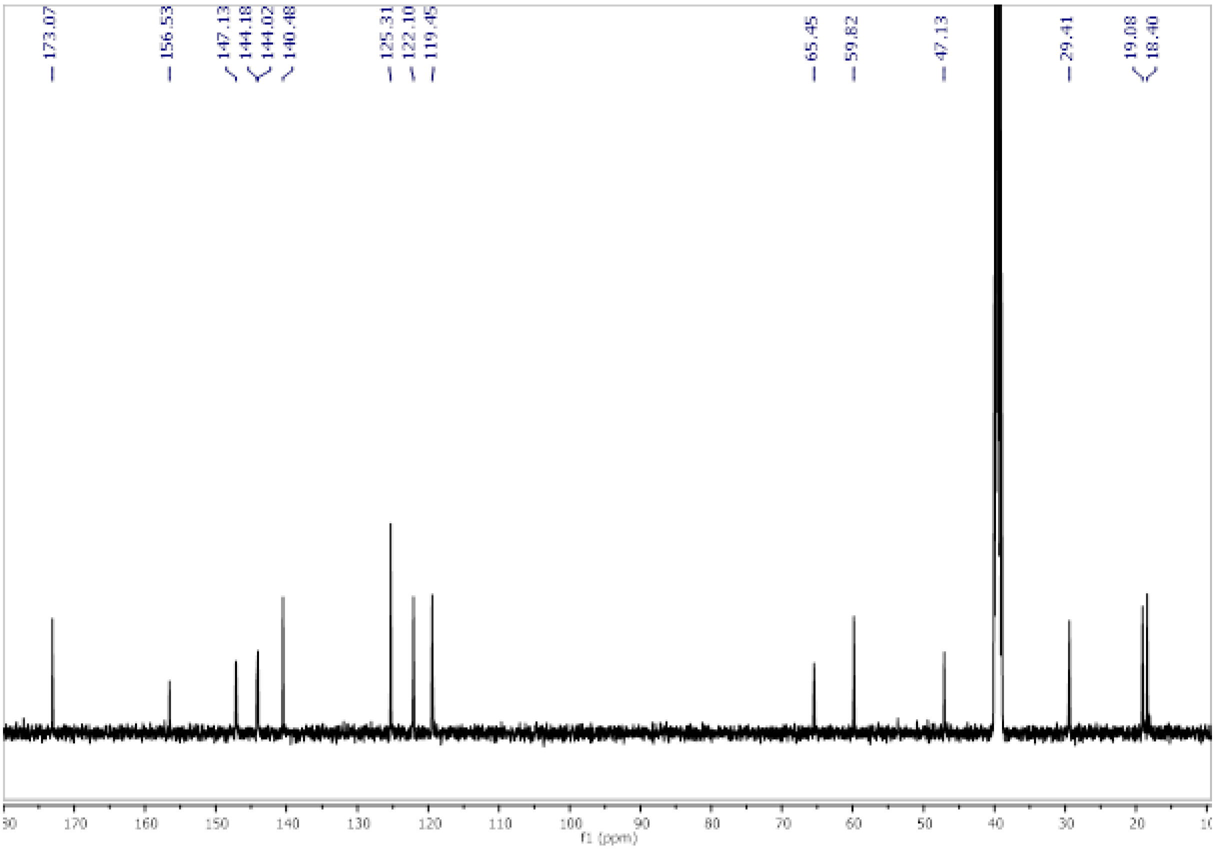

Figure S337: ^{13}C-NMR of Smoc-L-Val-OH **30**.

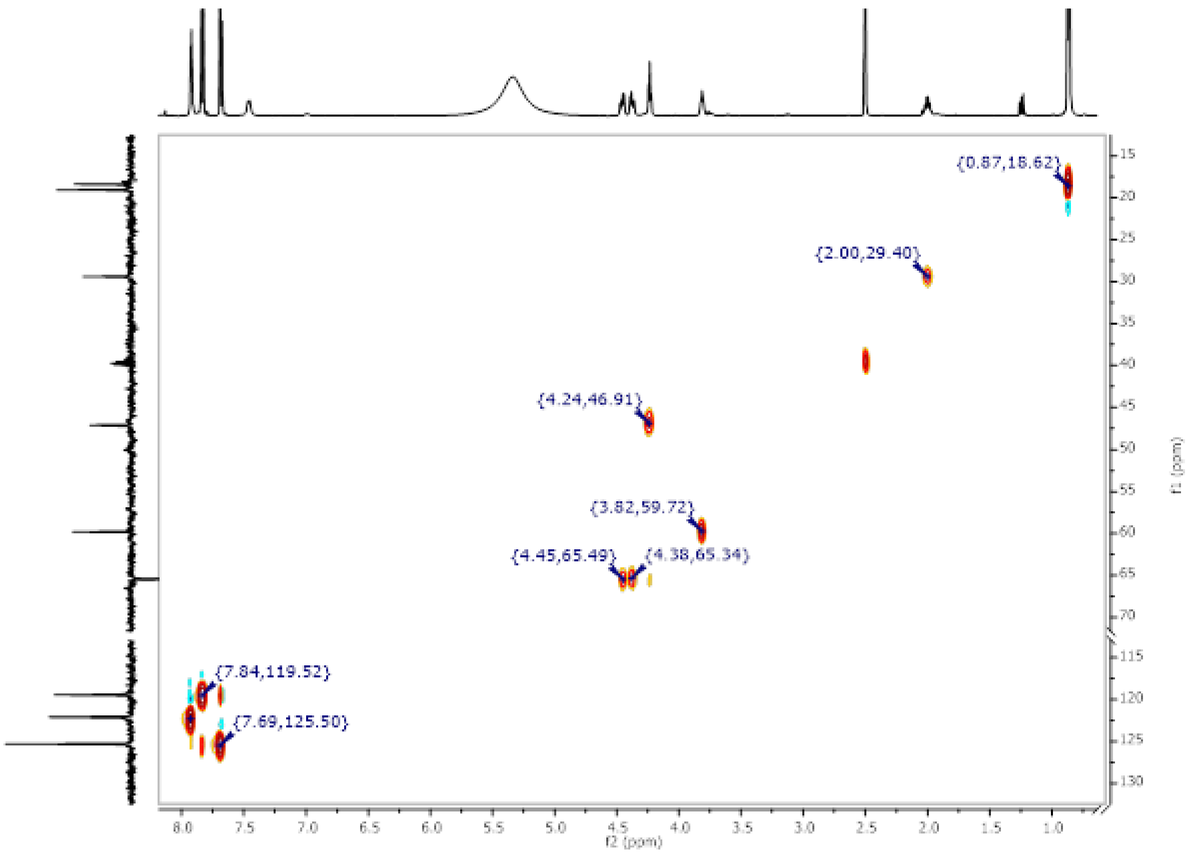

Figure S338: ^{1}H-^{13}C HSQC-NMR of Smoc-L-Val-OH **30**.

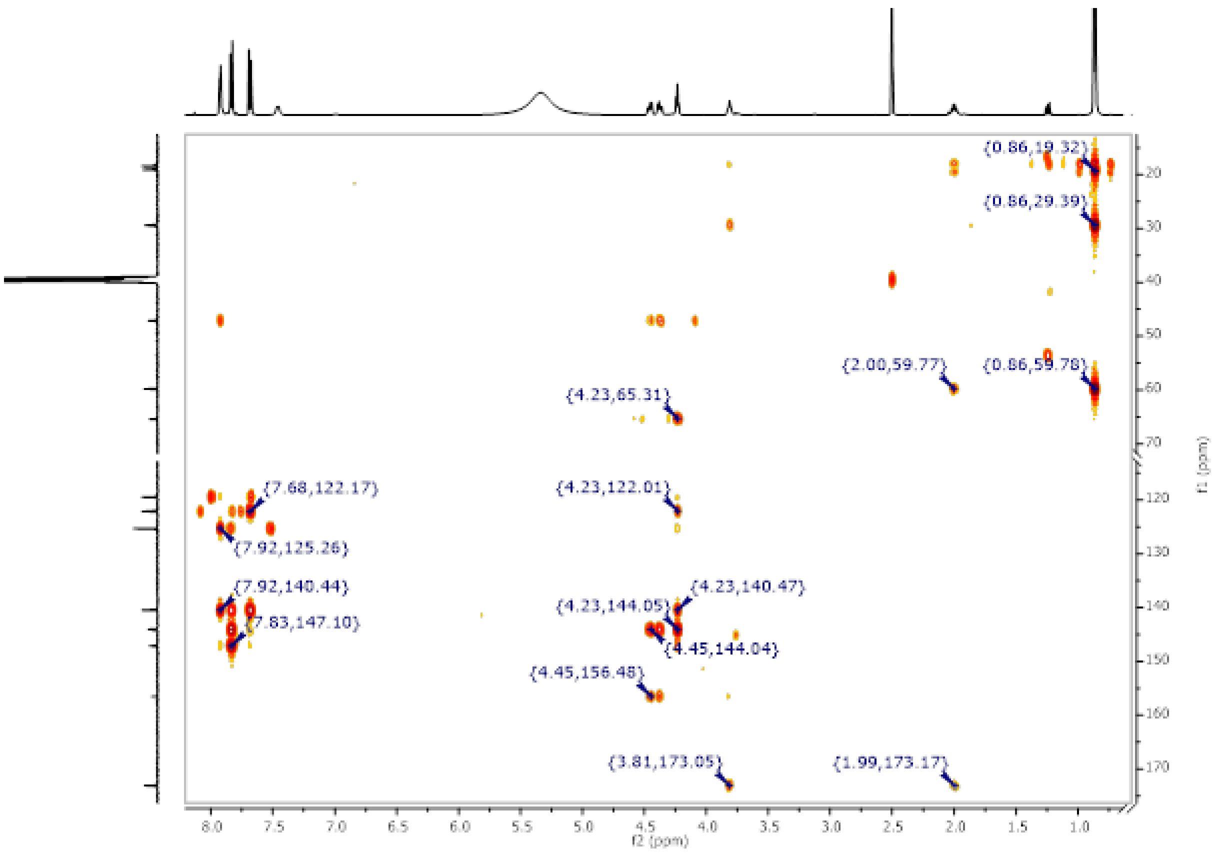

Figure S339: ^{1}H-^{13}C HMBC-NMR of Smoc-L-Val-OH **30**.

3.2.29. Analytical data of Smoc-β-Ala-OH 31

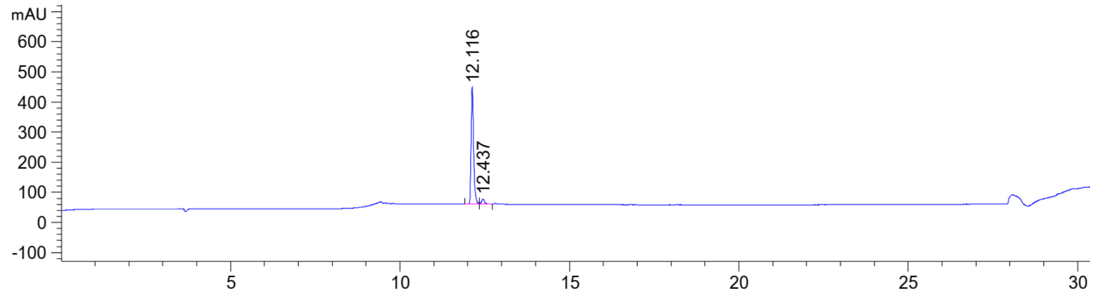

Figure S340: HPLC chromatogram of Smoc-β-Ala-OH **31** at λ=220 nm (0to40 MeCN).

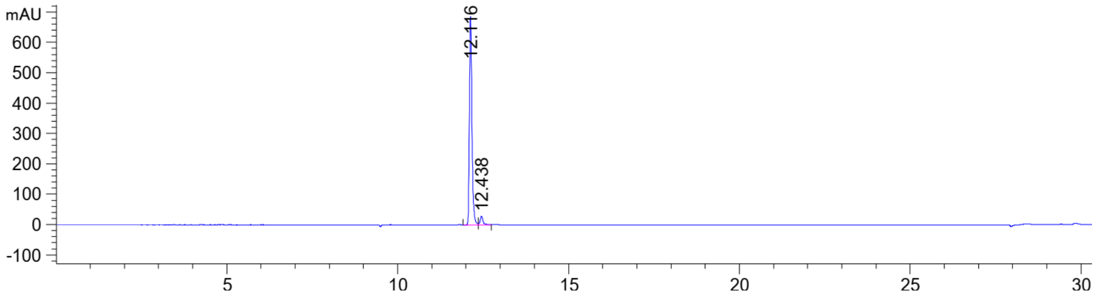

Figure S341: HPLC chromatogram of Smoc-β-Ala-OH **31** at λ=280 nm (0to40 MeCN).

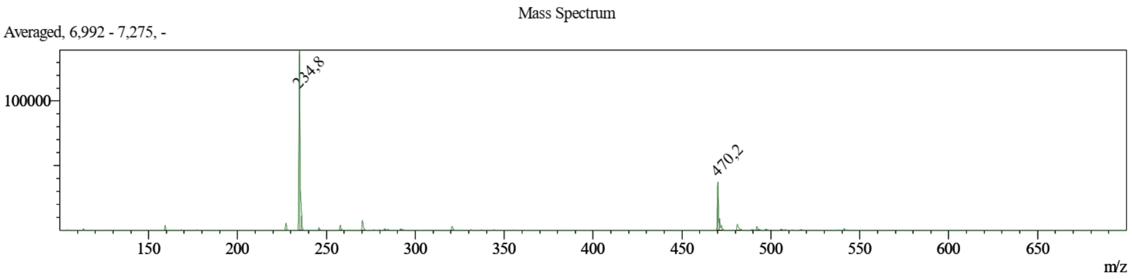

Figure S342: ESI-MS of Smoc-β-Ala-OH **31** (M measured=470.20 [M-H]$^-$, M calc.=471.45).

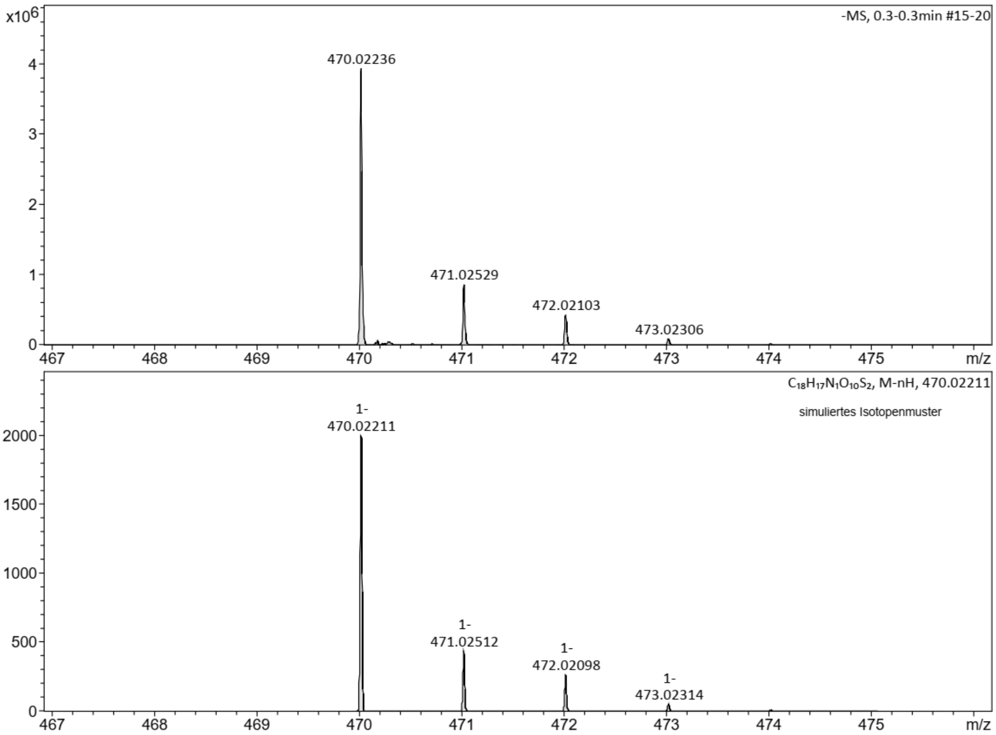

Figure S343: HR-MS of Smoc-β-Ala-OH **31** (M measured=470.02236 [M-H]$^-$, M calc.=470.02211).

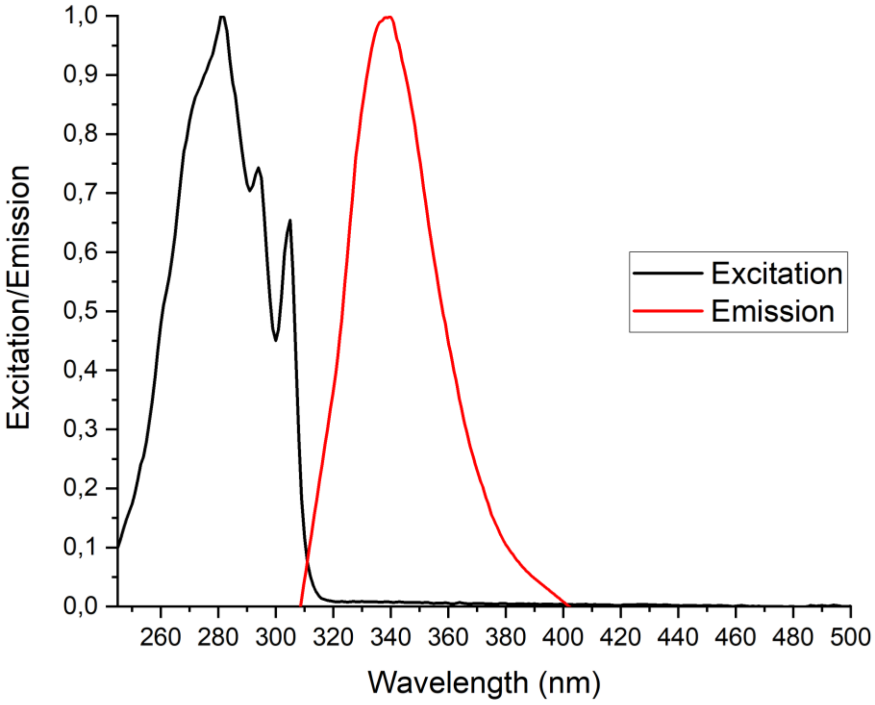

Figure S344: Excitation and emission spectra of Smoc-β-Ala-OH **31**, excitation and emission have been normalized between 0 and 1 for illustration.

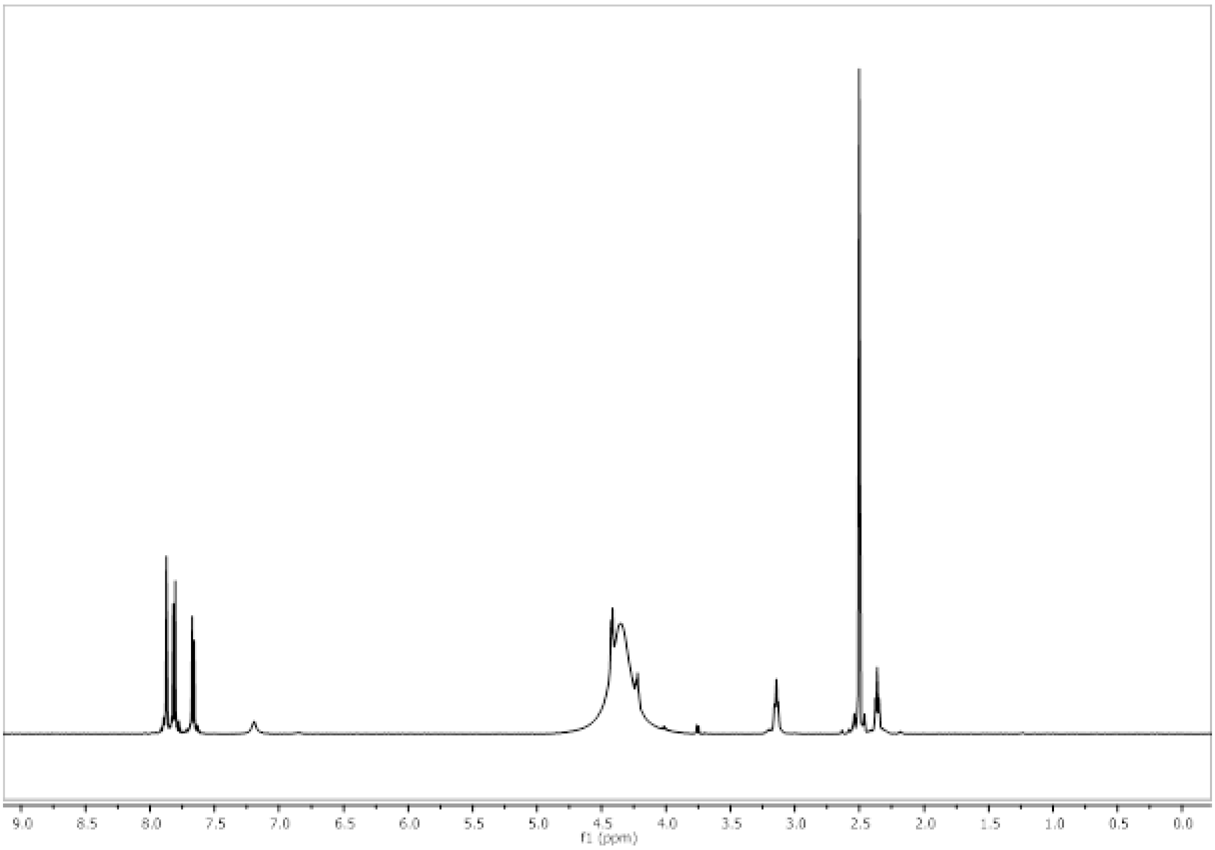

Figure S345: ^{1}H-NMR of Smoc-β-Ala-OH **31**.

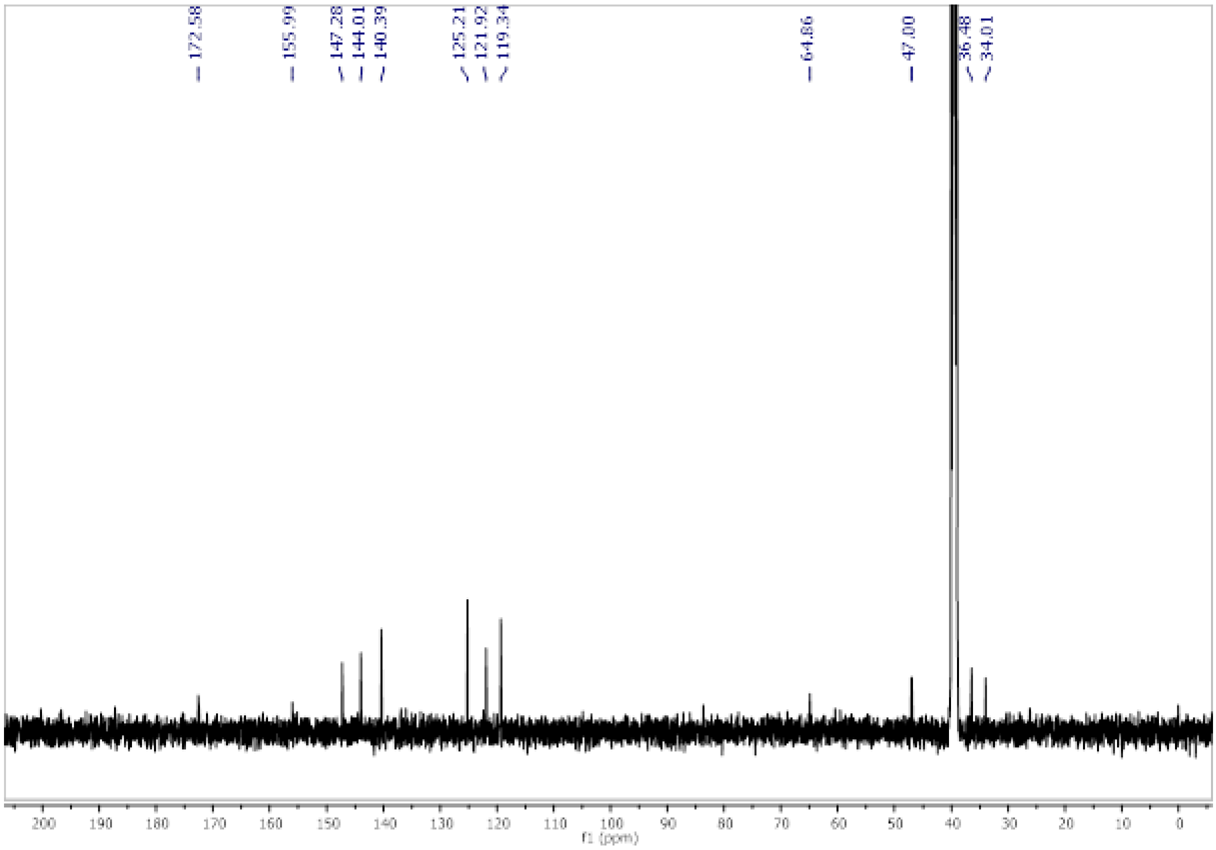

Figure S346: ^{13}C-NMR of Smoc-β-Ala-OH **31**.

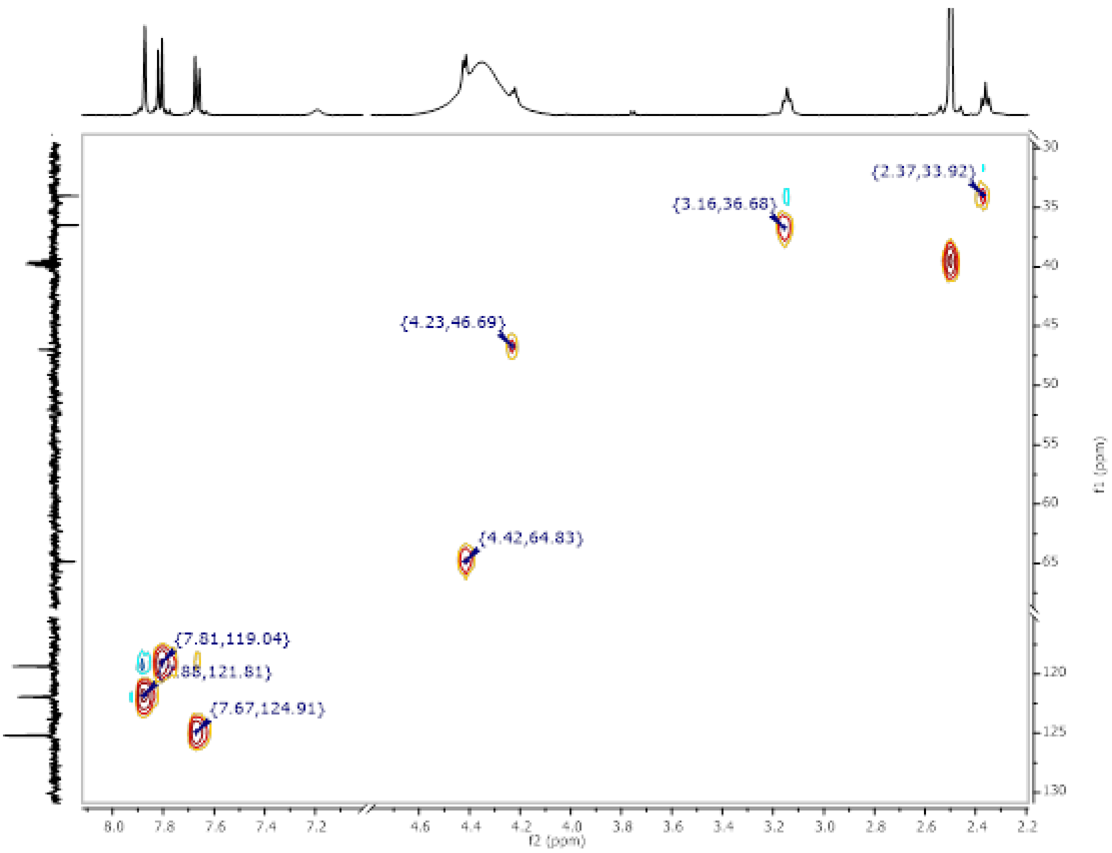

Figure S347: ^{1}H-^{13}C HSQC-NMR of Smoc-β-Ala-OH **31**.

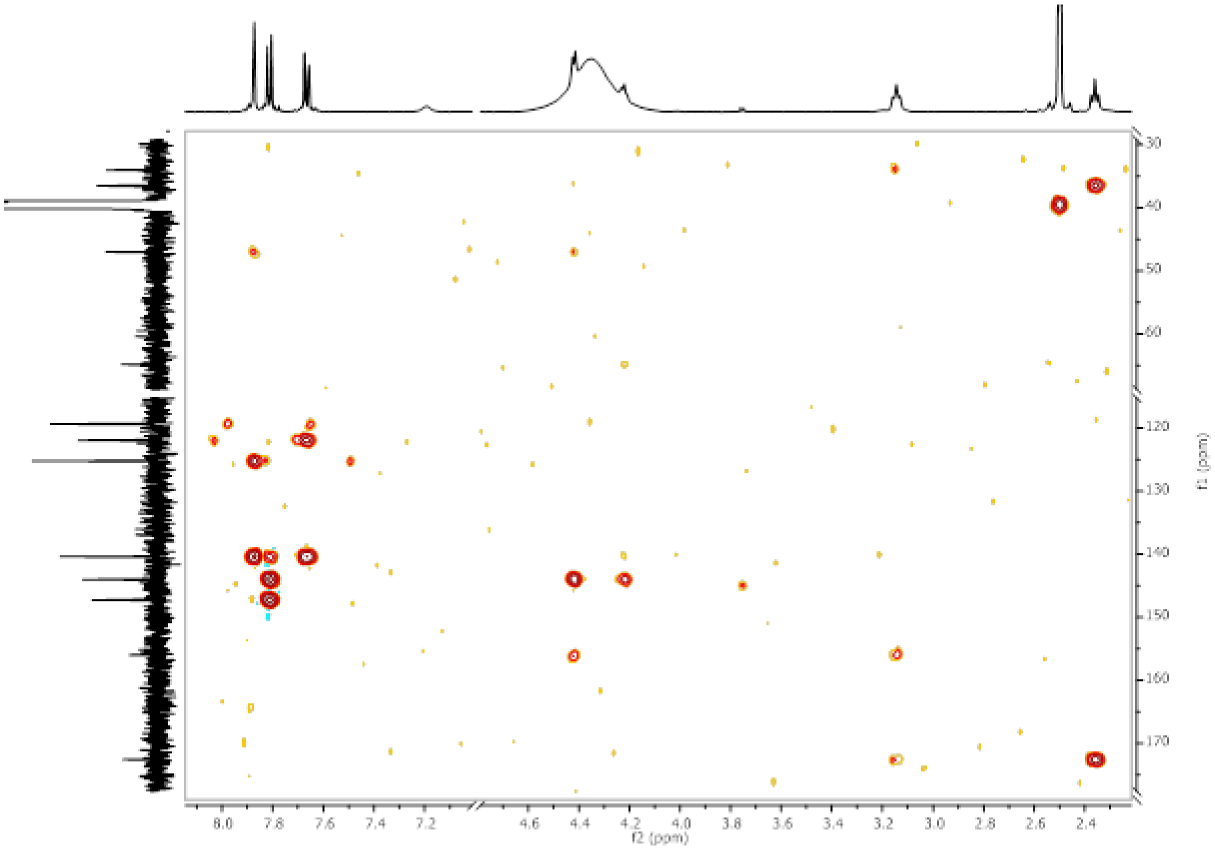

Figure S348: ^1H-^{13}C HMBC-NMR of Smoc-β-Ala-OH **31**.

3.2.30. Analytical data of Smoc-Aib-OH 32

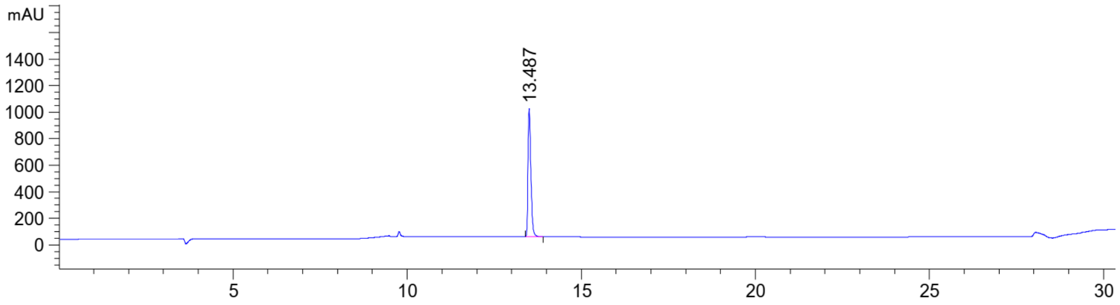

Figure S349: HPLC chromatogram of Smoc-Aib-OH **32** at λ=220 nm (0to40 MeCN).

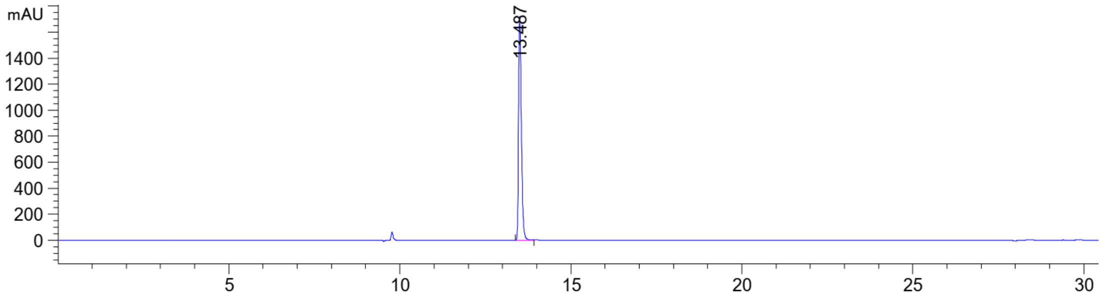

Figure S350: HPLC chromatogram of Smoc-Aib-OH **32** at λ=280 nm (0to40 MeCN).

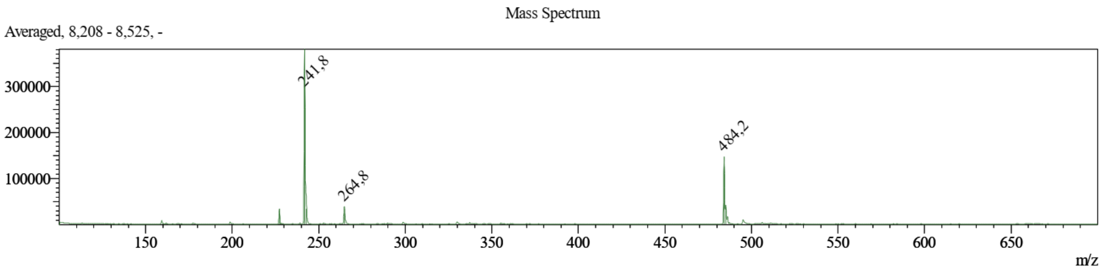

Figure S351: ESI-MS of Smoc-Aib-OH **32** (M measured=484.20 $[M-H]^-$, M calc.=485.48).

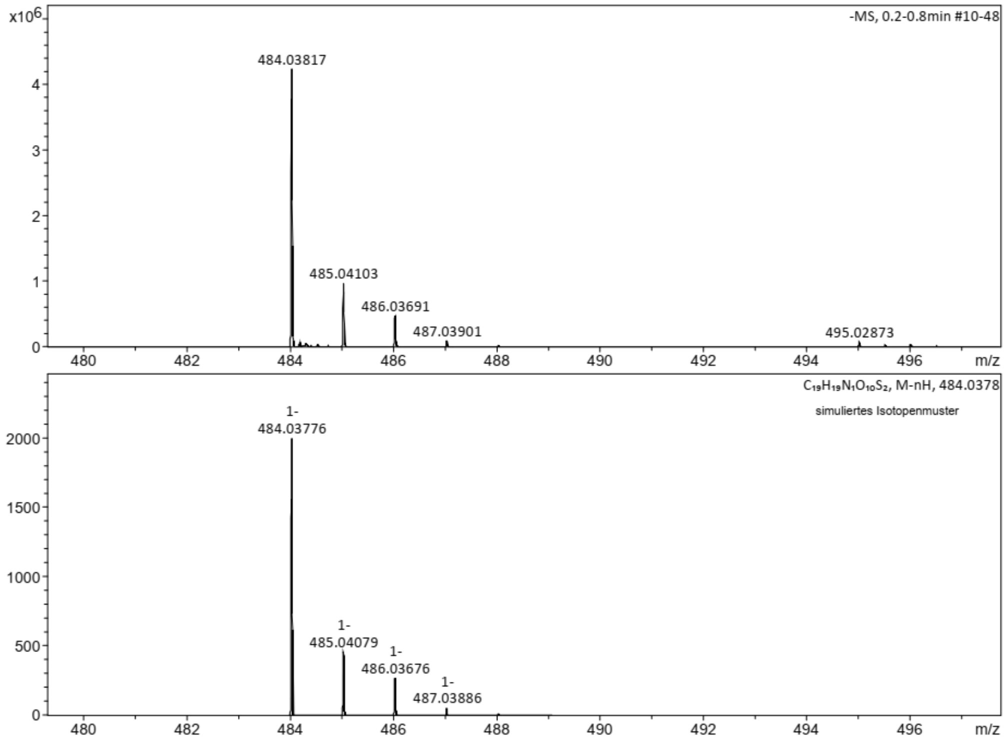

Figure S352: HR-MS of Smoc-Aib-OH **32** (M measured=484.03817 $[M-H]^-$, M calc.=484.03776).

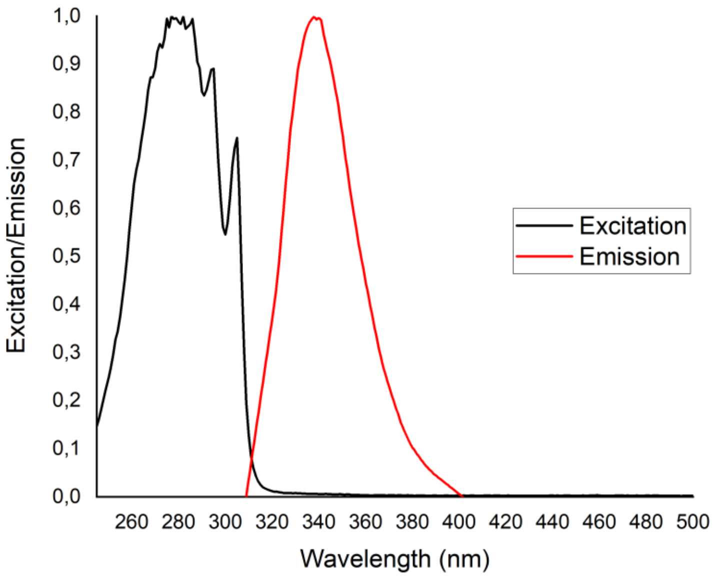

Figure S353: Excitation and emission spectra of Smoc-Aib-OH **32**, excitation and emission have been normalized between 0and 1 for illustration.

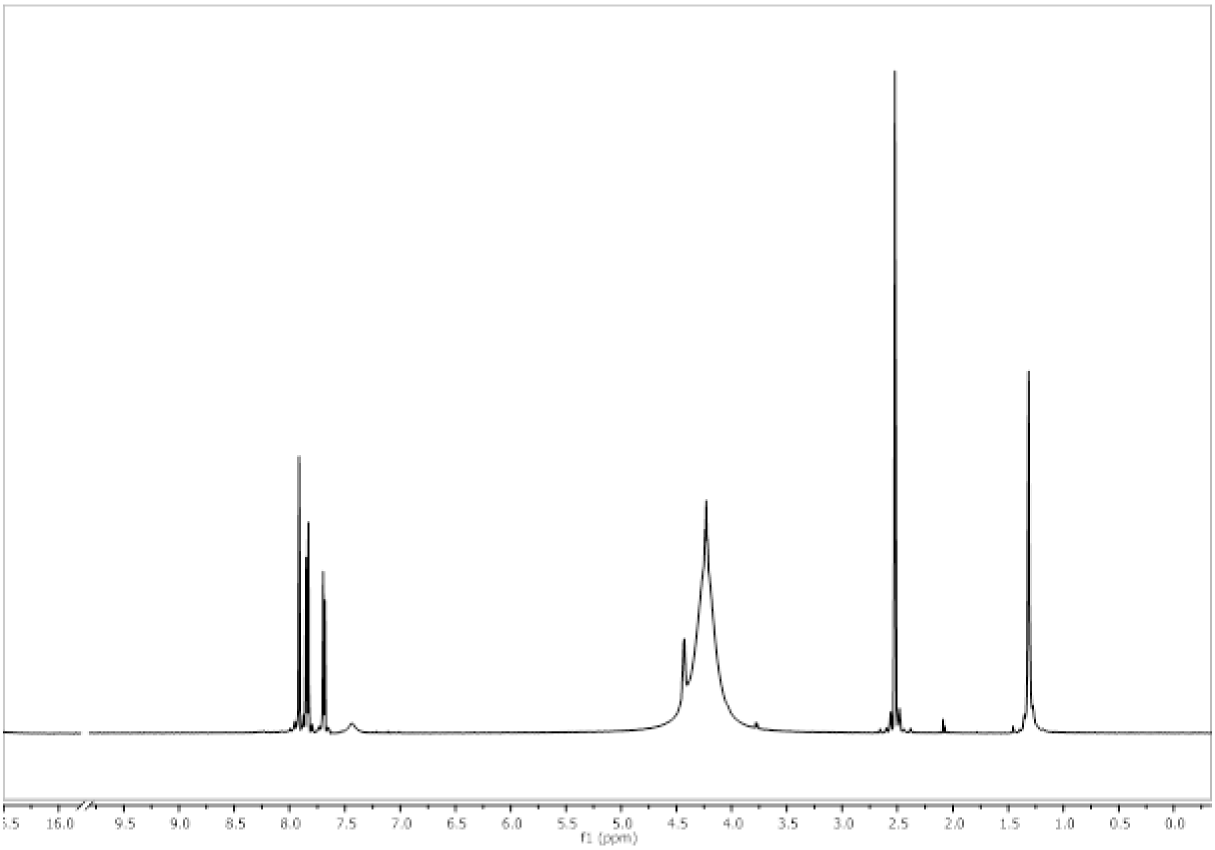

Figure S354: ^{1}H-NMR of Smoc-Aib-OH **32**.

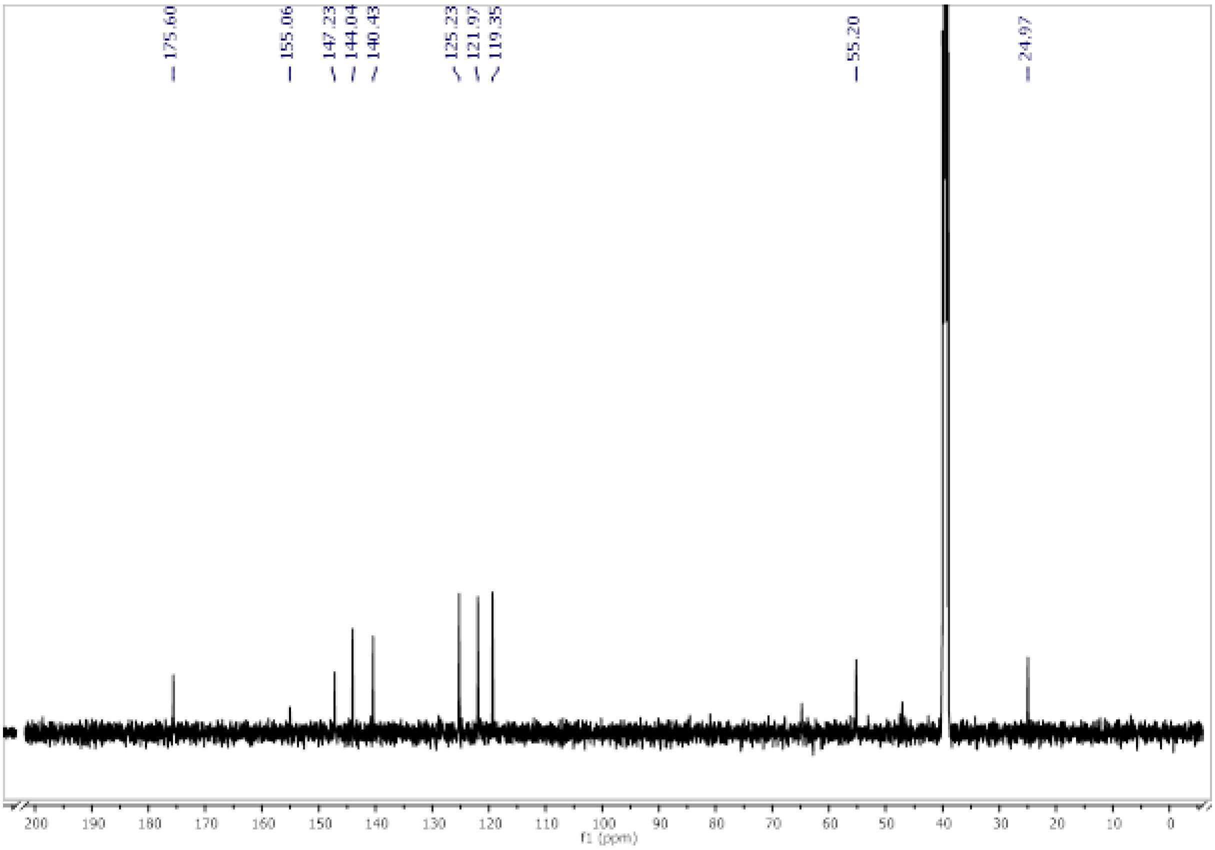

Figure S355: ^{13}C-NMR of Smoc-Aib-OH **32**.

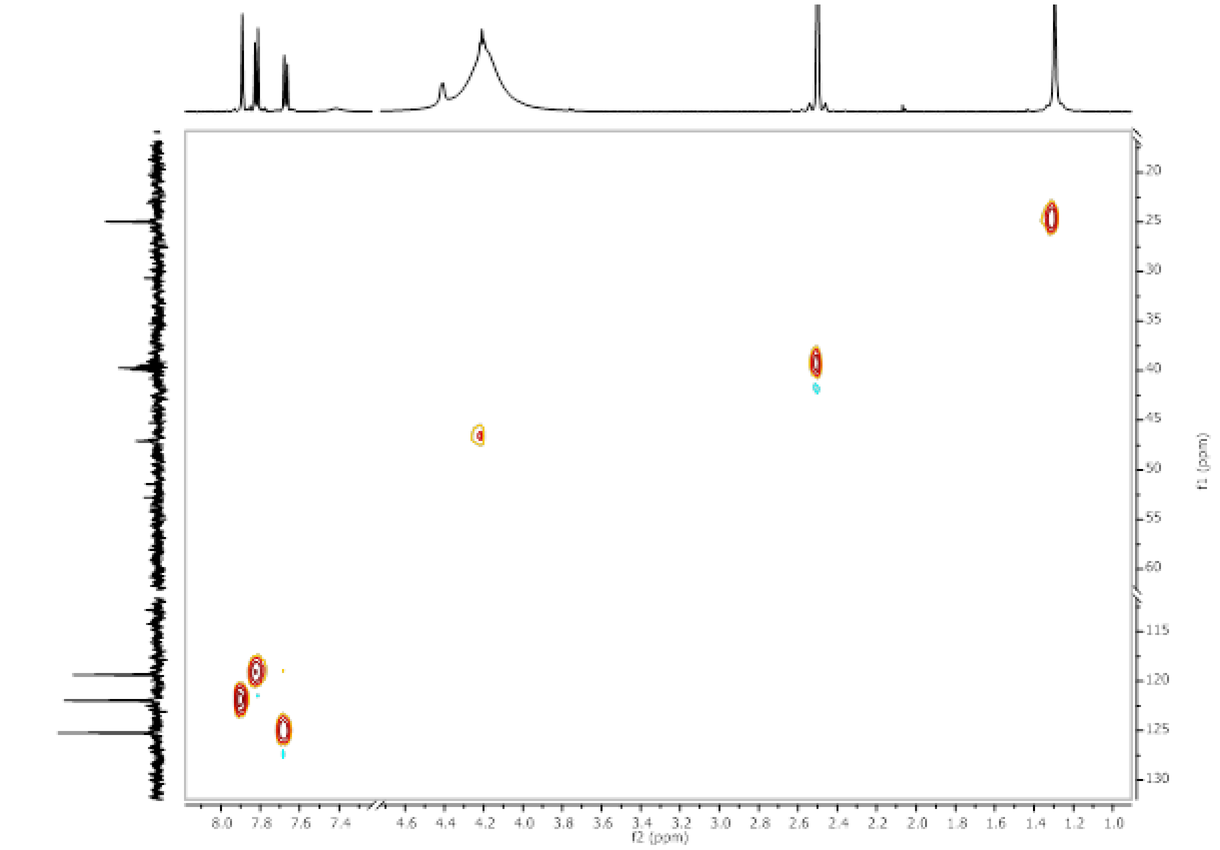

Figure S356: ^{1}H-^{13}C HSQC-NMR of Smoc-Aib-OH **32**.

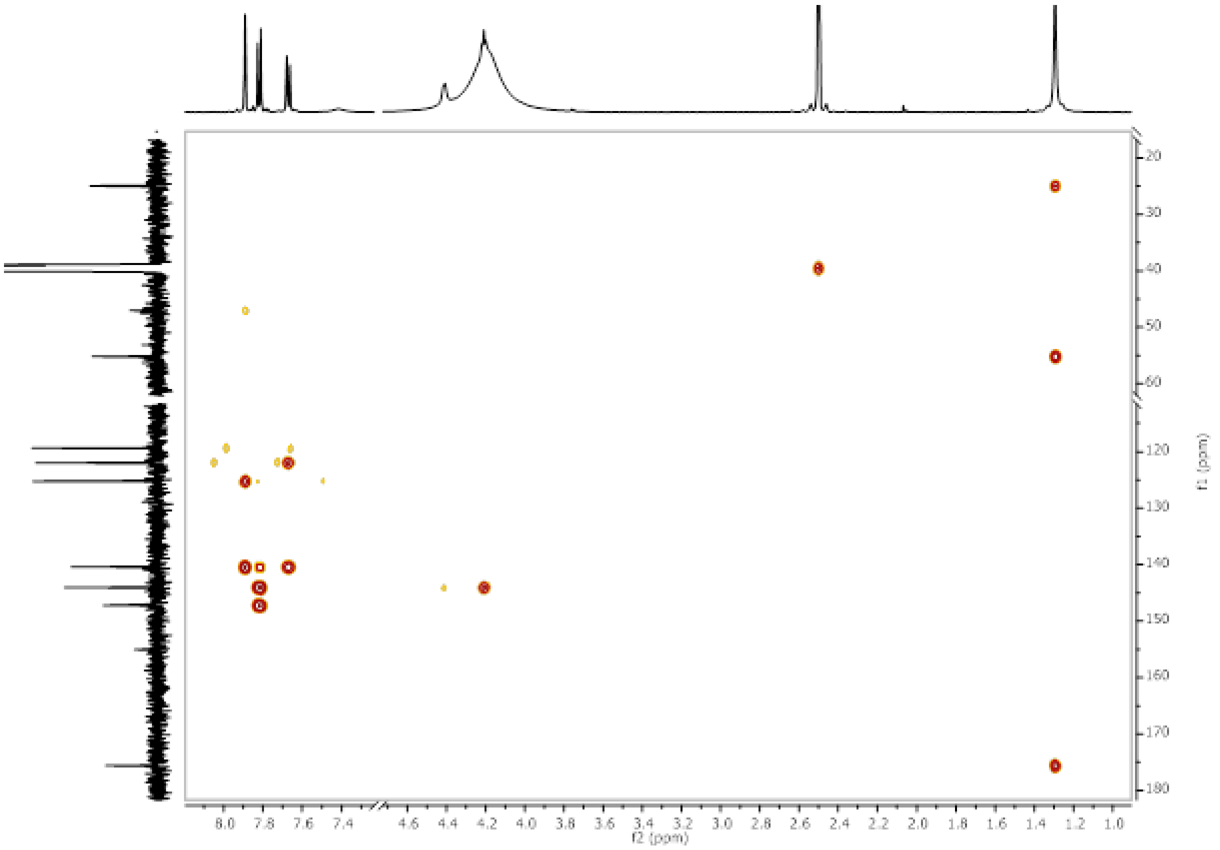

Figure S357: ^{1}H-^{13}C HMBC-NMR of Smoc-Aib-OH **32**.

3.3. Analytical data of deprotection studies

3.3.1. Analytical data of Smoc-Arg-OH 5 deprotection

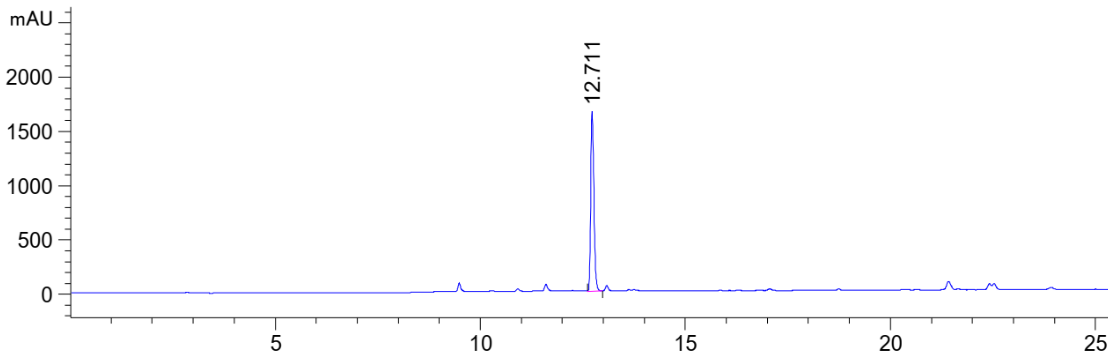

Figure S358: HPLC chromatogram of Smoc-L-Arg-OH **5** Ref for deprotection at λ=220 nm (0to40 MeCN).

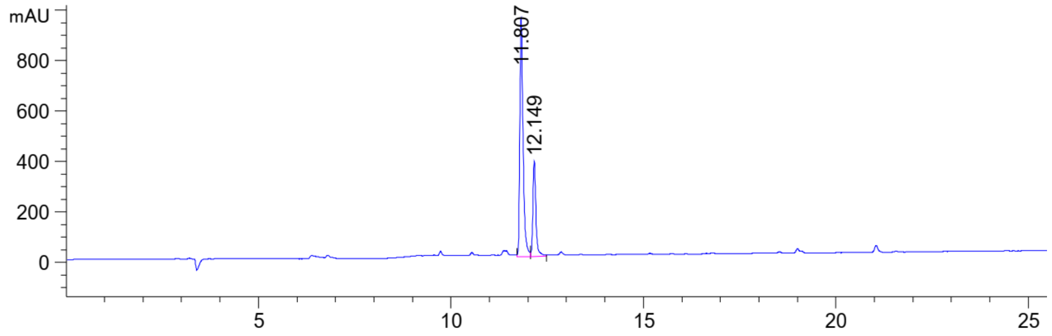

Figure S359: HPLC chromatogram of Smoc-L-Arg-OH **5** deprotection with 20% piperidine in water after 5min at λ=220 nm (0to40 MeCN).

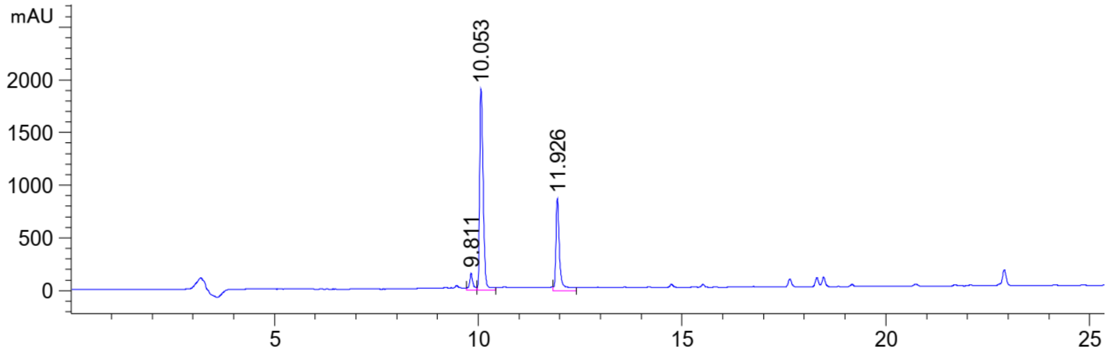

Figure S360: HPLC chromatogram of Smoc-L-Arg-OH **5** deprotection with 10% ethanolamine in water after 5min at λ=220 nm (0to40 MeCN).

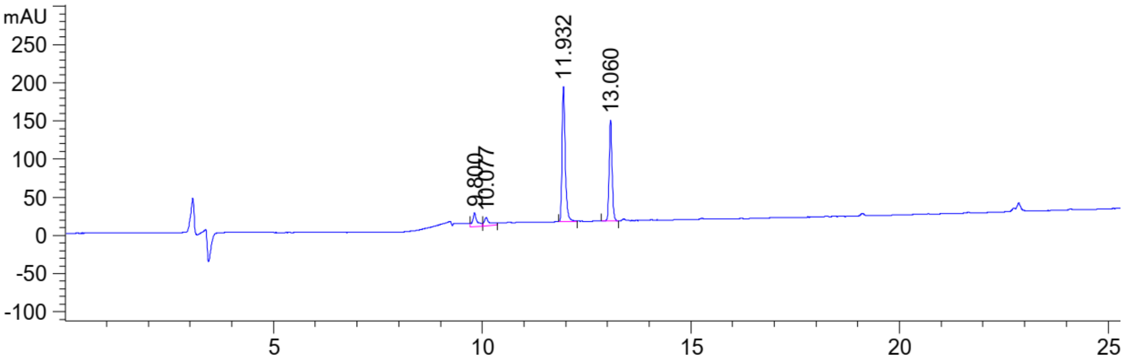

Figure S361: HPLC chromatogram of Smoc-L-Arg-OH **5** deprotection with 10% ethanolamine in Ethanol after 5min at λ=220 nm (0to40 MeCN).

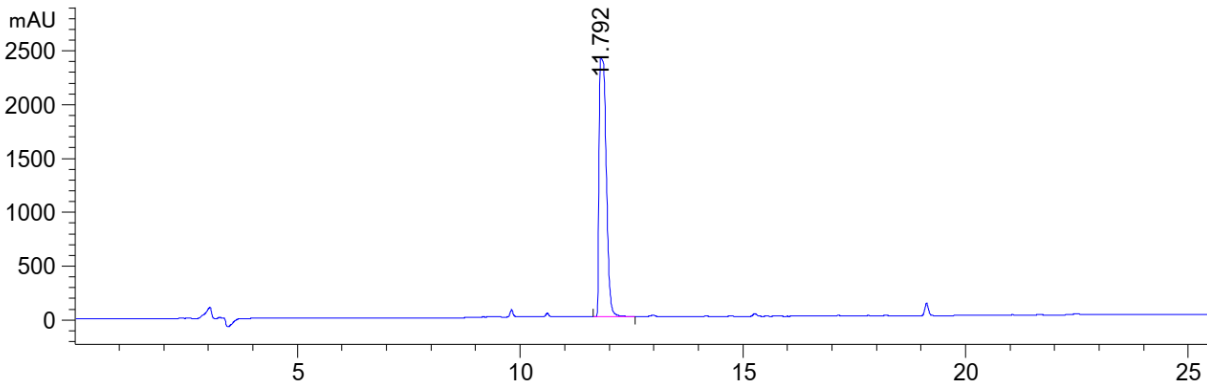

Figure S362: HPLC chromatogram of Smoc-L-Arg-OH **5** deprotection with 1M NaOH in water after 5min at λ=220 nm (0to40 MeCN).

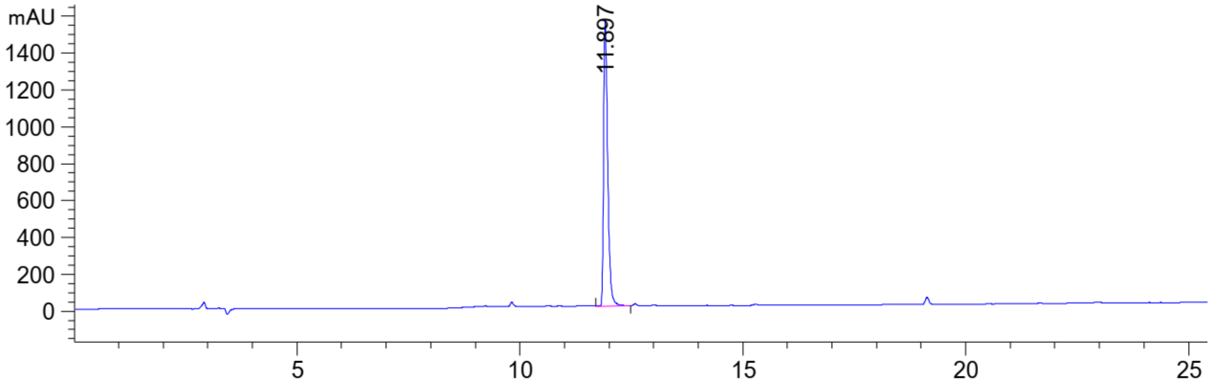

Figure S363: HPLC chromatogram of Smoc-L-Arg-OH **5** deprotection with 1M NaOH in ethanol after 5min at λ=220 nm (0to40 MeCN).

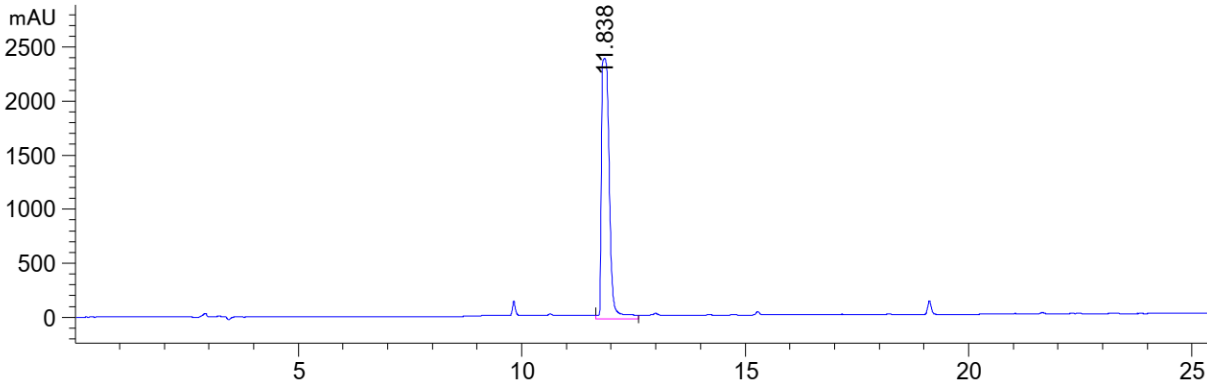

Figure S364: HPLC chromatogram of Smoc-L-Arg-OH **5** deprotection with 0.2M NaOH in water after 5min at λ=220 nm (0to40 MeCN).

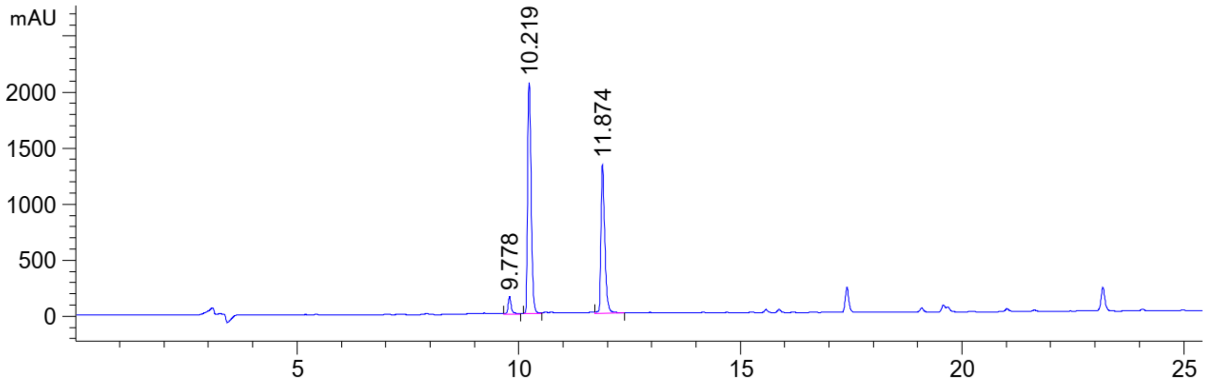

Figure S365: HPLC chromatogram of Smoc-L-Arg-OH **5** deprotection with 5% piperazine in water after 5min at λ=220 nm (0to40 MeCN).

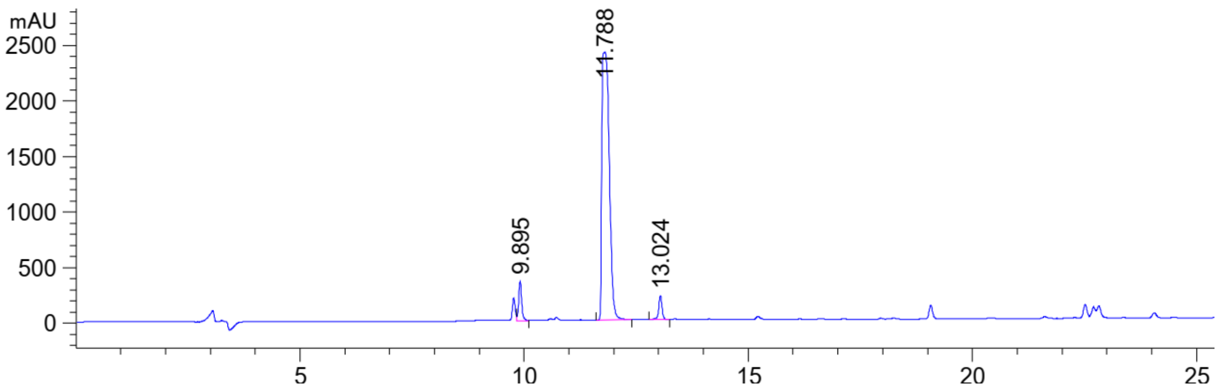

Figure S366: HPLC chromatogram of Smoc-L-Arg-OH **5** deprotection with 10% ammonia in water after 5min at λ=220 nm (0to40 MeCN).

3.3.2. Analytical data of Smoc-Leu-OH 16 deprotection

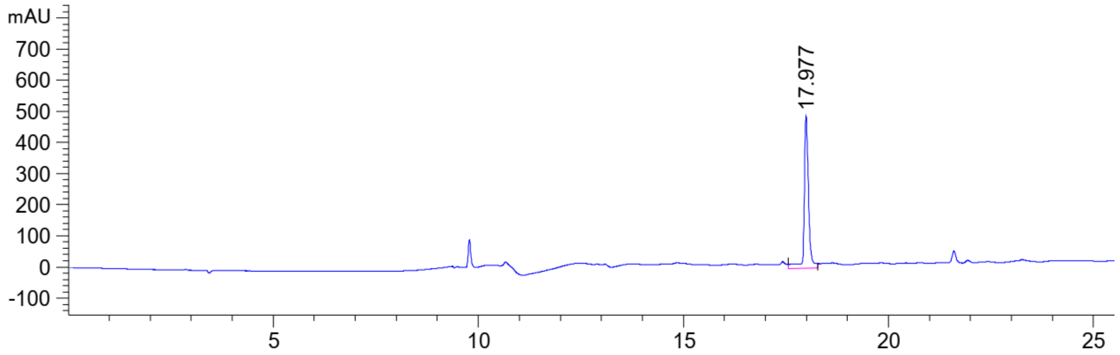

Figure S367: HPLC chromatogram of Smoc-L-Leu-OH **16** Ref for deprotection at λ=220 nm (0to40 MeCN).

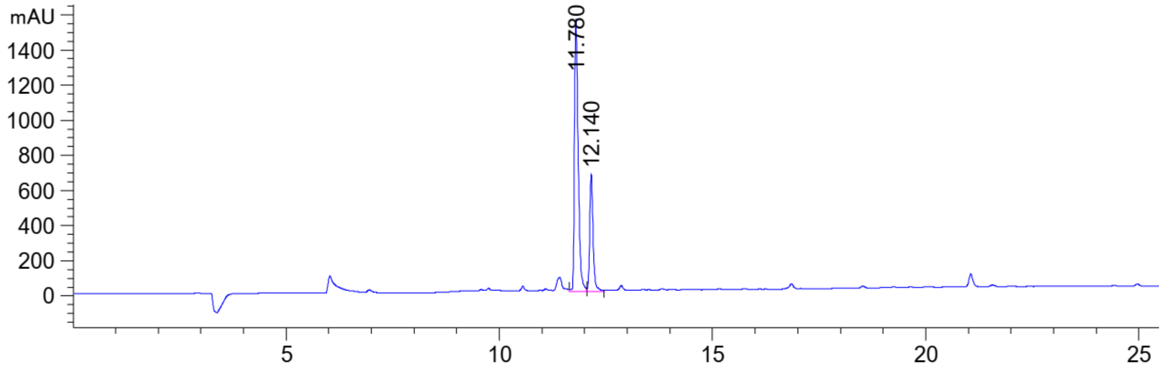

Figure S368: HPLC chromatogram of Smoc-L-Leu-OH **16** deprotection with 20% piperidine in water after 5min at λ=220 nm (0to40 MeCN).

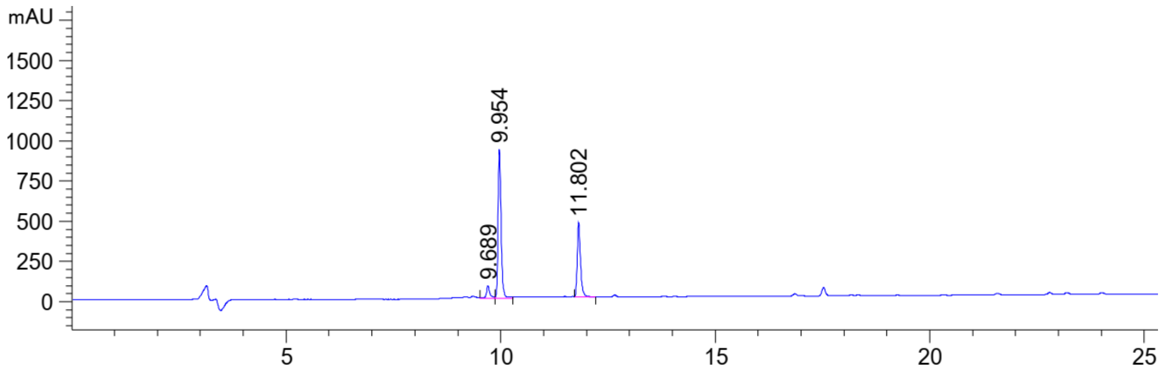

Figure S369: HPLC chromatogram of Smoc-L-Leu-OH **16** deprotection with 10% ethanolamine in water after 5min at λ=220 nm (0to40 MeCN).

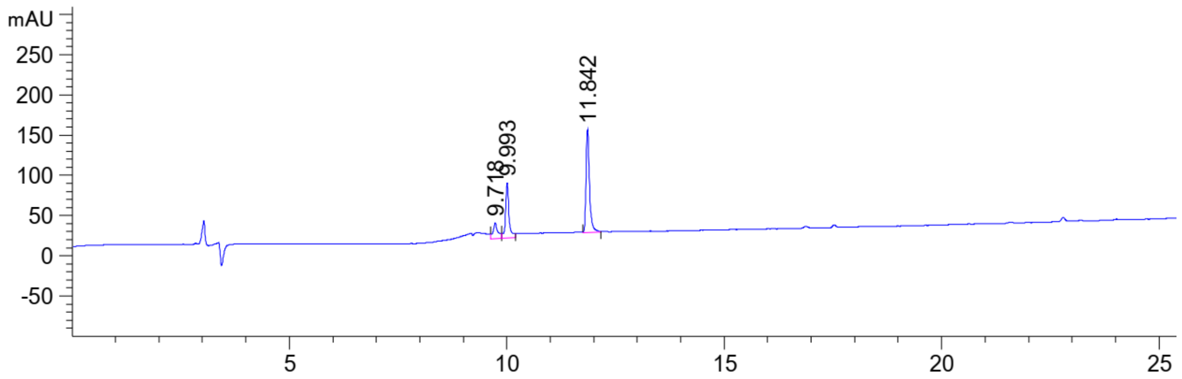

Figure S370: HPLC chromatogram of Smoc-L-Leu-OH **16** deprotection with 10% ethanolamine in Ethanol after 5min at λ=220 nm (0to40 MeCN).

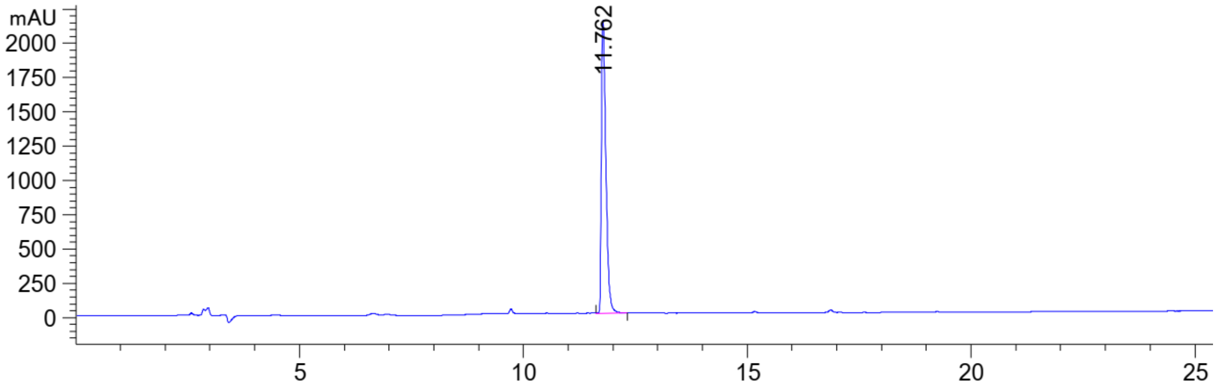

Figure S371: HPLC chromatogram of Smoc-L-Leu-OH **16** deprotection with 1M NaOH in water after 5min at λ=220 nm (0to40 MeCN).

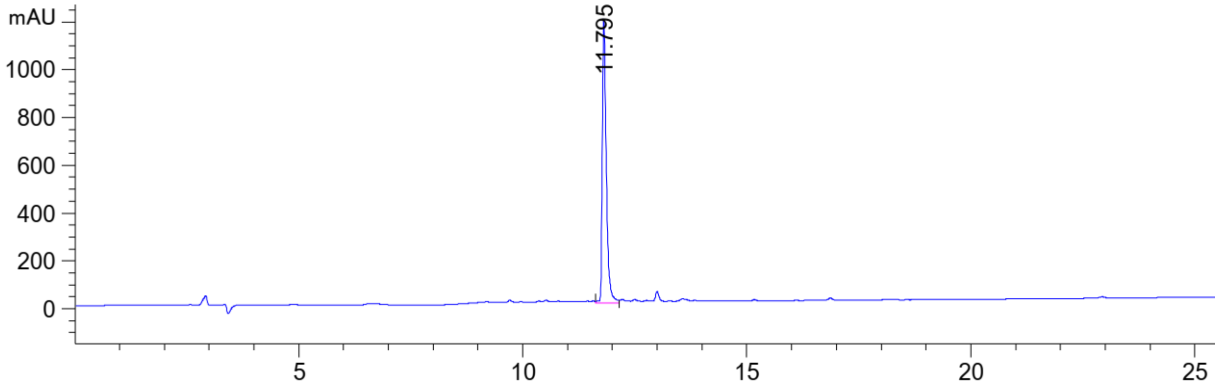

Figure S372: HPLC chromatogram of Smoc-L-Leu-OH **16** deprotection with 1M NaOH in ethanol after 5min at λ=220 nm (0to40 MeCN).

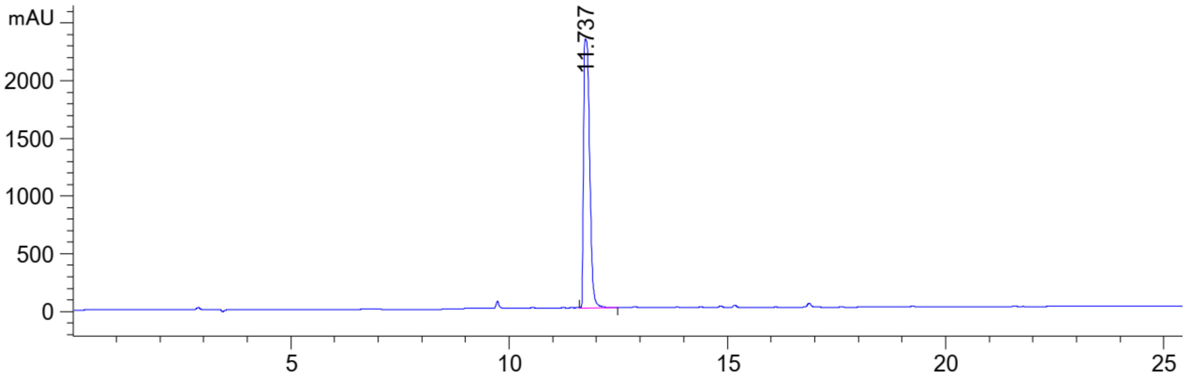

Figure S373: HPLC chromatogram of Smoc-L-Leu-OH **16** deprotection with 0.2M NaOH in water after 5min at λ=220 nm (0to40 MeCN).

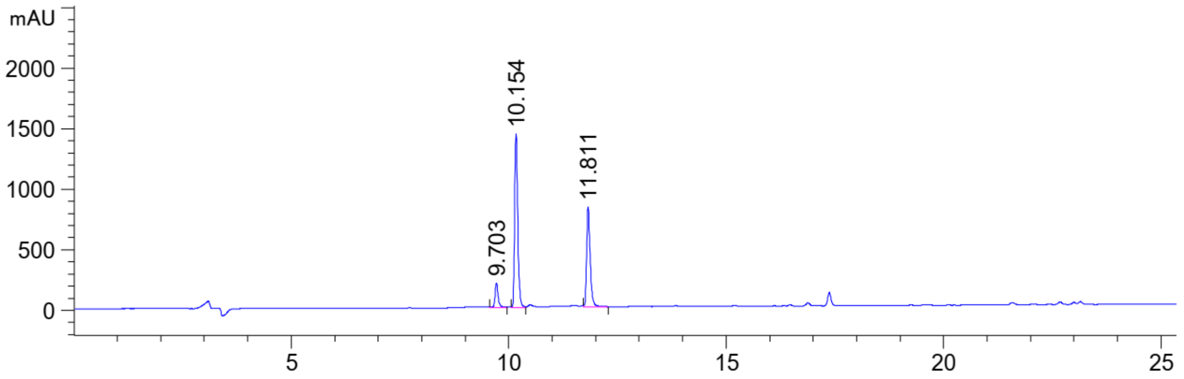

Figure S374: HPLC chromatogram of Smoc-L-Leu-OH **16** deprotection with 5% piperazine in water after 5min at λ=220 nm (0to40 MeCN).

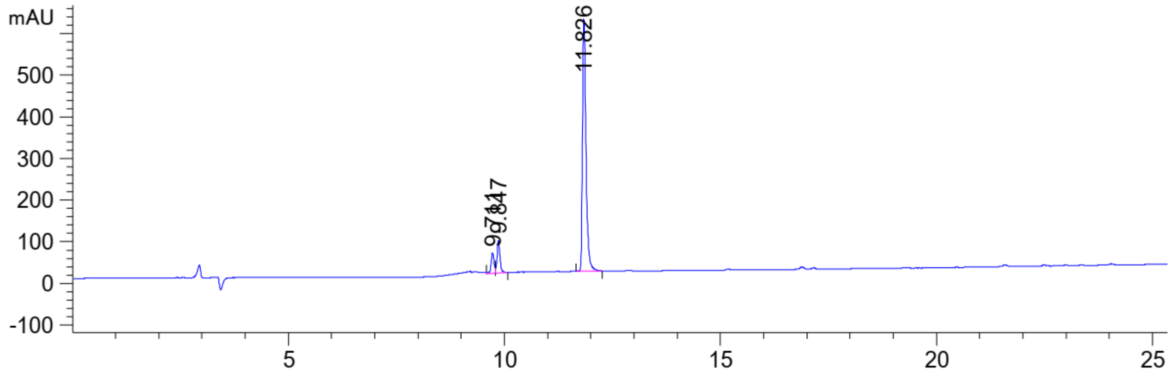

Figure S375: HPLC chromatogram of Smoc-L-Leu-OH **16** deprotection with 10% ammonia in water after 5min at λ=220 nm (0to40 MeCN).

3.3.3. Analytical data of Smoc-Tyr-OH 28 deprotection

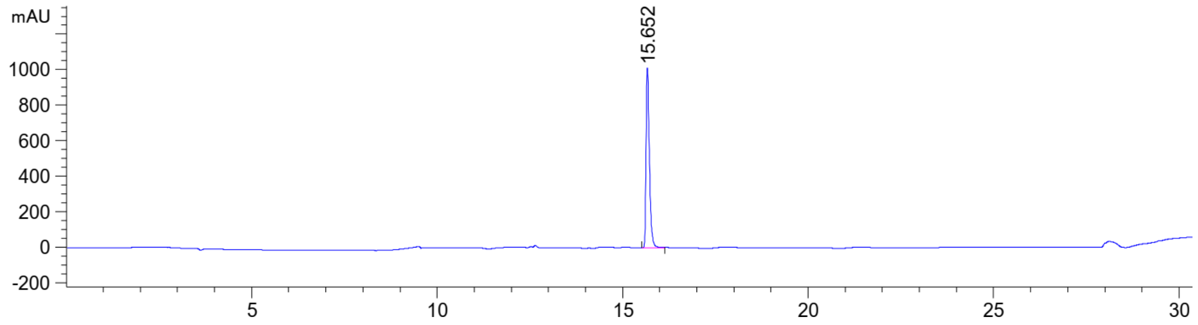

Figure S376: HPLC chromatogram of Smoc-L-Tyr-OH **28** Ref for deprotection at λ=220 nm (0to40 MeCN).

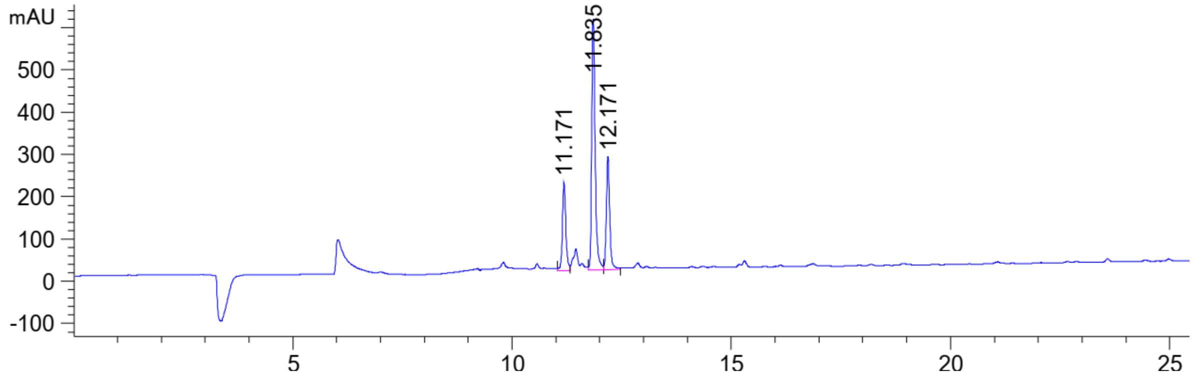

Figure S377: HPLC chromatogram of Smoc-L-Tyr-OH **28** deprotection with 20% piperidine in water after 5min at λ=220 nm (0to40 MeCN).

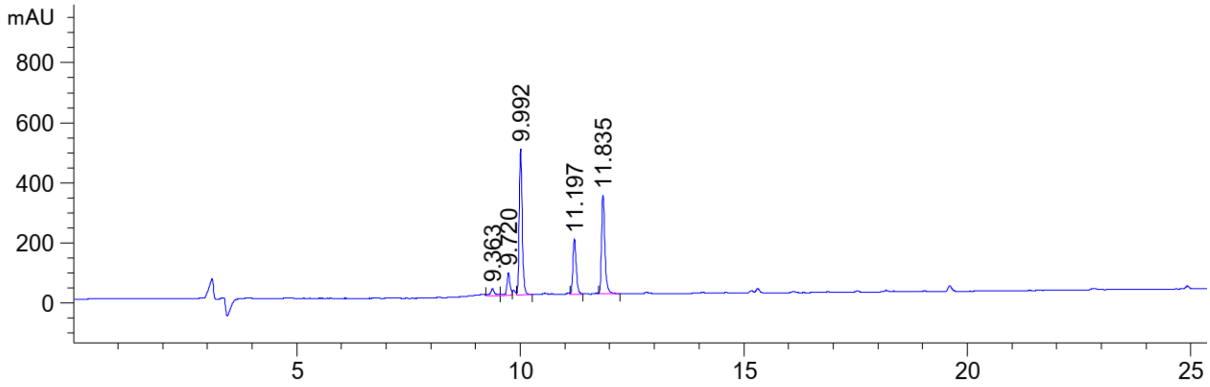

Figure S378: HPLC chromatogram of Smoc-L-Tyr-OH **28** deprotection with 10% ethanolamine in water after 5min at λ=220 nm (0to40 MeCN).

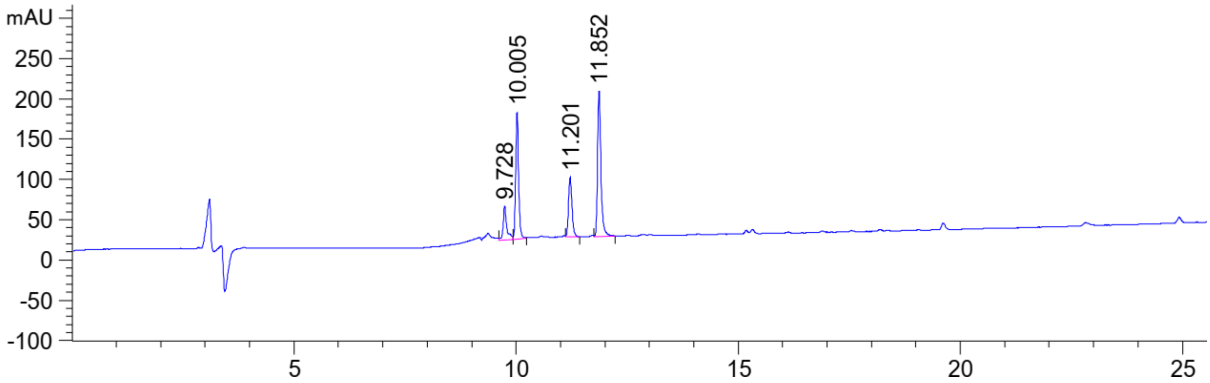

Figure S379: HPLC chromatogram of Smoc-L-Tyr-OH **28** deprotection with 10% ethanolamine in Ethanol after 5min at λ=220 nm (0to40 MeCN).

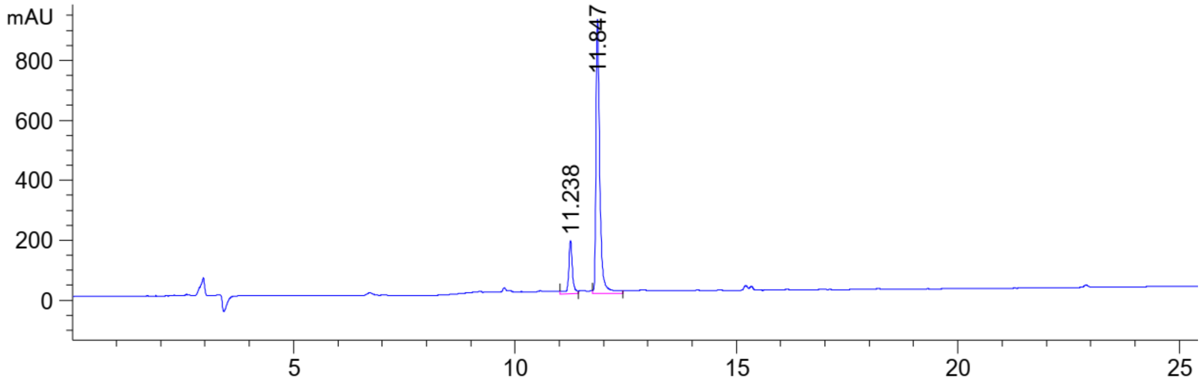

Figure S380: HPLC chromatogram of Smoc-L-Tyr-OH **28** deprotection with 1M NaOH in water after 5min at λ=220 nm (0to40 MeCN).

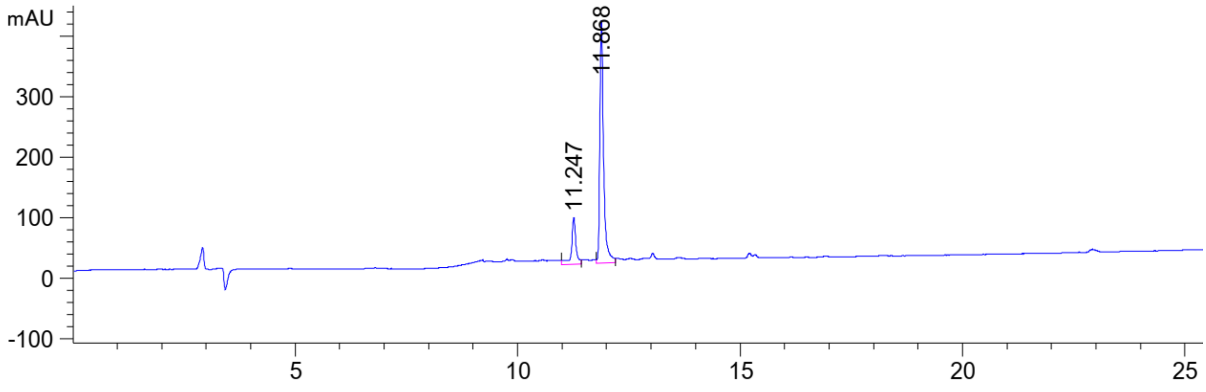

Figure S381: HPLC chromatogram of Smoc-L-Tyr-OH **28** deprotection with 1M NaOH in ethanol after 5min at λ=220 nm (0to40 MeCN).

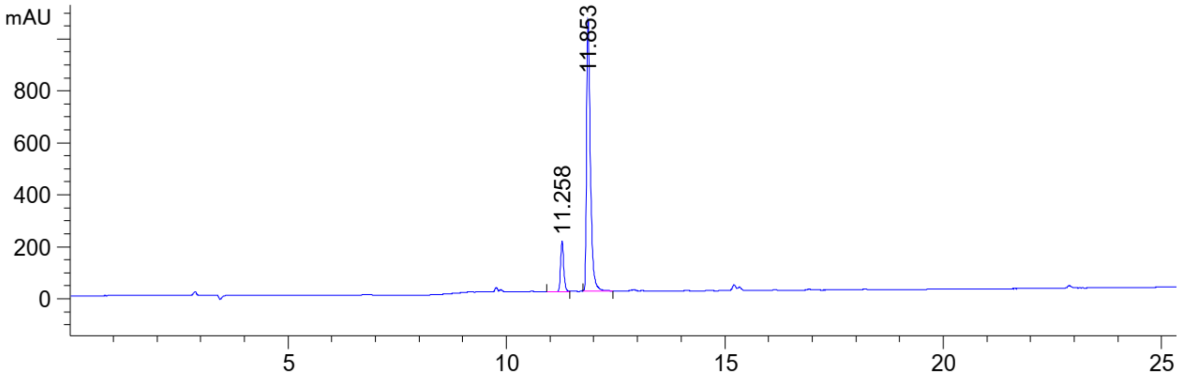

Figure S382: HPLC chromatogram of Smoc-L-Tyr-OH **28** deprotection with 0.2M NaOH in water after 5min at λ=220 nm (0to40 MeCN).

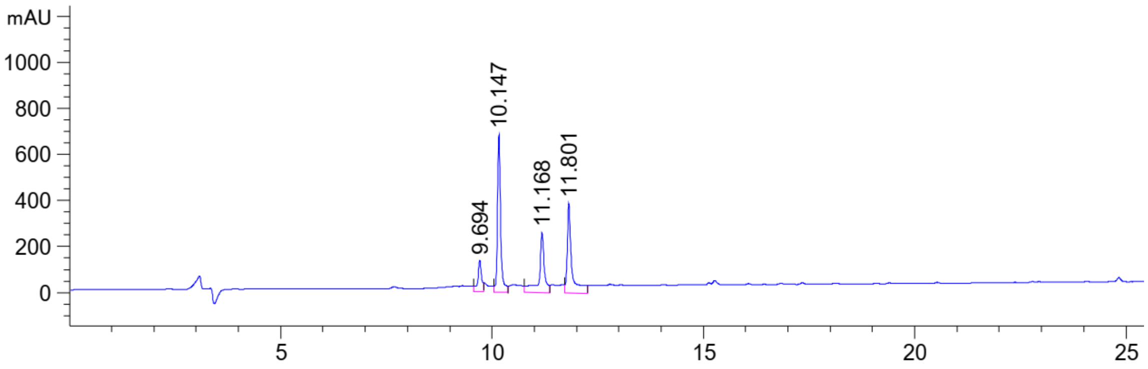

Figure S383: HPLC chromatogram of Smoc-L-Tyr-OH **28** deprotection with 5% piperazine in water after 5min at λ=220 nm (0to40 MeCN).

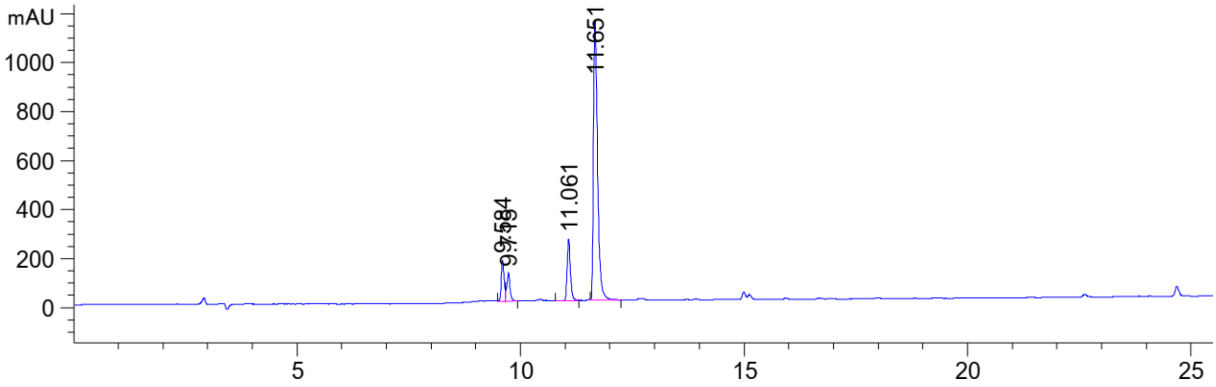

Figure S384: HPLC chromatogram of Smoc-L-Tyr-OH **28** deprotection with 10% ammonia in water after 5min at λ=220 nm (0to40 MeCN).

3.4. Analytical data of stability studies of Smoc-protected amino acids

3.4.1. Analytical data of Smoc-Arg-OH 5 stability studies

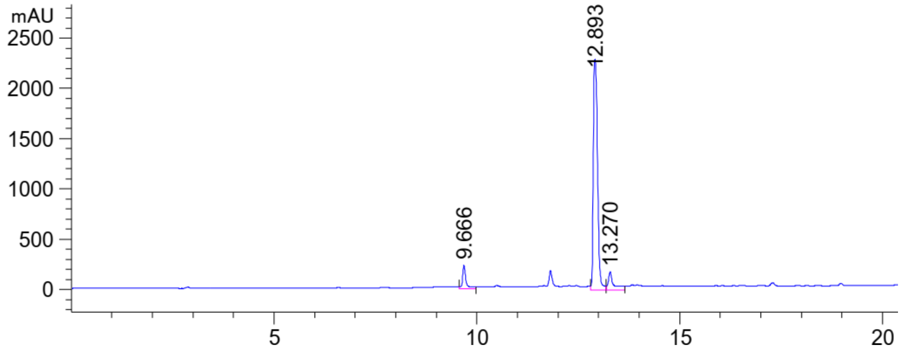

Figure S385: HPLC chromatogram of Smoc-L-Arg-OH **5** under reaction conditions reference at λ=220 nm (0to40 MeCN).

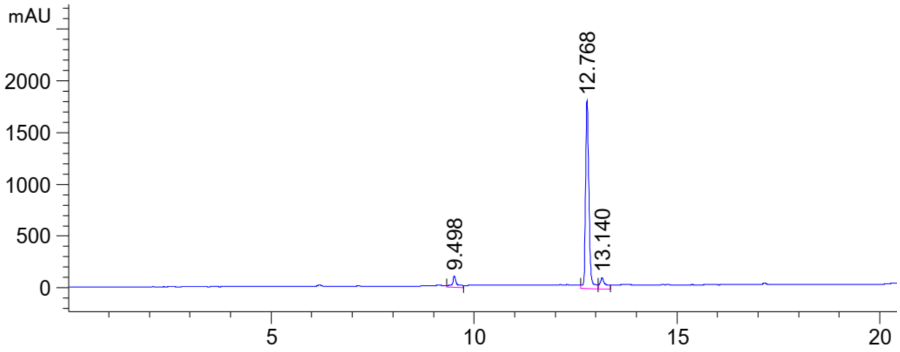

Figure S386: HPLC chromatogram of Smoc-L-Arg-OH **5** under reaction conditions after 7 days at λ=220 nm (0to40 MeCN).

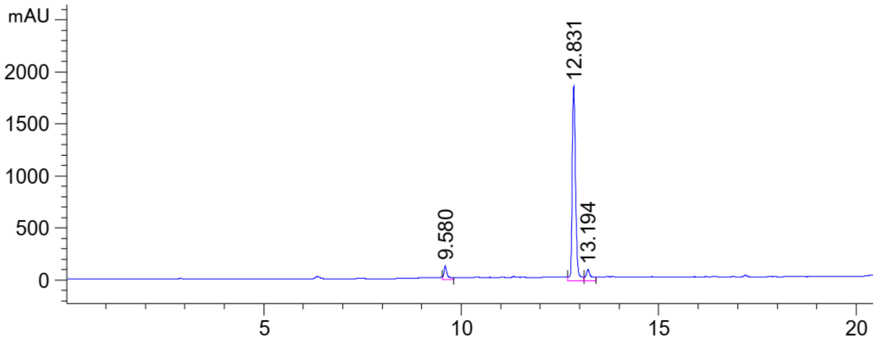

Figure S387: HPLC chromatogram of Smoc-L-Arg-OH **5** under reaction conditions after 14 days at λ=220-nm (0to40 MeCN).

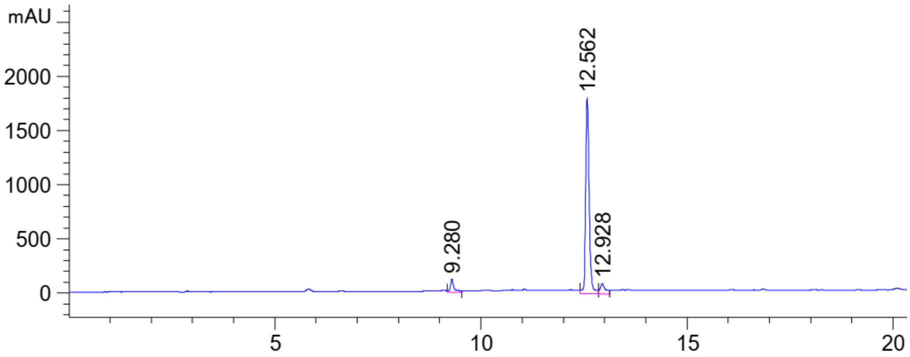

Figure S388: HPLC chromatogram of Smoc-L-Arg-OH **5** under reaction conditions after 21 days at λ=220-nm (0to40 MeCN).

3.4.2. Analytical data of Smoc-Ile-OH 15 stability studies

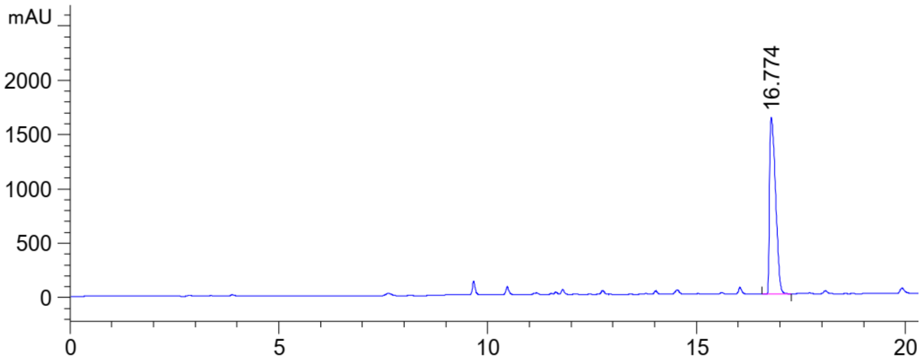

Figure S389: HPLC chromatogram of Smoc-L-Ile-OH **15** under reaction conditions reference at λ=220 nm (0to40 MeCN).

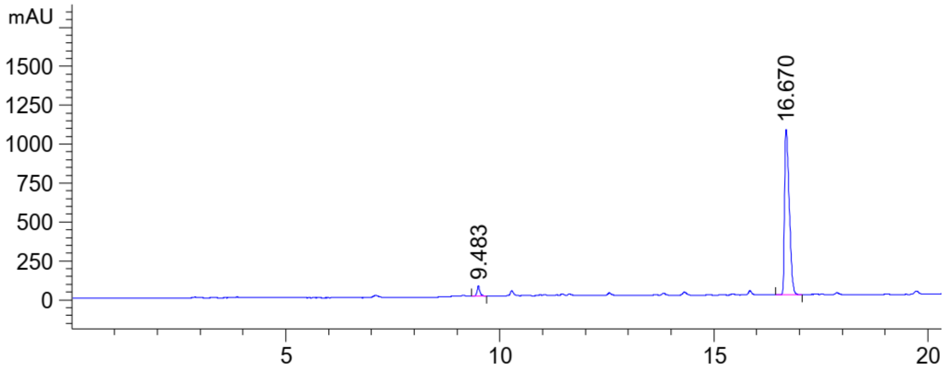

Figure S390: HPLC chromatogram of Smoc-L-Ile-OH **15** under reaction conditions after 7 days at λ=220 nm (0to40 MeCN).

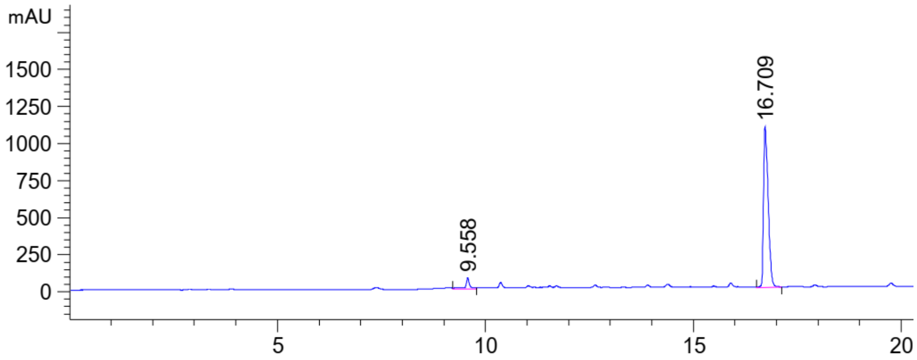

Figure S391: HPLC chromatogram of Smoc-L-Ile-OH **15** under reaction conditions after 14 days at λ=220-nm (0to40 MeCN).

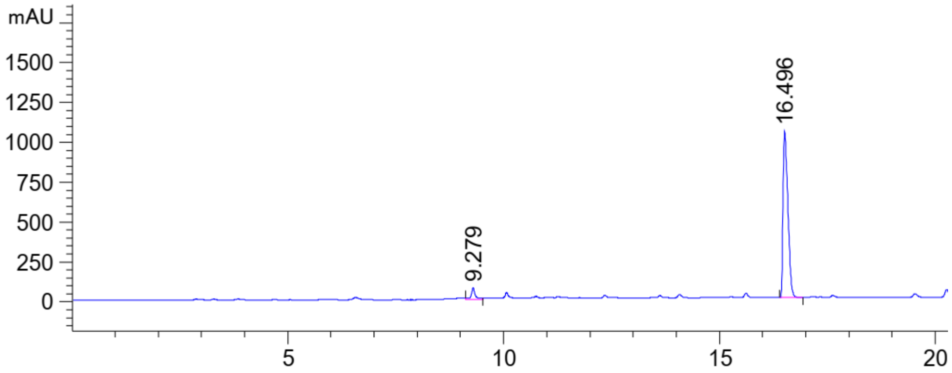

Figure S392: HPLC chromatogram of Smoc-L-Ile-OH **15** under reaction conditions after 21 days at λ=220-nm (0to40 MeCN).

3.4.3. Analytical data of Smoc-Phe-OH 20 stability studies

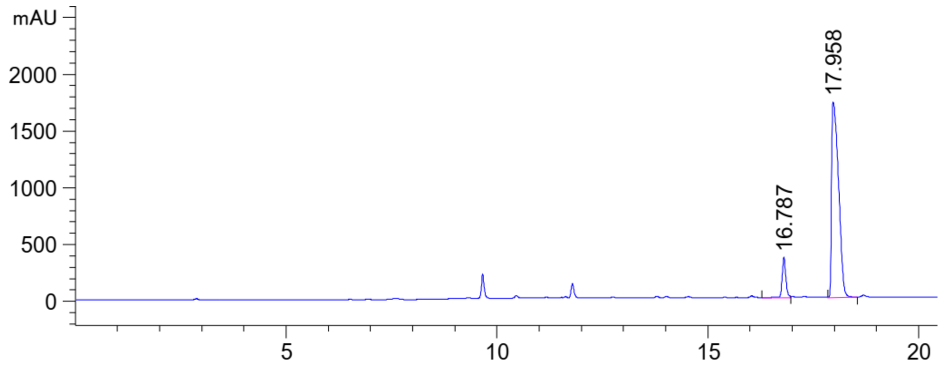

Figure S393: HPLC chromatogram of Smoc-L-Phe-OH **20** under reaction conditions reference at λ=220 nm (0to40 MeCN).

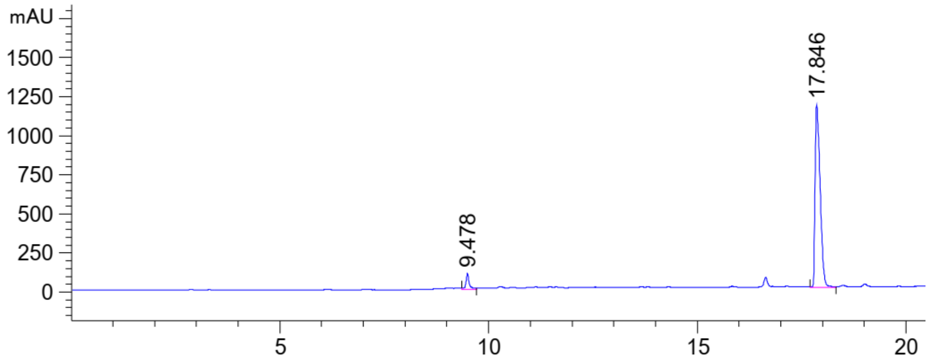

Figure S394: HPLC chromatogram of Smoc-L-Phe-OH **20** under reaction conditions after 7 days at λ=220 nm (0to40 MeCN).

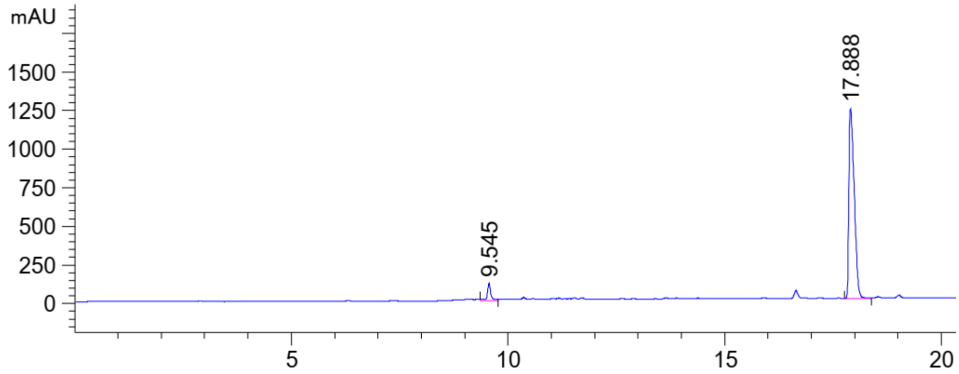

Figure S395: HPLC chromatogram of Smoc-L-Phe-OH **20** under reaction conditions after 14 days at λ=220-nm (0to40 MeCN).

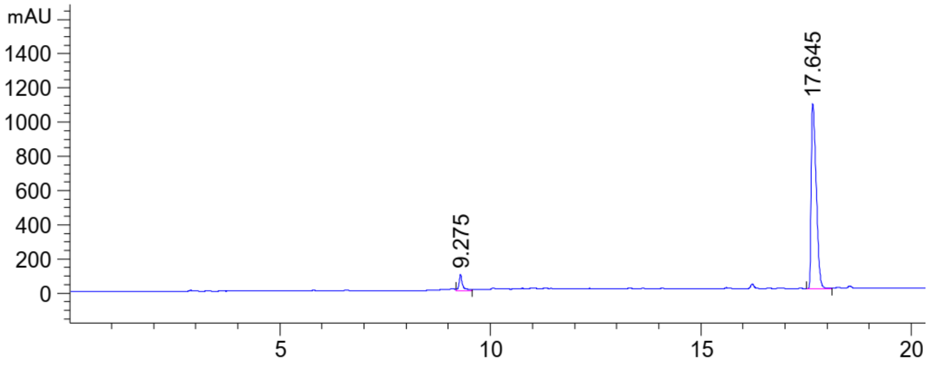

Figure S396: HPLC chromatogram of Smoc-L-Phe-OH **20** under reaction conditions after 21 days at λ=220-nm (0to40 MeCN).

3.4.4. Analytical data of Smoc-Pro-OH 21 stability studies

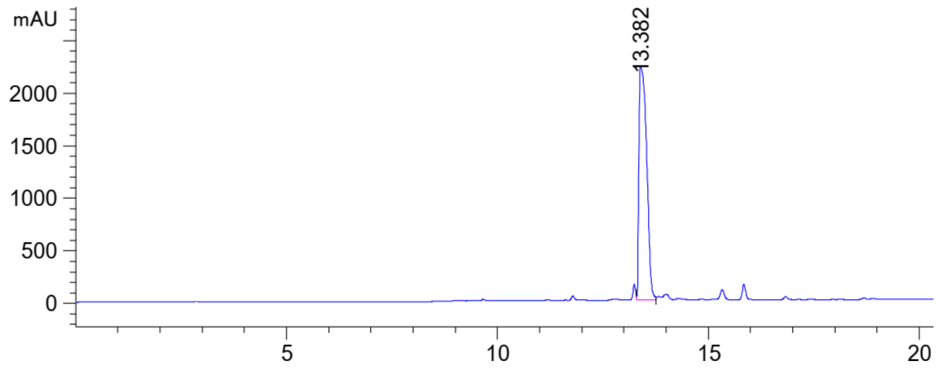

Figure S397: HPLC chromatogram of Smoc-L-Pro-OH **21** under reaction conditions reference at λ=220 nm (0to40 MeCN).

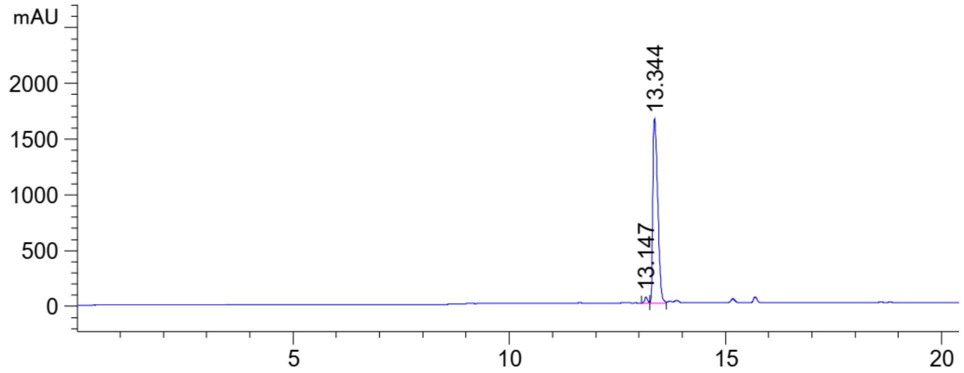

Figure S398: HPLC chromatogram of Smoc-L-Pro-OH **21** under reaction conditions after 7 days at λ=220 nm (0to40 MeCN).

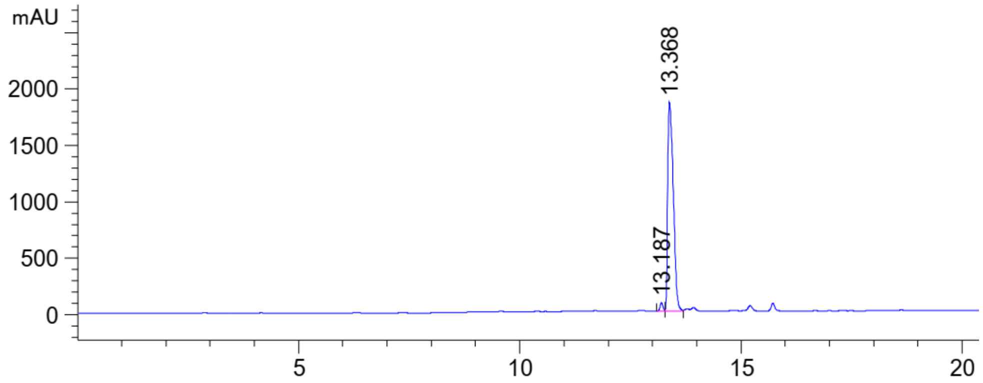

Figure S399: HPLC chromatogram of Smoc-L-Pro-OH **21** under reaction conditions after 14 days at λ=220-nm (0to40 MeCN).

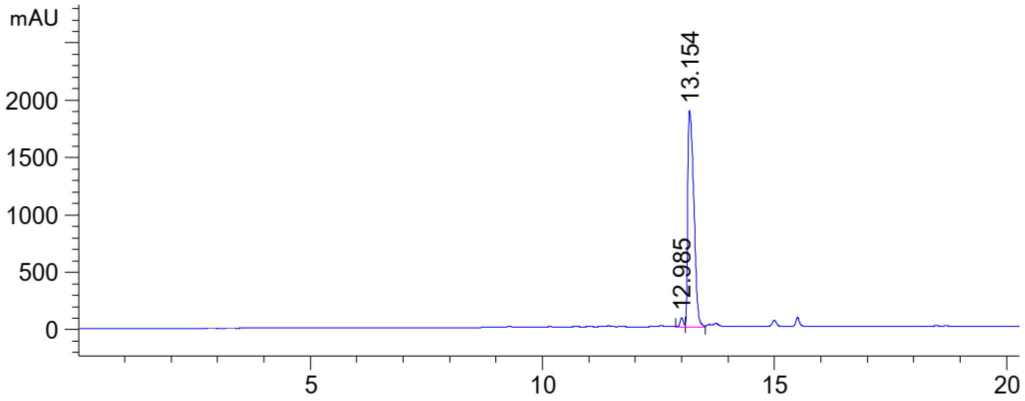

Figure S400: HPLC chromatogram of Smoc-L-Pro-OH **21** under reaction conditions after 21 days at λ=220-nm (0to40 MeCN).

3.4.5. Analytical data of Smoc-Ser-OH 22 stability studies

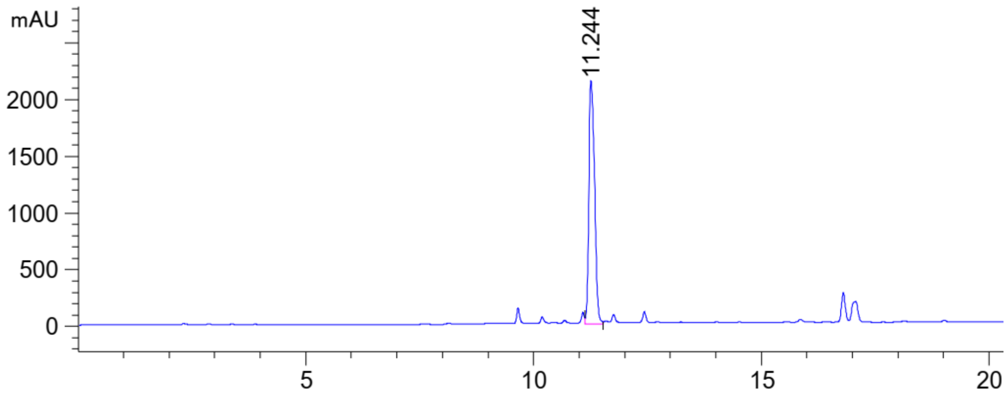

Figure S401: HPLC chromatogram of Smoc-L-Ser-OH **22** under reaction conditions reference at λ=220 nm (0to40 MeCN).

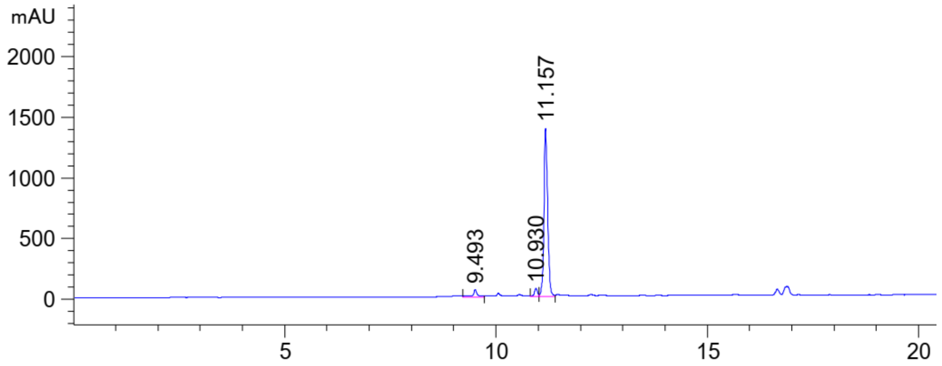

Figure S402: HPLC chromatogram of Smoc-L-Ser-OH **22** under reaction conditions after 7 days at λ=220 nm (0to40 MeCN).

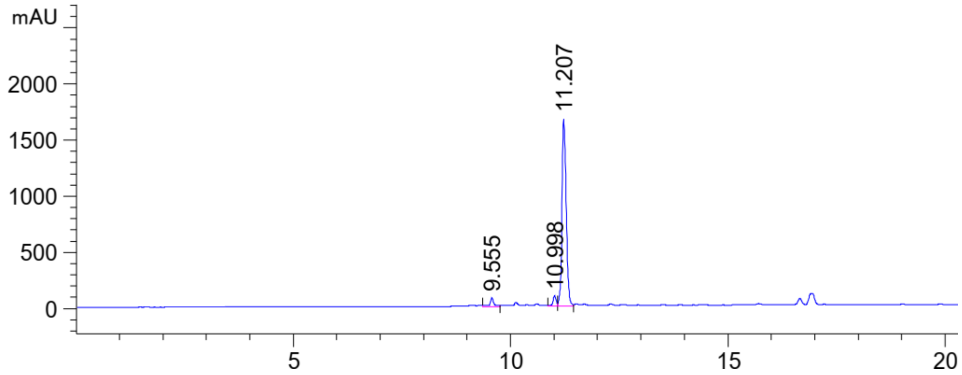

Figure S403: HPLC chromatogram of Smoc-L-Ser-OH **22** under reaction conditions after 14 days at λ=220-nm (0to40 MeCN).

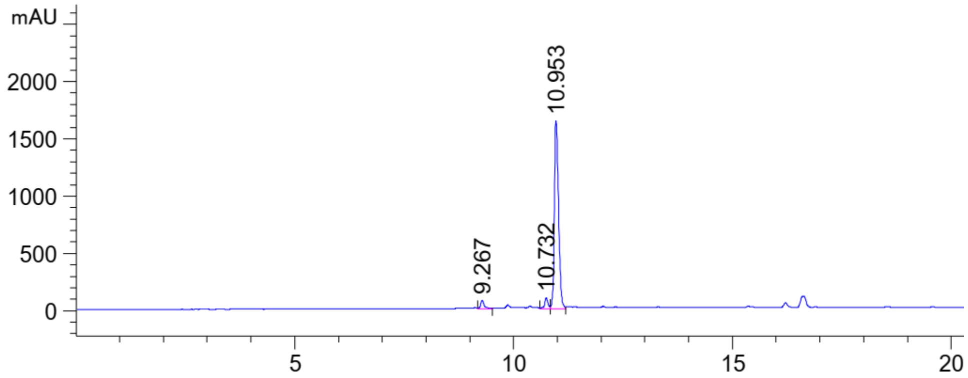

Figure S404: HPLC chromatogram of Smoc-L-Ser-OH **22** under reaction conditions after 21 days at λ=220-nm (0to40 MeCN).

3.5. Analytical data of coupling efficiency and solvent influence

3.5.1. Analytical Reference data

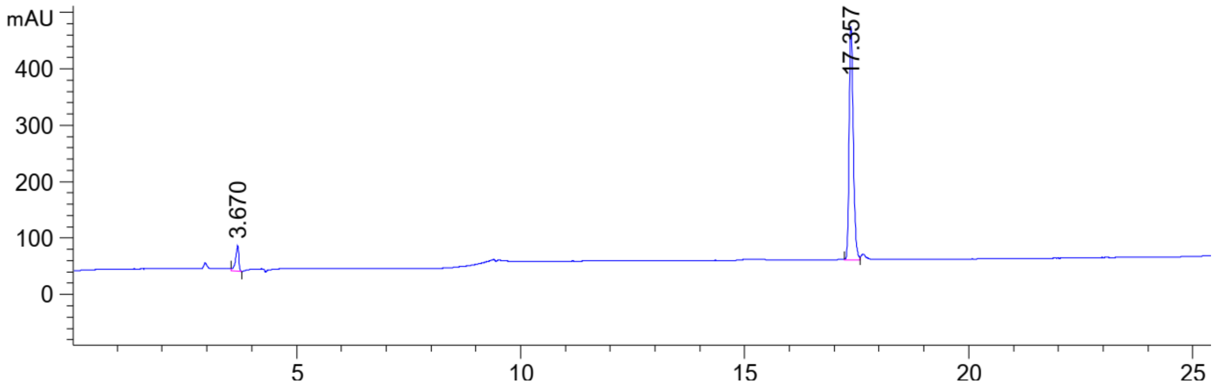

Figure S405: HPLC chromatogram of Smoc-L-Pro-L-Tyr-OMe **36** Reference at λ=220 nm (0to40 MeCN).

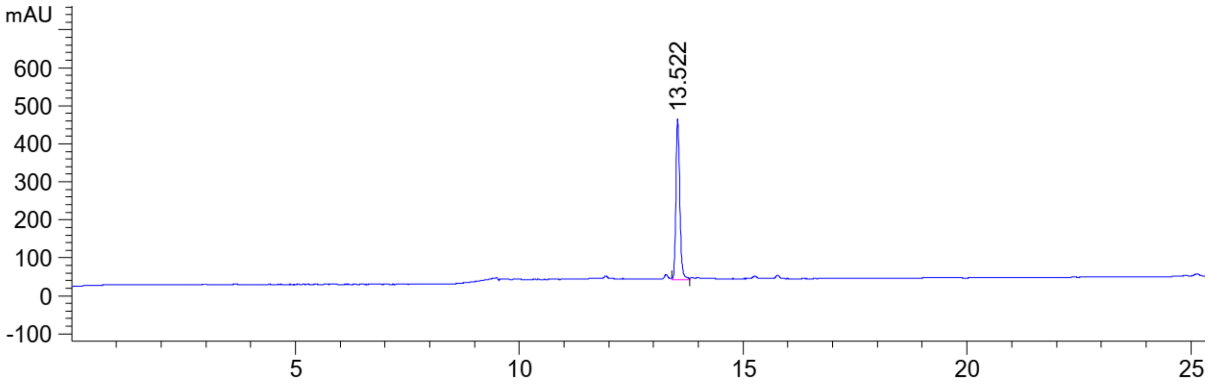

Figure S406: HPLC chromatogram of Smoc-L-Pro-OH **21** Reference at λ=220 nm (0to40 MeCN).

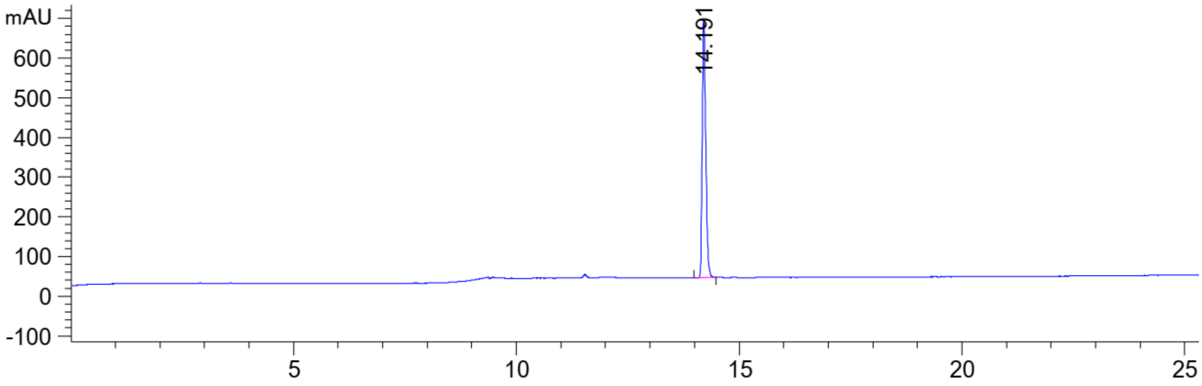

Figure S407: HPLC chromatogram of L-Tyr-OMe **35** Reference at λ=220 nm (0to40 MeCN).

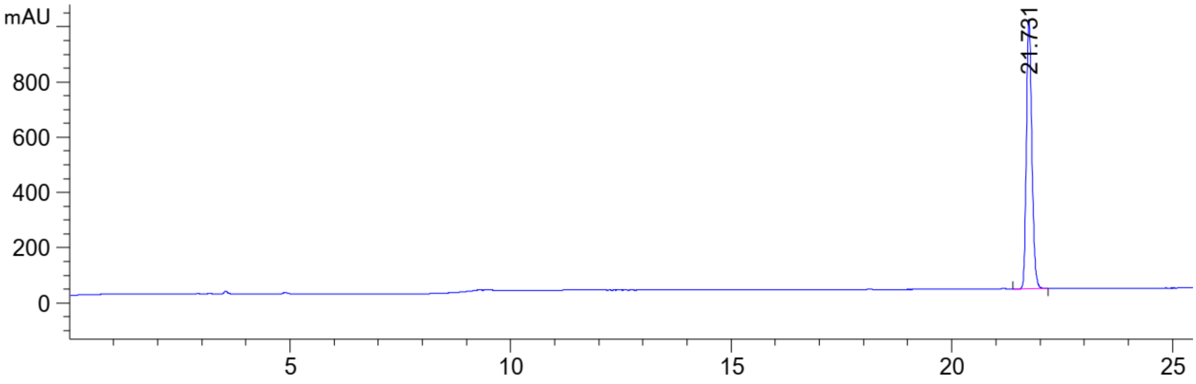

Figure S408: HPLC chromatogram of Oxyma **39** Reference at λ=220 nm (0to40 MeCN).

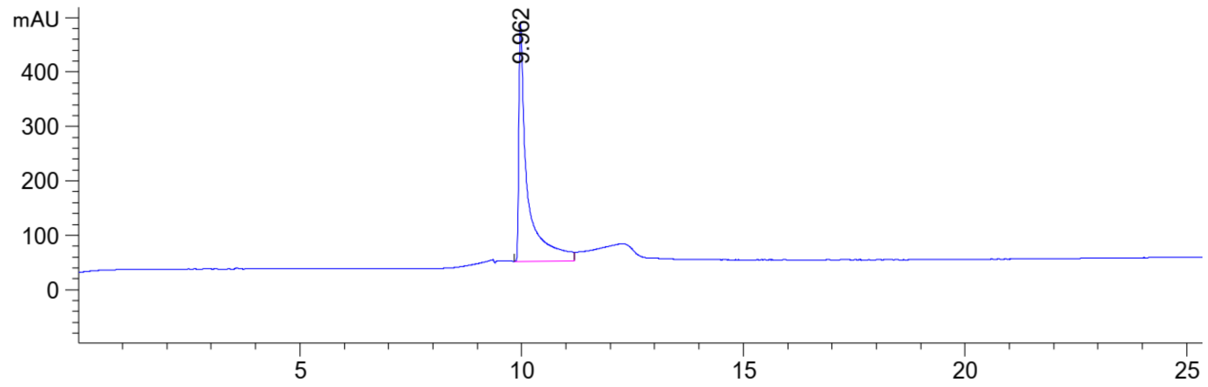

Figure S409: HPLC chromatogram of HOPO **40** Reference at λ=220 nm (0to40 MeCN).

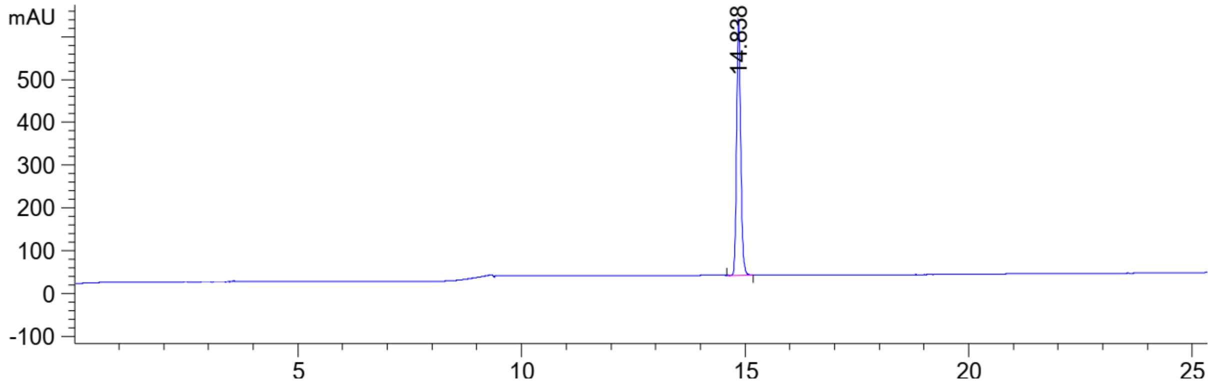

Figure S410: HPLC chromatogram of HONB **41** Reference at λ=220 nm (0to40 MeCN).

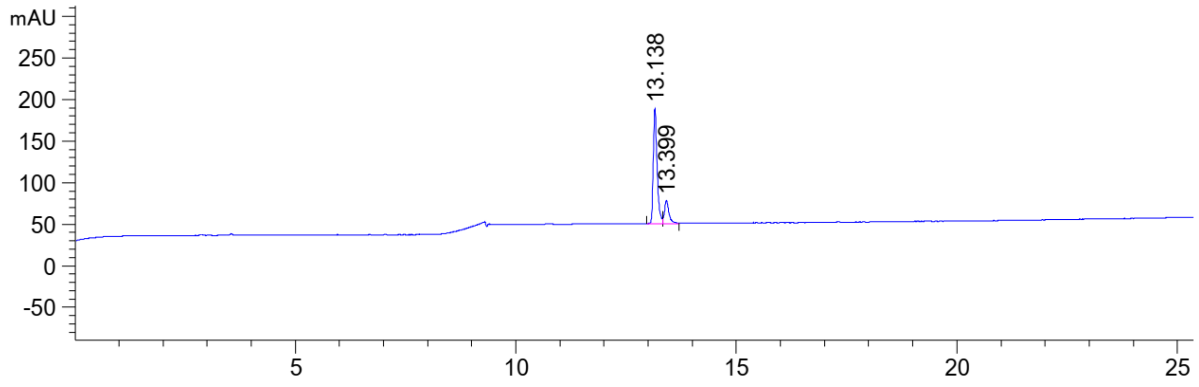

Figure S411: HPLC chromatogram of EEDQ **42** Reference at λ=220 nm (0to40 MeCN).

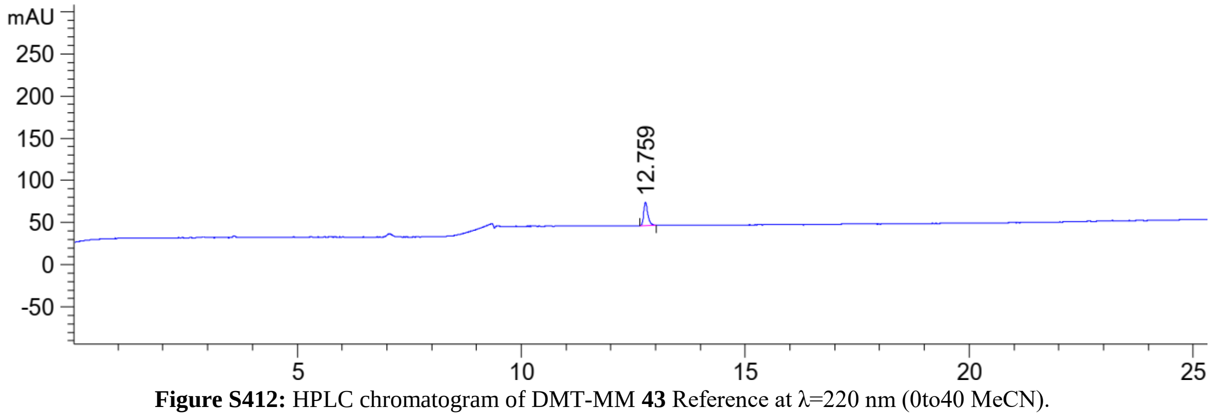

Figure S412: HPLC chromatogram of DMT-MM **43** Reference at λ=220 nm (0to40 MeCN).

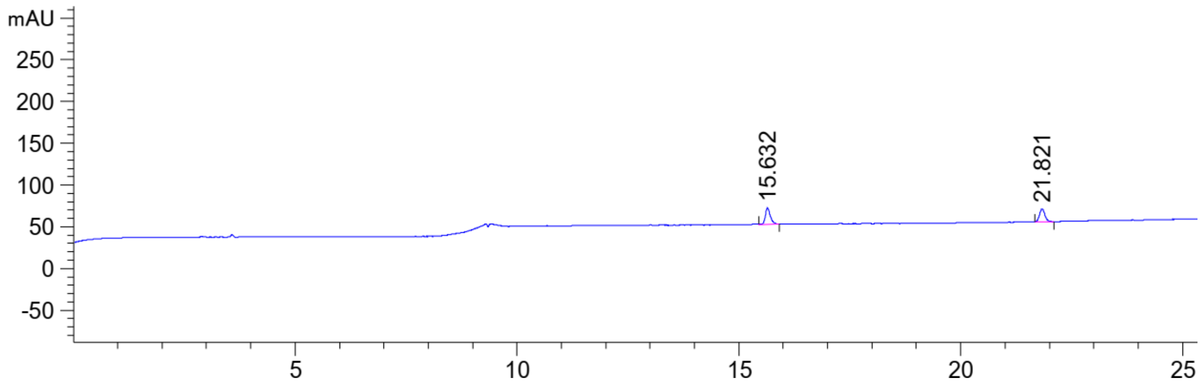

Figure S413: HPLC chromatogram of COMU **44** Reference at λ=220 nm (0to40 MeCN).

3.5.2. ESI-MS data of isolated side products

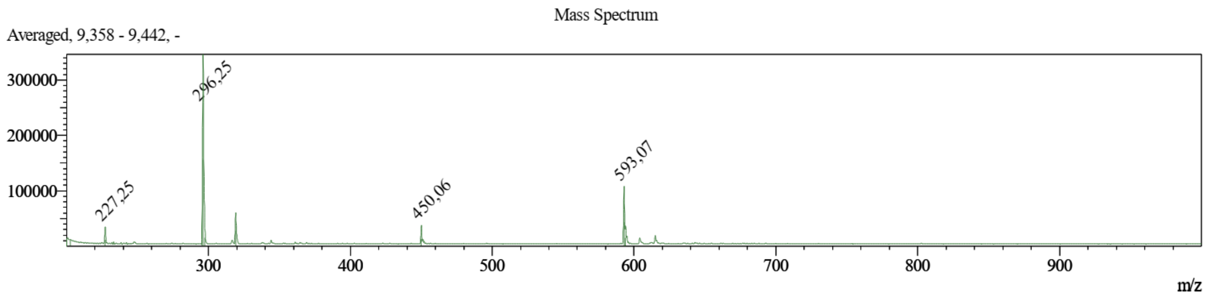

Figure S414: ESI-MS of isolated Smoc-Pro-NHS **45** (M measured=593.07 $[M-H]^-$, M calc.=592.54) at 15.5min.

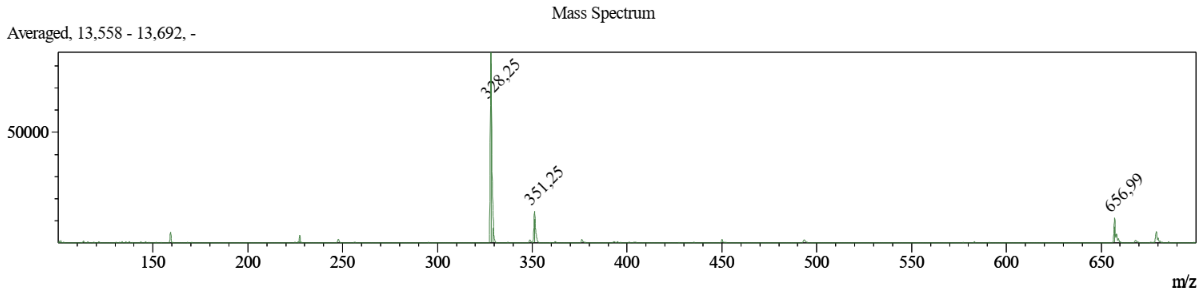

Figure S415: ESI-MS of isolated Smoc-Pro-HONB **46** (M measured=656.99 $[M-H]^-$, M calc.=656.63) at 20.55min.

3.5.3. Analytical data of the synthesis of Smoc-L-Pro-L-Tyr-OMe 36 in water

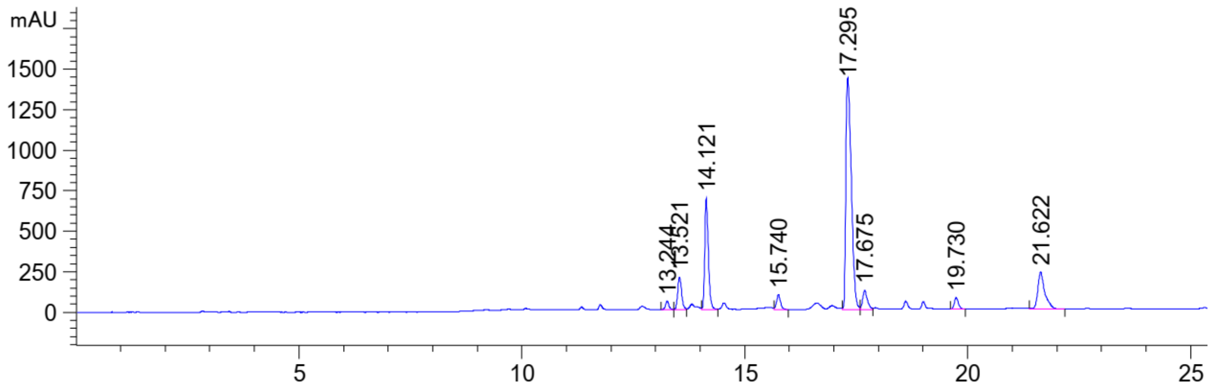

Figure S416: HPLC chromatogram of the synthesis of Smoc-L-Pro-L-Tyr-OMe **36** (17.3min) with EDC-HCl **37**/Oxyma **39** in water after 25min at λ=220 nm (0to40 MeCN).

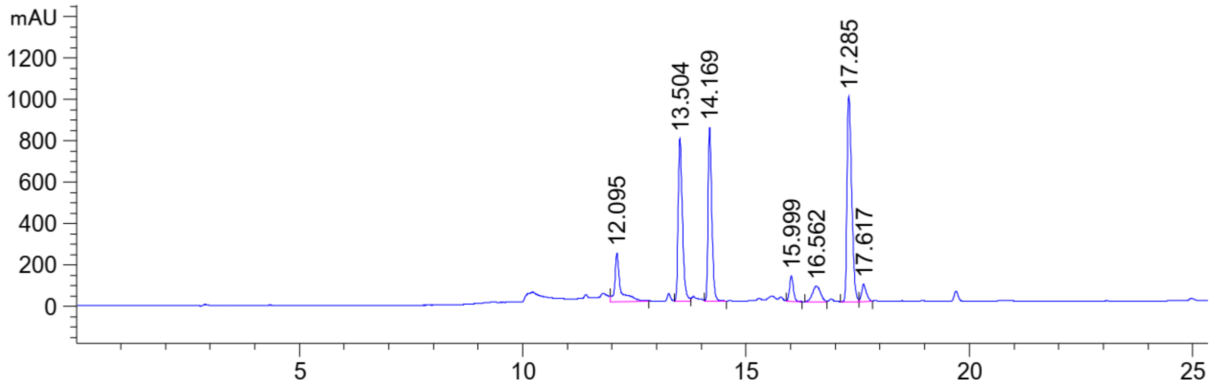

Figure S417: HPLC chromatogram of the synthesis of Smoc-L-Pro-L-Tyr-OMe **36** (17.3min) with EDC-HCl **37**/HOPO **40** in water after 25min at λ=220 nm (0to40 MeCN).

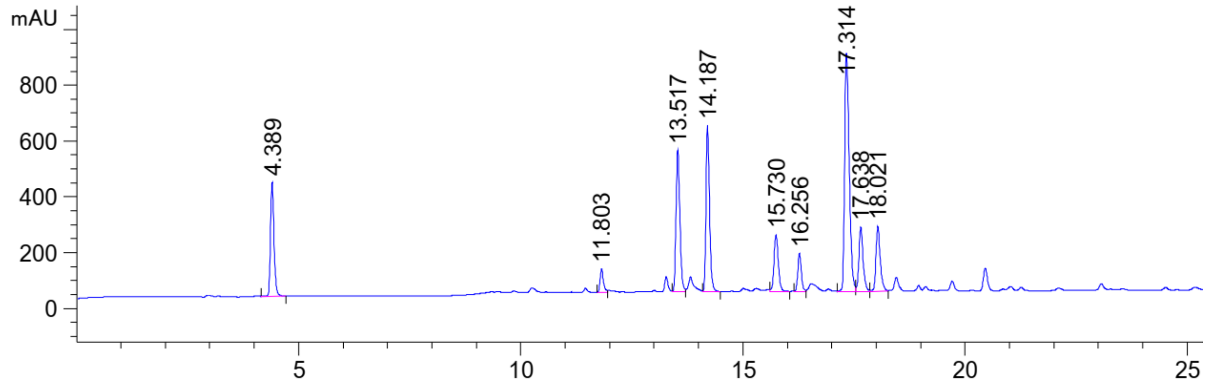

Figure S418: HPLC chromatogram of the synthesis of Smoc-L-Pro-L-Tyr-OMe **36** (17.3min) with EDC-HCl **37**/NHS **38** in water after 25min at λ=220 nm (0to40 MeCN).

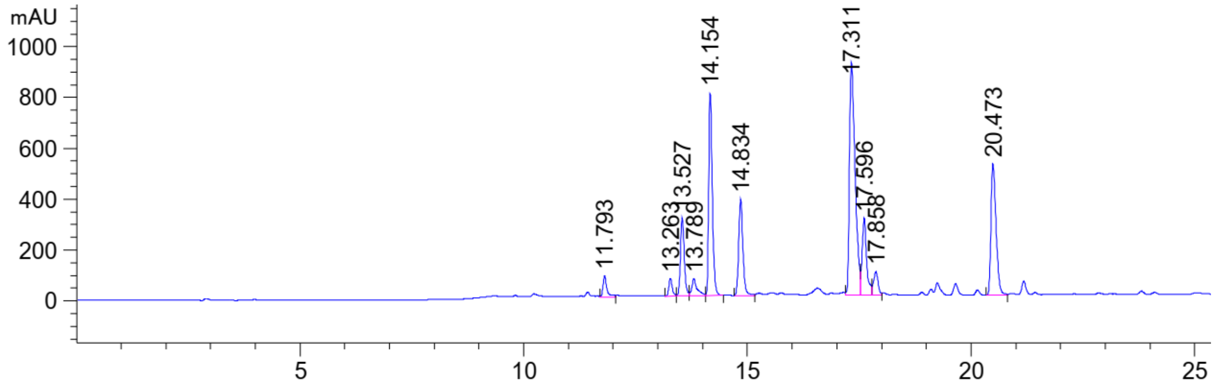

Figure S419: HPLC chromatogram of the synthesis of Smoc-L-Pro-L-Tyr-OMe **36** (17.3min) with EDC-HCl **37**/HONB **41** in water after 25min at λ=220 nm (0to40 MeCN).

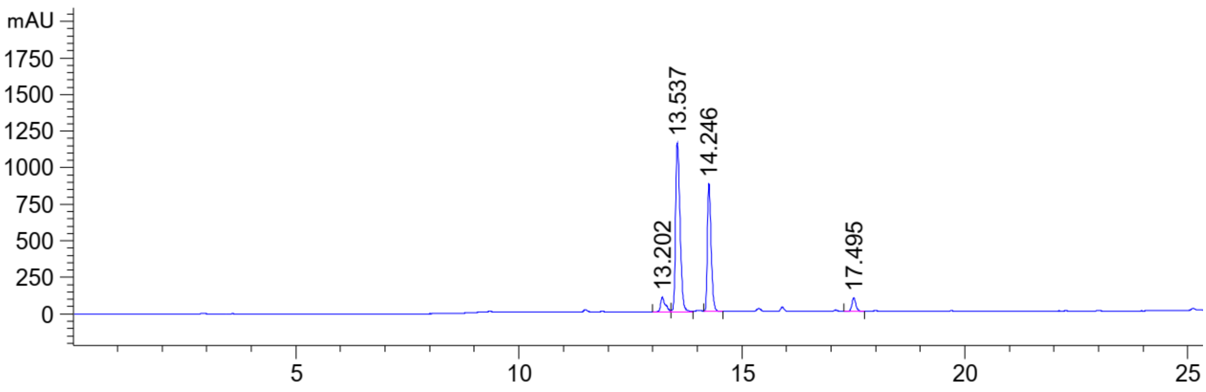

Figure S420: HPLC chromatogram of the synthesis of Smoc-L-Pro-L-Tyr-OMe **36** (17.5min) with EEDQ **42** in water after 25min at λ=220 nm (0to40 MeCN).

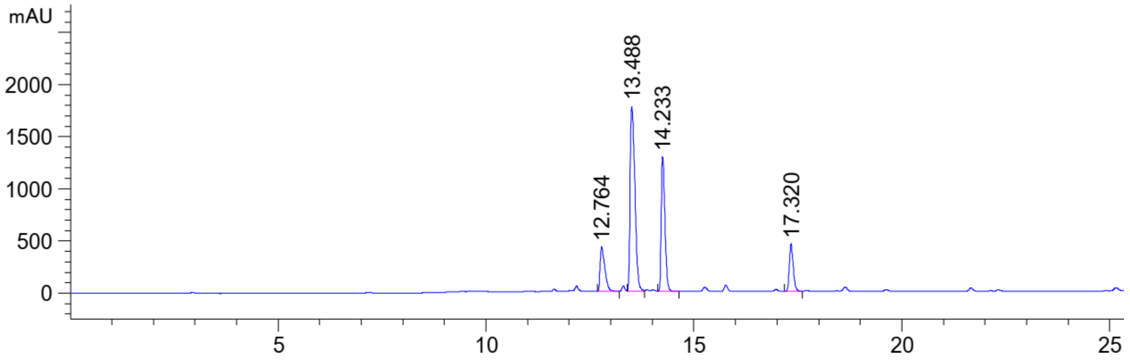

Figure S421: HPLC chromatogram of the synthesis of Smoc-L-Pro-L-Tyr-OMe **36** (17.3min) with DMT-MM **43** in water after 25min at λ=220 nm (0to40 MeCN).

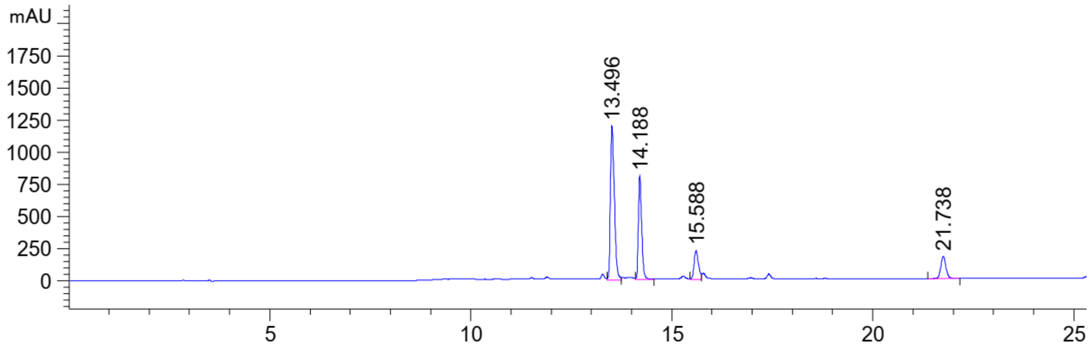

Figure S422: HPLC chromatogram of the synthesis of Smoc-L-Pro-L-Tyr-OMe **36** (17.2min) with COMU **44** in water after 25min at λ=220 nm (0to40 MeCN).

3.5.4. Analytical data of the synthesis of Smoc-Pro-Tyr-OMe 36 in 30% aq. MeCN

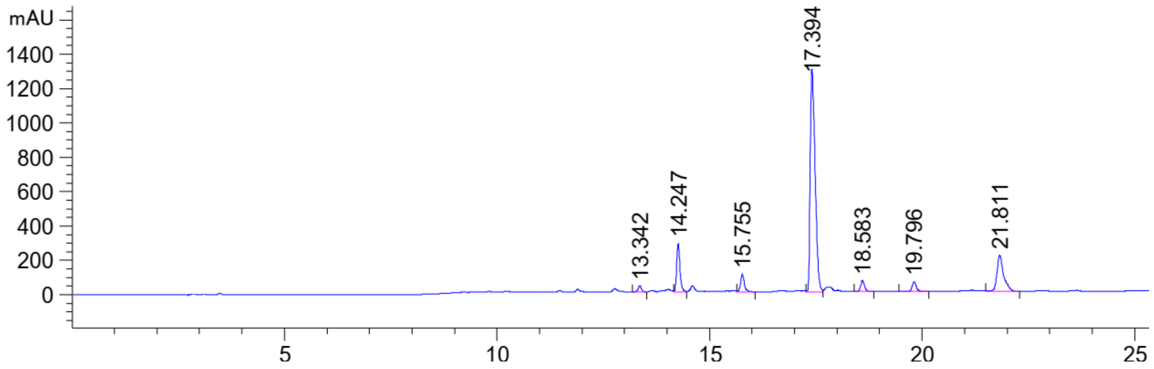

Figure S423: HPLC chromatogram of the synthesis of Smoc-L-Pro-L-Tyr-OMe **36** (17.4min) with EDC-HCl **37**/Oxyma **39** in 30% aq. MeCN after 25min at λ=220 nm (0to40 MeCN).

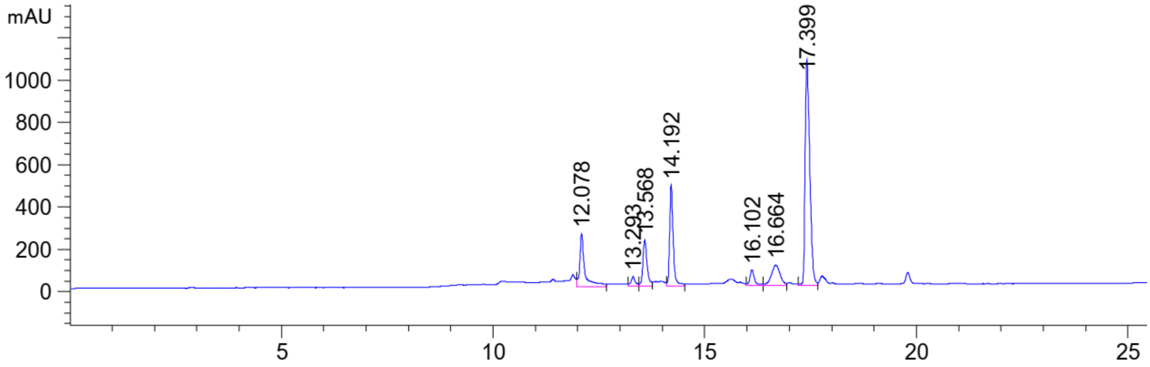

Figure S424: HPLC chromatogram of the synthesis of Smoc-L-Pro-L-Tyr-OMe **36** (17.4min) with EDC-HCl **37**/HOPO **40** in 30% aq. MeCN after 25min at λ=220 nm (0to40 MeCN).

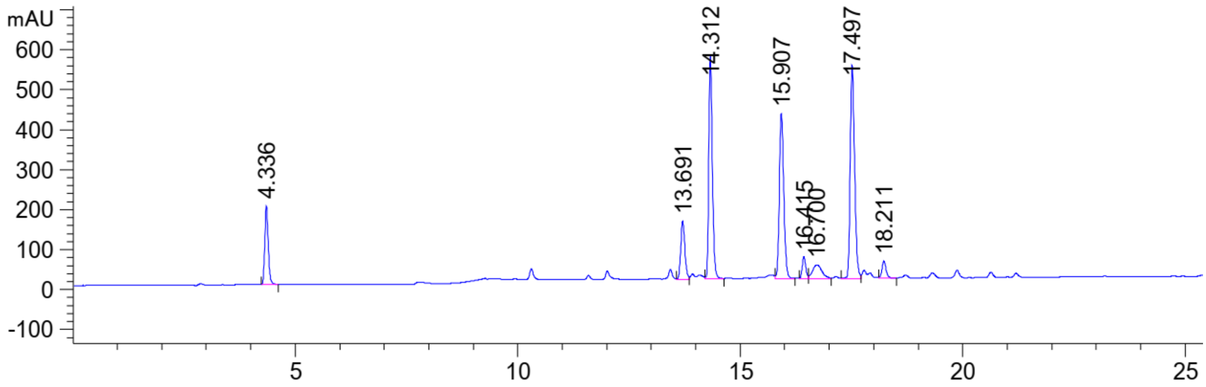

Figure S425: HPLC chromatogram of the synthesis of Smoc-L-Pro-L-Tyr-OMe **36** (17.4min) EDC-HCl **37**/NHS **38** in 30% aq. MeCN after 25min at λ=220 nm (0to40 MeCN).

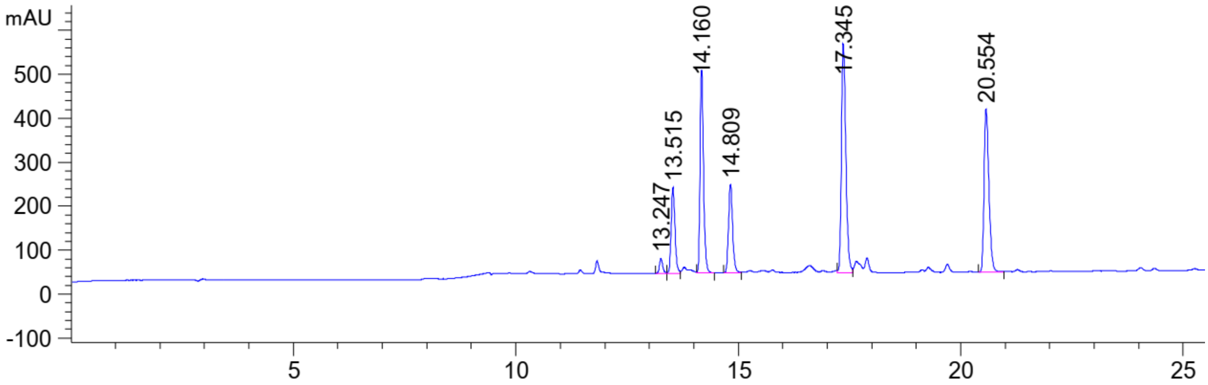

Figure S426: HPLC chromatogram of the synthesis of Smoc-L-Pro-L-Tyr-OMe **36** (17.2min) EDC-HCl **37**/HONB **41** in 30% aq. MeCN after 25min at λ=220 nm (0to40 MeCN).

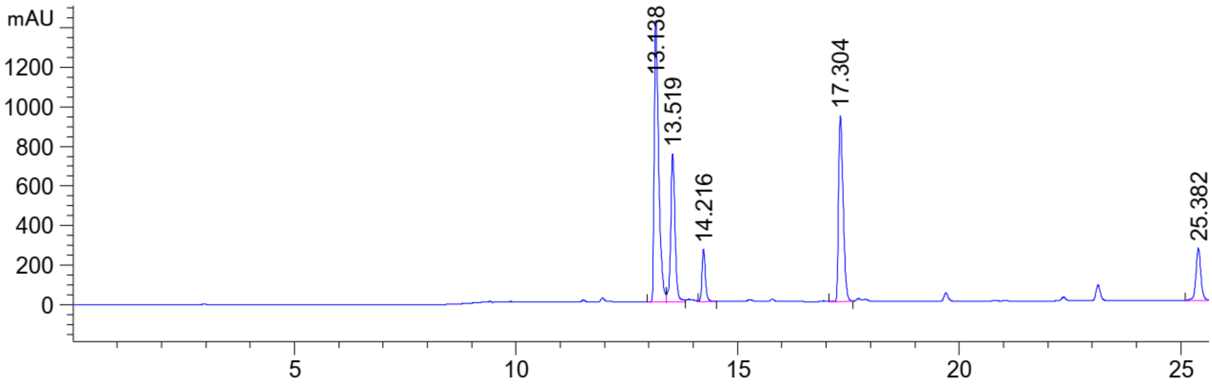

Figure S427: HPLC chromatogram of the synthesis of Smoc-L-Pro-L-Tyr-OMe **36** (17.2min) with EEDQ **42** in 30% aq. MeCN after 25min at λ=220 nm (0to40 MeCN).

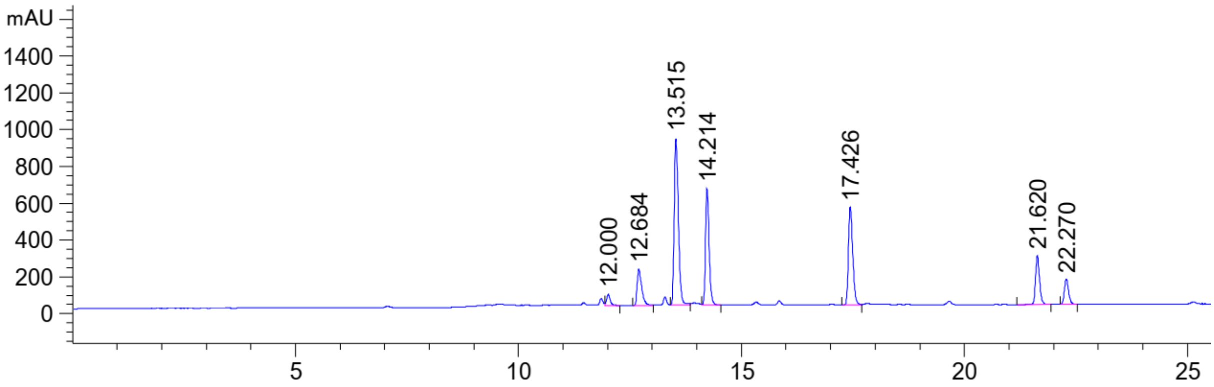

Figure S428: HPLC chromatogram of the synthesis of Smoc-L-Pro-L-Tyr-OMe **36** (17.2min) with DMT-MM **43** in 30% aq. MeCN after 25min at λ=220 nm (0to40 MeCN).

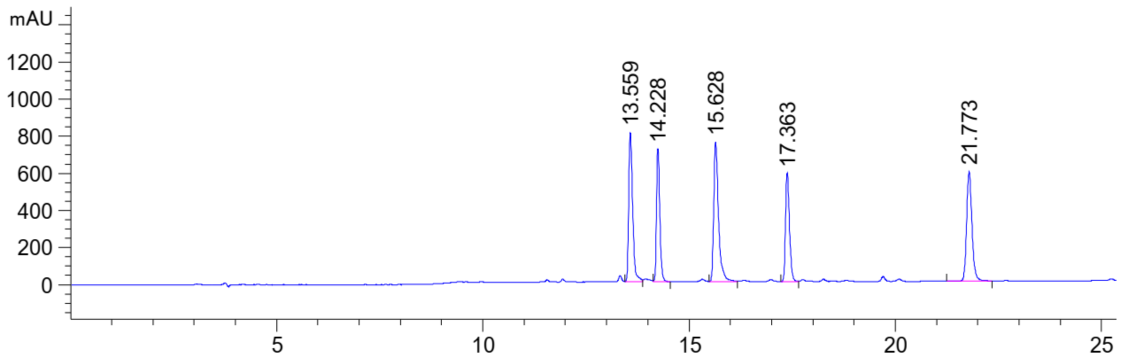

Figure S429: HPLC chromatogram of the synthesis of Smoc-L-Pro-L-Tyr-OMe **36** (17.2min) with COMU **44** in 30% aq. MeCN after 25min at λ=220 nm (0to40 MeCN).

3.5.5. Analytical data of the synthesis Smoc-Pro-Tyr-OMe 36 in 30% EtOAc water mixture (biphasic)

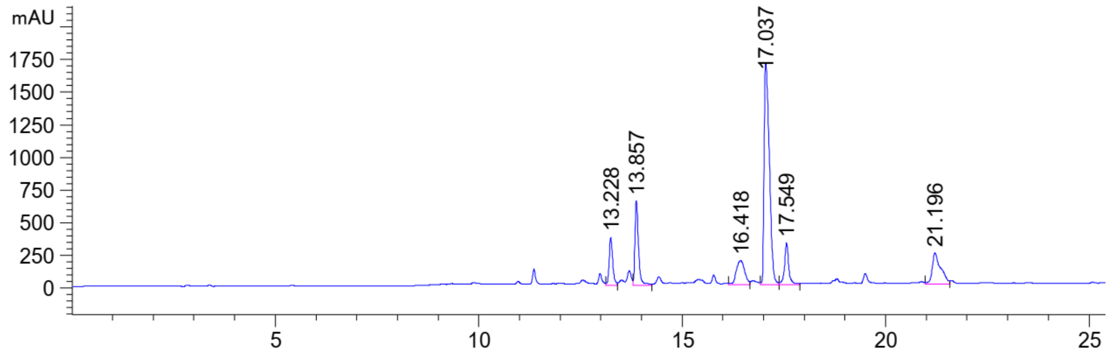

Figure S430: HPLC chromatogram of the synthesis of Smoc-L-Pro-L-Tyr-OMe **36** (17min) with EDC-HCl **37**/Oxyma **39** in 30% EtOAc water mixture (biphasic) after 25min at λ=220 nm (0to40 MeCN).

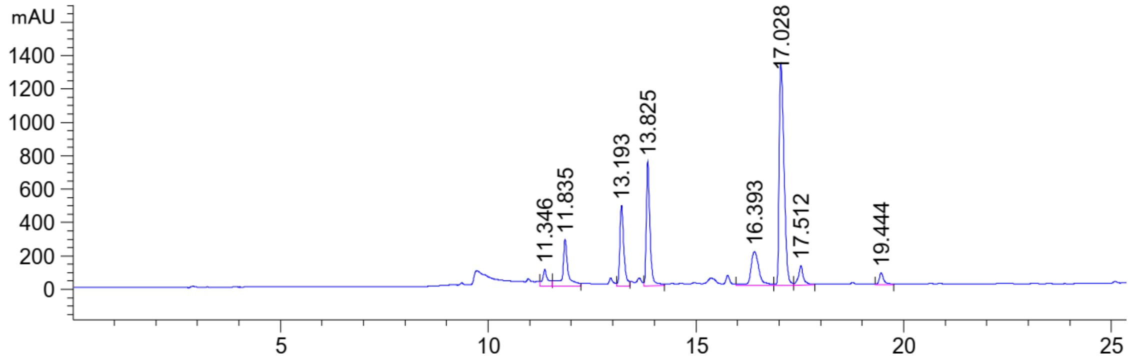

Figure S431: HPLC chromatogram of the synthesis of Smoc-L-Pro-L-Tyr-OMe **36** (17min) with EDC-HCl **37**/HOPO **40** in 30% EtOAc water mixture (biphasic) after 25min at λ=220 nm (0to40 MeCN).

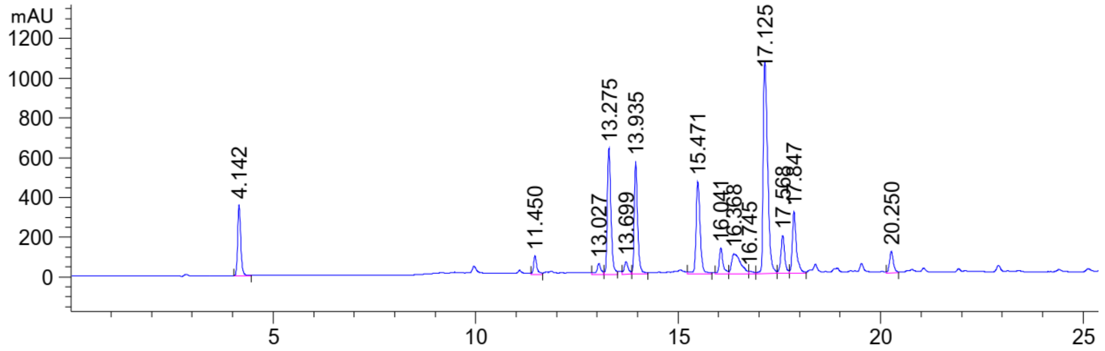

Figure S432: HPLC chromatogram of the synthesis of Smoc-L-Pro-L-Tyr-OMe **36** (17.1min) with EDC-HCl **37**/NHS **38** in 30% EtOAc water mixture (biphasic) after 25min at λ=220 nm (0to40 MeCN).

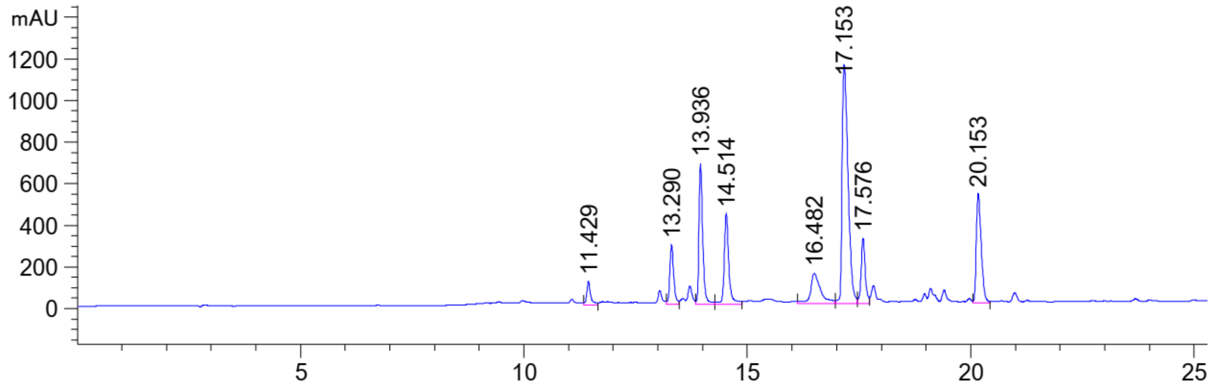

Figure S433: HPLC chromatogram of the synthesis of Smoc-L-Pro-L-Tyr-OMe **36** (17.2min) with EDC-HCl **37**/HONB **41** in 30% EtOAc water mixture (biphasic) after 25min at λ=220 nm (0to40 MeCN).

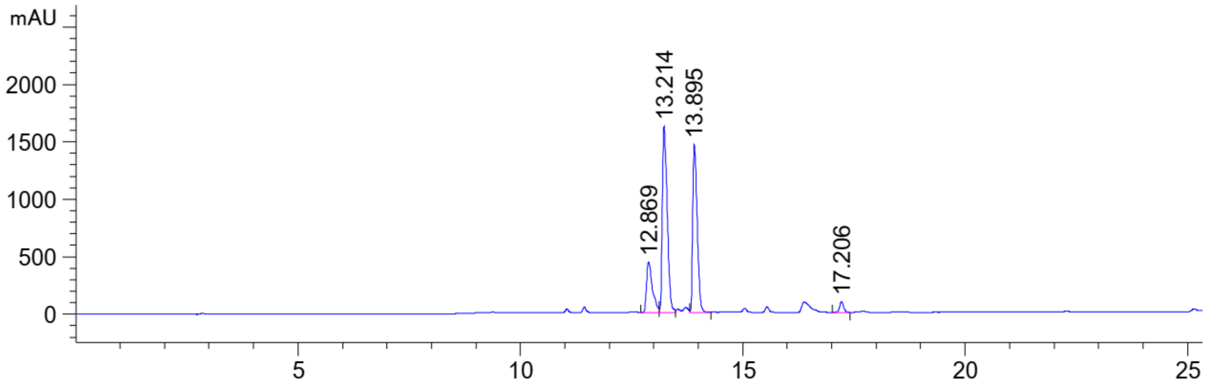

Figure S434: HPLC chromatogram of the synthesis of Smoc-L-Pro-L-Tyr-OMe **36** (17.2min) with EEDQ **42** in 30% EtOAc water mixture (biphasic) after 25min at λ=220 nm (0to40 MeCN).

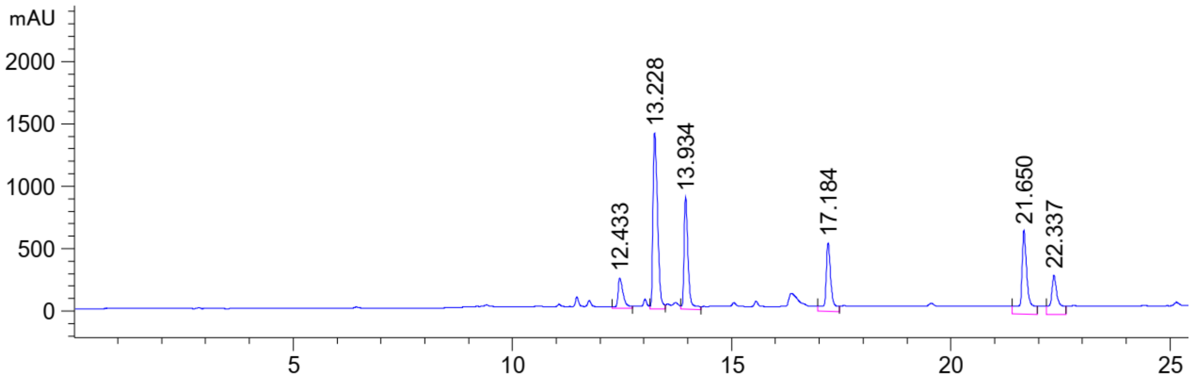

Figure S435: HPLC chromatogram of the synthesis of Smoc-L-Pro-L-Tyr-OMe 36 (17.2min) with DMT-MM **43** in 30% EtOAc water mixture (biphasic) after 25min at λ=220 nm (0to40 MeCN).

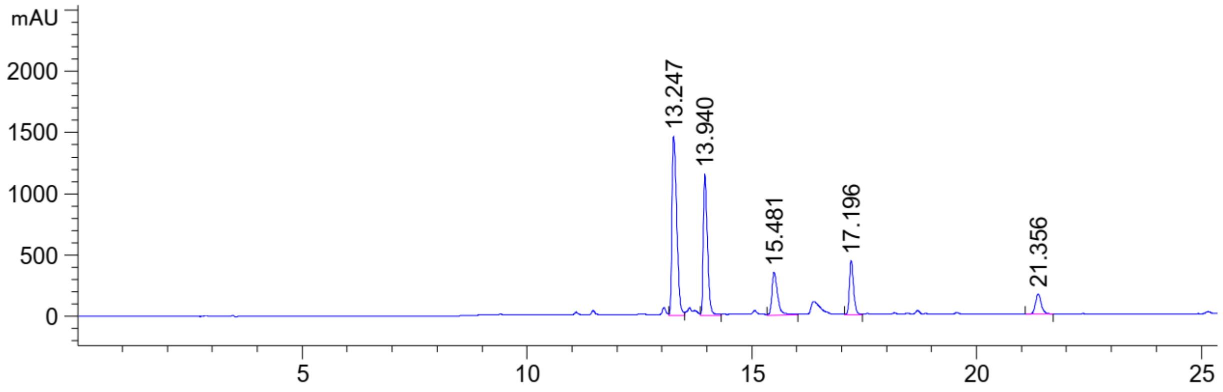

Figure S436: HPLC chromatogram of the synthesis of Smoc-L-Pro-L-Tyr-OMe **36**(17.2min) with COMU **44** in 30% EtOAc water mixture (biphasic) after 25min at λ=220 nm (0to40 MeCN).

3.5.6. Analytical data of the synthesis Smoc-Pro-Tyr-OMe 36 in 30% aq. ethanol

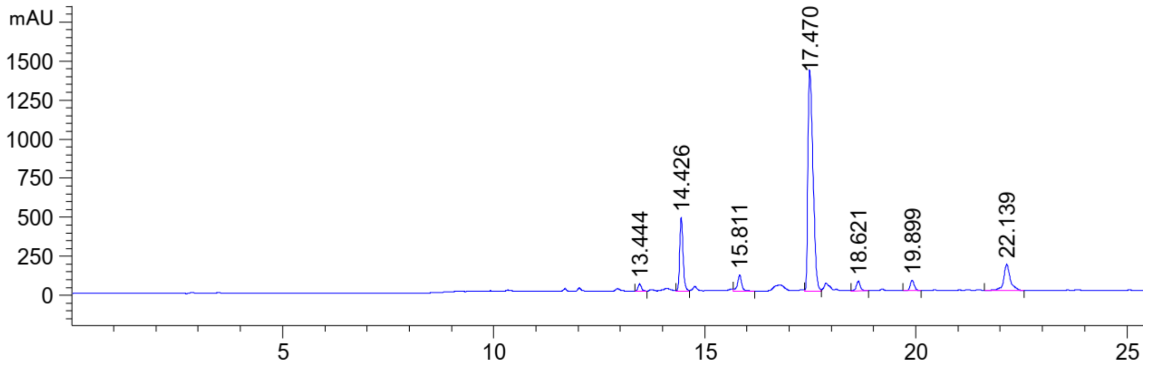

Figure S437: HPLC chromatogram of the synthesis of Smoc-L-Pro-L-Tyr-OMe **36** (17.5min) with EDC-HCl **37**/Oxyma **39** in 30% aq. ethanol after 25min at λ=220 nm (0to40 MeCN).

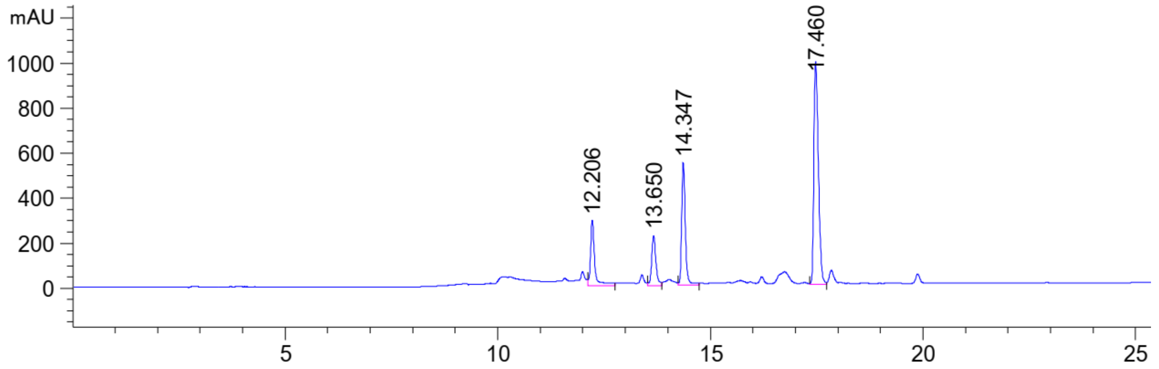

Figure S438: HPLC chromatogram of the synthesis of Smoc-L-Pro-L-Tyr-OMe **36** (17.5min) with EDC-HCl **37**/HOPO **40** in 30% aq. ethanol after 25min at λ=220 nm (0to40 MeCN).

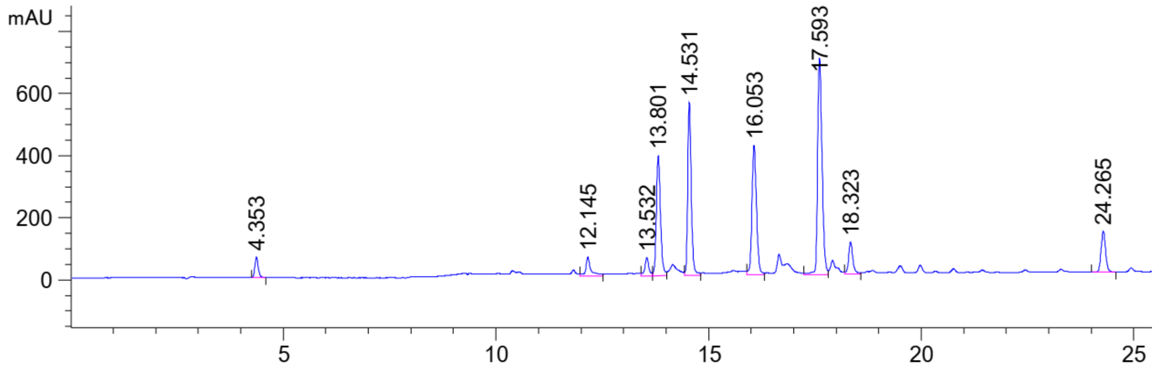

Figure S439: HPLC chromatogram of the synthesis of Smoc-L-Pro-L-Tyr-OMe **36** (17.6min) with EDC-HCl **37**/NHS **38** in 30% aq. ethanol after 25min at λ=220 nm (0to40 MeCN).

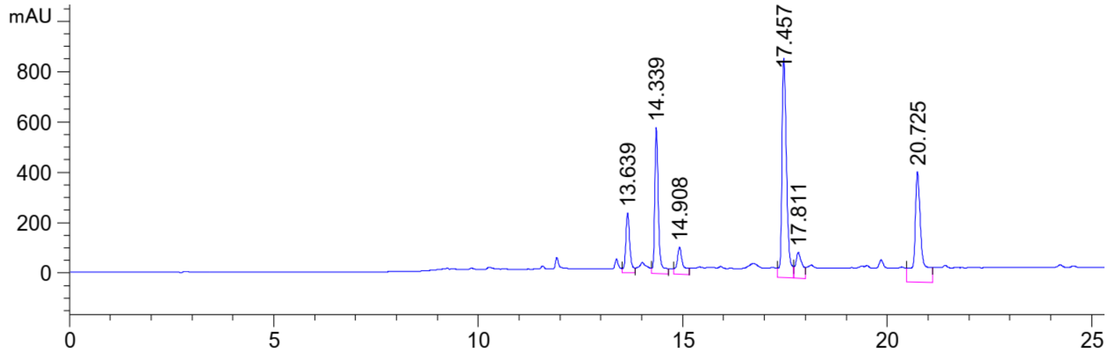

Figure S440: HPLC chromatogram of the synthesis of Smoc-L-Pro-L-Tyr-OMe **36** (17.5min) with EDC-HCl **37**/HONB **41** in 30% aq. ethanol after 25min at λ=220 nm (0to40 MeCN).

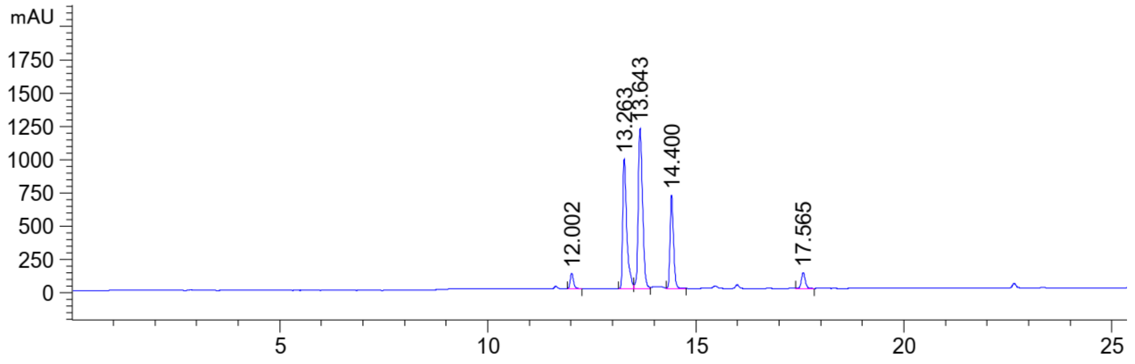

Figure S441: HPLC chromatogram of the synthesis of Smoc-L-Pro-L-Tyr-OMe **36** (17.5min) with EEDQ **42** in 30% aq. ethanol after 25min at λ=220 nm (0to40 MeCN).

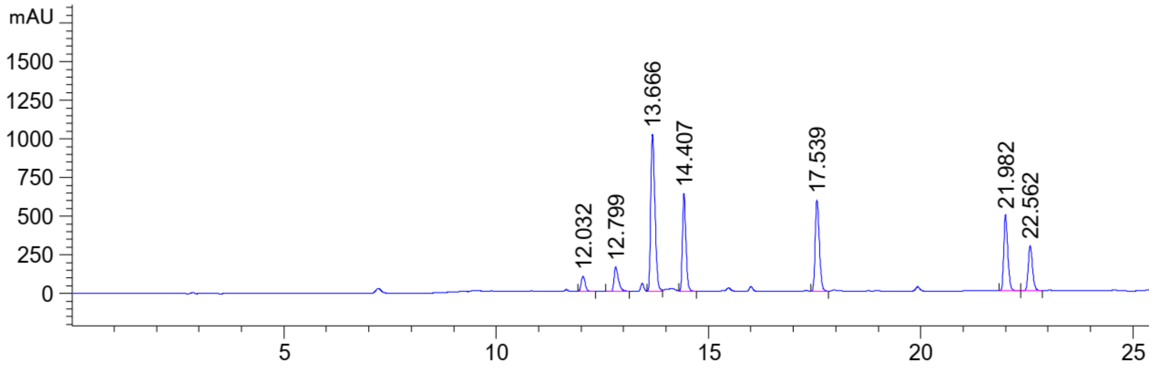

Figure S442: HPLC chromatogram of the synthesis of Smoc-L-Pro-L-Tyr-OMe **36** (17.5min) with DMT-MM **43** in 30% aq. ethanol after 25min at λ=220 nm (0to40 MeCN).

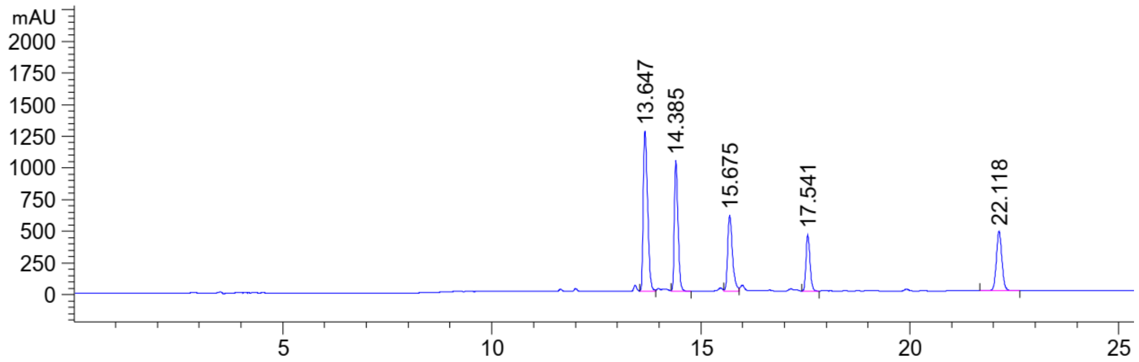

Figure S443: HPLC chromatogram of the synthesis of Smoc-L-Pro-L-Tyr-OMe **36** (17.5min) with COMU **44** in 30% aq. ethanol after 25min at λ=220 nm (0to40 MeCN).

3.5.7. Analytical data of the synthesis Smoc-Pro-Tyr-OMe 36 in 30% aq. isopropanol

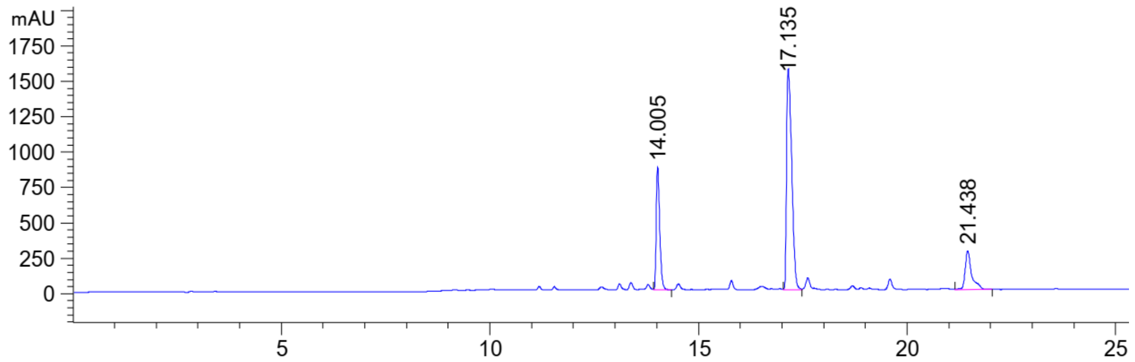

Figure S444: HPLC chromatogram of the synthesis of Smoc-L-Pro-L-Tyr-OMe **36** (17.2min) with EDC-HCl **37**/Oxyma **39** in 30% aq. isopropanol after 25min at λ=220 nm (0to40 MeCN).

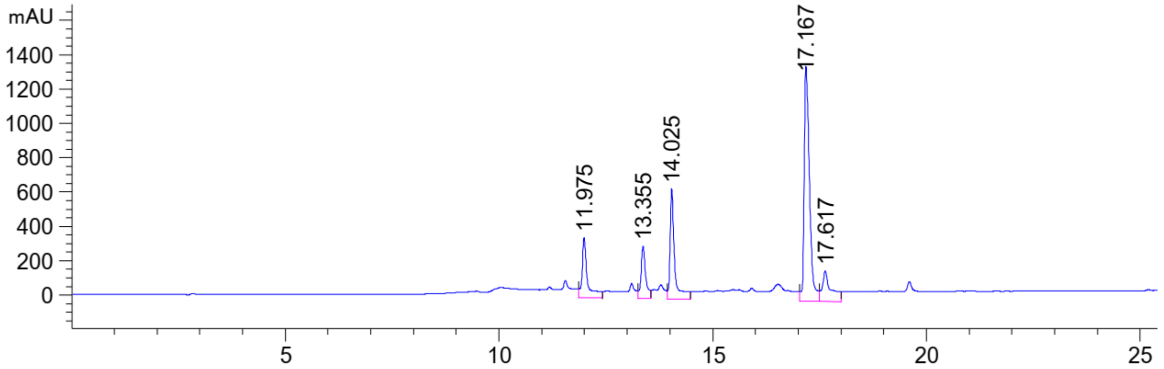

Figure S445: HPLC chromatogram of the synthesis of Smoc-L-Pro-L-Tyr-OMe **36** (17.2min) with EDC-HCl **37**/HOPO **40** in 30% aq. isopropanol after 25min at λ=220 nm (0to40 MeCN).

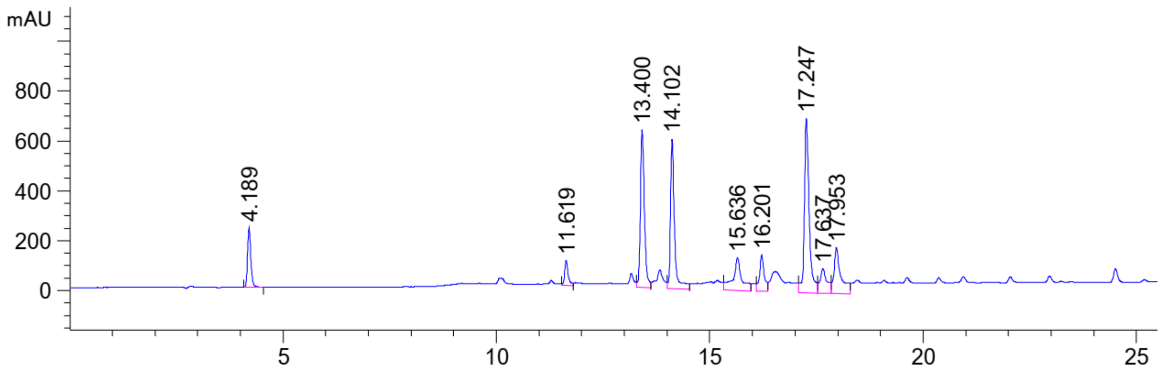

Figure S446: HPLC chromatogram of the synthesis of Smoc-L-Pro-L-Tyr-OMe **36** (17.2min) with EDC-HCl **37**/NHS **38** in 30% aq. isopropanol after 25min at λ=220 nm (0to40 MeCN).

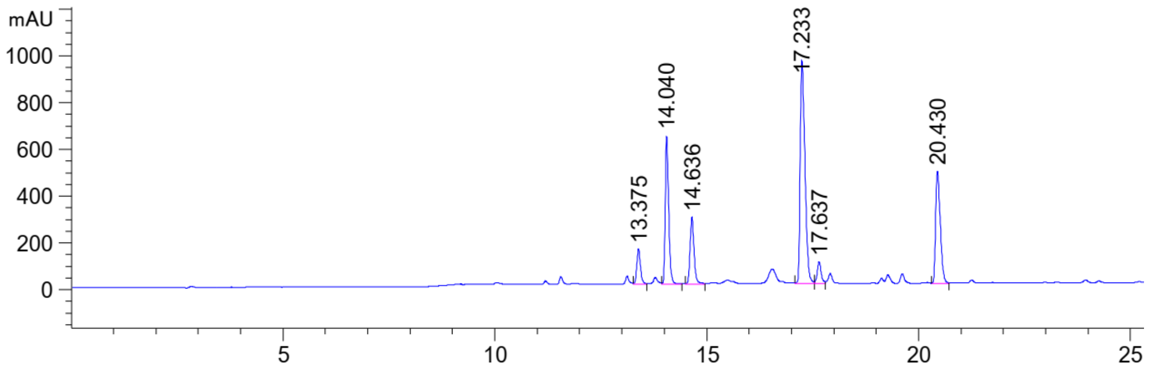

Figure S447: HPLC chromatogram of the synthesis of Smoc-L-Pro-L-Tyr-OMe **36** (17.2min) with EDC-HCl **37**/HONB **41** in 30% aq. isopropanol after 25min at λ=220 nm (0to40 MeCN).

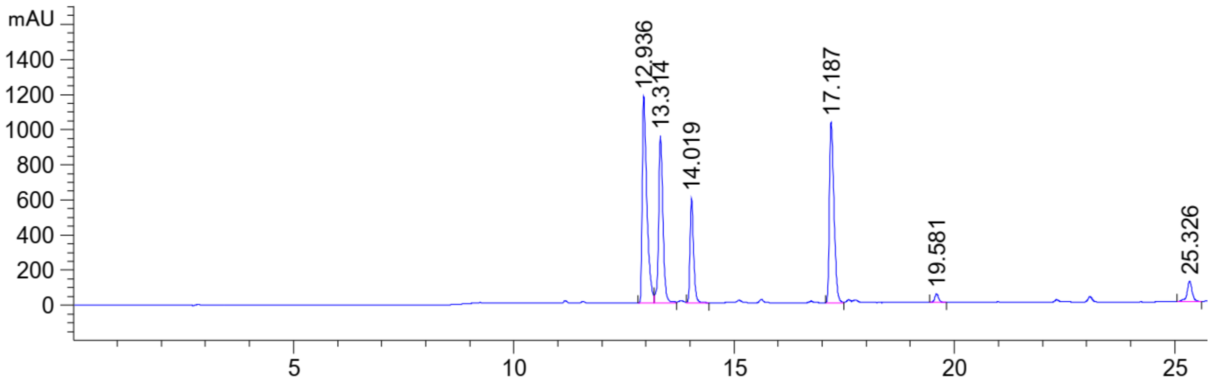

Figure S448: HPLC chromatogram of the synthesis of Smoc-L-Pro-L-Tyr-OMe **36** (17.2min) with EEDQ **42** in 30% aq. isopropanol after 25min at λ=220 nm (0to40 MeCN).

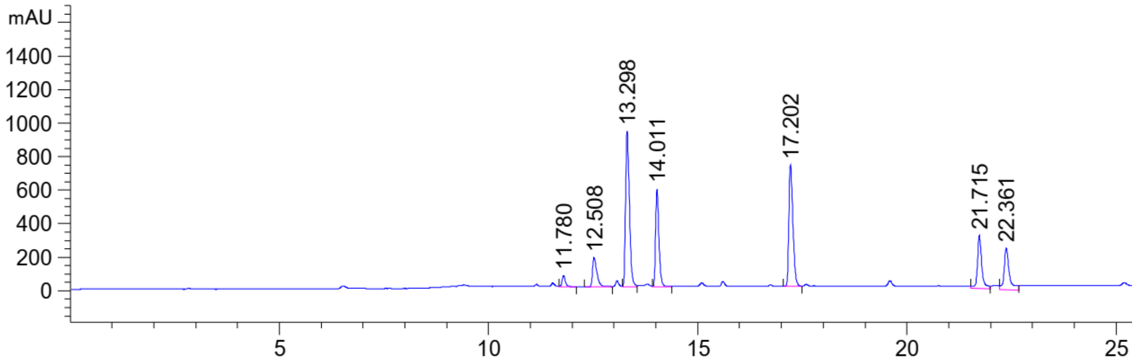

Figure S449: HPLC chromatogram of the synthesis of Smoc-L-Pro-L-Tyr-OMe **36** (17.2min) with DMT-MM **43** in 30% aq. isopropanol after 25min at λ=220 nm (0to40 MeCN).

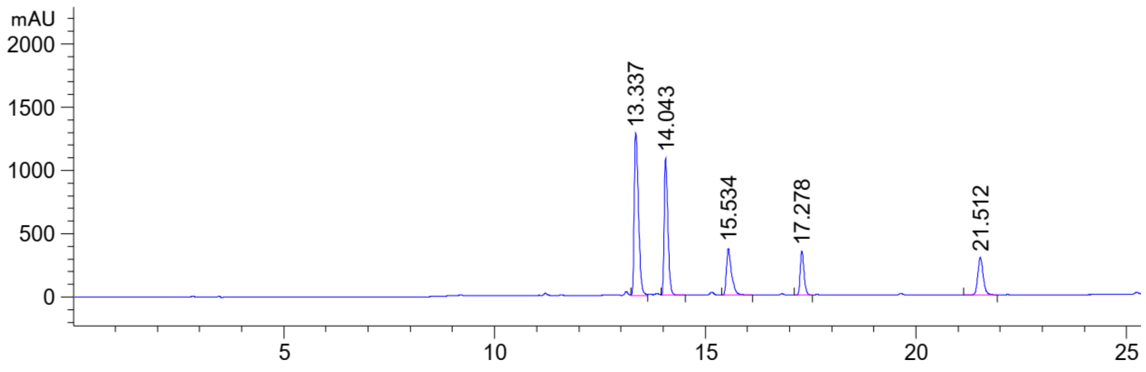

Figure S450: HPLC chromatogram of the synthesis of Smoc-L-Pro-L-Tyr-OMe **36** (17.2min) with COMU **44** in 30% aq. isopropanol after 25min at λ=220 nm (0to40 MeCN).

3.5.8. Analytical data of the synthesis Smoc-Pro-Tyr-OMe 36 in 30% MeTHF water mixture (biphasic)

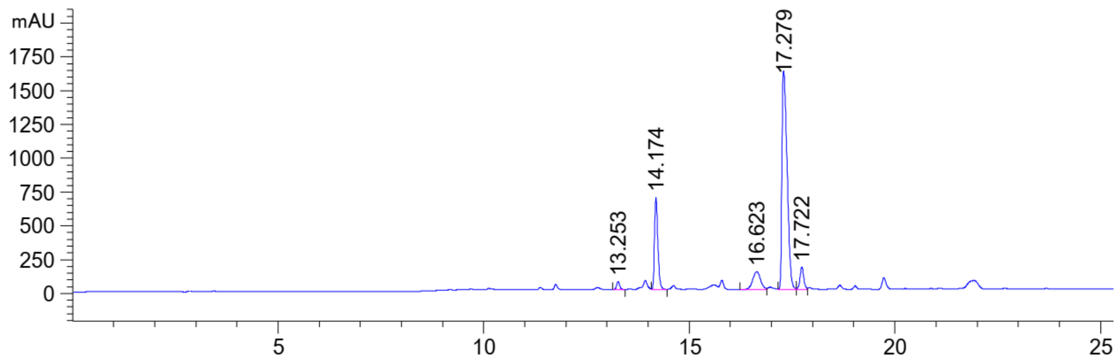

Figure S451: HPLC chromatogram of the synthesis of Smoc-L-Pro-L-Tyr-OMe **36** (17.3min) with EDC-HCl **37**/Oxyma **39** in 30% Me-THF water mixture (biphasic) after 25min at λ=220 nm (0to40 MeCN).

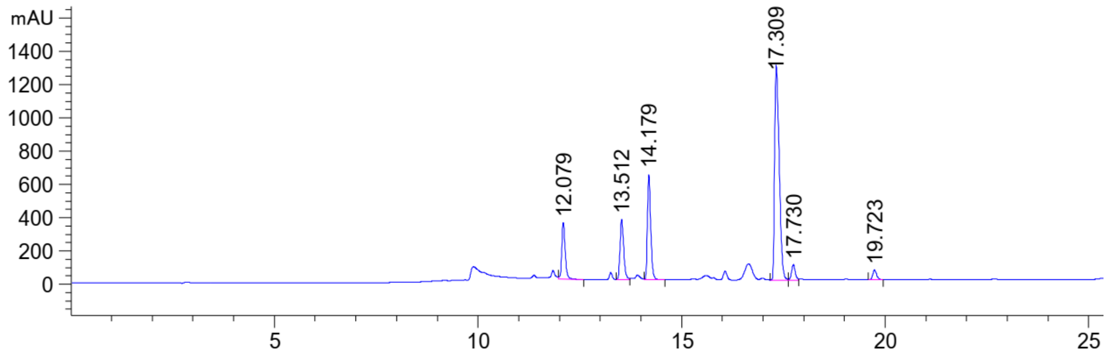

Figure S452: HPLC chromatogram of the synthesis of Smoc-L-Pro-L-Tyr-OMe **36** (17.3min) with EDC-HCl **37**/HOPO **40** in 30% Me-THF water mixture (biphasic) after 25min at λ=220 nm (0to40 MeCN).

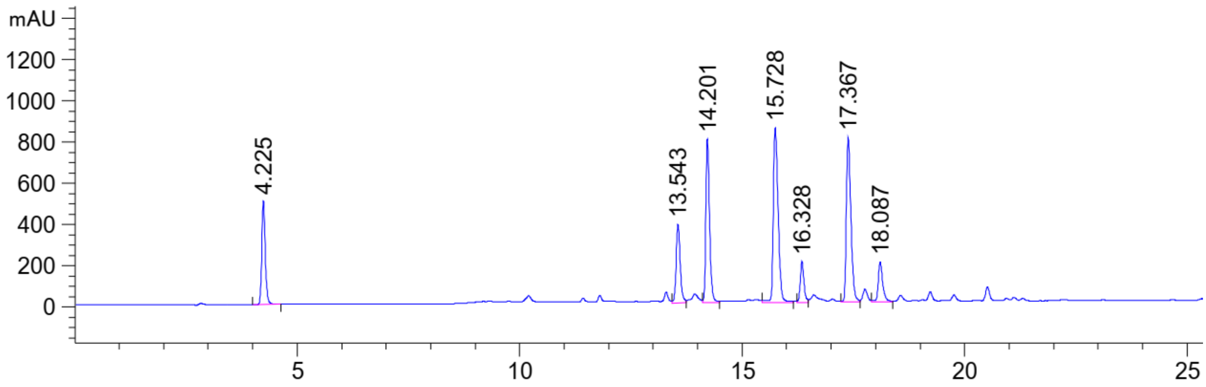

Figure S453: HPLC chromatogram of the synthesis of Smoc-L-Pro-L-Tyr-OMe **36** (17.4min) with EDC-HCl **37**/NHS **38** in 30% Me-THF water mixture (biphasic) after 25min at λ=220 nm (0to40 MeCN).

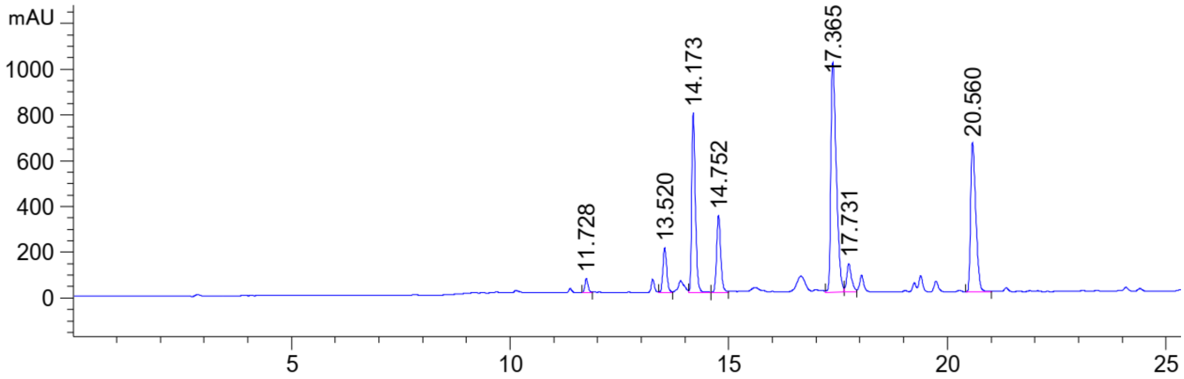

Figure S454: HPLC chromatogram of the synthesis of Smoc-L-Pro-L-Tyr-OMe **36** (17.4min) EDC-HCl **37**/HONB **41** in 30% Me-THF water mixture (biphasic) after 25min at λ=220 nm (0to40 MeCN).

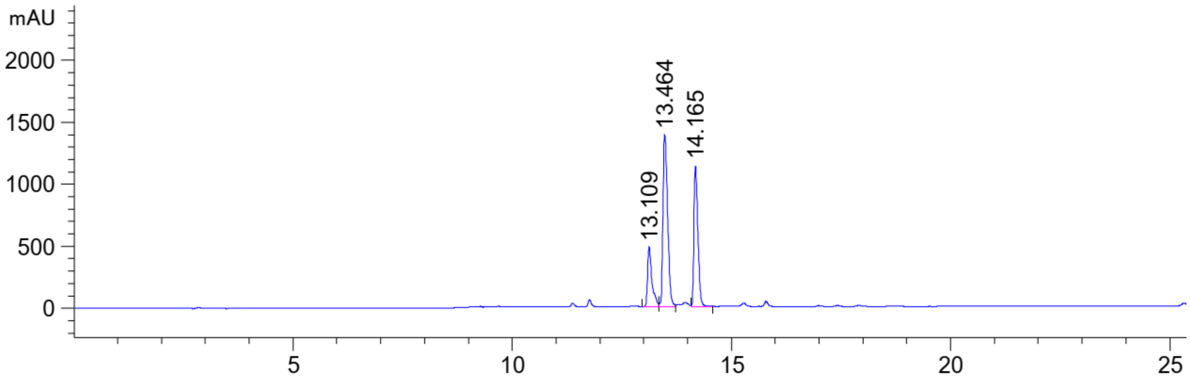

Figure S455: HPLC chromatogram of the synthesis of Smoc-L-Pro-L-Tyr-OMe **36** (17.4min) with EEDQ **42** in 30% Me-THF water mixture (biphasic) after 25min at λ=220 nm (0to40 MeCN).

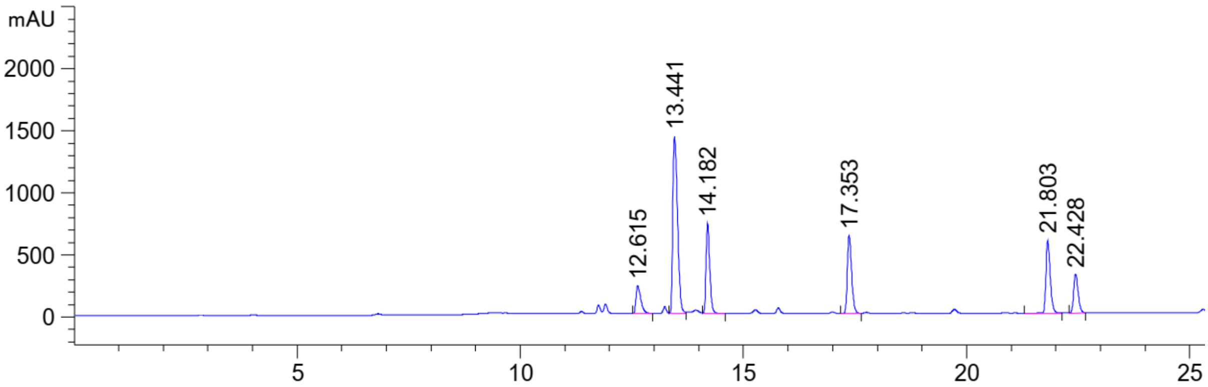

Figure S456: HPLC chromatogram of the synthesis of Smoc-L-Pro-L-Tyr-OMe **36** (17.4min) with DMT-MM **43** in 30% Me-THF water mixture (biphasic) after 25min at λ=220 nm (0to40 MeCN).

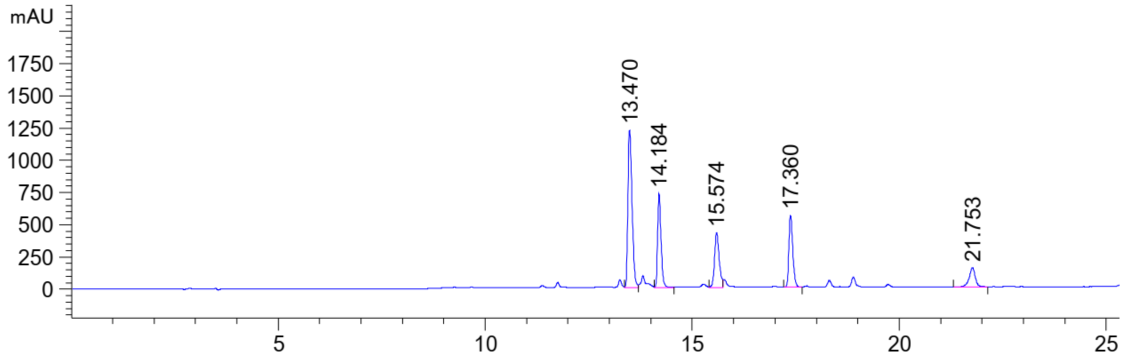

Figure S457: HPLC chromatogram of the synthesis of Smoc-L-Pro-L-Tyr-OMe **36** (17.4min) with COMU **44** in 30% Me-THF water mixture (biphasic) after 25min at λ=220 nm (0to40 MeCN).

3.5.9. Analytical data of the synthesis Smoc-Pro-Tyr-OMe 36 in 10% aq. Me-THF

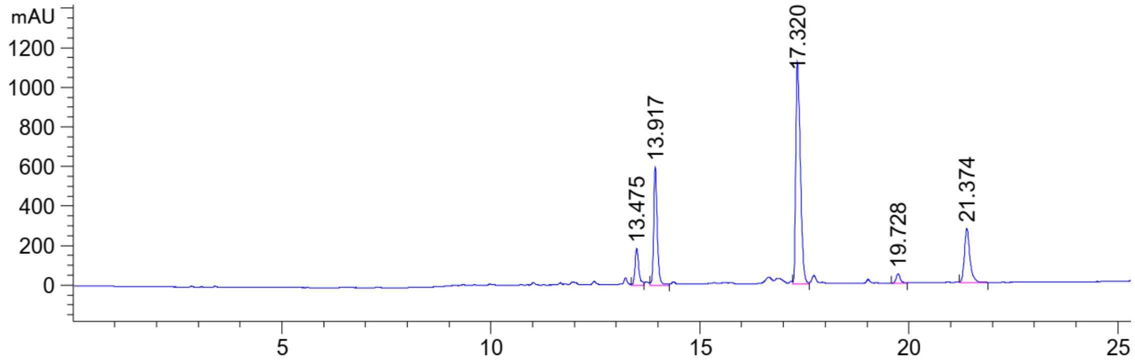

Figure S458: HPLC chromatogram of the synthesis of Smoc-L-Pro-L-Tyr-OMe **36** (17.3min) with EDC-HCl **37**/Oxyma **39** in 10% aq. Me-THF after 25min at λ=220 nm (0to40 MeCN).

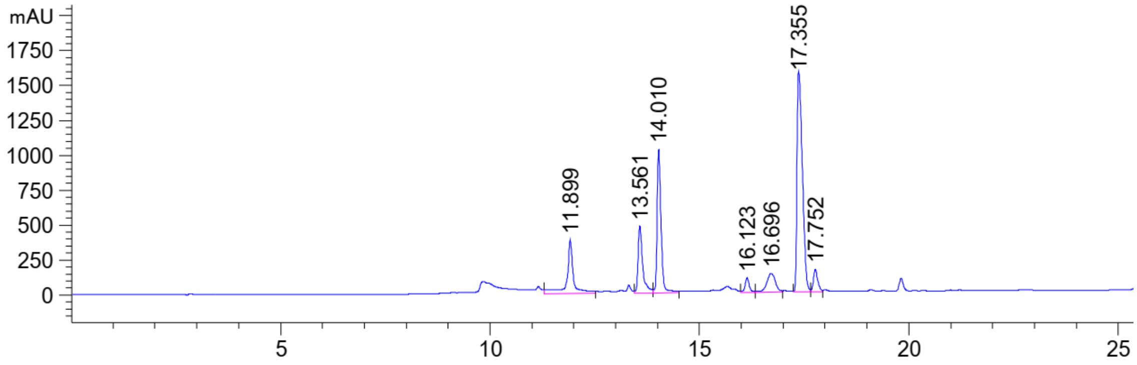

Figure S459: HPLC chromatogram of the synthesis of Smoc-L-Pro-L-Tyr-OMe **36** (17.3min) with EDC-HCl **37**/HOPO **40** in 10% aq. Me-THF after 25min at λ=220 nm (0to40 MeCN).

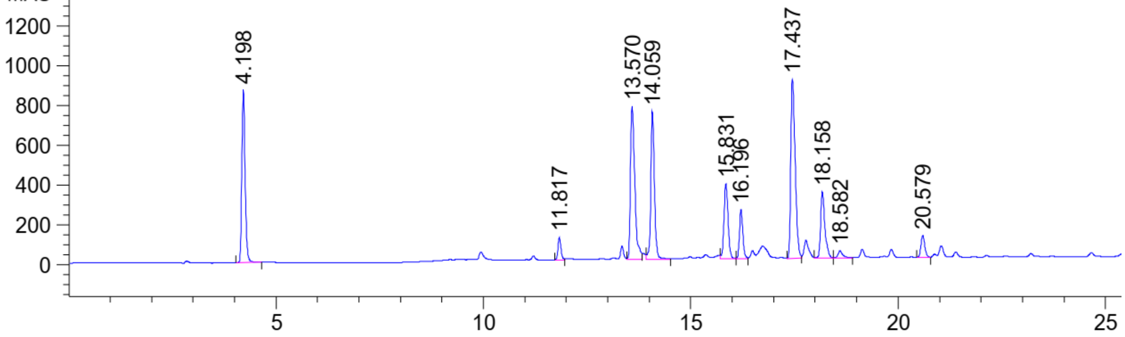

Figure S460: HPLC chromatogram of the synthesis of Smoc-L-Pro-L-Tyr-OMe **36** (17.3min) with EDC-HCl **37**/NHS **38** in 10% aq. Me-THF after 25min at λ=220 nm (0to40 MeCN).

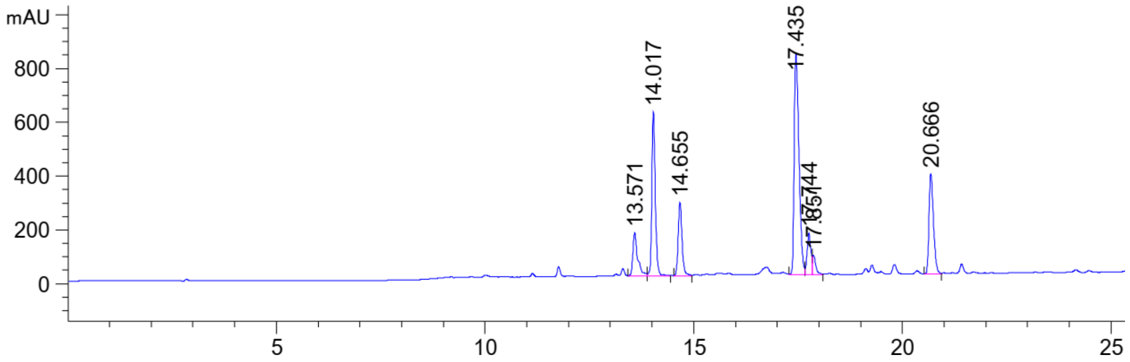

Figure S461: HPLC chromatogram of the synthesis of Smoc-L-Pro-L-Tyr-OMe **36** (17.3min) with EDC-HCl **37**/HONB **41** in 10% aq. Me-THF after 25min at λ=220 nm (0to40 MeCN).

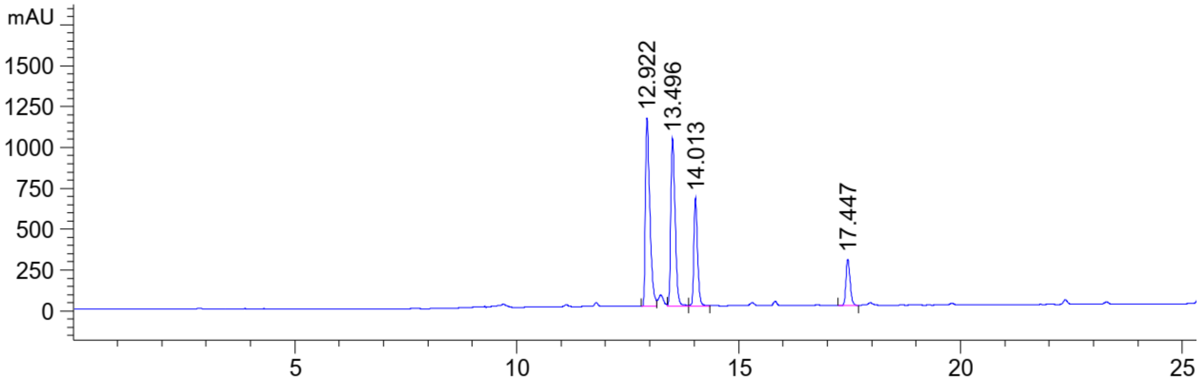

Figure S462: HPLC chromatogram of the synthesis of Smoc-L-Pro-L-Tyr-OMe **36** (17.4min) with EEDQ **42** in 10% aq. Me-THF after 25min at λ=220 nm (0to40 MeCN).

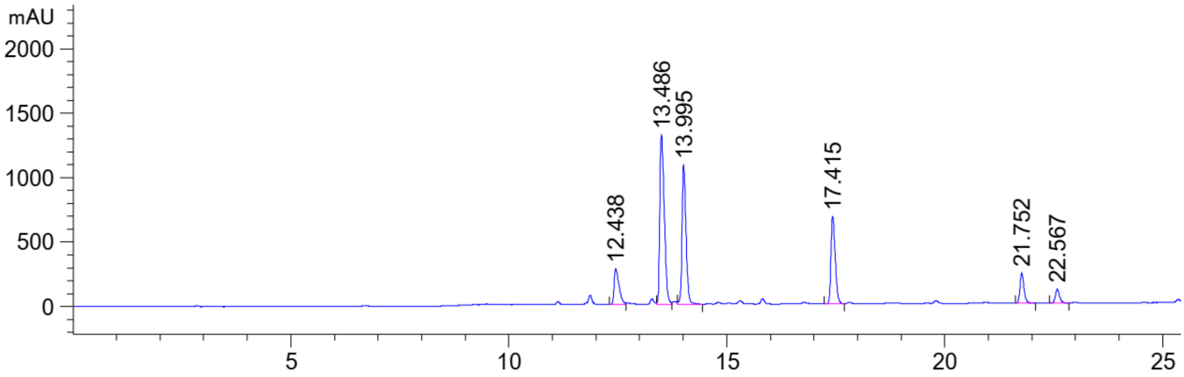

Figure S463: HPLC chromatogram of the synthesis of Smoc-L-Pro-L-Tyr-OMe **36** (17.4min) with DMT-MM **43** in 10% aq. Me-THF after 25min at λ=220 nm (0to40 MeCN).

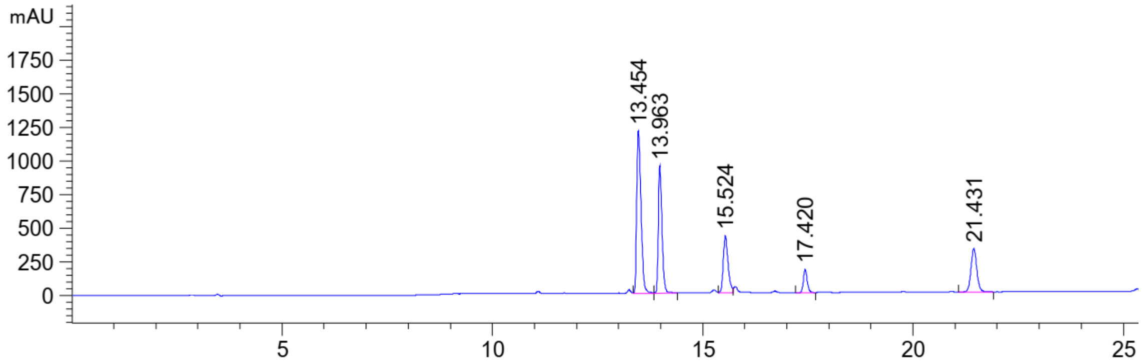

Figure S464: HPLC chromatogram of the synthesis of Smoc-L-Pro-L-Tyr-OMe **36** (17.4min) with COMU **44** in 10% aq. Me-THF after 25min at λ=220 nm (0to40 MeCN).

3.6. Analytical data of SPPS coupling efficiency test with Oxyma 39 and HOPO 40

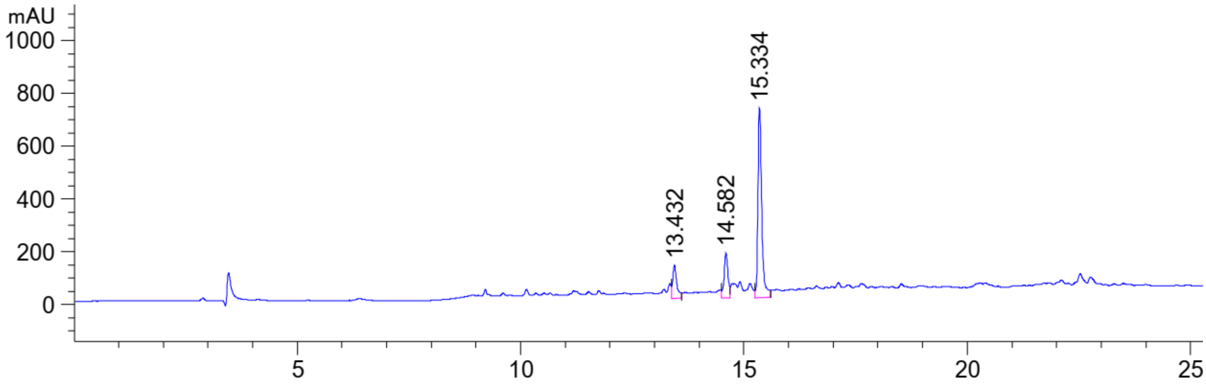

Figure S465: HPLC chromatogram of the synthesis of Smoc-LAGV-NH_2 **47** with EDC-HCl **37** and Oxyma **39** in water at λ=220 nm (0to60 MeCN).

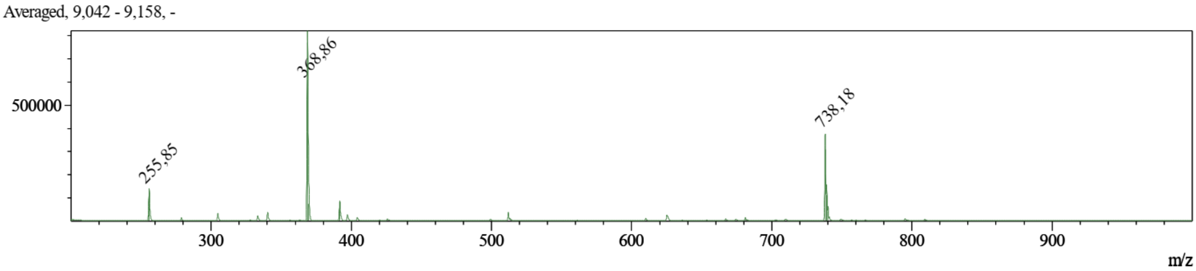

Figure S466: ESI-MS HPLC of the synthesis of Smoc-LAGV-NH2 **47** with EDC-HCl **37** and Oxyma **39** in water (M measured=738.18 $[M-2H]^-$, M calc.=740.80).

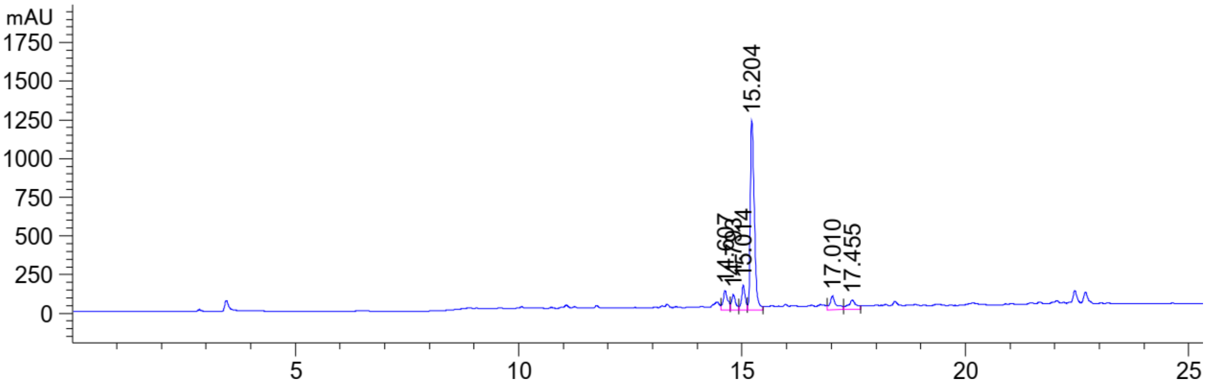

Figure S467: HPLC chromatogram of the synthesis of Smoc-LAGV-NH_2 **47** with EDC-HCl **37** and Oxyma **39** in 30% aq. MeCN at λ=220 nm (0to60 MeCN).

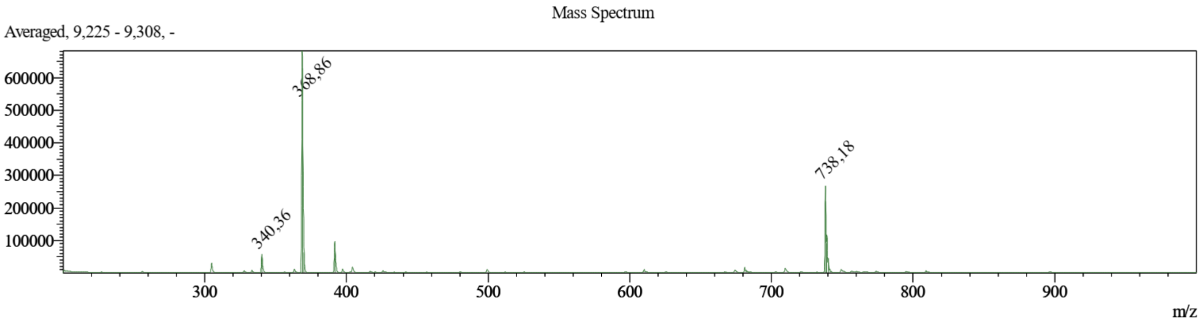

Figure S468: ESI-MS HPLC of the synthesis of Smoc-LAGV-NH_2 **47** with EDC-HCl **37** and Oxyma **39** in 30% aq. MeCN (M measured=738.18 $[M-H]^-$, M calc.=740.80).

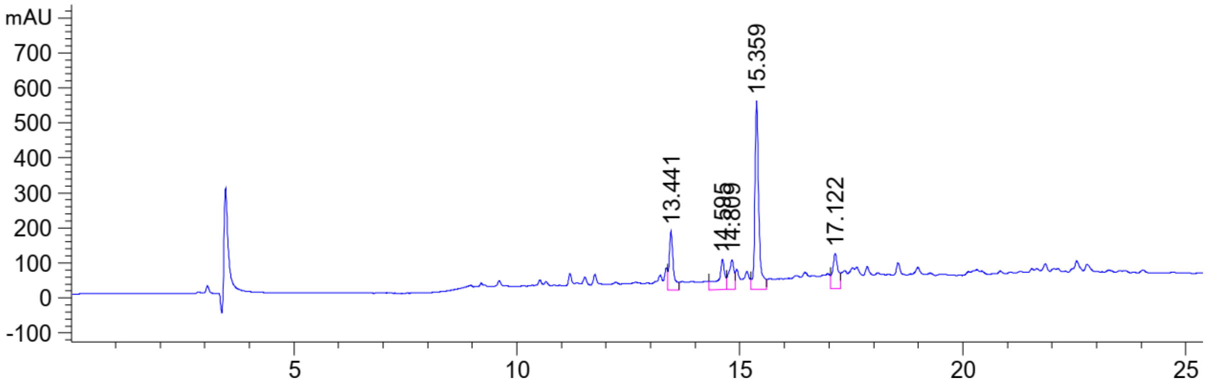

Figure S469: HPLC chromatogram of the synthesis of Smoc-LAGV-NH_2 **47** with EDC-HCl **37** and HOPO **40** in water at λ=220 nm (0to60 MeCN).

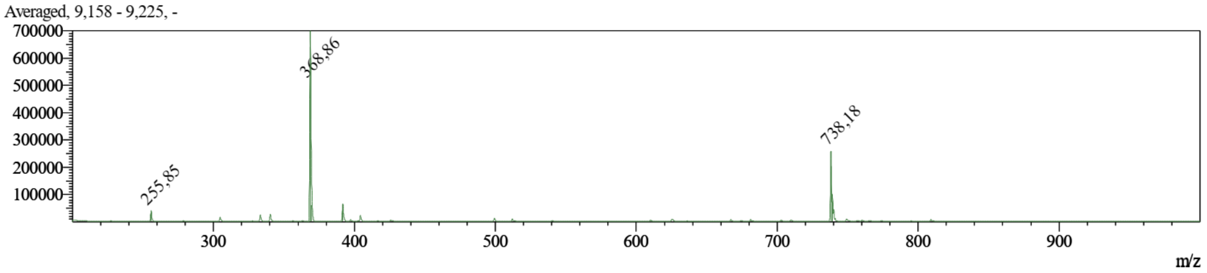

Figure S470: ESI-MS HPLC of the synthesis of Smoc-LAGV-NH_2 **47** with EDC-HCl **37** and HOPO **40** in water (M measured=738.18 $[M-H]^-$, M calc.=740.80).

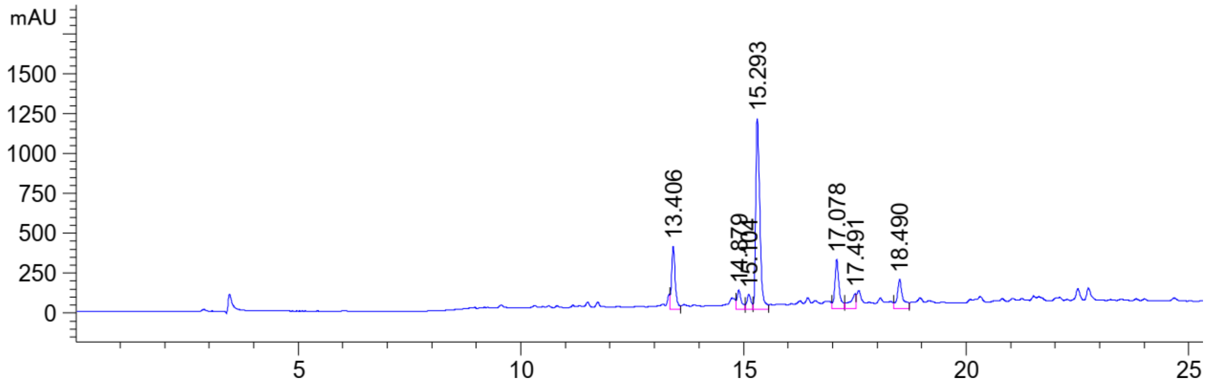

Figure S471: HPLC chromatogram of the synthesis of Smoc-LAGV-NH_2 **47** with EDC-HCl **37** and HOPO **40** in 30% aq. MeCN at λ=220 nm (0to60 MeCN).

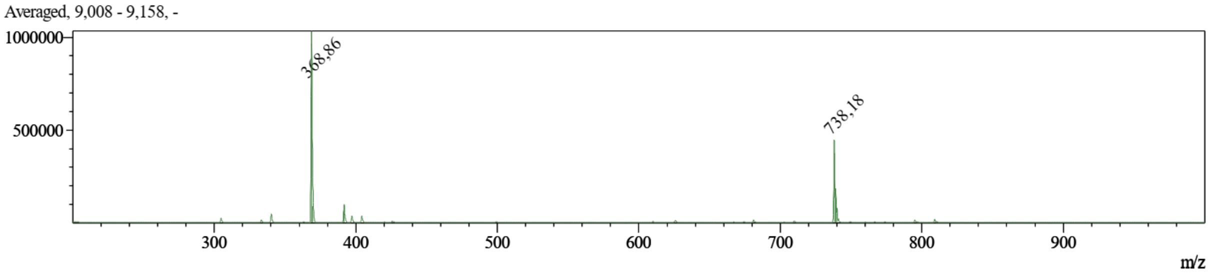

Figure S472: ESI-MS HPLC of the synthesis of Smoc-LAGV-NH_2 **47** with EDC-HCl **37** and HOPO **40** in 30% aq. MeCN (M measured=738.18 $[M-H]^-$, M calc.=740.80).

3.7. Peptides

3.7.1. Analytical data of H-AGELS-NH2 (Pentapeptide-31) 48

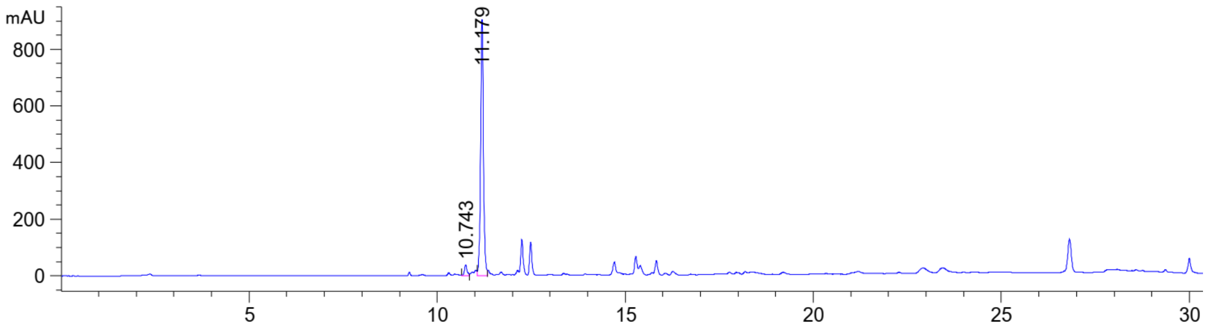

Figure S473: HPLC chromatogram of H-AGELS-NH$_2$ (Pentapeptide-31) **48** at λ=220 nm (0to60 MeCN).

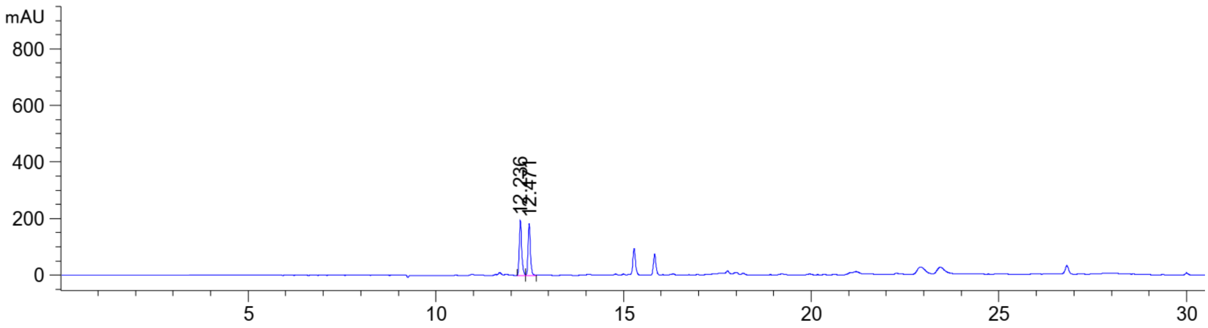

Figure S474: HPLC chromatogram of H-AGELS-NH$_2$ (Pentapeptide-31) **48** at λ=280 nm (0to60 MeCN).

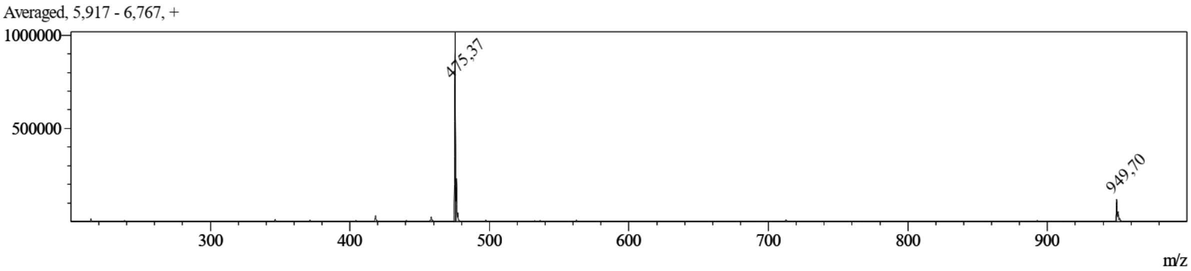

Figure S475: ESI-MS of H-AGELS-NH$_2$ (Pentapeptide-31) **48** (M measured=475.37 $[M+H]^+$, M calc.=474.52).

3.7.2. Analytical data of H-GPQGPQ-OH (Hexapeptide-9) 49

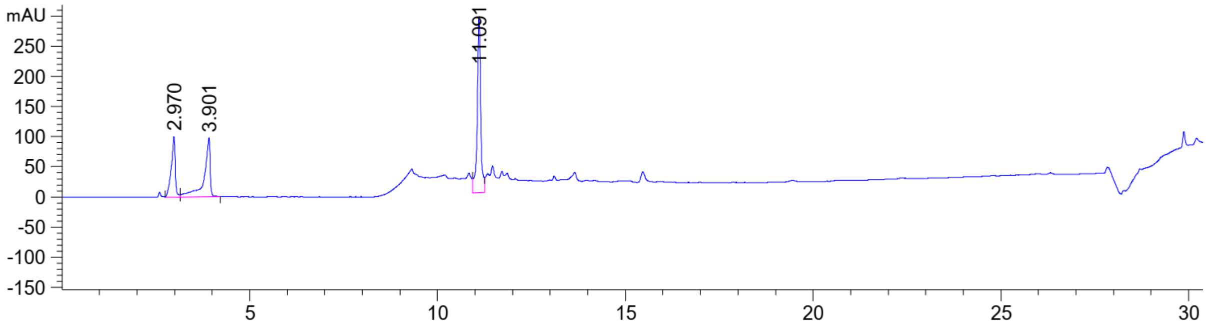

Figure S476: HPLC chromatogram of H-GPQGPQ-OH (Hexapeptide-9) **49** at λ=220 nm (0to60 MeCN).

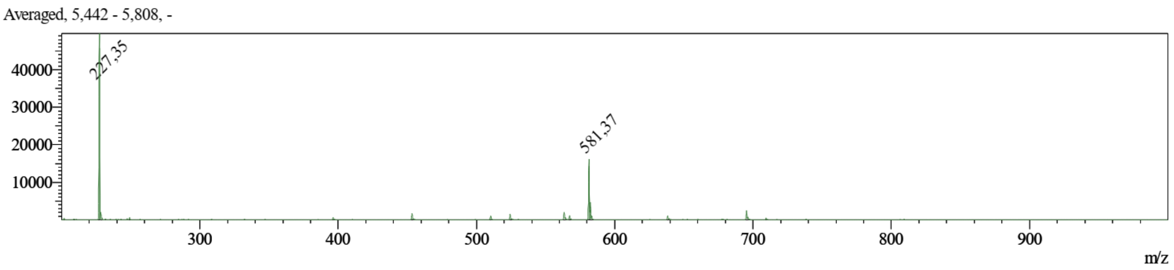

Figure S477: ESI-MS of H-GPQGPQ-OH (Hexapeptide-9) **49** (M measured=581.37 [M-H]$^-$, M calc.=581.37).

3.7.3. Analytical data of H-EEMQRR-NH2 (Hexapeptide 3) 50

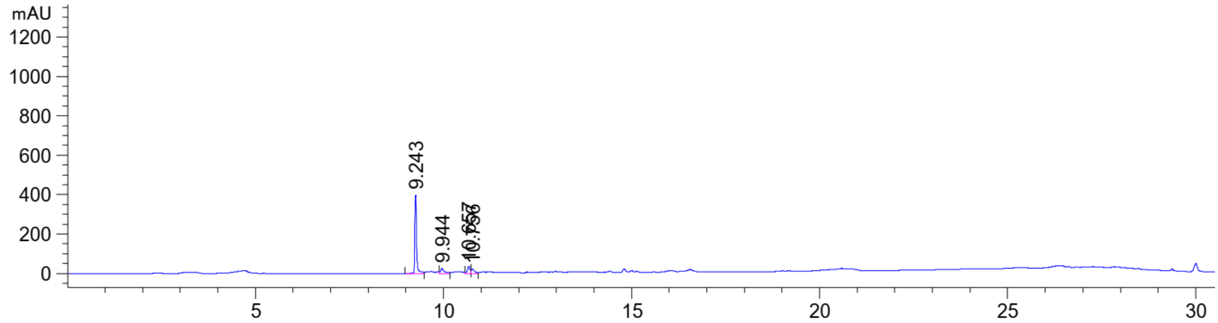

Figure S478: HPLC chromatogram of H-EEMQRR-NH$_2$ (Hexapeptide 3) **50** at λ=220 nm (0to60 MeCN).

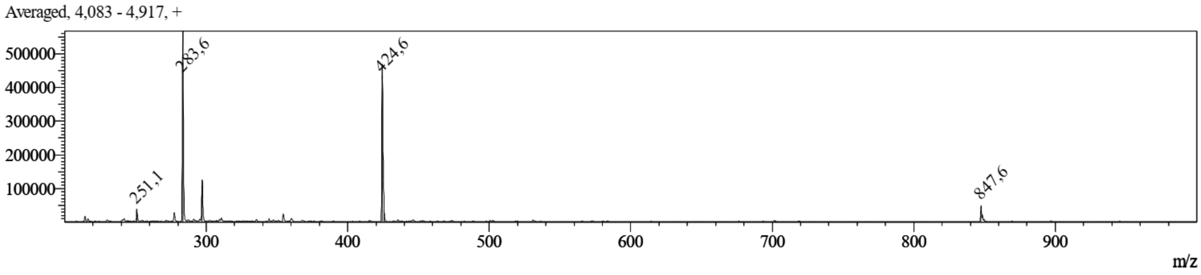

Figure S479: ESI-MS of H-EEMQRR-NH$_2$ (Hexapeptide 3) **50** (M measured=847.60 [M+H]$^+$, M calc.=846.96).

3.7.4. Analytical data of Ac-EEMQRR-NH2 (Acetyl-Hexapeptide 3) 51

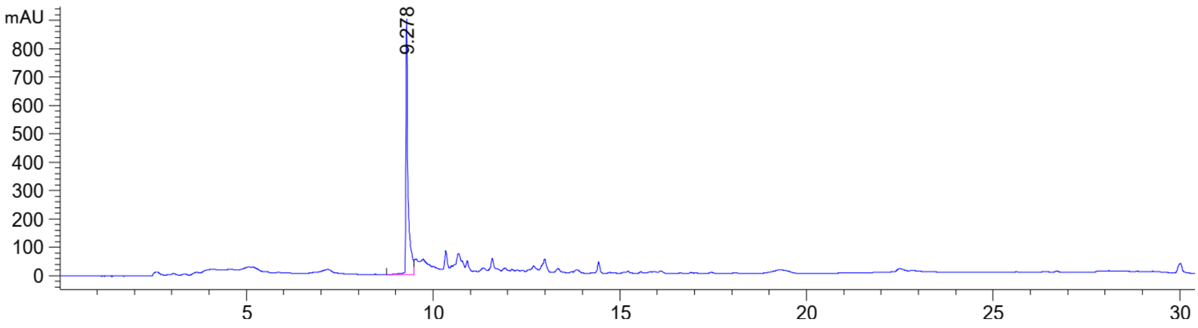

Figure S480: HPLC chromatogram of Ac-EEMQRR-NH$_2$ **51** at λ=220 nm (0to60 MeCN).

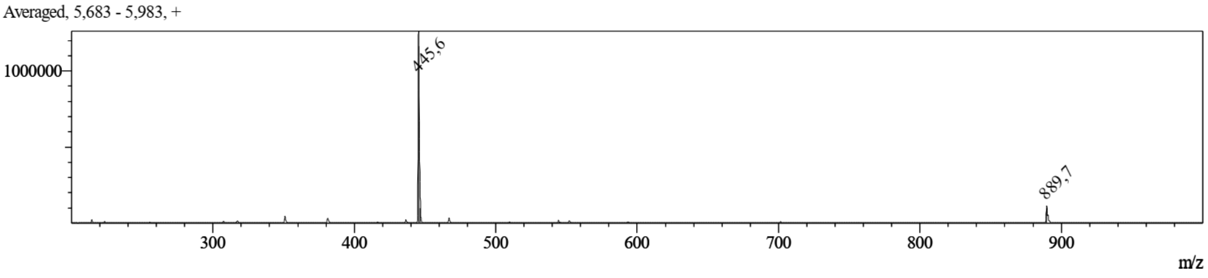

Figure S481: ESI-MS of Ac-EEMQRR-NH$_2$ **51** (M measured=889.70 [M+H]$^+$, M calc.=889.00).

3.7.5. Analytical data of Synthesis of Leu-Enkephalin amide 52

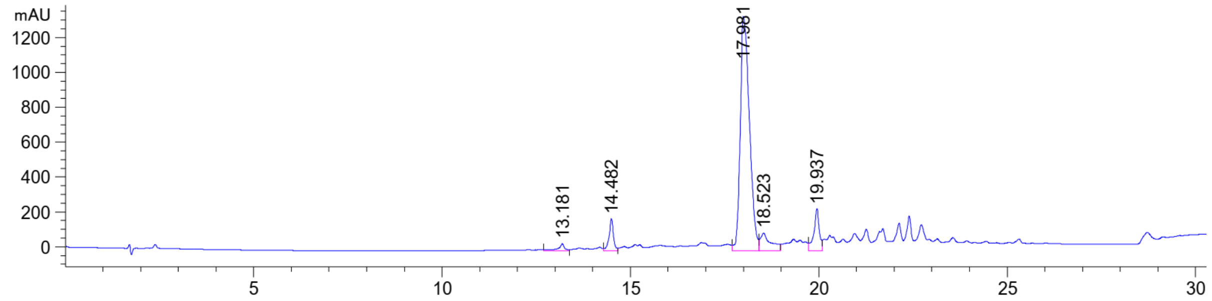

Figure S482: HPLC chromatogram of Synthesis of Leu-Enkephalin amide **52** at λ=220 nm (0to60 MeCN).

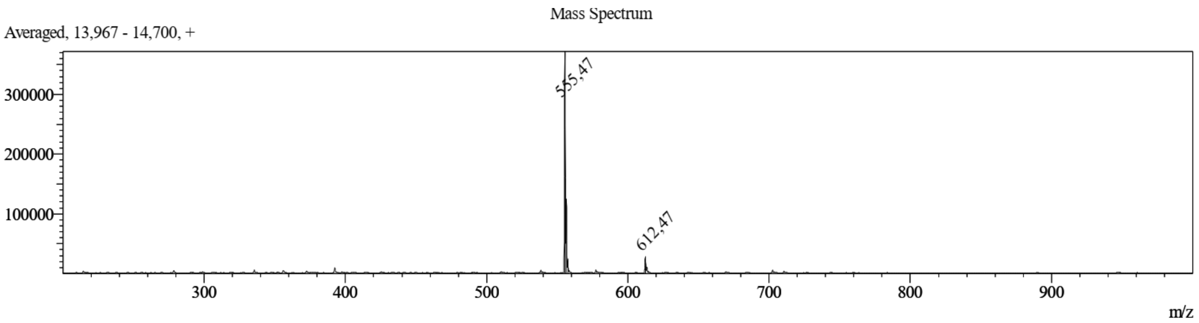

Figure S483: ESI-MS of Synthesis of Leu-Enkephalin amide **52** (M measured=554.65 $[M+H]^+$, M calc.=554.65).

3.7.6. Analytical data of Synthesis of Met-Enkephalin 53

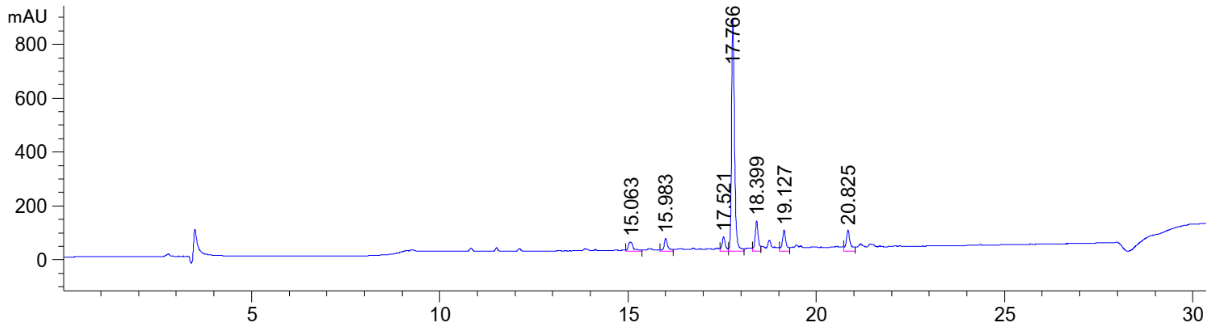

Figure S484: HPLC chromatogram of Synthesis of Met-Enkephalin **53** at λ=220 nm (0to60 MeCN).

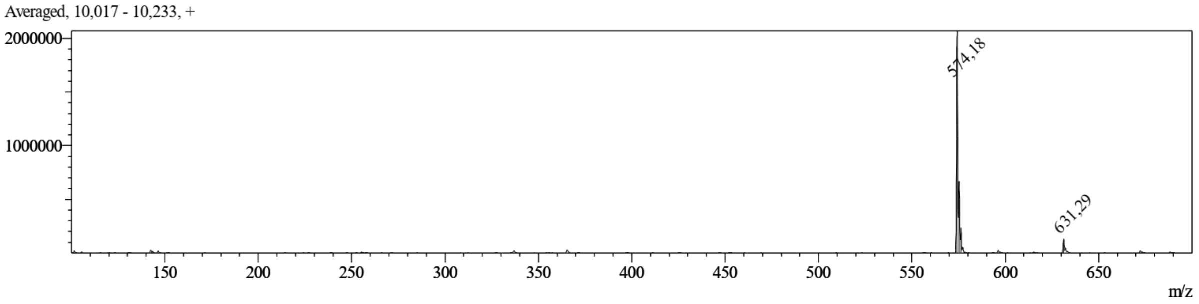

Figure S485: ESI-MS of Synthesis of Met-Enkephalin **53** (M measured=574.18 $[M+H]^+$, M calc.=573.67).

3.7.7. Analytical data of Synthesis of Leu-Enkephalin 54

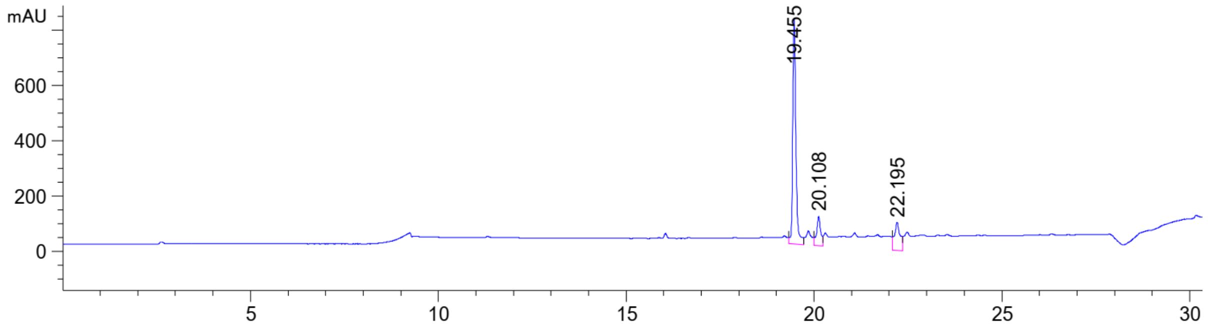

Figure S486: HPLC chromatogram of Synthesis of Leu-Enkephalin **54** at λ=220 nm (0to60 MeCN).

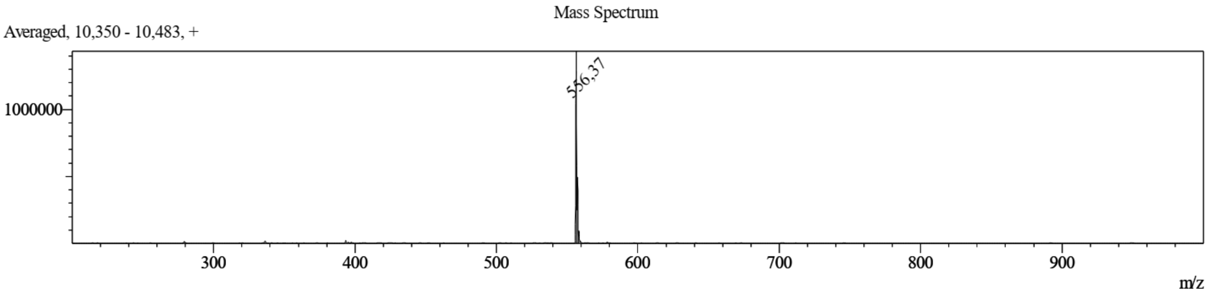

Figure S487: ESI-MS of Synthesis of Leu-Enkephalin **54** (M measured=556.37 $[M+H]^+$, M calc.=555.63).

3.7.8. Analytical data of Synthesis of H-VQAAIDYING-OH 55

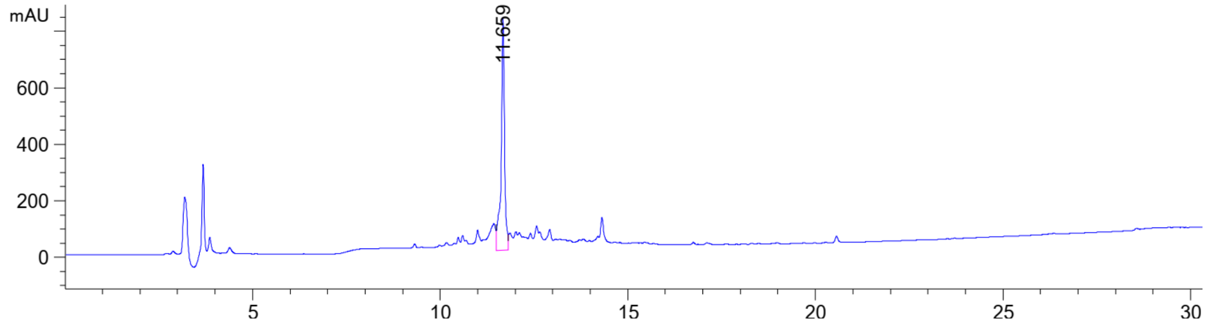

Figure S488: HPLC chromatogram of Synthesis of H-VQAAIDYING-OH **55** at λ=220 nm (10to100 MeCN).

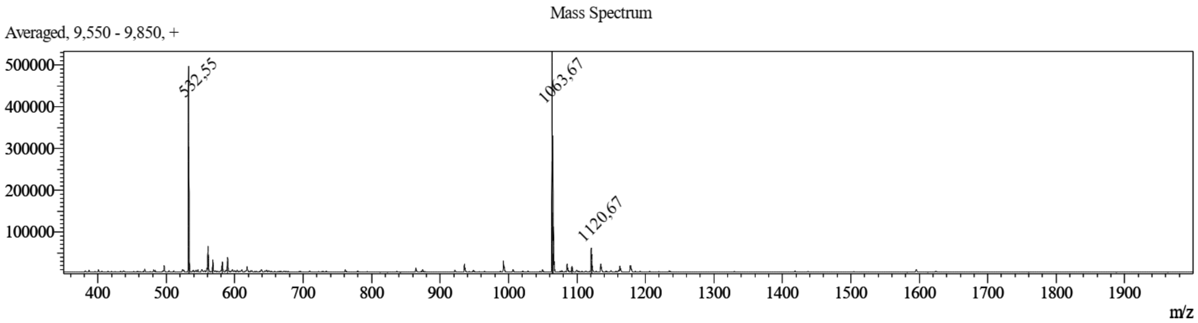

Figure S489: ESI-MS of Synthesis of H-VQAAIDYING-OH **55** (M measured=1063.67 $[M+H]^+$, M calc.=1062.19).

3.7.9. Analytical data of Synthesis of H-VQAAIDYING-NH2 56

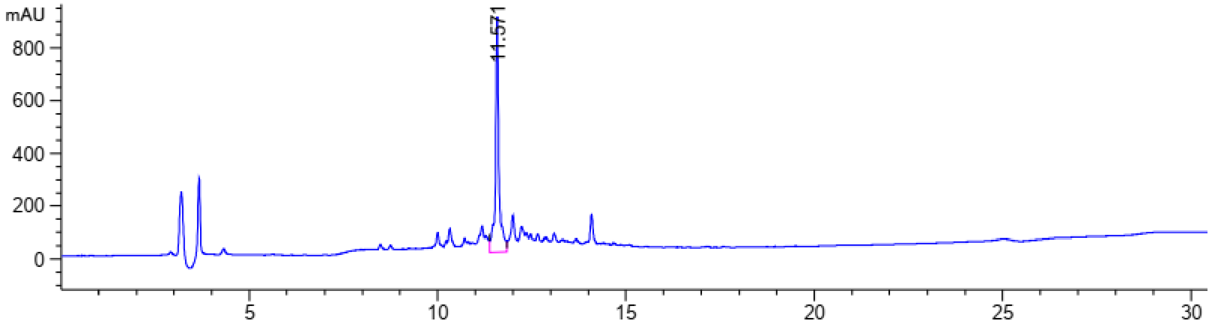

Figure S490: HPLC chromatogram of Synthesis of H-VQAAIDYING-NH2 **56** at λ=220 nm (10to100 MeCN).

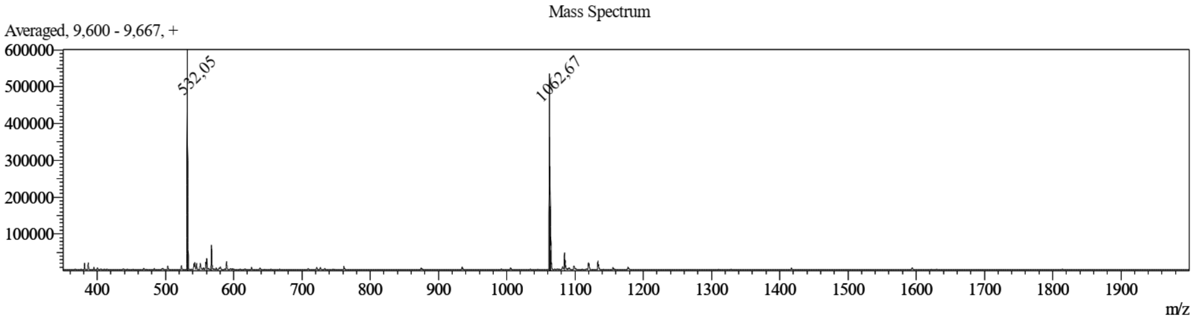

Figure S491: ESI-MS of Synthesis of H-VQAAIDYING-NH2 **56** (M measured=1062.67 $[M+H]^+$, M calc.=1063.18).

3.7.10. Analytical data of Synthesis of H-GPRP-OH 57

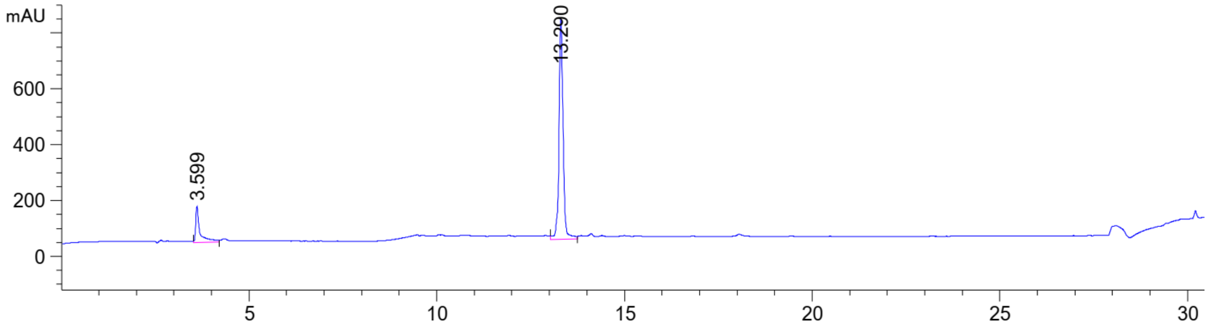

Figure S492: HPLC chromatogram of Synthesis of H-GPRP-OH **57** at λ=220 nm (0to40 MeCN).

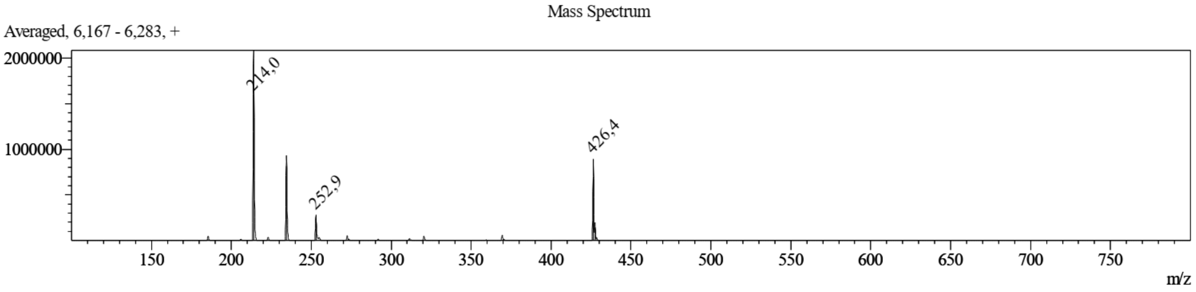

Figure S493: ESI-MS of Synthesis of H-GPRP-OH **57** (M measured=426.40 $[M+H]^+$, M calc.=425.49).

3.7.11. Analytical data of Synthesis of Smoc-VIAA-NH_2 58

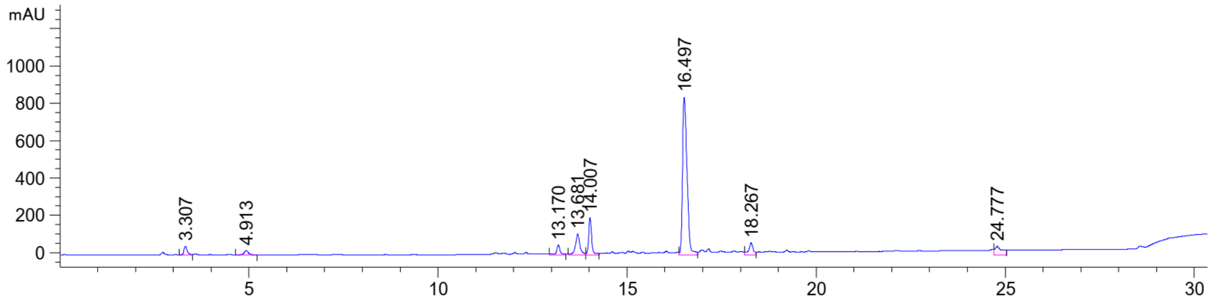

Figure S494: HPLC chromatogram of Synthesis of Smoc-VIAA-NH_2 **58** at λ=220 nm (0to40 MeCN).

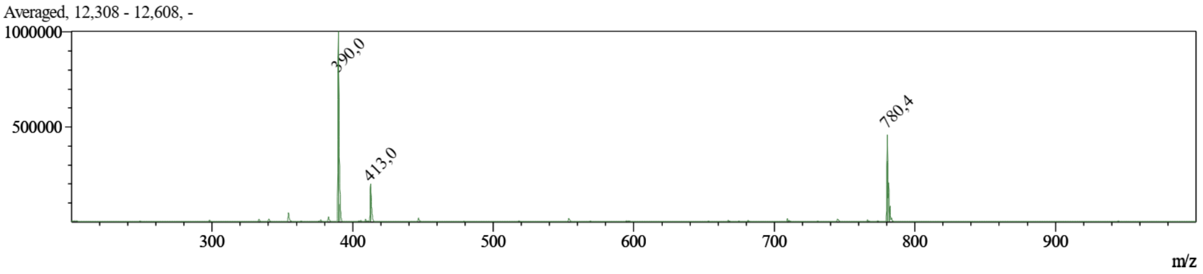

Figure S495: ESI-MS of Synthesis of Smoc-VIAA-NH_2 **58** (M measured=780.40 $[M+H]^+$, M calc.=781.89).

3.7.12. Analytical data of Synthesis of Smoc-DIIW-OH 59

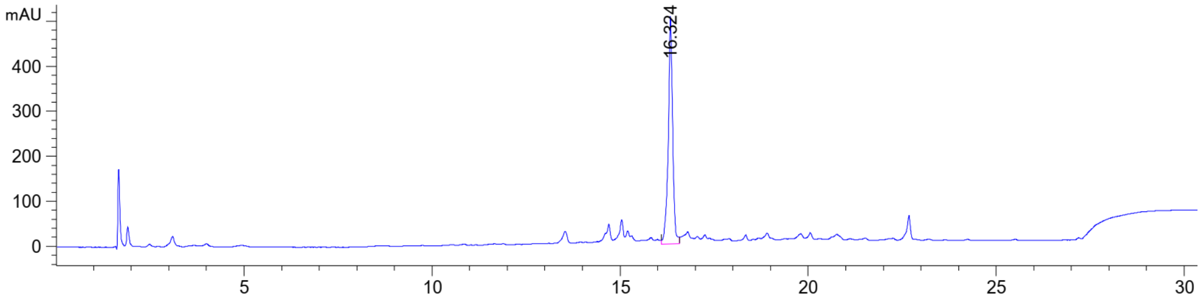

Figure S496: HPLC chromatogram of Synthesis of Smoc-DIIW-OH **59** at λ=220 nm (0to40 MeCN).

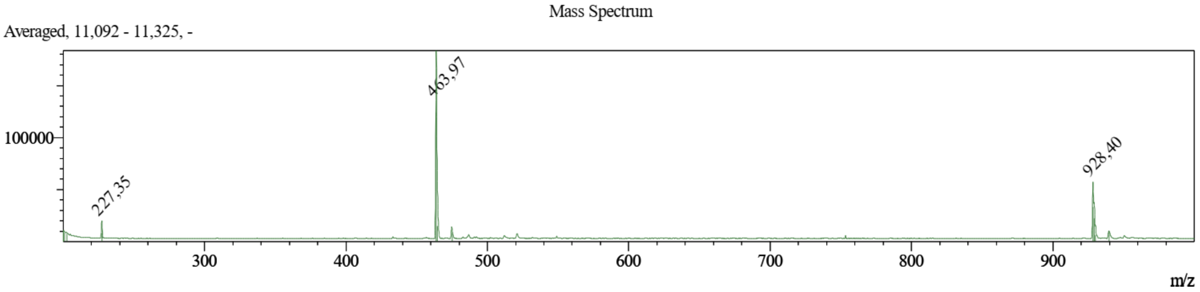

Figure S497: ESI-MS of Synthesis of Smoc-DIIW-OH **59** (M measured=928.40 $[M+H]^+$, M calc.=927.99).

3.7.13. Analytical data of Smoc-E(OtBu)K(Boc)R(Pbf)S(tBu)C(Trt)-OH 60

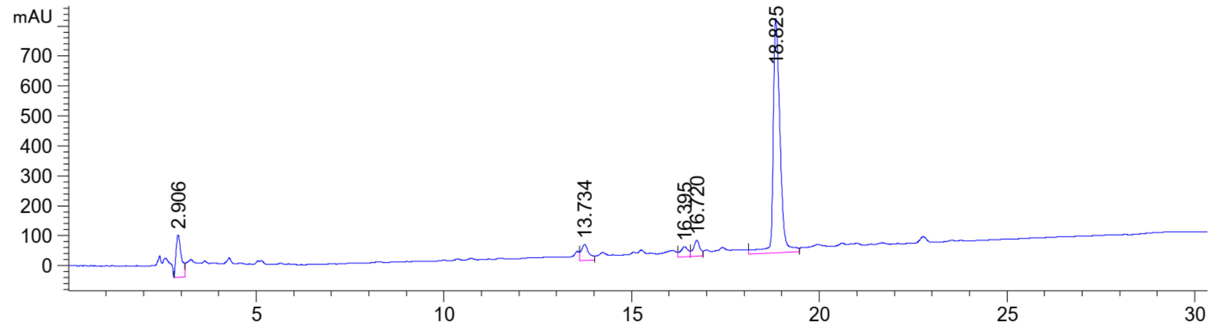

Figure S498: HPLC chromatogram of Smoc-E(OtBu)K(Boc)R(Pbf)S(tBu)C(Trt)-OH **60** at λ=220 nm (50to100 MeCN).

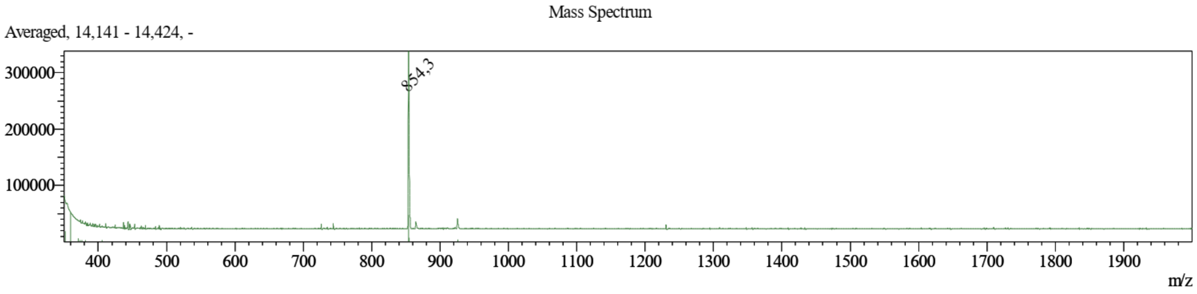

Figure S499: ESI-MS of Smoc-E(OtBu)K(Boc)R(Pbf)S(tBu)C(Trt)-OH **60** (M measured=854.30 $[M-2H]^{2-}$, M calc.= 855.53).

3.7.14. Analytical data of H-CYEIS-NH_2 61

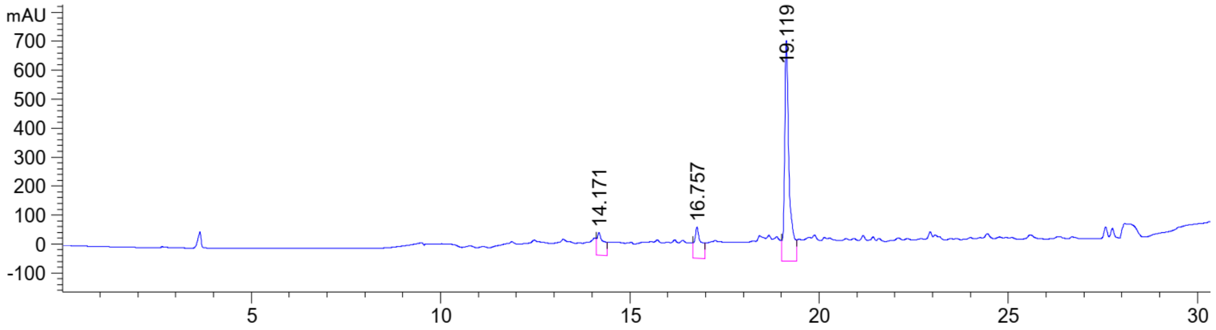

Figure S500: HPLC chromatogram of H-CYEIS-NH_2 **61** at λ=220 nm (0to40 MeCN).

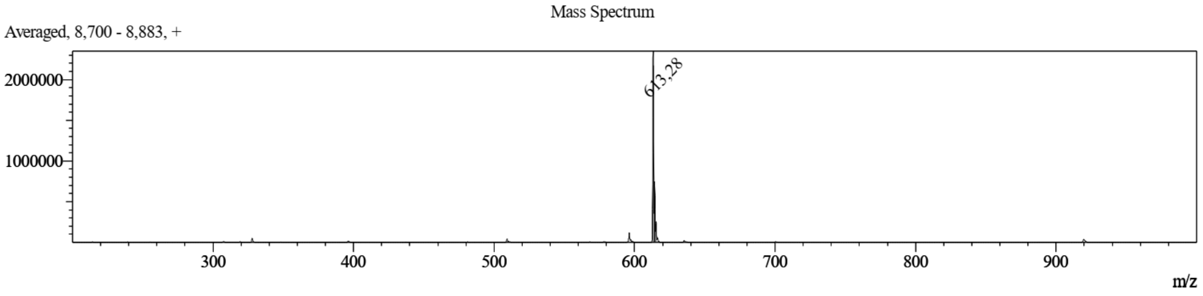

Figure S501: ESI-MS of H-CYEIS-NH_2 **61** (M measured=613.28 $[M+H]^{+}$, M calc.=612.70).

3.7.15. Analytical data of amino acid racemization of H-CYEIS-NH_2 61 by *C.A.T. GmbH & Co Chromatographie und Analysentechnik KG* (Tübingen, Germany)

C.A.T. GmbH & Co
Chromatographie und
Analysentechnik KG

Analysis number<br>9999005-1/19	H-CYEIS-NH2

Method description in accordance to SOP: **A.0.3.** **/rv 161020**

If Fmoc and DNP are present protective groups, they need to be cleaved.
The peptide / amino acid derivative is hydrolyzed in 6N DCl in D_2O. (In case of presence of Asn, it will be hydrolyzed to Asp, respectively Gln/Pyr to Glu, and so detected and determined).
If necessary an antioxidant and/or scavenger is added. After completion of hydrolysis excess of reagent is removed and the sample is esterified with deuterochloride in methyl alcohol. In accordance to the column specification homologue alcohols are possible. After evaporation of excess of reagent the residue is acylated using trifluoroacetic anhydrid or pentafluoropropionic anhydride. If histidine is to be determined, the -NH of the imidazol group is derivatized with propyl or butyl chloroformate in a separate step. The residue is dissolved and injected.

Calculation of enantiomeric purity:

$$\% \ D = \frac{Area_D}{Area_D + Area_L} * 100$$

Change Control:
Minor changes that are not in accordance to the method description are mentioned in the report. Major changes need to be approved.

System Suitability Test:

SST is running before each sequence. Results must meet the acceptance criteria.
For sensitive parameters performance qualification is determined weekly and must meet the acceptance criteria.

Data based on generic validation **Revision 010213**

Analyte:	:	Free proteinogenic amino acids
Standard deviation	:	≤± 0.1% (at ≤ 1.5% Enantiomer)<br>(For Cys and amino acids linked on to Cys possibly higher)
Limit of detection	:	<<0.1%
Limit of quantitation	:	0.10%
Range	:	0.1 - 5 % Enantiomer

The standard deviation of m-1 contribution that must be considered for some amino acids, is substance and matrix specific. Thus, also the standard deviation of the result for those amino acids could be higher. For amino acids which show complex mass pattern, such as Trp and Nal, standard deviation could be even higher than 0.3%.

We assure that the analysis is performed in accordance to the GMP guidelines and in accordance to ICH guideline Q2(R1)

181112 A_0_3_e.xls

2/8

Sitz: Tübingen-Hirschau; Registergericht: Stuttgart HRA 381110 Persönlich haftende Gesellschafterin: C.A.T. Beteiligungs-GmbH; Sitz: Tübingen Hirschau
Registergericht: Stuttgart HRB 380849; Geschäftsführer: Dr. Jürgen Gerhardt, Dr. Heike Gerhardt

Analysis number	H-CYEIS-NH2
9999005-1/19	

Results: A.0.3. /rv 161020

The listed amino acid(s) were identified via retention time and mass spectra.
The identity of the main component(s) comply.
The following impurity of the optical antipode was found:

**Isoleucine**	>99.7	% L-Isoleucine
	<0.10	% D-Isoleucine
	<0.10	% L-allo-Isoleucine
	<0.10	% D-allo-Isoleucine
**Serine**	<0.10	% D-Enantiomer
**Cysteine**	0.10	% D-Enantiomer
**Glutamic acid**	<0.10	% D-Enantiomer
**Tyrosine**	0.25	% D-Enantiomer

Notes:

Method specific deviations or irregularities are not observed.

The method is generically validated. However it may not meet all requirements for the release of drug substances and drug products. It is to prove if substance specific validation is required.

Sitz: Tübingen-Hirschau; Registergericht: Stuttgart HRA 381110 Persönlich haftende Gesellschafterin: C.A.T. Beteiligungs-GmbH; Sitz: Tübingen Hirschau
Registergericht: Stuttgart HRB 380849; Geschäftsführer: Dr. Jürgen Gerhardt, Dr. Heike Gerhardt

Signal: MS1Front SIM(182) EI

RT [min]	Type	Width [min]	Area	Height	Area%	Name
10.829	MM m	0.11	41.4	6.0	0.03	D Ail
11.806	MM m	0.04	5.8	2.0	0.00	D Ile
12.485	MM m	0.09	73.7	10.0	0.05	L Ail
13.046	BB	1.41	141005.4	17553.3	99.91	L Ile

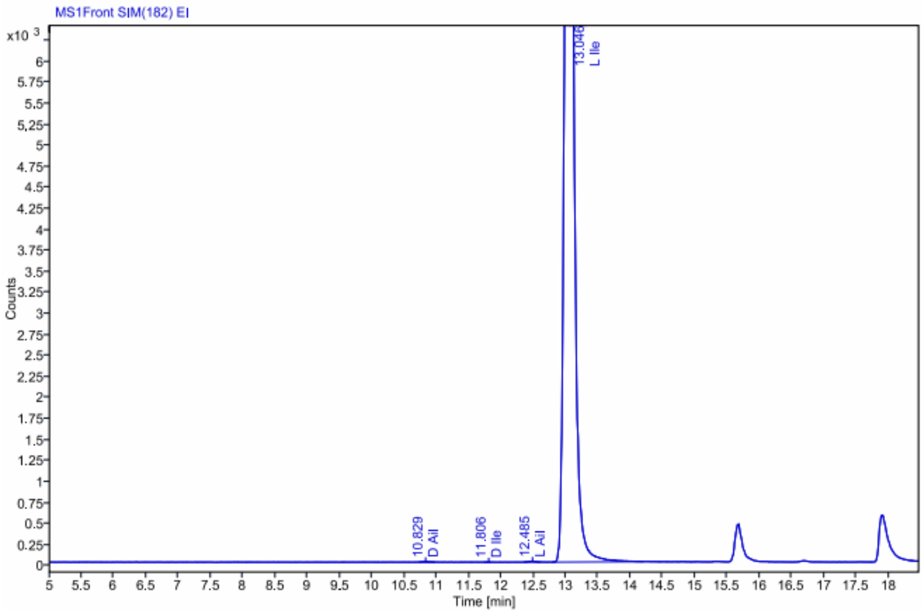

Signal: MS1Front SIM(138) EI

RT [min]	Type	Width [min]	Area	Height	Area%	Name
14.533	MM m	0.14	141.8	16.9	0.06	D Ser
15.662	BB	1.36	235395.0	29397.8	99.94	L Ser

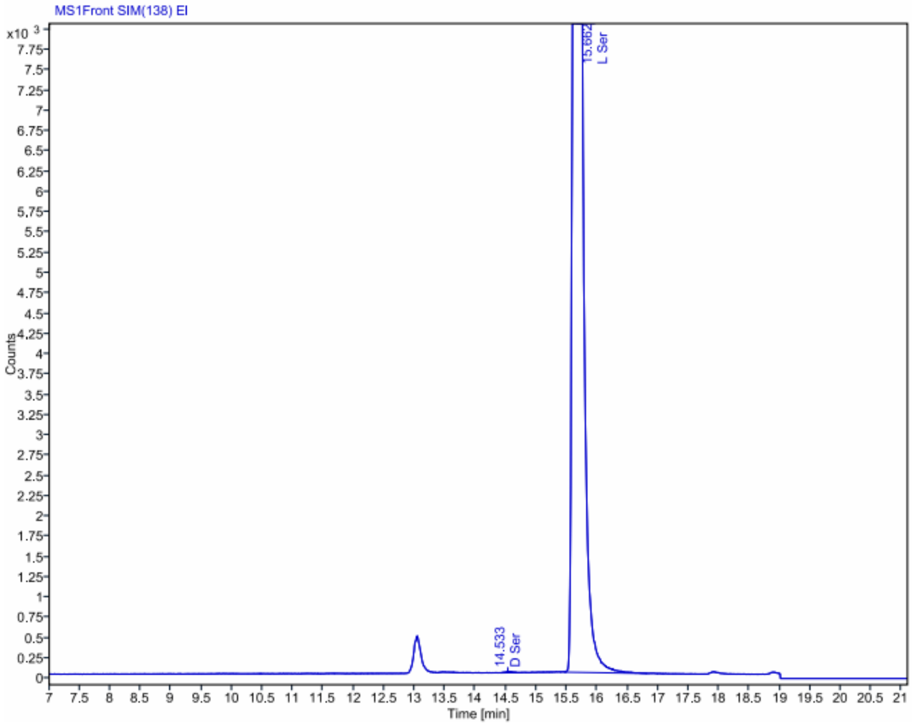

Signal:	MS1Front SIM(268) EI				
RT [min]	Type	Width [min]	Area	Height	Area% Name
21.549	MM m	0.09	42.3	6.0	0.09 D Cys
22.392	BB	1.37	44609.6	6939.5	99.91 L Cys

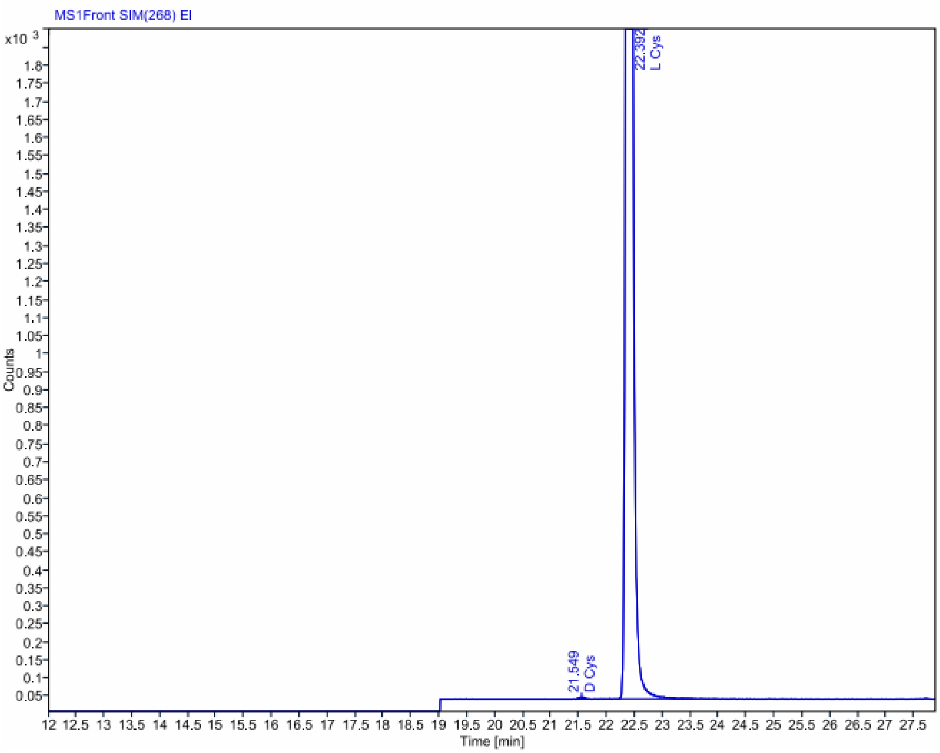

Signal:	MS1Front SIM(226) EI				
RT [min]	Type	Width [min]	Area	Height	Area% Name
26.570	MM m	0.05	15.6	4.0	0.06 D Glu
27.722	BB	1.11	25187.9	4882.1	99.94 L Glu

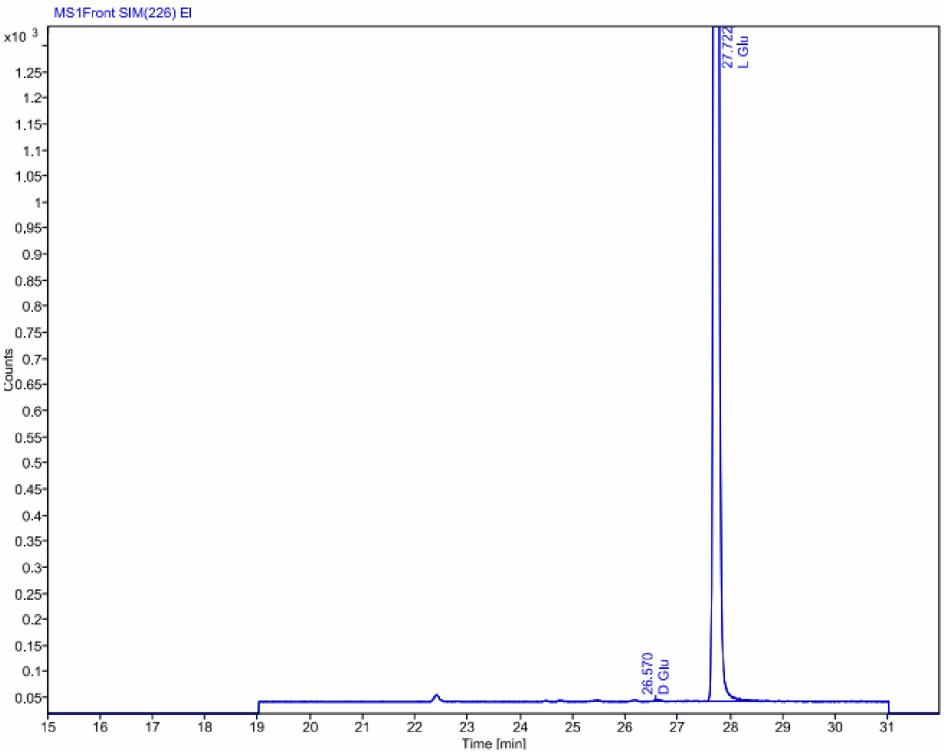

Signal:	MS1Front SIM(288) EI				
RT [min]	Type	Width [min]	Area	Height	Area% Name
32.116	MM m	0.06	15.2	4.0	0.25 D Tyr
32.538	BB	0.91	5998.4	2044.7	99.75 L Tyr

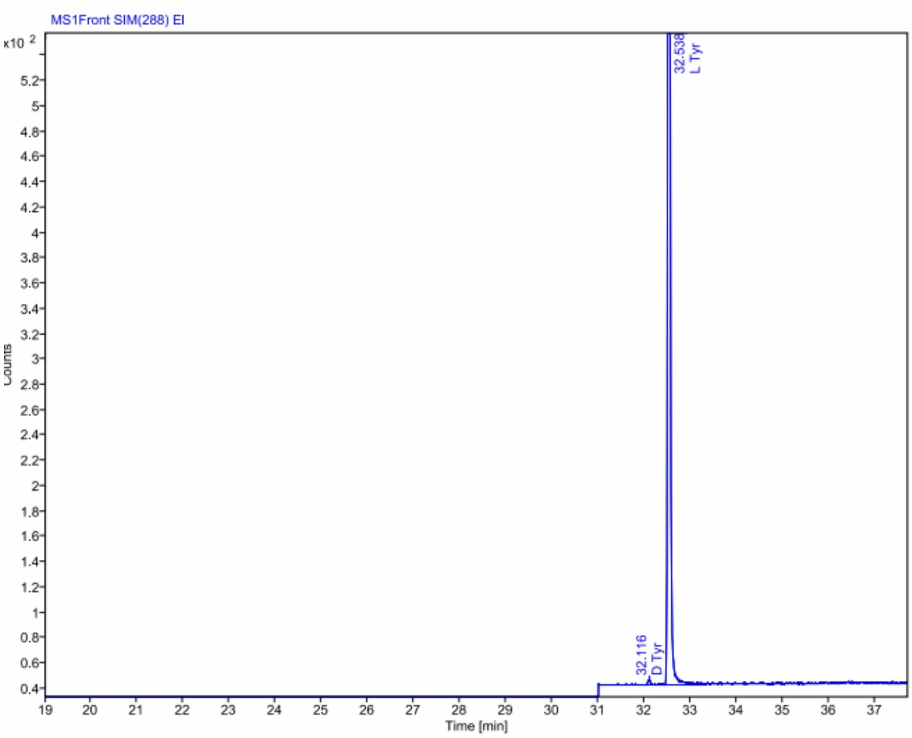

3.7.16. Analytical data of H-ANKPG-NH_2 62

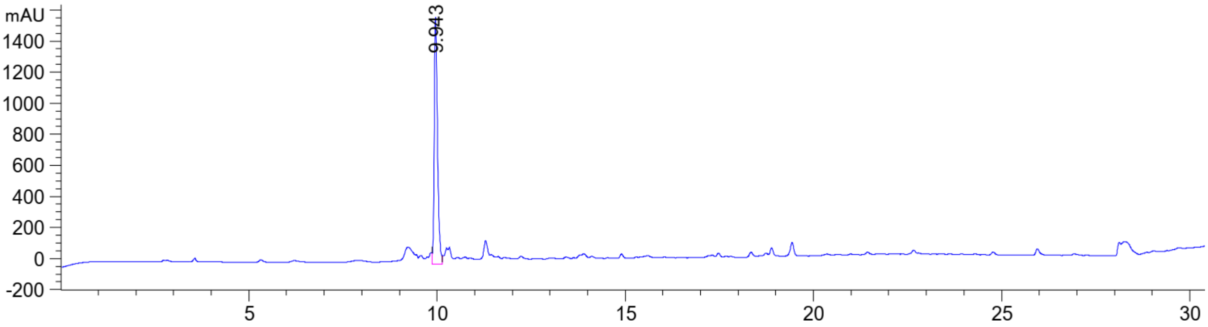

Figure S502: HPLC chromatogram of H-ANKPG-NH_2 **62** at λ=220 nm (0to40 MeCN).

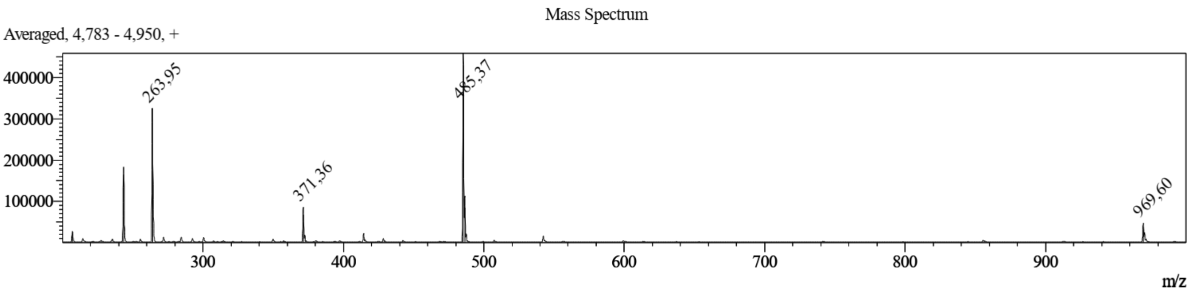

Figure S503: ESI-MS of H-ANKPG-NH_2 **62** (M measured=485.37 $[M+H]^+$, M calc.= 484.56).

3.7.17. Analytical data of amino acid racemization of H-ANKPG-NH_2 62 by *C.A.T. GmbH & Co Chromatographie und Analysentechnik KG* (Tübingen, Germany)

C.A.T. GmbH & Co
Chromatographie und
Analysentechnik KG

Analysis number<br>9999005-2/19	H-ANKPG-NH2

Method description in accordance to SOP: A.0.3. /rv 161020

If Fmoc and DNP are present protective groups, they need to be cleaved.
The peptide / amino acid derivative is hydrolyzed in 6N DCl in D_2O. (In case of presence of Asn, it will be hydrolyzed to Asp, respectively Gln/Pyr to Glu, and so detected and determined).
If necessary an antioxidant and/or scavenger is added. After completion of hydrolysis excess of reagent is removed and the sample is esterified with deuterochloride in methyl alcohol. In accordance to the column specification homologue alcohols are possible. After evaporation of excess of reagent the residue is acylated using trifluoroacetic anhydrid or pentafluoropropionic anhydride. If histidine is to be determined, the -NH of the imidazol group is derivatized with propyl or butyl chloroformate in a separate step. The residue is dissolved and injected.

Calculation of enantiomeric purity:

$$\% \ D = \frac{Area_{\ D}}{Area_{\ D} + Area_{\ L}} * 100$$

Change Control:
Minor changes that are not in accordance to the method description are mentioned in the report. Major changes need to be approved.

System Suitability Test:

SST is running before each sequence. Results must meet the acceptance criteria.
For sensitive parameters performance qualification is determined weekly and must meet the acceptance criteria.

Data based on generic validation Revision 010213

Analyte:	:	Free proteinogenic amino acids
Standard deviation	:	≤± 0.1% (at ≤ 1.5% Enantiomer)<br>(For Cys and amino acids linked on to Cys possibly higher)
Limit of detection	:	<<0.1%
Limit of quantitation	:	0.10%
Range	:	0.1 - 5 % Enantiomer

The standard deviation of m-1 contribution that must be considered for some amino acids, is substance and matrix specific. Thus, also the standard deviation of the result for those amino acids could be higher. For amino acids which show complex mass pattern, such as Trp and Nal, standard deviation could be even higher than 0.3%.

We assure that the analysis is performed in accordance to the GMP guidelines and in accordance to ICH guideline Q2(R1)

Sitz: Tübingen-Hirschau; Registergericht: Stuttgart HRA 381110 Persönlich haftende Gesellschafterin: C.A.T. Beteiligungs-GmbH; Sitz: Tübingen Hirschau
Registergericht: Stuttgart HRB 380849; Geschäftsführer: Dr. Jürgen Gerhardt, Dr. Heike Gerhardt

Analysis number	H-ANKPG-NH2
9999005-2/19	

Results: A.0.3. /rv 161020

The listed amino acid(s) were identified via retention time and mass spectra.
The identity of the main component(s) comply.
The following impurity of the optical antipode was found:

**Alanine**	0.50	% D-Enantiomer
**Proline**	0.43	% D-Enantiomer
**Aspartic acid**	2.04	% D-Enantiomer
**Lysine**	0.11	% D-Enantiomer

Notes:

Method specific deviations or irregularities are not observed.

The method is generically validated. However it may not meet all requirements for the release of drug substances and drug products. It is to prove if substance specific validation is required.

Sitz: Tübingen-Hirschau; Registergericht: Stuttgart HRA 381110 Persönlich haftende Gesellschafterin: C.A.T. Beteiligungs-GmbH; Sitz: Tübingen Hirschau
Registergericht: Stuttgart HRB 380849; Geschäftsführer: Dr. Jürgen Gerhardt, Dr. Heike Gerhardt

Signal: MS1Front SIM(140) EI

RT [min]	Type	Width [min]	Area	Height	Area%	Name
4.528	MM m	0.12	1254.2	136.5	0.50	D Ala
6.056	BB	1.89	251047.8	23385.5	99.50	L Ala

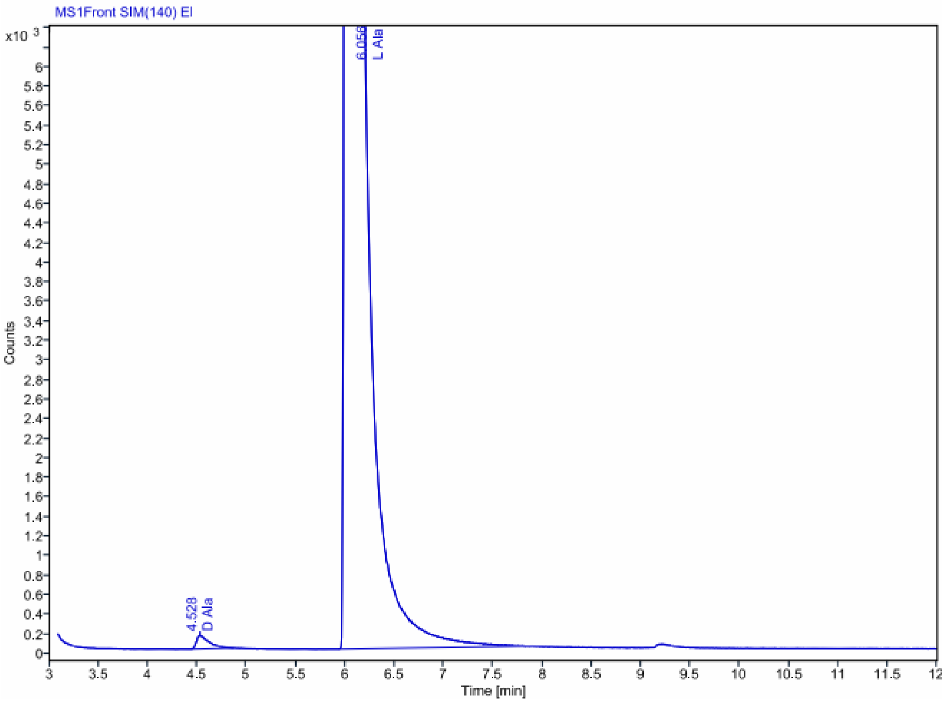

Signal: MS1Front SIM(166) EI

RT [min]	Type	Width [min]	Area	Height	Area%	Name
12.127	BV	0.2	1540.6	232.2	0.43	D Pro
12.321	VB	1.8	356320.5	37634.4	99.57	L Pro

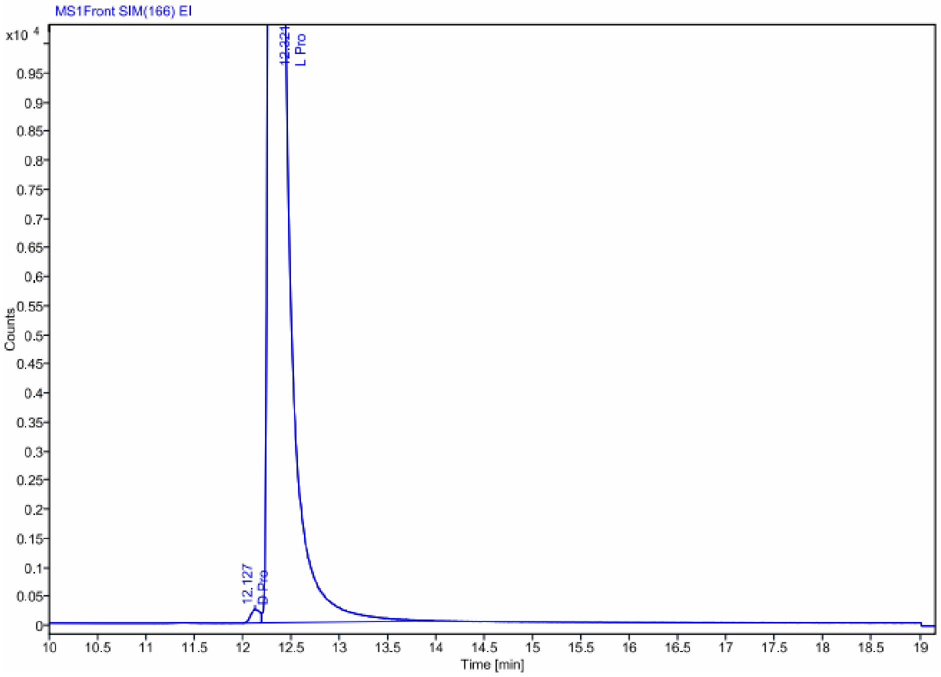

Signal: MS1Front SIM(214) EI

RT [min]	Type	Width [min]	Area	Height	Area%	Name
20.435	BV	0.49	2291.2	298.7	2.27	D Asp
20.961	VB	1.40	98466.7	14560.7	97.73	L Asp

M-1 contribution of 0.23% must be subtracted

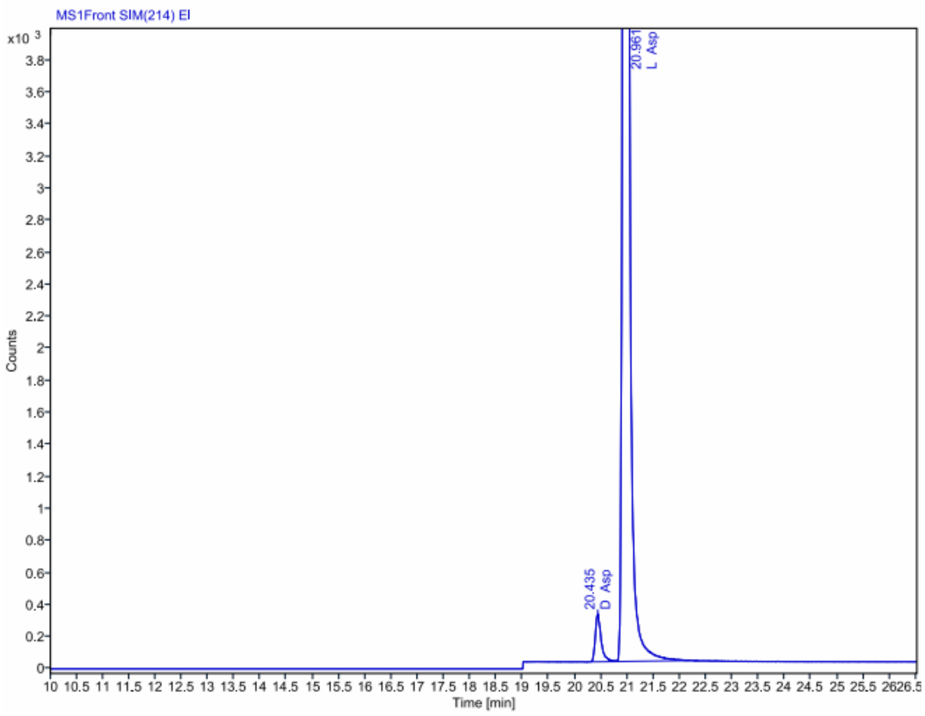

Signal: MS1Front SIM(180) EI

RT [min]	Type	Width [min]	Area	Height	Area%	Name
35.095	MM m	0.04	536.0	217.8	0.11	D Lys
35.354	BB	0.72	500473.9	251147.6	99.89	L Lys

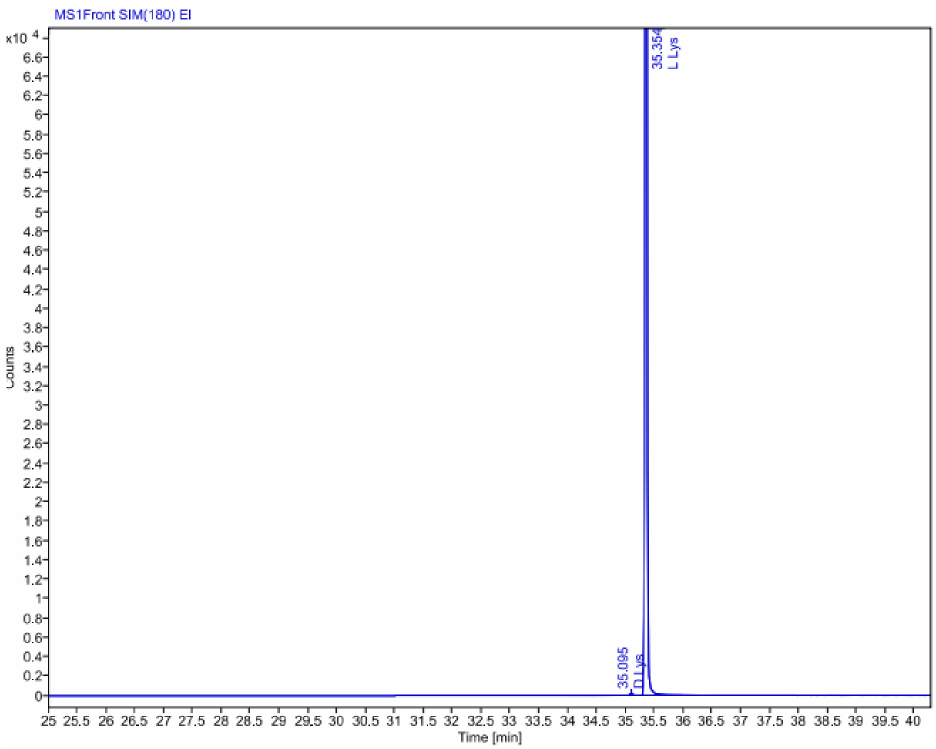

3.7.18. Analytical data of amino acid racemization of Smoc-Asn-OH 7 by *C.A.T. GmbH & Co Chromatographie und Analysentechnik KG* (Tübingen, Germany)

C.A.T. GmbH & Co
Chromatographie und
Analysentechnik KG

Analysis number<br>9999009-1/19	Smoc-Asn-OH

Method description in accordance to SOP: **A.0.3.** **/rv 161020**

If Fmoc and DNP are present protective groups, they need to be cleaved.
The peptide / amino acid derivative is hydrolyzed in 6N DCl in D_2O. (In case of presence of Asn, it will be hydrolyzed to Asp, respectively Gln/Pyr to Glu, and so detected and determined).
If necessary an antioxidant and/or scavenger is added. After completion of hydrolysis excess of reagent is removed and the sample is esterified with deuterochloride in methyl alcohol. In accordance to the column specification homologue alcohols are possible. After evaporation of excess of reagent the residue is acylated using trifluoroacetic anhydrid or pentafluoropropionic anhydride. If histidine is to be determined, the -NH of the imidazol group is derivatized with propyl or butyl chloroformate in a separate step. The residue is dissolved and injected.

Calculation of enantiomeric purity:

$$\% \ D = \frac{Area_{\ D}}{Area_{\ D} + Area_{\ L}} * 100$$

Change Control:
Minor changes that are not in accordance to the method description are mentioned in the report. Major changes need to be approved.

System Suitability Test:

SST is running before each sequence. Results must meet the acceptance criteria.
For sensitive parameters performance qualification is determined weekly and must meet the acceptance criteria.

Data based on generic validation **Revision 010213**

Analyte:	:	Free proteinogenic amino acids
Standard deviation	:	≤± 0.1% (at ≤ 1.5% Enantiomer)<br>(For Cys and amino acids linked on to Cys possibly higher)
Limit of detection	:	<<0.1%
Limit of quantitation	:	0.10%
Range	:	0.1 - 5 % Enantiomer

The standard deviation of m-1 contribution that must be considered for some amino acids, is substance and matrix specific. Thus, also the standard deviation of the result for those amino acids could be higher. For amino acids which show complex mass pattern, such as Trp and Nal, standard deviation could be even higher than 0.3%.
If the LOQ of generic validation cannot be met due to substance-specific infuences, the result will be reported as < <estimated LOQ >.

We assure that the analysis is performed in accordance to the GMP guidelines and in accordance to ICH guideline Q2(R1)

Sitz: Tübingen-Hirschau; Registergericht: Stuttgart HRA 381110 Persönlich haftende Gesellschafterin: C.A.T. Beteiligungs-GmbH; Sitz: Tübingen Hirschau
Registergericht: Stuttgart HRB 380849; Geschäftsführer: Dr. Jürgen Gerhardt, Dr. Heike Gerhardt

Analysis number	Smoc-Asn-OH
9999009-1/19	

Results: A.0.3. /rv 161020

The listed amino acid(s) were identified via retention time and mass spectra.
The identity of the main component(s) comply.
The following impurity of the optical antipode was found:

Aspartic acid 0.30 % D-Enantiomer

Notes:

Method specific deviations or irregularities are not observed.

The method is generically validated. However it may not meet all requirements for the release of drug substances and drug products. It is to prove if substance specific validation is required.

Signal: MS1Other SIM(212) EI

RT [min]	Type	Width [min]	Area	Height	Area%	Name
14.870	MM m	0.06	495.0	124.1	0.30	D_Asp
15.330	MM m	0.07	161845.4	31748.9	99.70	L_Asp

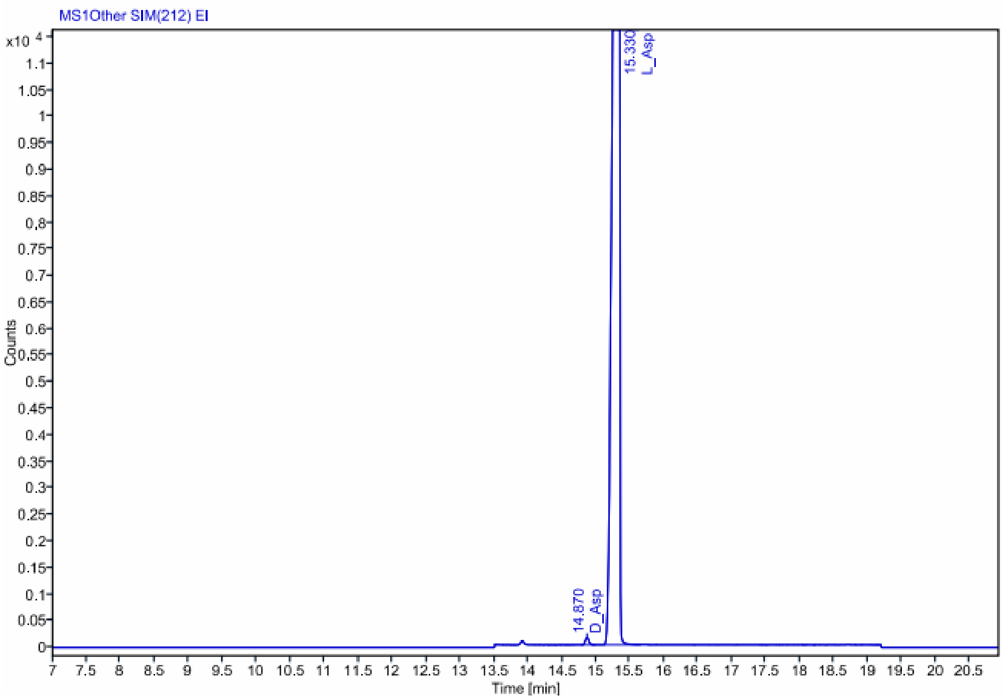

3.7.19. Analytical data of Pal-GHK-OH 63

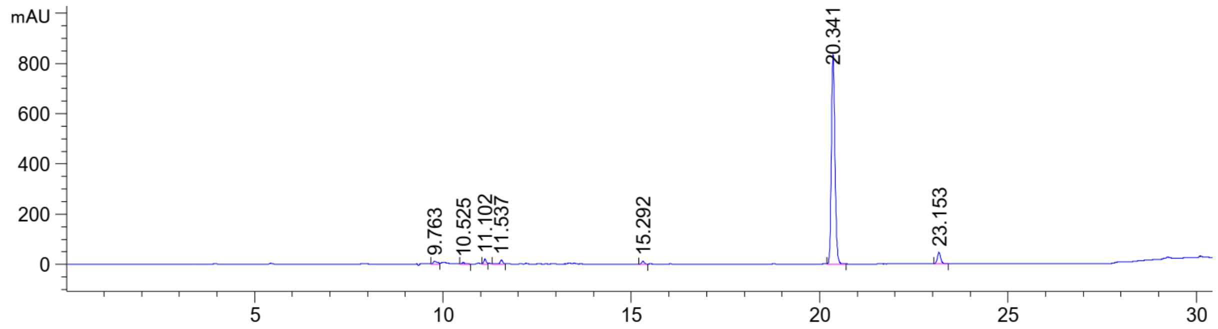

Figure S504: HPLC chromatogram of Pal-GHK-OH **63** at λ=220 nm (10to60 MeCN).

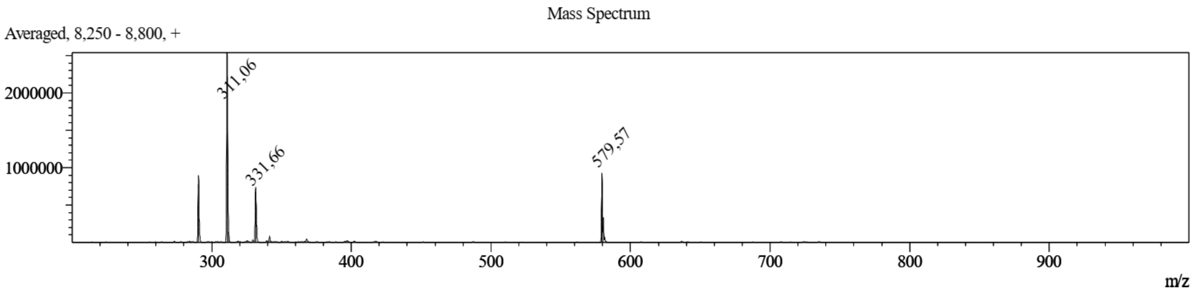

Figure S505: ESI-MS of Pal-GHK-OH **63** (M measured=579.57 $[M+H]^+$, M calc.= 578.80).

3.7.20. Analytical data of Pal-GQPR-OH 64

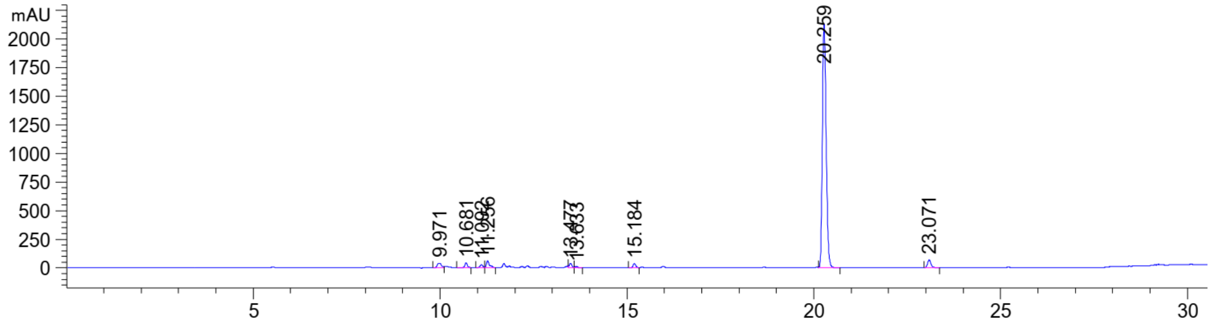

Figure S506: HPLC chromatogram of Pal-GQPR-OH **64** at λ=220 nm (0to60 MeCN).

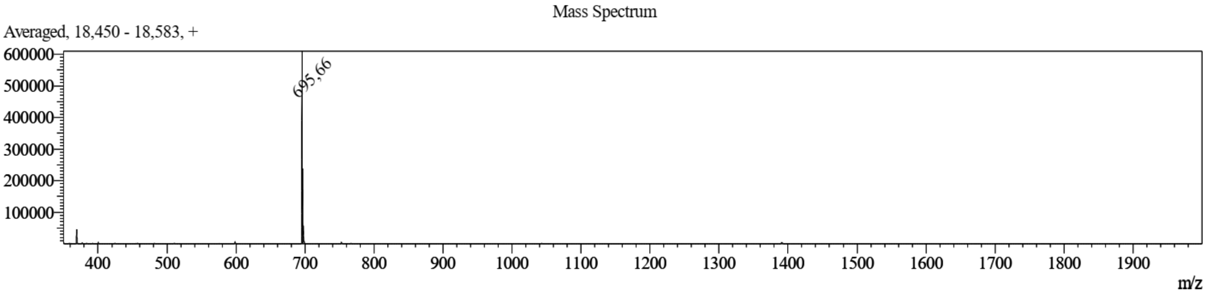

Figure S507: ESI-MS of Pal-GQPR-OH **64** (M measured=695.66 $[M+H]^+$, M calc.=695.66).

3.7.21. Analytical data of H-GPRPA-NH_2 Vialox (Pentapeptide-3) 65

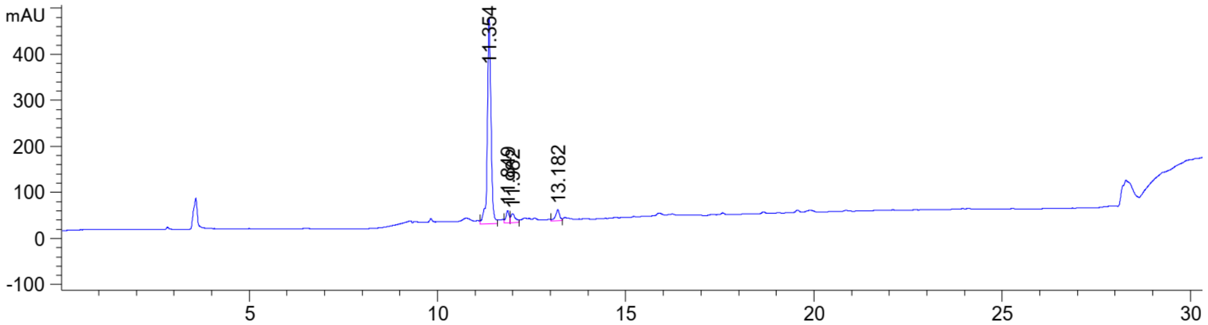

Figure S508: HPLC chromatogram of H-GPRPA-NH_2 **65** at λ=220 nm (0to60 MeCN).

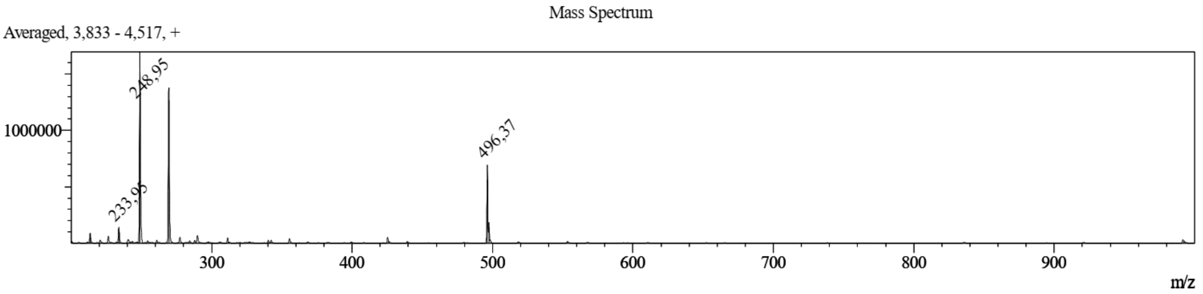

Figure S509: ESI-MS of H-GPRPA-NH_2 **65** (M measured=496.37 $[M+H]^+$, M calc.=495.59).

3.7.22. Analytical data of Oxytocin 66

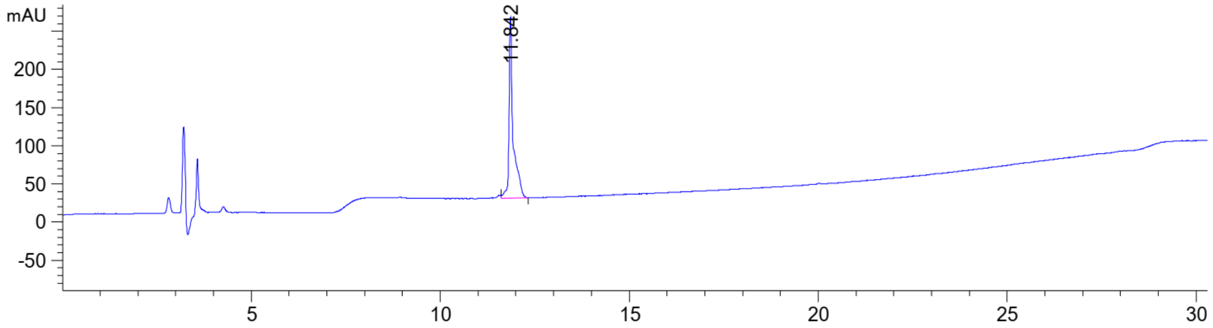

Figure S510: HPLC chromatogram of Oxytocin **66** at λ=220 nm (10to100 MeCN).

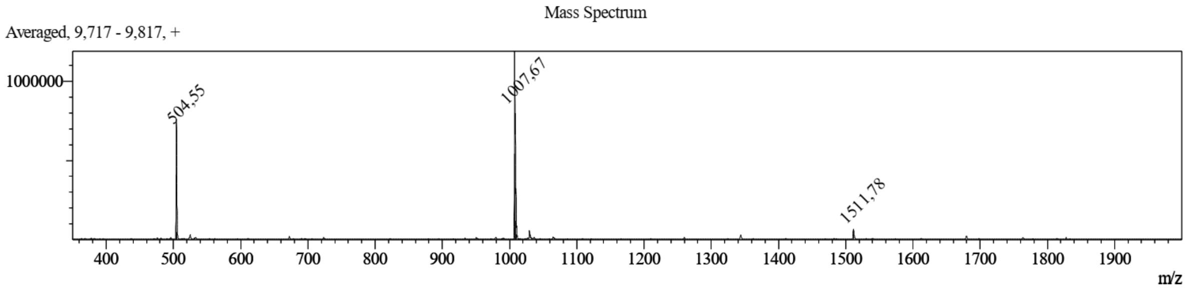

Figure S511: ESI-MS of Oxytocin **66** (M measured=1007.67 $[M+H]^+$, M calc.=1007.19).

3.7.23. Analytical data of Vasopressin 67 (peptide hormone)

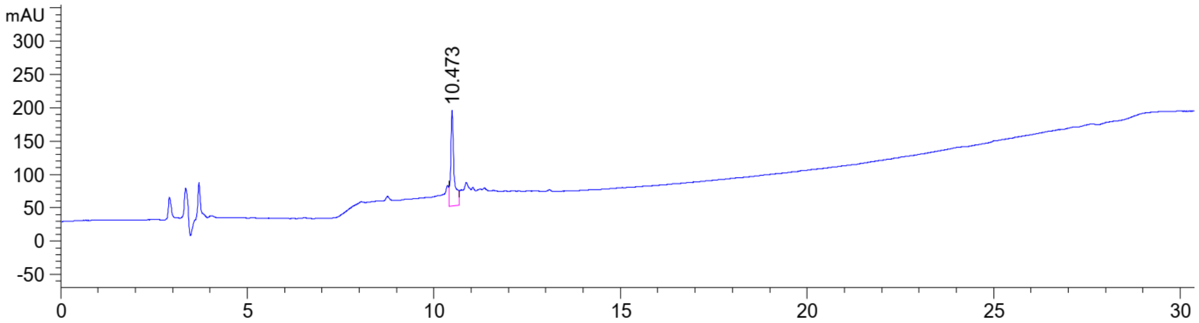

Figure S512: HPLC chromatogram of Vasopressin **67** at λ=220 nm (10to100 MeCN).

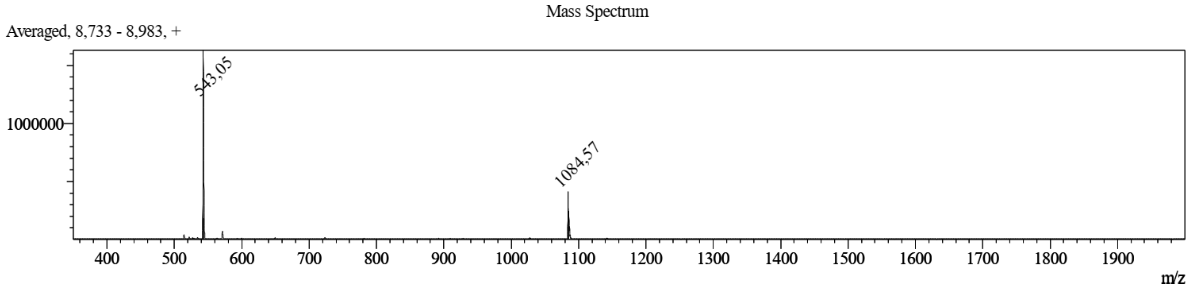

Figure S513: ESI-MS of Vasopressin **67** (M measured=1084.57 $[M+H]^+$, M calc.=1084.57).

3.7.24. Analytical data of heptaarginine 68

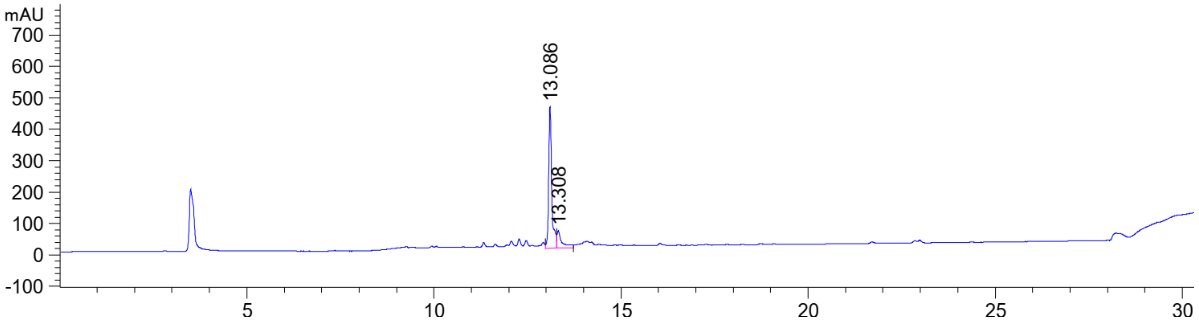

Figure S514: HPLC chromatogram of Smoc-heptaarginine **68** at λ=220 nm (10to100 MeCN).

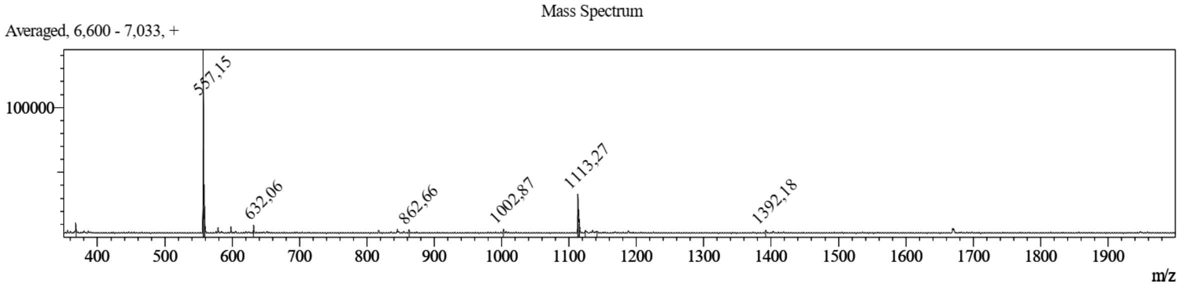

Figure S515: ESI-MS of heptaarginine **68** (M measured=1113.27 $[M+H]^+$, M calc.=1110.35).

3.7.25. Analytical data of H-YDAGFL-OH Leuphasyl 69

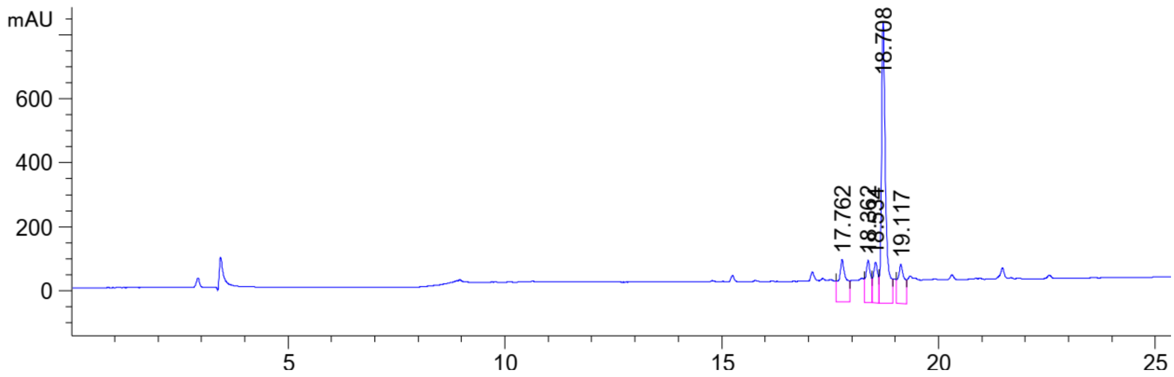

Figure S516: HPLC chromatogram of H-YDAGFL-OH **69** at λ=220 nm (0to60 MeCN).

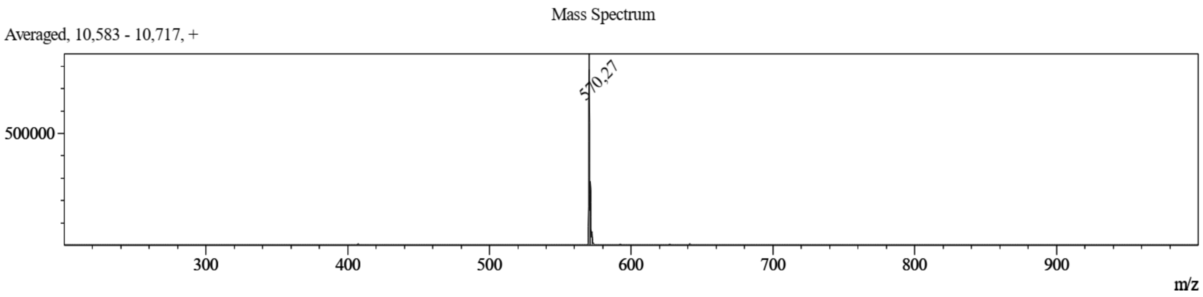

Figure S517: ESI-MS of H-YDAGFL-OH **69** (M measured=570.27 $[M+H]^+$, M calc.=569.66).

3.8. Analytical data of Aspartimide formation

3.8.1. Reference HPLC data of peptides 70-73

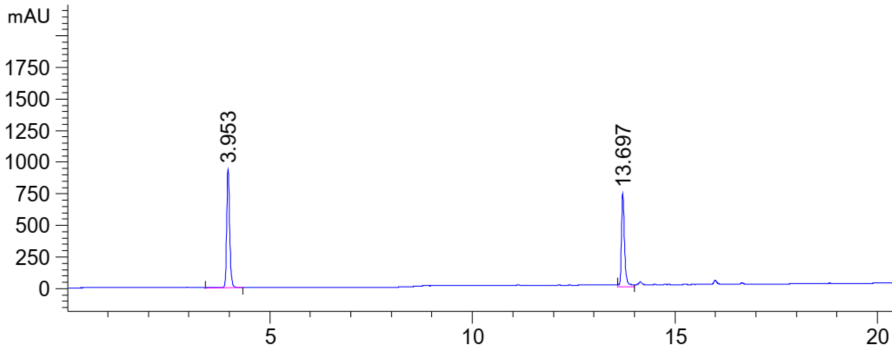

Figure S518: HPLC chromatogram of H-VKDGYI-NH_2 **70** as reference at λ=220 nm (0to60 MeCN) with ascorbic acid as standard at ≈3.95 min.

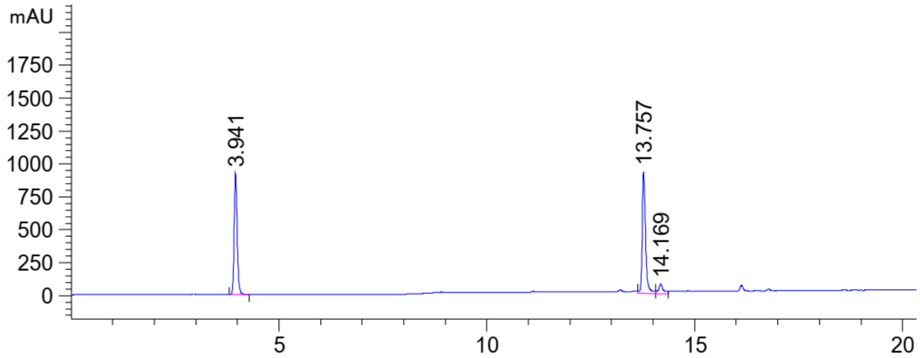

Figure S519: HPLC chromatogram of H-VK(D-D)GYI-NH_2 **71** as reference at λ=220 nm (0to60 MeCN) with ascorbic acid as standard at ≈3.95 min.

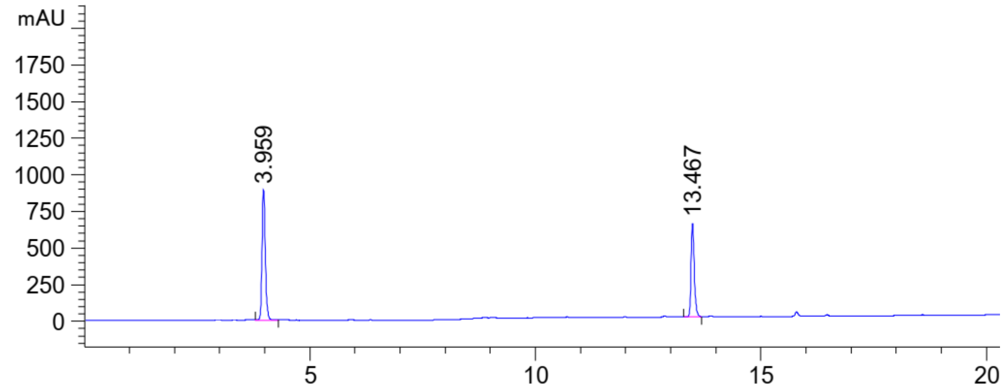

Figure S520: HPLC chromatogram of H-VKNGYI-NH_2 **72** as reference at λ=220 nm (0to60 MeCN) with ascorbic acid as standard at ≈3.95 min.

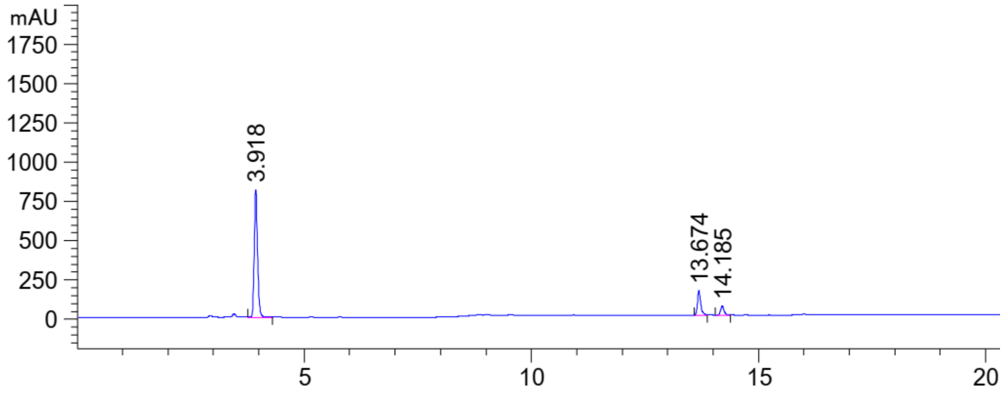

Figure S521: HPLC chromatogram of H-VK(β-D)GYI-NH_2**73** as reference at λ=220 nm (0to60 MeCN) with ascorbic acid as standard at ≈3.95 min.

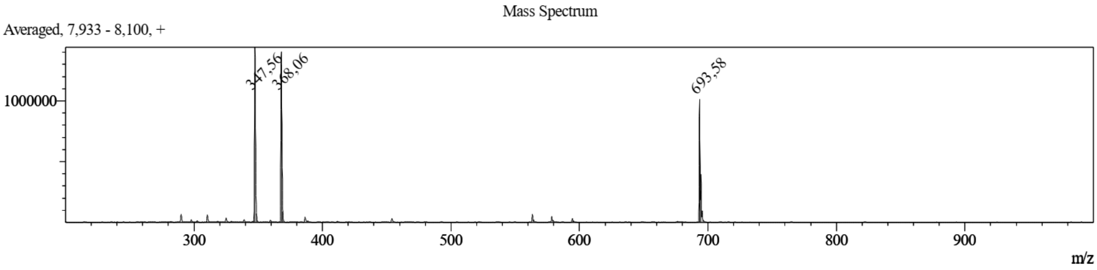

Figure S522: ESI-MS of H-VKDGYI-NH_2 **70** (M measured=693.58 $[M+H]^+$, M calc.=692.82).

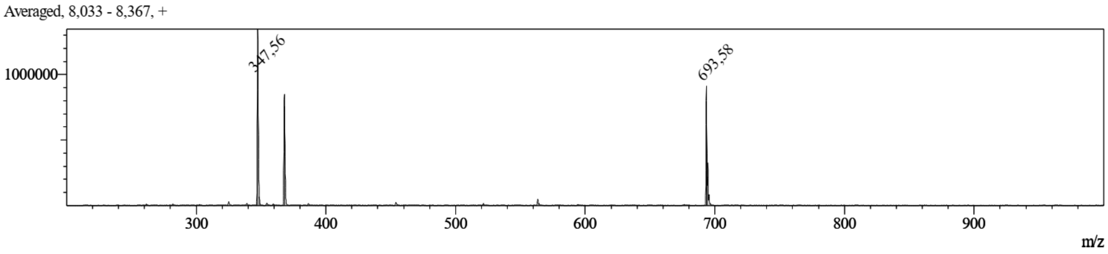

Figure S523: ESI-MS of H-VK(D-D)GYI-NH_2 **71** (M measured=693.58 $[M+H]^+$, M calc.=692.82).

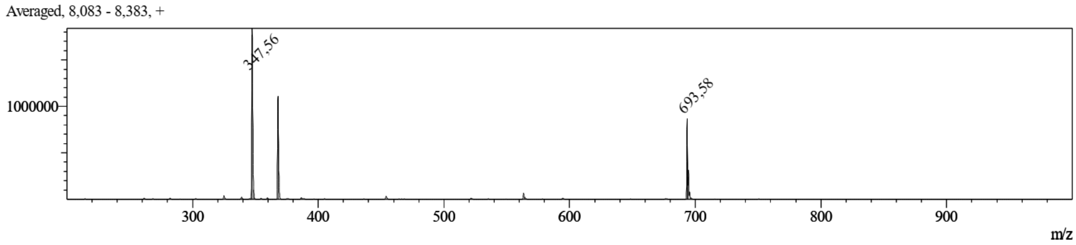

Figure S524: ESI-MS of H-VK(β-D)GYI-NH_2 **73** (M measured=693.58 $[M+H]^+$, M calc.=692.82).

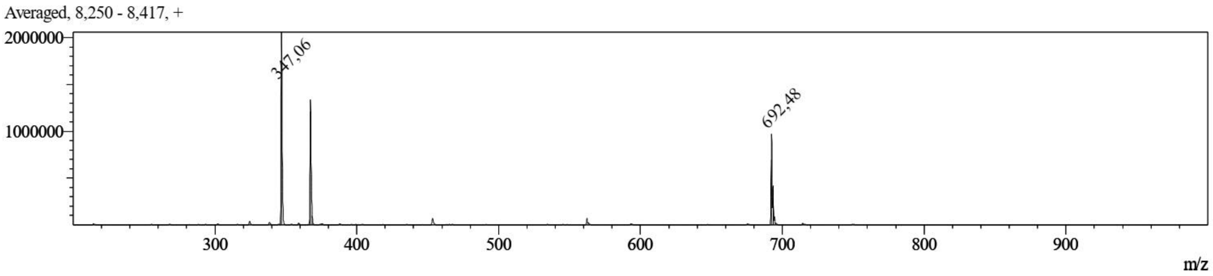

Figure S525: ESI-MS of H-VKNGYI-NH_2 **72** (M measured=692.48 $[M+H]^+$, M calc.=691.83).

3.8.2. HPLC data of H-VKDGYI-OH 70 after 3h incubation with different bases

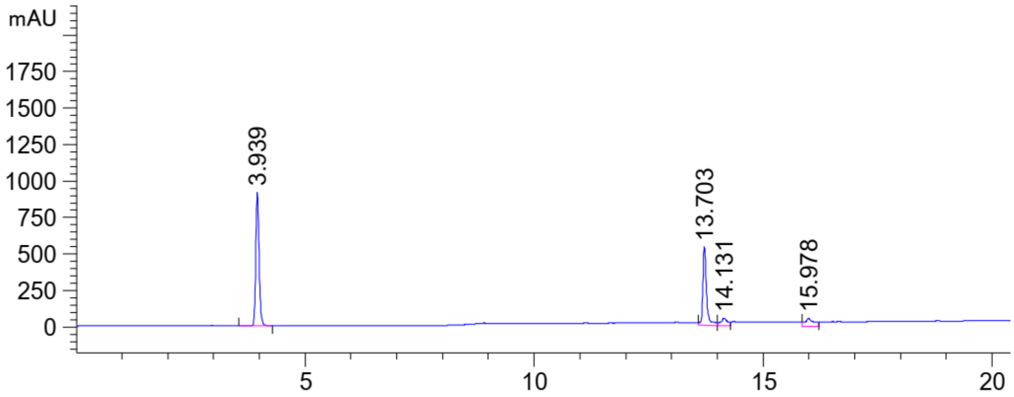

Figure S526: HPLC chromatogram of H-VKDGYI-NH_2 **70** after 3h with 5% piperazine in DMF at λ=220 nm (0to60 MeCN) with ascorbic acid as standard at ≈3.95 min.

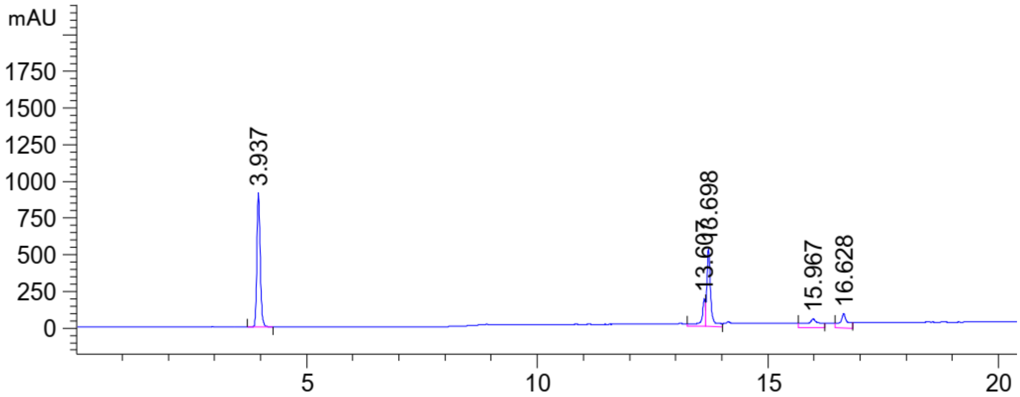

Figure S527: HPLC chromatogram of H-VKDGYI-NH_2 **70** after 3h with 5% piperazine in water at λ=220 nm (0to60 MeCN) with ascorbic acid as standard at ≈3.95 min.

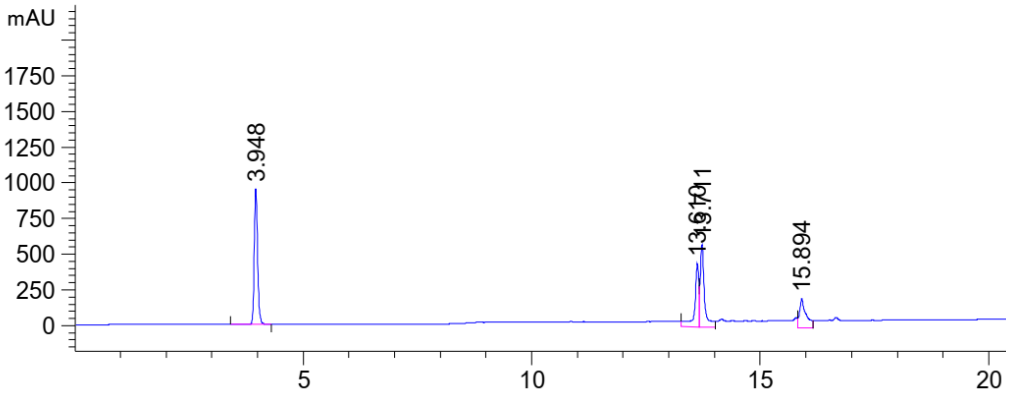

Figure S528: HPLC chromatogram of H-VKDGYI-NH_2 **70** after 3h with 10% ethanolamine in water at λ=220 nm (0to60 MeCN) with ascorbic acid as standard at ≈3.95 min.

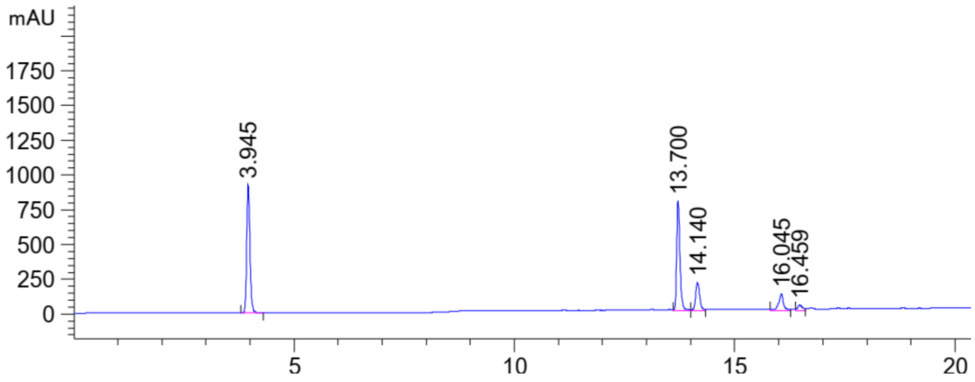

Figure S529: HPLC chromatogram of H-VKDGYI-NH_2 **70** after 3h with 20% piperidine in DMF at λ=220 nm (0to60 MeCN) with ascorbic acid as standard at ≈3.95 min.

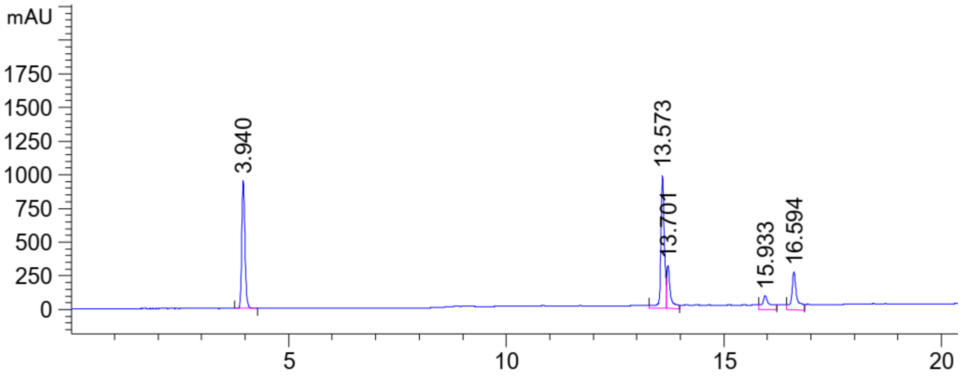

Figure S530: HPLC chromatogram of H-VKDGYI-NH_2 **70** after 3h with 1M NaOH in water at λ=220 nm (0to60 MeCN) with ascorbic acid as standard at ≈3.95 min.

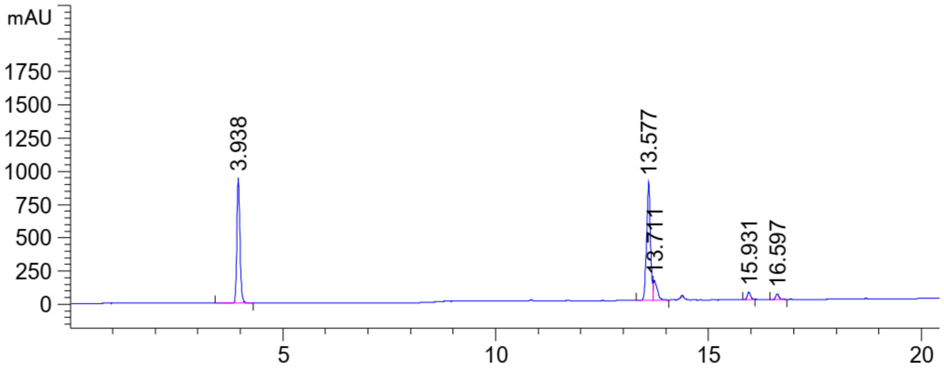

Figure S531: HPLC chromatogram of H-VKDGYI-NH_2 **70** after 3h with 1M NaOH in ethanol at λ=220 nm (0to60 MeCN) with ascorbic acid as standard at ≈3.95 min.

3.8.3. HPLC data of H-VKDGYI-OH 70 after 16h incubation with different bases

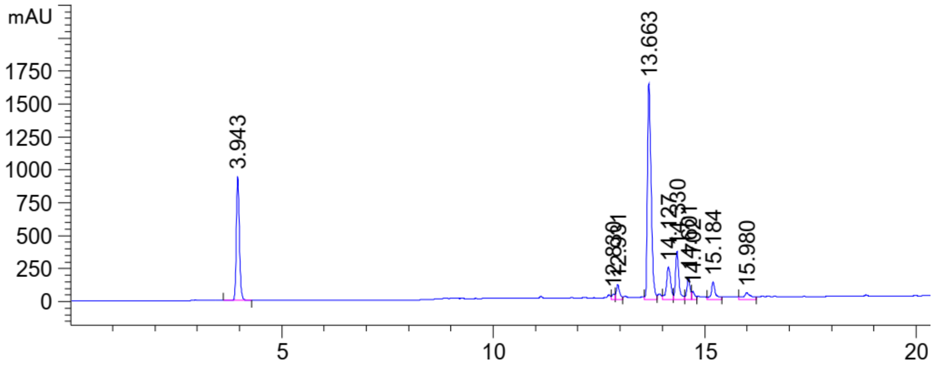

Figure S532: HPLC chromatogram of H-VKDGYI-NH_2 **70** after 16h with 5% piperazine in DMF at λ=220 nm (0to60 MeCN) with ascorbic acid as standard at ≈3.95 min.

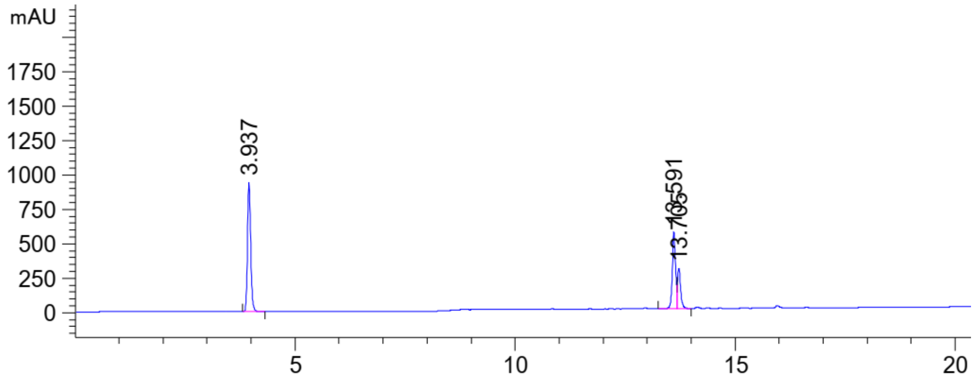

Figure S533: HPLC chromatogram of H-VKDGYI-NH_2 **70** after 16h with 5% piperazine in water at λ=220 nm (0to60 MeCN) with ascorbic acid as standard at ≈3.95 min.

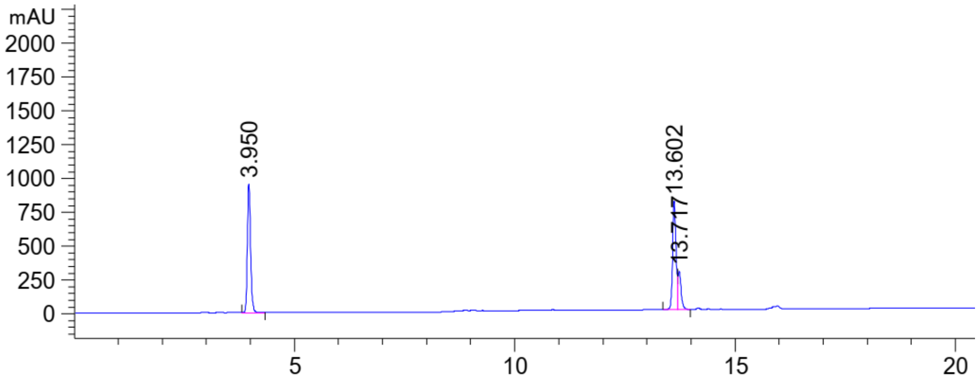

Figure S534: HPLC chromatogram of H-VKDGYI-NH_2 **70** after 16h with 10% ethanolamine in water at λ=220 nm (0to60 MeCN) with ascorbic acid as standard at ≈3.95 min.

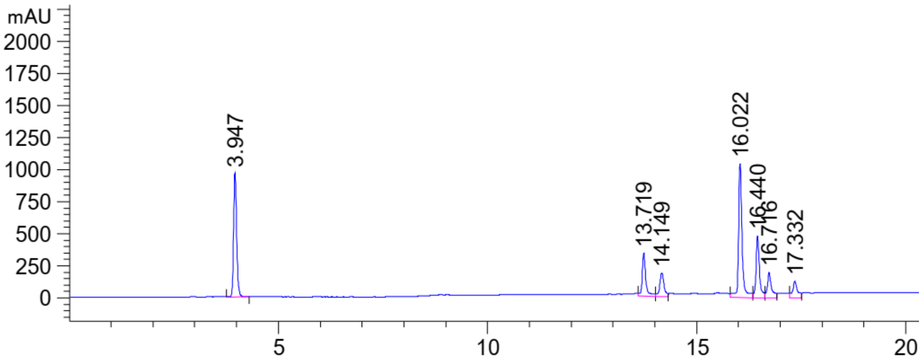

Figure S535: HPLC chromatogram of H-VKDGYI-NH_2 **70** after 16h with 20% piperidine in DMF at λ=220 nm (0to60 MeCN) with ascorbic acid as standard at ≈3.95 min.

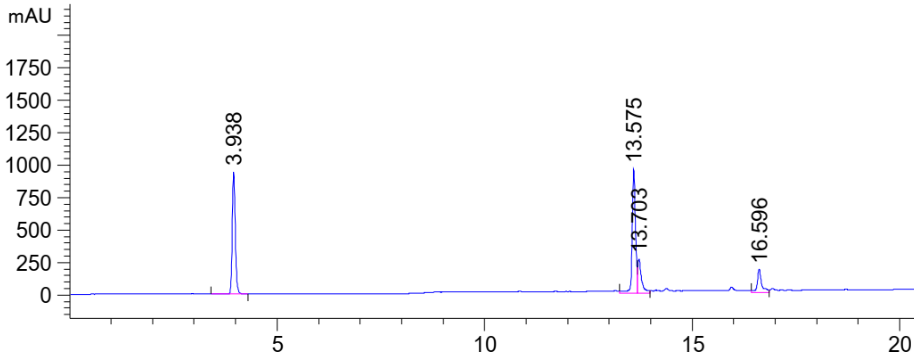

Figure S536: HPLC chromatogram of H-VKDGYI-NH_2 **70** after 16h with 1M NaOH in water at λ=220 nm (0to60 MeCN) with ascorbic acid as standard at ≈3.95 min.

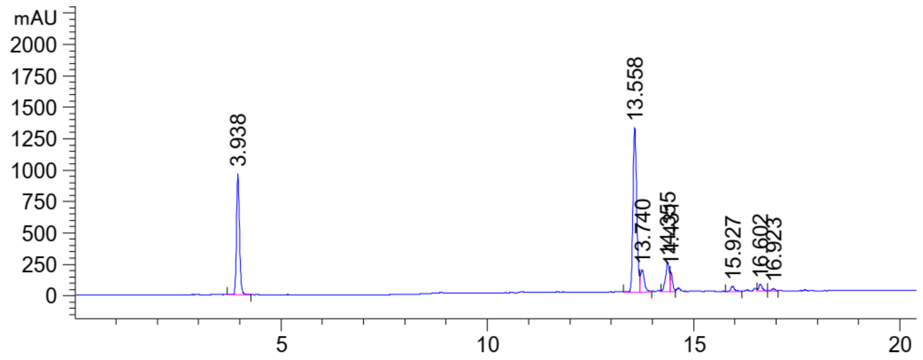

Figure S537: HPLC chromatogram of H-VKDGYI-NH_2 **70** after 16h with 1M NaOH in ethanol at λ=220 nm (0to60 MeCN) with ascorbic acid as standard at ≈3.95 min.

3.8.4. HPLC data of H-VK(D-D)GYI-NH_2 71 after 3h incubation with different bases

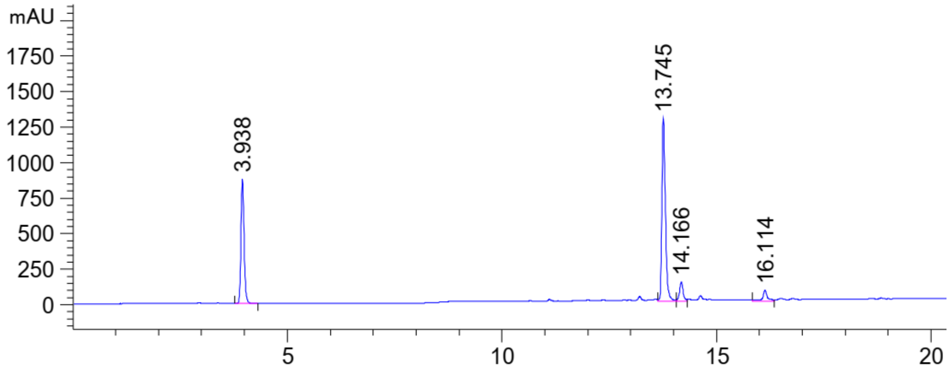

Figure S538: HPLC chromatogram of H-VK(D-D)GYI-NH_2 **71** after 3h with 5% piperazine in DMF at λ=220 nm (0to60 MeCN) with ascorbic acid as standard at ≈3.95 min.

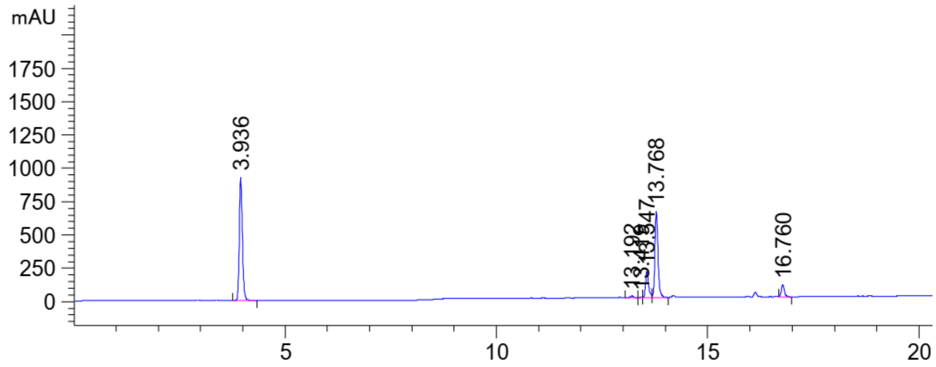

Figure S539: HPLC chromatogram of H-VK(D-D)GYI-NH_2 **71** after 3h with 5% piperazine in water at λ=220 nm (0to60 MeCN) with ascorbic acid as standard at ≈3.95 min.

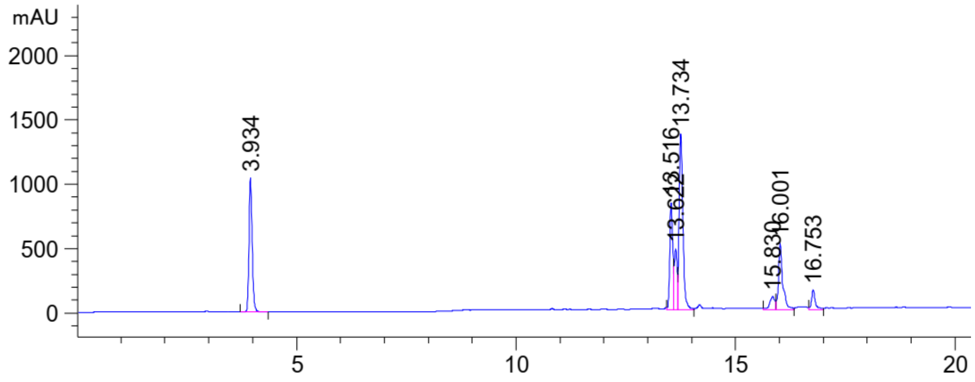

Figure S540: HPLC chromatogram of H-VK(D-D)GYI-NH_2 **71** after 3h with 10% ethanolamine in water at λ=220 nm (0to60 MeCN) with ascorbic acid as standard at ≈3.95 min.

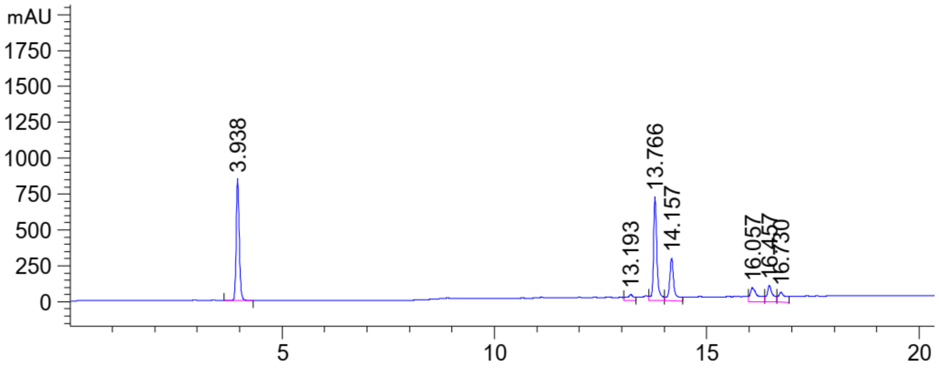

Figure S541: HPLC chromatogram of H H-VK(D-D)GYI-NH2 **71** after 3h with 20% piperidine in DMF at λ=220 nm (0to60 MeCN) with ascorbic acid as standard at ≈3.95 min.

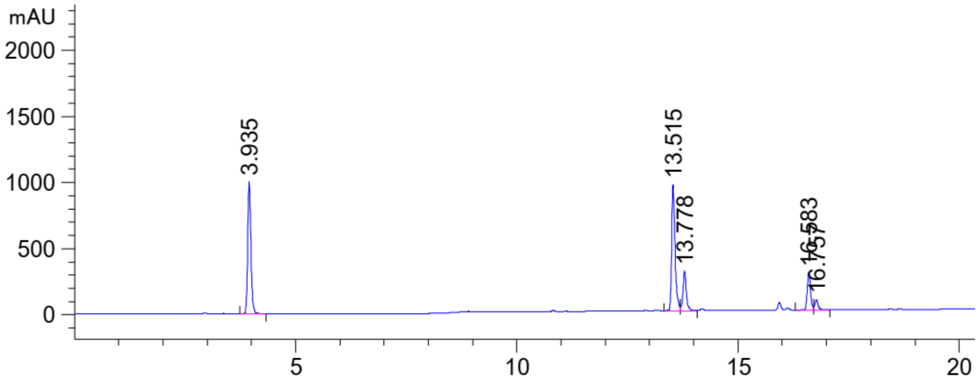

Figure S542: HPLC chromatogram of H-VK(D-D)GYI-NH2 **71** after 3h with 1M NaOH in water at λ=220 nm (0to60 MeCN) with ascorbic acid as standard at ≈3.95 min.

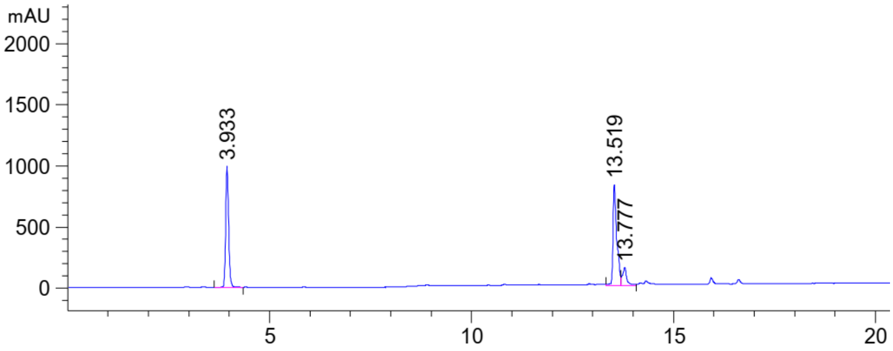

Figure S543: HPLC chromatogram of H-VK(D-D)GYI-NH2 **71** after 3h with 1M NaOH in ethanol at λ=220 nm (0to60 MeCN) with ascorbic acid as standard at ≈3.95 min.

3.8.5. HPLC data of H-VK(D-D)GYI-NH2 71 after 16h incubation with different bases

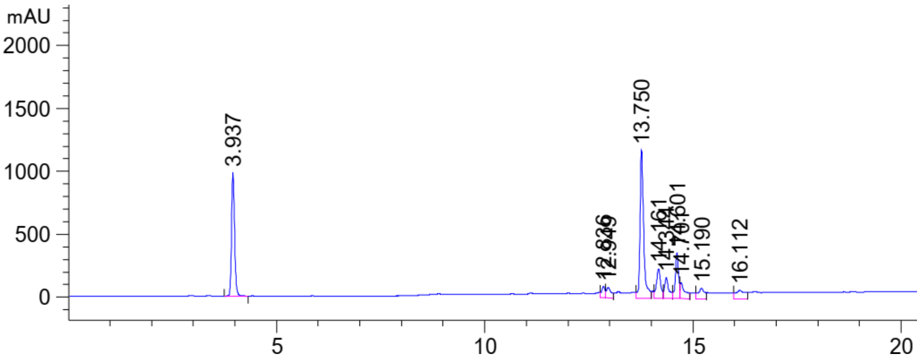

Figure S544: HPLC chromatogram of H-VK(D-D)GYI-NH2 **71** after 16h with 5% piperazine in DMF at λ=220 nm (0to60 MeCN) with ascorbic acid as standard at ≈3.95 min.

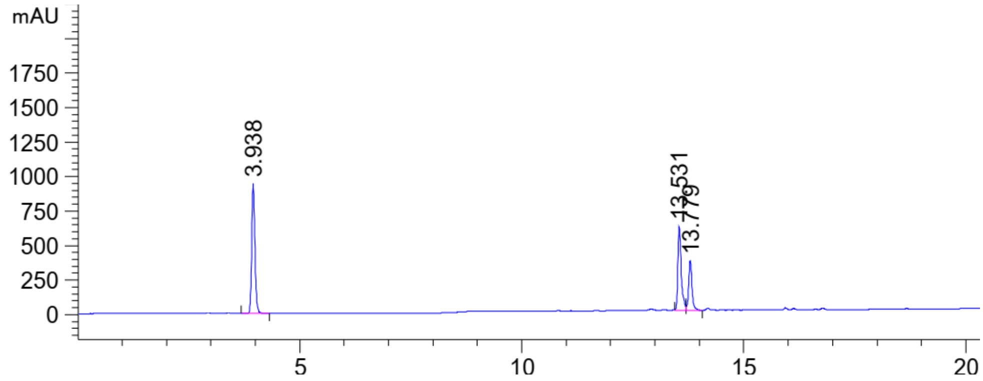

Figure S545: HPLC chromatogram of H-VK(D-D)GYI-NH_2 **71** after 16h with 5% piperazine in water at λ=220 nm (0to60 MeCN) with ascorbic acid as standard at ≈3.95 min.

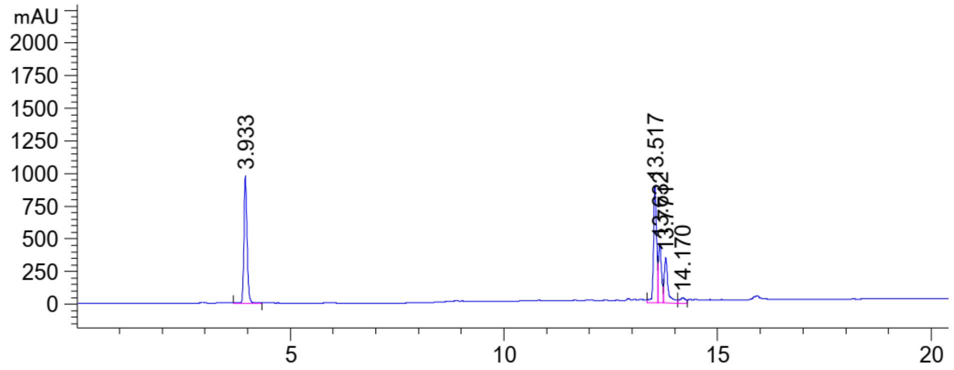

Figure S546: HPLC chromatogram of H-VK(D-D)GYI-NH_2 **71** after 16h with 10% ethanolamine in water at λ=220 nm (0to60 MeCN) with ascorbic acid as standard at ≈3.95 min.

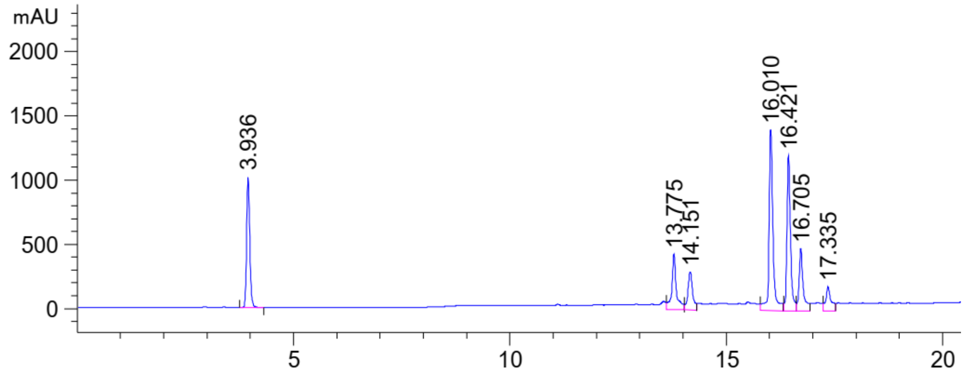

Figure S547: HPLC chromatogram of H-VK(D-D)GYI-NH_2 **71** after 16h with 20% piperidine in DMF at λ=220 nm (0to60 MeCN) with ascorbic acid as standard at ≈3.95 min.

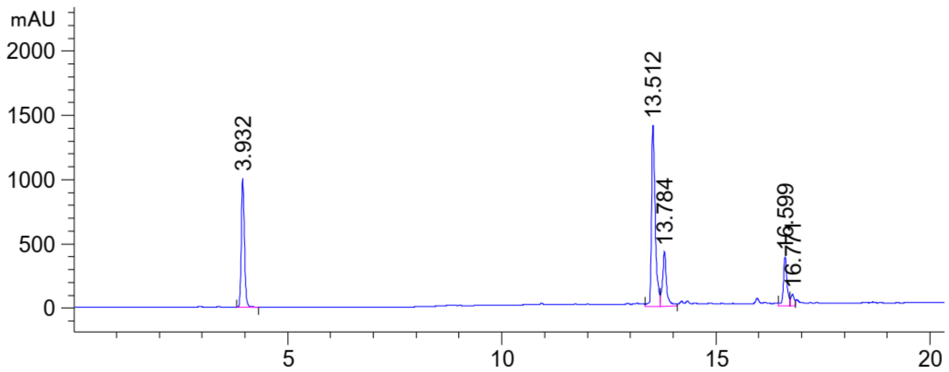

Figure S548: HPLC chromatogram of H-VK(D-D)GYI-NH_2 **71** after 16h with 1M NaOH in water at λ=220 nm (0to60 MeCN) with ascorbic acid as standard at ≈3.95 min.

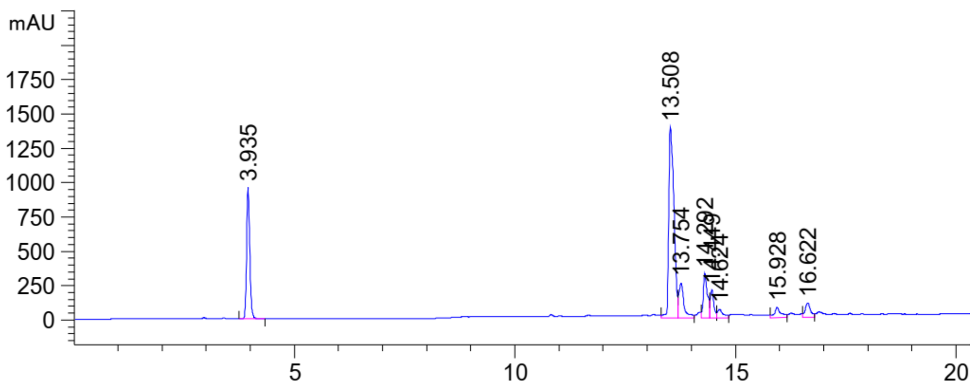

Figure S549: HPLC chromatogram of H-VK(D-D)GYI-NH_2 **71** after 16h with 1M NaOH in ethanol at λ=220 nm (0to60 MeCN) with ascorbic acid as standard at ≈3.95 min.

3.8.6. HPLC data of H-VKNGYI-NH_2 72 after 3h incubation with different bases

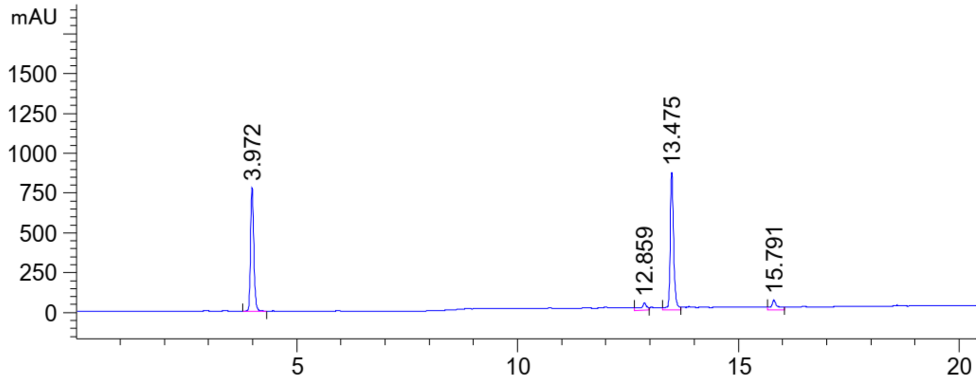

Figure S550: HPLC chromatogram of H-VKNGYI-NH_2 **72** after 3h with 5% piperazine in DMF at λ=220 nm (0to60 MeCN) with ascorbic acid as standard at ≈3.95 min.

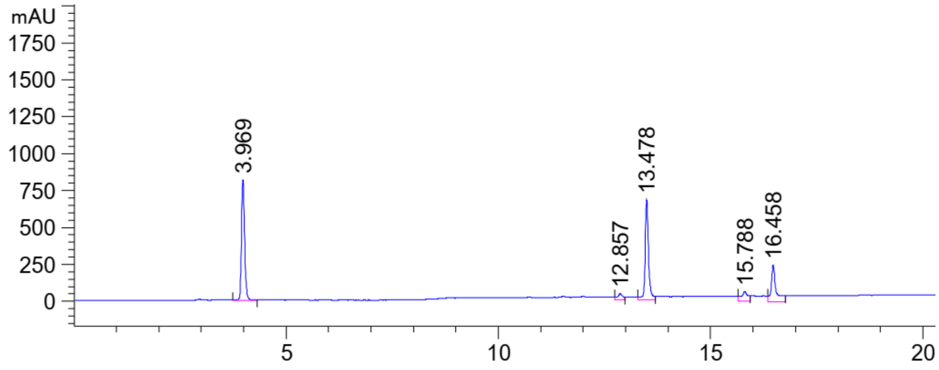

Figure S551: HPLC chromatogram of H-VKNGYI-NH_2 **72** after 3h with 5% piperazine in water at λ=220 nm (0to60 MeCN) with ascorbic acid as standard at ≈3.95 min.

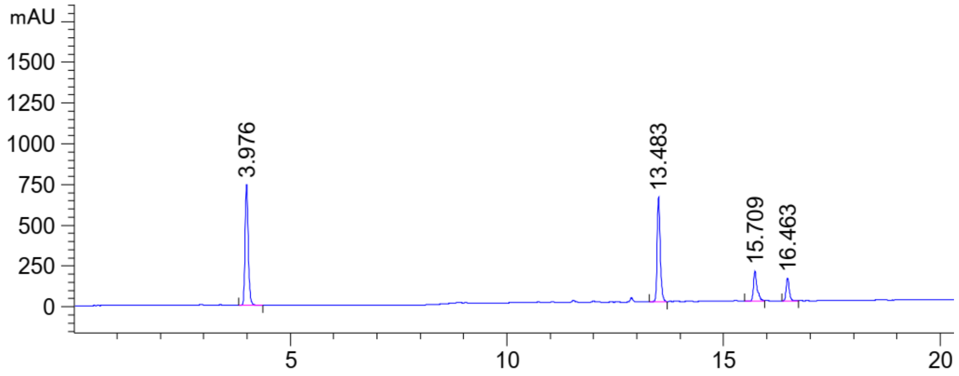

Figure S552: HPLC chromatogram of H-VKNGYI-NH_2 **72** after 3h with 10% ethanolamine in water at λ=220 nm (0to60 MeCN) with ascorbic acid as standard at ≈3.95 min.

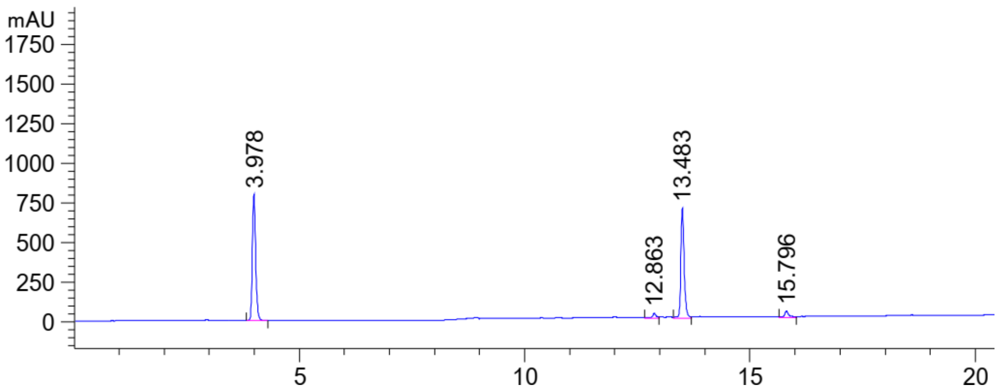

Figure S553: HPLC chromatogram of H H-VKNGYI-NH_2 **72** after 3h with 20% piperidine in DMF at λ=220 nm (0to60 MeCN) with ascorbic acid as standard at ≈3.95 min.

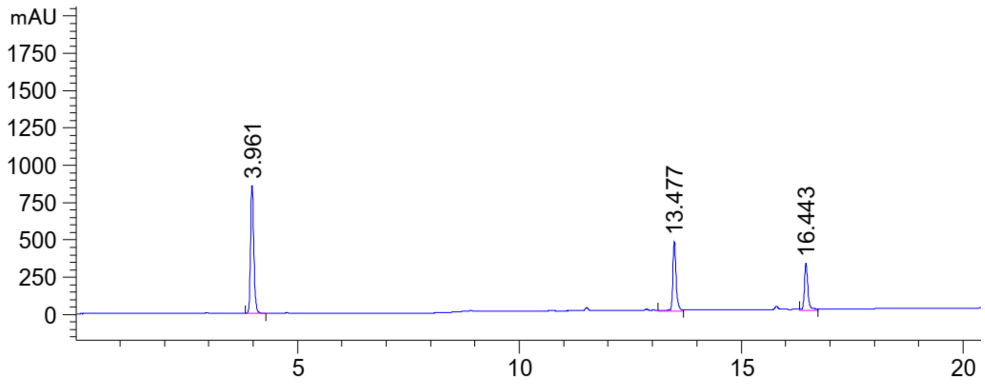

Figure S554: HPLC chromatogram of H-VKNGYI-NH_2 **72** after 3h with 1M NaOH in water at λ=220 nm (0to60 MeCN) with ascorbic acid as standard at ≈3.95 min.

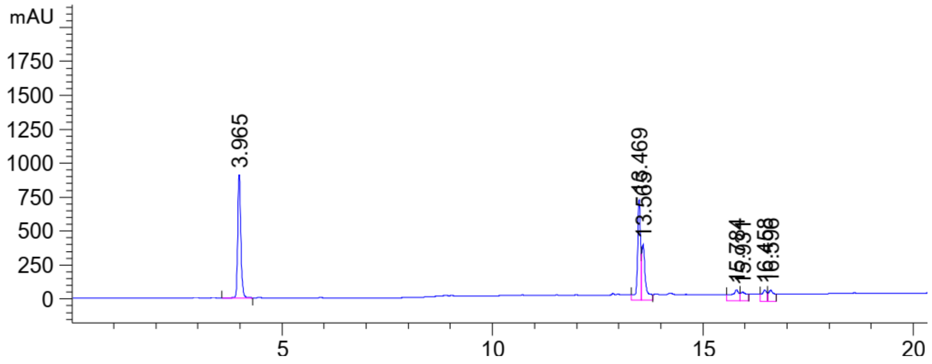

Figure S555: HPLC chromatogram of H-VKNGYI-NH_2 **72** after 3h with 1M NaOH in ethanol at λ=220 nm (0to60 MeCN) with ascorbic acid as standard at ≈3.95 min.

3.8.7. HPLC data of H-VKNGYI-NH_2 72 after 16h incubation with different bases

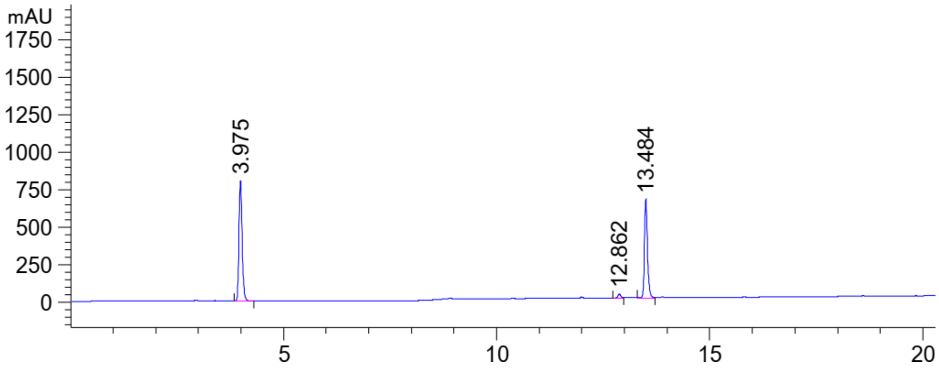

Figure S556: HPLC chromatogram of H-VKNGYI-NH_2 **72** after 16h with 5% piperazine in DMF at λ=220 nm (0to60 MeCN) with ascorbic acid as standard at ≈3.95 min.

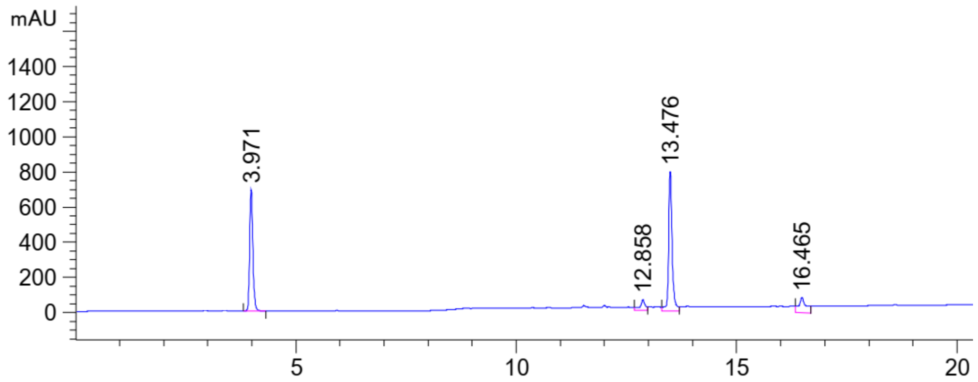

Figure S557: HPLC chromatogram of H-VKNGYI-NH_2 **72** after 16h with 5% piperazine in water at λ=220 nm (0to60 MeCN) with ascorbic acid as standard at ≈3.95 min.

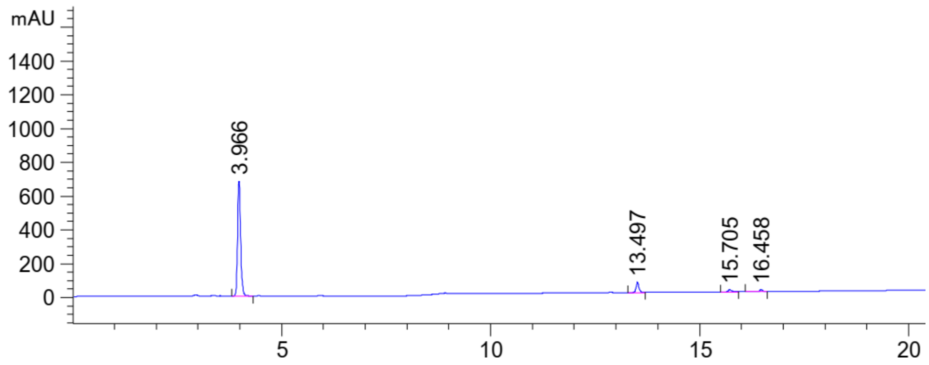

Figure S558: HPLC chromatogram of H-VKNGYI-NH_2 **72** after 16h with 10% ethanolamine in water at λ=220 nm (0to60 MeCN) with ascorbic acid as standard at ≈3.95 min.

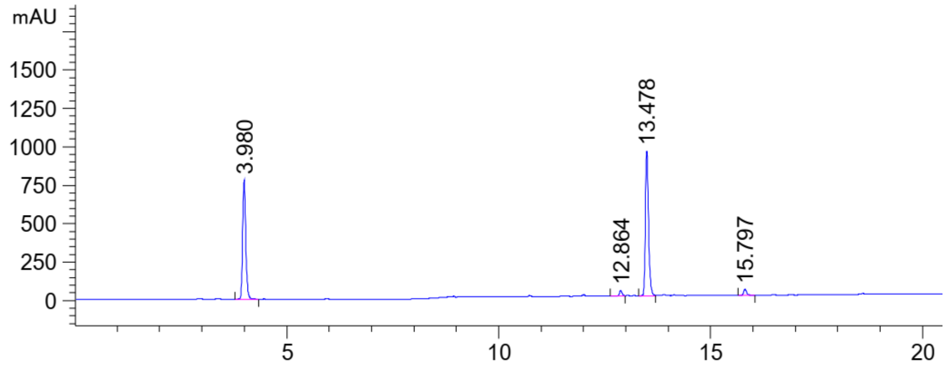

Figure S559: HPLC chromatogram of H-VKNGYI-NH_2 **72** after 16h with 20% piperidine in DMF at λ=220 nm (0to60 MeCN) with ascorbic acid as standard at ≈3.95 min.

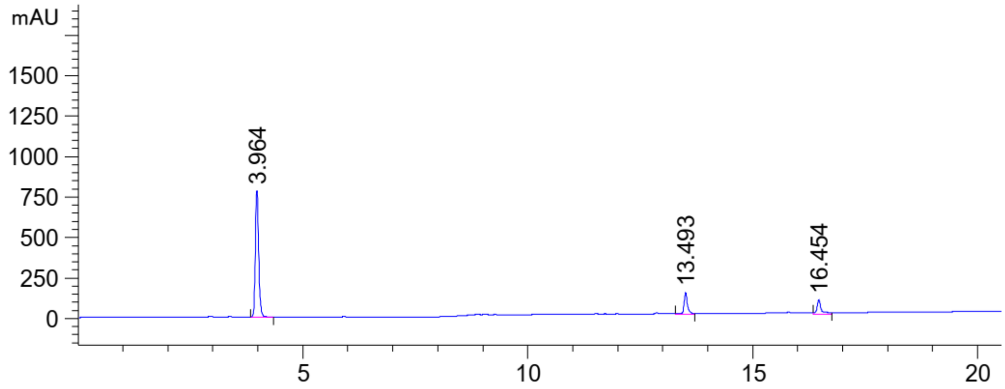

Figure S560: HPLC chromatogram of H-VKNGYI-NH_2 **72** after 16h with 1M NaOH in water at λ=220 nm (0to60 MeCN) with ascorbic acid as standard at ≈3.95 min.

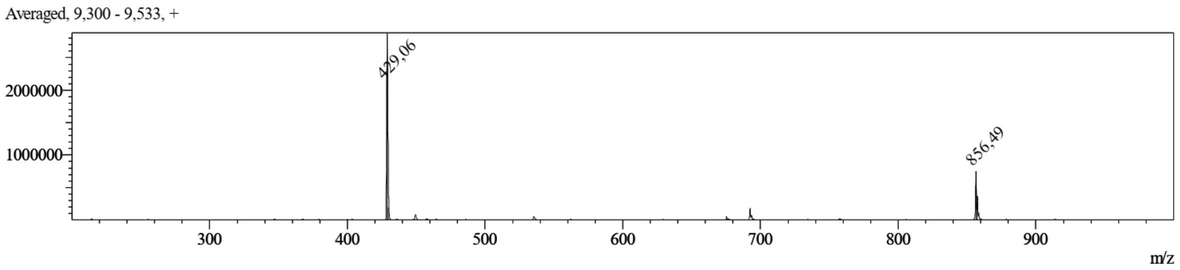

Figure S561: ESI-MS of H-VKNGYI-NH_2 **72** after 16h with 1M NaOH showing unidentified side product at 16.45min.

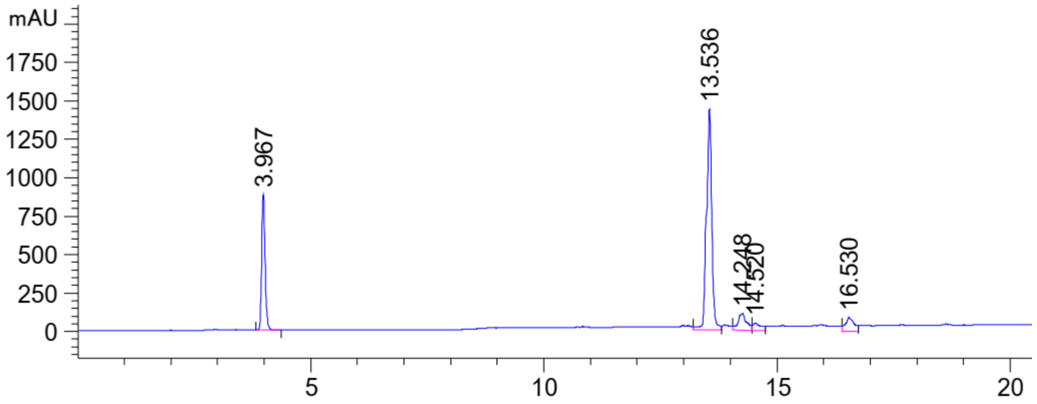

Figure S562: HPLC chromatogram of H-VKNGYI-NH_2 **72** after 16h with 1M NaOH in ethanol at λ=220 nm (0to60 MeCN) with ascorbic acid as standard at ≈3.95 min.

3.8.8. Temperature dependent formation of H-VK(β-D)GYI-NH_2 73 in water

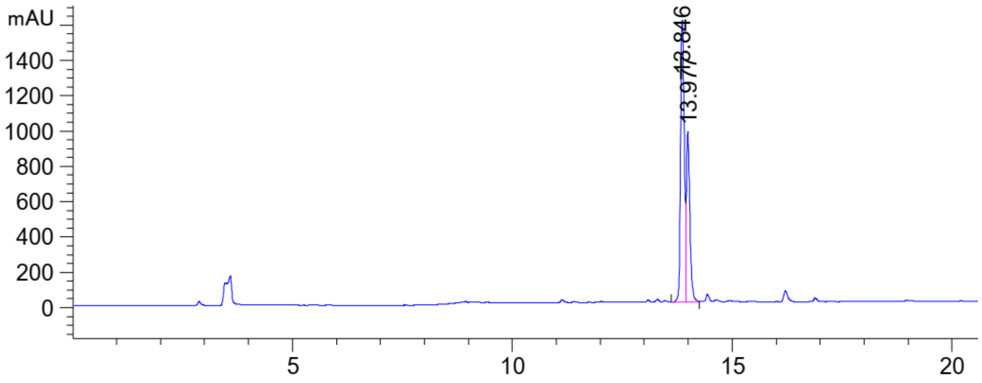

Figure S563: HPLC chromatogram of H-VK(β-D)GYI-NH_2 **73** formation at ambient temperature (26°C) in water at λ=220 nm (0to60 MeCN) with ascorbic acid as standard at ≈3.95 min.

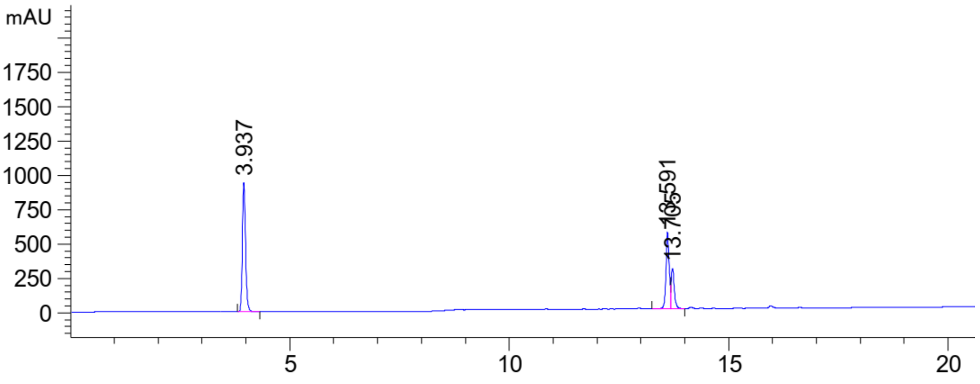

Figure S564: HPLC chromatogram of H-VK(β-D)GYI-NH_2 **73** formation at 40°C in water at λ=220 nm (0to60 MeCN) with ascorbic acid as standard at ≈3.95 min.

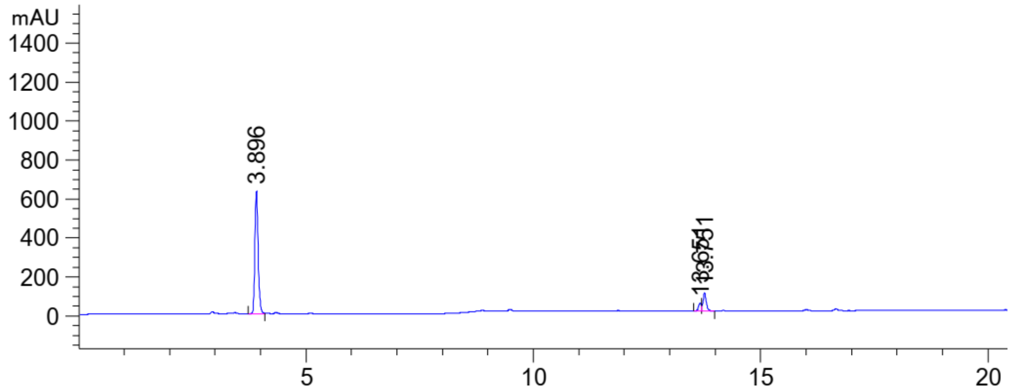

Figure S565: HPLC chromatogram of H-VK(β-D)GYI-NH2 **73** formation at 4°C in water at λ=220 nm (0to60 MeCN) with ascorbic acid as standard at ≈3.95 min.

3.9. HPLC data of the capping experiments

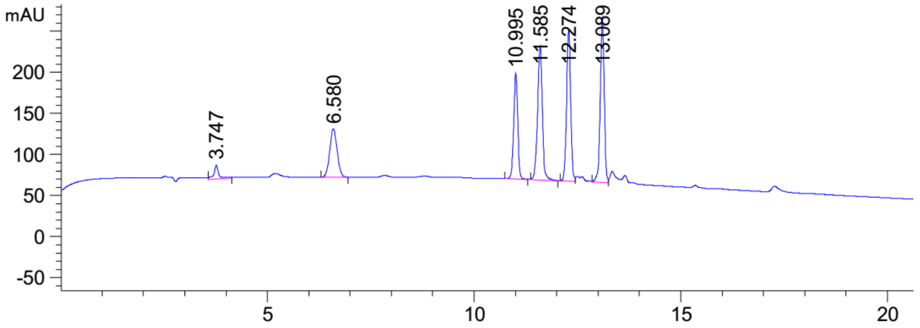

Figure S566: HPLC chromatogram of crude H-GPQGPQ-OH Hexapeptide 9 **49** in water using 0.95 eq. of *N*$_{\alpha}$-Smoc amino acid compared to prior coupling in order to maximize by-product formation, capping was performed with sulfoacetic acid **76** at λ=220 nm (0to40 MeCN).

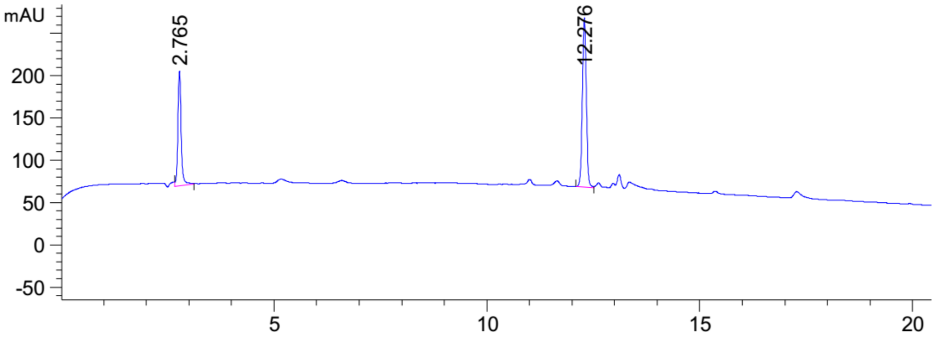

Figure S567: HPLC chromatogram of H-GPQGPQ-OH Hexapeptide 9 **49** after purification with IEC and the removal of all labelled side products at λ=220 nm (0to40 MeCN).

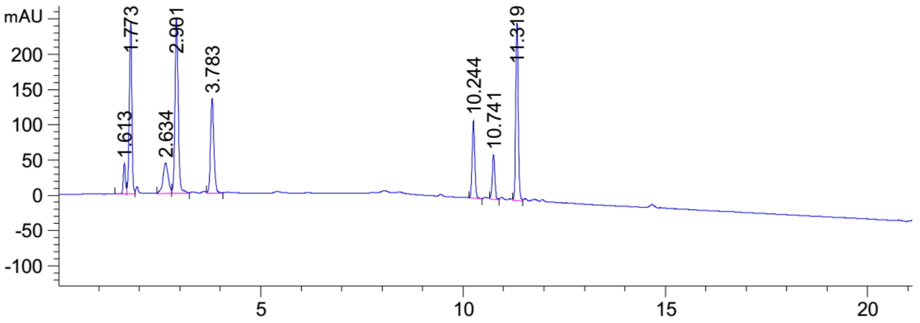

Figure S568: HPLC chromatogram of crude H-YGGFMRRV-NH2 **77** in DMF using 0.95 eq. of *N*$_{\alpha}$-Fmoc amino acid compared to prior coupling in order to maximize by-product formation, capping was performed with 4-sulfobenzoic acid **75** at λ=220 nm (10to60 MeCN).

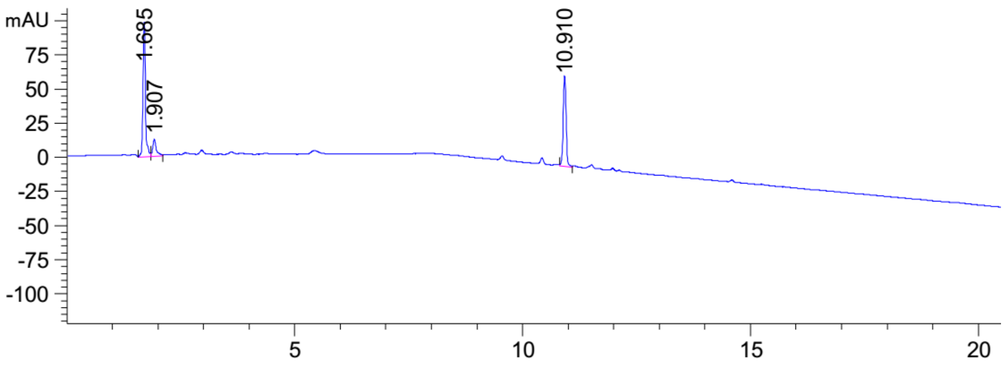

Figure S569: HPLC chromatogram of H-YGGFMRRV-NH2 **77** after purification with IEC and the removal of all labelled side products at λ=220 nm (10to60 MeCN).

References

[1] A. Ramazani, F. Zeinali Nasrabadi, A. Rezaei, M. Rouhani, H. Ahankar, P. Azimzadeh, S. Woo Joo, K. Slepokura, T. Lis, *Synthesis of N-acylurea derivatives from carboxylic acids and N,N'-dialkyl carbodiimides in water, Vol. 127*, **2015**.

[2] N. Fattahi, M. Ayubi, A. Ramazani, *Tetrahedron* **2018**, *74*, 4351-4356.

[3] R. Bollhagen, M. Schmiedberger, K. Barlos, E. Grell, *Journal of the Chemical Society, Chemical Communications* **1994**, 2559-2560.

[4] P. Palladino, D. A. Stetsenko, *Organic Letters* **2012**, *14*, 6346-6349.

[5] M. Quibell, D. Owen, L. C. Packman, T. Johnson, *Journal of the Chemical Society, Chemical Communications* **1994**, 2343-2344.

[6] L. C. Packman, *Tetrahedron Letters* **1995**, *36*, 7523-7526.

[7] J. Offer, M. Quibell, T. Johnson, *Journal of the Chemical Society, Perkin Transactions 1* **1996**, 175-182.

[8] M. Mergler, F. Dick, B. Sax, C. Stähelin, T. Vorherr, *Journal of Peptide Science* **2003**, *9*, 518-526.

[9] M. Mergler, F. Dick, B. Sax, P. Weiler, T. Vorherr, *Journal of Peptide Science* **2003**, *9*, 36-46.

[10] M. Mergler, F. Dick, *Journal of Peptide Science* **2005**, *11*, 650-657.

[11] S. A. Palasek, Z. J. Cox, J. M. Collins, *J Pept Sci* **2007**, *13*, 143-148.

[12] V. Cardona, I. Eberle, S. Barthélémy, J. Beythien, B. Doerner, P. Schneeberger, J. Keyte, P. D. White, *Int J Pept Res Ther* **2008**, *14*, 285-292.

[13] R. Röder, P. Henklein, H. Weißhoff, C. Mügge, M. Pätzel, U. Schubert, L. A. Carpino, P. Henklein, *Journal of Peptide Science* **2010**, *16*, 65-70.

[14] R. Subirós-Funosas, A. El-Faham, F. Albericio, *Tetrahedron* **2011**, *67*, 8595-8606.

[15] T. Michels, R. Dölling, U. Haberkorn, W. Mier, *Organic Letters* **2012**, *14*, 5218-5221.

[16] R. Behrendt, S. Huber, P. White, *Journal of Peptide Science* **2016**, *22*, 92-97.

[17] D. Samson, D. Rentsch, M. Minuth, T. Meier, G. Loidl, *Journal of Peptide Science* **2019**, *25*, e3193.

[18] K. Chandra, T. K. Roy, D. E. Shalev, A. Loyter, C. Gilon, R. B. Gerber, A. Friedler, *Angewandte Chemie International Edition* **2014**, *53*, 9450-9455.

[19] J. D. Wade, M. N. Mathieu, M. Macris, G. W. Tregear, *Letters in Peptide Science* **2000**, *7*, 107-112.

[20] J. Orpiszewski, N. Schormann, B. Kluve-Beckerman, J. J. Liepnieks, M. D. Benson, *FASEB journal : official publication of the Federation of American Societies for Experimental Biology* **2000**, *14*, 1255-1263.

[21] D. W. Aswad, M. V. Paranandi, B. T. Schurter, *Journal of Pharmaceutical and Biomedical Analysis* **2000**, *21*, 1129-1136.

[22] L. A. Carpino, *Accounts of Chemical Research* **1987**, *20*, 401-407.

[23] C. S. Nielsen, P. H. Hansen, A. Lihme, P. M. H. Heegaard, *Journal of Biochemical and Biophysical Methods* **1989**, *20*, 69-80.

[24] V. K. Sarin, S. B. H. Kent, J. P. Tam, R. B. Merrifield, *Analytical Biochemistry* **1981**, *117*, 147-157.

[25] R. S. Hodges, R. B. Merrifield, *Analytical Biochemistry* **1975**, *65*, 241-272.

[26] A. M. Felix, M. H. Jimenez, *Analytical Biochemistry* **1973**, *52*, 377-381.

[27] Brunfeld.K, Villemoe.P, Christen.T, *Febs Letters* **1972**, *22*, 238-&.

[28] O. Ludemann-Hombourger, *Speciality Chemicals Magazine* **2013**, 30-33.

[29] R. B. Merrifield, A. E. Bach, *The Journal of Organic Chemistry* **1978**, *43*, 4808-4816.

[30] A. Přibylka, V. Krchňák, E. Schütznerová, *Green. Chem*. **2019**, *21*, 775-779.

[31] T. Wang, S.J. Danishefsky, *PNAS*, **2013**, *110*, 11708-11713.
